# European Database of Carotenoid Levels in Foods. Factors Affecting Carotenoid Content

**DOI:** 10.3390/foods10050912

**Published:** 2021-04-21

**Authors:** M. Graça Dias, Grethe Iren A. Borge, Kristina Kljak, Anamarija I. Mandić, Paula Mapelli-Brahm, Begoña Olmedilla-Alonso, Adela M. Pintea, Francisco Ravasco, Vesna Tumbas Šaponjac, Jolanta Sereikaitė, Liliana Vargas-Murga, Jelena J. Vulić, Antonio J. Meléndez-Martínez

**Affiliations:** 1 Food and Nutrition Department, National Institute of Health Doutor Ricardo Jorge, IP, Av. Padre Cruz, 1649-016 Lisboa, Portugal; m.graca.dias@insa.min-saude.pt (M.G.D.); francisco.ravasco@insa.min-saude.pt (F.R.); 2 Nofima AS, Norwegian Institute of Food, Fisheries and Aquaculture Research, NO 1433 Ås, Norway; grethe.iren.borge@nofima.no; 3 Department of Animal Nutrition, Faculty of Agriculture, University of Zagreb, Svetošimunska Cesta 25, 10 000 Zagreb, Croatia; kkljak@agr.hr; 4 Institute of Food Technology in Novi Sad, University of Novi Sad, 21000 Novi Sad, Serbia; anamarija.mandic@fins.uns.ac.rs; 5 Food Colour & Quality Laboratory, Department of Nutrition & Food Science, Facultad de Farmacia, Universidad de Sevilla, 41012 Sevilla, Spain; pmapelli@us.es; 6 Institute of Food Science, Technology and Nutrition (ICTAN-CSIC), 28040 Madrid, Spain; bolmedilla@ictan.csic.es; 7 Chemistry and Biochemistry Department, University of Agricultural Sciences and Veterinary Medicine, 400372 Cluj-Napoca, Romania; apintea@usamvcluj.ro; 8 Faculty of Technology Novi Sad, University of Novi Sad, Bulevar cara Lazara 1, 21000 Novi Sad, Serbia; vesnat11@gmail.com (V.T.Š.); jvulic@uns.ac.rs (J.J.V.); 9 Department of Chemistry and Bioengineering, Vilnius Gediminas Technical University, 10223 Vilnius, Lithuania; jolanta.sereikaite@vgtu.lt; 10 Biothani Europe S.L. Can Lleganya, 17451 Sant Feliu de Buixalleu, Spain; lsvargas@biothani.com

**Keywords:** agro-food, agronomy, food composition, fruits and vegetables

## Abstract

Many studies indicate that diets including carotenoid-rich foods have positive effects on human health. Some of these compounds are precursors of the essential nutrient vitamin A. The present work is aimed at implementing a database of carotenoid contents of foods available in the European market. Factors affecting carotenoid content were also discussed. Analytical data available in peer-reviewed scientific literature from 1990 to 2018 and obtained by HPLC/UHPLC were considered. The database includes foods classified according to the FoodEx2 system and will benefit compilers, nutritionists and other professionals in areas related to food and human health. The results show the importance of food characterization to ensure its intercomparability, as large variations in carotenoid levels are observed between species and among varieties/cultivars/landraces. This highlights the significance of integrating nutritional criteria into agricultural choices and of promoting biodiversity. The uncertainty quantification associated with the measurements of the carotenoid content was very rarely evaluated in the literature consulted. According to the EuroFIR data quality evaluation system for food composition tables, the total data quality index mean was 24 in 35, reflecting efforts by researchers in the analytical methods, and less resources in the sampling plan documentation.

## 1. Introduction

The main dietary sources of carotenoids for humans are in general fruits and vegetables, although they are also present in other plant products (herbs, legumes, cereals or even oils), algae, animal foods, additives or supplements [1].

There are many studies indicating that carotenoid-rich diets can have a positive role in human health and contribute to reduce the risk of diseases associated with aging, such as certain cancers, cardiovascular disease, bone, skin or eye disorders and may be beneficial for mental and metabolic health, during pregnancy and early life. In addition to their role as colorants and bioactive compounds with possible positive effects on human health, some carotenoids also have a well-defined role as provitamin A nutrient, being key in combating vitamin A deficiency, especially in areas with low consumption of animal foods [2,3].

However, neither the European Food Safety Authority (EFSA) [4] nor the U.S. Food and Nutrition Board [5] have established official recommended daily intakes/nutritional reference values for the main dietary carotenoids. Indeed, there is still insufficient categorical evidence of the amounts needed to help promote health, even for a widely studied non-provitamin A carotenoid such as lutein, which has been positively associated to eye health and other benefits [6,7].

According to the European labeling requirement [8], the daily reference intake for vitamin A is 800 µg, with 15% (120 µg) being considered significant to the label’s nutritional table. The equivalence between carotenoids provitamin A and retinol is an issue on which a scientific consensus has not yet been established. According to the Food and Nutrition Board of the Institute of Medicine, in USA, for provitamin A carotenoids from food, 6 µg of β-carotene or 12 µg of α-carotene or 12 µg of β-cryptoxanthin are equivalent to 1 µg of retinol. In terms of activity, twice those amounts are needed to obtain that of 1 µg of retinol [9,10]. The first equivalence is named retinol equivalent (RE) and the second retinol activity equivalent (RAE). The Food and Agriculture Organization of the United Nations/International Network of Food Data Systems (FAO/INFOODS), adopted these equivalences with the note that they may be specific for each country [11]. The equivalence factors are estimated with large intervals of confidence due to factors that lead to high standard deviations, including large interindividual response, differences in food matrix and processing or analytical errors [12].

Apart from retinoids, carotenoids can also be enzymatically cleaved by humans into apocarotenoids and other derivatives, which are eliciting increasing interest, as they may be involved in biological actions. Given their versatility, carotenoids are importance continues growing in the era of sustainable healthy diets and for the development of innovative products such as novel foods or nutricosmetics, among others [2,3,13].

The promotion of currently underutilized local foods is increasingly a priority to improve environmental sustainability and reducing in particular, the carbon footprint as local foods have been often replaced by processed unhealthy foods due to its convenience. Frequently, mainly in low-income countries, the latter foods are produced far from the place of consumption having negative impacts in health and the environment. According to FAO, in 2019, more than 6000 plant species have been cultivated for food. Of these less than 200 make major contributions to food production globally, regionally or nationally and only nine species account for 66% of total crop production [14]. In this context, food composition databases are important tools for the adequate choice of foods and to promote biodiversity by highlighting neglected yet nutritionally rich species that lost importance with growing industrialization.

Food composition data are used in different fields of knowledge, mainly in the areas of agro-food, nutrition and health [15]. They are often the basis of studies that link the ingestion of carotenoids with health outcomes, so the quality of the data is of particular importance. The uncertainty of these data influences that of studies made from those and their conclusions drastically. This is not positive not only in scientific terms but also in order to support the implementation of public health policies.

The determination of the different components of foodstuffs is particularly time consuming and expensive due to their diversity and the different analytical methods involved. Carotenoids are particularly difficult to analyze accurately due to their lipophilicity, instability, structural similarity or scarcity of certified reference materials, which poses problems of accuracy and comparability of results. On the other hand, there is a clear dispersion of food carotenoids composition data. In general, food composition databases only include β-carotene, the carotenoid with the highest theoretical vitamin A activity and probably the most ubiquitous in foods [1,2].

In general, variations in the profile of carotenoids within a species are not observed with the naked eye unless there are color mutants. However, great variations are perceived in each carotenoid content and in the total carotenoid content in the different varieties of a species. This variability requires high resources in the design and implementation of sampling plans and highly specialized analytical and human resources for obtaining high quality data.

Several original data quality assessment systems were developed by several entities, such as the United States Department of Agriculture (USDA), the Agence Française de Sécurité des Produits Alimentaires (AFSSA), the Bioactive Substances in Food Information Systems (BASIS), the Centro per lo Studio e la Prevenzione Oncologica (CSPO) and the German Food Code and Nutrient Data Base (BLS). More recently the European Food Information Resource (EuroFIR) developed a system based on those in order to harmonize food composition data evaluation, by compilers [16]. Although its importance and usefulness are indisputable it is still not widely. Regarding carotenoids the first works on data quality assessment dates back to 1993, although only the analytical steps were considered [17,18].

This work is aimed at developing a comprehensive database of analytical data on carotenoid contents in plant foods produced/marketed in Europe reported in peer reviewed primary scientific sources. The quality of the data was assessed following current EuroFIR AISBL (International Non-Profit Organization) recommendations for compilers [16]. On the other hand, factors influencing carotenoid levels in foods are discussed.

## 2. Material and Methods

### 2.1. Data Collection

The identification of the papers for data collection was done through the b-on (Online Knowledge Library), a search engine that pre index metadata in a single central index. It contains over 16,750 scientific international publications from 16 publishers, through subscriptions negotiated on a national (Portugal) basis with these publishers.

The following keywords were searched in titles: carotenoid OR carotene OR xanthophyll OR lutein OR zeaxanthin OR cryptoxanthin OR lycopene OR phytoene OR phytofluene. Thereafter filters related to European countries were applied: Albania OR Armenia OR Austria OR Azerbaijan OR Belarus OR Belgium OR Bosnia OR Bulgaria OR Croatia OR Cyprus OR Czech Republic OR Denmark OR Estonia OR Europe* OR Finland OR France OR Georgia OR Germany OR Greece OR Hungary OR Iceland OR Ireland OR Italy OR Kazakhstan OR Kosovo OR Latvia OR Liechtenstein OR Lithuania OR Luxembourg OR Macedonia OR Malta OR Moldova OR Monaco OR Montenegro OR Netherlands OR Norway OR Poland OR Portugal OR Romania OR Russia OR Serbia OR Slovakia OR Slovenia OR Spain OR Sweden OR Switzerland OR Turkey OR Ukraine OR United Kingdom OR UK were searched in all fields. From this search, using filters, papers containing Food OR Vegetable OR Fruit were selected. Papers about biological fluids and food of animal origin were excluded manually.

The research was conducted in papers published in the period 1990–2018 and only original research full-text papers were scrutinized. Only analytical data obtained by HPLC or UHPLC systems equipped with at least spectrophotometric detector were considered. The use or not of the saponification step during extraction method was registered since this step affects the analytical measurement results [1]. Only food items intended for human consumption produced and/or marketed in Europe were considered. Food supplements were not in the scope of this collection.

Each paper was analyzed by researchers working in the area of carotenoids, in particular with knowledge of the analytical methods. All carotenoids reported in each paper were collected and the water content, when available. References to carotenoids content expressed as RE, RAE and % of the total, presented only in the graphic form and indicating only the minimum and maximum were discarded. The different units referred in the papers were converted to µg/100 g. The tables were organized to document the food, common name, scientific name including species and varieties/cultivars/landraces/accessions (whenever known). Other descriptors, including countries of origin and purchase, general food processing method, color and part analyzed, were also registered when available.

All foods items were classified according to the FoodEx2 hierarchical system developed by the European Food Safety Authority (EFSA), at least at level 1 (twenty food groups). When the number of foods at a given level was high, lower levels were used in order to distinguish them. That is, being a hierarchical system, when the group level comprised a large amount of foods (for instance, in the case of fruits and vegetables), the main sources of carotenoids, levels 3 and 4 were reached in order to obtain a greater distinction between the foods of these groups. Facet descriptors were used to provide additional information for processed foods.

### 2.2. Data Quality Assessment

The quality of data published was evaluated using the methodology developed in the scope of EuroFIR. This includes the evaluation of the measurement and the sampling methods [16]. Briefly, this method evaluates seven categories: food description, component identification, sampling plan, number of analytical samples, sample handling, sample analysis and analytical quality control. Quality was scored in each one by answering a total of 28 predefined questions (criteria). For each category several criteria were defined and for each one the evaluator should answer “Yes”, “No” or “Not Applicable” (when the criterion is not relevant for the nutrient in appreciation). These answers were translated quantitatively, accordingly the following answers points scheme: “Yes”, 5 points; “No” and “Not Applicable”, 0 points. The total points in each category were divided by the total number of “Yes” and “No”; by definition when one category gets only “No” or “Not Applicable” answers, the score should be 1. Through this evaluation, each category received a score between 1 (low quality) and 5 (high quality). At the end, the scores of each category were added and the overall quality index ranged between 7 (low quality) and 35 (high quality). The evaluation was done for all papers collected and, for each category and overall quality index, means were calculated.

## 3. Results and Discussion

### 3.1. Data Collection

The search tools of b-on was not selective enough and a general review of the abstracts resulting from the search, which returned initially >1000 results, was necessary. After this review, 373 references were collected and from these 25 were rejected, the main cause being the determination of carotenoids by spectrophotometric method instead of HPLC/UHPLC. The time frame was from 1990 to 2018, with emphasis on the most recent years. The year with the most papers was 2016. The references were from Archives of Biochemistry and Biophysics, Crop Science, European Food Research and Technology, Food and Nutrition Research, Food Chemistry, Food Research International, Food Science and Technology International, International Journal of Food Science and Technology, International Journal of Food Sciences and Nutrition, Journal of Agricultural and Food Chemistry, Journal of Chromatography A, Journal of Food Composition and Analysis, Journal of Food Science, Journal of Food, Agriculture and Environment, Journal of Separation Science, Journal of the Science of Food and Agriculture, LWT-Food Science and Technology, Nutrición Hospitalaria, Plant Foods for Human Nutrition, Planta, Postharvest Biology and Technology, Archivos Latinoamericanos de Nutrición, Food and Nutrition Bulletin, Revista do Instituto Adolfo Lutz and Scientia Horticulturae. Although b-on has a high journal coverage, digital object identifier (DOI) was not available in some of the oldest references, forcing a new internal referencing of these papers in order to create a unique identification.

To facilitate comparisons, all carotenoid content units were converted to µg/100 g, preferably in fresh weights, unless water content was not given, and in this case, the dry basis was flagged. The vast majority of papers did not report water content despite this parameter is key in order to be able to relate compositions in food composition databases.

The information about the use or not of saponification during the analytical process was collected as this reaction can lead to important losses of carotenoids or even the formation of artifacts, decreasing the accuracy of the analysis. To monitor this process, an internal standard is often added by some researchers, for example β-apo-8′-carotenal or echinenone. Despite being a lengthy step, saponification is often essential to hydrolyze the carotenoid esters, to eliminate lipids and eliminate chlorophylls [1,2]. However, only 10% of the papers refer the inclusion/not inclusion of this step.

In general, the part of the food analyzed was mentioned in the papers. The descriptor “edible part” was used very often. However, in foods that may generate ambiguities, a more detailed description will be an asset. This is the case, for example, of apple, pear, tomato and apricot, in which the peel may or may not be considered an edible part, influencing the carotenoid content.

All food items were codified by FoodEx2, using the exposure hierarchy, including facets for processed food. This classification system, initially developed for exposure evaluation to contaminants, is currently increasingly used in the area of food composition and mandatory in projects supported by EFSA. It is less complex in relation to other systems (e.g., LanguaL) and it is used by EFSA to facilitate the compatibility/comparability among different domains of food databases and from different countries at the EU level.

Supplementary Tables S1–S10 [19,20,21,22,23,24,25,26,27,28,29,30,31,32,33,34,35,36,37,38,39,40,41,42,43,44,45,46,47,48,49,50,51,52,53,54,55,56,57,58,59,60,61,62,63,64,65,66,67,68,69,70,71,72,73,74,75,76,77,78,79,80,81,82,83,84,85,86,87,88,89,90,91,92,93,94,95,96,97,98,99,100,101,102,103,104,105,106,107,108,109,110,111,112,113,114,115,116,117,118,119,120,121,122,123,124,125,126,127,128,129,130,131,132,133,134,135,136,137,138,139,140,141,142,143,144,145,146,147,148,149,150,151,152,153,154,155,156,157,158,159,160,161,162,163,164,165,166,167,168,169,170,171,172,173,174,175,176,177,178,179,180], corresponding to 10 level 1 FoodEX2 groups, Coffee, cocoa, tea and infusions; Seasoning, sauces and condiments; Composite dishes; Fruit and vegetable juices and nectars; Animal and vegetable fats and oils and primary derivatives thereof; Fruit and fruit products; Legumes, nuts, oilseeds and spices; Starchy roots or tubers and products thereof, sugar plants; Vegetables and vegetable products; Grains and grain-based products present the carotenoid content of food items. Sub tables by FoodEX2 subgroups (levels 2–4) and the respective codes, including facets (represented by #) were also used to obtain more discrimination and facilitate consultation.

The lack of quantitative data for a carotenoid in the tables meant in many cases that the carotenoid was not reported in the paper and not that the food did not contain that carotenoid. This has been common for the colorless carotenoids phytoene and phytofluene, which have been very often ignored [2].

Supplementary Tables S1–S10 contain 3507 values of carotenoid content, with 29.4% referring to β-carotene, 18.8% to lutein, 10.3% to β-cryptoxanthin, 8.8% to zeaxanthin, 7.0% to α-carotene and 3.9% to lycopene.

Excluding foods analyzed in their dry form, and according to this collection, the major sources of the carotenoids above were, in µg/100 g fresh weight, in descending order (extracts of Tables S1–S10 for some of these foods area presented on Appendix A):

– Carrot, spinach, goosefoot, peppers (red) and sheep sorrel for β-carotene.
– Spinach, goosefoot, sheep sorrel, Indian spinach and amaranth for lutein.
– Peppers (red), apricot, sarsaparilla, tamarillo and mandarin for β-cryptoxanthin.
– Peppers (red), goosefoot, duckweed, goji berries and maize for zeaxanthin.
– Carrot, pumpkin, carrot greens, cowpea and Ceylon spinach for α-carotene.
– Tomato, rosehip, ketchup, watermelon and tomato sauce for lycopene.

### 3.2. Data Quality Index

**Figure 1:**
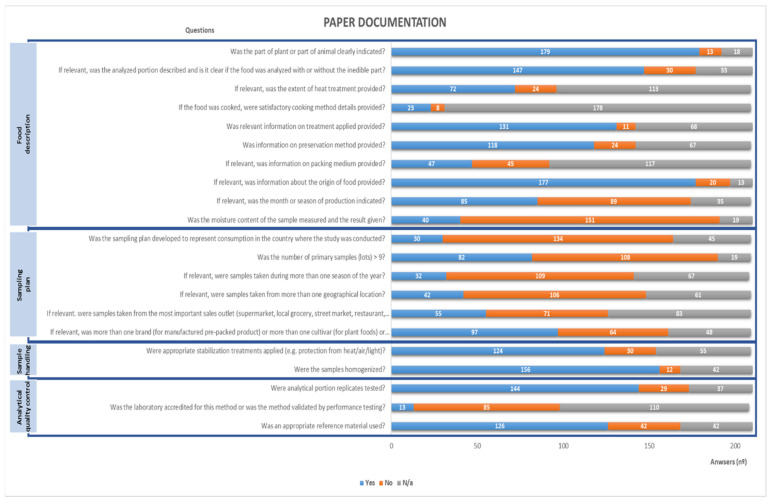
Paper documentation evaluation, for food composition databases, by the EuroFIR system.

Sample analysis and the component identification obtained immediately the maximum score, which was 5. Firstly, since HPLC (or more recently UHPLC) is the method generally accepted as suitable for the analysis of individual carotenoids, only data obtained using it were considered for data collection. Additionally, only clear unequivocal identified and quantified (including units) carotenoids were included in the database. The score for the number of analytical samples was 1 due to the specification of this gathering per analytical sample. Indeed, this category was not evaluated. The results of the global evaluation of all papers are presented in Figure 1, excluding categories evaluated a priori based on the assumptions of this collection. Considering the set of papers reviewed, the average scores obtained were 4 for the Food description, 5 for the Component identification, 2 for the Sampling plan, 1 for the Number of analytical samples, 4 for the Sample handling, 5 for the Sample analysis and 3 for the Analytical quality control. Considering that seven categories were under evaluation and that each one can get a maximum score of 5, overall, a mean rating of 24 out of a possible maximum of 35 was obtained by applying strictly the method. Considering the peculiarity of this collection it could be considered that a score of 23 out of 30 was obtained. Taking into account the EuroFIR criteria for quality of food composition databases, the results reveal that the data on carotenoids, available in the scientific literature present a greater weakness in terms of the sampling plan and then in terms of analytical control. This can be attributed to the difficulty in defining representative food sampling plans, the intention of the study (for instance the representativeness of the food in the country is not usually an important aspect) or the scarcity of certified reference materials/interlaboratory tests in this area. Another important aspect is that researchers from other areas may not be aware for the importance of these quality parameters and may not describe them in their publications.

#### 3.2.1. Factors Affecting the Level of Carotenoids

The data collected show great variations in the levels of carotenoids within and across plant foods (Tables S1–S10). All plant biosynthesize carotenoids in their photosynthetic tissues following a quite constant pattern in most cases, whereas many of them also biosynthesize a wider variety of them in structures such as fruits, several flower structures, tubers, seeds or roots [181,182]. Apart from the type of plant tissue there are manifold factors related to the accumulation of carotenoids in these foods, examples of some of which are outlined below. It is important to note that, because of the numerous factors governing the biosynthesis of carotenoids in plant foods, it is generally difficult to establish categorically the effect that a particular practice has on a given food. Thus, it is not uncommon to find in the bibliography that the effect of an agronomic practice is markedly different depending for instance on genetic factors or developmental stages.

#### 3.2.2. Factors Related to the Plant

##### Genetic Factors

The carotenoid profile in qualitative terms is usually quite constant across many varieties, although there can also be spontaneous or targeted color mutations that result in varieties with a distinctive carotenoid pattern. Thus, most sweet oranges (*Citrus sinensis* L. Osbeck) accumulate epoxycarotenoids (typically certain isomers of violaxanthin and antheraxanthin) as major carotenoids [55], whereas a few cultivars such as Cara Cara, Shara or Hong Anliu have a reddish flesh due to the accumulation of large amounts of lycopene (absent in most orange varieties) and also of the colorless carotenoids phytoene and phytofluene [52,183]. On the other hand, the variety Pinalate has a yellowish pulp resulting from the very high accumulation of ζ-carotene, phytoene and phytofluene, which are the main carotenoids in this variety [184]. There are also tomato color mutants, for instance the R tomato, which has yellowish flesh and does not accumulate carotenoids [185] or the Delta tomato, with increased δ-carotene levels (a carotenoid not usually detected in most tomato varieties) and reduced lycopene contents [186].

In some cases, structures that do not accumulate carotenoids or that accumulate very low amounts can become good sources of these compounds as a result of spontaneous mutations. One example would be the cauliflower, where a gene mutation results in the accumulation of high levels of β-carotene in the inflorescence and other structures [187]. The accumulation of carotenoids in previously non-carotenogenic structures can also be achieved by means of genetic engineering. The biosynthesis of β-carotene in the endosperm of golden rice being an excellent example [188].

On the other hand, there can be important differences in the carotenoid levels even within the same variety under the same cultivation conditions. Thus, for instance, in a study where the accumulation of carotenoids, tocopherols and phenolic compounds in diverse tomato and wild relatives was studied it was concluded that, when ripe, the highest levels of lycopene, phytoene and phytofluene were found in two accessions of the same tomato variety (var. cerasiforme). The differences ranged between ca. 2-fold (lycopene) and ca. 6-fold (phytoene), which highlights the impact that the genotype has in the accumulation of secondary metabolites [189].

##### Ripening

In carotenogenic fruits, ripening is normally accompanied by the degradation of chlorophylls and the increase in the biosynthesis of carotenoids although there are some exceptions. Thus, in fruits that remain green when ripe (kiwi) or those that owe their final color to anthocyanins (strawberries and other berries and olives) the carotenoid content can decrease over ripening [182].

##### Part of the Tissue

The carotenoid content is not normally evenly distributed in the plant structures. Indeed, it can vary considerably both longitudinally and transversally as it can be observed with the naked eye in foods like tomato fruits or carrots. Considering this is essential when generating compositional data on carotenoids [86]. A correct sampling procedure would involve, among many other steps, to quarter longitudinally the sample and then take and combine opposite sections [190].

##### Location of the Fruit in the Plant

The position of a carotenogenic fruit in the plant can have an important effect in their carotenoid levels. As an example, statistically significant differences in carotenoid contents have been reported in diverse tomato genotypes as a function of the location of the tomato clusters, although a consistent pattern of changes valid for all carotenoids and varieties was not observed. The authors argued that differences in the carotenoid content depending on the location could be due to differences in exposure to radiation [141], which is a factor known to affect carotenoid biosynthesis, as discussed below.

#### 3.2.3. Ambient Factors

##### Light Quality

It is well known that the spectral quality of the light that reaches the plant can have an impact in the biosynthesis of secondary metabolites. Thus, the use of shade nettings that allow for the selection of the desired spectral light is becoming an important technological concept in agronomy [191]. As an example, it has been reported that blue light can have a positive impact on the accumulation of lycopene and β-carotene in tomatoes [133]. On the other hand, short-term ultraviolet B (UV-B) irradiation on sweet basil did not lead to marked increases in the carotenoid levels of young plants and decreased them in flowering plants [192].

##### Light Quantity

The effect of high light exposure or intensity on the levels of not only carotenoids but also other secondary metabolites is thought to be positive in general [193]. For example, high light exposure can lead to increases in total carotenoids in the peel of apples [194] or in tomatoes [132].

Interestingly, the fine tuning dosage of blue light could be a good strategy to increase the chlorophyll and carotenoid levels of microgreens like parsley, mustard or beet, where increases in these compounds ranging from 1.2-fold to 4.3-fold have been observed [110].

##### Climate, Season and Geographic Site of Production

Climate is a complex phenomenon that is interrelated with factors including season or geographical location, among others.

Some studies carried out in Brazil with papayas [195], West Indian cherry [182] or mangoes [196] indicate that, in some cases, climatic factors can have a greater impact on the biosynthesis of carotenoids than cultivar differences and that greater exposure to sunlight and higher temperatures can elevate the carotenoid levels in some fruits. In some cases, 5–6-fold differences in the levels of carotenoids between different geographical regions were observed [182]. A similar conclusion was drawn from a study in which the carotenoid levels in diverse tomatoes grown in Ireland and Spain were compared [131].

Concerning seasonal effects, significant differences in the levels of both total and some individual carotenoids of kale have been reported, the total carotenoid content being higher in winter than in summer, 1.25-fold for cultivar cv. *Manteiga* [197] and 2-fold for galega v. *acephala* [32]. In a study in which the effect of regulated deficit irrigation, cluster, developmental stages and two seasons (autumn and spring) on the carotenoid levels of “Lazarino” and “Summerbrix” tomatoes was evaluated, it was concluded that, overall, lower levels of individual and total carotenoids were observed in autumn [198].

Features of climate changes, for instance the increase in temperature, the elevated atmospheric CO_2_ and the arrival of more UVB rays on Earth due to the decrease in the ozone layer seems to influence the content of carotenoids in food. In an experimental study the influence of temperature on total carotenoid content was evaluated and it was found that the total carotenoid content of five tomato varieties was lower at 35.4 °C than at 33.4 °C [199]. A meta-analysis conducted showed that in general an exposure of plants to high levels of CO_2_ decreased the content of carotenoids by 15%, with some exceptions, mainly when the plants were abiotically stressed, in which the concentration of carotenoids increased [200]. Plantains grown in the south hemisphere contained the more carotenoids provitamin A, α-carotene and β-carotene the greater the incidence of UVB rays [201].

#### 3.2.4. Agronomic Practices

##### Salinity Stress

Diverse treatments leading to salinity stress have been shown to increase lycopene and other carotenoid levels (in some cases up to 3-fold) in different tomato genotypes [127,134]. Salinity stress has also been shown to lead to elevated carotenoid contents in red pepper [202] and, more markedly, in romaine lettuce [103].

##### Water Deficit

The availability of water is an important problem in many parts of the world, so the importance of implementing practices leading to efficient water usage in agriculture continues growing. The effect of water stress or deficit irrigation on the commercial quality or other parameters, like the levels of secondary metabolites, is therefore a timely research topic. As an example, it has been observed that a regulated deficit irrigation with a reduction of 40–50% in the leaf water potential in some tomato varieties can lead to increases in the levels of not only carotenoids but also phenolic compounds without affecting significantly their commercial quality. More specifically, ca. 1.5-fold increases in lycopene and total carotenoid levels were observed in some cases [141]. Deficit irrigation has also been reported to reduce yield, but not lycopene and fruit quality in certain watermelon varieties [203]. Interestingly, grafting of mini-watermelon under irrigation deficit led to increases of lycopene levels ca. 40% compared to the ungrafted counterparts [204]. Grafting under water stress could also lead to increases in lycopene levels in cherry tomatoes [205]. Contrastingly, regulated deficit irrigation has also been shown to reduce carotenoid levels in peaches [206]. On the other hand, water stress has been reported to significantly reduce the levels of capsanthin in red peppers, the degree of reduction being related to the intensity of the stress [207].

##### Use of Agrochemicals

As part of an interesting study, the carotenoid levels of kales cv. “Manteiga” grown in a farm not using agrochemicals were compared with those grown in a neighboring farm that used glyphosate (herbicide), ethyl parathion (insecticide) and a leaf fertilizer containing nitrogen, phosphorus and potassium. The results indicated that the total carotenoid contents were significantly higher (1.2-fold) in the farm not using agrochemicals [182,198]. Significantly higher lycopene contents (ranging from 1.6-fold to 2-fold) were found in Rio Red grapefruits grown conventionally (using diverse fertilizers and products for pest and weed control) relative to organically-grown counterparts in a study in which the fruits were harvest at the early, mid and late season for three consecutive years [54]. There is also evidence that fertilization with nitrogen can affect positively the accumulation of carotenoids in red pepper [208] and carrot [209] and negatively in tomatoes [210], whereas phosphorous or potassium can have a positive effect in this crop [210].

### 3.3. Post-Harvest Treatments, Industrial Processing, Cooking and Storage Conditions

These topics are dealt with in detail elsewhere. In general, the losses of carotenoids increase with the intensity and the time of the treatments, with important differences among matrices and carotenoid species [118,211].

It is well-known that the synthesis of carotenoids can continue after harvest if the carotenogenic material remains intact, hence the carotenogenesis can be controlled to some extent by modulating parameters such as temperature, atmosphere or light, among others. Again, different behavior due factors including matrices, carotenoid species or conditions of the treatment have been reported. For instance, freezing storage for 6 months had no significant effect on β-carotene content of peas and carrots, although frozen storage for one year led to a decrease in α- and β-carotene in processed carrots [211].

It is very important to note that very frequently; published papers report that industrial processing or cooking lead to increases of the levels of carotenoids, these results being in most cases erroneous. Thus, in most cases the treatments inactivate proteins (including of course carotenogenic enzymes) and leads to carotenoid degradation. Claims about enhanced levels of carotenoids as a result of such industrial or culinary treatments should be accompanied by supporting evidence, for instance upregulation of carotenogenic genes or downregulation of genes encoding carotenoid oxygenases, which cleave carotenoids into retinoids, apocarotenoids or other products [13].

Another reason is that, very frequently, those treatments enhance the extractability of carotenoids from treated compared to raw matrices due to the changes such as the softening of membranes or walls [182,212]. In relation to this, it is important to note that, depending on the conditions; some treatments can decrease the content of carotenoids in the food but enhance their release from it during digestion, having therefore a potential positive impact on their bioavailability. Examples of this have been reported in carrot [166], tomato [138] and orange products [37], among others. On the other hand, sometimes, an important source of error is that the changes in the weight of the processed or cooked food are not taken into account; hence, the reported retentions of carotenoids are not realistic. Several formulae for their calculation are recommended in a reference text by Rodriguez-Amaya [182]:

% retention = (carotenoid content per g of cooked food × g of food after cooking/carotenoid content per g of raw food × g of food before cooking) × 100(1)

% retention = carotenoid content per g of cooked food (dry basis)/carotenoid content per g of raw food (dry basis) × 100(2)

% retention = carotenoid content (after cooking) per g of original raw food/carotenoid content per g of raw food × 100(3)

Apart from losses of carotenoids during processing, cooking and/or storage other phenomena including geometrical isomerization [213,214] or 5,6-epoxide to 5,8-furanoid rearrangements can take place [31,197]. Given that both kind of isomerizations are accompanied by changes in the light absorption spectra [186], noticeable food color changes as a result of them could be expected in some cases.

Taken together, it can be readily inferred that the levels of carotenoids do vary considerably in any food even in set of samples from the same geographical area. As some examples of this fact in samples analyzed in the same laboratory, 18–20-fold differences have been reported in marketed orange juices and tomato fruits [31,140,215] and 60-fold differences in olive oils [216].

## 4. Conclusions and Research Needs

The carotenoid analytical data collected for this work, showed a good average quality index, 24/35, according to the EuroFIR classification. It has been concluded that there is the need to improve the description and/or allocation of more resources to the definition of sampling plans and to the external control of the laboratory analytical methods to increase the number of traceable and comparable results. Only a complete analytical method validation, including accuracy evaluation, enables the estimation of the uncertainty of the measurement results (topic mentioned in only two of the publications analyzed) in order to assess its impact on studies based on dietary carotenoid content and in the assessment of actual differences in carotenoid levels. The uncertainty evaluation will also enable the definition of the number of significant figures of the results, avoiding the false impression of high accuracy of the results published with too many significant figures without support.

The analytical methods used to obtain the collected data generally included several mass transfer steps and in some cases a saponification reaction, which do lead to carotenoid losses, Hence it is important to mention clearly when the saponification step is included and measures taken to correct for losses (e.g., internal standards choice). On the other hand, the complete separation of the carotenoids in the chromatographic columns is in some cases difficult and requires specialized technicians.

The investment in faster analytical methods, less prone to error and using more environmentally friendly reagents and in less quantity will be an asset for the determination of carotenoids in foods. The analytical distinction between the *E* and *Z* isomers of the different carotenoids may be important to gain further insight into differences in bioaccessibility/bioavailability, vitamin A activity and other bioactivities.

Foods contain different carotenoids in different levels, so it is advisable to consume a diversified diet to obtain appropriate levels of the major health-promoting dietary carotenoids. This approach is also advised in the case of contaminants to minimize exposure to each contaminant.

Although food carotenoid databases are very useful tools to establish recommended intakes, bioaccessibility/bioavailability studies and more integrated studies considering all diet/components/individuals interactions are also needed as the carotenoid status depend on factors depending on the matrix, other dietary components and the individual. Although there is ample evidence that a diet rich in fruits and vegetables (e.g., Mediterranean Diet) has positive effects on human health and, establishing recommendations for dietary carotenoids intakes would contribute to reinforcing the education/informed choices by the population. In addition, it could contribute to decrease the consumption of heavily processed foods, which are often rich in saturated fat, sugar and salt. Provitamin A carotenoids are particularly very important for populations with limited availability of animal foods or for those that do not eat them by choice. In line with this fact, it is important to improve their conversion into vitamin A activity, overcoming limitations of RA and RAE formulas, and to appropriately consider them in the nutritional labeling of foods.

The wide variations in the carotenoid contents in different varieties/cultivars/landraces/accessions of a certain species illustrate the importance and the need for their documentation in food composition databases. This collection includes little consumed foods with very high levels of carotenoids (for instance rosehip or sarsaparrilla). This can contribute to take better advantage of biodiversity to enhance the carotenoid intake in particular. This database can be useful to include the carotenoid content/nutritional value criterion on the choice of varieties for agricultural cultivation, in addition to others such as high production or resistance to transport and pests. In environmental terms, studies on the carbon or water footprints of foods in general are also necessary as diet and environment are essential elements for human health.

## Supplementary Materials

The following are available online at https://www.mdpi.com/article/10.3390/foods10050912/s1, Table S1: Coffee, cocoa, tea and infusions (A03GG). Table S2: Seasoning, sauces and condiments (A042N). Table S3: Composite dishes (A03VA). Table S4: Fruit and vegetable juices and nectars (including concentrates) (A039K). Table S5: Animal and vegetable fats and oils and primary derivatives thereof (A036M). Table S6: Fruit and fruit products (A01BS). Table S7: Legumes, nuts, oilseeds and spices (A011X). Table S8: Starchy roots or tubers and products thereof, sugar plants (A00ZR). Table S9 Vegetables and vegetable products (A00FJ). Table S10: Grains and grain-based products (A000J).

## Appendix A

**Table A1:** Root and tuber vegetables (excluding starchy- and sugar-) (A00QF) (µg/100 g).

Food Name	Scientific Name	Origin (Country)	Purchase (Country)	Water (%)	Part Analysed	Colour	α-Carotene	β-Carotene	β-Cryptoxanthin	Ref.
Carrot	*Daucus carota* L.	United Kingdom	United Kingdom		peeled		4450 ± 940	32,000 ± 2880		[95]
Carrot	*Daucus carota* L.	Italy	Italy				2840–4960	4350–8840		[25]
Carrot	*Daucus carota* L.	Germany	Germany	91.3	edible part		4890	9020		[53]
Carrot	*Daucus carota* L.	Germany	Germany	84.3	edible part		3060	6500	12	[53]
Carrot	*Daucus carota* L.	Germany	Germany		edible part		4120	4650	28	[53]
Carrot	*Daucus carota* L.	Spain	Spain		edible part	orange	3245	8162		[24]
Carrot	*Daucus carota* L.	Spain	Spain		edible part	orange	2895	6628		[24]
Carrot	*Daucus carota* L.	Spain	Spain		edible part	orange	3700	9800		[24]
Carrot	*Daucus carota* L.	Turkey	Turkey		root	orange	1344–3011	4160–7162		[156]
Carrot	*Daucus carota* L.	Spain	Spain		edible part		3245	8162		[24]
Carrot	*Daucus carota* L.	Spain	Spain		edible part		2895	6628		[24]
Carrot	*Daucus carota* L.	Italy	Italy		all sample	L*52.2 ± 0.7a*24.1 ± 1.5b*36.1 ± 1	82,100 ± 1100	128,400 ± 800		[111]
Carrot	*Daucus carota* L.	Italy	Italy		all sample	L*52.1 ± 0.8a*22.6 ± 1.1b*35.7 ± 1.9	85,600 ± 2400	101,600 ± 700		[111]
Carrot	*Daucus carota* L.	Italy	Italy		all sample	L*50.2 ± 1.1a*21.4 ± 1.4b*35.3 ± 1.5	68,100 ± 9100	113,000 ± 16,700		[111]
Carrot	*Daucus carota* L.	Poland	Poland		roots			4820–9520		[157]
Carrot	*Daucus carota* L. cv Nerac	Ireland	Ireland		roots			188,000 ± 5000		[158]
Carrot	*Daucus carota* L. cv. Nantes	Brazil			tuber	orange	3500	6150	nd	[109]
Carrot	*Daucus carota* L. HCM	France	France		root	dark-orange	7583± 619	17,206 ± 643		[159]
Carrot	*Daucus carota* L. subsp. *sativus*	Finland	Finland				2200–4900	4600–10,300		[86]
Carrot	*Daucus carota* L. subsp. *sativus*	Spain	Spain				2900 ± 300	6600 ± 0		[86]
Carrot	*Daucus carota* L. subsp. *sativus*	United Kingdom	United Kingdom				2700–3600	8500–10,800		[86]
Carrot	*Daucus carota* L. subsp. *sativus*	USA					3900	5600		[86]
Carrot	*Daucus carota* L. subsp. *sativus*	Egypt					3400	6300		[86]
Carrot	*Daucus carota* L. subsp. *sativus*	Taiwan					2800 ± 300	5400 ± 600		[86]
Carrot	*Daucus carota* L. subsp. *sativus*	Malaysia					3400	6800		[86]
Carrot	*Daucus carota* L. var. Commercial French	France	France		root	orange	2322 ± 233	5404 ± 305		[159]
Carrot	*Daucus carota* L. var. Blanche à collet vert	France	France		root	white	nd	nd		[159]
Carrot	*Daucus carota* L. var. Blanche des vosges	France	France		root	white	nd	nd		[159]
Carrot	*Daucus carota* L. var. Carentan	France	France		root	orange	1644 ± 50	5932 ± 360		[159]
Carrot	*Daucus carota* L. var. Commercial French	France	France		root	orange	1972± 183	5433 ± 462		[159]
Carrot	*Daucus carota* L. var. Commercial French	France	France		root	orange	3131 ± 263	6633 ± 564		[159]
Carrot	*Daucus carota* L. var. Commercial French	France	France		root	orange	1419 ± 99	4149 ± 112		[159]
Carrot	*Daucus carota* L. var. Commercial French	France	France		root	orange	2291 ± 224	6190 ± 403		[159]
Carrot	*Daucus carota* L. var. Commercial French	France	France		root	orange	1916 ± 138	4730 ± 319		[159]
Carrot	*Daucus carota* L. var. Kokubu	France	France		root	orange	1748 ± 29	3740 ± 25		[159]
Carrot	*Daucus carota* L. var. La Merveille	France	France		root	orange	2092 ± 36	5869 ± 101		[159]
Carrot	*Daucus carota* L. var. Nantaise améliorée	France	France		root	orange	1369 ± 150	3625 ± 329		[159]
Carrot	*Daucus carota* L. var. San NaÏ	France	France		root	orange	1333 ± 85	3206 ± 182		[159]
Carrot	*Daucus carota* L. var. *sativa*, D.C.	Spain	Spain	88	root	orange	2895 ± 276	6628 ± 45		[24]
Carrot	*Daucus carota* L. var. *sativa*, D.C.	Spain	Spain	90	root	orange	3245 ± 128	8162 ± 364		[24]
Carrot	*Daucus carota* L. var. Violette jordanienne	France	France		root	purple	nd	381 ± 24		[159]
Carrot	*Daucus carota* L. var. Violette turque	France	France		root	purple	nd	318 ± 18		[159]
Carrot	*Daucus carota* L. var. yellowstone	France	France		root	yellow	nd	332 ± 15		[159]
Carrot	*Daucus carota* L., var. Bolero	Turkey	Turkey		root		1642 ± 101 3011 ± 217	4826 ± 465 7162 ± 503		[160]
Carrot	*Daucus carota* L., var. Fontana	Norway	Norway		root		5490–11,270	2210–5510		[161]
Carrot	*Daucus carota* L., var. Maestro-F1	Turkey	Turkey		root		1706 ± 60 2478 ± 179	4160 ± 148 6706 ± 42		[160]
Carrot	*Daucus carota* L., var. Merida	Norway	Norway		root		5550–10,200	1980–5220		[161]
Carrot	*Daucus carota* L., var. Nanco	Turkey	Turkey		root		1473 ± 34 2630 ± 33	4310 ± 118 6440 ± 146		[160]
Carrot	*Daucus carota* L., var. Nandrin	Norway	Norway		root		5630–12,080	1910–4530		[161]
Carrot	*Daucus carota* L., var. Nantindo	Turkey	Turkey		root		1665 ± 113–3006 ± 66	4767 ± 123 6637 ± 58		[160]
Carrot	*Daucus carota* L., var. Newburg	Norway	Norway		root		6100–10,800	2370–4090		[161]
Carrot	*Daucus carota* L., var. Presto-F1	Turkey	Turkey		root		2346 ± 45 2367 ± 217	5185 ± 468 6198 ± 138		[160]
Carrot	*Daucus carota* L., var. PS-F1	Turkey	Turkey		root		2688 ± 225 2967 ± 36	6168 ± 302 6373 ± 476		[160]
Carrot	*Daucus carota* L., var. Tito	Turkey	Turkey		root		1344 ± 17 1718 ± 153	4563 ± 311 4835 ± 473		[160]
Carrot	*Daucus carota* var. Jaune obtuse du Doubs	France	France		root	yellow	nd	332 ± 21		[159]
Carrot	*Daucus carota* var. New Kuroda	France	France		root	orange	1635 ± 17	3632 ± 64		[159]
Carrot	*Daucus carota* L. var. De Guérande	France	France		root	orange	1278 ± 234	3354 ± 457		[159]

**Table A2:** Spinaches and similar (A00MH) (µg/100 g).

Food Name	Scientific Name	FoodEx2_ TermCode	Origin (Country)	Purchase (Country)	Water (%)	Part Analysed	Colour	α-Carotene	β-Carotene	Lutein	Ref.
Spinach	*Spinacea oleracea* L.	A00MJ	Spain	Spain		edible part	green		4626	6422	[24]
Spinach	*Spinacea oleracea* L.	A00MJ	Spain	Spain	92	leaves + stalk	green		3254 ± 330	4229	[24]
Spinach	*Spinacea oleracea* L.	A00MJ	Spain	Spain	92	leaves + stalk	green		4626 ± 346	4229 ± 1310	[24]
Spinach	*Spinacea oleracea*, L.	A00MJ	Spain	Spain		edible part	green		3254	6422 ± 1190	[24]
Spinach	*Spinacia oleracea* L.	A00MJ	Brazil			leaves	green	nd	4423	5793	[109]
Spinach	*Spinacia oleracea* L.	A00MJ	Italy	Italy				nd	3100–4810	5930–7900	[25]
Spinach	*Spinacia oleracea* L.	A00MJ	Germany	Germany	90.8	edible part		90	3250	9540	[55]
Spinach	*Spinacia oleracea* L.	A00MJ	Spain	Spain		edible part			4626	8700 ± 100	[28]
Spinach	*Spinacia oleracea* L.	A00MJ	Spain	Spain		edible part			3254	218,700 ± 15,400	[28]
Spinach	*Spinacia oleracea* L.	A00MJ	Italy	Italy		all sample	L*30.5 ± 1.1a*8 ± 0.6b*10 ± 0.8		152,100 ± 5000	167,000 ± 10,400	[111]
Spinach	*Spinacia oleracea* L.	A00MJ	Italy	Italy		all sample	L*33.7 ± 1.1a*7.7 ± 1.1b*9.3 ± 1		148,100 ± 6500	194,200 ± 4200	[111]
Spinach	*Spinacia oleracea* L.	A00MJ	Italy	Italy		all sample	L*33.1 ± 0.8a*6.9 ± 0.9b*9.2 ± 0.8		18,4600 ± 9300	6422	[111]
Spinach	*Spinacia oleracea* L.	A00MJ	Slovenia	Slovenia	90.7	leaves	green			4840–13,900	[114]

**Table A3:** Mandarins and similar (A01CB) (µg/100 g).

Food Name	Scientific Name	FoodEx2_ TermCode	Origin (Country)	Purchase (Country)	Water (%)	Part Analysed	Colour	E(v. Trans)-α-Carotene	E(v. Trans)-β-Carotene	β-Cryptoxanthin	Lutein	Phytoene	Phytofluene	Violaxanthin	Zeaxanthin	Ref.
Mandarin	*Citrus* cv *Mediterranean*	A01CD	Italy	Italy		essential oil of peel fruit (free)				610 ± 62						[58]
Mandarin	*Citrus* cv *Mediterranean*	A01CD	Italy	Italy		essential oil of peel fruit (laurate)				1411 ± 140						[58]
Mandarin	*Citrus* cv *Mediterranean*	A01CD	Italy	Italy		essential oil of peel fruit (myristate)				1504 ± 132						[58]
Mandarin	*Citrus* cv *Mediterranean*	A01CD	Italy	Italy		essential oil of peel fruit (palmitate)				715 ± 74						[58]
Mandarin	*Citrus deliciosa*, Ten.	A01CJ	Spain	Spain	86	without skin	orange		213 ± 102	843 ± 216						[24]
Mandarin	*Citrus deliciosa*, Ten.	A01CJ	Spain	Spain	85	without skin	orange		130 ± 10	1106 ± 63						[24]
Mandarin	*Citrus reticulata*	A01CD	Spain	Spain			orange		213	843						[24]
Mandarin	*Citrus reticulata* Blanco cv. *Hansen*	A01CE	France	France		juice fruit			197	1500	163	446	500	467 (cis)	128	[43]
Mandarin	*Citrus reticulata* Blanco cv. *Hansen*	A01CE	France	France		juice fruit			70	162	230	100	110	445 (cis)	143	[43]
Mandarin	*Citrus reticulata* Blanco cv. *Hansen*	A01CE	French Polynesia			juice fruit			76	916	210	24	92	560 (cis)	131	[43]
Mandarin	*Citrus reticulate*, L. var. Tango	A01CD	Spain	Spain		fruit	orange	12.4	547	1331.6						[56]

**Table A4:** Cereal grains (and cereal-like grains) (A000L) (µg/100 g).

Food Name	Scientific Name	FoodEx2_ TermCode	Origin (Country)	Purchase (Country)	Saponification	Part Analysed	Colour	α-Carotene	(v. Trans)-α-Carotene	β-Carotene	E(v. Trans)-β-Carotene	β-Cryptoxanthin	E(V. Trans)-Lutein	Ref.
Maize	*Zea mays* L.	A000T	Spain	Spain		edible part	yellow	33		30				[24]
Maize	*Zea mays* L.	A000T	Croatia	Croatia		all sample				117.3± 8.17		158.5 ± 9.5		[174]
Maize	*Zea mays* L.	A000T#F28.A07KQ$F28.A0BA1	USA		no			15			14	0	202	[28]
Maize	*Zea mays* L.	A000T	Netherlands	Netherlands		kernels			20 ± 7		424 ± 6		157 ± 3	[175]
Maize	*Zea mays* L.	A000T	Netherlands	Netherlands		kernels			44 ± 9		447 ± 28		29 ± 2	[175]
Maize	*Zea mays* L.	A000T	Netherlands	Netherlands		kernels			58 ± 0		40 ± 2		93 ± 1	[175]
Maize	*Zea mays* L.	A000T	Netherlands	Netherlands		kernels			3 ± 1		246 ± 8		453 ± 8	[175]
Maize	*Zea mays* L.	A000T	Netherlands	Netherlands		kernels			44 ± 1		879 ± 28		37 ± 3	[175]
Maize	*Zea mays* L.	A000T	Netherlands	Netherlands		kernels			41 ± 7		448 ± 15		260 ± 10	[175]
Maize	*Zea mays* L.	A000T	Netherlands	Netherlands		kernels			17 ± 0		368 ± 2		988 ± 5	[175]
Maize	*Zea mays* L.	A000T	Netherlands	Netherlands		kernels			23 ± 2		56 ± 2		37 ± 2	[175]
Maize	*Zea mays* L.	A000T	Netherlands	Netherlands		kernels			11 ± 0		37 ± 0		41 ± 4	[175]
Maize	*Zea mays* L.	A000T	Netherlands	Netherlands		kernels			6 ± 4		253 ± 13		375 ± 15	[175]
Maize	*Zea mays* L.	A000T	Netherlands	Netherlands		kernels			16 ± 3		303 ± 16		251 ± 8	[175]
Maize	*Zea mays* L.	A000T	Netherlands	Netherlands		kernels			86 ± 6		277 ± 8		84 ± 2	[175]
Maize	*Zea mays* L.	A000T	Netherlands	Netherlands		kernels			23 ± 1		305 ± 20		371 ± 15	[175]

**Table A5:** Cucurbits with inedible peel (A00KD) (µg/100 g).

Food Name	Scientific Name	FoodEx2_ TermCode	Origin (Country)	Purchase (Country)	Water (%)	Part Analysed	Colour	α-Carotene	β-Carotene	β-Cryptoxanthin	Ref.
Pumpkin	*Cucurbita maxima*	A00KH	Spain	Spain		edible part			490	60	[24]
Pumpkin	*Cucurbita maxima*	A00KH	Spain	Spain		edible part		31	188		[24]
Pumpkin	*Cucurbita maxima*	A00KH	Spain	Spain		edible part		53	692		[24]
Pumpkin	*Cucurbita maxima* var. Autumn Cup	A00KH	Austria	Austria		flesh fruit		800	5200		[152]
Pumpkin	*Cucurbita maxima* var. Buen Gusto	A00KH	Austria	Austria		flesh fruit		1000	3300		[152]
Pumpkin	*Cucurbita maxima* var. Flat white Boer	A00KH	Austria	Austria		flesh fruit		7500	6200		[152]
Pumpkin	*Cucurbita maxima* var. Gelber Zentner	A00KH	Austria	Austria		flesh fruit		0	2200		[152]
Pumpkin	*Cucurbita maxima* var. Hyvita	A00KH	Austria	Austria		flesh fruit		990	2500		[152]
Pumpkin	*Cucurbita maxima* var. Imperial Elite	A00KH	Austria	Austria		flesh fruit		1100	7400		[152]
Pumpkin	*Cucurbita maxima* var. Japan 117	A00KH	Austria	Austria		flesh fruit		1000	7200		[152]
Pumpkin	*Cucurbita maxima* var. Mini green Hubbard	A00KH	Austria	Austria		flesh fruit		420	1400		[152]
Pumpkin	*Cucurbita maxima* var. Mishti kumra	A00KH	Bangladesh			fruit			362 ± 89.3		[157]
Pumpkin	*Cucurbita maxima* var. Snow Delite	A00KH	Austria	Austria		flesh fruit		1500	6400		[152]
Pumpkin	*Cucurbita maxima* var. Uchiki Kuri	A00KH	Austria	Austria		flesh fruit		1400	2500		[152]
Pumpkin	*Cucurbita maxima* var. Umber Cup	A00KH	Austria	Austria		flesh fruit		790	3700		[152]
Pumpkin	*Cucurbita maxima* var. Walfish	A00KH	Austria	Austria		flesh fruit		900	4300		[152]
Pumpkin	*Cucurbita maxima* x C. *moschata* var. Tetsuka Buto	A00KH	Austria	Austria		flesh fruit		2400	3500		[152]
Pumpkin	*Cucurbita moschata* Duchesne cv. Menina Brasileira and cv. Goianinha	A0DLT#F20.A07QF$F20.A07RD	Brazil			without skin and seed	orange	2530	6170	nd	[109]
Pumpkin	*Cucurbita moschata* var. Burpee Butterbush	A0DLT	Austria	Austria		flesh fruit		980	3100		[152]
Pumpkin	*Cucurbita moschata* var. Long Island Cheese	A0DLT	Austria	Austria		flesh fruit		5900	7000		[152]
Pumpkin	*Cucurbita moschata* var. Martinica	A0DLT	Austria	Austria		flesh fruit		1600	5400		[152]
Pumpkin	*Cucurbita moschata* var. Mousquée de Provence	A0DLT	Austria	Austria		flesh fruit		2800	4900		[152]
Pumpkin	*Cucurbita pepo*	A00KH	Italy	Italy					490	60	[25]
Pumpkin	*Cucurbita pepo* L.	A00KH	Poland	Poland	8.2	seed and oil	yellow-green	10–20	80–210	20–50	[153]
Pumpkin	*Cucurbita pepo* var. Acorn Table	A00KH	Austria	Austria		flesh fruit		150	2100		[152]
Pumpkin	*Cucurbita pepo* var. Acorn Tay Bell	A00KH	Austria	Austria		flesh fruit		150	2100		[152]
Pumpkin	*Cucurbita pepo* var. Carneval di Venezia	A00KH	Austria	Austria		flesh fruit		30	60		[152]
Pumpkin	*Cucurbita pepo* var. Melonette Jaspée Vende	A00KH	Austria	Austria		flesh fruit		50	1300		[152]
Pumpkin	*Cucurbita pepo* var. Tonda padana (Americano)	A00KH	Austria	Austria		flesh fruit		120	2300		[152]
Pumpkin	*Curcubita maxima*	A00KH	Spain	Spain		edible part	orange		490	60	[24]
Pumpkin	*Curcubita maxima*	A00KH	Spain	Spain		edible part	orange	31	188		[24]
Pumpkin	*Curcubita maxima*	A00KH	Spain	Spain		edible part	orange	53	692		[24]

**Table A6:** Rose hips and similar (A01FR) (µg/100 g).

Food Name	Scientific Name	FoodEx2_ TermCode	FoodEx2_ TermName	Origin (Country)	Purchase (Country)	Part Analysed	Colour	β-Carotene	E(v. Trans)-β-Carotene	Z(v. cis)-Β-carotene	E(v. Trans)-β-	Phytoene	Phytofluene	Ref.
Rosehip	*Rosa canina* L.	A0DSS	Dog rose	Turkey	Turkey	whole plants	red and pink	18–370						[75]
Rosehip	*Rosa canina* L.	A0DSS	Dog rose	Germany	Germany	all sample		2495 ± 207.5						[76]
Rosehip	*Rosa canina* L.	A0DSS	Dog rose	Germany	Germany	all sample		500 ± 35						[76]
Rosehip	*Rosa canina* L.	A0DSS	Dog rose	Germany	Germany	all sample			290 ± 32.5					[76]
Rosehip	*Rosa canina* L.	A0DSS	Dog rose	Germany	Germany	pulp			3200 ± 200	500 ± 320	1200 ± 100	400 ± 100	nd	[77]
Rosehip	*Rosa canina* L.	A0DSS	Dog rose	Germany	Germany	pulp			4200 ± 200	900 ± 240	100 ± 100	700 ± 100		[77]

**Table A7:** Rose hips and similar (A01FR) (µg/100 g) (continuation).

Food Name	Scientific Name	FoodEx2_ TermCode	FoodEx2_ TermName	Origin (Country)	Purchase (Country)	Part Analysed	Colour	Lycopene	E(v. Trans)-Lycopene	Z(v. Cis)-Lycopene	Violaxanthin	E(v. Trans)-Lutein	E(v. Trans)-Zeaxanthin	Ref.
Rosehip	*Rosa canina* L.	A0DSS	Dog rose	Germany	Germany	all sample		23,842.5 ± 777.5						[76]
Rosehip	*Rosa canina* L.	A0DSS	Dog rose	Germany	Germany	all sample		3615 ± 215						[76]
Rosehip	*Rosa canina* L.	A0DSS	Dog rose	Germany	Germany	pulp			7900 ± 1500	8400 ± 1600	300 ± 10	100 ± 10	2700 ± 30	[77]
Rosehip	*Rosa canina* L.	A0DSS	Dog rose	Germany	Germany	pulp			7400 ± 1200	6300 ± 600		700 ± 100	600 ± 100	[77]

**Table A8:** Cucurbits with inedible peel (A00KD) (µg/100 g).

Food Name	Scientific Name	FoodEx2_ TermCode	Origin (Country)	Purchase (Country)	Water (%)	Saponi-Fication	Part Analysed	Colour	α-Carotene	β-Carotene	E(v. Trans)-β-Carotene	β-Cryptoxanthin	Lycopene	E(v. Trans)-Lycopene	Ref.
Watermelon	*Citrullus lanatus (Thunb) Matsumura & Nakai*	A00KJ	Italy	Italy				red		46–546			959–1681		[154]
Watermelon	*Citrullus lanatus*	A00KJ	Spain	Spain			edible part	red		77		62	2454		[24]
Watermelon	*Citrullus lanatus*	A00KJ	USA			no			0		126	5			[28]
Watermelon	*Citrullus lanatus (Thunb.) Matsum & Nakai*	A00KJ	Brazil					red	nd	365		nd	3550		[109]
Watermelon	*Citrullus vulgaris*	A00KJ#F10.A0F2S	Indonesia				fruit	red		592 (314–777)		nd	11,389 (8731–13,523)		[59]
Watermelon	*Citrullus vulgaris*	A00KJ#F10.A0F5H	Indonesia				fruit	yellow		140 (56–287)		90 (59–110)	71 (nd -109)		[59]
Watermelon	*Citrullus vulgaris*	A00KJ#F10.A0F2S	Italy	Italy					nd	314–777		nd	4770–13,523		[25]
Watermelon	*Citrullus vulgaris*	A00KJ#F10.A0F5H	Italy	Italy					nd	56–287		59–110	nd–109		[25]
Watermelon	*Citrullus vulgaris, Schered.*	A00KJ#F20.A07QF$F20.A07RD	Spain	Spain	92		without rind or seeds	red		77 ± 29		62 ± 20	2454± 319		[24]
Watermelon	*Citrullus vulgaris, Schrad*	A00KJ	Spain	Spain			edible part	red			57.6 ± 4.8	1.2 ± 0.5			[56]
Watermelon	*Citrullus vulgaris*	A00KJ	Finland	Finland			pulp		-					3080	[26]

**Table A9:** Cucurbits with inedible peel (A00KD) (µg/100 g).

Food Name	Scientific Name	FoodEx2_ TermCode	Origin (Country)	Purchase (Country)	Water (%)	Saponi-Fication	Part Analysed	Colour	Lutein	E(v. Trans)-Lutein	E(v. Trans)-Zeaxanthin	Phytofluene	Phytoene	Ref.
Watermelon	*Citrullus lanatus*	A00KJ	Spain	Spain			edible part	red	40 ± 13				1122 ± 812	[24]
Watermelon	*Citrullus lanatus*	A00KJ	USA			no				4	0			[28]
Watermelon	*Citrullus lanatus*	A00KJ	Spain	Spain			Pulp	red				440	1170	[22]
