# Supplementary material for: European Database of Carotenoid Levels in Foods. Factors Affecting Carotenoid Content
Source: Foods. 2021 Apr 21;10(5):912. doi: 10.3390/foods10050912 (PMC8143354; doi:10.3390/foods10050912)
Supplement: Supplementary file 1 [file foods-10-00912-s001.zip › foods-1162108-supplementary.pdf]

Table S1. Coffee, cocoa, tea and infusions (A03GG)

Table 1.1 Tea leaves derivatives and tea ingredientes (A03HQ) (µg/100g)

Food name	Scientific name	FoodEx2_ TermCode	FoodEx2_ TermName	Origin (country)	Purchase (country)	Part analysed	Colour	E(v. trans)-β-carotene	Lutein	Ref.
Tea	*Camellia sinensis*	A04KK	Teas leaves, dry and/or fermented, and similar	Poland	Portugal	Herbal teas	Various colours	250-9220	770-29830	19

Table S2 Seasoning, sauces and condiments (A042N)

Table S2.1 Savoury sauces (A043V) (µg/100g)

Food name	Scientific name	FoodEx2_ TermCode	FoodEx2_ TermName	Origin (country)	Purchase (country)	Part analysed	Colour	β-carotene	E(v. trans)lycopene	Lutein	Lycopene	Phytoene	Phytofluene	Ref.
Harrissa sauce		A0ERG	Herbs/spices sauces	France	Belgium			4000						20
Pesto sauce		A044V	Pesto	Italy	Italy	Sauce	green	360-2780		420-2340				21
Spaguetti sauce		A043V	Savoury sauces	Spain	Spain	Sauce	red					2770	160	22
Spanish tomato frito		A044C	Tomato-containing cooked sauces	Spain	Spain		red	1812(all-E) ±13		296(all-E) ±2				23
Spanish tomato frito		A044C	Tomato-containing cooked sauces	Spain	Spain		red	2540(all-E) ±15		285(all-E) ±3				23
Spanish tomato frito		A044C	Tomato-containing cooked sauces	Spain	Spain		red	2120(all-E) ±11		301(all-E) ±2				23

Table S2.1 Savoury sauces (A043V) (µg/100g) (continuation)

Food name	Scientific name	FoodEx2_ TermCode	FoodEx2_ TermName	Origin (country)	Purchase (country)	Part analysed	Colour	β-carotene	E(v. trans)-lycopene	Lutein	Lycopene	Phytoene	Phytofluene	Ref.
Spanish tomato frito		A044C	Tomato-containing cooked sauces	Spain	Spain		red	1940(all-E) ±12		310(all-E) ±5				23
Spanish tomato frito		A044C	Tomato-containing cooked sauces	Spain	Spain		red	2969(all-E) ±11		342(all-E) ±4				23
Spanish tomato frito		A044C	Tomato-containing cooked sauces	Spain	Spain		red	2120(all-E) ±13		291(all-E) ±4				23
Spanish tomato frito		A044C	Tomato-containing cooked sauces	Spain	Spain		red	1560(all-E) ±11		255(all-E) ±3				23
Spanish tomato frito		A044C	Tomato-containing cooked sauces	Spain	Spain		red	1330(all-E) ±12		241(all-E) ±5				23
Spanish tomato frito		A044C	Tomato-containing cooked sauces	Spain	Spain		red	1780(all-E) ±19		258(all-E) ±3				23
Spanish tomato frito		A044C	Tomato-containing cooked sauces	Spain	Spain		red	1650(all-E) ±21		251(all-E) ±2				23
Spanish tomato frito		A044C	Tomato-containing cooked sauces	Spain	Spain		red	315		114	14571			24
Tomato ketchup		A044P	Tomato ketchup and related sauces	Italy	Italy			135–500		nd	4710–23 400			25
Tomato Ketchup		A044P	Tomato ketchup and related sauces	Spain	Spain	Sauce	red	5000			9900			24
Tomato Ketchup		A044P	Tomato ketchup and related sauces	Spain	Spain		red	5000			9900			24
Tomato ketchup		A044P	Tomato ketchup and related sauces	Spain	Spain	Sauce	red					3440 (3390-3490)	1290 (1030-1540)	22
Tomato ketchup		A044P	Tomato ketchup and related sauces	Finland	Finland		-		2540					26
Tomato sauce		A044C	Tomato-containing cooked sauces	Spain	Germany				99700					27

Table S2.1 Savoury sauces (A043V) (µg/100g) (continuation)

Food name	Scientific name	FoodEx2_ TermCode	FoodEx2_ TermName	Origin (country)	Purchase (country)	Part analysed	Colour	β-carotene	E(v. trans)- lycopene	Lutein	Lycopene	Phytoene	Phytofluene	Ref.
Tomato sauce		A044C	Tomato-containing cooked sauces	Spain	Germany				98500					27
Tomato sauce		A044C	Tomato-containing cooked sauces	Spain	Germany				120500					27
Tomato sauce		A044C	Tomato-containing cooked sauces	Spain	Germany				79500					27
Tomato sauce		A044C	Tomato-containing cooked sauces	Spain	Germany				101600					27
Tomato sauce		A044C	Tomato-containing cooked sauces	Spain	Germany				93900					27
Tomato sauce		A044C	Tomato-containing cooked sauces	Spain	Germany				99700					27
Tomato sauce		A044C	Tomato-containing cooked sauces	Spain	Germany				87400					27
Tomato sauce		A044C	Tomato-containing cooked sauces	Spain	Germany				91700					27
Tomato sauce		A044C	Tomato-containing cooked sauces	Spain	Spain	Sauce	red					2950	1270	22
Tomato sauce, instant		A044C	Tomato-containing cooked sauces	Italy	Italy						5600–39 400			25

Table S3. Composite dishes (A03VA)

Table S3.1 Potato based dishes (A03VD) (µg/100g)

Food name	Scientific name	FoodEx2_ TermCode	FoodEx2_ TermName	Origin (country)	Purchase (country)	Saponification	Colour	α-carotene	β-carotene	β-cryptoxanthin	E(v. trans)-β-carotene	E(v. trans)-lutein	Ref.
Aloo Bombay		A03VD	Potato based dishes	United Kingdom	Belgium				646				20

Table S3.2 Legumes based dishes (A03VM) (µg/100g)

Food name	Scientific name	FoodEx2_ TermCode	FoodEx2_ TermName	Origin (country)	Purchase (country)	Saponification	Colour	α-carotene	β-carotene	β-cryptoxanthin	E(v. trans)-β-carotene	E(v. trans)-lutein	Ref.
Comercial hummus		A03VN#F22.A07SH	Hummus, PREPARATION-PRODUCTION-PLACE = Food industry prepared	Israel	Belgium				17				20
Falafel		A03VM	Legumes based dishes	Israel	Belgium				82				20
Soy patty		A03VM	Legumes based dishes	Israel	Belgium				168				20

Table S3.3 Vegetable based dishes (A03XX) (µg/100g)

Food name	Scientific name	FoodEx2_ TermCode	FoodEx2_ TermName	Origin (country)	Purchase (country)	Saponification	Colour	α-carotene	β-carotene	β-cryptoxanthin	E(v. trans)-β-carotene	E(v. trans)-lutein	Ref.
Meloukhia sauce		A03XX	Vegetable based dishes	France	Belgium				83				20

Table S3.4 Rice based dishes, cooked (A040Z) (µg/100g)

Food name	Scientific name	FoodEx2_ TermCode	FoodEx2_ TermName	Origin (country)	Purchase (country)	Saponification	Colour	α-carotene	β-carotene	β-cryptoxanthin	E(v. trans)-β-carotene	E(v. trans)-lutein	Ref.
Cantonese rice		A040Z	Rice based dishes, cooked	Italy	Belgium				12				20

Table S3.5.1 Soups (ready-to-eat) (A041L) (µg/100g)

Food name	Scientific name	FoodEx2_ TermCode	FoodEx2_ TermName	Origin (country)	Purchase (country)	Saponification	Colour	α-carotene	β-carotene	β-cryptoxanthin	E(v. trans)-β-carotene	E(v. trans)-lutein	Ref.
Tomato soup		A041N	Tomato soup	Netherlands	Germany						7450±1010		27
Tomato soup		A041N	Tomato soup	Netherlands	Germany						6210±250		27
Tomato soup		A041N	Tomato soup	Netherlands	Germany						4090±200		27
Tomato soup		A041N	Tomato soup	Netherlands	Germany						3260±80		27

Table S3.5.2 Soups (ready-to-eat) (A041L) (µg/100g) (continuation)

Food name	Scientific name	FoodEx2_TermCode	FoodEx2_TermName	Origin (country)	Purchase (country)	Saponification	Colour	E(v. trans)-zeaxanthin	Phytoene	Phytofluene	Z(v. cis)-β-carotene	Ref.
Minestrone soup		A0BZ9	Mixed vegetables soup, with puree or pieces	Spain	Spain		yellow, red, green		280	170		22
Tomato soup		A041N	Tomato soup	Spain	Spain		red		1720	720		22

Table S3.6 Salads (A042B) (µg/100g)

Food name	Scientific name	FoodEx2_TermCode	FoodEx2_TermName	Origin (country)	Purchase (country)	Saponification	Colour	α-carotene	β-carotene	β-cryptoxanthin	E(v. trans)-β-carotene	E(v. trans)-lutein	Ref.
Fruit salad		A01QG	Fruit salad	Spain	Spain		multicolor		138	52			24
Fruit salad		A01QG	Fruit salad	Spain	Spain		multicolor		20	50			24

Table S3.7.1 Snacks other than chips and similar (A06HL) (µg/100g)

Food name	Scientific name	FoodEx2_TermCode	FoodEx2_TermName	Origin (country)	Purchase (country)	Saponification	Colour	α-carotene	β-carotene	β-cryptoxanthin	E(v. trans)-β-carotene	E(v. trans)-lutein	Ref.
Corn Cereals - Cheetos	*Zea mays*	A00FH#F04.A000T	Mixed cereal-based snacks, INGREDIENT = Maize grain	USA		no		0		8	0	66	28
Corn Cereals - Chex Mix	*Zea mays*	A00FH#F04.A000T	Mixed cereal-based snacks, INGREDIENT = Maize grain	USA		no		6		4	12	48	28

Table S3.7.1 Snacks other than chips and similar (A06HL) (µg/100g) (continuation)

Food name	Scientific name	FoodEx2_ TermCode	FoodEx2_ TermName	Origin (country)	Purchase (country)	Saponification	Colour	α-carotene	β-carotene	β-cryptoxanthin	E(v. trans)-β-carotene	E(v. trans)-lutein	Ref.
Corn Cereals - Fritos	*Zea mays*	A00FH#F04.A000T	Mixed cereal-based snacks, INGREDIENT = Maize grain	USA		no		13		3	8	17	28
Corn Cereals - Tortilla	*Zea mays*	A00FH#F04.A000T	Mixed cereal-based snacks, INGREDIENT = Maize grain	USA		no		0		30	0	276	28
Corn Cereals - Tortilla chip, Tostidos	*Zea mays*	A00FH#F04.A000T	Mixed cereal-based snacks, INGREDIENT = Maize grain	USA		no		0		0	0	0	28

Table S3.7.2 Snacks other than chips and similar (A06HL) (µg/100g) (continuation)

Food name	Scientific name	FoodEx2_ TermCode	FoodEx2_ TermName	Origin (country)	Purchase (country)	Saponification	Colour	E(v. trans)-zeaxanthin	Phytoene	Phytofluene	Z(v. cis)-β-carotene	Ref.
Corn Cereals - Cheetos	*Zea mays*	A00FH#F04.A000T	Mixed cereal-based snacks, INGREDIENT = Maize grain	USA		no		73			0	28
Corn Cereals - Chex Mix	*Zea mays*	A00FH#F04.A000T	Mixed cereal-based snacks, INGREDIENT = Maize grain	USA		no		25			3	28
Corn Cereals - Fritos	*Zea mays*	A00FH#F04.A000T	Mixed cereal-based snacks, INGREDIENT = Maize grain	USA		no		33			1	28
Corn Cereals - Tortilla	*Zea mays*	A00FH#F04.A000T	Mixed cereal-based snacks, INGREDIENT = Maize grain	USA		no		255			0	28
Corn Cereals - Tortilla chip, Tostidos	*Zea mays*	A00FH#F04.A000T	Mixed cereal-based snacks, INGREDIENT = Maize grain	USA		no		0			0	28

Table S4. Fruit and vegetable juices and nectars (including concentrates) (A039K)

Table S4.1.1 Fruit juices (100% from named source) (A0BY4) (µg/100g)

Food name	Scientific name	FoodEx2_ TermCode	FoodEx2_ TermName	Origin (country)	Purchase (country)	Water (%)	Process	Colour	α-carotene	β-carotene	β-cryptoxanthin	ζ-carotene	Antheraxanthin	Ref.
Mandarin	*Citrus reticulata Blanco or Citrus deliciosa Ten.*	A0BY4#F27.A 01CD	Fruit juices (100% from named source), SOURCE-COMMODITIES = Mandarins	Italy	Italy				nd±	55	752			25
Mandarin Willow leaf	*Citrus deliciosa Ten.*	A0BY4#F27.A 01CD	Fruit juices (100% from named source), SOURCE-COMMODITIES = Mandarins	France	France					198	1400			29
Mandarin Willow leaf	*Citrus deliciosa Ten.*	A0BY4#F27.A 01CD	Fruit juices (100% from named source), SOURCE-COMMODITIES = Mandarins	France	France					40	1051			29
Orange	*Citrus sinensis L.*	A03AM	Juice, orange	Brazil				orange	54-130	53-192	104-762	39-383		30
Orange	*Citrus sinensis L.*	A03AM	Juice, orange	Italy	Italy				nd – 31	nd – 98	16–151			25
Orange	*Citrus sinensis L.*	A03AM#F28. A07MR	Juice, orange, PROCESS = Reconstitution from concentrate, powder or other dehydrated form	Spain	Spain		reconstitution from concentrate	orange	3 (n.d.-25)±4	(n.d.- 96)	41 (14-87)±22	(nd - 17)	(nd - 29)	31
Orange	*Citrus sinensis L. var. navel lane late*	A03AM	Juice, orange	Portugal	Portugal			orange	440	300	130			32
Orange	*Citrus sinensis var. Valencia*	A03AM	Juice, orange	Spain	Spain		raw		52.40 ±17.04	53.74 ±21.95	11.82±1.41			33
Orange	*Citrus sinensis var. Valencia*	A03AM#F28. A07HV	Juice, orange, PROCESS = Pasteurisation	Spain	Spain		pasteurisation		30.14 ±11.68	32.79 ±13.01	8.00±1.59			33
Orange	*Citrus sinensis var. Valencia*	A03AM#F28. A07LJ	Juice, orange, PROCESS = Homogenizing or emulsifying	Spain	Spain		raw ultra high pressure homogenization		35.76 ±17.60	44.90 ±23.98	10.29±2.57			33

Table S4.1.1 Fruit juices (100% from named source) (A0BY4) (µg/100g) (Continuation)

Food name	Scientific name	FoodEx2_ TermCode	FoodEx2_ TermName	Origin (country)	Purchase (country)	Water (%)	Process	Colour	α-carotene	β-carotene	β-cryptoxanthin	ζ-carotene	Antheraxanthin	Ref.
Orange	*Citrus sinensis var. Valencia*	A03AM#F28. A07LJ	Juice, orange, PROCESS = Homogenizing or emulsifying	Spain	Spain		raw ultra high pressure homogenization		42.32 ±24.24	51.77 ±26.00	10.96±3.24			33
Orange	*Citrus sinensis var. Valencia*	A03AM#F28. A07LJ	Juice, orange, PROCESS = Homogenizing or emulsifying	Spain	Spain		raw ultra high pressure homogenization		35.58 ±23.25	37.01 ±26.35	10.29±2.12			33
Orange	*Citrus sinensis Osbeck*	A03AM	Juice, orange	Italy	Italy				0.09±0.01	0.44±0.08	2.48±0.27		11.7±0.88	34
Orange	*Citrus sinensis Osbeck*	A03AM	Juice, orange	Italy	Italy				0.07±0.01	0.40±0.02	2.14±0.17		8.13±0.4	34
Orange	*Citrus sinensis Osbeck*	A03AM	Juice, orange	Italy	Italy				0.06	0.24±0.01	1.41±0.06		6.21±0.92	34
Orange	*Citrus sinensis Osbeck*	A03AM	Juice, orange	Italy	Italy				0.03	0.15	1.26±0.05		5.36±0.9	34
Orange	*Citrus sinensis L. var. valencia late*	A03AM	Juice, orange	Spain	Spain			Orange	11±5	21±7	69±27			35
Orange	*Citrus sinensis L.*	A03AM	Juice, orange	Spain	Spain			Orange	2	24	99			24
Orange	*Citrus sinensis L.*	A03AM	Juice, orange	Spain	Spain			Orange	6	82	701			24
Orange	*Citrus sinensis L.*	A03AM#F22. A07SH	Juice, orange, PREPARATION-PRODUCTION-PLACE = Food industry prepared	Spain	Spain				2	24	99			24
Orange	*Citrus sinensis L.*	A03AM	Juice, orange	Spain	Spain		Natuiral juice		6	82	701			24
Orange	*Citrus sinensis L.*	A03AM	Juice, orange	Spain	Spain				nd-23	13455	33-121			35
Orange	*Citrus sinensis L. var. Bionda*	A03AM	Juice, orange	Italy	Italy						72 (16:0)			36
Orange	*Citrus sinensis L. var. Brasiliana*	A03AM	Juice, orange	Italy	Italy						159 (12:0) + 171 (14:0) + 55 (16:0)			36

Table S4.1.1 Fruit juices (100% from named source) (A0BY4) (µg/100g) (Continuation)

Food name	Scientific name	FoodEx2_ TermCode	FoodEx2_ TermName	Origin (country)	Purchase (country)	Water (%)	Process	Colour	α-carotene	β-carotene	β-cryptoxanthin	ζ-carotene	Antheraxanthin	Ref.
Orange	*Citrus sinensis L. var. Moro*	A03AM	Juice, orange	Italy	Italy						25 + 56 (16:0)			36
Orange	*Citrus sinensis L. var. Ovale*	A03AM	Juice, orange	Italy	Italy						141 (14:0) + 32 (16:0)		11	36
Orange	*Citrus sinensis L. var. Sanguinello*	A03AM	Juice, orange	Italy	Italy						59 + 185 (12:0) + 114 (14:0) + 111 (16:0)			36
Orange	*Citrus sinensis L. var. Tarocco*	A03AM	Juice, orange	Italy	Italy						46 (16:0)		11	36
Orange	*Citrus sinensis L. var. Valence*	A03AM	Juice, orange	Italy	Italy						15 + 82 (12:0) + 73 (14:0) + 84 (16:0)		38	36
Orange	*Citrus sinensis L. var. Washinghton*	A03AM	Juice, orange	Italy	Italy						14 + 46 (12:0) + 31 (14:0) + 33 (16:0)			36
Orange	*Citrus sinensis L. var. valencia late*	A03AM	Juice, orange	Spain	Spain		hand squeezed	orange	32±4	88±17	209±42			37
Orange	*Citrus sinensis L. var. valencia late*	A03AM	Juice, orange	Spain	Spain			orange	29±2	78±13	166±39			37
Orange	*Citrus sinensis L. var. valencia late*	A03AM#F28. A07HV	Juice, orange, PROCESS = Pasteurisation	Spain	Spain		pasteurization	orange	27±2	72±9	168±24			37
Orange	*Citrus sinensis L. var. navel late*	A03AM	Juice, orange	Spain	Spain			orange			71±3	37±1		38
Orange	*Citrus sinensis L. var. navel late*	A03AM	Juice, orange	Spain	Spain			orange			85±4	46±2		38
Orange	*Citrus sinensis L. var. navel late*	A03AM	Juice, orange	Spain	Spain			orange			85±1	46±1		38

Table S4.1.1 Fruit juices (100% from named source) (A0BY4) (µg/100g) (Continuation)

Food name	Scientific name	FoodEx2_ TermCode	FoodEx2_ TermName	Origin (country)	Purchase (country)	Water (%)	Process	Colour	α-carotene	β-carotene	β-cryptoxanthin	ζ-carotene	Antheraxanthin	Ref.
Orange	*Citrus sinensis L.*	A03AM	Juice, orange	Spain	Spain			orange						22
Orange	*Citrus sinensis L.*	A03AM	Juice, orange	USA					0		23			28
Pear	*Pyrus cv Conference*	A0BY4#F27.A01DP$F22.A07SH	Fruit juices (100% from named source), SOURCE-COMMODITIES = Pears, PREPARATION-PRODUCTION-PLACE = Food industry prepared	Spain	Spain	80 - 90				5.7±0.8	2.8 (all-E)±0.3	1.2±0.1		39
Pear	*Pyrus cv Blanquilla*	A0BY4#F27.A01DP$F22.A07SH	Fruit juices (100% from named source), SOURCE-COMMODITIES = Pears, PREPARATION-PRODUCTION-PLACE = Food industry prepared	Spain	Spain	80 - 90				2.9±0.5	2.0 (all-E)±0.2			39
Pear	*Pyrus cv Conference*	A0BY4#F27.A01DP	Fruit juices (100% from named source), SOURCE-COMMODITIES = Pears	Spain	Spain	80 - 90				13.1±2.8	3.8 (all-E)±0.4		2.7 (all-E)±0.1	39
Pear	*Pyrus cv Blanquilla*	A0BY4#F27.A01DP	Fruit juices (100% from named source), SOURCE-COMMODITIES = Pears	Spain	Spain	80 - 90				16.8±3.7	3.6 (all-E)±0.4		3.2 (all-E)±0.2	39

Table S4.1.2 Fruit juices (100% from named source) (A0BY4) (µg/100g) (continuation)

Food name	Scientific name	FoodEx2_ TermCode	FoodEx2_ TermName	Origin (country)	Purchase (country)	Water (%)	Process	Colour	Auroxanthin	E(v. trans)-α-carotene	E(v. trans)-β-carotene	E(v. trans)-lutein	E(v. trans)-lycopene	Ref.
Orange	-	A03AM#F28. A07MR	Juice, orange, PROCESS = Reconstitution from concentrate, powder or other dehydrated form	Spain	Spain		from concentrated	orange	A: 29±11 (9-54) B: 27±9 (5-44) C: (nd -29)					31
Orange	*Citrus sinensis L. var. Bionda*	A03AM	Juice, orange	Italy	Italy				19					36
Orange	*Citrus sinensis L. var. Brasiliana*	A03AM	Juice, orange	Italy	Italy				nd					36
Orange	*Citrus sinensis L. var. Moro*	A03AM	Juice, orange	Italy	Italy				nd					36
Orange	*Citrus sinensis L. var. Ovale*	A03AM	Juice, orange	Italy	Italy				19					36
Orange	*Citrus sinensis L. var. Sanguinello*	A03AM	Juice, orange	Italy	Italy				nd					36
Orange	*Citrus sinensis L. var. Tarocco*	A03AM	Juice, orange	Italy	Italy				nd					36
Orange	*Citrus sinensis L. var. Valence*	A03AM	Juice, orange	Italy	Italy				50					36
Orange	*Citrus sinensis L. var. Washinghton*	A03AM	Juice, orange	Italy	Italy				nd					36
Orange	*Citrus sinensis L. var. valencia late*	A03AM	Juice, orange	Spain	Spain		hand squeezed	orange	nd					37
Orange	*Citrus sinensis L. var. navel late*	A03AM	Juice, orange	Spain	Spain			orange		4±0	8±0	30±1		38
Orange	*Citrus sinensis L. var. navel late*	A03AM	Juice, orange	Spain	Spain			orange		5±0	9±0	35±2		38
Orange	*Citrus sinensis L. var. navel late*	A03AM	Juice, orange	Spain	Spain			orange		5±0	9±0	36±0		38
Orange	*Citrus sinensis*	A03AM	Juice, orange	USA							0	33		28

Table S4.1.3 Fruit juices (100% from named source) (A0BY4) (µg/100g) (continuation)

Food name	Scientific name	FoodEx2_ TermCode	FoodEx2_ TermName	Origin (country)	Purchase (country)	Water (%)	Process	Colour	E(v. trans)-zeaxanthin	Lutein	Luteoxanthin	Lycopene	Mutatoxanthin	Ref.
Mandarin Willow leaf	*Citrus deliciosa Ten*	A0BY4#F27.A01CD	Fruit juices (100% from named source), SOURCE-COMMODITIES = Mandarins	France	France					91				29
Mandarin Willow leaf	*Citrus deliciosa Ten*	A0BY4#F27.A01CD	Fruit juices (100% from named source), SOURCE-COMMODITIES = Mandarins	France	France					115				29
Orange	*Citrus sinensis*	A03AM	Juice, orange	Brazil				orange		227-390				30
Orange	-	A03AM#F28.A07MR	Juice, orange, PROCESS = Reconstitution from concentrate, powder or other dehydrated form	Spain	Spain		from concentrated	orange		23 (6-42)±9	(nd - 20)			31
Orange	*Citrus sinensis L. var. navel lane late*	A03AM	Juice, orange	Portugal	Portugal			orange		180				32
Orange	*Citrus sinensis var. Valencia*	A03AM	Juice, orange	Spain	Spain		raw			2.35±0.63				33
Orange	*Citrus sinensis var. Valencia*	A03AM#F28.A07HV	Juice, orange, PROCESS = Pasteurisation	Spain	Spain		pasteurized			1.84±0.85				33
Orange	*Citrus sinensis var. Valencia*	A03AM#F28.A07LJ	Juice, orange, PROCESS = Homogenizing or emulsifying	Spain	Spain		ultra high pressure homogenization			2.28±0.57				33
Orange	*Citrus sinensis var. Valencia*	A03AM#F28.A07LJ	Juice, orange, PROCESS = Homogenizing or emulsifying	Spain	Spain		ultra high pressure homogenization			2.10±0.81				33
Orange	*Citrus sinensis var. Valencia*	A03AM#F28.A07LJ	Juice, orange, PROCESS = Homogenizing or emulsifying	Spain	Spain		ultra high pressure homogenization			2.31±0.82				33
Orange	*Citrus sinensis Osbeck*	A03AM	Juice, orange	Italy	Italy					1.94±0.14	2.22±0.13			34
Orange	*Citrus sinensis Osbeck*	A03AM	Juice, orange	Italy	Italy					2.10±0.32	1.97±0.16			34

Table S4.1.3 Fruit juices (100% from named source) (A0BY4) (µg/100g) (continuation)

Food name	Scientific name	FoodEx2_ TermCode	FoodEx2_ TermName	Origin (country)	Purchase (country)	Water (%)	Process	Colour	E(v. trans)- zeaxanthin	Lutein	Luteoxanthin	Lycopene	Mutatoxanthin	Ref.
Orange	*Citrus sinensis Osbeck*	A03AM	Juice, orange	Italy	Italy					1.17±0.11	2.20±0.12			34
Orange	*Citrus sinensis Osbeck*	A03AM	Juice, orange	Italy	Italy					0.89±0.2	1.86±0.1			34
Orange	*Citrus sinensis L. var. Bionda*	A03AM	Juice, orange	Italy	Italy					21	23 (12:0) + 110 (14:0) + 85 (16:0)		12 + 21 (16:0)	34
Orange	*Citrus sinensis L. var. Brasiliana*	A03AM	Juice, orange	Italy	Italy						26 (14:0)		32 (16:0)	36
Orange	*Citrus sinensis L. var. Moro*	A03AM	Juice, orange	Italy	Italy						28 (12:0) + 55 (14:0) + 69 (16:0			36
Orange	*Citrus sinensis L. var. Ovale*	A03AM	Juice, orange	Italy	Italy					16	30 (12:0) + 107 (14:0) + 65 (16:0)		15	36
Orange	*Citrus sinensis L. var. Sanguinello*	A03AM	Juice, orange	Italy	Italy					15	47 (12:0) + 110 (14:0) + 95 (16:0)		13	36
Orange	*Citrus sinensis L. var. Tarocco*	A03AM	Juice, orange	Italy	Italy					19	12 + 28 (12:0) + 61 (14:0) + 55 (16:0)		12	36
Orange	*Citrus sinensis L. var. Valence*	A03AM	Juice, orange	Italy	Italy					18	14 + 59 (14:0)		12	36
Orange	*Citrus sinensis L. var. Washinghton*	A03AM	Juice, orange	Italy	Italy					12	10 (14:00)			36
Orange	*Citrus sinensis L. var. valencia late*	A03AM	Juice, orange	Spain	Spain		hand squeezed	orange		139±21				37
Orange	*Citrus sinensis L. var. valencia late*	A03AM	Juice, orange	Spain	Spain			orange		126±12				37

Table S4.1.3 Fruit juices (100% from named source) (A0BY4) (µg/100g) (continuation)

Food name	Scientific name	FoodEx2_ TermCode	FoodEx2_ TermName	Origin (country)	Purchase (country)	Water (%)	Process	Colour	E(v. trans)-zeaxanthin	Lutein	Luteoxanthin	Lycopene	Mutatoxanthin	Ref.
Orange	*Citrus sinensis L. var. valencia late*	A03AM#F28. A07HV	Juice, orange, PROCESS = Pasteurisation	Spain	Spain		pasteurization	orange		117±8				37
Orange	*Citrus sinensis L. var. navel late*	A03AM	Juice, orange	Spain	Spain			orange	40±2					38
Orange	*Citrus sinensis L. var. navel late*	A03AM	Juice, orange	Spain	Spain			orange	47±2					38
Orange	*Citrus sinensis L. var. navel late*	A03AM	Juice, orange	Spain	Spain			orange	47±0					38
Orange	*Citrus sinensis L.*	A03AM	Juice, orange	Spain	Spain			orange						22
Orange	*Citrus sinensis*	A03AM	Juice, orange	USA					26					28
Pear	*Pyrus cv Conference*	A0BY4#F27.A 01DP$F22.A0 7SH	Fruit juices (100% from named source), SOURCE-COMMODITIES = Pears, PREPARATION-PRODUCTION-PLACE = Food industry prepared	Spain	Spain	80 - 90				16.8 ± 2.7 (all-E) 2.6 ± 0.2 (isomers)				39
Pear	*Pyrus cv Blanquilla*	A0BY4#F27.A 01DP$F22.A0 7SH	Fruit juices (100% from named source), SOURCE-COMMODITIES = Pears, PREPARATION-PRODUCTION-PLACE = Food industry prepared	Spain	Spain	80 - 90				8.7 ± 1.5 (all-E) 2.3 ± 0.1 (isomers)				39
Pear	*Pyrus cv Conference*	A0BY4#F27.A 01DP	Fruit juices (100% from named source), SOURCE-COMMODITIES = Pears	Spain	Spain	80 - 90	freshly squeezed juice			36.6 ± 10.4 (all-E) 2.1 ± 0.4 (isomers)				39
Pear	*Pyrus cv Blanquilla*	A0BY4#F27.A 01DP	Fruit juices (100% from named source), SOURCE-COMMODITIES = Pears	Spain	Spain	80 - 90	freshly squeezed juice			35.9 ± 6.8 (all-E) 1.9 ± 0.4 (isomers)				39

Table S4.1.4 Fruit juices (100% from named source) (A0BY4) (µg/100g) (continuation)

Food name	Scientific name	FoodEx2_ TermCode	FoodEx2_ TermName	Origin (country)	Purchase (country)	Water (%)	Process	Colour	Neochrome	Neoxanthin	Phytoene	Phytofluene	Violaxanthin	Ref.
Caja	*Spondias lutea*	A0BY4#F27.A01HE	Fruit juices (100% from named source), SOURCE-COMMODITIES = Miscellaneous fruits with edible peel	Spain	Spain			orange			40			22
Clementine	*Citrus clementina*	A0BY4#F27.A01CE	Fruit juices (100% from named source), SOURCE-COMMODITIES = Clementines	Spain	Spain			orange			40	90		22
Grapefruit	*Citrus paradisi*	A03AL	Juice, grapefruit	Spain	Spain			red			210	70		22
Lemon	*Citrus reticulata*	A0BY4#F27.A01CH	Fruit juices (100% from named source), SOURCE-COMMODITIES = Tangerines	Spain	Spain			orange			150 (120-190)	280 (230-340)		22
Mandarin Willow leaf	*Citrus deliciosa Ten*	A0BY4#F27.A01CD	Fruit juices (100% from named source), SOURCE-COMMODITIES = Mandarins	France	France						230	301	267 (cis)	29
Mandarin Willow leaf	*Citrus deliciosa Ten*	A0BY4#F27.A01CD	Fruit juices (100% from named source), SOURCE-COMMODITIES = Mandarins	France	France						1±	74	179 (cis)	29
Orange	*Citrus sinensis*	A03AM	Juice, orange	Brazil				orange					114-169	30
Orange	*Citrus sinensis Osbeck*	A03AM	Juice, orange	Italy	Italy								1.69±0.23	34
Orange	*Citrus sinensis Osbeck*	A03AM	Juice, orange	Italy	Italy								1.35±0.06	34

Table S4.1.4 Fruit juices (100% from named source) (A0BY4) (µg/100g) (continuation)

Food name	Scientific name	FoodEx2_ TermCode	FoodEx2_ TermName	Origin (country)	Purchase (country)	Water (%)	Process	Colour	Neochrome	Neoxanthin	Phytoene	Phytofluene	Violaxanthin	Ref.
Orange	*Citrus sinensis Osbeck*	A03AM	Juice, orange	Italy	Italy								1.30±0.15	34
Orange	*Citrus sinensis Osbeck*	A03AM	Juice, orange	Italy	Italy								0.78±0.1	34
Orange	*Citrus sinensis L. var. Bionda*	A03AM	Juice, orange	Italy	Italy						12		49 (12:0;14:0) + 98 (14:0;14:0) + 140 (14:0;16:0)+ 69 (16:0; 16:0)	36
Orange	*Citrus sinensis L. var. Brasiliana*	A03AM	Juice, orange	Italy	Italy								38 (12:0;12:0) + 105 (12:0;14:0) + 85 (14:0;14:0); 83 (14:0;16:0)+ 71 (16:0; 16:0)	36
Orange	*Citrus sinensis L. var. Moro*	A03AM	Juice, orange	Italy	Italy						11		31 (14:0), 22 (16:0), 32 (12:0;12:0) + 96 (12:0;14:0) + 108 (14:0;14:0) + 105 (14:0;16:0)+ 67 (16:0; 16:0)	36
Orange	*Citrus sinensis L. var. Ovale*	A03AM	Juice, orange	Italy	Italy								12 + 14 (10:0) + 42 (12:0) + 20 (16:0) + 29 (12:0;12:0) + 74 (12:0;14:0) + 53 (14:0;14:0); 66 (14:0;16:0)+ 81 (16:0; 16:0)	36
Orange	*Citrus sinensis L. var. Sanguinello*	A03AM	Juice, orange	Italy	Italy						8		13 + 20 (10:0) + 13 (12:0) +56 (14:0) + 24 (16:0) + 38 (12:0;12:0) + 101 (12:0;14:0) + 88 (14:0;14:0) + 257 (14:0;16:0) + 92 (16:0; 16:0)	36

Table S4.1.4 Fruit juices (100% from named source) (A0BY4) (µg/100g) (continuation)

Food name	Scientific name	FoodEx2_ TermCode	FoodEx2_ TermName	Origin (country)	Purchase (country)	Water (%)	Process	Colour	Neochrome	Neoxanthin	Phytoene	Phytofluene	Violaxanthin	Ref.
Orange	*Citrus sinensis L. var. Tarocco*	A03AM	Juice, orange	Italy	Italy								10 + 32 (12:0;12:0) + 68 (12:0;14:0) + 59 (14:0;14:0) + 130 (14:0;16:0) + 60 (16:0; 16:0)	36
Orange	*Citrus sinensis L. var. Valence*	A03AM	Juice, orange	Italy	Italy						13		37 + 11 (10:0) + 80 (12:0) + 252 (14:0) + 209 (16:0) + 46 (12:0;12:0) + 210 (12:0;14:0) + 113 (14:0;14:0) + 92 (14:0;16:0) + 72 (16:0; 16:0)	36
Orange	*Citrus sinensis L. var. Washinghton*	A03AM	Juice, orange	Italy	Italy								10 (14:0) + 12 (12:0;12:0) + 14 (12:0;14:0) + 22 (14:0;14:0) + 23 (14:0;16:0) + 15 (16:0; 16:0)	36
Orange	*Citrus sinensis L.*	A03AM	Juice, orange	Spain	Spain						80 (50-100)	70 (50-100)		22
Pear	*Pyrus cv Conference*	A0BY4#F27.A01DP$F22.A07SH	Fruit juices (100% from named source), SOURCE-COMMODITIES = Pears, PREPARATION-PRODUCTION-PLACE = Food industry prepared	Spain	Spain	80 - 90					1.1±0.0			39
Pear	*Pyrus cv Blanquilla*	A0BY4#F27.A01DP$F22.A07SH	Fruit juices (100% from named source), SOURCE-COMMODITIES = Pears, PREPARATION-PRODUCTION-PLACE = Food industry prepared	Spain	Spain	80 - 90					0.8±0			39

Table S4.1.4 Fruit juices (100% from named source) (A0BY4) (µg/100g) (continuation)

Food name	Scientific name	FoodEx2_ TermCode	FoodEx2_ TermName	Origin (country)	Purchase (country)	Water (%)	Process	Colour	Neochrome	Neoxanthin	Phytoene	Phytofluene	Violaxanthin	Ref.
Pear	*Pyrus cv Conference*	A0BY4#F27.A 01DP	Fruit juices (100% from named source), SOURCE-COMMODITIES = Pears	Spain	Spain	80 - 90	freshly squeezed juice			2.6 ± 0.3 (all-E) 2.3 ± 0.1 (9'Z)	1.8±0.3		2.3 ± 0.2 (all-E) 2.2 ± 0.1 (9Z)	39
Pear	*Pyrus cv Blanquilla*	A0BY4#F27.A 01DP	Fruit juices (100% from named source), SOURCE-COMMODITIES = Pears	Spain	Spain	80 - 90	freshly squeezed juice			3.3 ± 0.1 (all-E) 2.5 ± 0.1 (9'Z)			2.6 ± 0.3 (all-E) 2.4 ± 0.1 (9Z)	39

Table S4.1.5 Fruit juices (100% from named source) (A0BY4) (µg/100g) (continuation)

Food name	Scientific name	FoodEx2_ TermCode	FoodEx2_ TermName	Origin (country)	Purchase (country)	Water (%)	Process	Colour	Z(v. cis)-lycopene	Z(v. cis)-β-carotene	Z(v. cis)-β-cryptoxanthin	Zeaxanthin	Zeinoxanthin	Ref.
Mandarin Willow leaf	*Citrus deliciosa Ten*	A0BY4#F27.A 01CD	Fruit juices (100% from named source), SOURCE-COMMODITIES = Mandarins	France	France							106		29
Mandarin Willow leaf	*Citrus deliciosa Ten*	A0BY4#F27.A 01CD	Fruit juices (100% from named source), SOURCE-COMMODITIES = Mandarins	France	France							110		29
Orange	*Citrus sinensis L. var. navel lane late*	A03AM	Juice, orange	Portugal	Portugal			orange				130±		32
Orange	*Citrus sinensis Osbeck*	A03AM	Juice, orange	Italy	Italy					0.06±0.01		2.18±0.25	0.37±0.08	34

Table S4.1.5 Fruit juices (100% from named source) (A0BY4) (μg/100g) (continuation)

Food name	Scientific name	FoodEx2_ TermCode	FoodEx2_ TermName	Origin (country)	Purchase (country)	Water (%)	Process	Colour	Z(v. cis)-lycopene	Z(v. cis)-β-carotene	Z(v. cis)-β-cryptoxanthin	Zeaxanthin	Zeinoxanthin	Ref.
Orange	*Citrus sinensis Osbeck*	A03AM	Juice, orange	Italy	Italy					0.07±0.01		1.62±0.32	0.41±0.06	34
Orange	*Citrus sinensis Osbeck*	A03AM	Juice, orange	Italy	Italy					0.04		1.06±0.12	0.35±0.06	34
Orange	*Citrus sinensis Osbeck*	A03AM	Juice, orange	Italy	Italy					0.01		0.92±0.12	0.18±0.08	34
Orange	*Citrus sinensis*	A03AM	Juice, orange	Brazil				orange				14-362		30
Orange	*Citrus sinensis var. Valencia*	A03AM#F28. A07HV	Juice, orange, PROCESS = Pasteurisation	Spain	Spain		pasteurized					3.61±1.03		33
Orange	*Citrus sinensis var. Valencia*	A03AM#F28. A07LJ	Juice, orange, PROCESS = Homogenizing or emulsifying	Spain	Spain		ultra high pressure homogenization					4.52±1.41		33
Orange	*Citrus sinensis var. Valencia*	A03AM#F28. A07LJ	Juice, orange, PROCESS = Homogenizing or emulsifying	Spain	Spain		ultra high pressure homogenization					4.53±1.36		33
Orange	*Citrus sinensis var. Valencia*	A03AM#F28. A07LJ	Juice, orange, PROCESS = Homogenizing or emulsifying	Spain	Spain		ultra high pressure homogenization					4.29±1.35		33
Orange	*Citrus sinensis L. var. valencia late*	A03AM	Juice, orange	Spain	Spain		hand squeezed	orange				252±33	70±7	37
Orange	*Citrus sinensis L. var. valencia late*	A03AM	Juice, orange	Spain	Spain			orange				219±28	61±8	37
Orange	*Citrus sinensis L. var. valencia late*	A03AM#F28. A07HV	Juice, orange, PROCESS = Pasteurisation	Spain	Spain		pasteurization	orange				193±17	56±4	37
Orange	*Citrus sinensis*	A03AM	Juice, orange	USA						0				28
Orange	-	A03AM#F28. A07MR	Juice, orange, PROCESS = Reconstitution from concentrate, powder or other dehydrated form	Spain	Spain		from concentrated	orange		(nd - 17)	(nd - 17)	35 (8-53)±11	7 (4-13)±2	31

Table S4.1.5 Fruit juices (100% from named source) (A0BY4) (µg/100g) (continuation)

Food name	Scientific name	FoodEx2_ TermCode	FoodEx2_ TermName	Origin (country)	Purchase (country)	Water (%)	Process	Colour	Z(v. cis)-lycopene	Z(v. cis)-β-carotene	Z(v. cis)-β-cryptoxanthin	Zeaxanthin	Zeinoxanthin	Ref.
Orange	*Citrus sinensis var. Valencia*	A03AM	Juice, orange	Spain	Spain		raw					4.75±1.10		33
Pear	*Pyrus cv Conference*	A0BY4#F27.A01DP$F22.A07SH	Fruit juices (100% from named source), SOURCE-COMMODITIES = Pears, PREPARATION-PRODUCTION-PLACE = Food industry prepared	Spain	Spain	80 - 90						1.6 (all-E)		39
Pear	*Pyrus cv Blanquilla*	A0BY4#F27.A01DP$F22.A07SH	Fruit juices (100% from named source), SOURCE-COMMODITIES = Pears, PREPARATION-PRODUCTION-PLACE = Food industry prepared	Spain	Spain	80 - 90						2.0 (all-E)		39
Pear	*Pyrus cv Conference*	A0BY4#F27.A01DP	Fruit juices (100% from named source), SOURCE-COMMODITIES = Pears	Spain	Spain	80 - 90	freshly squeezed juice					3.0 (all-E)±0.2		39
Pear	*Pyrus cv Blanquilla*	A0BY4#F27.A01DP	Fruit juices (100% from named source), SOURCE-COMMODITIES = Pears	Spain	Spain	80 - 90	freshly squeezed juice					4.9 (all-E)±0.4		39

Table S4.2.1 Vegetable juices (A04PQ) (µg/100g)

Food name	Scientific name	FoodEx2_ TermCode	FoodEx2_ TermName	Origin (country)	Purchase (country)	Water (%)	Process	Colour	α-carotene	β-carotene	β-cryptoxanthin	ζ-carotene	Antheraxanthin	Ref.
Fruit–soymilk		A03DE#F04.A03TJ	Mixed juices with added ingredients, INGREDIENT = Soya drink	Spain	Spain				6-9	36-50	5-8		15-16	40
Fruit–soymilk		A03DE#F04.A02LV$F04.A03TJ	Mixed juices with added ingredients, INGREDIENT = Cow milk, INGREDIENT = Soya drink	Spain	Spain					5.8-309				41
Fruit–soymilk	*Glycine max*	A03DE#F04.A03TJ	Mixed juices with added ingredients, INGREDIENT = Soya drink	Spain	Spain					342.3±17.6				42
Tomato	*Lycopersicon esculentum M.*	A03CJ	Juice, tomato	Italy	Italy					369				25
Tomato	*Lycopersicon esculentum M.*	A03CJ	Juice, tomato	Spain	Spain			red		423				24
Tomato	*Lycopersicon esculentum mill.*	A03CJ	Juice, tomato	Spain	Spain			red		423				24

Table S4.2.2 Vegetable juices (A04PQ) (µg/100g) (continuation)

Food name	Scientific name	FoodEx2_TermCode	FoodEx2_TermName	Origin (country)	Purchase (country)	Water (%)	Process	Colour	Auroxanthin	E(v. trans)-α-carotene	E(v. trans)-β-carotene	E(v. trans)-lutein	E(v. trans)-lycopene	Ref.
Tomato	*Lycopersicon esculentum M.*	A03CJ	Juice, tomato	Germany	Germany						5770		21400	27
Tomato	*Lycopersicon esculentum M.*	A03CJ	Juice, tomato	Germany	Germany						4220		133600	27
Tomato	*Lycopersicon esculentum M.*	A03CJ	Juice, tomato	Germany	Germany						4030		159400	27
Tomato	*Lycopersicon esculentum M.*	A03CJ	Juice, tomato	Germany	Germany						3760		150700	27

Table S4.2.3 Vegetable juices (A04PQ) (µg/100g) (continuation)

Food name	Scientific name	FoodEx2_TermCode	FoodEx2_TermName	Origin (country)	Purchase (country)	Water (%)	Process	Colour	E(v. trans)-zeaxanthin	Lutein	Luteoxanthin	Lycopene	Mutatoxanthin	Ref.
Fruit-milk/soya		A03DE#F04.A02LV$F04.A03TJ	Mixed juices with added ingredients, INGREDIENT = Cow milk, INGREDIENT = Soya drink	Spain	Spain					0.5-1.5				41
Fruit–soymilk		A03DE#F04.A03TJ	Mixed juices with added ingredients, INGREDIENT = Soya drink	Spain	Spain					66-108				40
Tomato	*Lycopersicon esculentum M.*	A03CJ	Juice, tomato	Italy	Italy					29		1024–11 000		25
Tomato	*Lycopersicon esculentum M.*	A03CJ	Juice, tomato	Spain	Spain			red				9675		24
Tomato	*Lycopersicon esculentum mill.*	A03CJ	Juice, tomato	Spain	Spain			red				9675		24
Watermelon	*Citrullus lanatus var. Seedless*	A04PQ#F27.A00KJ	Vegetable juices, SOURCE-COMMODITIES = Watermelons	Spain	Spain							6.2		43

Table S4.2.4 Vegetable juices (A04PQ) (µg/100g) (continuation)

Food name	Scientific name	FoodEx2_ TermCode	FoodEx2_ TermName	Origin (country)	Purchase (country)	Water (%)	Process	Colour	Neochrome	Neoxanthin	Phytoene	Phytofluene	Violaxanthin	Ref.
Carrot	*Daucus carota*	A03CK	Juice, carrot	Spain	Spain		juice	orange			1230	550		22
Tomato	*Solanum lycopersicum Mill.*	A03CJ	Juice, tomato	Spain	Spain		juice	red			1770 (1640-1900)	630 (440-830)		22

Table S4.2.5 Vegetable juices (A04PQ) (µg/100g) (continuation)

Food name	Scientific name	FoodEx2_ TermCode	FoodEx2_ TermName	Origin (country)	Purchase (country)	Water (%)	Process	Colour	Z(v. cis)-lycopene	Z(v. cis)-β-carotene	Z(v. cis)-β-cryptoxanthin	Zeaxanthin	Zeinoxanthin	Ref.
Fruit-milk/soya		A03DE#F04.A02LV$F04.A03TJ	Mixed juices with added ingredients, INGREDIENT = Cow milk, INGREDIENT = Soya drink	Spain	Spain							3.7-9.8		41
Fruit–soymilk		A03DE#F04.A03TJ	Mixed juices with added ingredients, INGREDIENT = Soya drink	Spain	Spain							56-76		40
Tomato	*Licopersicon esculentum Mill. cv. Daniella*	A03CJ	Juice, tomato	Spain	Spain			L* = 38.5 ± 0.4; a* = 18.1 ± 1.9; b* = 24.6 ± 1.8	630 - 660					44

Table S5. Animal and vegetable fats and oils and primary derivatives thereof (A036M)

Table S5.1.1 Olive oils (A036P) (µg/100g)

Food name	Scientific name	FoodEx2_ TermCode	FoodEx2_ TermName	Origin (country)	Purchase (country)	Water (%)	Part analysed	Colour	β-carotene	β-cryptoxanthin	Antheraxanthin	E(v. trans)-β-carotene	Lutein	Ref.
Extra virgin olive oils	*Olea europaea, L.*	A036P	Olive oils	Italy	Italy			purple; green-purple	123±4				119±13	45
Extra virgin olive oils	*Olea europaea, L.*	A036P	Olive oils	Greece	Greece			green; Light green	255.5±3.5				248±15.5	45
Extra virgin olive oils	*Olea europaea, L.*	A036P	Olive oils	Tunisia	Tunisia			small reddish spots; green-purple; Purple; Black	138.5±5				194.8±12.3	45
Extra virgin olive oils	*Olea europaea, L.*	A036P	Olive oils	Spain	Spain			light green; small reddish spots; Purple; Black	195.5±1.7				534.2±8.3	45
Extra virgin olive oils	*Olea europaea, L.*	A036Q	Olive oil, virgin or extra-virgin	Italy	Italy				230	nd			350	25
Olive oil	*Olea europaea L. cv. Picual*	A036P	Olive oils	Spain	Spain	-		green		3.0-5.8	17-25		47-54	46
Olive oil	*Olea europaea L.*	A036Q	Olive oil, virgin or extra-virgin	Spain	Spain			yellow	29.1±14.2				18419.9±998 1.5	47
Virgin olive oil	*Olea europaea L. cv. Picual*	A036Q	Olive oil, virgin or extra-virgin	Spain	Spain		a fat-free pigment extract		233		40		934	46
Virgin olive oil	*Olea europaea L. cv. Picudo*	A036Q	Olive oil, virgin or extra-virgin	Spain	Spain		a fat free pigment extract		240		36		687	46
Virgin olive oil	*Olea europaea L. cv. Subbetica*	A036Q	Olive oil, virgin or extra-virgin	Spain	Spain		a fat free pigment extract		223		45		526	46
Virgin olive oil	*Olea europaea L. cv. Hojiblanca*	A036Q	Olive oil, virgin or extra-virgin	Spain	Spain		a fat free pigment extract		219		31		599	46
Virgin olive oil	*Olea europaea L.*	A036Q	Olive oil, virgin or extra-virgin	Spain	Spain		a fat free pigment extract		108		16		178	46

Table S5.1.1 Olive oils (A036P) (µg/100g) (continuation)

Food name	Scientific name	FoodEx2_ TermCode	FoodEx2_ TermName	Origin (country)	Purchase (country)	Water (%)	Part analysed	Colour	β-carotene	β-cryptoxanthin	Antheraxanthin	E(v. trans)-β-carotene	Lutein	Ref.
Virgin olive oil	*Olea europaea L. cv. hojiblanca*	A036Q	Olive oil, virgin or extra-virgin	Spain	Spain			yellow	219				599	24
Virgin olive oil	*Olea europaea L. cv. Picual*	A036Q	Olive oil, virgin or extra-virgin	Spain	Spain			yellow	233				934	24
Virgin olive oil	*Olea europaea, L.*	A036Q	Olive oil, virgin or extra-virgin	Spain	Spain				219 - 233				599 - 934	24
Virgin olive oil	*Olea europaea, L. cv. Arbequina*	A036Q	Olive oil, virgin or extra-virgin	Spain	Spain			L*92.07±0.03a* −5.22±0.00b*77 .90±0.04			16.5±1.9	12.3±3.9	154±6.9	48
Virgin olive oil	*Olea europaea, L. cv. Arbequina*	A036Q	Olive oil, virgin or extra-virgin	Spain	Spain			L*86.21±0.29 a*−1.64±0.00 b*102.20±37.00			37.1±2.7	46.1±0.7	238±13.5	48
Virgin olive oil	*Olea europaea, L. cv. Arbequina*	A036Q	Olive oil, virgin or extra-virgin	Spain	Spain			L*85.83±0.10 a*−1.80±0.01 b*98.15±0.12			27.5±4.5	49±6	262.5±4.5	48

Table S5.1.2 Olive oils (A036P) (µg/100g) (continuation)

Food name	Scientific name	FoodEx2_ TermCode	FoodEx2_ TermName	Origin (country)	Purchase (country)	Water (%)	Part analysed	Colour	Luteoxanthin	Mutatoxanthin	Neoxanthin	Violaxanthin	Ref.
Olive oil	*Olea europaea L.* ***Picual***	A036P	Olive oils	Spain	Spain	-		green			2.5-4.0	50-55.8	46
Virgin olive oil	*Olea europaea L. cv. Picual*	A036Q	Olive oil, virgin or extra-virgin	Spain	Spain		a fat-free pigment extract		14	11	73	18	46
Virgin olive oil	*Olea europaea L. cv. Picudo*	A036Q	Olive oil, virgin or extra-virgin	Spain	Spain		a fat free pigment extract		0	0	44	42	46
Virgin olive oil	*Olea europaea L. cv. Subbetica*	A036Q	Olive oil, virgin or extra-virgin	Spain	Spain		a fat free pigment extract		0	0	54	64	46

Table S5.1.2 Olive oils (A036P) (µg/100g) (continuation)

Food name	Scientific name	FoodEx2_ TermCode	FoodEx2_ TermName	Origin (country)	Purchase (country)	Water (%)	Part analysed	Colour	Luteoxanthin	Mutatoxanthin	Neoxanthin	Violaxanthin	Ref.
Virgin olive oil	*Olea europaea L. cv. Hojiblanca*	A036Q	Olive oil, virgin or extra-virgin	Spain	Spain		a fat free pigment extract	/	11	5	33	19	46
Virgin olive oil	*Olea europaea olive L.*	A036Q	Olive oil, virgin or extra-virgin	Spain	Spain		a fat free pigment extract	/	0	0	9	6	46
Virgin olive oil	*Olea europaea, L. cv. Arbequina*	A036Q	Olive oil, virgin or extra-virgin	Spain	Spain			L*92.07±0.03a*−5 .22±0.00b*77.90± 0.04			12.1±0.4	31.3±5.2	48
Virgin olive oil	*Olea europaea, L. cv. Arbequina*	A036Q	Olive oil, virgin or extra-virgin	Spain	Spain			L*86.21±0.29 a*−1.64±0.00 b*102.20±37.00			30.2±2.2	71.3±4.7	48
Virgin olive oil	*Olea europaea, L., cv. Arbequina*	A036Q	Olive oil, virgin or extra-virgin	Spain	Spain			L*85.83±0.10 a*−1.80±0.01 b*98.15±0.12			24±4	61.5±8.5	48

Table S5.2 Margarines and similar (A0F1G) (µg/100g)

Food name	Scientific name	FoodEx2_ TermCode	FoodEx2_ TermName	Origin (country)	Purchase (country)	Water (%)	Part analysed	Colour	β-carotene	β-cryptoxanthin	Antheraxanthin	E(v. trans)-β-carotene	Lutein	Ref.
Margarine	-	A0F1G	Margarines and similar	Spain	Spain			yellow	540					24
Margarine	-	A0F1G	Margarines and similar	Spain	Spain				540					24

Table S6. Fruit and fruit products (A01BS)

Table S6.1.1 Citrus fruits (A01BT) (µg/100g)

Food name	Scientific name	FoodEx2_TermCode	FoodEx2_TermName	Origin (country)	Purchase (country)	Water (%)	Process	Saponification	Part analysed	Colour	α-carotene	β-carotene	β-cryptoxanthin	ζ-carotene	Antheraxanthin	Ref.
Citrus fruits	-	A01BT	Citrus fruits	Germany	Indonesia				peel and pulp	Orange	100±10	40-240	30-1020		190-640	49

Table S6.1.2 Citrus fruits (A01BT) (µg/100g) (continuation)

Food name	Scientific name	FoodEx2_TermCode	FoodEx2_TermName	Origin (country)	Purchase (country)	Water (%)	Process	Saponification	Part analysed	Colour	E(v. trans)-lycopene	E(v. trans)-zeaxanthin	Lutein	Luteoxanthin	Lycopene	Ref.
Citrus fruits	-	A01BT	Citrus fruits	Germany	Indonesia				peel and pulp	Orange			20-960			49

Table S6.1.3 Citrus fruits (A01BT) (µg/100g) (continuation)

Food name	Scientific name	FoodEx2_TermCode	FoodEx2_TermName	Origin (country)	Purchase (country)	Water (%)	Process	Saponification	Part analysed	Colour	Violaxanthin	Z(v. cis)-lycopene	Z(v. cis)-β-carotene	Z(v. cis)-β-cryptoxanthin	Zeaxanthin	Zeinoxanthin	Ref.
Citrus fruits	-	A01BT	Citrus fruits	Germany	Indonesia				peel and pulp	Orange	110-1930				20-360		49

Table S6.2.1 Grapefruits and similar (A01CX) (µg/100g)

Food name	Scientific name	FoodEx2_ TermCode	FoodEx2_ TermName	Origin (country)	Purchase (country)	Water (%)	Process	Saponification	Part analysed	Colour	α-carotene	β-carotene	β-cryptoxanthin	ζ-carotene	Antheraxanthin	Ref.
Grapefruit	*Citrus paradisi*	A01CY	Grapefruits	Germany	Germany	89.8			edible part			590	12	13	13	53
Grapefruit	*Citrus paradisi*	A01CY	Grapefruits	Spain	Spain				edible part	yellow	6	5	3			24
Grapefruit	*Citrus paradisi*	A01CY	Grapefruits	Spain	Spain				edible part	pink		1310				24
Grapefruit	*Citrus paradisi*	A01CY	Grapefruits	Spain	Spain				edible part	yellow	6	5	3			24
Grapefruit	*Citrus paradisi*	A01CY	Grapefruits	Spain	Spain				edible part	pink		1310				24
Grapefruit	*C. paradisi cv. Marsh*	A01CY	Grapefruits	China	China		lyophilized	yes	pulp	yellow/ orange/red	nd	35±3	nd			51
Grapefruit	*C. paradisi cv. Cock Tail*	A01CY	Grapefruits	China	China		lyophilized	yes	pulp	yellow/ orange/red	nd	258±16.3	128±3			51
Grapefruit	*C. paradisi cv. Thompson*	A01CY	Grapefruits	China	China		lyophilized	yes	pulp	yellow/ orange/red	nd	377±9	113±15			51
Grapefruit	*C. paradisi cv. red Blush*	A01CY	Grapefruits	China	China		lyophilized	yes	pulp	yellow/ orange/red	nd	811±6	nd			51
Grapefruit	*C. paradesi cv. Rio red*	A01CY	Grapefruits	China	China		lyophilized	yes	pulp	yellow/ orange/red	134±1	6466±21	100±1			51

Table S6.2.1 Grapefruits and similar (A01CX) (μg/100g)

Food name	Scientific name	FoodEx2_ TermCode	FoodEx2_ TermName	Origin (country)	Purchase (country)	Water (%)	Process	Saponification	Part analysed	Colour	α-carotene	β-carotene	β-cryptoxanthin	ζ-carotene	Antheraxanthin	Ref.
Grapefruit	*C. paradisi Macf. cv. Marsh*	A01CY	Grapefruits	South África	United Kingdom			yes	peel			0.82	16	5.4		52
Grapefruit	*C. paradisi Macf. cv. Marsh*	A01CY	Grapefruits	South África	United Kingdom			yes	juice vesicle				1.77	1.1		52
Grapefruit	*C. paradisi Macf. cv. Rub red*	A01CY	Grapefruits	South África	United Kingdom			yes	peel			84	45	86		52
Grapefruit	*C. paradisi Macf. cv. Rubi red*	A01CY	Grapefruits	South África	United Kingdom			yes	juice vesicle			293	20	215		52
Grapefruit	*C. paradisi Macf. cv. Star rubi*	A01CY	Grapefruits	South África	United Kingdom			yes	peel			1397		1072		52
Grapefruit	*C. paradisi Macf. cv. Star rubi*	A01CY	Grapefruits	South África	United Kingdom			yes	juice vesicle			4311		215		52
Grapefruit	*C. paradis cv. Oroblanco*	A0CFB	Sweeties	China			lyophilized	yes	pulp	yellow/ orange/red	nd	24±1.4	nd			51
Pomelos	*C. grandis L. Osbeck cv. Yuhuan*	A01DB	Pomelos	China	United Kingdom			yes	peel		11	54	107	103		52
Pomelos	*C. grandis L. Osbeck cv. Yuhuan*	A01DB	Pomelos	China	United Kingdom			yes	juice vesicle		26	77	19			52
Pomelos	*C. grandis L. Osbeck cv. Chuzhou Early red*	A01DB	Pomelos	China	United Kingdom			yes	peel		0.44	1	0.34			52
Pomelos	*C. grandis L. Osbeck cv. Chuzhou Early red*	A01DB	Pomelos	China	United Kingdom			yes	juice vesicle		47	632				52

Table S6.2.2 Grapefruits and similar (A01CX) (µg/100g) (continuation)

Food name	Scientific name	FoodEx2_ TermCode	FoodEx2_ TermName	Origin (country)	Purchase (country)	Water (%)	Process	Saponification	Part analysed	Colour	Auroxanthin	E(v. trans)-α-carotene	E(v. trans)-β-carotene	E(v. trans)-β-cryptoxanthin	E(v. trans)-lutein	Ref.
Grapefruit	*C. paradisi Macf.* ***cv. Marsh***	A01CY	Grapefruits	South África	United Kingdom			yes	peel		61					52

Table S6.2.3 Grapefruits and similar (A01CX) (µg/100g) (continuation)

Food name	Scientific name	FoodEx2_ TermCode	FoodEx2_ TermName	Origin (country)	Purchase (country)	Water (%)	Process	Saponification	Part analysed	Colour	E(v. trans)-lycopene	E(v. trans)-zeaxanthin	Lutein	Luteoxanthin	Lycopene	Ref.
Grapefruit	*Citrus paradisi*	A01CY	Grapefruits	Italy	Italy					**red**					750	25
Grapefruit	*Citrus paradisi*	A01CY	Grapefruits	Germany	Germany	89.8			edible part				20			53
Grapefruit	*Citrus paradisi*	A01CY	Grapefruits	Spain	Spain				edible part	Pink					3362	24
Grapefruit	*Citrus paradisi*	A01CY	Grapefruits	Spain	Spain				edible part	Pink					3362	24
Grapefruit	*Citrus paradisi cv. ruby*	A01CY	Grapefruits	Finland	Finland				pulp	red	5370					26
Grapefruit	*Citrus paradisi*	A01CY	Grapefruits	Finland	Finland				pulp	red	430					26
Grapefruit	*C. paradisi cv. Marsh*	A01CY	Grapefruits	China			lyophilized	yes	pulp	yellow/ orange/red			109±4		25±3	51
Grapefruit	*C. paradisi cv. Cock Tail*	A01CY	Grapefruits	China			lyophilized	yes	pulp	yellow/ orange/red			111±3		nd	51
Grapefruit	*C. paradisi cv. Thompson*	A01CY	Grapefruits	China			lyophilized	yes	pulp	yellow/ orange/red			106±1		nd	51
Grapefruit	*C. paradisi cv. red Blush*	A01CY	Grapefruits	China			lyophilized	yes	pulp	yellow/ orange/red			105±1		435±26	51
Grapefruit	*C. paradisi cv. Rio red*	A01CY	Grapefruits	China			lyophilized	yes	pulp	yellow/ orange/red			107±1		566±20	51

Table S6.2.3 Grapefruits and similar (A01CX) (µg/100g) (continuation)

Food name	Scientific name	FoodEx2_ TermCode	FoodEx2_ TermName	Origin (country)	Purchase (country)	Water (%)	Process	Saponification	Part analysed	Colour	E(v. trans)-lycopene	E(v. trans)-zeaxanthin	Lutein	Luteoxanthin	Lycopene	Ref.
Grapefruit	*C. paradisi Macf. cv. Rio red*	A01CY	Grapefruits	USA				no	juice						870	54
Grapefruit	*C. paradisi Macf. cv. Rio red*	A01CY	Grapefruits	USA				no	juice						520	54
Grapefruit	*C. paradisi Macf. cv. Rio red*	A01CY	Grapefruits	USA				no	juice						480	54
Grapefruit	*C. paradisi Macf. cv. Rio red*	A01CY#F21. A07SE	Grapefruits, PRODUCTION -METHOD = Organic production	USA				no	juice						520	54
Grapefruit	*C. paradisi Macf. cv. Rio red*	A01CY#F21. A07SE	Grapefruits, PRODUCTION -METHOD = Organic production	USA				no	juice						250	54
Grapefruit	*C. paradisi Macf. cv. Rio red*	A01CY#F21. A07SE	Grapefruits, PRODUCTION -METHOD = Organic production	USA				no	juice						300	54
Grapefruit	*C. paradisi Macf. cv. Marsh*	A01CY	Grapefruits	South África	United Kingdom			yes	peel					31		52
Grapefruit	*C. paradisi Macf. cv. Rubi red*	A01CY	Grapefruits	South África	United Kingdom			yes	peel					63	31	52
Grapefruit	*C. paradisi Macf. cv. Rubi red.*	A01CY	Grapefruits	South África	United Kingdom			yes	juice vesicle						16	52
Grapefruit	*C. paradisi Macf. cv. Star rubi*	A01CY	Grapefruits	South África	United Kingdom			yes	peel					57	4232	52
Grapefruit	*C. paradisi Macf. cv. Star rubi*	A01CY	Grapefruits	South África	United Kingdom			yes	juice vesicle						15935	52

Table S6.2.3 Grapefruits and similar (A01CX) (µg/100g) (continuation)

Food name	Scientific name	FoodEx2_ TermCode	FoodEx2_ TermName	Origin (country)	Purchase (country)	Water (%)	Process	Saponification	Part analysed	Colour	E(v. trans)-lycopene	E(v. trans)-zeaxanthin	Lutein	Luteoxanthin	Lycopene	Ref.
Grapefruit	*C. paradis cv. Oroblanco*	A0CFB	Sweeties	China			lyophilized	yes	pulp	yellow/ orange/red			105±1		nd	51
Pomelos	*C. grandis L. Osbeck cv. Yuhuan*	A01DB	Pomelos	China	United Kingdom			yes	peel					61		52
Pomelos	*C. grandis L. Osbeck cv. Yuhuan*	A01DB	Pomelos	China	United Kingdom			yes	juice vesicle						98	52
Pomelos	*C. grandis L. Osbeck cv. Chuzhou Early red*	A01DB	Pomelos	China	United Kingdom			yes	juice vesicle						1607	52

Table S6.2.4 Grapefruits and similar (A01CX) (µg/100g) (continuation)

Food name	Scientific name	FoodEx2_ TermCode	FoodEx2_ TermName	Origin (country)	Purchase (country)	Water (%)	Process	Saponification	Part analysed	Colour	Mutatoxanthin	Neochrome	Neoxanthin	Phytoene	Phytofluene	Ref.
Grapefruit	*C. paradisi Macf. cv. Marsh*	A01CY	Grapefruits	South África	United Kingdom			yes	peel					99	20	52
Grapefruit	*C. paradisi Macf. cv. Marsh*	A01CY	Grapefruits	South África	United Kingdom			yes	juice vesicle					7	1.7	52
Grapefruit	*C. paradisi Macf. cv. Rubi red*	A01CY	Grapefruits	South África	United Kingdom			yes	peel					1034	451	52
Grapefruit	*C. paradisi Macf. cv. Rubi red*	A01CY	Grapefruits	South África	United Kingdom			yes	juice vesicle					148	33	52
Grapefruit	*C. paradisi Macf. cv. Star rubi*	A01CY	Grapefruits	South África	United Kingdom			yes	peel					10177	3877	52

Table S6.2.4 Grapefruits and similar (A01CX) (µg/100g) (continuation)

Food name	Scientific name	FoodEx2_ TermCode	FoodEx2_ TermName	Origin (country)	Purchase (country)	Water (%)	Process	Saponification	Part analysed	Colour	Mutatoxanthin	Neochrome	Neoxanthin	Phytoene	Phytofluene	Ref.
Grapefruit	*C. paradisi Macf. cv. Star rubi*	A01CY	Grapefruits	South África	United Kingdom			yes	juice vesicle					6552	3328	52
Grapefruit	*Citrus paradisi*	A01CY	Grapefruits	Germany	Germany	89.8			edible part					70	60	53
Grapefruit	*Citrus paradisi*	A01CY	Grapefruits	Spain	Spain				pulp	orange				70	60	22
Grapefruit	*Citrus paradisi*	A01CY	Grapefruits	Spain	Spain				pulp	red				1250 (250-5130)	510 (170-1690)	22
Grapefruit	*Citrus paradisi*	A01CY	Grapefruits	Spain	Spain				pulp	pink				320 (10-620)	110 (10-210)	22
Pomelos	*C. grandis L. Osbeck cv. Yuhuan*	A01DB	Pomelos	China	United Kingdom			yes	peel					3001	726	52
Pomelos	*C. grandis L. Osbeck cv. Yuhuan*	A01DB	Pomelos	China	United Kingdom			yes	juice vesicle					519	427	52
Pomelos	*C. grandis L. Osbeck cv. Chuzhou Early red*	A01DB	Pomelos	China	United Kingdom			yes	peel					7.3	0.49	52
Pomelos	*C. grandis L. Osbeck cv. Chuzhou Early red*	A01DB	Pomelos	China	United Kingdom			yes	juice vesicle					174	43	52

Table S6.2.5 Grapefruits and similar (A01CX) (µg/100g) (continuation)

Food name	Scientific name	FoodEx2_ TermCode	FoodEx2_ TermName	Origin (country)	Purchase (country)	Water (%)	Process	Saponification	Part analysed	Colour	Violaxanthin	Z(v. cis)-lycopene	Z(v. cis)-β-carotene	Z(v. cis)-β-cryptoxanthin	Zeaxanthin	Zeinoxanthin	Ref.
Grapefruit	C. paradescv. Rio red	A01CY	Grapefruits	China			lyophilized	yes	pulp	yellow/ orange/ red					46±3		51
Grapefruit	C. paradis cv. Cock Tail	A01CY	Grapefruits	China			lyophilized	yes	pulp	yellow/ orange/ red					93±4		51
Grapefruit	C. paradis cv. Marsh	A01CY	Grapefruits	China			lyophilized	yes	pulp	yellow/ orange/ red					nd		51
Grapefruit	C. paradis cv. Oroblanco	A0CFB	Sweeties	China			lyophilized	yes	pulp	yellow/ orange/ red					nd		51
Grapefruit	C. paradis cv. red Blush	A01CY	Grapefruits	China			lyophilized	yes	pulp	yellow/ orange/ red					nd		51
Grapefruit	C. paradis cv. Thompson	A01CY	Grapefruits	China			lyophilized	yes	pulp	yellow/ orange/ red					87±5		51
Grapefruit	C. paradisi Macf. cv. Marsh	A01CY	Grapefruits	South África	United Kingdom			yes	peel		0.5				4.6		52
Grapefruit	C. paradisi Macf. cv. Marsh	A01CY	Grapefruits	South África	United Kingdom			yes	juice vesicle						7.4		52
Grapefruit	C. paradisi Macf. cv. Rubi red	A01CY	Grapefruits	South África	United Kingdom			yes	peel		166				15		52
Grapefruit	C. paradisi Macf. cv. Rubi red	A01CY	Grapefruits	South África	United Kingdom			yes	juice vesicle						31		52
Grapefruit	C. paradisi Macf. cv. Star rubi	A01CY	Grapefruits	South África	United Kingdom			yes	peel		10				23		52
Grapefruit	C. paradisi Macf. cv. Star rubi	A01CY	Grapefruits	South África	United Kingdom			yes	juice vesicle						113		52
Grapefruit	Citrus paradisi	A01CY	Grapefruits	Germany	Germany	89.8			edible part						9		53

Table S6.2.5 Grapefruits and similar (A01CX) (µg/100g) (continuation)

Food name	Scientific name	FoodEx2_ TermCode	FoodEx2_ TermName	Origin (country)	Purchase (country)	Water (%)	Process	Saponification	Part analysed	Colour	Violaxanthin	Z(v. cis)-lycopene	Z(v. cis)-β-carotene	Z(v. cis)-β-cryptoxanthin	Zeaxanthin	Zeinoxanthin	Ref.
Pomelos	C. grandis L. Osbeck cv Chuzhou Early red	A01DB	Pomelos	China	United Kingdom			yes	peel						0.02		52
Pomelos	C. grandis L. Osbeck cv Yuhuan	A01DB	Pomelos	China	United Kingdom			yes	peel						278		52
Pomelos	C. grandis L. Osbeck cv Yuhuan	A01DB	Pomelos	China	United Kingdom			yes	juice vesicle						6		52

Table S6.3.1 Oranges and similar (A01CP) (µg/100g)

Food name	Scientific name	FoodEx2_ TermCode	FoodEx2_ TermName	Origin (country)	Purchase (country)	Water (%)	Process	Saponification	Part analysed	Colour	α-carotene	β-carotene	β-cryptoxanthin	ζ-carotene	Antheraxanthin	Ref.
Orange	*C. sinensis L. Cara cara cv. Bonanza*	A01CR	Oranges, Sweet	China	United Kingdom			yes	peel			10	505	32		52
Orange	*C. sinensis L. Osbeck cv. Bonanza*	A01CR	Oranges, Sweet	China	United Kingdom			yes	peel				440	242		52
Orange	*C. sinensis L. Osbeck cv. Bonanza*	A01CR	Oranges, Sweet	China	United Kingdom			yes	juice vesicle				780	180		52
Orange	*C. sinensis L. Osbeck cv. Bonanza*	A01CR	Oranges, Sweet	China	United Kingdom			yes	juice vesicle			470	445	55		52
Orange	*Citrus sinensis (L) Osbeck var. Barnfield*	A01CR	Oranges, Sweet	Spain	Spain				juice fruit	dark orange	15.9	22.1	184.9		70.8 (isomers) 195.4 (9Z or 9'Z)	55
Orange	*Citrus sinensis (L) Osbeck var. Lane late*	A01CR	Oranges, Sweet	Spain	Spain				juice fruit	dark orange	21.7	16.3	146.8		93.7 (isomers) 140.3 (9Z or 9'Z)	55

Table S6.3.1 Oranges and similar (A01CP) (µg/100g) (continuation)

Food name	Scientific name	FoodEx2_ TermCode	FoodEx2_ TermName	Origin (country)	Purchase (country)	Water (%)	Process	Saponification	Part analysed	Colour	α-carotene	β-carotene	β-cryptoxanthin	ζ-carotene	Antheraxanthin	Ref.
Orange	*Citrus sinensis (L) Osbeck var. Nave late*	A01CR	Oranges, Sweet	Spain	Spain				juice fruit	dark orange	15.7	18.6	138.6		73.8 (isomers) 128.5 (9Z or 9'Z)	55
Orange	*Citrus sinensis (L.) Osbeck cv Pera*	A01CR	Oranges, Sweet	France	France				juice fruit			68	380			43
Orange	*Citrus sinensis (L.) Osbeck cv Pera*	A01CR	Oranges, Sweet	Brazil					juice fruit			26	46			43
Orange	*Citrus sinensis (L.) Osbeck cv Sanguinelli*	A01CR	Oranges, Sweet	France	France				juice fruit			37	418			43
Orange	*Citrus sinensis (L.) Osbeck cv Sanguinelli*	A01CR	Oranges, Sweet	France	France				juice fruit			10	47			43
Orange	*Citrus sinensis (L.) Osbeck cv Valencia*	A01CR	Oranges, Sweet	France	France				juice fruit			48	216			43
Orange	*Citrus sinensis (L.) Osbeck cv Valencia*	A01CR	Oranges, Sweet	France	France				juice fruit			14	92			43
Orange	*Citrus sinensis (L.) Osbeck cv Valencia*	A01CR	Oranges, Sweet	Costa Rica					juice fruit			19	56			43
Orange	*Citrus sinensis (L.) Osbeck cv Valencia*	A01CR	Oranges, Sweet	French Polynesia					juice fruit			8	11			43
Orange	*Citrus sinensis (L.) Osbeck cv Valencia*	A01CR	Oranges, Sweet	Cuba					juice fruit			12	10			43
Orange	*Citrus sinensis (L.) Osbeck var. AmberSweet*	A01CR	Oranges, Sweet	Spain	Spain				juice fruit	orange	nd	28.5	444.6		37.6 (isomers) 62.5 (9Z or 9'Z)	55
Orange	*Citrus sinensis (L.) Osbeck var. Barberina*	A01CR	Oranges, Sweet	Spain	Spain				juice fruit	orange	18.7	20.9	128.7		90.5 (isomers) 190.3 (9Z or 9'Z)	55
Orange	*Citrus sinensis (L.) Osbeck var. Cadenera*	A01CR	Oranges, Sweet	Spain	Spain				juice fruit	orange	13.8	5.9	65.0		41.9 (isomers) 39.8 (9Z or 9'Z)	55

Table S6.3.1 Oranges and similar (A01CP) (μg/100g) (continuation)

Food name	Scientific name	FoodEx2_ TermCode	FoodEx2_ TermName	Origin (country)	Purchase (country)	Water (%)	Process	Saponification	Part analysed	Colour	α-carotene	β-carotene	β-cryptoxanthin	ζ-carotene	Antheraxanthin	Ref.
Orange	*Citrus sinensis (L.) Osbeck var. Cara Cara*	A01CR	Oranges, Sweet	Spain	Spain				juice fruit	dark orange	15.4	179.9	132.2		44.7 (isomers) 65.4 (9Z or 9'Z)	55
Orange	*Citrus sinensis (L.) Osbeck var. Chislett*	A01CR	Oranges, Sweet	Spain	Spain				juice fruit	dark orange	15.5	23.3	137.3		64.2 (isomers) 166.3 (9Z or 9'Z)	55
Orange	*Citrus sinensis (L.) Osbeck var. Delta Valencia*	A01CR	Oranges, Sweet	Spain	Spain				juice fruit	orange	20.3	25.0	153.1		128.9 (isomers) 209.9 (9Z or 9'Z)	55
Orange	*Citrus sinensis (L.) Osbeck var. Fisher*	A01CR	Oranges, Sweet	Spain	Spain				juice fruit	dark orange	15.3	11.2	118.0		58.8 (isomers) 77.1 (9Z or 9'Z)	55
Orange	*Citrus sinensis (L.) Osbeck var. Foyos*	A01CR	Oranges, Sweet	Spain	Spain				juice fruit	dark orange	16.0	15.1	150.1		66.6 (isomers) 83.6 (9Z or 9'Z)	55
Orange	*Citrus sinensis (L.) Osbeck var. Fukumoto*	A01CR	Oranges, Sweet	Spain	Spain				juice fruit	dark orange	14.8	10.7	108.9		44.6 (isomers) 52.1 (9Z or 9'Z)	55
Orange	*Citrus sinensis (L.) Osbeck var. Hamlin*	A01CR	Oranges, Sweet	Spain	Spain				juice fruit	orange	15.7	15.3	179.9		112.9 (isomers) 170.3 (9Z or 9'Z)	55
Orange	*Citrus sinensis (L.) Osbeck var. Midknight Valencia*	A01CR	Oranges, Sweet	Spain	Spain				juice fruit	orange	18.3	28.2	173.0		95.2 (isomers) 211.9 (9Z or 9'Z)	55
Orange	*Citrus sinensis (L.) Osbeck var. Navelina*	A01CR	Oranges, Sweet	Spain	Spain				juice fruit	dark orange	15.5	18.4	181.3		69.1 (isomers) 94.8 (9Z o 9'Z)	55
Orange	*Citrus sinensis (L.) Osbeck var. Pera*	A01CR	Oranges, Sweet	Spain	Spain				juice fruit	orange	17.9	20.1	134.6		93.3 (isomers) 141.7 (9Z or 9'Z)	55
Orange	*Citrus sinensis (L.) Osbeck var. Powell*	A01CR	Oranges, Sweet	Spain	Spain				juice fruit	dark orange	16.1	22.1	130.6		63.6 (isomers) 148.1 (9Z or 9'Z)	55

Table S6.3.1 Oranges and similar (A01CP) (µg/100g) (continuation)

Food name	Scientific name	FoodEx2_ TermCode	FoodEx2_ TermName	Origin (country)	Purchase (country)	Water (%)	Process	Saponification	Part analysed	Colour	α-carotene	β-carotene	β-cryptoxanthin	ζ-carotene	Antheraxanthin	Ref.
Orange	*Citrus sinensis (L.) Osbeck var. Rohde late*	A01CR	Oranges, Sweet	Spain	Spain				juice fruit	dark orange	14.8	53.0	628.3		52.2 102.7 (9Z or 9'Z)	55
Orange	*Citrus sinensis (L.) Osbeck var. Rohde summer*	A01CR	Oranges, Sweet	Spain	Spain				juice fruit	dark orange	16.5	42.4	221.4		112.1 (isomers) 213.7 (9Z or 9'Z)	55
Orange	*Citrus sinensis (L.) Osbeck var. Salustiana*	A01CR	Oranges, Sweet	Spain	Spain				juice fruit	orange	16.0	18.1	146.4		97.4 (isomers) 96.5 (9Z or 9'Z)	55
Orange	*Citrus sinensis (L.) Osbeck var. Sanguinelli*	A01CR	Oranges, Sweet	Spain	Spain				juice fruit	orange	16.5	12.5	163.7		77.7 (isomers) 99.1 (9Z or 9'Z)	55
Orange	*Citrus sinensis (L.) Osbeck var. Shamoutti*	A01CR	Oranges, Sweet	Spain	Spain				juice fruit	orange	15.6	14.4	121.5		78.0 (isomers) 88.0 (9Z or 9'Z)	55
Orange	*Citrus sinensis L.*	A0DZB	Oranges	Italy	Italy						nd	171–476	74–141			25
Orange	*Citrus sinensis L.*	A0DZB	Oranges	Germany	Germany	85.2			edible part		6	13	50	20	80	53
Orange	*Citrus sinensis L.*	A0DZB	Oranges	Spain	Spain				edible part	orange	13	48	448			24
Orange	*Citrus sinensis L.*	A0DZB	Oranges	Spain	Spain				edible part		13	48	448			24
Orange	*Citrus sinensis L.*	A0DZB	Oranges	Spain	Spain	89			without skin	orange	13±5	48±12	448±27			24
Orange	*Citrus sinensis L., Lane Late var. navel*	A0DZB	Oranges	Spain	Spain				edible part	orange			85			56
Orange	*Citrus sinensis, L. Lane Late var. navel*	A0DZB	Oranges	Spain	Spain				fruit	orange	16.1	24.2	84.7			56

Table S6.3.2 Oranges and similar (A01CP) (µg/100g) (continuation)

Food name	Scientific name	FoodEx2_ TermCode	FoodEx2_ TermName	Origin (country)	Purchase (country)	Water (%)	Process	Saponification	Part analysed	Colour	Auroxanthin	E(v. trans)-α-carotene	E(v. trans)-β-carotene	E(v. trans)-β-cryptoxanthin	E(v. trans)-lutein	Ref.
Orange	*C. sinensis L. Cara cara cv. Bonanza*	A01CR	Oranges, Sweet	China	United Kingdom			yes	peel		203					52
Orange	*C. sinensis L. Osbeck cv. Bonanza*	A01CR	Oranges, Sweet	China	United Kingdom			yes	peel		4770					52
Orange	*C. sinensis L. Osbeck cv. Bonanza*	A01CR	Oranges, Sweet	China	United Kingdom			yes	juice vesicle		4743					52
Orange	*C. sinensis L. Osbeck cv. Bonanza*	A01CR	Oranges, Sweet	China	United Kingdom			yes	juice vesicle		115					52
Orange	*Citrus sinensis L.*	A0DZB	Oranges	Netherlands	Netherlands				edible part	orange			61±10	640±190	78.3±8.0	57
Orange	*Citrus sinensis L.*	A0DZB	Oranges	Netherlands	Netherlands				edible part	orange			33.5±6.5	544±98	27.9±1.5	57
Orange	*Citrus sinensis L.*	A0DZB	Oranges	Netherlands	Netherlands				edible part	orange			43±15	663±81	56±13	57
Orange	*Citrus sinensis L., Lane Late var. navel*	A0DZB	Oranges	Spain	Spain				edible part	orange		16	24			56
Orange	*Citrus sinensis, L. Lane Late var. navel*	A0DZB	Oranges	Spain	Spain				fruit	orange		16.1	24.2			56

Table S6.3.3 Oranges and similar (A01CP) (µg/100g) (continuation)

Food name	Scientific name	FoodEx2_ TermCode	FoodEx2_ TermName	Origin (country)	Purchase (country)	Water (%)	Process	Saponification	Part analysed	Colour	E(v. trans)-lycopene	E(v. trans)-zeaxanthin	Lutein	Luteoxanthin	Lycopene	Ref.
Orange	*C. sinensis L. Cara cara cv. Bonanza*	A01CR	Oranges, Sweet	China	United Kingdom			yes	peel					469		52
Orange	*C. sinensis L. Osbeck cv. Bonanza*	A01CR	Oranges, Sweet	China	United Kingdom			yes	juice vesicle					273	2374	52

Table S6.3.3 Oranges and similar (A01CP) (µg/100g) (continuation)

Food name	Scientific name	FoodEx2_ TermCode	FoodEx2_ TermName	Origin (country)	Purchase (country)	Water (%)	Process	Saponification	Part analysed	Colour	E(v. trans)- lycopene	E(v. trans)- zeaxanthin	Lutein	Luteoxanthin	Lycopene	Ref.
Orange	*Citrus sinensis L. Osbeck var. Barnfield*	A01CR	Oranges, Sweet	Spain	Spain				juice fruit	dark orange			68.1	53.5 (Z isomer)	nd	55
Orange	*Citrus sinensis L. Osbeck var. Lane late*	A01CR	Oranges, Sweet	Spain	Spain				juice fruit	dark orange			61.2	30.5 (Z isomer)	nd	55
Orange	*Citrus sinensis L. Osbeck var. Nave late*	A01CR	Oranges, Sweet	Spain	Spain				juice fruit	dark orange			42.1	41.0 (Z isomer)	nd	55
Orange	*Citrus sinensis L. Osbeck cv. Pera*	A01CR	Oranges, Sweet	France	France				juice fruit				240			43
Orange	*Citrus sinensis L. Osbeck cv. Pera*	A01CR	Oranges, Sweet	Brazil					juice fruit				134			43
Orange	*Citrus sinensis L. Osbeck cv. Sanguinelli*	A01CR	Oranges, Sweet	France	France				juice fruit				230			43
Orange	*Citrus sinensis L. Osbeck cv. Sanguinelli*	A01CR	Oranges, Sweet	France	France				juice fruit				175			43
Orange	*Citrus sinensis L. Osbeck cv. Valencia*	A01CR	Oranges, Sweet	France	France				juice fruit				190			43
Orange	*Citrus sinensis L. Osbeck cv. Valencia*	A01CR	Oranges, Sweet	France	France				juice fruit				130			43
Orange	*Citrus sinensis L. Osbeck cv. Valencia*	A01CR	Oranges, Sweet	Costa Rica					juice fruit				134			43

Table S6.3.3 Oranges and similar (A01CP) (µg/100g) (continuation)

Food name	Scientific name	FoodEx2_TermCode	FoodEx2_TermName	Origin (country)	Purchase (country)	Water (%)	Process	Saponification	Part analysed	Colour	E(v. trans)-lycopene	E(v. trans)-zeaxanthin	Lutein	Luteoxanthin	Lycopene	Ref.
Orange	*Citrus sinensis L. Osbeck cv. Valencia*	A01CR	Oranges, Sweet	French Polynesia					juice fruit				62			43
Orange	*Citrus sinensis L. Osbeck cv. Valencia*	A01CR	Oranges, Sweet	Cuba					juice fruit				25			43
Orange	*Citrus sinensis L. Osbeck var. AmberSweet*	A01CR	Oranges, Sweet	Spain	Spain				juice fruit	orange			23.1	17.2 (Z isomer)	nd	51
Orange	*Citrus sinensis L. Osbeck var. Barberina*	A01CR	Oranges, Sweet	Spain	Spain				juice fruit	orange			69.9	43.3 (Z isomer)	nd	55
Orange	*Citrus sinensis L. Osbeck var. Cadenera*	A01CR	Oranges, Sweet	Spain	Spain				juice fruit	orange			30.1	20.6 (Z isomer)	nd	55
Orange	*Citrus sinensis L. Osbeck var. Cara Cara*	A01CR	Oranges, Sweet	Spain	Spain				juice fruit	dark orange			31.3	23.0 (Z isomer)±0, 150	nd	55
Orange	*Citrus sinensis L. Osbeck var. Chislett*	A01CR	Oranges, Sweet	Spain	Spain				juice fruit	dark orange			46.5	75.5 (Z isomer)	nd	55
Orange	*Citrus sinensis L. Osbeck var. Delta Valencia*	A01CR	Oranges, Sweet	Spain	Spain				juice fruit	orange			102.4	137.4 (Z isomer)	nd	55
Orange	*Citrus sinensis L. Osbeck var. Fisher*	A01CR	Oranges, Sweet	Spain	Spain				juice fruit	dark orange			39.2	25.1 (Z isomer)	nd	55
Orange	*Citrus sinensis L. Osbeck var. Foyos*	A01CR	Oranges, Sweet	Spain	Spain				juice fruit	dark orange			51.4	32.9 (Z isomer)	nd	55

Table S6.3.3 Oranges and similar (A01CP) (µg/100g) (continuation)

Food name	Scientific name	FoodEx2_ TermCode	FoodEx2_ TermName	Origin (country)	Purchase (country)	Water (%)	Process	Saponification	Part analysed	Colour	E(v. trans)-lycopene	E(v. trans)-zeaxanthin	Lutein	Luteoxanthin	Lycopene	Ref.
Orange	*Citrus sinensis L. Osbeck var. Fukumoto*	A01CR	Oranges, Sweet	Spain	Spain				juice fruit	dark orange			41.5	22.2 (Z isomer)	nd	55
Orange	*Citrus sinensis L. Osbeck var. Hamlin*	A01CR	Oranges, Sweet	Spain	Spain				juice fruit	orange			56.0	56.1 (Z isomer)	nd	55
Orange	*Citrus sinensis L. Osbeck var. Midknight Valencia*	A01CR	Oranges, Sweet	Spain	Spain				juice fruit	orange			84.2	62.1 (Z isomer)	nd	55
Orange	*Citrus sinensis L. Osbeck var. Navelina*	A01CR	Oranges, Sweet	Spain	Spain				juice fruit	dark orange			62.3	25.8 (Z isomer)	nd	55
Orange	*Citrus sinensis L. Osbeck var. Pera*	A01CR	Oranges, Sweet	Spain	Spain				juice fruit	orange			73.1	45.2 (Z isomer)	nd	55
Orange	*Citrus sinensis L. Osbeck var. Powell*	A01CR	Oranges, Sweet	Spain	Spain				juice fruit	dark orange			54.0	51.5 (Z isomer)	nd	55
Orange	*Citrus sinensis L. Osbeck var. Rohde late*	A01CR	Oranges, Sweet	Spain	Spain				juice fruit	dark orange			27.1	32.7 (Z isomer)	nd	55
Orange	*Citrus sinensis L. Osbeck var. Rohde summer*	A01CR	Oranges, Sweet	Spain	Spain				juice fruit	dark orange			61.8	74.9 (Z isomer)	nd	55
Orange	*Citrus sinensis L. Osbeck var. Salustiana*	A01CR	Oranges, Sweet	Spain	Spain				juice fruit	orange			73.4	50.4 (Z isomer)	nd	55
Orange	*Citrus sinensis L. Osbeck var. Sanguinelli*	A01CR	Oranges, Sweet	Spain	Spain				juice fruit	orange			62.2	53.5 (Z isomer)	nd	55

Table S6.3.3 Oranges and similar (A01CP) (µg/100g) (continuation)

Food name	Scientific name	FoodEx2_ TermCode	FoodEx2_ TermName	Origin (country)	Purchase (country)	Water (%)	Process	Saponification	Part analysed	Colour	E(v. trans)-lycopene	E(v. trans)-zeaxanthin	Lutein	Luteoxanthin	Lycopene	Ref.
Orange	*Citrus sinensis L. Osbeck var. Shamoutti*	A01CR	Oranges, Sweet	Spain	Spain				juice fruit	orange			43.2	55.0 (Z isomer)	nd	55
Orange	*Citrus sinensis L.*	A0DZB	Oranges	Italy	Italy										nd	25
Orange	*Citrus sinensis L.*	A0DZB	Oranges	Germany	Germany	85.2			edible part				20			53
Orange	*Citrus sinensis L.*	A0DZB	Oranges	Netherlands	Netherlands				edible part	orange		65±16				57
Orange	*Citrus sinensis L.*	A0DZB	Oranges	Netherlands	Netherlands				edible part	orange		61.9±8.6				57
Orange	*Citrus sinensis L.*	A0DZB	Oranges	Netherlands	Netherlands				edible part	orange		98±27				57
Orange	*Citrus sinensis L.*	A0DZB	Oranges	Spain	Spain				edible part	orange			68			24
Orange	*Citrus sinensis L.*	A0DZB	Oranges	Spain	Spain				edible part				68			24
Orange	*Citrus sinensis L.*	A0DZB	Oranges	Spain	Spain				pulp	orange						22
Orange	*Citrus sinensis L.*	A0DZB	Oranges	Spain	Spain	89			without skin	orange			68±20			24

Table S6.3.4 Oranges and similar (A01CP) (µg/100g) (continuation)

Food name	Scientific name	FoodEx2_ TermCode	FoodEx2_ TermName	Origin (country)	Purchase (country)	Water (%)	Process	Saponification	Part analysed	Colour	Mutatoxanthin	Neochrome	Neoxanthin	Phytoene	Phytofluene	Ref.
Orange	*C. sinensis L. Cara cara cv. Bonanza*	A01CR	Oranges, Sweet	China	United Kingdom			yes	peel					85	22	52
Orange	*C. sinensis L. Osbeck cv. Bonanza*	A01CR	Oranges, Sweet	China	United Kingdom			yes	peel					1445	1095	52
Orange	*C. sinensis L. Osbeck cv. Bonanza*	A01CR	Oranges, Sweet	China	United Kingdom			yes	juice vesicle					7917	983	52
Orange	*C. sinensis L. Osbeck cv. Bonanza*	A01CR	Oranges, Sweet	China	United Kingdom			yes	juice vesicle					5710	1745	52
Orange	*Citrus sinensis L. Osbeck var. Barnfield*	A01CR	Oranges, Sweet	Spain	Spain				juice fruit	dark orange	53.0 (epimer A) 100.7 (epimer B)			130.1	41.8	55
Orange	*Citrus sinensis L. Osbeck var. Lane late*	A01CR	Oranges, Sweet	Spain	Spain				juice fruit	dark orange	54.7 (epimer A) 108.6 (epimer B)			113.6	37.1	55
Orange	*Citrus sinensis L. Osbeck var. Nave late*	A01CR	Oranges, Sweet	Spain	Spain				juice fruit	dark orange	48.1 (epimer A) 90.9 (epimer B)			120.6	38.0	55
Orange	*Citrus sinensis L. Osbeck cv. Pera*	A01CR	Oranges, Sweet	France	France				juice fruit					50	75	43
Orange	*Citrus sinensis L. Osbeck cv. Pera*	A01CR	Oranges, Sweet	Brazil					juice fruit					10	6	43
Orange	*Citrus sinensis L. Osbeck cv. Sanguinelli*	A01CR	Oranges, Sweet	France	France				juice fruit					100	110	43
Orange	*Citrus sinensis L. Osbeck cv. Sanguinelli*	A01CR	Oranges, Sweet	France	France				juice fruit					12	15	43

Table S6.3.4 Oranges and similar (A01CP) (μg/100g) (continuation)

Food name	Scientific name	FoodEx2_ TermCode	FoodEx2_ TermName	Origin (country)	Purchase (country)	Water (%)	Process	Saponification	Part analysed	Colour	Mutatoxanthin	Neochrome	Neoxanthin	Phytoene	Phytofluene	Ref.
Orange	*Citrus sinensis L. Osbeck cv. Valencia*	A01CR	Oranges, Sweet	France	France				juice fruit					36	55	43
Orange	*Citrus sinensis L. Osbeck cv. Valencia*	A01CR	Oranges, Sweet	France	France				juice fruit					10	10	43
Orange	*Citrus sinensis L. Osbeck cv. Valencia*	A01CR	Oranges, Sweet	Costa Rica					juice fruit					5	1	43
Orange	*Citrus sinensis L. Osbeck cv. Valencia*	A01CR	Oranges, Sweet	French Polynesia					juice fruit					0	0	43
Orange	*Citrus sinensis L. Osbeck cv. Valencia*	A01CR	Oranges, Sweet	Cuba					juice fruit					4	0	43
Orange	*Citrus sinensis L. Osbeck var. AmberSweet*	A01CR	Oranges, Sweet	Spain	Spain				juice fruit	orange	71.1 (epimer A) 30.1 (epimer B)			101.1	41.7	55
Orange	*Citrus sinensis L. Osbeck var. Barberina*	A01CR	Oranges, Sweet	Spain	Spain				juice fruit	orange	66.4 (epimer A) 110.8 (epimer B)			100.1	27.1	55
Orange	*Citrus sinensis L. Osbeck var. Cadenera*	A01CR	Oranges, Sweet	Spain	Spain				juice fruit	orange	27.3 (epimer A) 52.3 (epimer B)			52.0	12.9	55
Orange	*Citrus sinensis L. Osbeck var. Cara Cara*	A01CR	Oranges, Sweet	Spain	Spain				juice fruit	dark orange	28.9 (epimer A) 58.3 (epimer B)			1180.3	272.6± 0.198	55
Orange	*Citrus sinensis L. Osbeck var. Chislett*	A01CR	Oranges, Sweet	Spain	Spain				juice fruit	dark orange	53.5 (epimer A) 102.4 (epimer B)			123.5	43.4	55

Table S6.3.4 Oranges and similar (A01CP) (µg/100g) (continuation)

Food name	Scientific name	FoodEx2_ TermCode	FoodEx2_ TermName	Origin (country)	Purchase (country)	Water (%)	Process	Saponification	Part analysed	Colour	Mutatoxanthin	Neochrome	Neoxanthin	Phytoene	Phytofluene	Ref.
Orange	*Citrus sinensis L. Osbeck var. Delta Valencia*	A01CR	Oranges, Sweet	Spain	Spain				juice fruit	orange	77.6 (epimer A) 125.2 (epimer B)			112.9	32.1	55
Orange	*Citrus sinensis L. Osbeck var. Fisher*	A01CR	Oranges, Sweet	Spain	Spain				juice fruit	dark orange	34.3 (epimer A) 69.0 (epimer B)			61.1	15.4	55
Orange	*Citrus sinensis L. Osbeck var. Foyos*	A01CR	Oranges, Sweet	Spain	Spain				juice fruit	dark orange	42.7 (epimer A) 81.4 (epimer B)			75.2	21.5	55
Orange	*Citrus sinensis L. Osbeck var. Fukumoto*	A01CR	Oranges, Sweet	Spain	Spain				juice fruit	dark orange	35.4 (epimer A) 52.0 (epimer B)			46.9	13.4	55
Orange	*Citrus sinensis L. Osbeck var. Hamlin*	A01CR	Oranges, Sweet	Spain	Spain				juice fruit	orange	48.5 (epimer A) 105.1 (epimer B)			114.9	29.2	55
Orange	*Citrus sinensis L. Osbeck var. Midknight Valencia*	A01CR	Oranges, Sweet	Spain	Spain				juice fruit	orange	65.7 (epimer A) 129.4 (epimer B)			106.3	26.1	55
Orange	*Citrus sinensis L. Osbeck var. Navelina*	A01CR	Oranges, Sweet	Spain	Spain				juice fruit	dark orange	50.2 (epimer A) 90.6 (epimer B)			104.9	30.1	55
Orange	*Citrus sinensis L. Osbeck var. Pera*	A01CR	Oranges, Sweet	Spain	Spain				juice fruit	orange	55.2 (epimer A) 115.8 (epimer B)			84.4	16.5	55
Orange	*Citrus sinensis L. Osbeck var. Powell*	A01CR	Oranges, Sweet	Spain	Spain				juice fruit	dark orange	51.5 (epimer A) 93.2 (epimer B)			115.0	37.1	55

Table S6.3.4 Oranges and similar (A01CP) (µg/100g) (continuation)

Food name	Scientific name	FoodEx2_ TermCode	FoodEx2_ TermName	Origin (country)	Purchase (country)	Water (%)	Process	Saponification	Part analysed	Colour	Mutatoxanthin	Neochrome	Neoxanthin	Phytoene	Phytofluene	Ref.
Orange	*Citrus sinensis L. Osbeck var. Rohde late*	A01CR	Oranges, Sweet	Spain	Spain				juice fruit	dark orange	53.9 (epimer A) 36.2 (epimer B)			94.1	52.4	55
Orange	*Citrus sinensis L. Osbeck var. Rohde summer*	A01CR	Oranges, Sweet	Spain	Spain				juice fruit	dark orange	68.8 (epimer A) 130.9 (epimer B)			118.7	56.0	55
Orange	*Citrus sinensis L. Osbeck var. Salustiana*	A01CR	Oranges, Sweet	Spain	Spain				juice fruit	orange	58.2 (epimer A) 128.0 (epimer B)			89.4	19.5	55
Orange	*Citrus sinensis L. Osbeck var. Sanguinelli*	A01CR	Oranges, Sweet	Spain	Spain				juice fruit	orange	50.1 (epimer A) 74.6 (epimer B)			125.0	17.8	55
Orange	*Citrus sinensis L. Osbeck var. Shamoutti*	A01CR	Oranges, Sweet	Spain	Spain				juice fruit	orange	52.1 (epimer A) 98.8 (epimer B)			70.1	17.0	55
Orange	*Citrus sinensis L.*	A0DZB	Oranges	Germany	Germany	85.2			edible part					80	40	53
Orange	*Citrus sinensis L.*	A0DZB	Oranges	Spain	Spain				pulp	orange				230 (50-560)	110 (40-180)	22
Orange	*Citrus sinensis L.*	A0DZB	Oranges	Spain	Spain	89			without skin	orange				1065±74		24

Table S6.3.5 Oranges and similar (A01CP) (µg/100g) (continuation)

Food name	Scientific name	FoodEx2_ TermCode	FoodEx2_ TermName	Origin (country)	Purchase (country)	Water (%)	Process	Saponification	Part analysed	Colour	Violaxanthin	Z(v. cis)-lycopene	Z(v. cis)-β-carotene	Z(v. cis)-β-cryptoxanthin	Zeaxanthin	Zeinoxanthin	Ref.
Orange	*C. sinensis L. Cara cara cv. Bonanza*	A01CR	Oranges, Sweet	China	United Kingdom			yes	peel		116				112		52
Orange	*C. sinensis L. Osbeck cv. Bonanza*	A01CR	Oranges, Sweet	China	United Kingdom			yes	peel						20		52
Orange	*C. sinensis L. Osbeck cv. Bonanza*	A01CR	Oranges, Sweet	China	United Kingdom			yes	juice vesicle						147		52
Orange	*C. sinensis L. Osbeck cv. Bonanza*	A01CR	Oranges, Sweet	China	United Kingdom			yes	juice vesicle		86				141		52
Orange	*Citrus sinensis L. Osbeck var. Barnfield*	A01CR	Oranges, Sweet	Spain	Spain				juice fruit	dark orange	282.6 (all-E and Z isomers)				55.8	30.3	55
Orange	*Citrus sinensis L. Osbeck var. Lane late*	A01CR	Oranges, Sweet	Spain	Spain				juice fruit	dark orange	252.0 (all-E and Z isomers)				50.0	30.2	55
Orange	*Citrus sinensis L. Osbeck var. Nave late*	A01CR	Oranges, Sweet	Spain	Spain				juice fruit	dark orange	210.6 (all-E and Z isomers)				40.7		55
Orange	*Citrus sinensis L. Osbeck cv. Pera*	A01CR	Oranges, Sweet	France	France				juice fruit		505 (cis)				310		43
Orange	*Citrus sinensis L. Osbeck cv. Pera*	A01CR	Oranges, Sweet	Brazil					juice fruit		300 (cis)				109		43
Orange	*Citrus sinensis L. Osbeck cv. Sanguinelli*	A01CR	Oranges, Sweet	France	France				juice fruit		720 (cis)				171		43
Orange	*Citrus sinensis L. Osbeck cv. Sanguinelli*	A01CR	Oranges, Sweet	France	France				juice fruit		130 (cis)				89		43
Orange	*Citrus sinensis L. Osbeck cv. Valencia*	A01CR	Oranges, Sweet	France	France				juice fruit		539 (cis)				173		43
Orange	*Citrus sinensis L. Osbeck cv. Valencia*	A01CR	Oranges, Sweet	France	France				juice fruit		460 (cis)				117		43

Table S6.3.5 Oranges and similar (A01CP) (µg/100g) (continuation)

Food name	Scientific name	FoodEx2_ TermCode	FoodEx2_ TermName	Origin (country)	Purchase (country)	Water (%)	Process	Saponification	Part analysed	Colour	Violaxanthin	Z(v. cis)-lycopene	Z(v. cis)-β-carotene	Z(v. cis)-β-cryptoxanthin	Zeaxanthin	Zeinoxanthin	Ref.
Orange	*Citrus sinensis L. Osbeck cv. Valencia*	A01CR	Oranges, Sweet	Costa Rica					juice fruit		204 (cis)				125		43
Orange	*Citrus sinensis L. Osbeck cv. Valencia*	A01CR	Oranges, Sweet	French Polynesia					juice fruit		146 (cis)				41		43
Orange	*Citrus sinensis L. Osbeck cv. Valencia*	A01CR	Oranges, Sweet	Cuba					juice fruit		56 (cis)				22		43
Orange	*Citrus sinensis L. Osbeck var. AmberSwee t*	A01CR	Oranges, Sweet	Spain	Spain				juice fruit	orange	73.3 (all-E + Z isomers)				28.3	25.2	55
Orange	*Citrus sinensis L. Osbeck var. Barberina*	A01CR	Oranges, Sweet	Spain	Spain				juice fruit	orange	233.0 (all-E) + (Z) isomers)				52.1	38.0	55
Orange	*Citrus sinensis L. Osbeck var. Cadenera*	A01CR	Oranges, Sweet	Spain	Spain				juice fruit	orange	76.1 (all-(E) + (Z) isomers				29.3	18.1	55
Orange	*Citrus sinensis L. Osbeck var. Cara Cara*	A01CR	Oranges, Sweet	Spain	Spain				juice fruit	dark orange	82.8 (all-E + Z isomers)				34.0	18.0	55
Orange	*Citrus sinensis L. Osbeck var. Chislett*	A01CR	Oranges, Sweet	Spain	Spain				juice fruit	dark orange	204.6 (all-E + Z isomers)				50.0	24.9	55
Orange	*Citrus sinensis L. Osbeck var. Delta Valencia*	A01CR	Oranges, Sweet	Spain	Spain				juice fruit	orange	302.5 (all-(E) + (Z) isomers)				61.0	48.6	55

Table S6.3.5 Oranges and similar (A01CP) (µg/100g) (continuation)

Food name	Scientific name	FoodEx2_ TermCode	FoodEx2_ TermName	Origin (country)	Purchase (country)	Water (%)	Process	Saponification	Part analysed	Colour	Violaxanthin	Z(v. cis)-lycopene	Z(v. cis)-β-carotene	Z(v. cis)-β-cryptoxanthin	Zeaxanthin	Zeinoxanthin	Ref.
Orange	*Citrus sinensis L. Osbeck var. Fisher*	A01CR	Oranges, Sweet	Spain	Spain				juice fruit	dark orange	182.1 (all-(E) + (Z) isomers)				35.2	22.6	55
Orange	*Citrus sinensis L. Osbeck var. Foyos*	A01CR	Oranges, Sweet	Spain	Spain				juice fruit	dark orange	220.0 (all-(E) + (Z) isomers)				41.4	25.9	55
Orange	*Citrus sinensis L. Osbeck var. Fukumoto*	A01CR	Oranges, Sweet	Spain	Spain				juice fruit	dark orange	108.5 (all-E + Z isomers)				35.1	19.2	55
Orange	*Citrus sinensis L. Osbeck var. Hamlin*	A01CR	Oranges, Sweet	Spain	Spain				juice fruit	orange	238.2 (all-E + Z isomers)				48.4	33.4	55
Orange	*Citrus sinensis L. Osbeck var. Midknight Valencia*	A01CR	Oranges, Sweet	Spain	Spain				juice fruit	orange	277.1 (all-(E) + (Z) isomers)				72.0	38.1	55
Orange	*Citrus sinensis L. Osbeck var. Navelina*	A01CR	Oranges, Sweet	Spain	Spain				juice fruit	dark orange	170.8 (all-E + Z isomers)				59.3	28.0	55
Orange	*Citrus sinensis L. Osbeck var. Pera*	A01CR	Oranges, Sweet	Spain	Spain				juice fruit	orange	186.3 (all-E + Z isomers)				61.3	36.6	55
Orange	*Citrus sinensis L. Osbeck var. Powell*	A01CR	Oranges, Sweet	Spain	Spain				juice fruit	dark orange	208.0 (all E + Z isomers)				50.0	27.8	55
Orange	*Citrus sinensis L. Osbeck var. Rohde late*	A01CR	Oranges, Sweet	Spain	Spain				juice fruit	dark orange	74.6 (all E + Z)				30.3	32.7	55

Table S6.3.5 Oranges and similar (A01CP) (µg/100g) (continuation)

Food name	Scientific name	FoodEx2_ TermCode	FoodEx2_ TermName	Origin (country)	Purchase (country)	Water (%)	Process	Saponification	Part analysed	Colour	Violaxanthin	Z(v. cis)-lycopene	Z(v. cis)-β-carotene	Z(v. cis)-β-cryptoxanthin	Zeaxanthin	Zeinoxanthin	Ref.
Orange	*Citrus sinensis L. Osbeck var. Rohde summer*	A01CR	Oranges, Sweet	Spain	Spain				juice fruit	dark orange	300.8 (all E + Z isomers)				67.8	30.7	55
Orange	*Citrus sinensis L. Osbeck var. Salustiana*	A01CR	Oranges, Sweet	Spain	Spain				juice fruit	orange	270.5 (all-E + Z isomers)				73.7	32.4	55
Orange	*Citrus sinensis L. Osbeck var. Sanguinelli*	A01CR	Oranges, Sweet	Spain	Spain				juice fruit	orange	156.8 (all-E + Z isomers)±0,155				53.6	39.0	55
Orange	*Citrus sinensis L. Osbeck var. Shamoutti*	A01CR	Oranges, Sweet	Spain	Spain				juice fruit	orange	123.8 (all-E + Z isomers)				39.3	29.4	55
Orange	*Citrus sinensis L.*	A0DZB	Oranges	Germany	Germany	85.2									8		53
Orange	*Citrus sinensis L.*	A0DZB	Oranges	Spain	Spain					Orange					66		24
Orange	*Citrus sinensis L.*	A0DZB	Oranges	Spain	Spain										66		24
Orange	*Citrus sinensis L.*	A0DZB	Oranges	Spain	Spain	89			without skin	Orange					66±19		24

Table S6.4.1 Lemons and similar (A01BX) (µg/100g)

Food name	Scientific name	FoodEx2_TermCode	FoodEx2_TermName	Origin (country)	Purchase (country)	Water (%)	Process	Saponification	Part analysed	Colour	α-carotene	β-carotene	β-cryptoxanthin	ζ-carotene	Antheraxanthin	Ref.
Lemon	*Citrus limonum L. Osbeck.*	A01BY	Lemons	Germany	Germany	88.6					1	2	20	3		53
Lemon	*Citrus limonum L. Osbeck.*	A01BY	Lemons	Spain	Spain					yellow	tr.		14			24
Lemon	*Citrus limonum L. Osbeck.*	A01BY	Lemons	Spain	Spain								14			24
Lemon	*Citrus limonum L. Osbeck.*	A01BY#F20.A07QF	Lemons, PART-CONSUMED-ANALYSED = W/o peel	Spain	Spain	89			without skin	green	tr.		14±2			24

Table S6.4.2 Lemons and similar (A01BX) (µg/100g) (continuation)

Food name	Scientific name	FoodEx2_TermCode	FoodEx2_TermName	Origin (country)	Purchase (country)	Water (%)	Process	Saponification	Part analysed	Colour	E(v. trans)-lycopene	E(v. trans)-zeaxanthin	Lutein	Luteoxanthin	Lycopene	Ref.
Lemon	*Citrus limonum L. Osbeck.*	A01BY	Lemons	Spain	Spain					yellow			2			24
Lemon	*Citrus limonum L. Osbeck.*	A01BY	Lemons	Spain	Spain								2			24
Lemon	*Citrus limonum L. Osbeck.*	A01BY#F20.A07QF	Lemons, PART-CONSUMED-ANALYSED = W/o peel	Spain	Spain	89			without skin	green			2±0.3			24

Table S6.4.3 Lemons and similar (A01BX) (µg/100g) (continuation)

Food name	Scientific name	FoodEx2_Term Code	FoodEx2_Term Name	Origin (country)	Purchase (country)	Water (%)	Process	Saponification	Part analysed	Colour	Mutatoxanthin	Neochrome	Neoxanthin	Phytoene	Phytofluene	Ref.
Lemon	*Citrus limonum L. Osbeck.*	A01BY	Lemons	Germany	Germany	88.6								10	2	53
Lemon	*Citrus limonum L. Osbeck.*	A01BY	Lemons	Spain	Spain				Pulp	yellow				10		22

Table S6.4.4 Lemons and similar (A01BX) (µg/100g) (continuation)

Food name	Scientific name	FoodEx2_TermCode	FoodEx2_TermName	Origin (country)	Purchase (country)	Water (%)	Process	Saponification	Part analysed	Colour	Violaxanthin	Z(v. cis)-lycopene	Z(v. cis)-β-carotene	Z(v. cis)-β-cryptoxanthin	Zeaxanthin	Zeinoxanthin	Ref.
Lemon	*Citrus limonum L. Osbeck.*	A01BY	Lemons	Germany	Germany	88.6									1		54
Lemon	*Citrus limonum L. Osbeck.*	A01BY	Lemons	Spain	Spain					yellow					1		24
Lemon	*Citrus limonum L. Osbeck.*	A01BY	Lemons	Spain	Spain										1		24
Lemon	*Citrus limonum L. Osbeck.*	A01BY#F20. A07QF	Lemons, PART-CONSUMED-ANALYSED = W/o peel	Spain	Spain	89			without skin	green					1±0.3		24

Table S6.5.1 Mandarins and similar (A01CB) (µg/100g)

Food name	Scientific name	FoodEx2_TermCode	FoodEx2_TermName	Origin (country)	Purchase (country)	Water (%)	Process	Saponification	Part analysed	Colour	α-carotene	β-carotene	β-cryptoxanthin	ζ-carotene	Antheraxanthin	Ref.
Clementine	*Citrus clementina*	A01CE	Clementines	Germany	Germany	86.9			edible part		5	30	500±30	50	150	53
Clementine	*Citrus clementina Hort. ex Tan*	A01CE	Clementines	France	France				juice fruit			68	849			43
Clementine	*Citrus clementina Hort. ex Tan*	A01CE	Clementines	France	France				juice fruit			17	893			43
Mandarin	*Citrus cv Mediterranean*	A01CD	Mandarins	Italy	Italy				essential oil of peel fruit (free)				610±62			58
Mandarin	*Citrus cv Mediterranean*	A01CD	Mandarins	Italy	Italy				essential oil of peel fruit (laurate)				1411±140			58
Mandarin	*Citrus cv Mediterranean*	A01CD	Mandarins	Italy	Italy				essential oil of peel fruit (myristate)				1504±132			58
Mandarin	*Citrus cv Mediterranean*	A01CD	Mandarins	Italy	Italy				essential oil of peel fruit (palmitate)				715±74			58
Mandarin	*Citrus deliciosa, Ten.*	A01CJ	Mediterranean mandarins	Spain	Spain	86			without skin	orange		213±102	843±216			24
Mandarin	*Citrus deliciosa, Ten.*	A01CJ	Mediterranean mandarins	Spain	Spain	85			without skin	orange		130±10	1106±63			24
Mandarin	*Citrus reticulata*	A01CD	Mandarins	Spain	Spain					orange		213	843			24
Mandarin	*Citrus reticulata*	A01CD	Mandarins	Spain	Spain					orange		213	843			24
Mandarin	*Citrus reticulata Blanco cv. Hansen*	A01CE	Clementines	France	France				juice fruit			197	1500			43
Mandarin	*Citrus reticulata Blanco cv. Hansen*	A01CE	Clementines	France	France				juice fruit			70	162			43

Table S6.5.1 Mandarins and similar (A01CB) (µg/100g) (continuation)

Food name	Scientific name	FoodEx2_TermCode	FoodEx2_TermName	Origin (country)	Purchase (country)	Water (%)	Process	Saponification	Part analysed	Colour	α-carotene	β-carotene	β-cryptoxanthin	ζ-carotene	Antheraxanthin	Ref.
Mandarin	*Citrus reticulata Blanco cv. Hansen*	A01CE	Clementines	French Polynesia					juice fruit			76	916			43
Mandarin	*Citrus reticulata L., var. Tango*	A01CD	Mandarins	Spain	Spain					orange			1331.6			56
Mandarin	*Citrus reticulate, L. var. Tango*	A01CD	Mandarins	Spain	Spain				fruit	orange	12.4	547	1331.6			56
Tangerine	*Citrus reticulata*	A01CD	Mandarins	Indonesia					fruit			162 (109-254)	60 (n.d.-169)			59
Tangors	*Citrus nobilis*	A01CM	Tangors	Indonesia					fruit			275 (171-476)	114 (74-141)			59

Table S6.5.2 Mandarins and similar (A01CB) (µg/100g) (continuation)

Food name	Scientific name	FoodEx2_TermCode	FoodEx2_TermName	Origin (country)	Purchase (country)	Water (%)	Process	Saponification	Part analysed	Colour	Auroxanthin	E(v. trans)-α-carotene	E(v. trans)-β-carotene	E(v. trans)-β-cryptoxanthin	E(v. trans)-lutein	Ref.
Mandarin	*Citrus reticulata L., var. Tango*	A01CD	Mandarins	Spain	Spain					orange		12.4	547			56
Mandarin	*Citrus reticulate, L. var. Tango*	A01CD	Mandarins	Spain	Spain				Fruit	Orange		12,4	547			56

Table S6.5.3 Mandarins and similar (A01CB) (µg/100g) (continuation)

Food name	Scientific name	FoodEx2_ TermCode	FoodEx2_ TermName	Origin (country)	Purchase (country)	Water (%)	Process	Saponification	Part analysed	Colour	E(v. trans)-lycopene	E(v. trans)-zeaxanthin	Lutein	Luteoxanthin	Lycopene	Ref.
Clementine	*Citrus clementina*	A01CE	Clementines	Germany	Germany	86.9							60			53
Clementine	*Citrus clementina Hort. ex Tan*	A01CE	Clementines	France	France				juice fruit				61			43
Clementine	*Citrus clementina Hort. ex Tan*	A01CE	Clementines	France	France				juice fruit				80			43
Mandarin	*Citrus reticulata Blanco* **cv. Hansen**	A01CE	Clementines	France	France				juice fruit				163			43
Mandarin	*Citrus reticulata Blanco cv. Hansen*	A01CE	Clementines	France	France				juice fruit				230			43
Mandarin	*Citrus reticulata Blanco* **cv. Hansen**	A01CE	Clementines	French Polynesia					juice fruit				210			43
Tangerine	*Citrus reticulata*	A01CD	Mandarins	Indonesia					fruit						386 (222-670)	59

Table S6.5.4 Mandarins and similar (A01CB) (µg/100g) (continuation)

Food name	Scientific name	FoodEx2_Term Code	FoodEx2_Term Name	Origin (country)	Purchase (country)	Water (%)	Process	Saponification	Part analysed	Colour	Mutatoxanthin	Neochrome	Neoxanthin	Phytoene	Phytofluene	Ref.
Clementine	*Citrus clementina*	A01CE	Clementines	Germany	Germany	86.9								70	70	53
Clementine	*Citrus clementina*	A01CE	Clementines	Spain	Spain				pulp	orange				70	70	22
Clementine	*Citrus clementina Hort. ex Tan*	A01CE	Clementines	France	France				juice fruit					65	122	44
Clementine	*Citrus clementina Hort. ex Tan*	A01CE	Clementines	France	France				juice fruit					11	62	43
Mandarin	*Citrus reticulata Blanco* ***cv. Hansen***	A01CE	Clementines	France	France				juice fruit					446	500	43
Mandarin	*Citrus reticulata Blanco cv.* ***Hansen***	A01CE	Clementines	France	France				juice fruit					100	110	43
Mandarin	*Citrus reticulata Blanco* ***cv. Hansen***	A01CE	Clementines	French Polynesia					juice fruit					24	92	43

Table S6.5.5 Mandarins and similar (A01CB) (µg/100g) (continuation)

Food name	Scientific name	FoodEx2_ TermCode	FoodEx2_ TermName	Origin (country)	Purchase (country)	Water (%)	Process	Saponification	Part analysed	Colour	Violaxanthin	Z(v. cis)- lycopene	Z(v. cis)-β- carotene	Z(v. cis)-β- cryptoxanthin	Zeaxanthin	Zeinoxanthin	Ref.
Clementine	*Citrus clementina Hort. ex Tan*	A01CE	Clementines	France	France				juice fruit		206 (cis)				63		43
Clementine	*Citrus clementina Hort. ex Tan*	A01CE	Clementines	France	France				juice fruit		215 (cis)				106		43

Table S6.5.5 Mandarins and similar (A01CB) (µg/100g) (continuation)

Food name	Scientific name	FoodEx2_ TermCode	FoodEx2_ TermName	Origin (country)	Purchase (country)	Water (%)	Process	Saponification	Part analysed	Colour	Violaxanthin	Z(v. cis)- lycopene	Z(v. cis)-β- carotene	Z(v. cis)-β- cryptoxanthin	Zeaxanthin	Zeinoxanthin	Ref.
Mandarin	*Citrus reticulata Blanco* ***cv. Hansen***	A01CE	Clementines	France	France				juice fruit		467 (cis)				128		43
Mandarin	*Citrus reticulata Blanco* ***cv. Hansen***	A01CE	Clementines	France	France				juice fruit		445 (cis)				143		43
Mandarin	*Citrus reticulata Blanco* ***cv. Hansen***	A01CE	Clementines	French Polynesia					juice fruit		560 (cis)				131		43

Table S6.6.1 Apples and similar (A01DH) (µg/100g)

Food name	Scientific name	FoodEx2_ TermCode	FoodEx2_ TermName	Origin (country)	Purchase (country)	Water (%)	Process	Saponification	Part analysed	Colour	α-carotene	β-carotene	β-cryptoxanthin	ζ-carotene	Antheraxanthin	Ref.
Apple	*Malus domestca var. red Delicious*	A01DJ	Apples	Spain	Spain							16.6±0.9	12.6 (all-E)±0.4			39
Apple	*Malus domestica*	A01DJ	Apples	Indonesia								72 (n.d.-152)	106 (70-167)			59
Apple	*Malus domestica Borkh. var. bravo esmolfe*	A01DJ	Apples	Portugal	Portugal				edible part	yellow	1.3	10	0.9			32
Apple	*Malus domestica Borkh. var. golden delicious*	A01DJ	Apples	Portugal	Portugal				edible part	yellow	nd	34	nd			32
Apple	*Malus domestica Borkh. var. golden delicious*	A01DJ	Apples	Portugal	Portugal				edible part	yellow	nd	63	nd			32
Apple	*Malus domestica Borkh. var. jonagold*	A01DJ	Apples	Portugal	Portugal				edible part	yellow	nd	26	nd			32
Apple	*Malus domestica Borkh. var. reineta parda*	A01DJ	Apples	Portugal	Portugal				edible part	green	nd	17	4			32
Apple	*Malus domestica Borkh. var. royal gala*	A01DJ	Apples	Portugal	Portugal				edible part	red	nd	11	nd			32

Table S6.6.1 Apples and similar (A01DH) (µg/100g)

Food name	Scientific name	FoodEx2_ TermCode	FoodEx2_ TermName	Origin (country)	Purchase (country)	Water (%)	Process	Saponification	Part analysed	Colour	α-carotene	β-carotene	β-cryptoxanthin	ζ-carotene	Antheraxanthin	Ref.
Apple	*Malus domestica Borkh. var. starking*	A01DJ	Apples	Portugal	Portugal				edible part	red	nd	13	nd			32
Apple	*Malus domestica Borkh. var. starking*	A01DJ	Apples	Portugal	Portugal				edible part	red	nd	48	nd			32
Apple	*Malus domestica Borkh. var. starking*	A01DJ	Apples	Portugal	Portugal				edible part	red	nd	36	nd			32
Apple	*Malus domestica Elstar*	A01DJ	Apples	Germany	Germany	84.1			edible part			20	4		20	53
Apple	*Malus domestica var. Fuji*	A01DJ	Apples	Spain	Spain							22.7±1.2	13.7 (all-E)±0.7		12.1 (all-E)±0.3	40
Apple	*Malus domestica var. Golden Delicious*	A01DJ	Apples	Spain	Spain							41.7±3.5	19.4 (all-E)±0.1		11.3 (all-E)±0.3	39
Apple	*Malus domestica var. Granny Smith*	A01DJ	Apples	Spain	Spain							17.8±0.3	8.9 (all-E)±0.1			39
Apple	*Malus domestica var. Pink Lady*	A01DJ	Apples	Spain	Spain							22.4±1.5	16.3 (all-E)±0.5			39
Apple	*Malus domestica var. red Delicious*	A01DJ#F10. A0F2S	Apples, QUALITATIVE-INFO = red	USA				no	with skin		0		0			28
Apple	*Malus domestica var. Royal Gala*	A01DJ	Apples	Spain	Spain							14.6±0.9	12.7 (all-E)±0.3		9.4 (all-E)±0.1	39
Apple	*Malus pumila*	A01DJ	Apples	Germany	Germany	84.6			edible part			11	8		3	53
Apple	*Malus pumila*	A01DJ	Apples	Spain	Spain				edible part	yellow		20	8			24
Apple	*Malus pumila*	A01DJ	Apples	Spain	Spain				edible part			20	8			24

Table S6.6.2 Apples and similar (A01DH) (µg/100g) (continuation)

Food name	Scientific name	FoodEx2_ TermCode	FoodEx2_ TermName	Origin (country)	Purchase (country)	Water (%)	Process	Saponification	Part analysed	Colour	Auroxanthin	E(v. trans)-α-carotene	E(v. trans)-β-carotene	E(v. trans)-β-cryptoxanthin	E(v. trans)-lutein	Ref.
Apple	*Malus domestica var. red Delicious*	A01DJ#F10. A0F2S	Apples, QUALITATIVE-INFO = red	USA				no	with skin				18		15	28

Table S6.6.3 Apples and similar (A01DH) (µg/100g) (continuation)

Food name	Scientific name	FoodEx2_ TermCode	FoodEx2_ TermName	Origin (country)	Purchase (country)	Water (%)	Process	Saponification	Part analysed	Colour	E(v. trans)-lycopene	E(v. trans)-zeaxanthin	Lutein	Luteoxanthin	Lycopene	Ref.
Apple	*Malus domestica*	A01DJ	Apples	Indonesia					fruit						209 (60-308)	59
Apple	*Malus domestica Borkh. Var. bravo esmolfe*	A01DJ	Apples	Portugal	Portugal				edible part	yellow			17		nd	32
Apple	*Malus domestica Borkh. var. golden delicious*	A01DJ	Apples	Portugal	Portugal				edible part	yellow			3.2		nd	32
Apple	*Malus domestica Borkh. var. golden delicious*	A01DJ	Apples	Portugal	Portugal				edible part	yellow			1.6		nd	32
Apple	*Malus domestica Borkh. var. jonagold*	A01DJ	Apples	Portugal	Portugal				edible part	yellow			3.5		nd	32

Table S6.6.3 Apples and similar (A01DH) (µg/100g) (continuation)

Food name	Scientific name	FoodEx2_ TermCode	FoodEx2_ TermName	Origin (country)	Purchase (country)	Water (%)	Process	Saponification	Part analysed	Colour	E(v. trans)- lycopene	E(v. trans)- zeaxanthin	Lutein	Luteoxanthin	Lycopene	Ref.
Apple	*Malus domestica Borkh. var. reineta parda*	A01DJ	Apples	Portugal	Portugal				edible part	green			10		nd	32
Apple	*Malus domestica Borkh. var. royal gala*	A01DJ	Apples	Portugal	Portugal				edible part	red			2.2		nd	32
Apple	*Malus domestica Borkh. var. starking*	A01DJ	Apples	Portugal	Portugal				edible part	red			9.7		nd	32
Apple	*Malus domestica Borkh. var. starking*	A01DJ	Apples	Portugal	Portugal				edible part	red			16		nd	32
Apple	*Malus domestica Borkh. var. starking*	A01DJ	Apples	Portugal	Portugal				eedible part	red			10		nd	32
Apple	*Malus domestica Elstar*	A01DJ	Apples	Germany	Germany	84.1			edible part				20			53
Apple	*Malus domestica var. red Delicious*	A01DJ#F10. A0F2S	Apples, QUALITATI VE-INFO = red	USA				no	with skin			0				28
Apple	*Malus domestica var. Fuji*	A01DJ	Apples	Spain	Spain				fruit				19.0 (all-E)±0.3			39
Apple	*Malus domestica var. Golden Delicious*	A01DJ	Apples	Spain	Spain				fruit				36.8 (all-E)±2.2			39
Apple	*Malus domestica var. Granny Smith*	A01DJ	Apples	Spain	Spain				fruit				48.6 (all-E)±4.9			39

Table S6.6.3 Apples and similar (A01DH) (µg/100g) (continuation)

Food name	Scientific name	FoodEx2_ TermCode	FoodEx2_ TermName	Origin (country)	Purchase (country)	Water (%)	Process	Saponification	Part analysed	Colour	E(v. trans)-lycopene	E(v. trans)-zeaxanthin	Lutein	Luteoxanthin	Lycopene	Ref.
Apple	*Malus domestica var. Pink Lady*	A01DJ	Apples	Spain	Spain				fruit				18.6 (all-E)±0.2			39
Apple	*Malus domestica var. red Delicious*	A01DJ	Apples	Spain	Spain				fruit				28.4 (all-E)±0.9			39
Apple	*Malus domestica var. Royal Gala*	A01DJ	Apples	Spain	Spain				fruit				14.9 ± 0.1 (all-E)±0.1			39
Apple	*Malus pumila*	A01DJ	Apples	Germany	Germany	84.6			edible part				30			53
Apple	*Malus pumila*	A01DJ	Apples	Spain	Spain				edible part	yellowish			6			24
Apple	*Malus pumila*	A01DJ	Apples	Spain	Spain				edible part				6			24

Table S6.6.4 Apples and similar (A01DH) (µg/100g) (continuation)

Food name	Scientific name	FoodEx2_ TermCode	FoodEx2_ TermName	Origin (country)	Purchase (country)	Water (%)	Process	Saponification	Part analysed	Colour	Mutatoxanthin	Neochrome	Neoxanthin	Phytoene	Phytofluene	Ref.
Apple	*Malus domestica Elstar*	A01DJ	Apples	Germany	Germany	84.1			edible part				10			53
Apple	*Malus domestica var. Fuji*	A01DJ	Apples	Spain	Spain				fruit				12.9 ± 0.3 (9'Z) 67.3 ± 2.9 (all-E)			39

Table S6.6.4 Apples and similar (A01DH) (µg/100g) (continuation)

Food name	Scientific name	FoodEx2_ TermCode	FoodEx2_ TermName	Origin (country)	Purchase (country)	Water (%)	Process	Saponification	Part analysed	Colour	Mutatoxanthin	Neochrome	Neoxanthin	Phytoene	Phytofluene	Ref.
Apple	*Malus domestica var. Pink Lady*	A01DJ	Apples	Spain	Spain				fruit				11.5 ± 0.3 (9'Z) 22.9 ± 0.7 (all-E)			39
Apple	*Malus domestica var. red Delicious*	A01DJ	Apples	Spain	Spain				fruit				11.7 ± 0.3 (9'Z) 28.3 ± 1.6 (all-E)			39
Apple	*Malus domestica var. Golden Delicious*	A01DJ	Apples	Spain	Spain				fruit				16.6 ± 0.5 (9'Z) 87.2 ± 1.6 (all-E)			39
Apple	*Malus domestica var. Granny Smith*	A01DJ	Apples	Spain	Spain				fruit				11.9 ± 0.4 (9'Z) 16.0 ± 1.1 (all-E)			39
Apple	*Malus domestica var. Royal Gala*	A01DJ	Apples	Spain	Spain				fruit				10.6 ± 0.0 (9'Z) 31.2 ± 0.9 (all-E)			39
Apple	*Malus pumila*	A01DJ	Apples	Germany	Germany	84.6			edible part				3			53

Table S6.6.5 Apples and similar (A01DH) (μg/100g) (continuation)

Food name	Scientific name	FoodEx2_ TermCode	FoodEx2_ TermName	Origin (country)	Purchase (country)	Water (%)	Process	Saponification	Part analysed	Colour	Violaxanthin	Z(v. cis)-lycopene	Z(v. cis)-β-carotene	Z(v. cis)-β-cryptoxanthin	Zeaxanthin	Zeinoxanthin	Ref.
Apple	*Malus domestica Borkh. var. bravo esmolfe*	A01DJ	Apples	Portugal	Portugal				edible part	yellow					1.9		32
Apple	*Malus domestica Borkh. var. golden delicious*	A01DJ	Apples	Portugal	Portugal				edible part	yellow					nd		32
Apple	*Malus domestica Borkh. var. golden delicious*	A01DJ	Apples	Portugal	Portugal				edible part	yellow					1.8		32
Apple	*Malus domestica Borkh. var. jonagold*	A01DJ	Apples	Portugal	Portugal				edible part	yellow					nd		32
Apple	*Malus domestica Borkh. var. reineta parda*	A01DJ	Apples	Portugal	Portugal				edible part	green					2.0		32
Apple	*Malus domestica Borkh. var. royal gala*	A01DJ	Apples	Portugal	Portugal				edible part	red					3.0		32
Apple	*Malus domestica Borkh. var. starking*	A01DJ	Apples	Portugal	Portugal				edible part	red					nd		32
Apple	*Malus domestica Borkh. var. starking*	A01DJ	Apples	Portugal	Portugal				edible part	red					1.8		32
Apple	*Malus domestica Borkh. var. starking*	A01DJ	Apples	Portugal	Portugal				edible part	red					2.2		32
Apple	*Malus domestica var. Elstar*	A01DJ	Apples	Germany	Germany	84.1			edible part						3		53

Table S6.6.5 Apples and similar (A01DH) (µg/100g) (continuation)

Food name	Scientific name	FoodEx2_TermCode	FoodEx2_TermName	Origin (country)	Purchase (country)	Water (%)	Process	Saponification	Part analysed	Colour	Violaxanthin	Z(v. cis)-lycopene	Z(v. cis)-β-carotene	Z(v. cis)-β-cryptoxanthin	Zeaxanthin	Zeinoxanthin	Ref.
Apple	*Malus domestica var. red Delicious*	A01DJ#F10.A0F2S	Apples, QUALITATIVE-INFO = red	USA				no	with skin				6				28
Apple	*Malus pumila*	A01DJ	Apples	Germany	Germany	84.6			edible part						1		53
Apple	*Malus pumila*	A01DJ	Apples	Spain	Spain				edible part	yellow					tr.		24

Table S6.7.1 Pears and similar (A01DN)) (µg/100g)

Food name	Scientific name	FoodEx2_TermCode	FoodEx2_TermName	Origin (country)	Purchase (country)	Water (%)	Process	Saponification	Part analysed	Colour	α-carotene	β-carotene	β-cryptoxanthin	ζ-carotene	Antheraxanthin	Ref.
Pear	*Cucumis sativus L.*	A01DP	Pears	Spain	Spain				edible part	white		2	3			24
Pear	*Pyrus communis*	A01DP	Pears	Spain	Spain				edible part			2	3			24
Pear	*Pyrus communis L.*	A01DP	Pears	Germany	Germany	84.7			edible part			4	1		1	53
Pear	*Pyrus communis L. var. rocha*	A01DP	Pears	Portugal	Portugal				edible part	green		180	750			32
Pear	*Pyrus communis, L.*	A01DP#F20.A07QF$F20.A07RD	Pears, PART-CONSUMED-ANALYSED = W/o peel, W/o seeds	Spain	Spain	83			without skin or seeds	white		2±0.5	3±0.3			24
Pear	*Pyrus malus L.*	A01DP#F20.A07QF$F20.A07RD	Pears, PART-CONSUMED-ANALYSED = W/o peel, W/o seeds	Spain	Spain	86			without skin or seeds	white		20±4	8±4			24

Table S6.7.2 Pears and similar (A01DN) (µg/100g) (continuation)

Food name	Scientific name	FoodEx2_TermCode	FoodEx2_TermName	Origin (country)	Purchase (country)	Water (%)	Process	Saponification	Part analysed	Colour	E(v. trans)-lycopene	E(v. trans)-zeaxanthin	Lutein	Luteoxanthin	Lycopene	Ref.
Pear	*Cucumis sativus L.*	A01DP	Pears	Spain	Spain				edible part	white			11			24
Pear	*Pyrus communis L.*	A01DP	Pears	Germany	Germany	84.7			edible part				60			53
Pear	*Pyrus communis L.*	A01DP	Pears	Spain	Spain				edible part				11			24
Pear	*Pyrus communis L. var. rocha*	A01DP	Pears	Portugal	Portugal				edible part	green			990			32
Pear	*Pyrus communis, L.*	A01DP#F20.A07QF$F20.A07RD	Pears, PART-CONSUMED-ANALYSED = W/o peel, W/o seeds	Spain	Spain	83			without skin or seeds	white			11±2			24
Pear	*Pyrus malus L.*	A01DP#F20.A07QF$F20.A07RD	Pears, PART-CONSUMED-ANALYSED = W/o peel, W/o seeds	Spain	Spain	86			without skin or seeds	white			6±0.6			24

Table S6.7.3 Pears and similar (A01DN) (µg/100g) (continuation)

Food name	Scientific name	FoodEx2_TermCode	FoodEx2_TermName	Origin (country)	Purchase (country)	Water (%)	Process	Saponification	Part analysed	Colour	Mutatoxanthin	Neochrome	Neoxanthin	Phytoene	Phytofluene	Ref.
Pear	*Pyrus communis L.*	A01DP	Pears	Germany	Germany	84.7			edible part				10			53
Pear	*Pyrus communis, L.*	A01DP#F20.A07QF$F20.A07RD	Pears, PART-CONSUMED-ANALYSED = W/o peel, W/o seeds	Spain	Spain	83			without skin or seeds	white				28±8		24

Table S6.7.4 Pears and similar (A01DN) (µg/100g) (continuation)

Food name	Scientific name	FoodEx2_TermCode	FoodEx2_TermName	Origin (country)	Purchase (country)	Water (%)	Process	Saponification	Part analysed	Colour	Violaxanthin	Z(v. cis)-lycopene	Z(v. cis)-β-carotene	Z(v. cis)-β-cryptoxanthin	Zeaxanthin	Zeinoxanthin	Ref.
Pear	*Pyrus communis L. var. rocha*	A01DP	Pears	Portugal	Portugal				edible part	green					500		32
Pear	*Pyrus malus L.*	A01DP#F20.A07QF$F20.A07RD	Pears, PART-CONSUMED-ANALYSED = W/o peel, W/o seeds	Spain	Spain	86			without skin or seeds	white					tr.		24

Table S6.8.1 Loquats and similar (A0DVY) (µg/100g)

Food name	Scientific name	FoodEx2_TermCode	FoodEx2_TermName	Origin (country)	Purchase (country)	Water (%)	Process	Saponification	Part analysed	Colour	α-carotene	β-carotene	β-cryptoxanthin	ζ-carotene	Antheraxanthin	Ref.
Loquat	*Eriobotrya japonica L.*	A01DL	Loquats	Spain	Spain					orange	34.8	613.2	929.2			56
Loquat	*Eriobotrya Japonica L.*	A01DL	Loquats	Spain	Spain				edible part	orange			929.2			56
Loquat	*Eriobotrya japonica L.*	A01DL	Loquats	Spain	Spain				edible part			977	663			24
Loquat	*Eriobotrya japonica, L.*	A01DL	Loquats	Spain	Spain				edible part	orange		977	663			24
Loquat	*Eriobotrya japonica, L.*	A01DL#F20.A07QF$F20.A07QK	Loquats, PART-CONSUMED-ANALYSED = W/o peel, W/o stone	Spain	Spain	84			without skin or stone	green		977±132	663±109			24
Loquat	*Eriobotrya japonica, L.*	A01DL#F20.A07QF$F20.A07QK	Loquats, PART-CONSUMED-ANALYSED = W/o peel, W/o stone	Spain	Spain	84			without skin or stone	green		994±181	651±109			24

Table S6.8.2 Loquats and similar (A0DVY) (µg/100g) (continuation)

Food name	Scientific name	FoodEx2_ TermCode	FoodEx2_ TermName	Origin (country)	Purchase (country)	Water (%)	Process	Saponification	Part analysed	Colour	Auroxanthin	E(v. trans)-α-carotene	E(v. trans)-β-carotene	E(v. trans)-β-cryptoxanthin	E(v. trans)-lutein	Ref.
Loquat	*Eriobotrya Japonica L.*	A01DL	Loquats	Spain	Spain				edible part	orange		34.8	613.2			56

Table S6.8.3 Loquats and similar (A0DVY) (continuation)

Food name	Scientific name	FoodEx2_ TermCode	FoodEx2_ TermName	Origin (country)	Purchase (country)	Water (%)	Process	Saponification	Part analysed	Colour	Violaxanthin	Z(v. cis)-lycopene	Z(v. cis)-β-carotene	Z(v. cis)-β-cryptoxanthin	Zeaxanthin	Zeinoxanthin	Ref.
Loquat	*Eriobotrya japonica L.*	A01DL	Loquats	Spain	Spain					orange			14.9				56
Loquat	*Eriobotrya Japonica L.*	A01DL	Loquats	Spain	Spain				edible part	orange			14.9				56

Table S6.9.1 Apricots and similar (A0DVX) (μg/100g)

Food name	Scientific name	FoodEx2_ TermCode	FoodEx2_ TermName	Origin (country)	Purchase (country)	Water (%)	Process	Saponification	Part analysed	Colour	α-carotene	β-carotene	β-cryptoxanthin	ζ-carotene	Antheraxanthin	Ref.
Appricot	*Prunus armeniaca L.*	A01GF	Apricots	Germany	Germany	87.6			edible part		20	710	60		9	53
Apricot	*Prunus armeniaca L.*	A01GF	Apricots	Spain	Spain				flesh	white		924±353	254±91			60
Apricot	*Prunus armeniaca L.*	A01GF#F20. A0F7P	Apricots, PART-CONSUMED-ANALYSED = Only peel	Spain	Spain				peel	white (dark rose)		1969± 423	628±47			60
Apricot	*Prunus armeniaca L.*	A01GF	Apricots	Spain	Spain				flesh	white		1078± 475	267±148			60
Apricot	*Prunus armeniaca L.*	A01GF#F20. A0F7P	Apricots, PART-CONSUMED-ANALYSED = Only peel	Spain	Spain				peel	white (light red)		4059	640			60
Apricot	*Prunus armeniaca L.*	A01GF	Apricots	Spain	Spain				flesh	white		1189± 582	325±85			60
Apricot	*Prunus armeniaca L.*	A01GF#F20. A0F7P	Apricots, PART-CONSUMED-ANALYSED = Only peel	Spain	Spain				peel	white		2139± 462	671±115			60

Table S6.9.1 Apricots and similar (A0DVX) (µg/100g) (continuation)

Food name	Scientific name	FoodEx2_ TermCode	FoodEx2_ TermName	Origin (country)	Purchase (country)	Water (%)	Process	Saponification	Part analysed	Colour	α-carotene	β-carotene	β-cryptoxanthin	ζ-carotene	Antheraxanthin	Ref.
Apricot	*Prunus armeniaca L.*	A01GF	Apricots	Spain	Spain				flesh	white		1724±471	734±93			60
Apricot	*Prunus armeniaca L.*	A01GF#F20.A0F7P	Apricots, PART-CONSUMED-ANALYSED = Only peel	Spain	Spain				peel	yellow		4378±890	1060± 194			60
Apricot	*Prunus armeniaca L.*	A01GF	Apricots	Spain	Spain				flesh	white		1948±774	681±30			60
Apricot	*Prunus armeniaca L.*	A01GF#F20.A0F7P	Apricots, PART-CONSUMED-ANALYSED = Only peel	Spain	Spain				peel	yellow (red)		3566±366	940±72			60
Apricot	*Prunus armeniaca L.*	A01GF	Apricots	Spain	Spain				flesh	yellow		2007±561	211±76			60
Apricot	*Prunus armeniaca L.*	A01GF#F20.A0F7P	Apricots, PART-CONSUMED-ANALYSED = Only peel	Spain	Spain				peel	yellow (red)		3687±477	594±24			60
Apricot	*Prunus armeniaca L.*	A01GF	Apricots	Spain	Spain				flesh	yellow		2199±425	317±84			60
Apricot	*Prunus armeniaca L.*	A01GF#F20.A0F7P	Apricots, PART-CONSUMED-ANALYSED = Only peel	Spain	Spain				peel	yellow (red)		9567±422	716±96			60
Apricot	*Prunus armeniaca L.*	A01GF	Apricots	Spain	Spain				flesh	yellow		2026±264	667±63			60
Apricot	*Prunus armeniaca L.*	A01GF#F20.A0F7P	Apricots, PART-CONSUMED-ANALYSED = Only peel	Spain	Spain				peel	yellow (red)		7609±371	1267 ±180			60
Apricot	*Prunus armeniaca L.*	A01GF	Apricots	Spain	Spain				flesh	yellow		1971±404	978±59			60

Table S6.9.1 Apricots and similar (A0DVX) (µg/100g) (continuation)

Food name	Scientific name	FoodEx2_ TermCode	FoodEx2_ TermName	Origin (country)	Purchase (country)	Water (%)	Process	Saponification	Part analysed	Colour	α-carotene	β-carotene	β-cryptoxanthin	ζ-carotene	Antheraxanthin	Ref.
Apricot	*Prunus armeniaca L.*	A01GF#F20. A0F7P	Apricots, PART-CONSUMED-ANALYSED = Only peel	Spain	Spain				peel	yellow		6009±401	1451 ±158			60
Apricot	*Prunus armeniaca L.*	A01GF	Apricots	Spain	Spain				flesh	yellow		3374±254	674±123			60
Apricot	*Prunus armeniaca L.*	A01GF#F20. A0F7P	Apricots, PART-CONSUMED-ANALYSED = Only peel	Spain	Spain				peel	yellow		7647±158	951±75			60
Apricot	*Prunus armeniaca L.*	A01GF	Apricots	Spain	Spain				flesh	light orange		2648±166	914±94			60
Apricot	*Prunus armeniaca L.*	A01GF#F20. A0F7P	Apricots, PART-CONSUMED-ANALYSED = Only peel	Spain	Spain				peel	light orange (red)		8049±727	1444±51			60
Apricot	*Prunus armeniaca L.*	A01GF	Apricots	Spain	Spain				flesh	light orange		4992±697	944±136			60
Apricot	*Prunus armeniaca L.*	A01GF#F20. A0F7P	Apricots, PART-CONSUMED-ANALYSED = Only peel	Spain	Spain				peel	yellow (light red)		10014 ±380	1090±71			60
Apricot	*Prunus armeniaca L.*	A01GF	Apricots	Spain	Spain				flesh	light orange		4642±726	1143±257			60
Apricot	*Prunus armeniaca L.*	A01GF#F20. A0F7P	Apricots, PART-CONSUMED-ANALYSED = Only peel	Spain	Spain				peel	light orange (dark rose)		9634±717	1562±79			60
Apricot	*Prunus armeniaca L.*	A01GF	Apricots	Spain	Spain				flesh	light orange		6882±429	1314±157			60

Table S6.9.1 Apricots and similar (A0DVX) (µg/100g) (continuation)

Food name	Scientific name	FoodEx2_ TermCode	FoodEx2_ TermName	Origin (country)	Purchase (country)	Water (%)	Process	Saponification	Part analysed	Colour	α-carotene	β-carotene	β-cryptoxanthin	ζ-carotene	Antheraxanthin	Ref.
Apricot	*Prunus armeniaca L.*	A01GF#F20. A0F7P	Apricots, PART-CONSUMED-ANALYSED = Only peel	Spain	Spain				peel	white (light red)		10081 ±845	1579±254			60
Apricot	*Prunus armeniaca L.*	A01GF	Apricots	Spain	Spain				flesh	orange		5307±304	1504±206			60
Apricot	*Prunus armeniaca L.*	A01GF#F20. A0F7P	Apricots, PART-CONSUMED-ANALYSED = Only peel	Spain	Spain				peel	light orange (red)		8464±350	1857±307			60
Apricot	*Prunus armeniaca L.*	A01GF	Apricots	Spain	Spain				flesh	orange		4953±80	1300±85			60
Apricot	*Prunus armeniaca L.*	A01GF#F20. A0F7P	Apricots, PART-CONSUMED-ANALYSED = Only peel	Spain	Spain				peel	light orange (red)		14086 ±374	2264±102			60
Apricot	*Prunus armeniaca L.*	A01GF	Apricots	Spain	Spain				flesh	orange		5505±359	2147±310			60
Apricot	*Prunus armeniaca L.*	A01GF#F20. A0F7P	Apricots, PART-CONSUMED-ANALYSED = Only peel	Spain	Spain				peel	orange		19580 ±350	2261±262			60
Apricot	*Prunus armeniaca L.*	A01GF	Apricots	Spain	Spain				flesh	orange		8944±933	679±98			60
Apricot	*Prunus armeniaca L.*	A01GF#F20. A0F7P	Apricots, PART-CONSUMED-ANALYSED = Only peel	Spain	Spain				peel	light orange (red)		13076 ±1001	1479±176			60
Apricot	*Prunus armeniaca L.*	A01GF	Apricots	Spain	Spain				flesh	orange		5616±86	2228±264			60

Table S6.9.1 Apricots and similar (A0DVX) (µg/100g) (continuation)

Food name	Scientific name	FoodEx2_ TermCode	FoodEx2_ TermName	Origin (country)	Purchase (country)	Water (%)	Process	Saponification	Part analysed	Colour	α-carotene	β-carotene	β-cryptoxanthin	ζ-carotene	Antheraxanthin	Ref.
Apricot	*Prunus armeniaca L.*	A01GF#F20. A0F7P	Apricots, PART-CONSUMED-ANALYSED = Only peel	Spain	Spain				peel	orange		17996 ±320	3031±502			60
Apricot	*Prunus armeniaca L.*	A01GF	Apricots	Spain	Spain				flesh	orange		8050±313	1432±240			60
Apricot	*Prunus armeniaca L.*	A01GF#F20. A0F7P	Apricots, PART-CONSUMED-ANALYSED = Only peel	Spain	Spain				peel	light orange		14927 ±1523	1375±191			60
Apricot	*Prunus armeniaca L.*	A01GF	Apricots	Spain	Spain				flesh	orange		7307±562	1689±40			60
Apricot	*Prunus armeniaca L.*	A01GF#F20. A0F7P	Apricots, PART-CONSUMED-ANALYSED = Only peel	Spain	Spain				peel	orange		18051 ±2455	2831±405			60
Apricot	*Prunus armeniaca L.*	A01GF	Apricots	Spain	Spain				flesh	orange		8214±601	2118±425			60
Apricot	*Prunus armeniaca L.*	A01GF#F20. A0F7P	Apricots, PART-CONSUMED-ANALYSED = Only peel	Spain	Spain				peel	light orange (red)		19226 ±419	2128±278			60
Apricot	*Prunus armeniaca L.*	A01GF	Apricots	Spain	Spain				flesh	orange		6856±754	3045±704			60
Apricot	*Prunus armeniaca L.*	A01GF#F20. A0F7P	Apricots, PART-CONSUMED-ANALYSED = Only peel	Spain	Spain				peel	orange		23609 ±292	2802±78			60
Apricot	*Prunus armeniaca L.*	A01GF	Apricots	Spain	Spain				flesh	orange		9794±38	2355±702			60

Table S6.9.1 Apricots and similar (A0DVX) (µg/100g) (continuation)

Food name	Scientific name	FoodEx2_ TermCode	FoodEx2_ TermName	Origin (country)	Purchase (country)	Water (%)	Process	Saponification	Part analysed	Colour	α-carotene	β-carotene	β-cryptoxanthin	ζ-carotene	Antheraxanthin	Ref.
Apricot	*Prunus armeniaca L.*	A01GF#F20. A0F7P	Apricots, PART-CONSUMED-ANALYSED = Only peel	Spain	Spain				peel	light orange (red)		12752± 505	2373±451			60
Apricot	*Prunus armeniaca L.*	A01GF	Apricots	Spain	Spain				flesh	orange		7911±180	2211±336			60
Apricot	*Prunus armeniaca L.*	A01GF#F20. A0F7P	Apricots, PART-CONSUMED-ANALYSED = Only peel	Spain	Spain				peel	orange		30500± 1245	3412±160			60
Apricot	*Prunus armeniaca L.*	A01GF	Apricots	Spain	Spain				flesh	orange		11058± 537	729±75			60
Apricot	*Prunus armeniaca L.*	A01GF#F20. A0F7P	Apricots, PART-CONSUMED-ANALYSED = Only peel	Spain	Spain				peel	orange (orange-red)		25636± 586	3789±290			60
Apricot	*Prunus armeniaca L.*	A01GF	Apricots	Spain	Spain				flesh	orange		8267± 343	3137±483			60
Apricot	*Prunus armeniaca L.*	A01GF#F20. A0F7P	Apricots, PART-CONSUMED-ANALYSED = Only peel	Spain	Spain				peel	orange (light red)		18572± 961	3254±310			60
Apricot	*Prunus armeniaca L.*	A01GF	Apricots	Spain	Spain				flesh	orange		9421±477	2529±226			60
Apricot	*Prunus armeniaca L.*	A01GF#F20. A0F7P	Apricots, PART-CONSUMED-ANALYSED = Only peel	Spain	Spain				peel	orange (red)		19985± 1015	1493±63			60
Apricot	*Prunus armeniaca L.*	A01GF	Apricots	Spain	Spain				flesh	orange		10272± 566	2399±110			60

Table S6.9.1 Apricots and similar (A0DVX) (μg/100g) (continuation)

Food name	Scientific name	FoodEx2_ TermCode	FoodEx2_ TermName	Origin (country)	Purchase (country)	Water (%)	Process	Saponification	Part analysed	Colour	α-carotene	β-carotene	β-cryptoxanthin	ζ-carotene	Antheraxanthin	Ref.
Apricot	*Prunus armeniaca L.*	A01GF#F20. A0F7P	Apricots, PART-CONSUMED-ANALYSED = Only peel	Spain	Spain				peel	orange (orange-red)		21645± 1680	2145±200			60
Apricot	*Prunus armeniaca L.*	A01GF	Apricots	Spain	Spain				flesh	orange		9248±147	3672± 1126			60
Apricot	*Prunus armeniaca L.*	A01GF#F20. A0F7P	Apricots, PART-CONSUMED-ANALYSED = Only peel	Spain	Spain				peel	orange (red)		19657± 471	4835±498			60
Apricot	*Prunus armeniaca L.*	A01GF	Apricots	Italy	Italy	-			-	-	nd – 44	585–3800				24
Apricot	*Prunus armeniaca L.*	A01GF	Apricots	Spain	Spain				edible part	yellow - orange		140	28			24
Apricot	*Prunus armeniaca L.*	A01GF	Apricots	Hungary	Hungary				edible part	unknown		26,6±2.5				61
Apricot	*Prunus armeniaca L. var. Rojo Pasión*	A01GF	Apricots	Spain	Spain				flesh	light orange		3741±216	678±111			60
Apricot	*Prunus armeniaca L. var. Rojo Pasión*	A01GF#F20. A0F7P	Apricots, PART-CONSUMED-ANALYSED = Only peel	Spain	Spain				peel	yellow (red)		12934 ±1423	1079±50			60
Apricot	*Prunus armeniaca L. var. Búlida*	A01GF	Apricots	Spain	Spain				flesh	light orange		3668±365	1627±274			60
Apricot	*Prunus armeniaca L. var. Currot*	A01GF	Apricots	Spain	Spain				flesh	white		1264±476	672±291			60

Table S6.9.1 Apricots and similar (A0DVX) (µg/100g) (continuation)

Food name	Scientific name	FoodEx2_ TermCode	FoodEx2_ TermName	Origin (country)	Purchase (country)	Water (%)	Process	Saponification	Part analysed	Colour	α-carotene	β-carotene	β-cryptoxanthin	ζ-carotene	Antheraxanthin	Ref.
Apricot	*Prunus armeniaca L. var. Currot*	A01GF#F20. A0F7P	Apricots, PART-CONSUMED-ANALYSED = Only peel	Spain	Spain				peel	yellow (light red)		2638±257	1159±232			60
Apricot	*Prunus armeniaca L. var. Dorada*	A01GF	Apricots	Spain	Spain				flesh	light orange		4325±718	1361±380			60
Apricot	*Prunus armeniaca L. var. Dorada*	A01GF#F20. A0F7P	Apricots, PART-CONSUMED-ANALYSED = Only peel	Spain	Spain				peel	light orange		10659 ±321	2157±206			60
Apricot	*Prunus armeniaca L. var. Keckemetska ruza*	A01GF	Apricots	Croatia	Croatia				fruit - mature	orange		795.5± 25.17				62
Apricot	*Prunus armeniaca L. var. Madjarska najbola*	A01GF	Apricots	Croatia	Croatia				fruit - Immature	green		54.35± 2.05				62
Apricot	*Prunus armeniaca L. var. Madjarska najbola*	A01GF	Apricots	Croatia	Croatia				fruit -semi-mature	yellow		235.40± 12.25				62
Apricot	*Prunus armeniaca L. var. Madjarska najbola*	A01GF	Apricots	Croatia	Croatia				fruit - mature	orange		585.4± 20.08				62
Apricot	*Prunus armeniaca L. var. Madjarska najbola*	A01GF	Apricots	Croatia	Croatia				fruit - Immature	green		75.06± 8.76				62

Table S6.9.1 Apricots and similar (A0DVX) (µg/100g) (continuation)

Food name	Scientific name	FoodEx2_ TermCode	FoodEx2_ TermName	Origin (country)	Purchase (country)	Water (%)	Process	Saponification	Part analysed	Colour	α-carotene	β-carotene	β-cryptoxanthin	ζ-carotene	Antheraxanthin	Ref.
Apricot	*Prunus armeniaca L. var. Madjarska najbola*	A01GF	Apricots	Croatia	Croatia				fruit -semi-mature	yellow		418.7± 19.87				62
Apricot	*Prunus armeniaca L. var. Madjarska najbola*	A01GF	Apricots	Croatia	Croatia				fruit - Immature	green	nd	176.69± 9.95				62
Apricot	*Prunus armeniaca L. var. Madjarska najbola*	A01GF	Apricots	Croatia	Croatia				fruit -semi-mature	yellow	14.81± 0.56	622.98± 10.11				62
Apricot	*Prunus armeniaca L. var. Madjarska najbola*	A01GF	Apricots	Croatia	Croatia				fruit - mature	orange	22.31± 0.92	1074.99± 5.47				62
Apricot	*Prunus armeniaca L. var. Madjarska najbola*	A01GF	Apricots	Croatia	Croatia				fruit - Immature	green	12.64± 0.45	203.01± 2.97				62
Apricot	*Prunus armeniaca L. var. Madjarska najbola*	A01GF	Apricots	Croatia	Croatia				fruit -semi-mature	yellow	32.35± 1.08	750.5± 5.18				62
Apricot	*Prunus armeniaca L. var. Madjarska najbola*	A01GF	Apricots	Croatia	Croatia				fruit - mature	orange	43.67± 2.15	1374.95± 13.85				62
Apricot	*Prunus armeniaca L. var. Mauricio*	A01GF	Apricots	Spain	Spain				flesh	yellow		3089 ±535	506±114			60

Table S6.9.1 Apricots and similar (A0DVX) (µg/100g) (continuation)

Food name	Scientific name	FoodEx2_ TermCode	FoodEx2_ TermName	Origin (country)	Purchase (country)	Water (%)	Process	Saponification	Part analysed	Colour	α-carotene	β-carotene	β-cryptoxanthin	ζ-carotene	Antheraxanthin	Ref.
Apricot	*Prunus armeniaca L. var. Mauricio*	A01GF#F20. A0F7P	Apricots, PART-CONSUMED-ANALYSED = Only peel	Spain	Spain				peel	yellow		6420±550	1076±100			60
Apricot	*Prunus armeniaca L. var. Murciana*	A01GF	Apricots	Spain	Spain				flesh	light orange		5768±432	1052±193			60
Apricot	*Prunus armeniaca L. var. Murciana*	A01GF#F20. A0F7P	Apricots, PART-CONSUMED-ANALYSED = Only peel	Spain	Spain				peel	light orange (red)		18410 ±780	1828±140			60
Apricot	*Prunus armeniaca L. var. Selene*	A01GF	Apricots	Spain	Spain				flesh	orange		9265±56	2393±338			60
Apricot	*Prunus armeniaca L. var. Selene*	A01GF#F20. A0F7P	Apricots, PART-CONSUMED-ANALYSED = Only peel	Spain	Spain				peel	orange		17063± 1351	2955±93			60
Apricot	*Prunus armeniaca L. var. Velika rana*	A01GF	Apricots	Croatia	Croatia				fruit - Immature	green	nd	107.57± 2.55				62
Apricot	*Prunus armeniaca L. var. Velika rana*	A01GF	Apricots	Croatia	Croatia				fruit -semi-mature	yellow	nd	454.06± 5.87				62
Apricot	*Prunus armeniaca L. var. Velika rana*	A01GF	Apricots	Croatia	Croatia				fruit - mature	orange	nd	828.58± 10.15				62
Apricot	*Prunus armeniaca L. var. Velika rana*	A01GF	Apricots	Croatia	Croatia				fruit - Immature	green	nd	154.46± 2.76				62

Table S6.9.1 Apricots and similar (A0DVX) (µg/100g) (continuation)

Food name	Scientific name	FoodEx2_ TermCode	FoodEx2_ TermName	Origin (country)	Purchase (country)	Water (%)	Process	Saponification	Part analysed	Colour	α-carotene	β-carotene	β-cryptoxanthin	ζ-carotene	Antheraxanthin	Ref.
Apricot	*Prunus armeniaca L. var. Velika rana*	A01GF	Apricots	Croatia	Croatia				fruit -semi-mature	yellow	nd	585.69± 4.85				62
Apricot	*Prunus armeniaca L. var. Velika rana*	A01GF	Apricots	Croatia	Croatia				fruit - mature	orange	9.67±0,15	948.33± 7.88				62
Apricot	*Prunus armeniaca L., var. Búlida*	A01GF#F20. A0F7P	Apricots, PART-CONSUMED-ANALYSED = Only peel	Spain	Spain				peel	light orange		7545 ±224	2338 ±194			60
Apricot	*Prunus armeniaca L., var. Cafona*	A01GF	Apricots	Italy	Italy					yellow / orange		1153±90				63
Apricot	*Prunus armeniaca L., var. Cafona*	A01GF	Apricots	Italy	Italy					yellow / orange		795±97				63
Apricot	*Prunus armeniaca L., var. Pellecchiella*	A01GF	Apricots	Italy	Italy					yellow / orange		1680±31				63
Apricot	*Prunus armeniaca L., var. Pellecchiella*	A01GF	Apricots	Italy	Italy					yellow / orange		2218±7				63
Apricot	*Prunus armeniaca, L.*	A01GF	Apricots	Spain	Spain				edible part	orange		140	28			24
Apricot	*Prunus armeniaca, L.*	A01GF#F20. A07QK	Apricots, PART-CONSUMED-ANALYSED = W/o stone	Spain	Spain	89			Without stone	orange			28±11			24

Table S6.9.2 Apricots and similar (A0DVX) (µg/100g) (continuation)

Food name	**Scientific name**	**FoodEx2_ TermCode**	**FoodEx2_ TermName**	**Origin (country)**	**Purchase (country)**	**Water (%)**	**Process**	**Saponification**	**Part analysed**	**Colour**	**Auroxanthin**	**E(v. trans)-α-carotene**	**E(v. trans)-β-carotene**	**E(v. trans)-β-cryptoxanthin**	**E(v. trans)-lutein**	**Ref.**
Apricot	*Prunus armeniaca, var Bergeron*	A01GF	Apricots	France	France					orange			1890-2370			64

Table S6.9.3 Apricots and similar (A0DVX) (µg/100g) (continuation)

**Food name**	**Scientific name**	**FoodEx2_ TermCode**	**FoodEx2_ TermName**	**Origin (country)**	**Purchase (country)**	**Water (%)**	**Process**	**Saponification**	**Part analysed**	**Colour**	**E(v. trans)-lycopene**	**E(v. trans)-zeaxanthin**	**Lutein**	**Luteoxanthin**	**Lycopene**	**Ref.**
Apricot	*Prunus armeniaca L.*	A01GF	Apricots	Italy	Italy	-			-	-			123–188		54	25
Apricot	*Prunus armeniaca L.*	A01GF	Apricots	Germany	Germany	87.6			edible part				40			53
Apricot	*Prunus armeniaca L. var. Madjarska najbola*	A01GF	Apricots	Croatia	Croatia				fruit - Immature	green			18.47± 1.82			62
Apricot	*Prunus armeniaca L. var. Madjarska najbola*	A01GF	Apricots	Croatia	Croatia				fruit -semi-mature	yellow			71.7± 5.77			62
Apricot	*Prunus armeniaca L. var. Madjarska najbola*	A01GF	Apricots	Croatia	Croatia				fruit - mature	orange			131.3± 3.45			62

Table S6.9.3 Apricots and similar (A0DVX) (µg/100g) (continuation)

Food name	Scientific name	FoodEx2_ TermCode	FoodEx2_ TermName	Origin (country)	Purchase (country)	Water (%)	Process	Saponification	Part analysed	Colour	E(v. trans)-lycopene	E(v. trans)-zeaxanthin	Lutein	Luteoxanthin	Lycopene	Ref.
Apricot	*Prunus armeniaca L. var. Madjarska najbola*	A01GF	Apricots	Croatia	Croatia				fruit - Immature	green			35.15± 2.85			62
Apricot	*Prunus armeniaca L. var. Madjarska najbola*	A01GF	Apricots	Croatia	Croatia				fruit -semi-mature	yellow			96.84± 6.87			62
Apricot	*Prunus armeniaca L. var. Madjarska najbola*	A01GF	Apricots	Croatia	Croatia				fruit - mature	orange			188.11± 7.82			62
Apricot	*Prunus armeniaca L. var. Velika rana*	A01GF	Apricots	Croatia	Croatia				fruit - immature	green			10.18± 0.33			62
Apricot	*Prunus armeniaca L. var. Velika rana*	A01GF	Apricots	Croatia	Croatia				fruit -semi-mature	yellow			75.29± 1.08			62
Apricot	*Prunus armeniaca L. var. Velika rana*	A01GF	Apricots	Croatia	Croatia				fruit - mature	orange			123.44± 2.82			62
Apricot	*Prunus armeniaca L. var. Velika rana*	A01GF	Apricots	Croatia	Croatia				fruit - Immature	green			21.39± 1.25			62
Apricot	*Prunus armeniaca L. var. Velika rana*	A01GF	Apricots	Croatia	Croatia				fruit -semi-mature	yellow			88.18± 5.87			62
Apricot	*Prunus armeniaca L. var. Velika rana*	A01GF	Apricots	Croatia	Croatia				fruit - mature	orange			131.35± 4.55			62

Table S6.9.3 Apricots and similar (A0DVX) (µg/100g) (continuation)

Food name	Scientific name	FoodEx2_ TermCode	FoodEx2_ TermName	Origin (country)	Purchase (country)	Water (%)	Process	Saponification	Part analysed	Colour	E(v. trans)-lycopene	E(v. trans)-zeaxanthin	Lutein	Luteoxanthin	Lycopene	Ref.
Apricot	*Prunus armeniaca, L.*	A01GF	Apricots	Spain	Spain				edible part	orange			tr.			24
Apricot	*Prunus armeniaca, L.*	A01GF#F20. A07QK	Apricots, PART-CONSUMED-ANALYSED = W/o stone	Spain	Spain	89			without stone	orange			tr.		tr.	24

Table S6.9.4 Apricots and similar (A0DVX) (µg/100g) (continuation)

Food name	Scientific name	FoodEx2_ TermCode	FoodEx2_ TermName	Origin (country)	Purchase (country)	Water (%)	Process	Saponification	Part analysed	Colour	Mutatoxanthin	Neochrome	Neoxanthin	Phytoene	Phytofluene	Ref.
Apricot	*Prunus armeniaca L.*	A01GF	Apricots	Germany	Germany	87.6			edible part				14	1050	450	53
Apricot	*Prunus armeniaca L. var. Cafona*	A01GF	Apricots	Italy	Italy					yellow - orange				609±10	565±4	63
Apricot	*Prunus armeniaca L. var. Cafona*	A01GF	Apricots	Italy	Italy					yellow - orange				804±19	646±26	63
Apricot	*Prunus armeniaca L. var. Pellecchiella*	A01GF	Apricots	Italy	Italy					yellow - orange				641±14	697±11	63
Apricot	*Prunus armeniaca L. var. Pellecchiella*	A01GF	Apricots	Italy	Italy					yellow - orange				627±10	750±6	63

Table S6.9.4 Apricots and similar (A0DVX) (µg/100g) (continuation)

Food name	Scientific name	FoodEx2_ TermCode	FoodEx2_ TermName	Origin (country)	Purchase (country)	Water (%)	Process	Saponification	Part analysed	Colour	Mutatoxanthin	Neochrome	Neoxanthin	Phytoene	Phytofluene	Ref.
Apricot	*Prunus armeniaca, L.*	A01GF	Apricots	Spain	Spain					orange				276(6-710)	95(2-238)	22
Apricot	*Prunus armeniaca, L.*	A01GF	Apricots	Spain	Spain					white				1260(660-2190)	890(660-2190)	22
Apricot	*Prunus armeniaca, L.*	A01GF	Apricots	Spain	Spain					yellow				1340 (650-2650)	970 (450-1720)	22
Apricot	*Prunus armeniaca, L.*	A01GF	Apricots	Spain	Spain					light orange				2260 (1540-3290)	1440 (910-2020)	22
Apricot	*Prunus armeniaca, L.*	A01GF	Apricots	Spain	Spain					orange				1680 (1230-4970)	2780 (90-3310)	22
Apricot	*Prunus armeniaca, L.*	A01GF	Apricots	Spain	Spain					orange				100	60	22
Apricot	*Prunus armeniaca, L.*	A01GF	Apricots	Spain	Spain					orange				240	140	22
Apricot	*Prunus armeniaca, L.*	A01GF#F20. A07QK	Apricots, PART-CONSUMED-ANALYSED = W/o stone	Spain	Spain	85			without stone	orange				3151± 613		24

Table S6.9.5 Apricots and similar (A0DVX) (µg/100g) (continuation)

Food name	Scientific name	FoodEx2_ TermCode	FoodEx2_ TermName	Origin (country)	Purchase (country)	Water (%)	Process	Saponification	Part analysed	Colour	Violaxanthin	Z(v. cis)-lycopene	Z(v. cis)-β-carotene	Z(v. cis)-β-cryptoxanthin	Zeaxanthin	Zeinoxanthin	Ref.
Apricot	*Prunus armeniaca L.*	A01GF	Apricots	Italy	Italy	-									nd – 39		25
Apricot	*Prunus armeniaca L.*	A01GF	Apricots	Germany	Germany	87.6			edible part						6		53
Apricot	*Prunus armeniaca L.*	A01GF	Apricots	Spain	Spain				edible part	yellow orange							24
Apricot	*Prunus armeniaca L.*	A01GF	Apricots	Hungary	Hungary				edible part								61
Apricot	*Prunus armeniaca L. var. Keckemetska ruza*	A01GF	Apricots	Croatia	Croatia				fruit - immature	green					5.92-38.96		62
Apricot	*Prunus armeniaca L. var. Madjarska najbola*	A01GF	Apricots	Croatia	Croatia				fruit - immature	green					5.92±0.45		62
Apricot	*Prunus armeniaca L. var. Madjarska najbola*	A01GF	Apricots	Croatia	Croatia				fruit - semi-mature	yellow					18.62± 0.55		62
Apricot	*Prunus armeniaca L. var. Madjarska najbola*	A01GF	Apricots	Croatia	Croatia				fruit - mature	orange					25.43± 1.96		62
Apricot	*Prunus armeniaca L. var. Madjarska najbola*	A01GF	Apricots	Croatia	Croatia				fruit - immature	green					11.94± 0.56		62

Table S6.9.5 Apricots and similar (A0DVX) (µg/100g) (continuation)

Food name	Scientific name	FoodEx2_ TermCode	FoodEx2_ TermName	Origin (country)	Purchase (country)	Water (%)	Process	Saponification	Part analysed	Colour	Violaxanthin	Z(v. cis)-lycopene	Z(v. cis)-β-carotene	Z(v. cis)-β-cryptoxanthin	Zeaxanthin	Zeinoxanthin	Ref.
Apricot	*Prunus armeniaca L. var. Madjarska najbola*	A01GF	Apricots	Croatia	Croatia				fruit - semi-mature	yellow					31.07± 1.95		62
Apricot	*Prunus armeniaca L. var. Madjarska najbola*	A01GF	Apricots	Croatia	Croatia				fruit - mature	orange					38.96± 2.07		62
Apricot	*Prunus armeniaca L. var. Velika rana*	A01GF	Apricots	Croatia	Croatia				fruit - immature	green					nd		62
Apricot	*Prunus armeniaca L. var. Velika rana*	A01GF	Apricots	Croatia	Croatia				fruit - semi-mature	yellow					nd		62
Apricot	*Prunus armeniaca L. var. Velika rana*	A01GF	Apricots	Croatia	Croatia				fruit - mature	orange					nd		62
Apricot	*Prunus armeniaca L. var. Velika rana*	A01GF	Apricots	Croatia	Croatia				fruit - immature	green					nd		62
Apricot	*Prunus armeniaca L. var. Velika rana*	A01GF	Apricots	Croatia	Croatia				fruit - semi-mature	yellow					nd		62
Apricot	*Prunus armeniaca L. var. Velika rana*	A01GF	Apricots	Croatia	Croatia				fruit - mature	orange					nd		62
Apricot	*Prunus armeniaca, L.*	A01GF	Apricots	Spain	Spain				edible part	Orange					tr.		24

Table S6.9.5 Apricots and similar (A0DVX) (µg/100g) (continuation)

Food name	Scientific name	FoodEx2_TermCode	FoodEx2_TermName	Origin (country)	Purchase (country)	Water (%)	Process	Saponification	Part analysed	Colour	Violaxanthin	Z(v. cis)-lycopene	Z(v. cis)-β-carotene	Z(v. cis)-β-cryptoxanthin	Zeaxanthin	Zeinoxanthin	Ref.
Apricot	*Prunus armeniaca, L.*	A01GF#F20.A07QK	Apricots, PART-CONSUMED-ANALYSED = W/o stone	Spain	Spain	89			without stone	orange					tr.		24
Apricot	*Prunus armeniaca, var. Bergeron*	A01GF	Apricots	France	France	-			whole plant	orange			170-550	420-450			64

Table S6.10.1 Cherries and similar (A01GG) (µg/100g)

Food name	Scientific name	FoodEx2_TermCode	FoodEx2_TermName	Origin (country)	Purchase (country)	Water (%)	Process	Saponification	Part analysed	Colour	α-carotene	β-carotene	β-cryptoxanthin	ζ-carotene	Antheraxanthin	Ref.
Cherry	*Prunus avium, L.*	A01GK	Cherries (Sweet)	Germany	Germany	82.6			edible part		6	20	9		60	53
Cherry	*Prunus cerasus, L. var. austera*	A01GH	Sour cherries	Germany	Germany	87.4			edible part		60	400	20		70	53

Table S6.10.2 Cherries and similar (A01GG) (µg/100g) (continuation)

Food name	Scientific name	FoodEx2_TermCode	FoodEx2_TermName	Origin (country)	Purchase (country)	Water (%)	Process	Saponification	Part analysed	Colour	E(v. trans)-lycopene	E(v. trans)-zeaxanthin	Lutein	Luteoxanthin	Lycopene	Ref.
Cherry	*Prunus avium, L.*	A01GK	Cherries (Sweet)	Germany	Germany	82.6			edible part				60			53
Cherry	*Prunus avium, L.*	A01GK	Cherries (Sweet)	Spain	Spain				edible part	red			44		10	24
Cherry	*Prunus avium, L.*	A01GK	Cherries (Sweet)	Spain	Spain				edible part				44		10	24
Cherry	*Prunus avium, L.*	A01GK#F20 .A07QK	Cherries (Sweet), PART-CONSUMED-ANALYSED = W/o stone	Spain	Spain	84			without stone	red			44±12		10±0	24
Cherry	*Prunus avium, L. var. de saco*	A01GK	Cherries (Sweet)	Portugal	Portugal				edible part	red			430			32
Cherry	*Prunus cerasus, L. var. austera*	A01GH	Sour cherries	Germany	Germany	87.4			edible part				50			53

Table S6.10.3 Cherries and similar (A01GG) (µg/100g) (continuation)

Food name	Scientific name	FoodEx2_TermCode	FoodEx2_TermName	Origin (country)	Purchase (country)	Water (%)	Process	Saponification	Part analysed	Colour	Mutatoxanthin	Neochrome	Neoxanthin	Phytoene	Phytofluene	Ref.
Cherry	*Prunus avium, L.*	A01GK	Cherries (Sweet)	Germany	Germany	82.6			edible part				70			53
Cherry	*Prunus cerasus, L. var. austera*	A01GH	Sour cherries	Germany	Germany	87.4			edible part					50		53

Table S6.10.4 Cherries and similar (A01GG) (µg/100g) (continuation)

Food name	Scientific name	FoodEx2_ TermCode	FoodEx2_ TermName	Origin (country)	Purchase (country)	Water (%)	Process	Saponification	Part analysed	Colour	Violaxanthin	Z(v. cis)-lycopene	Z(v. cis)-β-carotene	Z(v. cis)-β-cryptoxanthin	Zeaxanthin	Zeinoxanthin	Ref.
Cherry	*Prunus avium, L.*	A01GK	Cherries (Sweet)	Spain	Spain				edible part	red					4		24
Cherry	*Prunus avium, L.*	A01GK	Cherries (Sweet)	Spain	Spain				edible part						4		24
Cherry	*Prunus avium, L.*	A01GK#F20 .A07QK	Cherries (Sweet), PART-CONSUMED-ANALYSED = W/o stone	Spain	Spain	84			without stone	red					4±0.4		24
Cherry	*Prunus avium, L. var. de saco*	A01GK	Cherries (Sweet)	Portugal	Portugal				edible part	red					410		32
Cherry	*Prunus cerasus, L. var. austera*	A01GH	Sour cherries	Germany	Germany	87.4			edible part						20		53

Table S6.11.1 Peaches and similar (A01GL) (µg/100g)

Food name	Scientific name	FoodEx2_ TermCode	FoodEx2_ TermName	Origin (country)	Purchase (country)	Water (%)	Process	Saponification	Part analysed	Colour	α-carotene	β-carotene	β-cryptoxanthin	ζ-carotene	Antheraxanthin	Ref.
Nectarine	*Prunus pérsica, L.*	A01GN#F20.A0F7P	Nectarines, PART-CONSUMED-ANALYSED = Only peel	Italy	Italy				Peel			5–307	nd – 31			25
Nectarine	*Prunus pérsica, L.*	A01GN#F20.A07QF$F20.A07QK	Nectarines, PART-CONSUMED-ANALYSED = W/o peel, W/o stone	Italy	Italy				flesh			2–131	nd – 21			25
Nectarine	*Prunus pérsica, L.*	A01GN	Nectarines	Germany	Germany	88.7			edible part		140	330	80	9	80	53
Nectarine	*Prunus pérsica, L.*	A01GN	Nectarines	Spain	Spain				edible part	orange		50	30			24
Nectarine	*Prunus pérsica, L.*	A01GN	Nectarines	Spain	Spain				edible part			50	30			24
Nectarine	*Prunus pérsica, L.*	A01GN	Nectarines	Spain	Spain				pulp	orange						22
Nectarine	*Prunus persica, L. var. nucipersica*	A01GN	Nectarines	USA				no			0		5			28
Peach	*Prunus pérsica, L.*	A01GM#F20.A0F7P	Common peaches, PART-CONSUMED-ANALYSED = Only peel	Italy	Italy				peel			11–379	nd – 36			25
Peach	*Prunus pérsica, L.*	A01GM#F20.A07QF$F20.A07QK	Common peaches, PART-CONSUMED-ANALYSED = W/o peel, W/o stone	Italy	Italy				flesh			4–168	nd – 16			25
Peach	*Prunus pérsica, L.*	A01GM	Common peaches	Germany	Germany	90.6			edible part			100	50	14	140	53

Table S6.11.1 Peaches and similar (A01GL) (µg/100g) (continuation)

Food name	Scientific name	FoodEx2_ TermCode	FoodEx2_ TermName	Origin (country)	Purchase (country)	Water (%)	Process	Saponification	Part analysed	Colour	α-carotene	β-carotene	β-cryptoxanthin	ζ-carotene	Antheraxanthin	Ref.
Peach	*Prunus persica, L.*	A01GM	Common peaches	Spain	Spain				fruit	orange		40.8	59			56
Peach	*Prunus pérsica, L.*	A01GM	Common peaches	Spain	Spain				edible part	orange	3	64	74			24
Peach	*Prunus pérsica, L.*	A01GM	Common peaches	Spain	Spain				edible part	orange		334	141			24
Peach	*Prunus pérsica, L.*	A01GM	Common peaches	Spain	Spain				edible part	orange			59			36
Peach	*Prunus pérsica, L.*	A01GM	Common peaches	Spain	Spain				edible part		3	64	74			24
Peach	*Prunus pérsica, L.*	A01GM	Common peaches	Spain	Spain				edible part			334	141			24
Peach	*Prunus pérsica, L.*	A01GM#F20.A07QK	Common peaches, PART-CONSUMED-ANALYSED = W/o stone	Slovenia	Slovenia	-			skin and flesh	orange		0.172 - 0.867				65
Peach	*Prunus pérsica, L.*	A01GM	Common peaches	USA				no			0		0			28
Peach	*Prunus persica, L. Sieb. and Zuce*	A01GM#F20.A07QF$F20.A07QK	Common peaches, PART-CONSUMED-ANALYSED = W/o peel, W/o stone	Spain	Spain	85			without skin or stone	orange	3±2	64±16	74±13			24
Peach	*Prunus pérsica, L. var. m. carnival*	A01GM	Common peaches	Portugal	Portugal				edible part	orange	8.2	170	210			32
Peach	*Prunus pérsica, L. var. platycarpa*	A0DVL	Flat peaches	Italy	Italy				pulp, peel and Seed	orange		61900-344700				66

Table S6.11.2 Peaches and similar (A01GL) (µg/100g) (continuation)

Food name	Scientific name	FoodEx2_ TermCode	FoodEx2_ TermName	Origin (country)	Purchase (country)	Water (%)	Process	Saponification	Part analysed	Colour	Auroxanthin	E(v. trans)-α-carotene	E(v. trans)-β-carotene	E(v. trans)-β-cryptoxanthin	E(v. trans)-lutein	Ref.
Nectarine	*Prunus pérsica, L. var. nucipersica*	A01GN	Nectarines	USA				no					96		8	28
Peach	*Prunus persica, L.*	A01GM	Common peaches	Spain	Spain				fruit	orange			35.6			56
Peach	*Prunus pérsica, L.*	A01GM	Common peaches	Netherlands	Netherlands				edible part	orange			101±21	33±13	132±21	57
Peach	*Prunus pérsica, L.*	A01GM	Common peaches	Netherlands	Netherlands				edible part	orange			105.7 ±3.3	38.6±1.9	95.7±5.4	57
Peach	*Prunus pérsica, L.*	A01GM	Common peaches	Netherlands	Netherlands				edible part	orange			113±28	74±11	171±54	57
Peach	*Prunus pérsica, L.*	A01GM	Common peaches	Spain	Spain				edible part	orange			35.6±5.3			56
Peach	*Prunus pérsica, L.*	A01GM	Common peaches	USA				no					141		11	28

Table S6.11.3 Peaches and similar (A01GL) (µg/100g) (continuation)

Food name	Scientific name	FoodEx2_ TermCode	FoodEx2_ TermName	Origin (country)	Purchase (country)	Water (%)	Process	Saponification	Part analysed	Colour	E(v. trans)-lycopene	E(v. trans)-zeaxanthin	Lutein	Luteoxanthin	Lycopene	Ref.
Nectarine	*Prunus pérsica, L.*	A01GN	Nectarines	Germany	Germany	88.7			edible part				980			53
Nectarine	*Prunus pérsica, L. var. nucipersica*	A01GN	Nectarines	USA				no				4				28
Peach	*Prunus pérsica, L.*	A01GM	Common peaches	Germany	Germany	90.6			edible part				30			53
Peach	*Prunus pérsica, L.*	A01GM	Common peaches	Netherlands	Netherlands				edible part	orange		59±64				57
Peach	*Prunus pérsica, L.*	A01GM	Common peaches	Netherlands	Netherlands				edible part	orange		49.7±3.3				57
Peach	*Prunus pérsica, L.*	A01GM	Common peaches	Netherlands	Netherlands				edible part	orange		79±46				57
Peach	*Prunus pérsica, L.*	A01GM	Common peaches	Spain	Spain				edible part	orange			16			24
Peach	*Prunus pérsica, L.*	A01GM	Common peaches	Spain	Spain				edible part				16			24
Peach	*Prunus pérsica, L.*	A01GM#F20.A07QK	Common peaches, PART-CONSUMED-ANALYSED = W/o stone	Slovenia	Slovenia	-			skin and flesh	orange			0.0009 - 0.0043			65
Peach	*Prunus persica, L. Sieb. and Zuce*	A01GM#F20.A07QF$F20.A07QK	Common peaches, PART-CONSUMED-ANALYSED = W/o peel, PART-CONSUMED-ANALYSED = W/o stone	Spain	Spain	85			without skin or stone	Orange			16±4			24
Peach	*Prunus pérsica, L. var. m. carnival*	A01GM	Common peaches	Portugal	Portugal				edible pars	orange			75			32
Peach. raw	*Prunus pérsica, L.*	A01GM	Common peaches	USA				no				3				28

Table S6.11.4 Peaches and similar (A01GL) (µg/100g) (continuation)

Food name	Scientific name	FoodEx2_ TermCode	FoodEx2_ TermName	Origin (country)	Purchase (country)	Water (%)	Process	Saponification	Part analysed	Colour	Mutatoxanthin	Neochrome	Neoxanthin	Phytoene	Phytofluene	Ref.
Nectarine	*Prunus pérsica, L.*	A01GN	Nectarines	Germany	Germany	88.7			edible part				90	400	1220	53
Nectarine	*Prunus pérsica, L.*	A01GN	Nectarines	Spain	Spain				pulp	orange				400	120	22
Peach	*Prunus pérsica, L.*	A01GM	Common peaches	Germany	Germany	90.6			edible part				9	180	70	53
Peach	*Prunus pérsica, L.*	A01GM#F20.A07QF$F20.A07QK	Common peaches, PART-CONSUMED-ANALYSED = W/o peel, W/o stone	Spain	Spain				pulp	orange				110 (10-180)	47 (2-70)	22
Peach	*Prunus pérsica, L.*	A01GM	Common peaches	Spain	Spain				fruit	orange				10		22
Peach	*Prunus pérsica, L.*	A01GM	Common peaches	Spain	Spain				fruit	orange				10	30	22
Peach	*Prunus persica, L. Sieb. and Zuce*	A01GM#F20.A07QF$F20.A07QK	Common peaches, PART-CONSUMED-ANALYSED = W/o peel, W/o stone	Spain	Spain	85			without skin or stone	orange				524±125		24

Table S6.11.5 Peaches and similar (A01GL) (µg/100g) (continuation)

Food name	Scientific name	FoodEx2_ TermCode	FoodEx2_ TermName	Origin (country)	Purchase (country)	Water (%)	Process	Saponification	Part analysed	Colour	Violaxanthin	Z(v. cis)-lycopene	Z(v. cis)-β-carotene	Z(v. cis)-β-cryptoxanthin	Zeaxanthin	Zeinoxanthin	Ref.
Nectarine	*Prunus pérsica, L.*	A01GN	Nectarines	Germany	Germany	88.7			edible part						80		53
Nectarine	*Prunus pérsica, L. var. nucipersica*	A01GN	Nectarines	USA				no					12				28
Peach	*Prunus persica, L.*	A01GM	Common peaches	Spain	Spain				fruit	orange			5.19				56
Peach	*Prunus pérsica, L.*	A01GM	Common peaches	Germany	Germany	90.6			edible part						60		53
Peach	*Prunus pérsica, L.*	A01GM	Common peaches	Spain	Spain				edible part	orange					31		24
Peach	*Prunus pérsica, L.*	A01GM	Common peaches	Spain	Spain				edible part	orange			5.2±1.9				56
Peach	*Prunus pérsica, L.*	A01GM	Common peaches	Spain	Spain				edible part						31		24
Peach	*Prunus pérsica, L.*	A01GM	Common peaches	USA				no					24				28
Peach	*Prunus persica, L. Sieb. and Zuce*	A01GM#F20.A07QF$F20.A07QK	Common peaches, PART-CONSUMED-ANALYSED = W/o peel, W/o stone	Spain	Spain	85			without skin or stone	orange					31±9		24
Peach	*Prunus pérsica, L. var. m. carnival*	A01GM	Common peaches	Portugal	Portugal				edible part	orange					26		32

Table S6.12.1 Plums and similar (A01GP) (μg/100g)

Food name	Scientific name	FoodEx2_ TermCode	FoodEx2_ TermName	Origin (country)	Purchase (country)	Water (%)	Process	Saponification	Part analysed	Colour	α-carotene	β-carotene	β-cryptoxanthin	ζ-carotene	Antheraxanthin	Ref.
Mirabelle	*Prunus domestica, L. var. syriaca*	A01GS	Mirabelles	Germany	Germany	81.3					7	180	20		60	53
Plum	*Prunus domestica, L.*	A01GQ#F20.A0F7P	Plums, PART-CONSUMED-ANALYSED = Only peel	Italy	Italy				peel			217–410	3–39			25
Plum	*Prunus domestica, L.*	A01GQ#F20.A07QF$F20.A07QK	Plums, PART-CONSUMED-ANALYSED = W/o peel, W/o stone	Italy	Italy				flesh			40–188	3–13			25
Plum	*Prunus domestica, L.*	A01GQ	Plums	Germany	Germany	85.4						70	5		8	53
Plum	*Prunus domestica, L.*	A01GQ#F20.A07QF$F20.A07QK	Plums, PART-CONSUMED-ANALYSED = W/o peel, W/o stone	Spain	Spain	86			without skin or stone	yellow		117±18				24
Plum	*Prunus domestica, L.*	A01GQ#F20.A07QF$F20.A07QK	Plums, PART-CONSUMED-ANALYSED = W/o peel, W/o stone	Spain	Spain	86			without skin or stone	yellow		107±8				24
Plum	*Prunus domestica, L.*	A01GS	Mirabelles	Spain	Spain					yellow		117				24
Plum	*Prunus domestica, L.*	A01GS	Mirabelles	Spain	Spain					yellow		117				24
Plum	*Prunus domestica, L. var. zwetschge*	A01GQ	Plums	Germany	Germany	82					12	90	14		30	53

Table S6.12.2 Plums and similar (A01GP) (µg/100g) (continuation)

Food name	Scientific name	FoodEx2_ TermCode	FoodEx2_ TermName	Origin (country)	Purchase (country)	Water (%)	Process	Saponification	Part analysed	Colour	E(v. trans)-lycopene	E(v. trans)-zeaxanthin	Lutein	Luteoxanthin	Lycopene	Ref.
Mirabelle	*Prunus domestica, L. var. syriaca*	A01GS	Mirabelles	Germany	Germany	81.3							20			53
Plum	*Prunus domestica, L.*	A01GQ	Plums	Germany	Germany	85.4							160			53
Plum	*Prunus domestica, L.*	A01GQ#F20.A07QF$F20.A07QK	Plums, PART-CONSUMED-ANALYSED = W/o peel, W/o stone	Spain	Spain	86			without skin or stone	yellow			83±8			24
Plum	*Prunus domestica, L.*	A01GQ#F20.A07QF$F20.A07QK	Plums, PART-CONSUMED-ANALYSED = W/o peel, W/o stone	Spain	Spain	86			without skin or stone	yellow			56±6			24
Plum	*Prunus domestica, L.*	A01GS	Mirabelles	Spain	Spain					yellow			83			24
Plum	*Prunus domestica, L.*	A01GS	Mirabelles	Spain	Spain					yellow			83			24
Plum	*Prunus domestica, L. var. zwetschge*	A01GQ	Plums	Germany	Germany	82							110±20			53

Table S6.12.3 Plums and similar (A01GP) (µg/100g) (continuation)

Food name	Scientific name	FoodEx2_ TermCode	FoodEx2_ TermName	Origin (country)	Purchase (country)	Water (%)	Process	Saponification	Part analysed	Colour	Mutatoxanthin	Neochrome	Neoxanthin	Phytoene	Phytofluene	Ref.
Mirabelle	*Prunus domestica, L. var. syriaca*	A01GS	Mirabelles	Germany	Germany	81.3			edible part				6			53
Plum	*Prunus domestica, L.*	A01GQ	Plums	Germany	Germany	85.4			edible part				10			53

Table S6.13.1 Grapes and similar fruits (A01DV) (μg/100g)

Food name	Scientific name	FoodEx2_TermCode	FoodEx2_TermName	Origin (country)	Purchase (country)	Water (%)	Process	Saponification	Part analysed	Colour	α-carotene	β-carotene	β-cryptoxanthin	ζ-carotene	Antheraxanthin	Ref.
Grapes	*Vitis vinifera, L.*	A01DX	Table grapes	Spain	Spain				edible part	red		39				24
Grapes	*Vitis vinifera, L.*	A01DX#F10.A0F2R	Table grapes, QUALITATIVE-INFO = white	Spain	Spain	78				green		16±1				24
Grapes	*Vitis vinifera, L.*	A01DX	Table grapes	Germany	Germany	78.6					0.3	3	0.5		3	53
Grapes	*Vitis vinifera, L.*	A01DX	Table grapes	Italy	Italy					white/red		54-340				67
Grapes	*Vitis vinifera, L.*	A01DX	Table grapes	Portugal	Portugal				berries, seeds and wines			358				68
Grapes	*Vitis vinifera, L.*	A01DX#F10.A0F2R	Table grapes, QUALITATIVE-INFO = white	Spain	Spain					white		17				24
Grapes	*Vitis vinifera, L.*	A01DX#F10.A0F2R	Table grapes, QUALITATIVE-INFO = white	Spain	Spain	78				green		16±1				24
Grapes	*Vitis vinifera, L.*	A01DX	Table grapes	Spain	Spain				edible part	red		39				24
Grapes	*Vitis vinifera, L.*	A01DX#F10.A0F2R	Table grapes, QUALITATIVE-INFO = white	Spain	Spain					white		17				24
Grapes	*Vitis vinifera, L.*	A01DX#F10.A0F2Q	Table grapes, QUALITATIVE-INFO = green	USA				no		green	0		0			28
Grapes	*Vitis vinifera, L.*	A01DX#F10.A0F2S	Table grapes, QUALITATIVE-INFO = red	USA				no		red	0		0			28
Grapes	*Vitis vinifera, L. var. Barbera*	A01DX#F10.A0F2S	Table grapes, QUALITATIVE-INFO = red	Italy	Italy					red		110±10 - 1200±80				69
Grapes	*Vitis vinifera, L. var. Erbaluce*	A01DX#F10.A0F2R	Table grapes, QUALITATIVE-INFO = white	Italy	Italy					white		nd - 670±80				69

Table S6.13.1 Grapes and similar fruits (A01DV) (µg/100g)

Food name	Scientific name	FoodEx2_TermCode	FoodEx2_TermName	Origin (country)	Purchase (country)	Water (%)	Process	Saponification	Part analysed	Colour	α-carotene	β-carotene	β-cryptoxanthin	ζ-carotene	Antheraxanthin	Ref.
Grapes	*Vitis vinifera, L. var. Nebbiolo*	A01DX#F10.A0F2S	Table Sgrapes, QUALITATIVE-INFO = red	Italy	Italy					red		90±10 - 790±50				69
Kiwiberries	*Actinidia arguta*	A0CFT	Kiwiberries	Poland	Poland				Fruit with peel	green		59±22				70
Kiwiberries	*Actinidia arguta cv. 'Anna'*	A0CFT	Kiwiberries	Poland	Poland				Fruit with peel	green		110±36				70
Kiwiberries	*Actinidia arguta cv. 'Bingo'*	A0CFT	Kiwiberries	Poland	Poland				fruit with peel	green		96±16				70
Kiwiberries	*Actinidia arguta cv. 'Geneva'*	A0CFT	Kiwiberries	Poland	Poland				fruit with peel	green		118±34				70
Kiwiberries	*Actinidia arguta cv. 'Jumbo'*	A0CFT	Kiwiberries	Poland	Poland				fruit with peel	green		151±9				70
Kiwiberries	*Actinidia arguta cv. 'Weiki'*	A0CFT	Kiwiberries	Poland	Poland				fruit with peel	green		247±39				70

Table S6.13.2 Grapes and similar fruits (A01DV) (µg/100g) (continuation)

Food name	Scientific name	FoodEx2_TermCode	FoodEx2_TermName	Origin (country)	Purchase (country)	Water (%)	Process	Saponification	Part analysed	Colour	Auroxanthin	E(v. trans)-α-carotene	E(v. trans)-β-carotene	E(v. trans)-β-cryptoxanthin	E(v. trans)-lutein	Ref.
Grapes	*Vitis vinifera, L.*	A01DX#F10.A0F2Q	Table grapes, QUALITATIVE-INFO = green	USA				no		green			34		53	28
Grapes	*Vitis vinifera, L.*	A01DX#F10.A0F2S	Table grapes, QUALITATIVE-INFO = red	USA				no		red			18		24	28

Table S6.13.3 Grapes and similar fruits (A01DV) (µg/100g) (continuation)

Food name	Scientific name	FoodEx2_ TermCode	FoodEx2_ TermName	Origin (country)	Purchase (country)	Water (%)	Process	Saponification	Part analysed	Colour	E(v. trans)-lycopene	E(v. trans)-zeaxanthin	Lutein	Luteoxanthin	Lycopene	Ref.
Grapes	*Vitis vinifera, L.*	A01DX	Table grapes	Germany	Germany	78.6			edible part	unknown			10			53
Grapes	*Vitis vinifera, L.*	A01DX#F10 .A0F2R	Table grapes, QUALITATIVE-INFO = white	Spain	Spain	78			whole fruit	green			13±1			24
Grapes	*Vitis vinifera, L.*	A01DX#F10 .A0F2R	Table grapes, QUALITATIVE-INFO = white	Spain	Spain	78			whole fruit	green			13±1			24
Grapes	*Vitis vinifera, L.*	A01DX	Table grapes	Portugal	Portugal				berries, seeds and wines				106			68
Grapes	*Vitis vinifera, L.*	A01DX	Table grapes	Italy	Italy					white/red			13-132			67
Grapes	*Vitis vinifera, L.*	A01DX#F10 .A0F2R	Table grapes, QUALITATIVE-INFO = white	Spain	Spain				edible part	white			13			24
Grapes	*Vitis vinifera, L.*	A01DX#F10 .A0F2Q	Table grapes, QUALITATIVE-INFO = green	USA				no		green		6				28
Grapes	*Vitis vinifera, L.*	A01DX#F10 .A0F2S	Table grapes, QUALITATIVE-INFO = red	USA				no		red		4				28
Grapes	*Vitis vinifera, L. var. Barbera*	A01DX#F10 .A0F2S	Table grapes, QUALITATIVE-INFO = red	Italy	Italy					red			nd - 1380±110			69
Grapes	*Vitis vinifera, L. var. Erbaluce*	A01DX#F10 .A0F2R	Table grapes, QUALITATIVE-INFO = white	Italy	Italy					white			nd - 1320±130			69
Grapes	*Vitis vinifera, L. var. Nebbiolo*	A01DX#F10 .A0F2S	Table grapes, QUALITATIVE-INFO = red	Italy	Italy					red			240±10 - 1380±80			69
Kiwiberries	*Actinidia arguta*	A0CFT	Kiwiberries	Poland	Poland				fruit with peel	green			544±67			70

Table S6.13.3 Grapes and similar fruits (A01DV) (µg/100g) (continuation)

Food name	Scientific name	FoodEx2_ TermCode	FoodEx2_ TermName	Origin (country)	Purchase (country)	Water (%)	Process	Saponification	Part analysed	Colour	E(v. trans)-lycopene	E(v. trans)-zeaxanthin	Lutein	Luteoxanthin	Lycopene	Ref.
Kiwiberries	*Actinidia arguta, cv. 'Anna'*	A0CFT	Kiwiberries	Poland	Poland				fruit with peel	green			701±31			70
Kiwiberries	*Actinidia arguta, cv. 'Bingo'*	A0CFT	Kiwiberries	Poland	Poland				fruit with peel	green			398±4			70
Kiwiberries	*Actinidia arguta, cv. 'Geneva'*	A0CFT	Kiwiberries	Poland	Poland				fruit with peel	green			830±74			70
Kiwiberries	*Actinidia arguta, cv. 'Jumbo'*	A0CFT	Kiwiberries	Poland	Poland				fruit with peel	green			858±47			70
Kiwiberries	*Actinidia arguta, cv. 'Weiki'*	A0CFT	Kiwiberries	Poland	Poland				fruit with peel	green			1161±96			70

Table S6.13.4 Grapes and similar fruits (A01DV) (µg/100g) (continuation)

Food name	Scientific name	FoodEx2_ TermCode	FoodEx2_ TermName	Origin (country)	Purchase (country)	Water (%)	Process	Saponification	Part analysed	Colour	Mutatoxanthin	Neochrome	Neoxanthin	Phytoene	Phytofluene	Ref.
Grapes	*Vitis vinifera, L.*	A01DX	Table grapes	Germany	Germany	78.6							3			53

Table S6.13.5 Grapes and similar fruits (A01DV) (µg/100g) (continuation)

Food name	Scientific name	FoodEx2_ TermCode	FoodEx2_ TermName	Origin (country)	Purchase (country)	Water (%)	Process	Saponification	Part analysed	Colour	Violaxanthin	Z(v. cis)-lycopene	Z(v. cis)-β-carotene	Z(v. cis)-β-cryptoxanthin	Zeaxanthin	Zeinoxanthin	Ref.
Grapes	*Vitis vinifera, L.*	A01DX	Table grapes	Germany	Germany	78.6									3		53
Grapes	*Vitis vinifera, L.*	A01DX	Table grapes	Italy	Italy					white/ red	4.2-19.5				3.95-27.7		67
Grapes	*Vitis vinifera, L.*	A01DX#F10 .A0F2Q	Table grapes, QUALITATIVE-INFO = green	USA	USA			no		green			5				28
Grapes	*Vitis vinifera, L.*	A01DX#F10 .A0F2S	Table grapes, QUALITATIVE-INFO = red	USA	USA			no		red			0				28
Kiwiberries	*Actinidia arguta*	A0CFT	Kiwiberries	Poland	Poland				fruit with peel	green					21±6		70
Kiwiberries	*Actinidia arguta cv. 'Anna'*	A0CFT	Kiwiberries	Poland	Poland				fruit with peel	green					24±3		70
Kiwiberries	*Actinidia arguta cv. 'Bingo'*	A0CFT	Kiwiberries	Poland	Poland				fruit with peel	green					26±7		70
Kiwiberries	*Actinidia arguta cv. 'Geneva'*	A0CFT	Kiwiberries	Poland	Poland				fruit with peel	green					58±26		70
Kiwiberries	*Actinidia arguta cv. 'Jumbo'*	A0CFT	Kiwiberries	Poland	Poland				fruit with peel	green					103±6		70
Kiwiberries	*Actinidia arguta cv. 'Weiki'*	A0CFT	Kiwiberries	Poland	Poland				fruit with peel	green					57±11		70

Table S6.14.1 Strawberries and similar (A01DZ) (µg/100g)

Food name	Scientific name	FoodEx2_ TermCode	FoodEx2_ TermName	Origin (country)	Purchase (country)	Water (%)	Process	Saponification	Part analysed	Colour	α-carotene	β-carotene	β-cryptoxanthin	ζ-carotene	Antheraxanthin	Ref.
Strawberry	*Fragaria ananassa*	A01EA	Strawberries	Germany	Germany	90.5					0.2	5	0.5		0.4	53
Strawberry	*Fragaria elatior, Ehrh.*	A01EA	Strawberries	Spain	Spain	91				red	4±1					24
Wild strawberry	*Fragaria vesca, L.*	A01EB	Wild strawberries	Spain	Spain					red		4				24
Wild strawberry	*Fragaria vesca, L.*	A01EB	Wild strawberries	Bulgaria	Bulgaria					red-purple-blue-black		4.9±0.7				71
Wild strawberry	*Fragaria vesca, L.*	A01EB	Wild strawberries	Spain	Spain							4				24

Table S6.14.2 Strawberries and similar (A01DZ) (µg/100g) (continuation)

Food name	Scientific name	FoodEx2_ TermCode	FoodEx2_ TermName	Origin (country)	Purchase (country)	Water (%)	Process	Saponification	Part analysed	Colour	E(v. trans)-lycopene	E(v. trans)-zeaxanthin	Lutein	Luteoxanthin	Lycopene	Ref.
Strawberry	*Fragaria ananassa*	A01EA	Strawberries	Germany	Germany	90.5							40			53
Strawberry	*Fragaria vesca*	A01EA	Strawberries	Spain	Spain					red			14			24
Strawberry	*Fragaria elatior, Ehrh.*	A01EA	Strawberries	Spain	Spain	91				red			14±7			24
Wild strawberry	*Fragaria vesca L.*	A01EB	Wild strawberries	Bulgaria	Bulgaria					red-purple-blue-black			21±3.6			71
Wild strawberry	*Fragaria vesca, L.*	A01EB	Wild strawberries	Spain	Spain								14			24

Table S6.14.3 Strawberries and similar (A01DZ) (µg/100g) (continuation)

Food name	Scientific name	FoodEx2_TermCode	FoodEx2_TermName	Origin (country)	Purchase (country)	Water (%)	Process	Saponification	Part analysed	Colour	Mutatoxanthin	Neochrome	Neoxanthin	Phytoene	Phytofluene	Ref.
Strawberry	*Fragaria ananassa*	A01EA	Strawberries	Germany	Germany	90.5							2			53

Table S6.14.4 Strawberries and similar (A01DZ) (µg/100g) (continuation)

Food name	Scientific name	FoodEx2_TermCode	FoodEx2_TermName	Origin (country)	Purchase (country)	Water (%)	Process	Saponification	Part analysed	Colour	Violaxanthin	Z(v. cis)-lycopene	Z(v. cis)-β-carotene	Z(v. cis)-β-cryptoxanthin	Zeaxanthin	Zeinoxanthin	Ref.
Strawberry	*Fragaria elatior, Ehrh.*	A01EA	Strawberries	Spain	Spain	91				red					tr.		24
Wild strawberry	*Fragaria vesca, L.*	A01EB	Wild strawberries	Spain	Spain					red					tr.		24
Wild strawberry	*Fragaria vesca, L.*	A01EB	Wild strawberries	Bulgaria	Bulgaria					red-purple-blue-black					nd		71

Table S6.15.1 Cane fruits (A01ED) (µg/100g)

Food name	Scientific name	FoodEx2_ TermCode	FoodEx2_ TermName	Origin (country)	Purchase (country)	Water (%)	Process	Saponification	Part analysed	Colour	α-carotene	β-carotene	β-cryptoxanthin	ζ-carotene	Antheraxanthin	Ref.
Blackberry	*Rubus fruticosus L.*	A01EH	Boysenberries	Bulgaria	Bulgaria				erries		9.2±0.7	100±13	30±3.7			71
Blackberry	*Rubus ulmifolius L.*	A0DTY	Blackberries and similar-	Germany	Germany	83					20	110	8		20	53
Blackberry	*Rubus ulmifolius L.*	A0DTY	Blackberries and similar-	Spain	Spain					purple	4	78				24
Blackberry	*Rubus ulmifolius L.*	A0DTY	Blackberries and similar-	Spain	Spain						4	78				24
Raspberry	*Rubus idaeus L.*	A01EP	Raspberries (red and yellow)	Germany	Germany	85.3			edible part		25	11	9		20	53

Table S6.15.1 Cane fruits (A01ED) (µg/100g) (continuation)

Food name	Scientific name	FoodEx2_ TermCode	FoodEx2_ TermName	Origin (country)	Purchase (country)	Water (%)	Process	Saponification	Part analysed	Colour	α-carotene	β-carotene	β-cryptoxanthin	ζ-carotene	Antheraxanthin	Ref.
Raspberry	*Rubus idaeus L.*	A01EP	Raspberries (red and yellow)	Spain	Spain					red	12	8				24
Raspberry	*Rubus idaeus L.*	A01EP	Raspberries (red and yellow)	Spain	Spain						12	8				24
Raspberry	*Rubus ideaus L.*	A01EP	Raspberries (red and yellow)	Bulgaria	Bulgaria						24	9.3±3.3	5.9±1.5			71

Table S6.15.2 Cane fruits (A01ED) (µg/100g) (continuation)

Food name	Scientific name	FoodEx2_TermCode	FoodEx2_TermName	Origin (country)	Purchase (country)	Water (%)	Process	Saponification	Part analysed	Colour	E(v. trans)-lycopene	E(v. trans)-zeaxanthin	Lutein	Luteoxanthin	Lycopene	Ref.
Blackberry	*Rubus fruticosus L.*	A01EH	Boysenberries	Bulgaria	Bulgaria								270±33			71
Blackberry	*Rubus ulmifolius L.*	A0DTY	Blackberries and similar-	Germany	Germany	83							650			53
Raspberry	*Rubus idaeus L.*	A01EP	Raspberries (red and yellow)	Germany	Germany	85.3							210			53
Raspberry	*Rubus ideaus L.*	A01EP	Raspberries (red and yellow)	Bulgaria	Bulgaria								320±37			71
Raspberry	*Rubus idaeus cv. Sugana red*	A01EP#F10. A0F2S	Raspberries (red and yellow), QUALITATIVE-INFO = red	Italy	Italy	86 ± 1.5 %				red			1980± 140			72
Raspberry	*Rubus idaeus cv. Tulameen*	A01EP#F10. A0F2S	Raspberries (red and yellow), QUALITATIVE-INFO = red	Italy	Italy	86 ± 1.5 %				red			1140± 120			72

Table S6.15.2 Cane fruits (A01ED) (µg/100g) (continuation)

Food name	Scientific name	FoodEx2_TermCode	FoodEx2_TermName	Origin (country)	Purchase (country)	Water (%)	Process	Saponification	Part analysed	Colour	E(v. trans)-lycopene	E(v. trans)-zeaxanthin	Lutein	Luteoxanthin	Lycopene	Ref.
Raspberry	*Rubus idaeus L. cv. Alpen Gold*	A01EP#F10. A0F5H	Raspberries (red and yellow), QUALITATIVE-INFO = yellow	Italy	Italy	86 ± 1.5 %				yellow			2080±240			72
Raspberry	*Rubus idaeus L. cv. Anne*	A01EP#F10. A0F5H	Raspberries (red and yellow), QUALITATIVE-INFO = yellow	Italy	Italy	86 ± 1.5 %				yellow			1370±150			72
Raspberry	*Rubus idaeus L. cv. Sugana Giallo*	A01EP#F10. A0F5H	Raspberries (red and yellow), QUALITATIVE-INFO = yellow	Italy	Italy	86 ± 1.5 %				yellow			1570±40			72

Table S6.15.3 Cane fruits (A01ED) (µg/100g) (continuation)

Food name	Scientific name	FoodEx2_ TermCode	FoodEx2_ TermName	Origin (country)	Purchase (country)	Water (%)	Process	Saponification	Part analysed	Colour	Mutatoxanthin	Neochrome	Neoxanthin	Phytoene	Phytofluene	Ref.
Blackberry	*Rubus ulmifolius L.*	A0DTY	Blackberries and similar-	Germany	Germany	83							9			53
Raspberry	*Rubus idaeus L.*	A01EP	Raspberries (red and yellow)	Germany	Germany	85.3							40			53
Raspberry	*Rubus idaeus cv. Sugana red*	A01EP#F10. A0F2S	Raspberries (red and yellow), QUALITATIVE-INFO = red	Italy	Italy	86 ± 1.5				red				30±10		72
Raspberry	*Rubus idaeus cv. Tulameen*	A01EP#F10. A0F2S	Raspberries (red and yellow), QUALITATIVE-INFO = red	Italy	Italy	86 ± 1.5				red				60±10		72

Table S6.15.3 Cane fruits (A01ED) (µg/100g) (continuation)

Food name	Scientific name	FoodEx2_ TermCode	FoodEx2_ TermName	Origin (country)	Purchase (country)	Water (%)	Process	Saponification	Part analysed	Colour	Mutatoxanthin	Neochrome	Neoxanthin	Phytoene	Phytofluene	Ref.
Raspberry	*Rubus idaeus L. cv. Alpen Gold*	A01EP#F10. A0F5H	Raspberries (red and yellow), QUALITATIVE-INFO = yellow	Italy	Italy	86 ± 1.5				yellow				360±10		72
Raspberry	*Rubus idaeus L. cv. Anne*	A01EP#F10. A0F5H	Raspberries (red and yellow), QUALITATIVE-INFO = yellow	Italy	Italy	86 ± 1.5				yellow				120±10		72
Raspberry	*Rubus idaeus L. cv. Sugana Giallo*	A01EP#F10. A0F5H	Raspberries (red and yellow), QUALITATIVE-INFO = yellow	Italy	Italy	86 ± 1.5				yellow				60±5		72

Table S6.15.4 Cane fruits (A01ED) (µg/100g) (continuation)

Food name	Scientific name	FoodEx2_ TermCode	FoodEx2_ TermName	Origin (country)	Purchase (country)	Water (%)	Process	Saponification	Part analysed	Colour	Violaxanthin	Z(v. cis)-lycopene	Z(v. cis)-β-carotene	Z(v. cis)-β-cryptoxanthin	Zeaxanthin	Zeinoxanthin	Ref.
Blackberry	*Rubus fruticosus L.*	A01EH	Boysenberries	Bulgaria	Bulgaria										29±0.8		71
Raspberry	*Rubus idaeus L.*	A01EP	Raspberries (red and yellow)	Germany	Germany	85.3									11		53
Raspberry	*Rubus ideaus L.*	A01EP	Raspberries (red and yellow)	Bulgaria	Bulgaria										11±2.6		71
Raspberry	*Rubus idaeus cv Sugana red*	A01EP#F10. A0F2S	Raspberries (red and yellow), QUALITATIVE-INFO = red	Italy	Italy	86 ± 1.5				red					46±1.3		72

Table S6.15.4 Cane fruits (A01ED) (µg/100g) (continuation)

Food name	Scientific name	FoodEx2_ TermCode	FoodEx2_ TermName	Origin (country)	Purchase (country)	Water (%)	Process	Saponification	Part analysed	Colour	Violaxanthin	Z(v. cis)-lycopene	Z(v. cis)-β-carotene	Z(v. cis)-β-cryptoxanthin	Zeaxanthin	Zeinoxanthin	Ref.
Raspberry	*Rubus idaeus cv Tulameen*	A01EP#F10. A0F2S	Raspberries (red and yellow), QUALITATIVE-INFO = red	Italy	Italy	86 ± 1.5				red					32±3.1		72
Raspberry	*Rubus idaeus L. cv Alpen Gold*	A01EP#F10. A0F5H	Raspberries (red and yellow), QUALITATIVE-INFO = yellow	Italy	Italy	86 ± 1.5				yellow					39±4.7		72
Raspberry	*Rubus idaeus L. cv Anne*	A01EP#F10. A0F5H	Raspberries (red and yellow), QUALITATIVE-INFO = yellow	Italy	Italy	86 ± 1.5				yellow					14±0.4		72
Raspberry	*Rubus idaeus L. cv Sugana Giallo*	A01EP#F10. A0F5H	Raspberries (red and yellow), QUALITATIVE-INFO = yellow	Italy	Italy	86 ± 1.5				yellow					49±3.1		72

Table S6.16.1 Blueberries and similar (A04JJ) (µg/100g)

Food name	Scientific name	FoodEx2_ TermCode	FoodEx2_ TermName	Origin (country)	Purchase (country)	Water (%)	Process	Saponification	Part analysed	Colour	α-carotene	β-carotene	β-cryptoxanthin	ζ-carotene	Antheraxanthin	Ref.
Bilberry	*Vaccinium myrtillus L.*	A01FF	Bilberries (European blueberries)	Germany	Germany	83.8						9	2			53
Bilberry	*Vaccinium myrtillus L.*	A01FF	Bilberries (European blueberries)	Finland	Finland	87.53						47				73

Table S6.16.1 Blueberries and similar (A04JJ) (µg/100g) (continuation)

Food name	Scientific name	FoodEx2_ TermCode	FoodEx2_ TermName	Origin (country)	Purchase (country)	Water (%)	Process	Saponification	Part analysed	Colour	α-carotene	β-carotene	β-cryptoxanthin	ζ-carotene	Antheraxanthin	Ref.
Blueberry	*Vaccinium myrtillus L.*	A01FF	Bilberries (European blueberries)	Bulgaria	Bulgaria				berries			49±10	5.1±1.0			71
Blueberry	*Vaccinum myrtillus L*	A01FF	Bilberries (European blueberries)	Spain	Spain					blue		204				24
Blueberry	*Vaccinum myrtillus L*	A01FF	Bilberries (European blueberries)	Spain	Spain					dark blue		204				24
Jostaberry	*Ribes nidigrolaria L.*	A0CFP	Jostaberries	Germany	Germany	82.6					20	200	20		90	53
Sea buckhorn	*Hippophae rhamnoides L. var. Mara SBT*	A01FZ	Sea buckthorns	Romania	Romania	8.53			berries	orange	3667.9	445777.8± 20130.8	69178.3± 8700.6			74

Table S6.16.2 Blueberries and similar (A04JJ) (µg/100g) (continuation)

Food name	Scientific name	FoodEx2_ TermCode	FoodEx2_ TermName	Origin (country)	Purchase (country)	Water (%)	Process	Saponification	Part analysed	Colour	E(v. trans)-lycopene	E(v. trans)-zeaxanthin	Lutein	Luteoxanthin	Lycopene	Ref.
Bilberry	*Vaccinium myrtillus L.*	A01FF	Bilberries (European blueberries)	Germany	Germany	83.8							140			53
Bilberry	*Vaccinium myrtillus L.*	A01FF	Bilberries (European blueberries)	Finland	Finland	87.53							184			73
Blueberry	*Vaccinium myrtillus L.*	A01FF	Bilberries (European blueberries)	Bulgaria	Bulgaria				berries				230±23			71
Jostaberry	*Ribes nidigrolaria L.*	A0CFP	Jostaberries	Germany	Germany	82.6							260			53

Table S6.16.3 Blueberries and similar (A04JJ) (µg/100g) (continuation)

Food name	Scientific name	FoodEx2_ TermCode	FoodEx2_ TermName	Origin (country)	Purchase (country)	Water (%)	Process	Saponification	Part analysed	Colour	Mutatoxanthin	Neochrome	Neoxanthin	Phytoene	Phytofluene	Ref.
Bilberry	*Vaccinium myrtillus L.*	A01FF	Bilberries (European blueberries)	Germany	Germany	83.8							5			53
Jostaberry	*Ribes nidigrolaria L.*	A0CFP	Jostaberries	Germany	Germany	82.6							122			53

Table S6.16.4 Blueberries and similar (A04JJ) (µg/100g) (continuation)

Food name	Scientific name	FoodEx2_TermCode	FoodEx2_TermName	Origin (country)	Purchase (country)	Water (%)	Process	Saponification	Part analysed	Colour	Violaxanthin	Z(v. cis)-lycopene	Z(v. cis)-β-carotene	Z(v. cis)-β-cryptoxanthin	Zeaxanthin	Zeinoxanthin	Ref.
Blueberry	*Vaccinium myrtillus L.*	A01FF	Bilberries (European blueberries)	Bulgaria	Bulgaria										14±2.9		71
Jostaberry	*Ribes nidigrolaria L.*	A0CFP	Jostaberries	Germany	Germany	82.6									40		53
Sea buckhorn	*Hippophae rhamnoides L.*	A01FZ	Sea buckthorns	Romania	Romania					orange			299061.8± 11600.8		35911.3± 6653.4		74

Table S6.17.1 Currants and similar (A0DSV) (µg/100g)

Food name	Scientific name	FoodEx2_TermCode	FoodEx2_TermName	Origin (country)	Purchase (country)	Water (%)	Process	Saponification	Part analysed	Colour	α-carotene	β-carotene	β-cryptoxanthin	ζ-carotene	Antheraxanthin	Ref.
Black currant	*Ribes nigrum*	A01FN	Blackcurrants	Germany	Germany	81.9				black		14			6	53
Black currant	*Ribes nigrum L.*	A01FN	Blackcurrants	Bulgaria	Bulgaria							62±8				71
red currant	*Ribes rubrum L.*	A01FP	Redcurrants	Germany	Germany	84.5				red	3	9			7	53
red currant	*Ribes rubrum L.*	A01FP	Redcurrants	Bulgaria	Bulgaria							13±2.7				71

Table S6.17.2 Currants and similar (A0DSV) (µg/100g) (continuation)

Food name	Scientific name	FoodEx2_TermCode	FoodEx2_TermName	Origin (country)	Purchase (country)	Water (%)	Process	Saponification	Part analysed	Colour	E(v. trans)-lycopene	E(v. trans)-zeaxanthin	Lutein	Luteoxanthin	Lycopene	Ref.
Black currant	*Ribes nigrum*	A01FN	Blackcurrants	Germany	Germany	81.9				black			180			53
Black currant	*Ribes nigrum L.*	A01FN	Blackcurrants	Bulgaria	Bulgaria								210±9.8			71
red currant	*Ribes rubrum L.*	A01FP	Redcurrants	Germany	Germany	84.5				red			70			53
red currant	*Ribes rubrum L.*	A01FP	Redcurrants	Bulgaria	Bulgaria								28±4.9			71

Table S6.17.3 Currants and similar (A0DSV) (µg/100g) (continuation)

Food name	Scientific name	FoodEx2_TermCode	FoodEx2_TermName	Origin (country)	Purchase (country)	Water (%)	Process	Saponification	Part analysed	Colour	Mutatoxanthin	Neochrome	Neoxanthin	Phytoene	Phytofluene	Ref.
Black currant	*Ribes nigrum L.*	A01FN	Blackcurrants	Germany	Germany	81.9				black			14			71
red currant	*Ribes rubrum L.*	A01FP	Redcurrants	Germany	Germany	84.5				red			3			71

Table S6.17.4 Currants and similar (A0DSV) (µg/100g) (continuation)

Food name	Scientific name	FoodEx2_TermCode	FoodEx2_TermName	Origin (country)	Purchase (country)	Water (%)	Process	Saponification	Part analysed	Colour	Violaxanthin	Z(v. cis)-lycopene	Z(v. cis)-β-carotene	Z(v. cis)-β-cryptoxanthin	Zeaxanthin	Zeinoxanthin	Ref.
Black currant	*Ribes nigrum L.*	A01FN	Blackcurrants	Bulgaria	Bulgaria										5.7±0.6		71
red currant	*Ribes rubrum L.*	A01FP	Redcurrants	Germany	Germany	84.5				red					2		53
red currant	*Ribes rubrum L.*	A01FP	Redcurrants	Bulgaria	Bulgaria										3.8±0.3		71

Table S6.18.1 Gooseberries and similar (A01FQ) (µg/100g)

Food name	Scientific name	FoodEx2_ TermCode	FoodEx2_ TermName	Origin (country)	Purchase (country)	Water (%)	Process	Saponification	Part analysed	Colour	α-carotene	β-carotene	β-cryptoxanthin	ζ-carotene	Antheraxanthin	Ref.
Gooseberry	*Ribes grosularia L.*	A0CFN	Gooseberries (green, red and yellow)	Germany	Germany	83.8					5	70			7	53

Table S6.18.2 Gooseberries and similar (A01FQ) (µg/100g) (continuation)

Food name	Scientific name	FoodEx2_ TermCode	FoodEx2_ TermName	Origin (country)	Purchase (country)	Water (%)	Process	Saponification	Part analysed	Colour	E(v. trans)-lycopene	E(v. trans)-zeaxanthin	Lutein	Luteoxanthin	Lycopene	Ref.
Gooseberry	*Ribes grosularia L.*	A0CFN	Gooseberries (green, red and yellow)	Germany	Germany	83.8							260			53

Table S6.18.3 Gooseberries and similar (A01FQ) (µg/100g) (continuation)

Food name	Scientific name	FoodEx2_ TermCode	FoodEx2_ TermName	Origin (country)	Purchase (country)	Water (%)	Process	Saponification	Part analysed	Colour	Mutatoxanthin	Neochrome	Neoxanthin	Phytoene	Phytofluene	Ref.
Gooseberry	*Ribes grosularia L.*	A0CFN	Gooseberries (green, red and yellow)	Germany	Germany	83.8							40			53

Table S6.19.1 Rose hips and similar (A01FR) (µg/100g)

Food name	Scientific name	FoodEx2_ TermCode	FoodEx2_ TermName	Origin (country)	Purchase (country)	Water (%)	Process	Saponification	Part analysed	Colour	α-carotene	β-carotene	β-cryptoxanthin	ζ-carotene	Antheraxanthin	Ref.
Rosehip	*Rosa canina L.*	A0DSS	Dog rose	Turkey	Turkey	-			whole plants	red and pink		18-370				75
Rosehip	*Rosa canina L.*	A0DSS	Dog rose	Germany	Germany				all sample			2495± 207.5				76
Rosehip	*Rosa canina L.*	A0DSS	Dog rose	Germany	Germany				all sample			500±35				76

Table S6.19.2 Rose hips and similar (A01FR) (µg/100g) (continuation)

Food name	Scientific name	FoodEx2_ TermCode	FoodEx2_ TermName	Origin (country)	Purchase (country)	Water (%)	Process	Saponification	Part analysed	Colour	Auroxanthin	E(v. trans)-α-carotene	E(v. trans)-β-carotene	E(v. trans)-β-cryptoxanthin	E(v. trans)-lutein	Ref.
Rosehip	*Rosa canina L.*	A0DSS	Dog rose	Germany	Germany				all sample				290±32.5			76
Rosehip	*Rosa canina L.*	A0DSS	Dog rose	Germany	Germany				pulp				3200±200	1200±100	100±10	77
Rosehip	*Rosa canina L.*	A0DSS	Dog rose	Germany	Germany				pulp				4200±200	100±100	700±100	77

Table S6.19.3 Rose hips and similar (A01FR) (µg/100g) (continuation)

Food name	Scientific name	FoodEx2_ TermCode	FoodEx2_ TermName	Origin (country)	Purchase (country)	Water (%)	Process	Saponification	Part analysed	Colour	E(v. trans)-lycopene	E(v. trans)-zeaxanthin	Lutein	Luteoxanthin	Lycopene	Ref.
Rosehip	*Rosa canina L.*	A0DSS	Dog rose	Germany	Germany				all sample						23842.5 ±777.5	76
Rosehip	*Rosa canina L.*	A0DSS	Dog rose	Germany	Germany				all sample						3615±215	76
Rosehip	*Rosa canina L.*	A0DSS	Dog rose	Germany	Germany				pulp		7900± 1500	2700±30				77
Rosehip	*Rosa canina L.*	A0DSS	Dog rose	Germany	Germany				pulp		7400± 1200	600±100				77

Table S6.19.4 Rose hips and similar (A01FR) (µg/100g) (continuation)

Food name	Scientific name	FoodEx2_ TermCode	FoodEx2_ TermName	Origin (country)	Purchase (country)	Water (%)	Process	Saponification	Part analysed	Colour	Mutatoxanthin	Neochrome	Neoxanthin	Phytoene	Phytofluene	Ref.
Rosehip	*Rosa canina L.*	A0DSS	Dog rose	Germany	Germany				pulp					400±100	nd	77
Rosehip	*Rosa canina L.*	A0DSS	Dog rose	Germany	Germany				pulp					700±100		77

Table S6.19.5 Rose hips and similar (A01FR) (µg/100g) (continuation)

Food name	Scientific name	FoodEx2_ TermCode	FoodEx2_ TermName	Origin (country)	Purchase (country)	Water (%)	Process	Saponification	Part analysed	Colour	Violaxanthin	Z(v. cis)-lycopene	Z(v. cis)-β-carotene	Z(v. cis)-β-cryptoxanthin	Zeaxanthin	Zeinoxanthin	Ref.
Rosehip	*Rosa canina L.*	A0DSS	Dog rose	Germany	Germany				pulp		300±10	8400± 1600	500±320				77
Rosehip	*Rosa canina L.*	A0DSS	Dog rose	Germany	Germany				pulp			6300±600	900±240				77

Table S6.20.1 Elderberries and similar (A04JM) (µg/100g)

Food name	Scientific name	FoodEx2_ TermCode	FoodEx2_ TermName	Origin (country)	Purchase (country)	Water (%)	Process	Saponification	Part analysed	Colour	α-carotene	β-carotene	β-cryptoxanthin	ζ-carotene	Antheraxanthin	Ref.
Saskatoon berry	*Amelanchier alnifolia Nutt.*	A0CFZ	Saskatoons	Poland	Poland						257-948					78

Table S6.20.2 Elderberries and similar (A04JM) (μg/100g) (continuation)

Food name	Scientific name	FoodEx2_TermCode	FoodEx2_TermName	Origin (country)	Purchase (country)	Water (%)	Process	Saponification	Part analysed	Colour	Auroxanthin	E(v. trans)-α-carotene	E(v. trans)-β-carotene	E(v. trans)-β-cryptoxanthin	E(v. trans)-lutein	Ref.
Saskatoon berry	*Amelanchier alnifolia Nutt.*	A0CFZ	Saskatoons	Poland	Poland								21368-28847		13574-17270	78

Table S6.20.3 Elderberries and similar (A04JM) (μg/100g) (continuation)

Food name	Scientific name	FoodEx2_TermCode	FoodEx2_TermName	Origin (country)	Purchase (country)	Water (%)	Process	Saponification	Part analysed	Colour	Mutatoxanthin	Neochrome	Neoxanthin	Phytoene	Phytofluene	Ref.
Autumn olive	*Elaeagnus umbellata*	A0DSC	Silverberries	Japan	Spain					red				410 (0-650)	110 (7-170)	22

Table S6.20.4 Elderberries and similar (A04JM) (μg/100g) (continuation)

Food name	Scientific name	FoodEx2_TermCode	FoodEx2_TermName	Origin (country)	Purchase (country)	Water (%)	Process	Saponification	Part analysed	Colour	Violaxanthin	Z(v. cis)-lycopene	Z(v. cis)-β-carotene	Z(v. cis)-β-cryptoxanthin	Zeaxanthin	Zeinoxanthin	Ref.
Saskatoon berry	*Amelanchier alnifolia Nutt.*	A0CFZ	Saskatoons	Poland	Poland				berry						285-567		78

Table S6.21.1 Other not listed other small fruits and berries (A01GB) (µg/100g)

Food name	Scientific name	FoodEx2_ TermCode	FoodEx2_ TermName	Origin (country)	Purchase (country)	Water (%)	Process	Saponification	Part analysed	Colour	α-carotene	β-carotene	β-cryptoxanthin	ζ-carotene	Antheraxanthin	Ref.
Sarsaparilla	*Smilax aspera L.*	A01GB	Other not listed other small fruits and berries	Spain	Spain				berries	red			4214±141		58±21	79
Tampoi Kuning	*Baccaurea reticulata*	A01GB	Other not listed other small fruits and berries	Malaysia					flesh/pulp fruit			10720				80
Tampoi Putih	*Baccaurea macrocarpa*	A01GB	Other not listed other small fruits and berries	Malaysia					flesh/pulp fruit			0				80

Table S6.21.2 Other not listed other small fruits and berries (A01GB) (µg/100g) (continuation)

Food name	Scientific name	FoodEx2_ TermCode	FoodEx2_ TermName	Origin (country)	Purchase (country)	Water (%)	Process	Saponification	Part analysed	Colour	Auroxanthin	E(v. trans)-α-carotene	E(v. trans)-β-carotene	E(v. trans)-β-cryptoxanthin	E(v. trans)-lutein	Ref.
Sarsaparilla	*Smilax aspera L.*	A01GB	Other not listed other small fruits and berries	Spain	Spain					red			6576±257	742±44	68±4	79

Table S6.21.3 Other not listed other small fruits and berries (A01GB) (µg/100g) (continuation)

Food name	Scientific name	FoodEx2_ TermCode	FoodEx2_ TermName	Origin (country)	Purchase (country)	Water (%)	Process	Saponification	Part analysed	Colour	E(v. trans)-lycopene	E(v. trans)-zeaxanthin	Lutein	Luteoxanthin	Lycopene	Ref.
Sarsaparilla	*Smilax aspera L.*	A01GB	Other not listed other small fruits and berries	Spain	Spain					red	24244± 3169	435±78				79

Table S6.21.4 Other not listed other small fruits and berries (A01GB) (µg/100g) (continuation)

Food name	Scientific name	FoodEx2_ TermCode	FoodEx2_ TermName	Origin (country)	Purchase (country)	Water (%)	Process	Saponification	Part analysed	Colour	Violaxanthin	Z(v. cis)-lycopene	Z(v. cis)-β-carotene	Z(v. cis)-β-cryptoxanthin	Zeaxanthin	Zeinoxanthin	Ref.
Sarsaparilla	*Smilax aspera L.*	A01GB	Other not listed other small fruits and berries	Spain	Spain					red					856±86		79

Table S6.22.1 Miscellaneous fruits with edible peel (A01HE) (µg/100g)

Food name	Scientific name	FoodEx2_ TermCode	FoodEx2_ TermName	Origin (country)	Purchase (country)	Water (%)	Process	Saponification	Part analysed	Colour	Mutatoxanthin	Neochrome	Neoxanthin	Phytoene	Phytofluene	Ref.
Caja	*Spondias lutea*	A01HE	Miscellaneous fruits with edible peel	Spain	Spain				pulp	orange				40 (20-50)		22

Table S6.23.1 Dates and similar (A0DSB) (µg/100g) (continuation)

Food name	Scientific name	FoodEx2_ TermCode	FoodEx2_ TermName	Origin (country)	Purchase (country)	Water (%)	Process	Saponification	Part analysed	Colour	E(v. trans)-lycopene	E(v. trans)-zeaxanthin	Lutein	Luteoxanthin	Lycopene	Ref.
Date	*Phoenix dactylifera L..*	A01HF	Dates	Finland	Finland				pulp	-	20					26

Table S6.24.1 Figs and similar (A0DRY) (µg/100g)

Food name	Scientific name	FoodEx2_ TermCode	FoodEx2_ TermName	Origin (country)	Purchase (country)	Water (%)	Process	Saponification	Part analysed	Colour	α-carotene	β-carotene	β-cryptoxanthin	ζ-carotene	Antheraxanthin	Ref.
Fig	*Ficus carica L*	A01HG	Figs	Italy	Italy						10	40	20			25
Fig	*Ficus carica L cv Sarilop*	A01HG	Figs	Turkey	Turkey	50-60				yellow		140	204			81
Fig	*Ficus carica L cv Sarizeybek*	A01HG	Figs	Turkey	Turkey	80				yellow		148	185			81

Table S6.24.2 Figs and similar (A0DRY) (µg/100g) (continuation)

Food name	Scientific name	FoodEx2_ TermCode	FoodEx2_ TermName	Origin (country)	Purchase (country)	Water (%)	Process	Saponification	Part analysed	Colour	E(v. trans)-lycopene	E(v. trans)-zeaxanthin	Lutein	Luteoxanthin	Lycopene	Ref.
Fig	*Ficus carica L.*	A01HG	Figs	Italy	Italy								80		320	25
Fig	*Ficus carica L. cv Sarilop*	A01HG	Figs	Turkey	Turkey	50-60				yellow			614.0			81
Fig	*Ficus carica L. cv Sarizeybek*	A01HG	Figs	Turkey	Turkey	80				yellow			715			81

Table S6.24.3 Figs and similar (A0DRY) (µg/100g) (continuation)

Food name	Scientific name	FoodEx2_ TermCode	FoodEx2_ TermName	Origin (country)	Purchase (country)	Water (%)	Process	Saponification	Part analysed	Colour	Violaxanthin	Z(v. cis)-lycopene	Z(v. cis)-β-carotene	Z(v. cis)-β-cryptoxanthin	Zeaxanthin	Zeinoxanthin	Ref.
Fig	*Ficus carica L. cv Sarilop*	A01HG	Figs	Turkey	Turkey	50-60			fresh fruit	yellow					332.0		81
Fig	*Ficus carica L. cv Sarizeybek*	A01HG	Figs	Turkey	Turkey	80			fresh fruit	yellow					220		81

Table S6.25.1 Table olives and similar (A01HH) (µg/100g)

Food name	Scientific name	FoodEx2_ TermCode	FoodEx2_ TermName	Origin (country)	Purchase (country)	Water (%)	Process	Saponification	Part analysed	Colour	α-carotene	β-carotene	β-cryptoxanthin	ζ-carotene	Antheraxanthin	Ref.
green olive with bone	*Olea europaea L.*	A01BP	Table olives	Spain	Spain					green		207	4			24

Table S6.26.1 Carambolas and similar (A01HL) (µg/100g)

Food name	Scientific name	FoodEx2_ TermCode	FoodEx2_ TermName	Origin (country)	Purchase (country)	Water (%)	Process	Saponification	Part analysed	Colour	α-carotene	β-carotene	β-cryptoxanthin	ζ-carotene	Antheraxanthin	Ref.
Jambu Mawar	*Sysygium jambos*	A01HV	Pommarosas	Malaysia					flesh/pulp fruit			0				80
Jambu Susu	*Sysygium malaccense*	A01HT	Malay pommarosas	Malaysia					flesh/pulp fruit			0				80
Kedondong	*Spondias dulcis*	A01JB	Ambarellas	Indonesia								201 (143-367)	309 (276-338)			59
Starfruit	*Averrhoa carambola*	A01HM	Carambolas	Indonesia								42 (18-81)	36 (3-60)			59

Table S6.26.2 Carambolas and similar (A01HL) (µg/100g) (continuation)

Food name	Scientific name	FoodEx2_ TermCode	FoodEx2_ TermName	Origin (country)	Purchase (country)	Water (%)	Process	Saponification	Part analysed	Colour	E(v. trans)-lycopene	E(v. trans)-zeaxanthin	Lutein	Luteoxanthin	Lycopene	Ref.
Kedondong	*Spondias dulcis*	A01JB	Ambarellas	Indonesia											364 (313-464)	59
Starfruit	*Averrhoa carambola*	A01HM	Carambolas	Indonesia											42 (16-94)	59

Table S6.27.1 Kaki and similar (A01HP) (µg/100g)

Food name	Scientific name	FoodEx2_ TermCode	FoodEx2_ TermName	Origin (country)	Purchase (country)	Water (%)	Process	Saponification	Part analysed	Colour	α-carotene	β-carotene	β-cryptoxanthin	ζ-carotene	Antheraxanthin	Ref.
Persimmon	*Diospyros kaki L.*	A01HQ	Kaki	Spain	Spain					reddish	6.3	53.5	215.6			56
Persimmon	*Diospyros kaki L.*	A01HQ	Kaki	Spain	Spain					red		253	1447			24
Persimmon	*Diospyros kaki L.*	A01HQ	Kaki	Spain	Spain					orange / red			215.6± 71.4			56
Persimmon	*Diospyros kaki L.*	A01HQ	Kaki	Spain	Spain							253	1447			24
Persimmon	*Diospyros kaki L., cv. Rojo Brillante*	A01HQ	Kaki	Spain	Spain					orange / red		49.1±6.2				82
Persimmon	*Diospyros kaki L., cv. Rojo Brillante*	A01HQ	Kaki	Spain	Spain					orange / red		52±1.4				82
Persimmon	*Diospyros kaki L., cv. Rojo Brillante*	A01HQ	Kaki	Spain	Spain					red		45.4± 13.1				82
Persimmon	*Diospyros kaki L., cv. Rojo Brillante*	A01HQ	Kaki	Spain	Spain					red		77±1.4				82
Persimmon	*Diospyrus kaki L. cv. Amankaki*	A01HQ#F20 .A0F7P	Kaki, PART-CONSUMED-ANALYSED = Only peel	Slovenia	Slovenia				skin		108.8	404.4	32.7			83
Persimmon	*Diospyrus kaki L. cv. Amankaki*	A01HQ#F20 .A0F7P$F20 .A07QF$F2 0.A07RD	Kaki, PART-CONSUMED-ANALYSED = Only peel, W/o peel, W/o seeds	Slovenia	Slovenia				pulp		72.6	39.1	28.7			83
Persimmon	*Diospyrus kaki L. cv. Cal Fuyu*	A01HQ#F20 .A0F7P	Kaki, PART-CONSUMED-ANALYSED = Only peel	Slovenia	Slovenia				skin		136.8	417.9	76.7			83

Table S6.27.1 Kaki and similar (A01HP) (µg/100g) (continuation)

Food name	Scientific name	FoodEx2_ TermCode	FoodEx2_ TermName	Origin (country)	Purchase (country)	Water (%)	Process	Saponification	Part analysed	Colour	α-carotene	β-carotene	β-cryptoxanthin	ζ-carotene	Antheraxanthin	Ref.
Persimmon	*Diospyrus kaki L. cv. Cal Fuyu*	A01HQ#F20.A0F7P$F20.A07QF$F20.A07RD	Kaki, PART-CONSUMED-ANALYSED = Only peel, W/o peel, W/o seeds	Slovenia	Slovenia				pulp		7.56	30.3	7.65			83
Persimmon	*Diospyrus kaki L. cv. Fuji*	A01HQ#F20.A0F7P	Kaki, PART-CONSUMED-ANALYSED = Only peel	Slovenia	Slovenia				skin		138.5	428.6	78.4			83
Persimmon	*Diospyrus kaki L. cv. Fuji*	A01HQ#F20.A0F7P$F20.A07QF$F20.A07RD	Kaki, PART-CONSUMED-ANALYSED = Only peel, W/o peel, W/o seeds	Slovenia	Slovenia				pulp		7.77	31.4	7.97			83
Persimmon	*Diospyrus kaki L. cv. Hana Fuyu*	A01HQ#F20.A0F7P	Kaki, PART-CONSUMED-ANALYSED = Only peel	Slovenia	Slovenia				skin		204.7	874.7	78.9			83
Persimmon	*Diospyrus kaki L. cv. Hana Fuyu*	A01HQ#F20.A0F7P$F20.A07QF$F20.A07RD	Kaki, PART-CONSUMED-ANALYSED = Only peel, W/o peel, W/o seeds	Slovenia	Slovenia				pulp		8.34	40.3	25.7			83
Persimmon	*Diospyrus kaki L. cv. Jiro*	A01HQ#F20.A0F7P	Kaki, PART-CONSUMED-ANALYSED = Only peel	Slovenia	Slovenia				skin		78.4	265.3	47.7			83
Persimmon	*Diospyrus kaki L. cv. Jiro*	A01HQ#F20.A0F7P$F20.A07QF$F20.A07RD	Kaki, PART-CONSUMED-ANALYSED = Only peel, W/o peel, W/o seeds	Slovenia	Slovenia				pulp		12.1	25.9	17.8			83
Persimmon	*Diospyrus kaki L. cv. O'Gosho*	A01HQ#F20.A0F7P	Kaki, PART-CONSUMED-ANALYSED = Only peel	Slovenia	Slovenia				skin		189.2	584.4	68.6			83

Table S6.27.1 Kaki and similar (A01HP) (µg/100g) (continuation)

Food name	Scientific name	FoodEx2_ TermCode	FoodEx2_ TermName	Origin (country)	Purchase (country)	Water (%)	Process	Saponification	Part analysed	Colour	α-carotene	β-carotene	β-cryptoxanthin	ζ-carotene	Antheraxanthin	Ref.
Persimmon	*Diospyrus kaki L. cv. O'Gosho*	A01HQ#F20.A0F7P$F20.A07QF$F20.A07RD	Kaki, PART-CONSUMED-ANALYSED = Only peel, W/o peel, W/o seeds	Slovenia	Slovenia				pulp		8.31	45.9	14.8			83
Persimmon	*Diospyrus kaki L. cv. Tenjin O'Gosho*	A01HQ#F20.A0F7P	Kaki, PART-CONSUMED-ANALYSED = Only peel	Slovenia	Slovenia				skin		197.6	678.0	125.4			83
Persimmon	*Diospyrus kaki L. cv. Tenjin O'Gosho*	A01HQ#F20.A0F7P$F20.A07QF$F20.A07RD	Kaki, PART-CONSUMED-ANALYSED = Only peel, W/o peel, W/o seeds	Slovenia	Slovenia				pulp		16.0	36.0	15.4			83
Persimmon	*Diospyrus kaki L. cv. Thiene*	A01HQ#F20.A0F7P	Kaki, PART-CONSUMED-ANALYSED = Only peel	Slovenia	Slovenia				skin		65.2	239.0	28.3			83
Persimmon	*Diospyrus kaki L. cv. Thiene*	A01HQ#F20.A0F7P$F20.A07QF$F20.A07RD	Kaki, PART-CONSUMED-ANALYSED = Only peel, W/o peel, W/o seeds	Slovenia	Slovenia				pulp		9.2	38.4	20.6			83
Persimmon	*Diospyrus kaki L. cv. Tipo*	A01HQ#F20.A0F7P	Kaki, PART-CONSUMED-ANALYSED = Only peel	Slovenia	Slovenia				skin		67.1	273.3	36.6			83
Persimmon	*Diospyrus kaki L. cv. Tipo*	A01HQ#F20.A0F7P$F20.A07QF$F20.A07RD	Kaki, PART-CONSUMED-ANALYSED = Only peel, W/o peel, W/o seeds	Slovenia	Slovenia				pulp		8.59	46.8	21.0			83
Persimmon	*Diospyrus kaki L. cv. Tone Wase*	A01HQ#F20.A0F7P	Kaki, PART-CONSUMED-ANALYSED = Only peel	Slovenia	Slovenia				skin		144.7	494.0	58.1			83

Table S6.27.1 Kaki and similar (A01HP) (µg/100g) (continuation)

Food name	Scientific name	FoodEx2_TermCode	FoodEx2_TermName	Origin (country)	Purchase (country)	Water (%)	Process	Saponification	Part analysed	Colour	α-carotene	β-carotene	β-cryptoxanthin	ζ-carotene	Antheraxanthin	Ref.
Persimmon	*Diospyrus kaki L. cv. Tone Wase*	A01HQ#F20.A0F7P$F20.A07QF$F20.A07RD	Kaki, PART-CONSUMED-ANALYSED = Only peel, W/o peel, W/o seeds	Slovenia	Slovenia				pulp		7.95	30.5	23.6			83
Persimmon	*Diospyrus kaki L. cv. Triumph*	A01HQ#F20.A0F7P	Kaki, PART-CONSUMED-ANALYSED = Only peel	Slovenia	Slovenia				skin		118.7	428.5	123.2			83
Persimmon	*Diospyrus kaki L. cv. Triumph*	A01HQ#F20.A0F7P$F20.A07QF$F20.A07RD	Kaki, PART-CONSUMED-ANALYSED = Only peel, W/o peel, W/o seeds	Slovenia	Slovenia				pulp		11.3	44.8	26.2			83

Table S6.27.2 Kaki and similar (A01HP) (µg/100g) (continuation)

Food name	Scientific name	FoodEx2_TermCode	FoodEx2_TermName	Origin (country)	Purchase (country)	Water (%)	Process	Saponification	Part analysed	Colour	Auroxanthin	E(v. trans)-α-carotene	E(v. trans)-β-carotene	E(v. trans)-β-cryptoxanthin	E(v. trans)-lutein	Ref.
Persimmon	*Diospyros kaki L.*	A01HQ	Kaki	Spain	Spain					reddish			38.9			56
Persimmon	*Diospyros kaki L.*	A01HQ	Kaki	Spain	Spain				edible part	orange / red		6.3±3.8	38.9±4.2			56

Table S6.27.3 Kaki and similar (A01HP) (µg/100g) (continuation)

Food name	Scientific name	FoodEx2_ TermCode	FoodEx2_ TermName	Origin (country)	Purchase (country)	Water (%)	Process	Saponification	Part analysed	Colour	E(v. trans)- lycopene	E(v. trans)- zeaxanthin	Lutein	Luteoxanthin	Lycopene	Ref.
Persimmon	*Diospyros kaki L., cv. Rojo Brillante*	A01HQ	Kaki	Spain	Spain				fruit	orange / red			31.5±3.1		9.1±1.6	82
Persimmon	*Diospyros kaki L., cv. Rojo Brillante*	A01HQ	Kaki	Spain	Spain				fruit	orange / red			29.8±1.4		3.5±0.04	82
Persimmon	*Diospyros kaki L., cv. Rojo Brillante*	A01HQ	Kaki	Spain	Spain				fruit	red			25.6± 10.4		43.2± 16.6	82
Persimmon	*Diospyros kaki L., cv. Rojo Brillante*	A01HQ	Kaki	Spain	Spain				fruit	red			46.4±3.5		63±19.4	82

Table S6.27.4 Kaki and similar (A01HP) (µg/100g) (continuation)

Food name	Scientific name	FoodEx2_ TermCode	FoodEx2_ TermName	Origin (country)	Purchase (country)	Water (%)	Process	Saponification	Part analysed	Colour	Violaxanthin	Z(v. cis)- lycopene	Z(v. cis)-β- carotene	Z(v. cis)-β- cryptoxanthin	Zeaxanthin	Zeinoxanthin	Ref.
Persimmon	*Diospyros kaki L.*	A01HQ	Kaki	Spain	Spain				edible part	red							24
Persimmon	*Diospyros kaki L.*	A01HQ	Kaki	Spain	Spain				fruit	red			9-cis-b-carotene 14.6				56
Persimmon	*Diospyros kaki L.*	A01HQ	Kaki	Spain	Spain				edible part	orange / red			14.6±8.1				56
Persimmon	*Diospyros kaki L.*	A01HQ	Kaki	Spain	Spain				edible part								24

Table S6.27.4 Kaki and similar (A01HP) (µg/100g) (continuation)

Food name	Scientific name	FoodEx2_ TermCode	FoodEx2_ TermName	Origin (country)	Purchase (country)	Water (%)	Process	Saponification	Part analysed	Colour	Violaxanthin	Z(v. cis)-lycopene	Z(v. cis)-β-carotene	Z(v. cis)-β-cryptoxanthin	Zeaxanthin	Zeinoxanthin	Ref.
Persimmon	*Diospyros kaki L., cv. Rojo Brillante*	A01HQ	Kaki	Spain	Spain				fruit	orange / red					12.4±1.9		82
Persimmon	*Diospyros kaki L., cv. Rojo Brillante*	A01HQ	Kaki	Spain	Spain				fruit	orange / red					15.2±1.8		82
Persimmon	*Diospyros kaki L., cv. Rojo Brillante*	A01HQ	Kaki	Spain	Spain				fruit	red					8.8±1.4		82
Persimmon	*Diospyros kaki L., cv. Rojo Brillante*	A01HQ	Kaki	Spain	Spain				fruit	red					23±3.2		82
Persimmon	*Diospyrus kaki L. cv. Amankaki*	A01HQ#F20.A0F7P	Kaki, PART-CONSUMED-ANALYSED = Only peel	Slovenia	Slovenia				skin						19.0		83
Persimmon	*Diospyrus kaki L. cv. Amankaki*	A01HQ#F20.A0F7P$F20.A07QF$F20.A07RD	Kaki, PART-CONSUMED-ANALYSED = Only peel, W/o peel, W/o seeds	Slovenia	Slovenia				pulp						5.23		83
Persimmon	*Diospyrus kaki L. cv. Cal Fuyu*	A01HQ#F20.A0F7P	Kaki, PART-CONSUMED-ANALYSED = Only peel	Slovenia	Slovenia				skin						30		83
Persimmon	*Diospyrus kaki L. cv. Cal Fuyu*	A01HQ#F20.A0F7P$F20.A07QF$F20.A07RD	Kaki, PART-CONSUMED-ANALYSED = Only peel, W/o peel, W/o seeds	Slovenia	Slovenia				pulp						3.51		83
Persimmon	*Diospyrus kaki L. cv. Fuji*	A01HQ#F20.A0F7P	Kaki, PART-CONSUMED-ANALYSED = Only peel	Slovenia	Slovenia				skin						30.0		83

Table S6.27.4 Kaki and similar (A01HP) (µg/100g) (continuation)

Food name	Scientific name	FoodEx2_ TermCode	FoodEx2_ TermName	Origin (country)	Purchase (country)	Water (%)	Process	Saponification	Part analysed	Colour	Violaxanthin	Z(v. cis)- lycopene	Z(v. cis)-β- carotene	Z(v. cis)-β- cryptoxanthin	Zeaxanthin	Zeinoxanthin	Ref.
Persimmon	*Diospyrus kaki L. cv. Fuji*	A01HQ#F20.A0F7P$F20.A07QF$F20.A07RD	Kaki, PART-CONSUMED-ANALYSED = Only peel, W/o peel, W/o seeds	Slovenia	Slovenia				pulp						3.54		83
Persimmon	*Diospyrus kaki L. cv. Hana Fuyu*	A01HQ#F20.A0F7P	Kaki, PART-CONSUMED-ANALYSED = Only peel	Slovenia	Slovenia				skin						41.5		83
Persimmon	*Diospyrus kaki L. cv. Hana Fuyu*	A01HQ#F20.A0F7P$F20.A07QF$F20.A07RD	Kaki, PART-CONSUMED-ANALYSED = Only peel, W/o peel, W/o seeds	Slovenia	Slovenia				pulp						8.81		83
Persimmon	*Diospyrus kaki L. cv. Jiro*	A01HQ#F20.A0F7P	Kaki, PART-CONSUMED-ANALYSED = Only peel	Slovenia	Slovenia				skin						16.5		83
Persimmon	*Diospyrus kaki L. cv. Jiro*	A01HQ#F20.A0F7P$F20.A07QF$F20.A07RD	Kaki, PART-CONSUMED-ANALYSED = Only peel, W/o peel, W/o seeds	Slovenia	Slovenia				pulp						4.87		83
Persimmon	*Diospyrus kaki L. cv. O'Gosho*	A01HQ#F20.A0F7P	Kaki, PART-CONSUMED-ANALYSED = Only peel	Slovenia	Slovenia				skin						43.5		83
Persimmon	*Diospyrus kaki L. cv. O'Gosho*	A01HQ#F20.A0F7P$F20.A07QF$F20.A07RD	Kaki, PART-CONSUMED-ANALYSED = Only peel, W/o peel, W/o seeds	Slovenia	Slovenia				pulp						8.24		83
Persimmon	*Diospyrus kaki L. cv. Tenjin O'Gosho*	A01HQ#F20.A0F7P	Kaki, PART-CONSUMED-ANALYSED = Only peel	Slovenia	Slovenia				skin						35.6		83

Table S6.27.4 Kaki and similar (A01HP) (µg/100g) (continuation)

Food name	Scientific name	FoodEx2_ TermCode	FoodEx2_ TermName	Origin (country)	Purchase (country)	Water (%)	Process	Saponification	Part analysed	Colour	Violaxanthin	Z(v. cis)-lycopene	Z(v. cis)-β-carotene	Z(v. cis)-β-cryptoxanthin	Zeaxanthin	Zeinoxanthin	Ref.
Persimmon	*Diospyrus kaki L. cv. Tenjin O'Gosho*	A01HQ#F20.A0F7P$F20.A07QF$F20.A07RD	Kaki, PART-CONSUMED-ANALYSED = Only peel, W/o peel, W/o seeds	Slovenia	Slovenia				pulp						5.83		83
Persimmon	*Diospyrus kaki L. cv. Thiene*	A01HQ#F20.A0F7P	Kaki, PART-CONSUMED-ANALYSED = Only peel	Slovenia	Slovenia				skin						20.0		83
Persimmon	*Diospyrus kaki L. cv. Thiene*	A01HQ#F20.A0F7P$F20.A07QF$F20.A07RD	Kaki, PART-CONSUMED-ANALYSED = W/o peel, W/o seeds	Slovenia	Slovenia				pulp						9.75		83
Persimmon	*Diospyrus kaki L. cv. Tipo*	A01HQ#F20.A0F7P	Kaki, PART-CONSUMED-ANALYSED = Only peel	Slovenia	Slovenia				skin						18.4		83
Persimmon	*Diospyrus kaki L. cv. Tipo*	A01HQ#F20.A0F7P$F20.A07QF$F20.A07RD	Kaki, PART-CONSUMED-ANALYSED = Only peel, W/o peel, W/o seeds	Slovenia	Slovenia				pulp						5.36		83
Persimmon	*Diospyrus kaki L. cv. Tone Wase*	A01HQ#F20.A0F7P	Kaki, PART-CONSUMED-ANALYSED = Only peel	Slovenia	Slovenia				skin						31.0		83
Persimmon	*Diospyrus kaki L. cv. Tone Wase*	A01HQ#F20.A0F7P$F20.A07QF$F20.A07RD	Kaki, PART-CONSUMED-ANALYSED = Only peel, W/o peel, W/o seeds	Slovenia	Slovenia				pulp						3.70		83
Persimmon	*Diospyrus kaki L. cv. Triumph*	A01HQ#F20.A0F7P	Kaki, PART-CONSUMED-ANALYSED = Only peel	Slovenia	Slovenia				skin						26.7		83
Persimmon	*Diospyrus kaki L. cv. Triumph*	A01HQ#F20.A0F7P$F20.A07QF$F20.A07RD	Kaki, PART-CONSUMED-ANALYSED = Only peel, W/o peel, W/o seeds	Slovenia	Slovenia				pulp						5.25		83

Table S6.28.1 Jambuls and similar (A04JN) (µg/100g)

Food name	Scientific name	FoodEx2_ TermCode	FoodEx2_ TermName	Origin (country)	Purchase (country)	Water (%)	Process	Saponification	Part analysed	Colour	α-carotene	β-carotene	β-cryptoxanthin	ζ-carotene	Antheraxanthin	Ref.
Camu-camu	*Myrciaria dubia*	A0DRL#F20. A07RD	Camu camus, PART-CONSUMED-ANALYSED = W/o seeds	Brazil					pulp and peel, without seeds			2423±428				84
Camu-camu	*Myrciaria dubia*	A0DRL#F20. A07RD	Camu camus, PART-CONSUMED-ANALYSED = W/o seeds	Brazil					pulp and peel, without seeds			2589±213				84
Strawberry tree	*Arbutus unedo L.*	A01FT	Arbutus berries	Spain	Spain	53.0 ± 0.1			fruit	red (external) orange (flesh)		600 (all-E)	120 (all-E)		1890 (all-E)	85
West Indian cherry	*Malpighia emarginata*	A01JC	Acerolas	Brazil								2600±400	360±70			86
West Indian cherry	*Malpighia emarginata*	A01JC	Acerolas	Brazil								2200±100	210±40			86
West Indian cherry	*Malpighia emarginata*	A01JC	Acerolas	Brazil								400±60	50±20			86
West indian cherry	*Malpighia punicifolia L. cv. Cati; cv. Olivier*	A01JC#F20. A07RD	Acerolas, PART-CONSUMED-ANALYSED = W/o seeds	Brazil					with skin, without seed	red	26	856	36			87

Table S6.28.2 Jambuls and similar (A04JN) (µg/100g) (continuation)

Food name	Scientific name	FoodEx2_ TermCode	FoodEx2_ TermName	Origin (country)	Purchase (country)	Water (%)	Process	Saponification	Part analysed	Colour	Auroxanthin	E(v. trans)-α-carotene	E(v. trans)-β-carotene	E(v. trans)-β-cryptoxanthin	E(v. trans)-lutein	Ref.
Strawberry tree	*Arbutus unedo L.*	A01FT	Arbutus berries	Spain	Spain	53.0 ± 0.1			fully mature fruit	red (external) orange (flesh)	tr.					85

Table S6.28.3 Jambuls and similar (A04JN) (µg/100g) (continuation)

Food name	Scientific name	FoodEx2_TermCode	FoodEx2_TermName	Origin (country)	Purchase (country)	Water (%)	Process	Saponification	Part analysed	Colour	E(v. trans)-lycopene	E(v. trans)-zeaxanthin	Lutein	Luteoxanthin	Lycopene	Ref.
Strawberry tree	*Arbutus unedo L.*	A01FT	Arbutus berries	Spain	Spain	53.0 ± 0.1			fruit	red (external) orange (flesh)			2050 (5,6-epoxide) 1890 (all-E) 160 (9Z) 210 (13Z)	150		85
West indian cherry	*Malpighia punicifolia L. cv. Cati; cv. Olivier*	A01JC#F20.A07RD	Acerolas, PART-CONSUMED-ANALYSED = W/o seeds	Brazil					with skin, without seed	red			64		nd	87

Table S6.28.4 Jambuls and similar (A04JN) (µg/100g) (continuation)

Food name	Scientific name	FoodEx2_TermCode	FoodEx2_TermName	Origin (country)	Purchase (country)	Water (%)	Process	Saponification	Part analysed	Colour	Mutatoxanthin	Neochrome	Neoxanthin	Phytoene	Phytofluene	Ref.
Strawberry tree	*Arbutus unedo L.*	A01FT	Arbutus berries	Spain	Spain	53.0 ± 0.1			fruit	red (external) orange (flesh)		120	2640 (all-E) 2460 (9'Z)			85
West indian cherry	*Malpighia punicifolia L. cv. Cati; cv. Olivier*	A01JC#F20.A07RD	Acerolas, PART-CONSUMED-ANALYSED = W/o seeds	Brazil	Brazil				with skin, without seed	red			5			87

Table S6.28.5 Jambuls and similar (A04JN) (µg/100g) (continuation)

Food name	Scientific name	FoodEx2_ TermCode	FoodEx2_ TermName	Origin (country)	Purchase (country)	Water (%)	Process	Saponification	Part analysed	Colour	Violaxanthin	Z(v. cis)-lycopene	Z(v. cis)-β-carotene	Z(v. cis)-β-cryptoxanthin	Zeaxanthin	Zeinoxanthin	Ref.
Strawberry tree	*Arbutus unedo L.*	A01FT	Arbutus berries	Spain	Spain	53.0 ± 0.1			fruit	red (external) orange (flesh)	12350 (all-E) 8780 (9Z)				910 (all-E)		85
West indian cherry	*Malpighia punicifolia L. cv. Cati ; cv. Olivier*	A01JC#F20. A07RD	Acerolas, PART-CONSUMED-ANALYSED = W/o seeds	Brazil	Brazil				with skin, without seed	red	1				2		87

Table S6.29.1 Miscellaneous fruits with inedible peel, small (A01JS) (µg/100g)

Food name	Scientific name	FoodEx2_ TermCode	FoodEx2_ TermName	Origin (country)	Purchase (country)	Water (%)	Process	Saponification	Part analysed	Colour	α-carotene	β-carotene	β-cryptoxanthin	ζ-carotene	Antheraxanthin	Ref.
Jentik-jentik	*Baccaurea polyneura*	A01KG	Other Miscellaneou s fruits with inedible peel, small	Malaysia					flesh/pulp fruit			17460				80
Kiwi	*Actinidia chinensis*	A01JT#F10. A0F5H	Kiwi fruits (green, red, yellow), QUALITATIVE-INFO = yellow	Spain	Spain					yellow		32				24
Kiwi	*Actinidia chinensis*	A01JT#F10. A0F5H	Kiwi fruits (green, red, yellow), QUALITATIVE-INFO = yellow	Spain	Spain					yellow		32				24
Kiwi	*Actinidia chinensis*	A0DRE#F20 .A07QF	Golden kiwifruit, PART-CONSUMED-ANALYSED = W/o peel	Spain	Spain	83			without skin	green		16±3				24

Table S6.29.1 Miscellaneous fruits with inedible peel, small (A01JS) (µg/100g) (continuation)

Food name	Scientific name	FoodEx2_ TermCode	FoodEx2_ TermName	Origin (country)	Purchase (country)	Water (%)	Process	Saponification	Part analysed	Colour	α-carotene	β-carotene	β-cryptoxanthin	ζ-carotene	Antheraxanthin	Ref.
Kiwi	*Actinidia chinensis*	A0DRF	Fuzzy kiwifruit	Spain	Spain					green		16				24
Kiwi	*Actinidia chinensis*	A0DRF	Fuzzy kiwifruit	Spain	Spain					green		16				24
Kiwi	*Actinidia deliciosa*	A0DRF	Fuzzy kiwifruit	Germany	Germany	87.2						12				53
Kiwi	*Actinidia deliciosa*	A01JT	Kiwi fruits (green, red, yellow)	USA				no			4		0			28
Kiwi	*Actinidia deliciosa cv. Hayward*	A0DRF	Fuzzy kiwifruit	South Korea						green		nd				70
Kiwi	*Actinidia deliciosa Planch cv. Hayward*	A0DRF	Fuzzy kiwifruit	Italy	Italy	82.6 ± 0.3			outer and inner pericarp and columella			110±0				88
Kiwi	*Actinidia deliciosa Planch cv. Hayward*	A0DRF	Fuzzy kiwifruit	Italy	Italy	84.9 ± 0.5			outer and inner pericarp and columella			50±0				88
Kiwi	*Actinidia deliciosa Planch cv. Hayward*	A0DRF	Fuzzy kiwifruit	Italy	Italy	86.4 ± 0.1			outer and inner pericarp and columella			50±0				88
kiwi	*Actinidia deliciosa Planch cv. Hayward*	A0DRF	Fuzzy kiwifruit	Italy	Italy	84.3 ± 0.6			outer and inner pericarp and columella			40±10				88
Kiwi	*Actinidia deliciosa Planch.) cv. Hayward*	A0DRF	Fuzzy kiwifruit	Italy	Italy	83.9 ± 0.4			outer and inner pericarp and columella			40±0				88
Kiwi	*Actinidia eriantha*	A01JT	Kiwi fruits (green, red, yellow)	Italy	Italy							<20				25

Table S6.29.1 Miscellaneous fruits with inedible peel, small (A01JS) (µg/100g) (continuation)

Food name	Scientific name	FoodEx2_ TermCode	FoodEx2_ TermName	Origin (country)	Purchase (country)	Water (%)	Process	Saponification	Part analysed	Colour	α-carotene	β-carotene	β-cryptoxanthin	ζ-carotene	Antheraxanthin	Ref.
Kiwi	*Actinidia eriantha cv. Bidan*	A01JT	Kiwi fruits (green, red, yellow)	South Korea					fruit	green		111±24				70
Passion fruit	*Passiflora edulis*	A01KC	Passionfruits	Spain	Spain						35	525	46			24
Salak	*Salaca edulis*	A01JS	Miscellaneous fruits with inedible peel, small	Indonesia					fruit			2997 (1884-4344)	nd			59

Table S6.29.2 Miscellaneous fruits with inedible peel, small (A01JS) (µg/100g) (continuation)

Food name	Scientific name	FoodEx2_ TermCode	FoodEx2_ TermName	Origin (country)	Purchase (country)	Water (%)	Process	Saponification	Part analysed	Colour	Auroxanthin	E(v. trans)-α-carotene	E(v. trans)-β-carotene	E(v. trans)-β-cryptoxanthin	E(v. trans)-lutein	Ref.
Kiwi	Actinidia deliciosa	A01JT	Kiwi fruits (green, red, yellow)	USA				no					0		171	28

Table S6.29.3 Miscellaneous fruits with inedible peel, small (A01JS) (µg/100g) (continuation)

Food name	Scientific name	FoodEx2_ TermCode	FoodEx2_ TermName	Origin (country)	Purchase (country)	Water (%)	Process	Saponification	Part analysed	Colour	E(v. trans)-lycopene	E(v. trans)-zeaxanthin	Lutein	Luteoxanthin	Lycopene	Ref.
Kiwi	*Actinidia chinensis*	A01JT#F10. A0F5H	Kiwi fruits (green, red, yellow), QUALITATIVE-INFO = yellow	Spain	Spain					yellow			41			24
Kiwi	*Actinidia chinensis*	A01JT#F10. A0F5H	Kiwi fruits (green, red, yellow), QUALITATIVE-INFO = yellow	Spain	Spain					yellow			41			24
Kiwi	*Actinidia chinensis*	A0DRE#F20 .A07QF	Golden kiwifruit, PART-CONSUMED-ANALYSED = W/o peel	Spain	Spain	83			without skin	green			96±17			24
Kiwi	*Actinidia chinensis*	A0DRF	Fuzzy kiwifruit	Spain	Spain				edible part	green			96			24
Kiwi	*Actinidia chinensis*	A0DRF	Fuzzy kiwifruit	Spain	Spain				edible part	green			96			24
Kiwi	*Actinidia deliciosa*	A0DRF	Fuzzy kiwifruit	Germany	Germany	87.2							14			53
Kiwi	*Actinidia deliciosa*	A01JT	Kiwi fruits (green, red, yellow)	USA				no				0				28
Kiwi	*Actinidia deliciosa cv. Hayward*	A0DRF	Fuzzy kiwifruit	South Korea					fruit	green			673±67			70
Kiwi	*Actinidia deliciosa Planch cv. Hayward*	A0DRF	Fuzzy kiwifruit	Italy	Italy	82.6 ± 0.3			outer and inner pericarp and columella				460±70			88
Kiwi	*Actinidia deliciosa Planch cv. Hayward*	A0DRF	Fuzzy kiwifruit	Italy	Italy	84.9 ± 0.5			outer and inner pericarp and columella				210±10			88
Kiwi	*Actinidia deliciosa Planch cv. Hayward*	A0DRF	Fuzzy kiwifruit	Italy	Italy	86.4 ± 0.1			outer and inner pericarp and columella				170±10			88

Table S6.29.3 Miscellaneous fruits with inedible peel, small (A01JS) (µg/100g) (continuation)

Food name	Scientific name	FoodEx2_TermCode	FoodEx2_TermName	Origin (country)	Purchase (country)	Water (%)	Process	Saponification	Part analysed	Colour	E(v. trans)-lycopene	E(v. trans)-zeaxanthin	Lutein	Luteoxanthin	Lycopene	Ref.
Kiwi	*Actinidia deliciosa Planch cv. Hayward*	A0DRF	Fuzzy kiwifruit	Italy	Italy	84.3 ± 0.6			outer and inner pericarp and columella				190±20			88
Kiwi	*Actinidia deliciosa Planch cv. Hayward*	A0DRF	Fuzzy kiwifruit	Italy	Italy	83.9 ± 0.4			outer and inner pericarp and columella				160±20			88
Kiwi	*Actinidia eriantha*	A01JT	Kiwi fruits (green, red, yellow)	Italy	Italy										< 10	25
Kiwi	*Actinidia eriantha cv. Bidan*	A01JT	Kiwi fruits (green, red, yellow)	South Korea						green			2415±306			70
Salak	*Salaca edulis*	A01JS	Miscellaneous fruits with inedible peel, small	Indonesia											1130 (687-1384)	59

Table S6.29.5 Miscellaneous fruits with inedible peel, small (A01JS) (µg/100g) (continuation)

Food name	Scientific name	FoodEx2_TermCode	FoodEx2_TermName	Origin (country)	Purchase (country)	Water (%)	Process	Saponification	Part analysed	Colour	Violaxanthin	Z(v. cis)-lycopene	Z(v. cis)-β-carotene	Z(v. cis)-β-cryptoxanthin	Zeaxanthin	Zeinoxanthin	Ref.
Kiwi	*Actinidia deliciosa*	A01JT	Kiwi fruits (green, red, yellow)	USA				no					31				28
Kiwi	*Actinidia deliciosa cv. Hayward*	A0DRF	Fuzzy kiwifruit	South Korea					fruit	green					nd		70
Kiwi	*Actinidia eriantha cv. Bidan*	A01JT	Kiwi fruits (green, red, yellow)	South Korea					fruit	green					nd		70

Table S6.30.1 Miscellaneous fruits with inedible peel, large (A01LA) (µg/100g)

Food name	Scientific name	FoodEx2_TermCode	FoodEx2_TermName	Origin (country)	Purchase (country)	Water (%)	Process	Saponification	Part analysed	Colour	α-carotene	β-carotene	β-cryptoxanthin	ζ-carotene	Antheraxanthin	Ref.
Bacang	*Mangifera foetida*	A01LA	Miscellaneous fruits with inedible peel, large	Malaysia					flesh/pulp fruit			0				80
Bacang	*Mangifera foetida*	A01LA	Miscellaneous fruits with inedible peel, large	Malaysia					flesh/pulp fruit			0				80
Bacang	*Mangifera foetida*	A01LA	Miscellaneous fruits with inedible peel, large	Malaysia					flesh/pulp fruit			0				80
Cerapu	*Garcinia prainiana*	A01LA	Miscellaneous fruits with inedible peel, large	Malaysia					flesh/pulp fruit			5580				80
Cerapu	*Garcinia prainiana*	A01LA	Miscellaneous fruits with inedible peel, large	Malaysia					flesh/pulp fruit			14590 (all trans)				80
Kemang	*Mangifera caesia*	A01LA	Miscellaneous fruits with inedible peel, large	Indonesia					fruit			59 (n.d.-144)	65 (nd-196)			59

Table S6.30.2 Miscellaneous fruits with inedible peel, large (A01LA) (µg/100g) (continuation)

Food name	Scientific name	FoodEx2_TermCode	FoodEx2_TermName	Origin (country)	Purchase (country)	Water (%)	Process	Saponification	Part analysed	Colour	E(v. trans)-lycopene	E(v. trans)-zeaxanthin	Lutein	Luteoxanthin	Lycopene	Ref.
Kemang	*Mangifera caesia*	A01LA	Miscellaneous fruits with inedible peel, large	Indonesia					fruit						1805 (1014-2870)	59

Table S6.31.1 Avocados and similar (A0DQP) (µg/100g)

Food name	Scientific name	FoodEx2_ TermCode	FoodEx2_ TermName	Origin (country)	Purchase (country)	Water (%)	Process	Saponification	Part analysed	Colour	α-carotene	β-carotene	β-cryptoxanthin	ζ-carotene	Antheraxanthin	Ref.
Avocado	*Persea americana, Mill.*	A01LB	Avocados	Italy	Italy						19–30	48–81	21–32			25
Avocado	*Persea americana, Mill.*	A01LB	Avocados	Germany	Germany	80					10	40	20			53
Avocado	*Persea americana, Mill.*	A01LB	Avocados	Spain	Spain					green	29	81	40			24
Avocado	*Persea americana*	A01LB	Avocados	Spain	Spain						29	81	40			24
Avocado	*Persea americana, Mill.*	A01LB#F20. A07QF$F20 .A07QK	Avocados, PART-CONSUMED-ANALYSED = W/o peel, W/o stone	Spain	Spain	66			without skin or seeds	green	29±1	81±7	40±2			24
Avocado	*Persea americana, Mill.*	A01LB#F20. A07QF$F20 .A07QK	Avocados, PART-CONSUMED-ANALYSED = W/o peel, W/o stone	Spain	Spain	66			without skin or seeds	green	20±2	69±5	30±3			24

Table S6.31.2 Avocados and similar (A0DQP) (µg/100g) (continuation)

Food name	Scientific name	FoodEx2_ TermCode	FoodEx2_ TermName	Origin (country)	Purchase (country)	Water (%)	Process	Saponification	Part analysed	Colour	E(v. trans)-lycopene	E(v. trans)-zeaxanthin	Lutein	Luteoxanthin	Lycopene	Ref.
Avocado	*Persea americana, Mill.*	A01LB	Avocados	Italy	Italy								213–361			25
Avocado	*Persea americana, Mill.*	A01LB	Avocados	Germany	Germany	80							230			53

Table S6.31.2 Avocados and similar (A0DQP) (µg/100g) (continuation)

Food name	Scientific name	FoodEx2_ TermCode	FoodEx2_ TermName	Origin (country)	Purchase (country)	Water (%)	Process	Saponification	Part analysed	Colour	E(v. trans)-lycopene	E(v. trans)-zeaxanthin	Lutein	Luteoxanthin	Lycopene	Ref.
Avocado	*Persea americana, Mill.*	A01LB	Avocados	Spain	Spain					green			314			24
Avocado	*Persea americana*	A01LB	Avocados	Spain	Spain								314			24
Avocado	*Persea americana, Mill.*	A01LB#F20. A07QF$F20 .A07QK	Avocados, PART-CONSUMED-ANALYSED = W/o peel, W/o stone	Spain	Spain	66			without skin or seeds	green			314±18			24
Avocado	*Persea americana, Mill.*	A01LB#F20. A07QF$F20 .A07QK	Avocados, PART-CONSUMED-ANALYSED = W/o peel, W/o stone	Spain	Spain	66			without skin or seeds	green			235±10			24

Table S6.31.3 Avocados and similar (A0DQP) (µg/100g) (continuation)

Food name	Scientific name	FoodEx2_ TermCode	FoodEx2_ TermName	Origin (country)	Purchase (country)	Water (%)	Process	Saponification	Part analysed	Colour	Mutatoxanthin	Neochrome	Neoxanthin	Phytoene	Phytofluene	Ref.
Avocado	*Persea americana Mill.*	A01LB	Avocados	Germany	Germany	80							7	30		53
Avocado	*Persea americana, Mill.*	A01LB	Avocados	Spain	Spain				flesh	green				30		22

Table S6.31.4 Avocados and similar (A0DQP) (μg/100g) (continuation)

Food name	Scientific name	FoodEx2_ TermCode	FoodEx2_ TermName	Origin (country)	Purchase (country)	Water (%)	Process	Saponification	Part analysed	Colour	Violaxanthin	Z(v. cis)-lycopene	Z(v. cis)-β-carotene	Z(v. cis)-β-cryptoxanthin	Zeaxanthin	Zeinoxanthin	Ref.
Avocado	*Persea americana Mill.*	A01LB	Avocados	Italy	Italy										8–18		25

Table S6.32.1 Bananas and similar (A04JS) (μg/100g)

Food name	Scientific name	FoodEx2_ TermCode	FoodEx2_ TermName	Origin (country)	Purchase (country)	Water (%)	Process	Saponification	Part analysed	Colour	α-carotene	β-carotene	β-cryptoxanthin	ζ-carotene	Antheraxanthin	Ref.
Banana	*Musa paradísica, L.*	A0DQK	Common banana - paradisiaca cv.s	Spain	Spain					yellow	63	77				24
Banana	*Musa paradísica, L.*	A0DQK	Common banana - paradisiaca cv.s	Spain	Spain	73			without skin	white	63±24	77±28				24
Banana	*Musa paradísica, L.*	A0DQG	Plantains - paradisiaca cv.s	Indonesia					fruit			97 (72-122)	3 (nd - 5)			59
Banana	*Musa paradísica, L.*	A0DQK	Common banana - paradisiaca cv.s	Australia		63-80			fruit	yellow/ orange	61-1055					89
Banana	*Musa paradísica, L.*	A0DQK	Common banana - paradisiaca cv.s	Germany	Germany	73.2					20	21	1			53
Banana	*Musa paradísica, L.*	A0DQK	Common banana - paradisiaca cv.s	Spain	Spain						63	77				24
Banana	*Musa spp.*	A04JS	Bananas and similar-	Italy	Italy						60–156	43–131	nd – 5			25

Table S6.32.2 Bananas and similar (A04JS) (µg/100g) (continuation)

Food name	Scientific name	FoodEx2_ TermCode	FoodEx2_ TermName	Origin (country)	Purchase (country)	Water (%)	Process	Saponification	Part analysed	Colour	Auroxanthin	E(v. trans)-α-carotene	E(v. trans)-β-carotene	E(v. trans)-β-cryptoxanthin	E(v. trans)-lutein	Ref.
Banana	*Musa spp. cv. Apantu*	A04JS	Bananas and similar-	Congo (Kinshasa)					fruit	yellow		3287	6387			90
Banana	*Musa spp. cv. Bira*	A04JS	Bananas and similar-	Congo (Kinshasa)					fruit	yellow		3284	6339			90
Banana	*Musa spp. cv. Bungaoisan*	A04JS	Bananas and similar-	Congo (Kinshasa)					fruit	yellow		779	857			90
Banana	*Musa spp. cv. Hung Tu*	A04JS	Bananas and similar-	Congo (Kinshasa)					fruit	yellow		1849	5653			90
Banana	*Musa spp. cv. Lahi*	A04JS	Bananas and similar-	Congo (Kinshasa)					fruit	yellow		2807	6541			90
Banana	*Musa spp. cv. Sepi*	A04JS	Bananas and similar-	Congo (Kinshasa)					fruit	yellow (ripe)		4728	5611			90
Banana	*Musa spp. cv.To'o*	A04JS	Bananas and similar-	Congo (Kinshasa)					fruit	yellow		2055	5267			90

Table S6.32.3 Bananas and similar (A04JS) (µg/100g) (continuation)

Food name	Scientific name	FoodEx2_ TermCode	FoodEx2_ TermName	Origin (country)	Purchase (country)	Water (%)	Process	Saponification	Part analysed	Colour	E(v. trans)-lycopene	E(v. trans)-zeaxanthin	Lutein	Luteoxanthin	Lycopene	Ref.
Banana	*Musa paradisiaca , L.*	A0DQK	Common banana - paradisiaca cv.s	Spain	Spain					yellow			7			24
Banana	*Musa paradisiaca , L.*	A0DQK	Common banana - paradisiaca cv.s	Spain	Spain	73			without skin	white			7±2			24
Banana	*Musa paradisíaca , L.*	A0DQG	Plantains - paradisiaca cv.s	Indonesia					fruit						114 (16-247)	59
Banana	*Musa paradisíaca , L.*	A0DQK	Common banana - paradisiaca cv.s	Australia		63-80			fruit	yellow/ orange			7-146			89

Table S6.32.3 Bananas and similar (A04JS) (µg/100g) (continuation)

Food name	Scientific name	FoodEx2_ TermCode	FoodEx2_ TermName	Origin (country)	Purchase (country)	Water (%)	Process	Saponification	Part analysed	Colour	E(v. trans)- lycopene	E(v. trans)- zeaxanthin	Lutein	Luteoxanthin	Lycopene	Ref.
Banana	*Musa paradísica, L.*	A0DQK	Common banana - paradisiaca cv.s	Germany	Germany	73.2							20			53
Banana	*Musa paradísica, L.*	A0DQK	Common banana - paradisiaca cv.s	Spain	Spain								7			24
Banana	*Musa spp.*	A04JS	Bananas and similar-	Italy	Italy								86–192		nd – 247	25
Banana	*Musa spp. cv. Apantu*	A04JS	Bananas and similar-	Congo (Kinshasa)					fruit	yellow			128.4			90
Banana	*Musa spp. cv. Bira*	A04JS	Bananas and similar-	Congo (Kinshasa)					fruit	yellow			114.9			90
Banana	*Musa spp. cv. Bungaoisan*	A04JS	Bananas and similar-	Congo (Kinshasa)					fruit	yellow			100.9			90
Banana	*Musa spp. cv. Hung Tu*	A04JS	Bananas and similar-	Congo (Kinshasa)					fruit	yellow			0			90
Banana	*Musa spp. cv. Lahi*	A04JS	Bananas and similar-	Congo (Kinshasa)					fruit	yellow			141			90
Banana	*Musa spp. cv. Sepi*	A04JS	Bananas and similar-	Congo (Kinshasa)					fruit	yellow			0			90
Banana	*Musa spp. cv. To'o*	A04JS	Bananas and similar-	Congo (Kinshasa)					fruit	yellow			22.3			90

Table S6.32.4 Bananas and similar (A04JS) (μg/100g) (continuation)

Food name	Scientific name	FoodEx2_ TermCode	FoodEx2_ TermName	Origin (country)	Purchase (country)	Water (%)	Process	Saponification	Part analysed	Colour	Mutatoxanthin	Neochrome	Neoxanthin	Phytoene	Phytofluene	Ref.
Banana	*Musa paradisiaca , L.*	A0DQK	Common banana - paradisiaca cv.s	Spain	Spain				Fruit	light yellow				30		22

Table S6.32.5 Bananas and similar (A04JS) (μg/100g) (continuation)

Food name	Scientific name	FoodEx2_ TermCode	FoodEx2_ TermName	Origin (country)	Purchase (country)	Water (%)	Process	Saponification	Part analysed	Colour	Violaxanthin	Z(v. cis)-lycopene	Z(v. cis)-β-carotene	Z(v. cis)-β-cryptoxanthin	Zeaxanthin	Zeinoxanthin	Ref.
Banana	*Musa spp cv. Apantu*	A04JS	Bananas and similar-	Congo (Kinshasa)					fruit	yellow			263.3				90
Banana	*Musa spp. cv. Bira*	A04JS	Bananas and similar-	Congo (Kinshasa)					fruit	yellow			529.7				90
Banana	*Musa spp. cv. Bungaoisan*	A04JS	Bananas and similar-	Congo (Kinshasa)					fruit	yellow			114.2				90
Banana	*Musa spp. cv. Lahi*	A04JS	Bananas and similar-	Congo (Kinshasa)					fruit	yellow			656.7				90
Banana	*Musa spp. cv. Sepi*	A04JS	Bananas and similar-	Congo (Kinshasa)					fruit	yellow (ripe)			257.6				90
Banana	*Musa spp. cv. To'o*	A04JS	Bananas and similar-	Congo (Kinshasa)					fruit	yellow			247.9				90
Banana	*Musa spp. cv.Hung Tu*	A04JS	Bananas and similar-	Congo (Kinshasa)					fruit	yellow			258.2				90

Table S6.33.1 Mangoes and similar (A0DQF) (µg/100g)

Food name	Scientific name	FoodEx2_ TermCode	FoodEx2_ TermName	Origin (country)	Purchase (country)	Water (%)	Process	Saponification	Part analysed	Colour	α-carotene	β-carotene	β-cryptoxanthin	ζ-carotene	Antheraxanthin	Ref.
Kuini	*Mangifera odorata*	A0DQF	Mangoes and similar-	Malaysia					flesh/pulp			0				80
Mango	*Magnifera indica L.*	A01LF	Mangoes	Spain	Spain				edible part	orange		152				24
Mango	*Magnifera indica L.*	A01LF	Mangoes	Costa Rica	United Kingdom				peeled and de-stoned	-		4980±980				95
Mango	*Mangifera indica L.*	A01LF	Mangoes	USA				no			14		0			28
Mango	*Mangifera indica L.*	A01LF	Mangoes	Indonesia					fruit			553 (109-1201)	137 (17-317)			59
Mango	*Mangifera indica L.*	A01LF	Mangoes	Spain	Spain							152				24
Mango	*Mangifera indica L.*	A01LF	Mangoes	Italy	Italy						nd	109–1201	17–317			25

Table S6.33.2 Mangoes and similar (A0DQF) (µg/100g) (continuation)

Food name	Scientific name	FoodEx2_ TermCode	FoodEx2_ TermName	Origin (country)	Purchase (country)	Water (%)	Process	Saponification	Part analysed	Colour	Auroxanthin	E(v. trans)-α-carotene	E(v. trans)-β-carotene	E(v. trans)-β-cryptoxanthin	E(v. trans)-lutein	Ref.
Mango	*Mangifera indica L.*	A01LF	Mangoes	Brazil	Brazil				fruit	ripe			580±250			86
Mango	*Mangifera indica L.*	A01LF	Mangoes	USA	USA			no					142		6	28
Mango	*Mangifera indica L. cv. Keitt*	A01LF	Mangoes	Brazil	Brazil				fruit	green			170±30			86

Table S6.33.2 Mangoes and similar (A0DQF) (μg/100g) (continuation)

Food name	Scientific name	FoodEx2_ TermCode	FoodEx2_ TermName	Origin (country)	Purchase (country)	Water (%)	Process	Saponification	Part analysed	Colour	Auroxanthin	E(v. trans)-α-carotene	E(v. trans)-β-carotene	E(v. trans)-β-cryptoxanthin	E(v. trans)-lutein	Ref.
Mango	*Mangifera indica L. cv. Keitt*	A01LF	Mangoes	Brazil	Brazil				fruit	ripe			1500±200			86
Mango	*Mangifera indica L. cv. Tommy Atkins*	A01LF	Mangoes	Brazil	Brazil				fruit	green			200±80			86
Mango	*Mangifera indicant L. cv. Keitt*	A01LF	Mangoes	Brazil	Brazil				fruit	ripe			670±160			86

Table S6.33.3 Mangoes and similar (A0DQF) (μg/100g) (continuation)

Food name	Scientific name	FoodEx2_ TermCode	FoodEx2_ TermName	Origin (country)	Purchase (country)	Water (%)	Process	Saponification	Part analysed	Colour	E(v. trans)-lycopene	E(v. trans)-zeaxanthin	Lutein	Luteoxanthin	Lycopene	Ref.
Mango	*Mangifera indica L.*	A01LF	Mangoes	Indonesia		na			fruit						353 (49-724)	59
Mango	*Mangifera indica L.*	A01LF	Mangoes	Italy	Italy										<10–724	25
Mango	*Mangifera indica L.*	A01LF	Mangoes	USA				no				0				28

Table S6.33.4 Mangoes and similar (A0DQF) (μg/100g) (continuation)

Food name	Scientific name	FoodEx2_ TermCode	FoodEx2_ TermName	Origin (country)	Purchase (country)	Water (%)	Process	Saponification	Part analysed	Colour	Violaxanthin	Z(v. cis)-lycopene	Z(v. cis)-β-carotene	Z(v. cis)-β-cryptoxanthin	Zeaxanthin	Zeinoxanthin	Ref.
Mango	Mangifera indica	A01LF	Mangoes	USA				no					43				28

Table S6.34.1 Papayas and similar (A0DQE) (μg/100g)

Food name	Scientific name	FoodEx2_ TermCode	FoodEx2_ TermName	Origin (country)	Purchase (country)	Water (%)	Process	Saponification	Part analysed	Colour	α-carotene	β-carotene	β-cryptoxanthin	ζ-carotene	Antheraxanthin	Ref.
Mangosteen	*Garcinia mangostana*	A01JX	Mangosteens	Indonesia					fruit			nd	44 (nd - 70			59
Papaya	*Carica papaya*	A01LG	Papayas	Indonesia					fruit			440 (322-664)	180 (n.d.-425)			59
Papaya	*Carica papaya*	A01LG	Papayas	Spain	Spain					orange		276	76			24
Papaya	*Carica papaya*	A01LG	Papayas	Spain	Spain				pulp	orange						22
Papaya	*Carica papaya L.*	A01LG	Papayas	Italy	Italy						nd	81–664	nd – 1034			25
Papaya	*Carica papaya L.*	A01LG	Papayas	Germany	Germany	85.3					50	380	80±40	170	8	53
Papaya	*Carica papaya L.*	A01LG	Papayas	Spain	Spain							276	76			24
Papaya	*Carica papaya L.*	A01LG	Papayas	Finland	Finland				pulp	-						26
Papaya	*Carica papaya L. cv. Common*	A01LG	Papayas	Brazil					fuit			120±90	810±170			86
Papaya	*Carica papaya L. cv. Formosa*	A01LG	Papayas	Brazil					fuit			140±50	530±110			86
Papaya	*Carica papaya L. cv. Formosa*	A01LG	Papayas	Brazil					fuit			610±140	860±220			86
Papaya	*Carica papaya L. cv. Tailandia*	A01LG	Papayas	Brazil					fuit			230±70	970±180			86
Papaya	*Carica papayaL. cv. Solo*	A01LG	Papayas	Brazil					fuit			250±100	910±240			86
Tamarillo	*Solanum betaceum Cav.*	A01HZ	Tamarillos	Ecuador		87.5 ± 0.3			fruit	yellow		460±30	110±10			92

Table S6.34.1 Papayas and similar (A0DQE) (µg/100g) (continuation)

Food name	Scientific name	FoodEx2_ TermCode	FoodEx2_ TermName	Origin (country)	Purchase (country)	Water (%)	Process	Saponification	Part analysed	Colour	α-carotene	β-carotene	β-cryptoxanthin	ζ-carotene	Antheraxanthin	Ref.
Tamarillo	*Solanum betaceum Cav.*	A01HZ	Tamarillos	Ecuador		87.5 ± 0.3			fruit	red		510±30	150±8			92
Tamarillo	*Solanum betaceum Cav.*	A01HZ	Tamarillos	Ecuador		87.5 ± 0.3			fruit	yellow			1350±10			92
Tamarillo	*Solanum betaceum Cav.*	A01HZ	Tamarillos	Ecuador		87.5 ± 0.3			fruit	red			1580±10			92
Tamarillo	*Solanum betaceum Cav.*	A01HZ	Tamarillos	Ecuador					peeled without seeds			79.2±1.8	69.6±8.7			93
Tamarillo	*Solanum betaceum Cav.*	A01HZ	Tamarillos	Ecuador								64.9±3	52.7±7.7			93
Tamarillo	*Solanum betaceum Cav.*	A01HZ	Tamarillos	Ecuador					peeled without seeds			61.1±5.4	52.0±9.7			93
Tamarillo	*Solanum betaceum Cav.*	A01HZ	Tamarillos	Ecuador					peeled without seeds			85.5± 11.9	79.1± 11.3			93
Tamarillo	*Solanum betaceum Cav.*	A01HZ	Tamarillos	Ecuador					peeled without seeds			70.1± 11.5	63.9±5			93
Tamarillo	*Solanum betaceum Cav.*	A01HZ	Tamarillos	Ecuador					peeled without seeds			73.4±7.5	67.5±9.4			93

Table S6.34.2 Papayas and similar (A0DQE) (µg/100g) (continuation)

Food name	Scientific name	FoodEx2_ TermCode	FoodEx2_ TermName	Origin (country)	Purchase (country)	Water (%)	Process	Saponification	Part analysed	Colour	E(v. trans)-lycopene	E(v. trans)-zeaxanthin	Lutein	Luteoxanthin	Lycopene	Ref.
Mangosteen	*Garcinia mangostana*	A01JX	Mangosteens	Indonesia					fruit						177 (nd - 409)	59
Papaya	*Carica papaya L. cv. Tailandia*	A01LG	Papayas	Brazil					fuit						4000± 600	86
Papaya	*Carica papaya L.*	A01LG	Papayas	Indonesia					fruit						5750 (4305-7564)	59
Papaya	*Carica papaya L.*	A01LG	Papayas	Italy	Italy								93–318		nd – 7564	25
Papaya	*Carica papaya L.*	A01LG	Papayas	Germany	Germany	85.3			edible part				8			53
Papaya	*Carica papaya L.*	A01LG	Papayas	Finland	Finland				pulp	-	1610					26
Papaya	*Carica papaya L. cv. Formosa*	A01LG	Papayas	Brazil					fuit						1900± 400	86
Papaya	*Carica papaya L. cv. Formosa*	A01LG	Papayas	Brazil					fuit						2600± 300	86
Papaya	*Carica papaya L. cv. Solo*	A01LG	Papayas	Brazil					fuit						2100± 1600	86
Tamarillo	*Solanum betaceum Cav.*	A01HZ	Tamarillos	Ecuador		87.5 ± 0.3			fruit	yellow			98±5			92
Tamarillo	*Solanum betaceum Cav.*	A01HZ	Tamarillos	Ecuador		87.5 ± 0.3			fruit	red			125±5			92

Table S6.34.3 Papayas and similar (A0DQE) (µg/100g) (continuation)

Food name	Scientific name	FoodEx2_ TermCode	FoodEx2_ TermName	Origin (country)	Purchase (country)	Water (%)	Process	Saponification	Part analysed	Colour	Mutatoxanthin	Neochrome	Neoxanthin	Phytoene	Phytofluene	Ref.
Papaya	*Carica papaya L.*	A01LG	Papayas	Germany	Germany	85.3								250	260	53
Papaya	*Carica papaya L.*	A01LG	Papayas	Spain	Spain				pulp	orange				250	260	22

Table S6.34.4 Papayas and similar (A0DQE) (µg/100g) (continuation)

Food name	Scientific name	FoodEx2_ TermCode	FoodEx2_ TermName	Origin (country)	Purchase (country)	Water (%)	Process	Saponification	Part analysed	Colour	Violaxanthin	Z(v. cis)-lycopene	Z(v. cis)-β-carotene	Z(v. cis)-β-cryptoxanthin	Zeaxanthin	Zeinoxanthin	Ref.
Papaya	*Carica papaya L.*	A01LG	Papayas	Germany	Germany	85.3			edible part						9		53
Tamarillo	*Solanum betaceum Cav.*	A01HZ	Tamarillos	Ecuador		87.5 ± 0.3			fruit	yellow					10±2		92
Tamarillo	*Solanum betaceum Cav.*	A01HZ	Tamarillos	Ecuador		87.5 ± 0.3			fruit	red					30±6		92
Tamarillo	*Solanum betaceum Cav.*	A01HZ	Tamarillos	Ecuador		87.5 ± 0.3			fruit	yellow					59±2		92
Tamarillo	*Solanum betaceum Cav.*	A01HZ	Tamarillos	Ecuador		87.5 ± 0.3			fruit	red					170±6		92
Tamarillo	*Solanum betaceum Cav.*	A01HZ	Tamarillos	Ecuador					peeled without seeds						86.4±5.3		93

Table S6.34.4 Papayas and similar (A0DQE) (µg/100g) (continuation)

Food name	Scientific name	FoodEx2_ TermCode	FoodEx2_ TermName	Origin (country)	Purchase (country)	Water (%)	Process	Saponification	Part analysed	Colour	Violaxanthin	Z(v. cis)-lycopene	Z(v. cis)-β-carotene	Z(v. cis)-β-cryptoxanthin	Zeaxanthin	Zeinoxanthin	Ref.
Tamarillo	*Solanum betaceum Cav.*	A01HZ	Tamarillos	Ecuador					peeled without seeds						64.1±0.8		93
Tamarillo	*Solanum betaceum Cav.*	A01HZ	Tamarillos	Ecuador					peeled without seeds						68.5±9.5		93
Tamarillo	*Solanum betaceum Cav.*	A01HZ	Tamarillos	Ecuador					peeled without seeds						89.1±5.2		93
Tamarillo	*Solanum betaceum Cav.*	A01HZ	Tamarillos	Ecuador					peeled without seeds						67.4±4.2		93

Table S6.35.1 Cherimoyas and similar (A04JT) (µg/100g)

Food name	Scientific name	FoodEx2_ TermCode	FoodEx2_ TermName	Origin (country)	Purchase (country)	Water (%)	Process	Saponification	Part analysed	Colour	α-carotene	β-carotene	β-cryptoxanthin	ζ-carotene	Antheraxanthin	Ref.
Rambutan	*Nephelium lappaceum*	A01KA	Rambutans	Indonesia					fruit			nd	nd			59
Sawo	*Manilkara zapota*	A01KT	Sapodillas	Indonesia					fruit			350 (90-505)	119 (17-174)			59

Table S6.35.2 Cherimoyas and similar (A04JT) (µg/100g) (continuation)

Food name	Scientific name	FoodEx2_ TermCode	FoodEx2_ TermName	Origin (country)	Purchase (country)	Water (%)	Process	Saponification	Part analysed	Colour	E(v. trans)-lycopene	E(v. trans)-zeaxanthin	Lutein	Luteoxanthin	Lycopene	Ref.
Rambutan	*Nephelium lappaceum*	A01KA	Rambutans	Indonesia					fruit						148 (nd - 277)	59
Sawo	*Manilkara zapota*	A01KT	Sapodillas	Indonesia					fruit						1386 (469-2447)	59

Table S6.36.1 Guavas and similar (A01LN) (µg/100g)

Food name	Scientific name	FoodEx2_ TermCode	FoodEx2_ TermName	Origin (country)	Purchase (country)	Water (%)	Process	Saponification	Part analysed	Colour	α-carotene	β-carotene	β-cryptoxanthin	ζ-carotene	Antheraxanthin	Ref.
Guava	*Psidium guajava L.*	A0CGD	Guavas	Indonesia					fruit			984 (102-2669)	66 (19-118)			59
Guava	*Psidium guajava L.*	A0CGD	Guavas	Italy	Italy						nd	102–2669	19–118			25
Guava	*Psidium guajava L.*	A0CGD	Guavas	Finland	Finland				pulp	-						26

Table S6.36.2 Guavas and similar (A01LN) (µg/100g) (continuation)

Food name	Scientific name	FoodEx2_ TermCode	FoodEx2_ TermName	Origin (country)	Purchase (country)	Water (%)	Process	Saponification	Part analysed	Colour	E(v. trans)-lycopene	E(v. trans)-zeaxanthin	Lutein	Luteoxanthin	Lycopene	Ref.
Guava	*Psidium guajava L.*	A0CGD	Guavas	Indonesia		na			fruit						1150 (769-1816)	59
Guava	*Psidium guajava L.*	A0CGD	Guavas	Italy	Italy										769–1816	25
Guava	*Psidium guajava L.*	A0CGD	Guavas	Finland	Finland				pulp	-	0					26

Table S6.37.1 Pineapples and similar (A0DPV) (µg/100g)

Food name	Scientific name	FoodEx2_ TermCode	FoodEx2_ TermName	Origin (country)	Purchase (country)	Water (%)	Process	Saponification	Part analysed	Colour	α-carotene	β-carotene	β-cryptoxanthin	ζ-carotene	Antheraxanthin	Ref.
Pineapple	*Ananas comosus L.*	A01LP	Pineapples	Indonesia					fruit			230 (139-347)	89 (70-124)			59
Pineapple	*Ananas comosus L.*	A01LP	Pineapples	Italy	Italy						nd	139–347	70–124			25
Pineapple	*Ananas comosus L.*	A01LP#F20. A07QF	Pineapples, PART-CONSUMED-ANALYSED = W/o peel	Spain	Spain				edible part	yellow		57				24
Pineapple	*Ananas comosus L.*	A01LP#F20. A07QF	Pineapples, PART-CONSUMED-ANALYSED = W/o peel	Spain	Spain				edible part	yellow	1	18	2			24
Pineapple	*Ananas comosus L.*	A01LP#F20. A07QF	Pineapples, PART-CONSUMED-ANALYSED = W/o peel	Spain	Spain				edible part			57				24

Table S6.37.2 Pineapples and similar (A0DPV) (µg/100g) (continuation)

Food name	Scientific name	FoodEx2_ TermCode	FoodEx2_ TermName	Origin (country)	Purchase (country)	Water (%)	Process	Saponification	Part analysed	Colour	E(v. trans)-lycopene	E(v. trans)-zeaxanthin	Lutein	Luteoxanthin	Lycopene	Ref.
Pineapple	*Ananas comosus L.*	A01LP	Pineapples	Indonesia					fruit						399(265-605)	59
Pineapple	*Ananas comosus L.*	A01LP	Pineapples	Italy	Italy										265–605	25

Table S6.38.1 Breadfruits and similar (A04JV) (µg/100g)

Food name	Scientific name	FoodEx2_ TermCode	FoodEx2_ TermName	Origin (country)	Purchase (country)	Water (%)	Process	Saponification	Part analysed	Colour	α-carotene	β-carotene	β-cryptoxanthin	ζ-carotene	Antheraxanthin	Ref.
Jackfruit	*Artocarpus heterophyllus*	A01LR	Jackfruits	Indonesia					fruit			360 (40-772)	36 (nd - 84			59

Table S6.38.2 Breadfruits and similar (A04JV) (µg/100g) (continuation)

Food name	Scientific name	FoodEx2_ TermCode	FoodEx2_ TermName	Origin (country)	Purchase (country)	Water (%)	Process	Saponification	Part analysed	Colour	E(v. trans)-lycopene	E(v. trans)-zeaxanthin	Lutein	Luteoxanthin	Lycopene	Ref.
Jackfruit	*Artocarpus heterophyllus*	A01LR	Jackfruits	Indonesia					fruit						37 (nd - 111)	59

Table S6.39.1 Durians and similar (A0DPR) (µg/100g)

Food name	Scientific name	FoodEx2_ TermCode	FoodEx2_ TermName	Origin (country)	Purchase (country)	Water (%)	Process	Saponification	Part analysed	Colour	α-carotene	β-carotene	β-cryptoxanthin	ζ-carotene	Antheraxanthin	Ref.
Durian	*Durio lowianus cv. Daun*	A01LS	Durians	Malaysia					flesh/pulp			0				80
Durian	*Durio kutejensis cv. Nyekak*	A01LS	Durians	Malaysia					flesh/pulp			7570				80
Durian	*Durio kutejensis cv. Nyekak*	A01LS	Durians	Malaysia					flesh/pulp			10990				80

Table S6.40.1 Dried fruit (A01MA) (µg/100g)

Food name	Scientific name	FoodEx2_ TermCode	FoodEx2_ TermName	Origin (country)	Purchase (country)	Water (%)	Process	Saponification	Part analysed	Colour	α-carotene	β-carotene	β-cryptoxanthin	ζ-carotene	Antheraxanthin	Ref.
Apricot	*Prunus armeniaca L.*	A01MD	Dried apricots	Spain	Spain		dried		edible part	orange	9	827	38			24
Apricot	*Prunus armeniaca L.*	A01MD	Dried apricots	USA			dried	no			436		51			28
Apricot	*Prunus armeniaca, L.*	A01MD	Dried apricots	Spain	Spain				edible part	orange	9	827	38			24

Table S6.40.2 Dried fruit (A01MA) (µg/100g) (continuation)

Food name	Scientific name	FoodEx2_ TermCode	FoodEx2_ TermName	Origin (country)	Purchase (country)	Water (%)	Process	Saponification	Part analysed	Colour	Auroxanthin	E(v. trans)-α-carotene	E(v. trans)-β-carotene	E(v. trans)-β-cryptoxanthin	E(v. trans)-lutein	Ref.
Apricot	*Prunus armeniaca L.*	A01MD	Dried apricots	USA			dried	no					2249		0	28
Rosehip	*Rosa canina L.*	A01MA#F27.A0DSS$F03.A06JD	Dried fruit, SOURCE-COMMODITIES = Dog rose, PHYSICAL-STATE = Powder	Germany	Germany		Powder		powder				5100± 500	500±20	400±20	77

Table S6.40.3 Dried fruit (A01MA) (µg/100g) (continuation)

Food name	Scientific name	FoodEx2_TermCode	FoodEx2_TermName	Origin (country)	Purchase (country)	Water (%)	Process	Saponification	Part analysed	Colour	E(v. trans)-lycopene	E(v. trans)-zeaxanthin	Lutein	Luteoxanthin	Lycopene	Ref.
Apricot	*Prunus armeniaca*	A01MD	Dried apricots	USA			dried	no				0				28
Rosehip	*Rosa canina L.*	A01MA#F27.A0DSS$F03.A06JD	Dried fruit, SOURCE-COMMODITIES = Dog rose, PHYSICAL-STATE = Powder	Germany	Germany		powder				4700± 500	1200± 100				77

Table S6.40.4 Dried fruit (A01MA) (µg/100g) (continuation)

Food name	Scientific name	FoodEx2_TermCode	FoodEx2_TermName	Origin (country)	Purchase (country)	Water (%)	Process	Saponification	Part analysed	Colour	Violaxanthin	Z(v. cis)-lycopene	Z(v. cis)-β-carotene	Z(v. cis)-β-cryptoxanthin	Zeaxanthin	Zeinoxanthin	Ref.
Apricot	*Prunus armeniaca L.*	A01MD	Dried apricots	USA			dried	no					185				28
Rosehip	*Rosa canina L.*	A01MA#F27.A0DSS$F03.A06JD	Dried fruit, SOURCE-COMMODITIES = Dog rose, PHYSICAL-STATE = Powder	Germany	Germany		powder		powder				820±70				77

Table S6.41.1 Other processed fruit products (excluding beverages) (A01QD) (µg/100g)

Food name	Scientific name	FoodEx2_ TermCode	FoodEx2_ TermName	Origin (country)	Purchase (country)	Water (%)	Process	Saponification	Part analysed	Colour	α-carotene	β-carotene	β-cryptoxanthin	ζ-carotene	Antheraxanthin	Ref.
Fruit salad		A01PC#F06. A06XY	Canned or jarred mixed fruit, SURROUNDING-MEDIUM = In concentrated Sweet liquid-syrup	Spain	Spain		in syrup					138	52			24
Fruit salad		A01PC#F06. A169N	Canned or jarred mixed fruit, SURROUNDING-MEDIUM = In own juice	Spain	Spain		in its juice					20	50			24
Olives	*Olea europaea, L.*	A01NN#F27 .A01BP$F06 .A06YD	Canned or jarred fruit, SOURCE-COMMODITIES = Table olives, SURROUNDING-MEDIUM = In vinegar	Spain	Spain		canned with vinegar			green		207	4			24
Peach	*Prunus pérsica L.*	A01NY	Canned or jarred peach	USA			canned	no			0		48			28
Peach	*Prunus pérsica L. cv Miraflores*	A01QJ#F27. A01GM$F2 2.A07SH	Fruit or fruit-vegetable puree, SOURCE-COMMODITIES = Common peaches, PREPARATION-PRODUCTION-PLACE = Food industry prepared	Spain	Spain	80 - 90	puree		commercial puree			226.5± 22.2	512.0 (all-E)±56.5	78.4± 11.4		39
Peach	*Prunus pérsica L. cv Miraflores*	A01QJ#F27. A01GM	Fruit or fruit-vegetable puree, SOURCE-COMMODITIES = Common peaches	Spain	Spain	80 - 90	puree		freshly blended puree			307.8± 25.3	635.67 (all-E)±79.4	109.8± 29.6	162.1 (all-E)±22.1	39
Peach	*Prunus persica cv Spring Lady*	A01QJ#F27. A01GM$F2 2.A07SH	Fruit or fruit-vegetable puree, SOURCE-COMMODITIES = Common peaches, PREPARATION-PRODUCTION-PLACE = Food industry prepared	Spain	Spain	80 - 90	puree		commercial puree			153.6±12	166.4 (all-E)±36.5	21.4±12		39

Table S6.41.1 Other processed fruit products (excluding beverages) (A01QD) (µg/100g) (continuation)

Food name	Scientific name	FoodEx2_ TermCode	FoodEx2_ TermName	Origin (country)	Purchase (country)	Water (%)	Process	Saponification	Part analysed	Colour	α-carotene	β-carotene	β-cryptoxanthin	ζ-carotene	Antheraxanthin	Ref.
Peach	*Prunus persica cv Spring Lady*	A01QJ#F27. A01GM	Fruit or fruit-vegetable puree, SOURCE-COMMODITIES = Common peaches	Spain	Spain	80 - 90	puree		freshly blended puree			165.0± 25.4	145.9 (all-E)±28.8	19.6± 2.4	84.2 (all-E)±11.8	39
Pineapple	*Ananas sativus*	A01PB#F06. A169N	Canned or jarred pineapple, SURROUNDING-MEDIUM = In own juice	Spain	Spain		Canned in its juice				1	18	2			24
Rosehip	*Rosa canina L.*	A01QJ#F27. A0DSS	Fruit or fruit-vegetable puree, SOURCE-COMMODITIES = Dog rose	Germany	Germany		puree		puree							77
Rosehip	*Rosa canina L.*	A01QJ#F27. A0DSS	Fruit or fruit-vegetable puree, SOURCE-COMMODITIES = Dog rose	Finland	Finland		paste		-	-						26
Rosehip	*Rosa canina L.*	A01QJ#F27. A0DSS	Fruit or fruit-vegetable puree, SOURCE-COMMODITIES = Dog rose	Germany	Germany		puree		puree							77

Table S6.41.2 Other processed fruit products (excluding beverages) (A01QD) (µg/100g) (continuation)

Food name	Scientific name	FoodEx2_TermCode	FoodEx2_TermName	Origin (country)	Purchase (country)	Water (%)	Process	Saponification	Part analysed	Colour	Auroxanthin	E(v. trans)-α-carotene	E(v. trans)-β-carotene	E(v. trans)-β-cryptoxanthin	E(v. trans)-lutein	Ref.
Peach	*Prunus pérsica L.*	A01NY	Canned or jarred peach	USA			canned	no					0		0	28
Peach	*Prunus pérsica L. cv Miraflores*	A01QJ#F27. A01GM$F22.A07SH	Fruit or fruit-vegetable puree, SOURCE-COMMODITIES = Common peaches, PREPARATION-PRODUCTION-PLACE = Food industry prepared	Spain	Spain	80 - 90	puree		commercial puree		112.0 ± 10.2 (A) 80.5 ± 5.8 (B) 88.6 ± 7.4 (C)					39
Peach	*Prunus persica cv Spring Lady*	A01QJ#F27. A01GM$F22.A07SH	Fruit or fruit-vegetable puree, SOURCE-COMMODITIES = Common peaches, PREPARATION-PRODUCTION-PLACE = Food industry prepared	Spain	Spain	80 - 90	puree		commercial puree		84.7 ± 11.2 (A) 60.9 ± 6.6 (B) 67.8 ± 6.5 (C)					39
Rosehip	*Rosa canina L.*	A01QJ#F27. A0DSS	Fruit or fruit-vegetable puree, SOURCE-COMMODITIES = Dog rose	Germany	Germany		puree		puree				1900±200	60±0	300±40	57
Rosehip	*Rosa canina L.*	A01QJ#F27. A0DSS	Fruit or fruit-vegetable puree, SOURCE-COMMODITIES = Dog rose	Germany	Germany		puree		puree				1900±200	60±0	300±40	77

Table S6.41.3 Other processed fruit products (excluding beverages) (A01QD) (µg/100g) (continuation)

Food name	Scientific name	FoodEx2_ TermCode	FoodEx2_ TermName	Origin (country)	Purchase (country)	Water (%)	Process	Saponification	Part analysed	Colour	E(v. trans)- lycopene	E(v. trans)- zeaxanthin	Lutein	Luteoxanthin	Lycopene	Ref.
Peach	*Prunus pérsica L.*	A01NY	Canned or jarred peach	USA			canned	no				8				28
Peach	*Prunus pérsica L. cv Miraflores*	A01QJ#F27. A01GM$F2 2.A07SH	Fruit or fruit-vegetable puree, SOURCE-COMMODITIES = Common peaches, PREPARATION-PRODUCTION-PLACE = Food industry prepared	Spain	Spain	80 - 90	puree		commercial puree				55.2±3.6			39
Peach	*Prunus pérsica L. cv Miraflores*	A01QJ#F27. A01GM	Fruit or fruit-vegetable puree, SOURCE-COMMODITIES = Common peaches	Spain	Spain	80 - 90	puree		freshly blended puree				83.9 (all-E)±7.6			39
Peach	*Prunus persica cv Spring Lady*	A01QJ#F27. A01GM$F2 2.A07SH	Fruit or fruit-vegetable puree, SOURCE-COMMODITIES = Common peaches, PREPARATION-PRODUCTION-PLACE = Food industry prepared	Spain	Spain	80 - 90	puree		commercial puree				42.0±2.3			39
Peach	*Prunus persica cv Spring Lady*	A01QJ#F27. A01GM	Fruit or fruit-vegetable puree, SOURCE-COMMODITIES = Common peaches	Spain	Spain	80 - 90	puree		freshly blended puree				49.9 (all-E)±3.7			39
Rosehip	*Rosa canina L.*	A01QJ#F27. A0DSS	Fruit or fruit-vegetable puree, SOURCE-COMMODITIES = Dog rose	Germany	Germany		puree		puree		4800±200	300±100				77
Rosehip	*Rosa canina L.*	A01QJ#F27. A0DSS	Fruit or fruit-vegetable puree, SOURCE-COMMODITIES = Dog rose	Germany	Germany		puree		puree		4800±200	300±100				77
Rosehip	*Rosa canina L.*	A01QJ#F27. A0DSS	Fruit or fruit-vegetable puree, SOURCE-COMMODITIES = Dog rose	Finland	Finland		paste		-	-	290					26

Table S6.41.4 Other processed fruit products (excluding beverages) (A01QD) (µg/100g) (continuation)

Food name	Scientific name	FoodEx2_ TermCode	FoodEx2_ TermName	Origin (country)	Purchase (country)	Water (%)	Process	Saponification	Part analysed	Colour	Mutatoxanthin	Neochrome	Neoxanthin	Phytoene	Phytofluene	Ref.
Peach	*Prunus persica cv Miraflores*	A01QJ#F27. A01GM$F22.A07SH	Fruit or fruit-vegetable puree, SOURCE-COMMODITIES = Common peaches, PREPARATION-PRODUCTION-PLACE = Food industry prepared	Spain	Spain	80 - 90	puree		commercial puree		69.7 ± 5.0 (A) 56.5 ± 4.3 (B)			96.2±8.2	34.0±0.9	39
Peach	*Prunus persica cv Miraflores*	A01QJ#F27. A01GM	Fruit or fruit-vegetable puree, SOURCE-COMMODITIES = Common peaches	Spain	Spain	80 - 90	puree		freshly blended puree				703.8 ± 57.4 (all-E) 54.8 ± 4.0 (9Z)	119.07±18.8	47.8±4.9	39
Peach	*Prunus persica cv Spring Lady*	A01QJ#F27. A01GM$F22.A07SH	Fruit or fruit-vegetable puree, SOURCE-COMMODITIES = Common peaches, PREPARATION-PRODUCTION-PLACE = Food industry prepared	Spain	Spain	80 - 90	puree		commercial puree		50.3 ± 3.9 (A) 41.2 ± 4.1 (B)			52.4±4.8	19.6±0.9	39
Peach	*Prunus persica cv Spring Lady*	A01QJ#F27. A01GM	Fruit or fruit-vegetable puree, SOURCE-COMMODITIES = Common peaches	Spain	Spain	80 - 90	puree		freshly blended puree				53.7 ± 2.0 (all-E) 42.4 ± 2.0 (9'Z)	39.9±7.7	17.7±1.1	39

Table S6.41.5 Other processed fruit products (excluding beverages) (A01QD) (µg/100g) (continuation)

Food name	Scientific name	FoodEx2_ TermCode	FoodEx2_ TermName	Origin (country)	Purchase (country)	Water (%)	Process	Saponification	Part analysed	Colour	Violaxanthin	Z(v. cis)- lycopene	Z(v. cis)-β- carotene	Z(v. cis)-β- cryptoxanthin	Zeaxanthin	Zeinoxanthin	Ref.
Peach	*Prunus persica*	A01NY	Canned or jarred peach	USA			canned	no					0				28
Peach	*Prunus persica cv Miraflores*	A01QJ#F27. A01GM$F2 2.A07SH	Fruit or fruit-vegetable puree, SOURCE-COMMODITIES = Common peaches, PREPARATION-PRODUCTION-PLACE = Food industry prepared	Spain	Spain	80 - 90	puree		commer cial puree						87.6 (all-E)±10.6		39
Peach	*Prunus persica cv Miraflores*	A01QJ#F27. A01GM	Fruit or fruit-vegetable puree, SOURCE-COMMODITIE S = Common peaches	Spain	Spain	80 - 90	puree		freshly blended puree		703.8 ± 57.4 (all-E) 190.4 ± 32.3 (9Z)				105.5 (all-E)±29.7		39
Peach	*Prunus persica cv Spring Lady*	A01QJ#F27. A01GM$F2 2.A07SH	Fruit or fruit-vegetable puree, SOURCE-COMMODITIES = Common peaches, PREPARATION-PRODUCTION-PLACE = Food industry prepared	Spain	Spain	80 - 90	puree		commer cial puree						47.3 (all-E)±3.2		39
Peach	*Prunus persica cv Spring Lady*	A01QJ#F27. A01GM	Fruit or fruit-vegetable puree, SOURCE-COMMODITIE S = Common peaches	Spain	Spain	80 - 90	puree		freshly blended puree		412.7 ± 54.4 (all-E) 181.5 ± 29.3 (9Z)				43.5 (all-E)±5,6		39
Rosehip	*Rosa canina L.*	A01QJ#F27. A0DSS	Fruit or fruit-vegetable puree, SOURCE-COMMODITIE S = Dog rose	Germany	Germany		puree		puree				610±50		.		77
Rosehip	*Rosa canina L.*	A01QJ#F27. A0DSS	Fruit or fruit-vegetable puree, SOURCE-COMMODITIES = Dog rose	Germany	Germany		puree		puree				610±50				77

Table S7. Legumes, nuts, oilseeds and spices (A011X)

Table S7.1.1 Legumes fresh seeds (beans, peas etc.) (A011Y) (µg/100g)

Food name	Scientific name	FoodEx2_ TermCode	FoodEx2_ TermName	Origin (country)	Purchase (country)	Water (%)	Saponification	Part analysed	Colour	α-carotene	β-carotene	E(v. trans)-β-carotene	E(v. trans)-lutein	Lutein	Ref.
Broad bean	*Vicia faba L.*	A012A	Broad beans (without pods)	Spain	Spain			edible part	green		406				24
Broad bean	*Vicia faba L.*	A012A	Broad beans (without pods)	Spain	Spain			edible part	green		406				24
Pea	*Pisum sativum L.*	A012J	Garden peas (without pods)	Italy	Italy				green	nd	520			1910	25
Pea	*Pisum sativum L.*	A012J	Garden peas (without pods)	Spain	Spain			edible part	green		548				24
Pea	*Pisum sativum L.*	A012J	Garden peas (without pods)	Spain	Spain			edible part	green	33	320				24
Pea	*Pisum sativum L.*	A012J	Garden peas (without pods)	Spain	Spain			edible part	green		320				24
Pea	*Pisum sativum L.*	A012J	Garden peas (without pods)	Spain	Spain			edible part	green		548				24
Pea	*Pisum sativum L.*	A012J	Garden peas (without pods)	Spain	Spain			edible part	green	33	320				24
Pea	*Pisum sativum L.*	A012J	Garden peas (without pods)	Spain	Spain			edible part	green		320				24

Table S7.2.1 Pulses (dried legume seeds) (A012R) (µg/100g)

Food name	Scientific name	FoodEx2_ TermCode	FoodEx2_ TermName	Origin (country)	Purchase (country)	Water (%)	Saponification	Part analysed	Colour	α-carotene	β-carotene	E(v. trans)-β-carotene	E(v. trans)-lutein	Lutein	Ref.
Lima beans	*Phaseolus lunatus L.*	A013A#F28.A0BA1$F28.A07MS	Lima beans (dry), PROCESS = Cooking and similar thermal preparation processes, Soaking	USA			no			0		76	155		28

Table S7.2.2 Pulses (dried legume seeds) (A012R) (µg/100g) (continuation)

Food name	Scientific name	FoodEx2_ TermCode	FoodEx2_ TermName	Origin (country)	Purchase (country)	Water (%)	Saponification	Part analysed	Colour	Neoxanthin	Violaxanthin	Z(v. cis)-β-carotene	Zeaxanthin	Ref.
Lima beans	*Phaseolus lunatus L.*	A013A#F28.A0BA1$F28.A07MS	Lima beans (dry), PROCESS = Cooking and similar thermal preparation processes, PROCESS = Soaking	USA			no					11		28

Table S7.3.1 Tree nuts (A014C) (µg/100g)

Food name	Scientific name	FoodEx2_ TermCode	FoodEx2_ TermName	Origin (country)	Purchase (country)	Water (%)	Saponification	Part analysed	Colour	α-carotene	β-carotene	E(v. trans)-β-carotene	E(v. trans)-lutein	Lutein	Ref.
Pistachio	*Pistacia vera L.*	A014Q	Pistachios	Italy	Italy						nd – 510			770–4900	25

Table S7.3.1 Tree nuts (A014C) (µg/100g) (continuation)

Food name	Scientific name	FoodEx2_ TermCode	FoodEx2_ TermName	Origin (country)	Purchase (country)	Water (%)	Saponification	Part analysed	Colour	α-carotene	β-carotene	E(v. trans)-β-carotene	E(v. trans)-lutein	Lutein	Ref.
Pistachio	*Pistacia vera L.*	A014Q#F20 .A07QZ	Pistachios, PART-CONSUMED-ANALYSED = W/o husks	Greece	Austria			edible part			400			4400	94
Pistachio	*Pistacia vera L.*	A014Q#F20 .A07QZ	Pistachios, PART-CONSUMED-ANALYSED = W/o husks	USA			no			0		0	1405		28

Table S7.4.1 Oilseeds (A015F) (µg/100g)

Food name	Scientific name	FoodEx2_ TermCode	FoodEx2_ TermName	Origin (country)	Purchase (country)	Water (%)	Saponification	Part analysed	Colour	α-carotene	β-carotene	E(v. trans)-β-carotene	E(v. trans)-lutein	Lutein	Ref.
Seeds		A015F	Oilseeds	United Kingdom	United Kingdom			seeds			100			480	95

Table S7.4.2 Oilseeds (A015F) (µg/100g) (continuation)

Food name	Scientific name	FoodEx2_ TermCode	FoodEx2_ TermName	Origin (country)	Purchase (country)	Water (%)	Saponification	Part analysed	Colour	Neoxanthin	Violaxanthin	Z(v. cis)-β-carotene	Zeaxanthin	Ref.
Seeds		A015F	Oilseeds	United Kingdom	United Kingdom			seeds					80	95

Table S7.5.1 Oil fruits (A016L) (µg/100g)

Food name	Scientific name	FoodEx2_TermCode	FoodEx2_TermName	Origin (country)	Purchase (country)	Water (%)	Saponification	Part analysed	Colour	α-carotene	β-carotene	E(v. trans)-β-carotene	E(v. trans)-lutein	Lutein	Ref.
Olive	*Olea europaea L*	A016M	Olives for oil production	Iran					green					250 - 467	96
Olive	*Olea europaea L.*	A016M	Olives for oil production	USA			no		green	0		100	79		28

Table S7.5.2 Oil fruits (A016L) (µg/100g) (continuation)

Food name	Scientific name	FoodEx2_TermCode	FoodEx2_TermName	Origin (country)	Purchase (country)	Water (%)	Saponification	Part analysed	Colour	Neoxanthin	Violaxanthin	Z(v. cis)-β-carotene	Zeaxanthin	Ref.
Olive	*Olea europaea L.*	A016M	Olives for oil production	USA			no		green			0		28

Table S7.6.1 Fruit spices (A018Q) (µg/100g)

Food name	Scientific name	FoodEx2_TermCode	FoodEx2_TermName	Origin (country)	Purchase (country)	Water (%)	Saponification	Part analysed	Colour	α-carotene	β-carotene	E(v. trans)-β-carotene	E(v. trans)-lutein	Lutein	Ref.
Tamarind	*Tamarindus indica*	A019J	Tamarind	Spain	Spain			edible part	orange		8				24
Tamarind	*Tamarindus indica*	A019J	Tamarind	Spain	Spain			edible part	brown		8				24

Table S7.7.1 bud spices (A01AK) (µg/100g)

Food name	Scientific name	FoodEx2_ TermCode	FoodEx2_ TermName	Origin (country)	Purchase (country)	Water (%)	Saponification	Part analysed	Colour	α-carotene	β-carotene	E(v. trans)-β-carotene	E(v. trans)-lutein	Lutein	Ref.
Capers	*Capparis spinosa*	A01AM	Capers buds	Pakistan	Spain			edible part	green		83				24
Capers	*Capparis spinosa*	A01AM	Capers buds	Spain	Spain			edible part	green, dark green		83				24

Table S7.8.1 Flowers or part of flower used as spices or similar (A0F0H) (µg/100g)

Food name	Scientific name	FoodEx2_ TermCode	FoodEx2_ TermName	Origin (country)	Purchase (country)	Water (%)	Saponification	Part analysed	Colour	α-carotene	β-carotene	E(v. trans)-β-carotene	E(v. trans)-lutein	Lutein	Ref.
.	*Humulus lupulus*	A00YZ	Hops	Spain	Spain	84.9		young shoots	green		3.76 (1.56-12.63)			5.49 (1.88-17.20)	97

Table S7.8.2 Flowers or part of flower used as spices or similar (A0F0H) (µg/100g) (continuation)

Food name	Scientific name	FoodEx2_ TermCode	FoodEx2_ TermName	Origin (country)	Purchase (country)	Water (%)	Saponification	Part analysed	Colour	Neoxanthin	Violaxanthin	Z(v. cis)-β-carotene	Zeaxanthin	Ref.
Hops	*Humulus lupulus*	A00YZ	Hops	Spain	Spain	84.9		young shoots	green	7.23 (1.32-15.70)	2.08 (0.41-6.73)			97

Table S8. Starchy roots or tubers and products thereof, sugar plants (A00ZR)

Table S8.1.1 Potatoes and similar (A0DPP) (µg/100g)

Food name	Scientific name	FoodEx2_ TermCode	FoodEx2_ TermName	Origin (country)	Purchase (country)	Water (%)	Part analysed	Colour	α-carotene	β-carotene	β-cryptoxanthin	ζ-carotene	Ref.
Potato	*Solanum andigena var. jesus*	A00ZT	Potatoes	Spain	Spain		flesh	purple and white		4.5±0.3			98
Potato	*Solanum andigena var. kasta*	A00ZT	Potatoes	Spain	Spain		flesh	purple and white		9.7±0.4			98
Potato	*Solanum andigena var. marfona*	A00ZT	Potatoes	Spain	Spain		flesh	yellow		3.8±0.1			98
Potato	*Solanum andigena var. morada*	A00ZT	Potatoes	Spain	Spain		flesh	yellow		2.1±0.1			98
Potato	*Solanum andigena var. muro Shocco*	A00ZT	Potatoes	Spain	Spain		flesh	purple and white		22.3±0.7			98
Potato	*Solanum andigena var. Negrita*	A00ZT	Potatoes	Spain	Spain		flesh	white and purple		6.2±0.2			98
Potato	*Solanum andigena var. Puca Quitish*	A00ZT	Potatoes	Spain	Spain		flesh	purple and white		9.2±0.4			98
Potato	*Solanum andigena var. sinpancachi*	A00ZT	Potatoes	Spain	Spain		flesh	yellow		7.4±0.7			98
Potato	*Solanum goniocalix var. kashpadana amarilla*	A00ZT	Potatoes	Spain	Spain		flesh	light yellow		34.6±2.1			98
Potato	*Solanum phureja var. chaucha*	A00ZT	Potatoes	Spain	Spain		flesh	yellow with purple spots		9.1±1.7			98
Potato	*Solanum phureja var. elodie*	A00ZT	Potatoes	Spain	Spain		flesh	yellow		0.3±0			98
Potato	*Solanum phureja var. iker*	A00ZT	Potatoes	Spain	Spain		flesh	yellow		1.6±0			98

Table S8.1.1 Potatoes and similar (A0DPP) (µg/100g) (continuation)

Food name	Scientific name	FoodEx2_ TermCode	FoodEx2_ TermName	Origin (country)	Purchase (country)	Water (%)	Part analysed	Colour	α-carotene	β-carotene	β-cryptoxanthin	ζ-carotene	Ref.
Potato	*Solanum stenotonum var. morada turuna*	A00ZT	Potatoes	Spain	Spain		flesh	yellow and maroon		21.1±0.8			98
Potato	*Solanum stenotonum var. morar Nayra Mari*	A00ZT	Potatoes	Spain	Spain		flesh	purple and yellow		13.3±1.1			98
Potato	*Solanum stenotonum var. poluya*	A00ZT	Potatoes	Spain	Spain		flesh	yellow with maroon spots		13.6±1.4			98
Potato	*Solanum stenotonum var. señora Warni*	A00ZT	Potatoes	Spain	Spain		flesh	yellow		7.3±0.4			98
Potato	*Solanum tuberosum L.*	A00ZT	Potatoes	Germany	Germany	78.2	edible part			5	3		53
Potato	*Solanum tuberosum L.*	A00ZT	Potatoes	Spain	Spain		edible part	white	tr.	2	tr.		24
Potato	*Solanum tuberosum L.*	A00ZT	Potatoes	Spain	Spain		edible part	white	tr.	1	tr.		24
Potato	*Solanum tuberosum L.*	A00ZT	Potatoes	Spain	Spain		edible part			2			24
Potato	*Solanum tuberosum L.*	A00ZT	Potatoes	Spain	Spain		edible part			1			24
Potato	*Solanum tuberosum L.*	A00ZT	Potatoes	Spain	Spain		flesh	yellow and maroon		4.3±0.1			98
Potato	*Solanum tuberosum L.*	A00ZT	Potatoes	Spain	Spain		flesh	purple and white		8.3±0.2			98
Potato	*Solanum tuberosum L.*	A00ZT#F20.A07QF	Potatoes, PART-CONSUMED-ANALYSED = W/o peel	Latvia	Latvia		tubers without peel	light yellow to violet		68 - 385			24
Potato	*Solanum tuberosum L.*	A00ZT#F20.A07QF	Potatoes, PART-CONSUMED-ANALYSED = W/o peel	Spain	Spain	82	Tuber without skin	yellow		1±0.2			24

Table S8.1.1 Potatoes and similar (A0DPP) (µg/100g) (continuation)

Food name	Scientific name	FoodEx2_ TermCode	FoodEx2_ TermName	Origin (country)	Purchase (country)	Water (%)	Part analysed	Colour	α-carotene	β-carotene	β-cryptoxanthin	ζ-carotene	Ref.
Potato	*Solanum tuberosum L.*	A00ZT#F20.A07QF	Potatoes, PART-CONSUMED-ANALYSED = W/o peel	Spain	Spain	80	tuber without skin	yellow		2±0.3			24
Potato	*Solanum tuberosum L. var. alegria oro*	A00ZT	Potatoes	Spain	Spain		flesh	yellow		0.9±0			98
Potato	*Solanum tuberosum L. var. harana*	A00ZT	Potatoes	Spain	Spain		flesh	yellow		3.9±0.3			98
Potato	*Solanum tuberosum L. var. agria*	A00ZT	Potatoes	Spain	Spain		flesh	yellow		2.9±0.2			98
Potato	*Solanum tuberosum L. var. ambition*	A00ZT	Potatoes	Spain	Spain		flesh	yellow		2.7±0			98
Potato	*Solanum tuberosum L. var. arene*	A00ZT	Potatoes	Spain	Spain		flesh	yellow		0.9±0			98
Potato	*Solanum tuberosum L. var. arrow*	A00ZT	Potatoes	Spain	Spain		flesh	light yellow		8.0±0.2			98
Potato	*Solanum tuberosum L. var. ayala*	A00ZT	Potatoes	Spain	Spain		flesh	yellow		3.4±0			98
Potato	*Solanum tuberosum L. var. buesa*	A00ZT	Potatoes	Spain	Spain		flesh	yellow		1.5±0.1			98
Potato	*Solanum tuberosum L. var. cazona*	A00ZT	Potatoes	Spain	Spain		flesh	yellow		1.3±0.1			98
Potato	*Solanum tuberosum L. var. cherie*	A00ZT	Potatoes	Spain	Spain		flesh	yellow		0.3±0			98
Potato	*Solanum tuberosum L. var. corine*	A00ZT	Potatoes	Spain	Spain		flesh	light yellow		2.4±0.1			98

Table S8.1.1 Potatoes and similar (A0DPP) (µg/100g) (continuation)

Food name	Scientific name	FoodEx2_ TermCode	FoodEx2_ TermName	Origin (country)	Purchase (country)	Water (%)	Part analysed	Colour	α-carotene	β-carotene	β-cryptoxanthin	ζ-carotene	Ref.
Potato	*Solanum tuberosum L. var. desiree*	A00ZT	Potatoes	Spain	Spain		flesh	yellow		0.2±0			98
Potato	*Solanum tuberosum L. var. duquesa*	A00ZT	Potatoes	Spain	Spain		flesh	yellow		2.5±0.3			98
Potato	*Solanum tuberosum L. var. duquesa*	A00ZT	Potatoes	Spain	Spain		flesh	yellow		2.5±0.3			98
Potato	*Solanum tuberosum L. var. fina de Carvallo*	A00ZT	Potatoes	Spain	Spain		flesh	yellow		2.6±0			98
Potato	*Solanum tuberosum L. var. fina de Gredos*	A00ZT	Potatoes	Spain	Spain		flesh	light yellow		1.1±0.1			98
Potato	*Solanum tuberosum L. var. ibicenca*	A00ZT	Potatoes	Spain	Spain		flesh	yellow		6.6±0.4			98
Potato	*Solanum tuberosum L. var. kenebec*	A00ZT	Potatoes	Spain	Spain		flesh	light yellow		4.7 ±0.4			98
Potato	*Solanum tuberosum L. var. leire*	A00ZT	Potatoes	Spain	Spain		flesh	yellow		2.5±0.1			98
Potato	*Solanum tuberosum L. var. maica*	A00ZT	Potatoes	Spain	Spain		flesh	yight yellow		3.2±0.1			98
Potato	*Solanum tuberosum L. var. miranda*	A00ZT	Potatoes	Spain	Spain		flesh	yellow		6.3±0.5			98
Potato	*Solanum tuberosum L. var. mirari*	A00ZT	Potatoes	Spain	Spain		flesh	yellow		1.6±0.1			98
Potato	*Solanum tuberosum L. var. monalisa*	A00ZT	Potatoes	Spain	Spain		flesh	yellow		1.1±0.1			98

Table S8.1.1 Potatoes and similar (A0DPP) (µg/100g) (continuation)

Food name	Scientific name	FoodEx2_ TermCode	FoodEx2_ TermName	Origin (country)	Purchase (country)	Water (%)	Part analysed	Colour	α-carotene	β-carotene	β-cryptoxanthin	ζ-carotene	Ref.
Potato	*Solanum tuberosum L. var. montico*	A00ZT	Potatoes	Spain	Spain		flesh	light yellow		2.6±0.1			98
Potato	*Solanum tuberosum L. var. murato*	A00ZT	Potatoes	Spain	Spain		flesh	yellow		1.2±0.1			98
Potato	*Solanum tuberosum L. var. Nagore*	A00ZT	Potatoes	Spain	Spain		flesh	yellow		1.8±0,1			98
Potato	*Solanum tuberosum L. var. Nerea*	A00ZT	Potatoes	Spain	Spain		flesh	yellow		0.6±0			98
Potato	*Solanum tuberosum L. var. onda*	A00ZT	Potatoes	Spain	Spain		flesh	yellow		1.3±0.1			98
Potato	*Solanum tuberosum L. var. opal*	A00ZT	Potatoes	Spain	Spain		flesh	yellow		0.3±0			98
Potato	*Solanum tuberosum L. var. Pedro Muñoz*	A00ZT	Potatoes	Spain	Spain		flesh	yellow		5.8±0.2			98
Potato	*Solanum tuberosum L. var. red pontiac*	A00ZT	Potatoes	Spain	Spain		flesh	light yellow		4.7±0			98
Potato	*Solanum tuberosum L. var. roja riñón*	A00ZT	Potatoes	Spain	Spain		flesh	yellow		0.7±0.1		65.4±2.3	98
Potato	*Solanum tuberosum L. var. romula*	A00ZT	Potatoes	Spain	Spain		flesh	yellow		4.2±0.2			98
Potato	*Solanum tuberosum L. var. Sofía*	A00ZT	Potatoes	Spain	Spain		flesh	yellow		1.5±0.1			98
Potato	*Solanum tuberosum L. var. spunta*	A00ZT	Potatoes	Spain	Spain		flesh	yellow		0.6±0.1			98

Table S8.1.1 Potatoes and similar (A0DPP) (µg/100g) (continuation)

Food name	Scientific name	FoodEx2_ TermCode	FoodEx2_ TermName	Origin (country)	Purchase (country)	Water (%)	Part analysed	Colour	α-carotene	β-carotene	β-cryptoxanthin	ζ-carotene	Ref.
Potato	*Solanum tuberosum L. var. stemster*	A00ZT	Potatoes	Spain	Spain		flesh	yellow		4.5±0.1			98
Potato	*Solanum tuberosum L. var. tramontana*	A00ZT	Potatoes	Spain	Spain		flesh	yellow		0.2±0			98
Potato	*Solanum tuberosum L. var. Victor*	A00ZT	Potatoes	Spain	Spain		flesh	yellow		6.8±0.3			98
Potato	*Solanum tuberosum L. var. zadorra*	A00ZT	Potatoes	Spain	Spain		flesh	yellow		2.3±0.2			98
Potato	*Solanum tuberosum L. var. zafira*	A00ZT	Potatoes	Spain	Spain		flesh	yellow		1.0±0			98
Potato	*Solanum tuberosum L. var. zela*	A00ZT	Potatoes	Spain	Spain		flesh	yellow		4.0±0.2			98
Potato	*Solanum tuberosum L. var. zorba*	A00ZT	Potatoes	Spain	Spain		flesh	yellow		0.2±0			98
Potato	*Solanum tuberosum L. var. zunta*	A00ZT	Potatoes	Spain	Spain		flesh	yellow		2.1±0.3			98

Table S8.1.2 Potatoes and similar (A0DPP) (µg/100g) (continuation)

Food name	Scientific name	FoodEx2_ TermCode	FoodEx2_ TermName	Origin (country)	Purchase (country)	Water (%)	Part analysed	Colour	Antheraxanthin	E(v. trans)-α-carotene	E(v. trans)-β-carotene	Lutein	Ref.
Potato	*Solanum andigena 'Puca Quitish'*	A00ZT	Potatoes	Spain	Spain		flesh	purple and white	5.8±0.2			40.0±1.7	98
Potato	*Solanum andigena var. jesus*	A00ZT	Potatoes	Spain	Spain		flesh	purple and white	4.5±0.3			33.4±2.2	98
Potato	*Solanum andigena var. kasta*	A00ZT	Potatoes	Spain	Spain		flesh	purple and white	5.0±0.2			15.5±0.1	98
Potato	*Solanum andigena var. marfona*	A00ZT	Potatoes	Spain	Spain		flesh	yellow	28.4±1.7			93.5±4.3	98
Potato	*Solanum andigena var. morada*	A00ZT	Potatoes	Spain	Spain		flesh	yellow	3.1±0.1			14.3±0.6	98
Potato	*Solanum andigena var. muro Shocco*	A00ZT	Potatoes	Spain	Spain		flesh	purple and white	12.6±0.3			87.0±0.2	98
Potato	*Solanum andigena var. Negrita*	A00ZT	Potatoes	Spain	Spain		flesh	white and purple	7.4±0.4			38.6±1.3	98
Potato	*Solanum andigena var. sinpancachi*	A00ZT	Potatoes	Spain	Spain		flesh	yellow	28.4±3.7			102.9±9.9	98
Potato	*Solanum goniocalix var. kashpadana amarilla*	A00ZT	Potatoes	Spain	Spain		flesh	light yellow	34.6±2.1			19.9±0.8	98
Potato	*Solanum phureja var. chaucha*	A00ZT	Potatoes	Spain	Spain		flesh	yellow with purple spots	40.3±1.9			143.8±8.6	98
Potato	*Solanum phureja var. elodie*	A00ZT	Potatoes	Spain	Spain		flesh	yellow	9.2±0.1			53.9±2.4	98
Potato	*Solanum phureja var. iker*	A00ZT	Potatoes	Spain	Spain		flesh	yellow	94.3±3			115.5±2.9	98

Table S8.1.2 Potatoes and similar (A0DPP) (µg/100g) (continuation)

Food name	Scientific name	FoodEx2_ TermCode	FoodEx2_ TermName	Origin (country)	Purchase (country)	Water (%)	Part analysed	Colour	Antheraxanthin	E(v. trans)-α-carotene	E(v. trans)-β-carotene	Lutein	Ref.
Potato	*Solanum stenotonum var. morada turuna*	A00ZT	Potatoes	Spain	Spain		flesh	yellow and maroon	19.8±0.2			180.9±1	98
Potato	*Solanum stenotonum var. morar Nayra Mari*	A00ZT	Potatoes	Spain	Spain		flesh	purple and yellow	14.8±1.4			102.9±5.8	98
Potato	*Solanum stenotonum var. poluya*	A00ZT	Potatoes	Spain	Spain		flesh	yellow with maroon spots	51.2±2.4			91.0±6.1	98
Potato	*Solanum stenotonum var. señora Warni*	A00ZT	Potatoes	Spain	Spain		flesh	yellow	15.9±1.6			68.5±7.8	98
Potato	*Solanum tuberosum L.*	A00ZT	Potatoes	Germany	Germany	78.2	edible part					100	53
Potato	*Solanum tuberosum L.*	A00ZT	Potatoes	Spain	Spain		edible part	white				44	24
Potato	*Solanum tuberosum L.*	A00ZT	Potatoes	Spain	Spain		edible part	white				12	24
Potato	*Solanum tuberosum L.*	A00ZT	Potatoes	Spain	Spain		edible part					44	24
Potato	*Solanum tuberosum L.*	A00ZT	Potatoes	Spain	Spain		edible part					12	24
Potato	*Solanum tuberosum L.*	A00ZT	Potatoes	Spain	Spain		flesh	yellow /maroon	39.7±0.5			106.4±0.6	98
Potato	*Solanum tuberosum L.*	A00ZT	Potatoes	Spain	Spain		flesh	purple and white	5.7±0.1			38.7±0.6	98
Potato	*Solanum tuberosum L.*	A00ZT#F20.A07QF	Potatoes, PART-CONSUMED-ANALYSED = W/o peel	Spain	Spain	82	tuber without skin	yellowish				12±1	24
Potato	*Solanum tuberosum L.*	A00ZT#F20.A07QF	Potatoes, PART-CONSUMED-ANALYSED = W/o peel	Spain	Spain	80	tuber without skin	yellowish				44±1	24
Potato	*Solanum tuberosum L. var. montico*	A00ZT	Potatoes	Spain	Spain		flesh	light yellow	31.6±0.6			84.0±3.4	98

Table S8.1.2 Potatoes and similar (A0DPP) (µg/100g) (continuation)

Food name	Scientific name	FoodEx2_ TermCode	FoodEx2_ TermName	Origin (country)	Purchase (country)	Water (%)	Part analysed	Colour	Antheraxanthin	E(v. trans)-α-carotene	E(v. trans)-β-carotene	Lutein	Ref.
Potato	*Solanum tuberosum L. var.opal*	A00ZT	Potatoes	Spain	Spain		flesh	yellow	8.1±0.6			16.7±1.9	98
Potato	*Solanum tuberosum L. Andigenum*	A00ZT#F28.A07GL	Potatoes, PROCESS = Boiling	Peru			tuber boiled	yellow				155.7±5.4	99
Potato	*Solanum tuberosum L. Andigenum*	A00ZT#F28.A07GL	Potatoes, PROCESS = Boiling	Peru			tuber boiled	yellow				295.0±10.6	99
Potato	*Solanum tuberosum L. Andigenum*	A00ZT#F28.A07GL	Potatoes, PROCESS = Boiling	Peru			tuber boiled	yellow				132.1±2.6	99
Potato	*Solanum tuberosum L. Andigenum var. Amarilla del Centro*	A00ZT#F28.A07GL	Potatoes, PROCESS = Boiling	Peru			edible tubers boiled	yellow fleshed				168.4±4.1	99
Potato	*Solanum tuberosum L. Andigenum var. Amarilla Tumbay*	A00ZT#F28.A07GL	Potatoes, PROCESS = Boiling	Peru			edible tubers boiled	yellow fleshed				395.7±10.6	99
Potato	*Solanum tuberosum L. Andigenum var. Ishkupuru*	A00ZT#F28.A07GL	Potatoes, PROCESS = Boiling	Peru			edible tubers boiled	yellow fleshed				263.2±13.5	99
Potato	*Solanum tuberosum L. Andigenum var. Yema de Huevo*	A00ZT#F28.A07GL	Potatoes, PROCESS = Boiling	Peru			edible tubers boiled	yellow fleshed				65.4±2.5	99
Potato	*Solanum tuberosum L. var. agria*	A00ZT	Potatoes	Spain	Spain		flesh	yellow	22.8±0.1			97.5±5.1	98
Potato	*Solanum tuberosum L. var. alegria oro*	A00ZT	Potatoes	Spain	Spain		flesh	yellow	15.9±0.3			36.3±0.5	98
Potato	*Solanum tuberosum L. var. ambition*	A00ZT	Potatoes	Spain	Spain		flesh	yellow	24.3±0.3			72.4±3.7	98

Table S8.1.2 Potatoes and similar (A0DPP) (µg/100g) (continuation)

**Food name**	**Scientific name**	**FoodEx2_ TermCode**	**FoodEx2_ TermName**	**Origin (country)**	**Purchase (country)**	**Water (%)**	**Part analysed**	**Colour**	**Antheraxanthin**	**E(v. trans)-α-carotene**	**E(v. trans)-β-carotene**	**Lutein**	Ref.
Potato	*Solanum tuberosum L. var. arene*	A00ZT	Potatoes	Spain	Spain		flesh	yellow	16.5±0.5			77.2±3.8	98
Potato	*Solanum tuberosum L. var. arrow*	A00ZT	Potatoes	Spain	Spain		flesh	light yellow	7.3±0.4			50.2±1.3	98
Potato	*Solanum tuberosum L. var. ayala*	A00ZT	Potatoes	Spain	Spain		flesh	yellow	53.1±2.1			104.8±4.8	98
Potato	*Solanum tuberosum L. var. buesa*	A00ZT	Potatoes	Spain	Spain		flesh	yellow	24.1±0.3			51.7±0.2	98
Potato	*Solanum tuberosum L. var. cazona*	A00ZT	Potatoes	Spain	Spain		flesh	yellow	2.2±0.3			27.2±1.8	98
Potato	*Solanum tuberosum L. var. cherie*	A00ZT	Potatoes	Spain	Spain		flesh	yellow	11.0±0.6			49.6±2.3	98
Potato	*Solanum tuberosum L. var. corine*	A00ZT	Potatoes	Spain	Spain		flesh	light yellow	13.5±0.2			51.0±2.1	98
Potato	*Solanum tuberosum L. var. desiree*	A00ZT	Potatoes	Spain	Spain		flesh	yellow	8.7±0.4			21.8±0.9	98
Potato	*Solanum tuberosum L. var. duquesa*	A00ZT	Potatoes	Spain	Spain		flesh	yellow	30.1±2.1			55.4±2.6	98
Potato	*Solanum tuberosum L. var. duquesa*	A00ZT	Potatoes	Spain	Spain		flesh	yellow	30.1±2.1			55.4±2.6	98
Potato	*Solanum tuberosum L. var. fina de Carvallo*	A00ZT	Potatoes	Spain	Spain		flesh	yellow	4.4±0.2			43.9±1.1	98
Potato	*Solanum tuberosum L. var. fina de Gredos*	A00ZT	Potatoes	Spain	Spain		flesh	light yellow	9.2±0			35.5±0.4	98

Table S8.1.2 Potatoes and similar (A0DPP) (µg/100g) (continuation)

Food name	Scientific name	FoodEx2_ TermCode	FoodEx2_ TermName	Origin (country)	Purchase (country)	Water (%)	Part analysed	Colour	Antheraxanthin	E(v. trans)-α-carotene	E(v. trans)-β-carotene	Lutein	Ref.
Potato	*Solanum tuberosum L. var. harana*	A00ZT	Potatoes	Spain	Spain		flesh	yellow	36.2±1.5			88.5±1.5	98
Potato	*Solanum tuberosum L. var. ibicenca*	A00ZT	Potatoes	Spain	Spain		flesh	yellow	27.9±1			140.3±7.9	98
Potato	*Solanum tuberosum L. var. kenebec*	A00ZT	Potatoes	Spain	Spain		flesh	light yellow	5.9±0.2			58.9±3.4	98
Potato	*Solanum tuberosum L. var. leire*	A00ZT	Potatoes	Spain	Spain		flesh	yellow	63.2±4.7			105.6±5.3	98
Potato	*Solanum tuberosum L. var. maica*	A00ZT	Potatoes	Spain	Spain		flesh	light yellow	4.9±0.1			36.9±0.5	98
Potato	*Solanum tuberosum L. var. miranda*	A00ZT	Potatoes	Spain	Spain		flesh	yellow	36.4±1.6			130.6±7.5	98
Potato	*Solanum tuberosum L. var. mirari*	A00ZT	Potatoes	Spain	Spain		flesh	yellow	27.5±2.1			53.4±2.4	98
Potato	*Solanum tuberosum L. var. monalisa*	A00ZT	Potatoes	Spain	Spain		flesh	yellow	14.6±2.7			32.9±5	98
Potato	*Solanum tuberosum L. var. murato*	A00ZT	Potatoes	Spain	Spain		flesh	yellow	21.5±0.9			91.4±7.4	98
Potato	*Solanum tuberosum L. var. Nagore*	A00ZT	Potatoes	Spain	Spain		flesh	yellow	13.9±0.7			37.3±0.2	98
Potato	*Solanum tuberosum L. var. Nerea*	A00ZT	Potatoes	Spain	Spain		flesh	yellow	22.8±1.3			77.4±4.2	98
Potato	*Solanum tuberosum L. var. onda*	A00ZT	Potatoes	Spain	Spain		flesh	yellow	18.5±0.3			52.2±0.5	98

Table S8.1.2 Potatoes and similar (A0DPP) (µg/100g) (continuation)

Food name	Scientific name	FoodEx2_ TermCode	FoodEx2_ TermName	Origin (country)	Purchase (country)	Water (%)	Part analysed	Colour	Antheraxanthin	E(v. trans)-α-carotene	E(v. trans)-β-carotene	Lutein	Ref.
Potato	*Solanum tuberosum L. var. Pedro Muñoz*	A00ZT	Potatoes	Spain	Spain		flesh	yellow	14.8±0			62.6 ±0.1	98
Potato	*Solanum tuberosum L. var. red pontiac*	A00ZT	Potatoes	Spain	Spain		flesh	light yellow	10.8±0.3			89.3±1.3	98
Potato	*Solanum tuberosum L. var. roja riñón*	A00ZT	Potatoes	Spain	Spain		flesh	yellow	22.0±2			67.0±2.3	98
Potato	*Solanum tuberosum L. var. romula*	A00ZT	Potatoes	Spain	Spain		flesh	yellow	29.0±1			101.2±5.1	98
Potato	*Solanum tuberosum L. var. Sofía*	A00ZT	Potatoes	Spain	Spain		flesh	yellow	19.1±2.4			59.1±4.8	98
Potato	*Solanum tuberosum L. var. spunta*	A00ZT	Potatoes	Spain	Spain		flesh	yellow	13.2±1.1			31.2±1.2	98
Potato	*Solanum tuberosum L. var. stemster*	A00ZT	Potatoes	Spain	Spain		flesh	yellow	21.7±1.1			56.2±1.9	98
Potato	*Solanum tuberosum L. var. tramontana*	A00ZT	Potatoes	Spain	Spain		flesh	yellow	6.1±1.3			12.5±2.2	98
Potato	*Solanum tuberosum L. var. Victor*	A00ZT	Potatoes	Spain	Spain		flesh	yellow	10.2±0			54.5±4.1	98
Potato	*Solanum tuberosum L. var. zadorra*	A00ZT	Potatoes	Spain	Spain		flesh	yellow	31.6±1.2			70.2±5.9	98
Potato	*Solanum tuberosum L. var. zafira*	A00ZT	Potatoes	Spain	Spain		flesh	yellow	17.1±0.8			43.9±0.2	98
Potato	*Solanum tuberosum L. var. zela*	A00ZT	Potatoes	Spain	Spain		flesh	yellow	21.7±2.2			74.0±9.8	98

Table S8.1.2 Potatoes and similar (A0DPP) (µg/100g) (continuation)

Food name	Scientific name	FoodEx2_ TermCode	FoodEx2_ TermName	Origin (country)	Purchase (country)	Water (%)	Part analysed	Colour	Antheraxanthin	E(v. trans)-α-carotene	E(v. trans)-β-carotene	Lutein	Ref.
Potato	*Solanum tuberosum L. var. zorba*	A00ZT	Potatoes	Spain	Spain		flesh	yellow	3.4±0.1			6.2±0	98
Potato	*Solanum tuberosum L. var. zunta*	A00ZT	Potatoes	Spain	Spain		flesh	yellow	19.3±1.6			47.3±2.3	98

Table S8.1.3 Potatoes and similar (A0DPP) (µg/100g) (continuation)

Food name	Scientific name	FoodEx2_ TermCode	FoodEx2_ TermName	Origin (country)	Purchase (country)	Water (%)	Part analysed	Colour	Neoxanthin	Phytoene	Violaxanthin	Z(v. cis)-β-carotene	Zeaxanthin	Ref.
Potato	*Solanum andigena var. jesus*	A00ZT	Potatoes	Spain	Spain		flesh	purple and white	28.6±1.8		8.6±0.4			98
Potato	*Solanum andigena var. kasta*	A00ZT	Potatoes	Spain	Spain		flesh	purple and white	55.0±1		17.0±1.6			98
Potato	*Solanum andigena var. marfona*	A00ZT	Potatoes	Spain	Spain		flesh	yellow	272.8±22.3		219.1±10.6			98
Potato	*Solanum andigena var. morada*	A00ZT	Potatoes	Spain	Spain		flesh	yellow	17.6±0.5		12.7±0.4			98
Potato	*Solanum andigena var. muro Shocco*	A00ZT	Potatoes	Spain	Spain		flesh	purple and white	81.4±0.4		42.2±1			98
Potato	*Solanum andigena var. Negrita*	A00ZT	Potatoes	Spain	Spain		flesh	white and purple	29.3±1.7		9.6±0.6			98
Potato	*Solanum andigena var. Puca Quitish*	A00ZT	Potatoes	Spain	Spain		flesh	purple and white	28.3±0.6		11.0±0.4			98

Table S8.1.3 Potatoes and similar (A0DPP) (µg/100g) (continuation)

Food name	Scientific name	FoodEx2_ TermCode	FoodEx2_ TermName	Origin (country)	Purchase (country)	Water (%)	Part analysed	Colour	Neoxanthin	Phytoene	Violaxanthin	Z(v. cis)-β-carotene	Zeaxanthin	Ref.
Potato	*Solanum andigena var. sinpancachi*	A00ZT	Potatoes	Spain	Spain		flesh	yellow	106.5±6.5		330.9±30.8			98
Potato	*Solanum goniocalix var. kashpadana amarilla*	A00ZT	Potatoes	Spain	Spain		flesh	light yellow	112.2±11.3		19.9±0.8			98
Potato	*Solanum phureja var. chaucha*	A00ZT	Potatoes	Spain	Spain		flesh	yellow with purple spots	175.2±7.5		307.4±17.8			98
Potato	*Solanum phureja var. iker*	A00ZT	Potatoes	Spain	Spain		flesh	yellow	33.9±0.9		115.5±2.9			98
Potato	*Solanum phureja var.elodie*	A00ZT	Potatoes	Spain	Spain		flesh	yellow	64.5±3.5		24.3±0.9			98
Potato	*Solanum stenotonum var. morada turuna*	A00ZT	Potatoes	Spain	Spain		flesh	yellow and maroon	41.6±0.9		39.1±1.3			98
Potato	*Solanum stenotonum var. morar Nayra Mari*	A00ZT	Potatoes	Spain	Spain		flesh	purple and yellow	66.7±5.4		18.9±1.3			98
Potato	*Solanum stenotonum var. poluya*	A00ZT	Potatoes	Spain	Spain		flesh	yellow with maroon spots	206.0±8.4		248.8±11.3			98
Potato	*Solanum stenotonum var. señora Warni*	A00ZT	Potatoes	Spain	Spain		flesh	yellow	69.5±8.7		81.1±10.3			98
Potato	*Solanum tuberosum* L.	A00ZT	Potatoes	Germany	Germany	78.2	edible part		14		18		16	53
Potato	*Solanum tuberosum* L.	A00ZT	Potatoes	Spain	Spain		edible part	white					21	24
Potato	*Solanum tuberosum* L.	A00ZT	Potatoes	Spain	Spain		edible part	white					4	24
Potato	*Solanum tuberosum* L.	A00ZT	Potatoes	Spain	Spain		edible part						21	24

Table S8.1.3 Potatoes and similar (A0DPP) (µg/100g) (continuation)

Food name	Scientific name	FoodEx2_ TermCode	FoodEx2_ TermName	Origin (country)	Purchase (country)	Water (%)	Part analysed	Colour	Neoxanthin	Phytoene	Violaxanthin	Z(v. cis)-β-carotene	Zeaxanthin	Ref.
Potato	*Solanum tuberosum L.*	A00ZT	Potatoes	Spain	Spain		edible part						4	24
Potato	*Solanum tuberosum L.*	A00ZT	Potatoes	Spain	Spain		flesh	yellow and maroon	114.7±1.5		219.6±5.4			98
Potato	*Solanum tuberosum L.*	A00ZT	Potatoes	Spain	Spain		flesh	purple and white	27.7±0.3		14.1±0.2			98
Potato	*Solanum tuberosum L.*	A00ZT#F20. A07QF	Potatoes, PART-CONSUMED-ANALYSED = W/o peel	Spain	Spain	82	without skin	yellow					4±0.5	24
Potato	*Solanum tuberosum L.*	A00ZT#F20. A07QF	Potatoes, PART-CONSUMED-ANALYSED = W/o peel	Spain	Spain	80	without skin	yellow					21±0.5	24
Potato	*Solanum tuberosum L. Andigenum*	A00ZT#F28. A07GL	Potatoes, PROCESS = Boiling	Peru	Peru		tuber boiled	yellow					1196.2± 32.1	99
Potato	*Solanum tuberosum L. Andigenum*	A00ZT#F28. A07GL	Potatoes, PROCESS = Boiling	Peru	Peru		tuber boiled	yellow					654.2±34.9	99
Potato	*Solanum tuberosum L. Andigenum*	A00ZT#F28. A07GL	Potatoes, PROCESS = Boiling	Peru	Peru		tuber boiled	yellow					682.7±28.2	99
Potato	*Solanum tuberosum L. Andigenum var. Amarilla del Centro*	A00ZT#F28. A07GL	Potatoes, PROCESS = Boiling	Peru			edible tubers boliled	yellow fleshed					697.1±14.6	99
Potato	*Solanum tuberosum L. Andigenum var. Amarilla Tumbay*	A00ZT#F28. A07GL	Potatoes, PROCESS = Boiling	Peru			edible tubers bolied	yellow fleshed					51.3±1.6	99
Potato	*Solanum tuberosum L. Andigenum var. Ishkupuru*	A00ZT#F28. A07GL	Potatoes, PROCESS = Boiling	Peru			edible tubers boiled	yellow fleshed					66.6±5.9	99

Table S8.1.3 Potatoes and similar (A0DPP) (µg/100g) (continuation)

Food name	Scientific name	FoodEx2_ TermCode	FoodEx2_ TermName	Origin (country)	Purchase (country)	Water (%)	Part analysed	Colour	Neoxanthin	Phytoene	Violaxanthin	Z(v. cis)-β-carotene	Zeaxanthin	Ref.
Potato	*Solanum tuberosum L. Andigenum var. Yema de Huevo*	A00ZT#F28. A07GL	Potatoes, PROCESS = Boiling	Peru			edible tubers boiled	yellow fleshed					1106.1± 17.6	99
Potato	*Solanum tuberosum L. var. agria*	A00ZT	Potatoes	Spain	Spain		flesh	yellow	124.3±3.3		292.6±6.3			98
Potato	*Solanum tuberosum L. var. alegria oro*	A00ZT	Potatoes	Spain	Spain		flesh	yellow	61.9±1.4		143.0±1.5			98
Potato	*Solanum tuberosum L. var. ambition*	A00ZT	Potatoes	Spain	Spain		flesh	yellow	123.7±1.7		152.1±2.7			98
Potato	*Solanum tuberosum L. var. arene*	A00ZT	Potatoes	Spain	Spain		flesh	yellow	63.9±3		143.8±4.6			98
Potato	*Solanum tuberosum L. var. arrow*	A00ZT	Potatoes	Spain	Spain		flesh	light yellow	24.5±1		20.7±0.9			98
Potato	*Solanum tuberosum L. var. ayala*	A00ZT	Potatoes	Spain	Spain		flesh	yellow	124.1±0.4		350.8±9.9			98
Potato	*Solanum tuberosum L. var. buesa*	A00ZT	Potatoes	Spain	Spain		flesh	yellow	42.1±1		132.9±5.3			98
Potato	*Solanum tuberosum L. var. cazona*	A00ZT	Potatoes	Spain	Spain		flesh	yellow	11.7±1.4		8.2±0.9			98
Potato	*Solanum tuberosum L. var. cherie*	A00ZT	Potatoes	Spain	Spain		flesh	yellow	81.1±3.2		129.4±5.8			98
Potato	*Solanum tuberosum L. var. corine*	A00ZT	Potatoes	Spain	Spain		flesh	light yellow	59.4±1.4		177.5±7.8			98
Potato	*Solanum tuberosum L. var. desiree*	A00ZT	Potatoes	Spain	Spain		flesh	yellow	42.1±1.5		16.7±0.6			98

Table S8.1.3 Potatoes and similar (A0DPP) (μg/100g) (continuation)

Food name	Scientific name	FoodEx2_ TermCode	FoodEx2_ TermName	Origin (country)	Purchase (country)	Water (%)	Part analysed	Colour	Neoxanthin	Phytoene	Violaxanthin	Z(v. cis)-β-carotene	Zeaxanthin	Ref.
Potato	*Solanum tuberosum L. var. duquesa*	A00ZT	Potatoes	Spain	Spain		flesh	yellow	44.2±2.6		262.9±9.7			98
Potato	*Solanum tuberosum L. var. duquesa*	A00ZT	Potatoes	Spain	Spain		flesh	yellow	44.2±2.6		262.9±9.7			98
Potato	*Solanum tuberosum L. var. fina de Carvallo*	A00ZT	Potatoes	Spain	Spain		flesh	yellow	15.1±0.5		6.1±0.1			98
Potato	*Solanum tuberosum L. var. fina de Gredos*	A00ZT	Potatoes	Spain	Spain		flesh	light yellow	57.8±0.4		77.9±0.7			98
Potato	*Solanum tuberosum L. var. harana*	A00ZT	Potatoes	Spain	Spain		flesh	yellow	111.9±2.6		213.7±2.7			98
Potato	*Solanum tuberosum L. var. ibicenca*	A00ZT	Potatoes	Spain	Spain		flesh	yellow	79.4±3.8		276.9±9.4			98
Potato	*Solanum tuberosum L. var. kenebec*	A00ZT	Potatoes	Spain	Spain		flesh	light yellow	58.9±3.4		106.0±1.4			98
Potato	*Solanum tuberosum L. var. leire*	A00ZT	Potatoes	Spain	Spain		flesh	yellow	63.9±7.3		282.4±20.5			98
Potato	*Solanum tuberosum L. var. maica*	A00ZT	Potatoes	Spain	Spain		flesh	light yellow	22.4±0.7		9.1±0.1			98
Potato	*Solanum tuberosum L. var. miranda*	A00ZT	Potatoes	Spain	Spain		flesh	yellow	137.2±5.1		255.9±14.8			98
Potato	*Solanum tuberosum L. var. mirari*	A00ZT	Potatoes	Spain	Spain		flesh	yellow	63.1±3.2		183.6±12			98
Potato	*Solanum tuberosum L. var. monalisa*	A00ZT	Potatoes	Spain	Spain		flesh	yellow	81.4±12.1		99.6±16.1			98

Table S8.1.3 Potatoes and similar (A0DPP) (μg/100g) (continuation)

Food name	Scientific name	FoodEx2_ TermCode	FoodEx2_ TermName	Origin (country)	Purchase (country)	Water (%)	Part analysed	Colour	Neoxanthin	Phytoene	Violaxanthin	Z(v. cis)-β-carotene	Zeaxanthin	Ref.
Potato	*Solanum tuberosum L. var. montico*	A00ZT	Potatoes	Spain	Spain		flesh	light yellow	57.0±2		314.5±14.8			98
Potato	*Solanum tuberosum L. var. murato*	A00ZT	Potatoes	Spain	Spain		flesh	yellow	143.6±7.1		234.8±9.7			98
Potato	*Solanum tuberosum L. var. Nagore*	A00ZT	Potatoes	Spain	Spain		flesh	yellow	139.2±2.5		171.1±2.9			98
Potato	*Solanum tuberosum L. var. Nerea*	A00ZT	Potatoes	Spain	Spain		flesh	yellow	127.3±4.9		286.9±10.7			98
Potato	*Solanum tuberosum L. var. onda*	A00ZT	Potatoes	Spain	Spain		flesh	yellow	62.0±2.4		184.5±7.2			98
Potato	*Solanum tuberosum L. var. opal*	A00ZT	Potatoes	Spain	Spain		flesh	yellow	59.1±4.9		53.6±5.4			98
Potato	*Solanum tuberosum L. var. Pedro Muñoz*	A00ZT	Potatoes	Spain	Spain		flesh	yellow	140.3±0.9		81.3±0.7			98
Potato	*Solanum tuberosum L. var. red pontiac*	A00ZT	Potatoes	Spain	Spain		flesh	light yellow	34.8±0.2		14.1±0.1			98
Potato	*Solanum tuberosum L. var. roja riñón*	A00ZT	Potatoes	Spain	Spain		flesh	yellow	61.5±3.1		202.7 ±11.5			98
Potato	*Solanum tuberosum L. var. romula*	A00ZT	Potatoes	Spain	Spain		flesh	yellow	189.3±13.3		331.6±25			98
Potato	*Solanum tuberosum L. var. Sofía*	A00ZT	Potatoes	Spain	Spain		flesh	yellow	70.2±5.6		239.7±21.7			98
Potato	*Solanum tuberosum L. var. spunta*	A00ZT	Potatoes	Spain	Spain		flesh	yellow	72.4±3.4		122.5±7.2			98

Table S8.1.3 Potatoes and similar (A0DPP) (µg/100g) (continuation)

Food name	Scientific name	FoodEx2_ TermCode	FoodEx2_ TermName	Origin (country)	Purchase (country)	Water (%)	Part analysed	Colour	Neoxanthin	Phytoene	Violaxanthin	Z(v. cis)-β-carotene	Zeaxanthin	Ref.
Potato	*Solanum tuberosum L. var. stemster*	A00ZT	Potatoes	Spain	Spain		flesh	yellow	95.2±3.7		255.3±5.4			98
Potato	*Solanum tuberosum L. var. tramontana*	A00ZT	Potatoes	Spain	Spain		flesh	yellow	17.8±2.6		44.3±6.2			98
Potato	*Solanum tuberosum L. var. Victor*	A00ZT	Potatoes	Spain	Spain		flesh	yellow	37.5±1.4		34.1±0.6			98
Potato	*Solanum tuberosum L. var. zadorra*	A00ZT	Potatoes	Spain	Spain		flesh	yellow	47.8±1.4		270.4±13.7			98
Potato	*Solanum tuberosum L. var. zafira*	A00ZT	Potatoes	Spain	Spain		flesh	yellow	103.8±0.3		203.0±1.4			98
Potato	*Solanum tuberosum L. var. zela*	A00ZT	Potatoes	Spain	Spain		flesh	yellow	76.9±8.8		182.9±18.4			98
Potato	*Solanum tuberosum L. var. zorba*	A00ZT	Potatoes	Spain	Spain		flesh	yellow	36.2±0.4		18.0±0.3			98
Potato	*Solanum tuberosum L. var. zunta*	A00ZT	Potatoes	Spain	Spain		flesh	yellow	52.8±2.1		258.3±8.5			98

Table S8.2.1 Cassava roots and similar (A04JX) (µg/100g)

Food name	Scientific name	FoodEx2_ TermCode	FoodEx2_ TermName	Origin (country)	Purchase (country)	Water (%)	Part analysed	Colour	α-carotene	β-carotene	β-cryptoxanthin	ζ-carotene	Ref.
Cassava	*Manihot esculenta*	A00ZZ	Cassava roots	Brazil			root	yellow	nd	269	nd		100
Cassava	*Manihot esculenta*	A00ZZ	Cassava roots	Vanuatu			tubery roots	yellow/white	nd				100
Cassava	*Manihot esculenta*	A00ZZ	Cassava roots	Spain	Spain		edible part	white		8			24
Cassava	*Manihot esculenta*	A00ZZ	Cassava roots	Spain	Spain		edible part			8			24
Giant taro	*Alocasia macrorrhyza*	A010B	Taros	Vanuatu			corms	white	nd				100
Macabo	*Xanthosoma sagittifolium (L.)*	A010A	Tannias	Vanuatu			cormels	white, pink	nd				100
Pomtayer	*Xanthosoma sagittifolium (L.)*	A010A	Tannias	Netherlands	Belgium					513			20
Taro	*Colocasia esculenta*	A010B	Taros	Vanuatu			corms	red violet, red, yellow, pink, purple, white-yellow, white-purple, orange-purple, white	nd				100

Table S8.2.2 Cassava roots and similar (A04JX) (µg/100g) (continuation)

Food name	Scientific name	FoodEx2_ TermCode	FoodEx2_ TermName	Origin (country)	Purchase (country)	Water (%)	Part analysed	Colour	Antheraxanthin	E(v. trans)-α-carotene	E(v. trans)-β-carotene	Lutein	Ref.
Cassava	*Manihot esculenta*	A00ZZ	Cassava roots	Brazil			root	yellow				nd	100
Cassava	*Manihot esculenta*	A00ZZ	Cassava roots	Vanuatu			tubery roots	yellow/white			14.1–490.0	nd – 4.9	100
Giant taro	*Alocasia macrorrhyza*	A010B	Taros	Vanuatu			corms	white			tr.	tr.- 15.2	100

Table S8.2.2 Cassava roots and similar (A04JX) (µg/100g) (continuation)

Food name	Scientific name	FoodEx2_ TermCode	FoodEx2_ TermName	Origin (country)	Purchase (country)	Water (%)	Part analysed	Colour	Antheraxanthin	E(v. trans)-α-carotene	E(v. trans)-β-carotene	Lutein	Ref.
Macabo	*Xanthosoma sagittifolium (L.)*	A010A	Tannias	Vanuatu			cormels	white, pink			nd	11.4 - 19.1	100
Taro	*Colocasia esculenta*	A010B	Taros	Vanuatu			Corms	red violet, red, yellow, pink, purple, white-yellow, white-purple, orange-purple, white			tr.- 146.1	tr. - 11.4	100

Table S8.2.3 Cassava roots and similar (A04JX) (µg/100g) (continuation)

Food name	Scientific name	FoodEx2_ TermCode	FoodEx2_ TermName	Origin (country)	Purchase (country)	Water (%)	Part analysed	Colour	Neoxanthin	Phytoene	Violaxanthin	Z(v. cis)-β-carotene	Zeaxanthin	Ref.
Cassava	*Manihot esculenta*	A00ZZ	Cassava roots	Brazil			root	yellow	nd		nd		nd	100
Cassava	*Manihot esculenta*	A00ZZ	Cassava roots	Vanuatu			tubery roots	yellow/white		tr.–7032.4		13-cis-b-Carotene: tr.–195.6 and 9-cis-b-Carotene; tr.–189.1	nd	100
Giant taro	*Alocasia macrorrhyza*	A010B	Taros	Vanuatu			corms	white		nd		nd	nd	100
Macabo	*Xanthosoma sagittifolium (L.)*	A010A	Tannias	Vanuatu			cormels	white, pink		nd		nd	nd - tr.	100
Taro	*Colocasia esculenta*	A010B	Taros	Vanuatu			corms	red violet, red, yellow, pink, purple, white-yellow, white-purple, orange-purple, white		nd - tr.		13-cis: tr.-46.7; 9-cis: tr. -18.0	nd - tr.	100

Table S8.3.1 Sweet potatoes and similar (A0DPH) (µg/100g)

Food name	Scientific name	FoodEx2_ TermCode	FoodEx2_ TermName	Origin (country)	Purchase (country)	Water (%)	Part analysed	Colour	α-carotene	β-carotene	β-cryptoxanthin	ζ-carotene	Ref.
Sweet potato	*Ipomea batatas L. var. Brasilia*	A010C	Sweet potatoes	Brazil			tuber	yellowish	nd	1150	nd		100
Sweet Potato	*Ipomoea batatas L.*	A010C	Sweet potatoes	Italy	Italy					7830			25
Sweet potato	*Ipomoea batatas L.*	A010C#F20.A 07QF	Sweet potatoes, PART-CONSUMED-ANALYSED = W/o peel	Vanuatu			without peel	orange, white, pink, yellow, purple, red violet	nd				100
Sweet potato	*Ipomoea batatas L.*	A010C	Sweet potatoes	Spain	Spain		edible part	orange		9488			24
Sweet potato	*Ipomoea batatas L.*	A010C#F20.A 07QF	Sweet potatoes, PART-CONSUMED-ANALYSED = W/o peel	United States	United Kingdom		without peel	-		38100±5150			95
Sweet potato	*Ipomoea batatas L.*	A00ZT	Potatoes	Spain	Spain		edible part			9488			24

Table S8.3.2 Sweet potatoes and similar (A0DPH) (µg/100g) (continuation)

Food name	Scientific name	FoodEx2_ TermCode	FoodEx2_ TermName	Origin (country)	Purchase (country)	Water (%)	Part analysed	Colour	Antheraxanthin	E(v. trans)-α-carotene	E(v. trans)-β-carotene	Lutein	Ref.
Sweet potato	*Ipomea batatas L. var Brasilia*	A010C	Sweet potatoes	Brazil			tuber	yellowish				nd	100
Sweet potato	*Ipomoea batatas L.*	A010C	Sweet potatoes	Italy	Italy							50	25
Sweet potato	*Ipomoea batatas L.*	A010C#F20.A 07QF	Sweet potatoes, PART-CONSUMED-ANALYSED = W/o peel	Vanuatu			without peel	orange, white, pink, yellow, purple, red violet			nd - 14543.1	nd	100

Table S8.3.2 Sweet potatoes and similar (A0DPH) (µg/100g) (continuation)

Food name	Scientific name	FoodEx2_ TermCode	FoodEx2_ TermName	Origin (country)	Purchase (country)	Water (%)	Part analysed	Colour	Antheraxanthin	E(v. trans)-α-carotene	E(v. trans)-β-carotene	Lutein	Ref.
Sweet potato	*Ipomoea batatas L. var. Ejumula*	A010C#F10.A 0F5J	Sweet potatoes, QUALITATIVE-INFO = Orange	Uganda			roots	a* 12.31 ± 1.0			18560 - 32480		105
Sweet potato	*Ipomoea batatas L. var. Ejumula*	A010C#F10.A 0F5J	Sweet potatoes, QUALITATIVE-INFO = Orange	Uganda			roots	a* 12.31 ± 1.0			15920 - 22940		105
Sweet potato	*Ipomoea batatas L. var. Ejumula*	A010C#F10.A 0F5J	Sweet potatoes, QUALITATIVE-INFO = Orange	Uganda			roots	a* 12.31 ± 1.0			20170 - 22290		105
Sweet potato	*Ipomoea batatas L. var. Ejumula*	A010C#F10.A 0F5J	Sweet potatoes, QUALITATIVE-INFO = Orange	Uganda			roots	a* 12.31 ± 1.0			18560 - 24470		105
Sweet potato	*Ipomoea batatas L. var. Ejumula*	A010C#F10.A 0F5J	Sweet potatoes, QUALITATIVE-INFO = Orange	Uganda		< 10	roots	a* 12.31 ± 1.0			27430±2280		105
Sweet potato	*Ipomoea batatas L. var. Ejumula*	A010C#F10.A 0F5J	Sweet potatoes, QUALITATIVE-INFO = Orange	Uganda		<10	roots	a* 12.31 ± 1.0			26460±760		105
Sweet potato	*Ipomoea batatas L. var. Ejumula*	A010C#F10.A 0F5J	Sweet potatoes, QUALITATIVE-INFO = Orange	Uganda		<10	roots	a* 12.31 ± 1.0			25270±1500		105
Sweet potato	*Ipomoea batatas L. var. Sowola 6/94/9*	A010C#F10.A 0F5J	Sweet potatoes, QUALITATIVE-INFO = Orange	Uganda			roots	a* 12.35 ± 1.3			15600 - 25110		105
Sweet potato	*Ipomoea batatas L. var. Sowola 6/94/9*	A010C#F10.A 0F5J	Sweet potatoes, QUALITATIVE-INFO = Orange	Uganda			roots	a* 12.35 ± 1.3			11430 - 19990		105
Sweet potato	*Ipomoea batatas L. var. SPK004*	A010C#F10.A 0F5J	Sweet potatoes, QUALITATIVE-INFO = Orange	Uganda			roots	a* 6.41 ± 2.		4820 - 19170			105
Sweet potato	*Ipomoea batatas L. var. SPK004*	A010C#F10.A 0F5J	Sweet potatoes, QUALITATIVE-INFO = Orange	Uganda			roots	a* 6.41 ± 2.1			7180 - 10960		105
Sweet potato	*Ipomoea batatas L. var. SPK004*	A010C#F10.A 0F5J	Sweet potatoes, QUALITATIVE-INFO = Orange	Uganda			roots	a* 6.41 ± 2.1			3330 - 8940		105
Sweet potato	*Ipomoea batatas L. var. SPK004*	A010C#F10.A 0F5J	Sweet potatoes, QUALITATIVE-INFO = Orange	Uganda			roots	a* 6.41 ± 2.1			6010 - 15870		105

Table S8.3.2 Sweet potatoes and similar (A0DPH) (µg/100g) (continuation)

Food name	Scientific name	FoodEx2_ TermCode	FoodEx2_ TermName	Origin (country)	Purchase (country)	Water (%)	Part analysed	Colour	Antheraxanthin	E(v. trans)-α-carotene	E(v. trans)-β-carotene	Lutein	Ref.
Sweet potato	*Ipomoea batatas L. var. SPK004/1*	A010C#F10.A0F5J	Sweet potatoes, QUALITATIVE-INFO = Orange	Uganda			roots	a* 6.41 ± 2.4			4430 - 19270		105
Sweet potato	*Ipomoea batatas L. var. SPK004/1*	A010C#F10.A0F5J	Sweet potatoes, QUALITATIVE-INFO = Orange	Uganda			roots	a* 6.41 ± 2.1			4150 - 8390		105
Sweet potato	*Ipomoea batatas L. var. SPK004/1*	A010C#F10.A0F5J	Sweet potatoes, QUALITATIVE-INFO = Orange	Uganda			roots	a* 8.39 ± 1.8			2770 - 11970		105
Sweet potato	*Ipomoea batatas L. var. SPK004/1*	A010C#F10.A0F5J	Sweet potatoes, QUALITATIVE-INFO = Orange	Uganda			roots	a* 8.39 ± 1.8			5940 - 16100		105
Sweet potato	*Ipomoea batatas L. var. SPK004/1/1*	A010C#F10.A0F5J	Sweet potatoes, QUALITATIVE-INFO = Orange	Uganda			roots	a* 8.39 ± 1.8			8560 - 21930		105
Sweet potato	*Ipomoea batatas L. var. SPK004/1/1*	A010C#F10.A0F5J	Sweet potatoes, QUALITATIVE-INFO = Orange	Uganda			roots	a* 8.39 ± 1.8			7270 - 12910		105
Sweet potato	*Ipomoea batatas L. var. SPK004/1/1*	A010C#F10.A0F5J	Sweet potatoes, QUALITATIVE-INFO = Orange	Uganda			roots	a* 8.39 ± 1.8			9970 - 13290		105
Sweet potato	*Ipomoea batatas L. var. SPK004/1/1*	A010C#F10.A0F5J	Sweet potatoes, QUALITATIVE-INFO = Orange	Uganda			roots	a* 8.39 ± 1.8			7080 - 19180		105
Sweet potato	*Ipomoea batatas L. var. SPK004/6*	A010C#F10.A0F5J	Sweet potatoes, QUALITATIVE-INFO = Orange	Uganda			roots	a* 13.74 ± 2.0			20630 - 46030		105
Sweet potato	*Ipomoea batatas L. var. SPK004/6*	A010C#F10.A0F5J	Sweet potatoes, QUALITATIVE-INFO = Orange	Uganda			roots	a* 13.74 ± 2.0			22300 - 27540		105
Sweet potato	*Ipomoea batatas L. var. SPK004/6*	A010C#F10.A0F5J	Sweet potatoes, QUALITATIVE-INFO = Orange	Uganda			roots	a* 13.74 ± 2.0			16100 - 40440		105
Sweet potato	*Ipomoea batatas L. var. SPK004/6*	A010C#F10.A0F5J	Sweet potatoes, QUALITATIVE-INFO = Orange	Uganda			roots	a* 13.74 ± 2.0			19490 - 32030		105
Sweet potato	*Ipomoea batatas L. var. SPK004/6/6*	A010C#F10.A0F5J	Sweet potatoes, QUALITATIVE-INFO = Orange	Uganda			roots	a* 12.14 ± 2.6			9790 - 36380		105

Table S8.3.2 Sweet potatoes and similar (A0DPH) (µg/100g) (continuation)

Food name	Scientific name	FoodEx2_ TermCode	FoodEx2_ TermName	Origin (country)	Purchase (country)	Water (%)	Part analysed	Colour	Antheraxanthin	E(v. trans)-α-carotene	E(v. trans)-β-carotene	Lutein	Ref.
Sweet potato	*Ipomoea batatas L. var. SPK004/6/6*	A010C#F10.A0F5J	Sweet potatoes, QUALITATIVE-INFO = Orange	Uganda			roots	a* 12.14 ± 2.6			15220 - 22430		105
Sweet potato	*Ipomoea batatas L. var. SPK004/6/6*	A010C#F10.A0F5J	Sweet potatoes, QUALITATIVE-INFO = Orange	Uganda			roots	a* 12.14 ± 2.6			12640 - 26610		105
Sweet potato	*Ipomoea batatas L. var. SPK004/6/6*	A010C#F10.A0F5J	Sweet potatoes, QUALITATIVE-INFO = Orange	Uganda			roots	a* 12.14 ± 2.6			8080 - 25820		105

Table S8.3.3 Sweet potatoes and similar (A0DPH) (µg/100g) (continuation)

Food name	Scientific name	FoodEx2_ TermCode	FoodEx2_ TermName	Origin (country)	Purchase (country)	Water (%)	Part analysed	Colour	Neoxanthin	Phytoene	Violaxanthin	Z(v. cis)-β-carotene	Zeaxanthin	Ref.
Sweet potato	*Ipomea batatas L.*	A010C	Sweet potatoes	Brazil			tuber	yellowish	nd		nd		nd	100
Sweet potato	*Ipomoea batatas L.*	A010C#F20.A07QF	Sweet potatoes, PART-CONSUMED-ANALYSED = W/o peel	Vanuatu		-	roots without peel	orange, white, pink, yellow, purple, red violet				nd	nd	100
Sweet potato	*Ipomoea batatas L. var. Ejumula*	A010C#F10.A0F5J	Sweet potatoes, QUALITATIVE-INFO = Orange	Uganda			roots	a* 12.31 ± 1.0				1240±240		105
Sweet potato	*Ipomoea batatas L. var. Ejumula*	A010C#F10.A0F5J	Sweet potatoes, QUALITATIVE-INFO = Orange	Uganda			roots	a* 12.31 ± 1.0				890±150		105
Sweet potato	*Ipomoea batatas L. var. Ejumula*	A010C#F10.A0F5J	Sweet potatoes, QUALITATIVE-INFO = Orange	Uganda			roots	a* 12.31 ± 1.0				1150±180		105

Table S8.3.3 Sweet potatoes and similar (A0DPH) (µg/100g) (continuation)

Food name	Scientific name	FoodEx2_ TermCode	FoodEx2_ TermName	Origin (country)	Purchase (country)	Water (%)	Part analysed	Colour	Neoxanthin	Phytoene	Violaxanthin	Z(v. cis)-β-carotene	Zeaxanthin	Ref.
Sweet potato	*Ipomoea batatas L. var. Ejumula*	A010C#F10. A0F5J	Sweet potatoes, QUALITATIVE-INFO = Orange	Uganda		< 10 %	roots	a* 12.31 ± 1.0				350±30		105
Sweet potato	*Ipomoea batatas L. var. Ejumula*	A010C#F10. A0F5J	Sweet potatoes, QUALITATIVE-INFO = Orange	Uganda		<10 %	roots	a* 12.31 ± 1.0				200±130		105
Sweet potato	*Ipomoea batatas L. var. Ejumula*	A010C#F10. A0F5J	Sweet potatoes, QUALITATIVE-INFO = Orange	Uganda		<10 %	roots	a* 12.31 ± 1.0				90±20		105
Sweet potato	*Ipomoea batatas L. var. Sowola 6/94/9*	A010C#F10. A0F5J	Sweet potatoes, QUALITATIVE-INFO = Orange	Uganda			roots	a* 12.35 ± 1.3				1210±250		105
Sweet potato	*Ipomoea batatas L. var. SPK004*	A010C#F10. A0F5J	Sweet potatoes, QUALITATIVE-INFO = Orange	Uganda			roots	a* 6.41 ± 2.1				870±180		105
Sweet potato	*Ipomoea batatas L. var. SPK004*	A010C#F10. A0F5J	Sweet potatoes, QUALITATIVE-INFO = Orange	Uganda			roots	a* 6.41 ± 2.1				680±210		105
Sweet potato	*Ipomoea batatas L. var. SPK004*	A010C#F10. A0F5J	Sweet potatoes, QUALITATIVE-INFO = Orange	Uganda			roots	a* 6.41 ± 2.1				650±210		105
Sweet potato	*Ipomoea batatas L. var. SPK004/1*	A010C#F10. A0F5J	Sweet potatoes, QUALITATIVE-INFO = Orange	Uganda			roots	a* 6.41 ± 2.1				470±50		105
Sweet potato	*Ipomoea batatas L. var. SPK004/1*	A010C#F10. A0F5J	Sweet potatoes, QUALITATIVE-INFO = Orange	Uganda			roots	a* 8.39 ± 1.8				450±270		105
Sweet potato	*Ipomoea batatas L. var. SPK004/1*	A010C#F10. A0F5J	Sweet potatoes, QUALITATIVE-INFO = Orange	Uganda			roots	a* 8.39 ± 1.8				900±290		105
Sweet potato	*Ipomoea batatas L. var. SPK004/1/1*	A010C#F10. A0F5J	Sweet potatoes, QUALITATIVE-INFO = Orange	Uganda			roots	a* 8.39 ± 1.8				640±200		105

Table S8.3.3 Sweet potatoes and similar (A0DPH) (µg/100g) (continuation)

Food name	Scientific name	FoodEx2_ TermCode	FoodEx2_ TermName	Origin (country)	Purchase (country)	Water (%)	Part analysed	Colour	Neoxanthin	Phytoene	Violaxanthin	Z(v. cis)-β-carotene	Zeaxanthin	Ref.
Sweet potato	*Ipomoea batatas L. var. SPK004/1/1*	A010C#F10. A0F5J	Sweet potatoes, QUALITATIVE-INFO = Orange	Uganda			roots	a* 8.39 ± 1.8				660±130		105
Sweet potato	*Ipomoea batatas L. var. SPK004/1/1*	A010C#F10. A0F5J	Sweet potatoes, QUALITATIVE-INFO = Orange	Uganda			roots	a* 8.39 ± 1.8				360±90		105
Sweet potato	*Ipomoea batatas L. var. SPK004/6*	A010C#F10. A0F5J	Sweet potatoes, QUALITATIVE-INFO = Orange	Uganda			roots	a* 13.74 ± 2.0				1810±180		105
Sweet potato	*Ipomoea batatas L. var. SPK004/6*	A010C#F10. A0F5J	Sweet potatoes, QUALITATIVE-INFO = Orange	Uganda			roots	a* 13.74 ± 2.0				1580±650		105
Sweet potato	*Ipomoea batatas L. var. SPK004/6*	A010C#F10. A0F5J	Sweet potatoes, QUALITATIVE-INFO = Orange	Uganda			roots	a* 13.74 ± 2.0				2350±290		105
Sweet potato	*Ipomoea batatas L. var. SPK004/6/6*	A010C#F10. A0F5J	Sweet potatoes, QUALITATIVE-INFO = Orange	Uganda			roots	a* 12.14 ± 2.6				1590±260		105
Sweet potato	*Ipomoea batatas L. var. SPK004/6/6*	A010C#F10. A0F5J	Sweet potatoes, QUALITATIVE-INFO = Orange	Uganda			roots	a* 12.14 ± 2.6				1570±690		105
Sweet potato	*Ipomoea batatas L. var. SPK004/6/6*	A010C#F10. A0F5J	Sweet potatoes, QUALITATIVE-INFO = Orange	Uganda			roots	a* 12.14 ± 2.6				1060±410		105

Table S8.4.1 Yams and similar (A04JY) (µg/100g)

Food name	Scientific name	FoodEx2_ TermCode	FoodEx2_ TermName	Origin (country)	Purchase (country)	Water (%)	Part analysed	Colour	α-carotene	β-carotene	β-cryptoxanthin	ζ-carotene	Ref.
Yams	*Dioscorea alata L.*	A010D	Yams	Vanuatu			tubers	purple, white, red, pink, red violet, yellow, orange	nd - tr.				100
Yams	*Dioscorea bulbifera*	A010D	Yams	Vanuatu			bulbils	yellow, purple, yellow-green, red	nd - tr.				100
Yams	*Dioscorea bulbifera*	A010D	Yams	Vanuatu			tubers	yellow, purple, white	nd - tr.				100
Yams	*Dioscorea cayenensis*	A010D#F10.A0F5H	Yams, QUALITATIVE-INFO = yellow	Vanuatu			tubers	yellow, white	nd				100
Yams	*Dioscorea esculenta*	A010D	Yams	Vanuatu			tubers	pink, white	nd - tr.				100
Yams	*Dioscorea pentaphylla*	A010D	Yams	Vanuatu			tubers	white	nd - tr.				100

Table S8.4.2 Yams and similar (A04JY) (µg/100g) (continuation)

Food name	Scientific name	FoodEx2_ TermCode	FoodEx2_ TermName	Origin (country)	Purchase (country)	Water (%)	Part analysed	Colour	Antheraxanthin	E(v. trans)-α-carotene	E(v. trans)-β-carotene	Lutein	Ref.
Yams	*Dioscorea alata L.*	A010D	Yams	Vanuatu			tubers	purple, white, red, pink, red violet, yellow, orange			tr.- 16.1	nd - tr.	100
Yams	*Dioscorea bulbifera*	A010D	Yams	Vanuatu			bulbils	yellow, purple, yellow-green, red			nd - 17.7	51.2 - 172.1	100
Yams	*Dioscorea bulbifera*	A010D	Yams	Vanuatu			tubers	yellow, purple, white			nd - 11.1	12.4 - 67.9	100

Table S8.4.2 Yams and similar (A04JY) (µg/100g) (continuation)

Food name	Scientific name	FoodEx2_ TermCode	FoodEx2_ TermName	Origin (country)	Purchase (country)	Water (%)	Part analysed	Colour	Antheraxanthin	E(v. trans)-α-carotene	E(v. trans)-β-carotene	Lutein	Ref.
Yams	*Dioscorea cayenensis*	A010D#F10.A0F5H	Yams, QUALITATIVE-INFO = yellow	Vanuatu			tubers	yellow, white			tr. - 10.1	nd- tr.	100
Yams	*Dioscorea esculenta*	A010D	Yams	Vanuatu			tubers	pink, white			tr. - 6.6	tr.	100
Yams	*Dioscorea pentaphylla*	A010D	Yams	Vanuatu			tubers	white			nd - tr.	nd	100

Table S8.4.3 Yams and similar (A04JY) (µg/100g) (continuation)

Food name	Scientific name	FoodEx2_ TermCode	FoodEx2_ TermName	Origin (country)	Purchase (country)	Water (%)	Part analysed	Colour	Neoxanthin	Phytoene	Violaxanthin	Z(v. cis)-β-carotene	Zeaxanthin	Ref.
Yams	*Dioscorea alata L.*	A010D	Yams	Vanuatu			tubers	purple, white, red, pink, red violet, yellow, orange		nd - tr.		nd	nd - tr.	100
Yams	*Dioscorea bulbifera*	A010D	Yams	Vanuatu			bulbils	yellow, purple, yellow-green, red		nd - 680.2		nd	nd - tr.	100
Yams	*Dioscorea bulbifera*	A010D	Yams	Vanuatu			tubers	yellow, purple, white		nd - 488.2		nd	nd - tr.	100
Yams	*Dioscorea cayenensis*	A010D#F10.A0F5H	Yams, QUALITATIVE-INFO = yellow	Vanuatu			tubers	yellow, white		nd			nd - tr.	100
Yams	*Dioscorea esculenta*	A010D	Yams	Vanuatu			tubers	pink, white		nd		nd - tr.	nd - tr.	100
Yams	*Dioscorea pentaphylla*	A010D	Yams	Vanuatu			tubers	white		tr. - 18.6		nd	nd	100

Table S8.5.1 Sugar plants (A010R) (µg/100g)

Food name	Scientific name	FoodEx2_TermCode	FoodEx2_TermName	Origin (country)	Purchase (country)	Water (%)	Part analysed	Colour	α-carotene	β-carotene	β-cryptoxanthin	ζ-carotene	Ref.
Chicory	*Cichorium intybus cv. Anivip*	A010Z	Chicory roots	Slovenia	Slovenia	88.1±2	edible part			7310±1120			97
Chicory	*Cichorium intybus cv. Monivip*	A010Z	Chicory roots	Slovenia	Slovenia	89.2±3.4	edible part			3940±650			97

Table S8.5.2 Sugar plants (A010R) (µg/100g) (continuation)

Food name	Scientific name	FoodEx2_TermCode	FoodEx2_TermName	Origin (country)	Purchase (country)	Water (%)	Part analysed	Colour	Antheraxanthin	E(v. trans)-α-carotene	E(v. trans)-β-carotene	Lutein	Ref.
Chicory	Cichorium intybus cv. Anivip	A010Z	Chicory roots	Slovenia	Slovenia	88.1±2	edible part		520±160			5910±840	97
Chicory	Cichorium intybus cv. Monivip	A010Z	Chicory roots	Slovenia	Slovenia	89.2±3.4	edible part		380±120			3870±450	97

Table S8.5.3 Sugar plants (A010R) (µg/100g) (continuation)

Food name	Scientific name	FoodEx2_TermCode	FoodEx2_TermName	Origin (country)	Purchase (country)	Water (%)	Part analysed	Colour	Neoxanthin	Phytoene	Violaxanthin	Z(v. cis)-β-carotene	Zeaxanthin	Ref.
Chicory	Cichorium intybus cv. Anivip	A010Z	Chicory roots	Slovenia	Slovenia	88.1±2.	edible part		1070±260		1670±440		90±20	97
Chicory	Cichorium intybus cv. Monivip	A010Z	Chicory roots	Slovenia	Slovenia	89.2±3.4	edible part		410±260		690±270		70±10	97

Table S9. Vegetables and vegetable products (A00FJ)

Table S9.1.1 Lamb's lettuces and similar (A0DLC) (µg/100g)

Food name	Scientific name	FoodEx2_ TermCode	FoodEx2_ TermName	Origin (country)	Purchase (country)	Water (%)	Process	Saponification	Part analysed	Colour	α-carotene	β-carotene	β-cryptoxanthin	ζ-carotene	Antheraxanthin	Ref.
Lamb’s lettuce	*Valerianella locusta L.*	A00KT	Lamb's lettuces	Spain	Spain					green		2655				24
Lamb's lettuce	*Valerianella locusta L.*	A00KT	Lamb's lettuces	Germany	Germany	92.6					80	3220	100			53
Lamb’s lettuce	*Valerianella locusta L.*	A00KT	Lamb's lettuces	Spain	Spain					dark green		2655				24

Table S9.1.2 Lamb's lettuces and similar (A0DLC) (µg/100g) (continuation)

Food name	Scientific name	FoodEx2_ TermCode	FoodEx2_ TermName	Origin (country)	Purchase (country)	Water (%)	Process	Saponification	Part analysed	Colour	Lutein	Luteoxanthin	Lycopene	Neoxanthin	Phytoene	Ref.
Lamb's lettuce	*Valerianella locusta*	A00KT	Lamb's lettuces	Spain	Spain					green	4357					24
Lamb's lettuce	*Valerianella locusta*	A00KT	Lamb's lettuces	Germany	Germany	92.6					9650			210		53
Lamb's lettuce	*Valerianella locusta*	A00KT	Lamb's lettuces	Spain	Spain					dark green	4357					24

Table S9.1.3 Lamb's lettuces and similar (A0DLC) (µg/100g) (continuation)

Food name	Scientific name	FoodEx2_ TermCode	FoodEx2_ TermName	Origin (country)	Purchase (country)	Water (%)	Process	Saponification	Part analysed	Colour	Phytofluene	Violaxanthin	Z(v. cis)-lycopene	Z(v. cis)-β-carotene	Z(v. cis)-β-cryptoxanthin	Zeaxanthin	Ref.
Lamb's lettuce	*Valerianella locusta*	A00KT	Lamb's lettuces	Germany	Germany	92.6						1640					53

Table S9.2.1 Lettuces and similar (A0DLB) (µg/100g)

Food name	Scientific name	FoodEx2_ TermCode	FoodEx2_ TermName	Origin (country)	Purchase (country)	Water (%)	Process	Saponification	Part analysed	Colour	α-carotene	β-carotene	β-cryptoxanthin	ζ-carotene	Antheraxanthin	Ref.
Lettuce	*Lactuca indica*	A00KX	Lettuces (generic)	India					without stems			42720				98
Lettuce	*Lactuca sativa L.*	A00KX	Lettuces (generic)	Germany	Germany	94.5					40	1290	30			53
Lettuce	*Lactuca sativa L.*	A00KX	Lettuces (generic)	Brazil					leaves	green	nd	1550	nd			109
Lettuce	*Lactuca sativa L.*	A00KX	Lettuces (generic)	Spain	Spain				leaves	green		3300±220				101
Lettuce	*Lactuca sativa L.*	A00KX	Lettuces (generic)	Spain	Spain				leaves	green		3330±140				101
Lettuce	*Lactuca sativa L.*	A00KX	Lettuces (generic)	Spain	United Kingdom				white stalk removed			22570± 4040				95
Lettuce	*Lactuca sativa L.*	A00KX	Lettuces (generic)	Italy	Italy							870–2960				25
Lettuce	*Lactuca sativa L.*	A00KX	Lettuces (generic)	Spain	Spain	95			leaves	green		172±8				24
Lettuce	*Lactuca sativa L. cv. Carrascoy*	A00KX	Lettuces (generic)	Spain	Spain				leaves	green		2640±300				101
Lettuce	*Lactuca sativa L. cv. Iceberg*	A00KZ	Crisp lettuces	Germany	Germany	95.5						330	6			53
Lettuce	*Lactuca sativa L. cv. Iceberg*	A00KZ	Crisp lettuces	Spain	Spain					green		48				24
Lettuce	*Lactuca sativa L. cv. Iceberg*	A00KZ	Crisp lettuces	Spain	Spain							48				24
Lettuce	*Lactuca sativa L. cv. Iceberg*	A00KZ	Crisp lettuces	Spain	Spain	96			leaves	green		48±2				24
Lettuce	*Lactuca sativa L. cv. Romaine*	A00LC	Romaines	Spain	Spain					green		172				24
Lettuce	*Lactuca sativa L. cv. Romaine*	A00LC	Romaines	Spain	Spain							172				24

Table S9.2.1 Lettuces and similar (A0DLB) (µg/100g) (continuation)

Food name	Scientific name	FoodEx2_ TermCode	FoodEx2_ TermName	Origin (country)	Purchase (country)	Water (%)	Process	Saponification	Part analysed	Colour	α-carotene	β-carotene	β-cryptoxanthin	ζ-carotene	Antheraxanthin	Ref.
Lettuce	*Lactuca sativa L. cv. Romaine*	A00LC	Romaines	USA				yes	leaves			190000				103
Lettuce	*Lactuca sativa L. cv. Romaine*	A00LC	Romaines	USA				yes	leaves			342000				103
Lettuce	*Lactuca sativa L. cv. Romaine*	A00LC	Romaines	USA				yes	leaves			513000				103
Lettuce	*Lactuca sativa L. var. Aitana*	A00KX	Lettuces (generic)	Spain	Spain				leaves	green		3490±230				101
Lettuce	*Lactuca sativa L. var. Alhama*	A00KX	Lettuces (generic)	Spain	Spain				leaves	green		3390±280				101
Lettuce	*Lactuca sativa L. var. Collado*	A00KX	Lettuces (generic)	Spain	Spain				leaves	green		3290±90				101
Lettuce	*Lactuca sativa L. var. Espuña*	A00KX	Lettuces (generic)	Spain	Spain				leaves	green		2460±500				101
Lettuce	*Lactuca sativa L. var. Etna*	A00KX	Lettuces (generic)	Spain	Spain				leaves	green		1880±220				101
Lettuce	*Lactuca sativa L. var. Ferro*	A00KX	Lettuces (generic)	Spain	Spain				leaves	green		2030±340				101
Lettuce	*Lactuca sativa L. var. Isasa*	A00KX	Lettuces (generic)	Spain	Spain				leaves	green		2010±130				101
Lettuce	*Lactuca sativa L. var. Maite*	A00KX	Lettuces (generic)	Spain	Spain				leaves	green		2270±290				101
Lettuce	*Lactuca sativa L. var. Marta*	A00KX	Lettuces (generic)	Spain	Spain				leaves	green		4180±590				101
Lettuce	*Lactuca sativa L. var. Petra*	A00KX	Lettuces (generic)	Spain	Spain				leaves	green		1950±100				101

Table S9.2.1 Lettuces and similar (A0DLB) (µg/100g) (continuation)

Food name	Scientific name	FoodEx2_ TermCode	FoodEx2_ TermName	Origin (country)	Purchase (country)	Water (%)	Process	Saponification	Part analysed	Colour	α-carotene	β-carotene	β-cryptoxanthin	ζ-carotene	Antheraxanthin	Ref.
Lettuce	*Lactuca sativa L. var. Ricote*	A00KX	Lettuces (generic)	Spain	Spain				leaves	green		2050±100				101
Lettuce	*Lactuca sativa L. var. Sandra*	A00KX	Lettuces (generic)	Spain	Spain				leaves	green		2070±410				101
Lettuce	*Lactuca sativa L. var. Urbión*	A00KX	Lettuces (generic)	Spain	Spain				leaves	green		2060±50				101
Lettuce	*Lactuca sativa var. Capitata Iceberg*	A00KZ	Crisp lettuces	USA				no			0		172			28
Lettuce	*Lactuca sativa L. var. longifolia*	A00LC	Romaines	USA				no			0		0			28

Table S9.2.2 Lettuces and similar (A0DLB) (µg/100g) (continuation)

Food name	Scientific name	FoodEx2_ TermCode	FoodEx2_ TermName	Origin (country)	Purchase (country)	Water (%)	Process	Saponification	Part analysed	Colour	Capsanthin	Capsorubin	Cucurbitaxanthin	E(v. trans)-α-carotene	E(v. trans)-β-carotene	Ref.
Lettuce	*Lactuca sativa var. Capitata 'Iceberg'*	A00KZ	Crisp lettuces	USA				no							0	28
Lettuce	*Lactuca sativa L. var. longifolia*	A00LC	Romaines	USA				no							2730	28

Table S9.2.3 Lettuces and similar (A0DLB) (µg/100g) (continuation)

Food name	Scientific name	FoodEx2_ TermCode	FoodEx2_ TermName	Origin (country)	Purchase (country)	Water (%)	Process	Saponification	Part analysed	Colour	E(v. trans)-β-cryptoxanthin	E(v. trans)-lutein	E(v. trans)-lycopene	E(v. trans)-zeaxanthin	Lactucaxanthin	Ref.
Lettuce	*Lactuca sativa L.*	A00KX	Lettuces (generic)	Spain	Spain				leaves	green					930±80	101
Lettuce	*Lactuca sativa L.*	A00KX	Lettuces (generic)	Spain	Spain				leaves	green					840±30	101
Lettuce	*Lactuca sativa L. cv. Carrascoy*	A00KX	Lettuces (generic)	Spain	Spain				leaves	green					680±30	101
Lettuce	*Lactuca sativa L. var. Aitana*	A00KX	Lettuces (generic)	Spain	Spain				leaves	green					820±70	101
Lettuce	*Lactuca sativa L. var. Alhama*	A00KX	Lettuces (generic)	Spain	Spain				leaves	green					860±80	101
Lettuce	*Lactuca sativa L. var. Collado*	A00KX	Lettuces (generic)	Spain	Spain				leaves	green					810±70	101
Lettuce	*Lactuca sativa L. var. Espuña*	A00KX	Lettuces (generic)	Spain	Spain				leaves	green					590±110	101
Lettuce	*Lactuca sativa L. var. Etna*	A00KX	Lettuces (generic)	Spain	Spain				leaves	green					580±60	101
Lettuce	*Lactuca sativa L. var. Ferro*	A00KX	Lettuces (generic)	Spain	Spain				leaves	green					460±60	101
Lettuce	*Lactuca sativa L. var. Isasa*	A00KX	Lettuces (generic)	Spain	Spain				leaves	green					400±60	101
Lettuce	*Lactuca sativa L. var. Maite*	A00KX	Lettuces (generic)	Spain	Spain				leaves	green					650±50	101
Lettuce	*Lactuca sativa L. var. Marta*	A00KX	Lettuces (generic)	Spain	Spain				leaves	green					1110±70	101
Lettuce	*Lactuca sativa L. var. Petra*	A00KX	Lettuces (generic)	Spain	Spain				leaves	green					470±20	101
Lettuce	*Lactuca sativa L. var. Ricote*	A00KX	Lettuces (generic)	Spain	Spain				leaves	green					500±70	101
Lettuce	*Lactuca sativa L. var. Sandra*	A00KX	Lettuces (generic)	Spain	Spain				leaves	green					480±70	101
Lettuce	*Lactuca sativa L. var. Urbión*	A00KX	Lettuces (generic)	Spain	Spain				leaves	green					530±50	101
Lettuce	*Lactuca sativa var. Capitata 'Iceberg'*	A00KZ	Crisp lettuces	USA				no				171		12		28
Lettuce	Lactuca sativa L. var. longifolia	A00LC	Romaines	USA				no				3824		0		28

Table S9.2.4 Lettuces and similar (A0DLB) (µg/100g) (continuation)

Food name	Scientific name	FoodEx2_ TermCode	FoodEx2_ TermName	Origin (country)	Purchase (country)	Water (%)	Process	Saponification	Part analysed	Colour	Lutein	Luteoxanthin	Lycopene	Neoxanthin	Phytoene	Ref.
Lettuce	Lactuca sativa L	A00KX	Lettuces (generic)	Spain	United Kingdom				white stalk removed		12390± 880			5180± 470		95
Lettuce	Lactuca sativa L.	A00KX	Lettuces (generic)	Germany	Germany	94.5					2920			140		53
Lettuce	Lactuca sativa L.	A00KX	Lettuces (generic)	Brazil					leaves	green	1430		nd	760		109
Lettuce	Lactuca sativa L.	A00KX	Lettuces (generic)	Spain	Spain				leaves	green	1410± 110			450±60		101
Lettuce	Lactuca sativa L.	A00KX	Lettuces (generic)	Spain	Spain				leaves	green	1270±40			390±10		101
Lettuce	Lactuca sativa L.	A00KX	Lettuces (generic)	India					without stems		87120			5750		98
Lettuce	Lactuca sativa L.	A00KX	Lettuces (generic)	Italy	Italy						1000–4780					25
Lettuce	Lactuca sativa L.	A00KX	Lettuces (generic)	Spain	Spain	95			leaves	green	340±17					24
Lettuce	Lactuca sativa L. cv Iceberg	A00KZ	Crisp lettuces	Germany	Germany	95.5					690			33		53
Lettuce	Lactuca sativa L. cv. Carrascoy	A00KX	Lettuces (generic)	Spain	Spain				leaves	green	1170±90			340±30		101
Lettuce	Lactuca sativa L. cv. Iceberg	A00KZ	Crisp lettuces	Spain	Spain						140					24
Lettuce	Lactuca sativa L. cv. Iceberg	A00KZ	Crisp lettuces	Spain	Spain	96			leaves	green	140±3					24
Lettuce	Lactuca sativa L. cv. Icerberg	A00KZ	Crisp lettuces	Spain	Spain					green	140					24
Lettuce	Lactuca sativa L. cv. Romaine	A00LC	Romaines	USA				yes	leaves		160000					103
Lettuce	Lactuca sativa L. cv. Romaine	A00LC	Romaines	USA				yes	leaves		210000					103
Lettuce	Lactuca sativa L. cv. Romaine	A00LC	Romaines	USA				yes	leaves		270000					103
Lettuce	Lactuca sativa L. var. Aitana	A00KX	Lettuces (generic)	Spain	Spain				leaves	green	1390± 120			460±40		101
Lettuce	Lactuca sativa L. var. Alhama	A00KX	Lettuces (generic)	Spain	Spain				leaves	green	1340± 100			410±30		101
Lettuce	Lactuca sativa L. var. Collado	A00KX	Lettuces (generic)	Spain	Spain				leaves	green	1160±20			350±30		101

Table S9.2.4 Lettuces and similar (A0DLB) (µg/100g) (continuation)

Food name	Scientific name	FoodEx2_ TermCode	FoodEx2_ TermName	Origin (country)	Purchase (country)	Water (%)	Process	Saponification	Part analysed	Colour	Lutein	Luteoxanthin	Lycopene	Neoxanthin	Phytoene	Ref.
Lettuce	Lactuca sativa L. var. Espuña	A00KX	Lettuces (generic)	Spain	Spain				leaves	green	1000± 210			320±60		101
Lettuce	Lactuca sativa L. var. Etna	A00KX	Lettuces (generic)	Spain	Spain				leaves	green	840±70			260±20		101
Lettuce	Lactuca sativa L. var. Ferro	A00KX	Lettuces (generic)	Spain	Spain				leaves	green	780±110			250±40		101
Lettuce	Lactuca sativa L. var. Isasa	A00KX	Lettuces (generic)	Spain	Spain				leaves	green	770±50			230±30		101
Lettuce	Lactuca sativa L. var. Maite	A00KX	Lettuces (generic)	Spain	Spain				leaves	green	970±130			310±30		101
Lettuce	Lactuca sativa L. var. Marta	A00KX	Lettuces (generic)	Spain	Spain				leaves	green	1490± 200			500±70		101
Lettuce	Lactuca sativa L. var. Petra	A00KX	Lettuces (generic)	Spain	Spain				leaves	green	780±70			250±20		101
Lettuce	Lactuca sativa L. var. Ricote	A00KX	Lettuces (generic)	Spain	Spain				leaves	green	780±30			260±20		101
Lettuce	Lactuca sativa L. var. Urbión	A00KX	Lettuces (generic)	Spain	Spain				leaves	green	840±40			270±10		101
Lettuce	Lactuca sativa L. var.Sandra	A00KX	Lettuces (generic)	Spain	Spain				leaves	green	870±130			270±40		101

Table S9.2.5 Lettuces and similar (A0DLB) (µg/100g) (continuation)

Food name	Scientific name	FoodEx2_ TermCode	FoodEx2_ TermName	Origin (country)	Purchase (country)	Water (%)	Process	Saponification	Part analysed	Colour	Phytofluene	Violaxanthin	Z(v. cis)-lycopene	Z(v. cis)-β-carotene	Z(v. cis)-β-cryptoxanthin	Zeaxanthin	Ref.
Lettuce	*Lactuca sativa L*	A00KX	Lettuces (generic)	Spain	United Kingdom				white stalk removed			16710± 1430					95
Lettuce	*Lactuca sativa L.*	A00KX	Lettuces (generic)	Brazil					leaves	green		1870				nd	109
Lettuce	*Lactuca sativa L.*	A00KX	Lettuces (generic)	Germany	Germany	94.5						2360					53

Table S9.2.5 Lettuces and similar (A0DLB) (µg/100g) (continuation)

Food name	Scientific name	FoodEx2_ TermCode	FoodEx2_ TermName	Origin (country)	Purchase (country)	Water (%)	Process	Saponification	Part analysed	Colour	Phytofluene	Violaxanthin	Z(v. cis)-lycopene	Z(v. cis)-β-carotene	Z(v. cis)-β-cryptoxanthin	Zeaxanthin	Ref.
Lettuce	*Lactuca sativa L.*	A00KX	Lettuces (generic)	Spain	Spain				leaves	green		690±70					101
Lettuce	*Lactuca sativa L.*	A00KX	Lettuces (generic)	Spain	Spain				leaves	green		620±30					101
Lettuce	*Lactuca sativa L.*	A00KX	Lettuces (generic)	India					without stems			29090					98
Lettuce	*Lactuca sativa L. cv. Carrascoy*	A00KX	Lettuces (generic)	Spain	Spain				leaves	green		590±30					101
Lettuce	*Lactuca sativa L. cv. Iceberg*	A00KZ	Crisp lettuces	Germany	Germany	95.5			edible part			330					53
Lettuce	*Lactuca sativa L. var. Aitana*	A00KX	Lettuces (generic)	Spain	Spain				leaves	green		750±50					101
Lettuce	*Lactuca sativa L. var. Alhama*	A00KX	Lettuces (generic)	Spain	Spain				leaves	green		620±40					101
Lettuce	*Lactuca sativa L. var. Collado*	A00KX	Lettuces (generic)	Spain	Spain				leaves	green		530±30					101
Lettuce	*Lactuca sativa L. var. Espuña*	A00KX	Lettuces (generic)	Spain	Spain				leaves	green		550±10					101
Lettuce	*Lactuca sativa L. var. Etna*	A00KX	Lettuces (generic)	Spain	Spain				leaves	green		430±30					101
Lettuce	*Lactuca sativa L. var. Ferro*	A00KX	Lettuces (generic)	Spain	Spain				leaves	green		420±50					101
Lettuce	*Lactuca sativa L. var. Isasa*	A00KX	Lettuces (generic)	Spain	Spain				leaves	green		500±120					101
Lettuce	*Lactuca sativa L. var. Maite*	A00KX	Lettuces (generic)	Spain	Spain				leaves	green		530±70					101
Lettuce	*Lactuca sativa L. var. Marta*	A00KX	Lettuces (generic)	Spain	Spain				leaves	green		750±60					101

Table S9.2.5 Lettuces and similar (A0DLB) (µg/100g) (continuation)

Food name	Scientific name	FoodEx2_ TermCode	FoodEx2_ TermName	Origin (country)	Purchase (country)	Water (%)	Process	Saponification	Part analysed	Colour	Phytofluene	Violaxanthin	Z(v. cis)-lycopene	Z(v. cis)-β-carotene	Z(v. cis)-β-cryptoxanthin	Zeaxanthin	Ref.
Lettuce	Lactuca sativa L. var. Petra	A00KX	Lettuces (generic)	Spain	Spain				leaves	green		460±10					101
Lettuce	Lactuca sativa L. var. Ricote	A00KX	Lettuces (generic)	Spain	Spain				leaves	green		450±30					101
Lettuce	Lactuca sativa L. var. Sandra	A00KX	Lettuces (generic)	Spain	Spain				leaves	green		490±60					101
Lettuce	Lactuca sativa L. var. Urbión	A00KX	Lettuces (generic)	Spain	Spain				leaves	green		480±30					101
Lettuce	Lactuca sativa var. Capitata Iceberg	A00KZ	Crisp lettuces	USA				no						0			28
Lettuce	Lactuca sativa L. var. longifolia	A00LC	Romaines	USA				no						509			28

Table S9.3.1 Escaroles and similar (A00LD) (µg/100g)

Food name	Scientific name	FoodEx2_ TermCode	FoodEx2_ TermName	Origin (country)	Purchase (country)	Water (%)	Process	Saponification	Part analysed	Colour	α-carotene	β-carotene	β-cryptoxanthin	ζ-carotene	Antheraxanthin	Ref.
Escarole	*Cichorium endivia L.*	A00LE	Escaroles	Italy	Italy							1340–4350				25
Escarole	*Cichorium endívia L.*	A00LE	Escaroles	Germany	Germany	95.9						890				53
Escarole	*Cichorium endívia L.*	A00LE	Escaroles	USA				no			0		3			28
Escarole	*Cichorium endivia L. Valdena*	A00LE	Escaroles	Brazil					leaves	green	nd	2795	nd			109

Table S9.3.2 Escaroles and similar (A00LD) (µg/100g) (continuation)

Food name	Scientific name	FoodEx2_ TermCode	FoodEx2_ TermName	Origin (country)	Purchase (country)	Water (%)	Process	Saponification	Part analysed	Colour	Capsanthin	Capsorubin	Cucurbitaxanthin	E(v. trans)-α-carotene	E(v. trans)-β-carotene	Ref.
Escarole	*Cichorium endívia L.*	A00LE	Escaroles	USA				no							49	28

Table S9.3.3 Escaroles and similar (A00LD) (µg/100g) (continuation)

Food name	Scientific name	FoodEx2_ TermCode	FoodEx2_ TermName	Origin (country)	Purchase (country)	Water (%)	Process	Saponification	Part analysed	Colour	E(v. trans)-β-cryptoxanthin	E(v. trans)-lutein	E(v. trans)-lycopene	E(v. trans)-zeaxanthin	Lactucaxanthin	Ref.
Escarole	*Cichorium endívia L.*	A00LE	Escaroles	USA				no				399		3		28

Table S9.3.4 Escaroles and similar (A00LD) (µg/100g) (continuation)

Food name	Scientific name	FoodEx2_ TermCode	FoodEx2_ TermName	Origin (country)	Purchase (country)	Water (%)	Process	Saponification	Part analysed	Colour	Lutein	Luteoxanthin	Lycopene	Neoxanthin	Phytoene	Ref.
Escarole	*Cichorium endivia L.*	A00LE	Escaroles	Italy	Italy						2060–6150					25
Escarole	*Cichorium endívia L.*	A00LE	Escaroles	Germany	Germany	95.9					2080					51
Escarole	*Cichorium endivia L. Valdena*	A00LE	Escaroles	Brazil					leaves	green	3895		nd	1425		109

Table S9.3.5 Escaroles and similar (A00LD) (µg/100g) (continuation)

Food name	Scientific name	FoodEx2_ TermCode	FoodEx2_ TermName	Origin (country)	Purchase (country)	Water (%)	Process	Saponification	Part analysed	Colour	Phytofluene	Violaxanthin	Z(v. cis)-lycopene	Z(v. cis)-β-carotene	Z(v. cis)-β-cryptoxanthin	Zeaxanthin	Ref.
Escarole	*Cichorium endívia L.*	A00LE	Escaroles	Germany	Germany	95.9			edible part			430					53
Escarole	*Cichorium endívia L.*	A00LE	Escaroles	USA				no						8			28
Escarole	*Cichorium endivia L. Valdena*	A00LE	Escaroles	Brazil					leaves	green		2065				nd	109

Table S9.4.1 Roman rocket and similar (A00LM) (µg/100g)

Food name	Scientific name	FoodEx2_ TermCode	FoodEx2_ TermName	Origin (country)	Purchase (country)	Water (%)	Process	Saponification	Part analysed	Colour	α-carotene	β-carotene	β-cryptoxanthin	ζ-carotene	Antheraxanthin	Ref.
Roman rocket	*Eruca sativa Mill.*	A00LN	Roman rocket	Brazil					leaves	green	nd	2840	nd			109
Roman rocket	*Eruca sativa Mill.*	A00LN	Roman rocket	Slovenia	Slovenia	86.8± 2.9					280±40	7960± 1430			840±210	97
Wall rocket	*Diplotaxis tenuifolia L.*	A00LP	Wall rocket	Slovenia	Slovenia	85.7± 2.6					170±60	7010± 1040			390±20	97
Wall rocket	*Diplotaxis tenuifolia L.*	A00LP	Wall rocket	Spain	Spain					green		3575				24
Wall rocket	*Diplotaxis tenuifolia L.*	A00LP	Wall rocket	Spain	Spain					green		3575				24

Table S9.4.2 Roman rocket and similar (A00LM) (µg/100g) (continuation)

Food name	Scientific name	FoodEx2_ TermCode	FoodEx2_ TermName	Origin (country)	Purchase (country)	Water (%)	Process	Saponification	Part analysed	Colour	Lutein	Luteoxanthin	Lycopene	Neoxanthin	Phytoene	Ref.
Roman rocket	*Eruca sativa Mill.*	A00LN	Roman rocket	Brazil					leaves	green	5000		nd	1810		109
Roman rocket	*Eruca sativa Mill.*	A00LN	Roman rocket	Slovenia	Slovenia	86.8± 2.9					7440± 780			830±280		97
Wall rocket	*Diplotaxis tenuifolia L.*	A00LP	Wall rocket	Slovenia	Slovenia	85.7± 2.6					5820± 510			350±90		97
Wall rocket	*Diplotaxis tenuifolia L.*	A00LP	Wall rocket	Spain	Spain				edible part	green	8071					24
Wall rocket	*Diplotaxis tenuifolia L.*	A00LP	Wall rocket	Spain	Spain				edible part	green	8061					24

Table S9.4.3 Roman rocket and similar (A00LM) (µg/100g) (continuation)

Food name	Scientific name	FoodEx2_ TermCode	FoodEx2_ TermName	Origin (country)	Purchase (country)	Water (%)	Process	Saponification	Part analysed	Colour	Phytofluene	Violaxanthin	Z(v. cis)-lycopene	Z(v. cis)-β-carotene	Z(v. cis)-β-cryptoxanthin	Zeaxanthin	Ref.
Roman rocket	*Eruca sativa Mill.*	A00LN	Roman rocket	Brazil					leaves	green		2970				nd	109
Roman rocket	*Eruca sativa Mill.*	A00LN	Roman rocket	Slovenia	Slovenia	86.8± 2.9						1560± 420				60±10	97
Wall rocket	*Diplotaxis tenuifolia L.*	A00LP	Wall rocket	Slovenia	Slovenia	85.7± 2.6						710±250				50±10	97

Table S9.5.1 red mustard leaves and similar (A0DKP) (µg/100g)

Food name	Scientific name	FoodEx2_ TermCode	FoodEx2_ TermName	Origin (country)	Purchase (country)	Water (%)	Process	Saponification	Part analysed	Colour	α-carotene	β-carotene	β-cryptoxanthin	ζ-carotene	Antheraxanthin	Ref.
Mustard	*Brassica juncea L. var. red Lion*	A00LQ	red mustard leaves	Lithuania	Lithuania						1040 - 2710	800 - 2310				104

Table S9.5.2 red mustard leaves and similar (A0DKP) (µg/100g) (continuation)

Food name	Scientific name	FoodEx2_ TermCode	FoodEx2_ TermName	Origin (country)	Purchase (country)	Water (%)	Process	Saponification	Part analysed	Colour	Lutein	Luteoxanthin	Lycopene	Neoxanthin	Phytoene	Ref.
Mustard	*Brassica juncea L. var. red Lion*	A00LQ	red mustard leaves	Lithuania	Lithuania									1460 - 8470		104

Table S9.5.3 red mustard leaves and similar (A0DKP) (µg/100g) (continuation)

Food name	Scientific name	FoodEx2_ TermCode	FoodEx2_ TermName	Origin (country)	Purchase (country)	Water (%)	Process	Saponification	Part analysed	Colour	Phytofluene	Violaxanthin	Z(v. cis)-lycopene	Z(v. cis)-β-carotene	Z(v. cis)-β-cryptoxanthin	Zeaxanthin	Ref.
Mustard	Brassica juncea L. 'red Lion'	A00LQ	red mustard leaves	Lithuania	Lithuania							7160 - 18310					104

Table S9.6.1 Baby leaves crops (including brassica species) and similar (A00MA) (µg/100g)

Food name	Scientific name	FoodEx2_ TermCode	FoodEx2_ TermName	Origin (country)	Purchase (country)	Water (%)	Process	Saponification	Part analysed	Colour	α-carotene	β-carotene	β-cryptoxanthin	ζ-carotene	Antheraxanthin	Ref.
Radish	*Raphanus sativa var. Mula pata*	A00MB	Radish leaves (including radish tops)	Bangladesh					leaves			1871± 875				106

Table S9.7.1 Other lettuces and salad plants (A00NF) (µg/100g)

Food name	Scientific name	FoodEx2_ TermCode	FoodEx2_ TermName	Origin (country)	Purchase (country)	Water (%)	Process	Saponification	Part analysed	Colour	α-carotene	β-carotene	β-cryptoxanthin	ζ-carotene	Antheraxanthin	Ref.
Carrot greens	*Daucus carota L.*	A00NF#F27.A 00QH	Other lettuces and salad plants, SOURCE-COMMODITIES = Carrots	India					leaves without stems		21530	12090				98
Ceylon spinach	*Talinum cuniefolium Willd.*	A00NF	Other lettuces and salad plants	India					leaves without stems		18770	42440				98
Blackshade black	*Solanum nigrum L.*	A00NL	Nightshade, black	India					leaves without stems	black		50110				98

Table S9.7.2 Other lettuces and salad plants (A00NF) (µg/100g) (continuation)

Food name	Scientific name	FoodEx2_ TermCode	FoodEx2_ TermName	Origin (country)	Purchase (country)	Water (%)	Process	Saponification	Part analysed	Colour	Lutein	Luteoxanthin	Lycopene	Neoxanthin	Phytoene	Ref.
Carrot greens	*Daucus carota L.*	A00NF#F27.A 00QH	Other lettuces and salad plants, SOURCE-COMMODITIES = Carrots	India					leaves without stems		40170			2090		98
Ceylon spinach	*Talinum cuniefolium Willd.*	A00NF	Other lettuces and salad plants	India					leaves without stems		89790			7950		98
Nightshade black	*Solanum nigrum L.*	A00NL	Nightshade, black	India					leaves without stems	black	84860			2790		98

Table S9.7.3 Other lettuces and salad plants (A00NF) (µg/100g) (continuation)

Food name	Scientific name	FoodEx2_TermCode	FoodEx2_TermName	Origin (country)	Purchase (country)	Water (%)	Process	Saponification	Part analysed	Colour	Phytofluene	Violaxanthin	Z(v. cis)-lycopene	Z(v. cis)-β-carotene	Z(v. cis)-β-cryptoxanthin	Zeaxanthin	Ref.
Carrot greens	*Daucus carota L.*	A00NF#F27.A00QH	Other lettuces and salad plants, SOURCE-COMMODITIES = Carrots	India	India				leaves without stems			7000				590	98
Ceylon spinach	*Talinum cuniefolium Willd.*	A00NF	Other lettuces and salad plants	India	India				leaves without stems			10510				1220	98
Nightshade black	*Solanum nigrum L.*	A00NL	Nightshade, black	India	India				leaves without stems	black		22170					98

Table S9.8.1 Spinaches and similar (A00MH) (µg/100g)

Food name	Scientific name	FoodEx2_TermCode	FoodEx2_TermName	Origin (country)	Purchase (country)	Water (%)	Process	Saponification	Part analysed	Colour	α-carotene	β-carotene	β-cryptoxanthin	ζ-carotene	Antheraxanthin	Ref.
Amaranth	Amaranthus cruentus L.	A0DKG	red amaranth leaves	Tanzania		86.8			young and tender leaves		10260	19120				108
Amaranth	Amaranthus cruentus L.	A0DKG	red amaranth leaves	Tanzania		86.8			young and tender leaves		15380	14230				108
Amaranth	Amaranthus cruentus L.	A0DKG	red amaranth leaves	Tanzania		86.8			young and tender leaves		17770	15850				108
Amaranth	Amaranthus cruentus L.	A0DKG	red amaranth leaves	Tanzania		86.8			young and tender leaves		17230	16660				108

Table S9.8.1 Spinaches and similar (A00MH) (µg/100g) (continuation)

Food name	Scientific name	FoodEx2_ TermCode	FoodEx2_ TermName	Origin (country)	Purchase (country)	Water (%)	Process	Saponification	Part analysed	Colour	α-carotene	β-carotene	β-cryptoxanthin	ζ-carotene	Antheraxanthin	Ref.
Amaranth	Amaranthus cruentus L.	A0DKG	red amaranth leaves	Tanzania		86.8			young and tender leaves		trace	300				108
Amaranth	Amaranthus cruentus L.	A0DKG	red amaranth leaves	Tanzania		86.8			young and tender leaves		170	790				108
Amaranth	Amaranthus cruentus L.	A0DKG	red amaranth leaves	Tanzania		86.8			young and tender leaves		trace	160				108
Amaranth	Amaranthus cruentus L.	A0DKG	red amaranth leaves	Tanzania		86.8			young and tender leaves		trace	tr.				108
Amaranth	Amaranthus cruentus L.	A0DKG	red amaranth leaves	Tanzania		86.8			young and tender leaves		trace	360				108
Amaranth	Amaranthus cruentus L.	A0DKG	red amaranth leaves	Tanzania		86.8			young and tender leaves		trace	tr.				108
Amaranth	Amaranthus gangeticus L.	A00ML	Amaranth leaves	India					without stems			18670				98
Amaranth	Amaranthus gangeticus L.	A00ML	Amaranth leaves	Bangladesh					stem			306±334				106
Amaranth	Amaranthus gangeticusL var. Lal shak	A0DKG	red amaranth leaves	Bangladesh						red		1709±280				106
Amaranth	Amaranthus spp.	A00ML	Amaranth leaves	Bangladesh								1526±56.7				106
Amaranth	Amaranthus viridis L.	A0DKD	green amaranth leaves	India					without stems		6750	58950				98
Amaranth	Amaranthus tristis L.	A0DKE	Chinese amaranth leaves	India					without stems			16760				98
Cowpea	Vigna unguiculata L.	A0DKA	Black eyed peas leaves	Tanzania		88.6			young and tender leaves		8050	14720				108

Table S9.8.1 Spinaches and similar (A00MH) (µg/100g) (continuation)

Food name	Scientific name	FoodEx2_ TermCode	FoodEx2_ TermName	Origin (country)	Purchase (country)	Water (%)	Process	Saponification	Part analysed	Colour	α-carotene	β-carotene	β-cryptoxanthin	ζ-carotene	Antheraxanthin	Ref.
Cowpea	*Vigna unguiculata L.*	A0DKA	Black eyed peas leaves	Tanzania		88.6			young and tender leaves		21400	19860				108
Cowpea	*Vigna unguiculata L.*	A0DKA	Black eyed peas leaves	Tanzania		88.6			young and tender leaves		21040	21060				108
Cowpea	*Vigna unguiculata L.*	A0DKA	Black eyed peas leaves	Tanzania		88.6			young and tender leaves		18810	29400				108
Cowpea	*Vigna unguiculata L.*	A0DKA	Black eyed peas leaves	Tanzania		88.6			young and tender leaves		18300	22890				108
Cowpea	*Vigna unguiculata L.*	A0DKA	Black eyed peas leaves	Tanzania		88.6			young and tender leaves		680	820				108
Cowpea	*Vigna unguiculata L.*	A0DKA	Black eyed peas leaves	Tanzania		88.6			young and tender leaves		1240	4960				108
Cowpea	*Vigna unguiculata L.*	A0DKA	Black eyed peas leaves	Tanzania		88.6			young and tender leaves		300	480				108
Cowpea	*Vigna unguiculata L.*	A0DKA	Black eyed peas leaves	Tanzania		88.6			young and tender leaves		670	670				108
Cowpea	*Vigna unguiculata L.*	A0DKA	Black eyed peas leaves	Tanzania		88.6			young and tender leaves		340	800				108
Cowpea	*Vigna unguiculata L.*	A0DKA	Black eyed peas leaves	Tanzania		88.6			young and tender leaves		1110	2210				108
Mustard	*Brassica juncea L. var. red Lion*	A0DKP	red mustard leaves and similar-	Lithuania	Lithuania			no	leaves		23	48				110

Table S9.8.1 Spinaches and similar (A00MH) (µg/100g) (continuation)

Food name	Scientific name	FoodEx2_ TermCode	FoodEx2_ TermName	Origin (country)	Purchase (country)	Water (%)	Process	Saponification	Part analysed	Colour	α-carotene	β-carotene	β-cryptoxanthin	ζ-carotene	Antheraxanthin	Ref.
Mustard	*Brassica juncea L. var. red Lion*	A0DKP	red mustard leaves and similar-	Lithuania	Lithuania			no	leaves		12	23				110
Mustard	*Brassica juncea L. var. red Lion*	A0DKP	red mustard leaves and similar-	Lithuania	Lithuania			no	leaves		11	21				110
Mustard	*Brassica juncea L. var. red Lion*	A0DKP	red mustard leaves and similar-	Lithuania	Lithuania			no	leaves		14	25				110
Mustard	*Brassica juncea L. var. red Lion*	A0DKP	red mustard leaves and similar-	Lithuania	Lithuania			no	leaves		21	42				110
Spinach	*Spinacea oleracea L.*	A00MJ	Spinaches	Spain	Spain				edible part	green		4626				24
Spinach	*Spinacea oleracea L.*	A00MJ	Spinaches	Spain	Spain	92			leaves+ stalk	green		3254± 330				24
Spinach	*Spinacea oleracea L.*	A00MJ	Spinaches	Spain	Spain	92			leaves+ stalk	green		4626± 346				24
Spinach	*Spinacea oleracea, L.*	A00MJ	Spinaches	Spain	Spain				edible part	green		3254				24
Spinach	*Spinacia oleracea L.*	A00MJ	Spinaches	Brazil					leaves	green	nd	4423	nd			109
Spinach	*Spinacia oleracea L.*	A00MJ	Spinaches	Italy	Italy						nd	3100–4810	nd			25
Spinach	*Spinacia oleracea L.*	A00MJ	Spinaches	Germany	Germany	90.8			edible part		90	3250				55
Spinach	*Spinacia oleracea L.*	A00MJ	Spinaches	Spain	Spain				edible part			4626				28
Spinach	*Spinacia oleracea L.*	A00MJ	Spinaches	Spain	Spain				edible part			3254				28
Spinach	*Spinacia oleracea L.*	A00MJ	Spinaches	Italy	Italy				all sample	L*30.5± 1.1a*8± 0.6b*10 ±0.8		152100± 5000				111
Spinach	*Spinacia oleracea L.*	A00MJ	Spinaches	Italy	Italy				all sample	L*33.7± 1.1a*7. 7±1.1b* 9.3±1		148100±6 500				111

Table S9.8.1 Spinaches and similar (A00MH) (µg/100g) (continuation)

Food name	Scientific name	FoodEx2_ TermCode	FoodEx2_ TermName	Origin (country)	Purchase (country)	Water (%)	Process	Saponification	Part analysed	Colour	α-carotene	β-carotene	β-cryptoxanthin	ζ-carotene	Antheraxanthin	Ref.
Spinach	*Spinacia oleracea L.*	A00MJ	Spinaches	Italy	Italy				all sample	L*33.1± 0.8a*6. 9±0.9b* 9.2±0.8		184600±9 300				111
Spinach	*Spinacia olerácea L.*	A00MJ#F28.A 0BA1	Spinaches, PROCESS = Cooking and similar thermal preparation processes	USA			Cooked	no			0		0			28
Spinach	*Spinacia olerácea L.*	A00MJ	Spinaches	USA			Raw	no			0		0			28
Sweet potato	*Ipomea batatas L.*	A00NX	Sweet potato leaves	Tanzania		81.6			young and tender leaves		1660	7690				108
Sweet potato	*Ipomea batatas L.*	A00NX	Sweet potato leaves	Tanzania		81.6			young and tender leaves		1770	5380				108
Sweet potato	*Ipomea batatas L.*	A00NX	Sweet potato leaves	Tanzania		81.6			young and tender leaves		1950	4730				108
Sweet potato	*Ipomea batatas L.*	A00NX	Sweet potato leaves	Tanzania		81.6			young and tender leaves		2760	3780				108
Sweet potato	*Ipomea batatas L.*	A00NX	Sweet potato leaves	Tanzania		81.6			young and tender leaves		670	480				108
Sweet potato	*Ipomea batatas L.*	A00NX	Sweet potato leaves	Tanzania		81.6			young and tender leaves		670	1290				108
Sweet potato	*Ipomea batatas L.*	A00NX	Sweet potato leaves	Tanzania		81.6			young and tender leaves		110	300				108
Sweet potato	*Ipomea batatas L.*	A00NX	Sweet potato leaves	Tanzania		81.6			young and tender leaves		170	400				108

Table S9.8.1 Spinaches and similar (A00MH) (µg/100g) (continuation)

Food name	Scientific name	FoodEx2_ TermCode	FoodEx2_ TermName	Origin (country)	Purchase (country)	Water (%)	Process	Saponification	Part analysed	Colour	α-carotene	β-carotene	β-cryptoxanthin	ζ-carotene	Antheraxanthin	Ref.
Sweet potato	*Ipomea batatas L.*	A00NX	Sweet potato leaves	Tanzania		81.6			young and tender leaves		170	510				108
Sweet potato	*Ipomea batatas L.*	A00NX	Sweet potato leaves	Tanzania		81.6			young and tender leaves		170	780				108
Sweet potato	*Ipomea batatas L.*	A00NX	Sweet potato leaves	Tanzania		81.6			young and tender leaves		990	8990				108
Tayer	*Xanthosoma sagittifolium (L.)*	A00NT	Tannia leaves	Netherlands	Belgium							7919				20

Table S9.8.2 Spinaches and similar (A00MH) (µg/100g) (continuation)

Food name	Scientific name	FoodEx2_ TermCode	FoodEx2_ TermName	Origin (country)	Purchase (country)	Water (%)	Process	Saponification	Part analysed	Colour	Capsanthin	Capsorubin	Cucurbitaxanthin	E(v. trans)-α-carotene	E(v. trans)-β-carotene	Ref.
Spinach	*Spinacia olerácea L.*	A00MJ#F28.A0BA1	Spinaches, PROCESS = Cooking and similar thermal preparation processes	USA			cooked	no							8852	28
Spinach	*Spinacia olerácea L.*	A00MJ	Spinaches	USA			raw	no							4388	28

Table S9.8.3 Spinaches and similar (A00MH) (µg/100g) (continuation)

Food name	Scientific name	FoodEx2_ TermCode	FoodEx2_ TermName	Origin (country)	Purchase (country)	Water (%)	Process	Saponification	Part analysed	Colour	E(v. trans)-β-cryptoxanthin	E(v. trans)-lutein	E(v. trans)-lycopene	E(v. trans)-zeaxanthin	Lactucaxanthin	Ref.
Spinach	Spinacia oleracea	A00MJ#F28.A0BA1	Spinaches, PROCESS = Cooking and similar thermal preparation processes	USA			cooked	no				12640		0		28
Spinach	Spinacia oleracea	A00MJ	Spinaches	USA			raw	no				6603		0		28
Spinach	Spinacia oleracea, var. Matador	A00MJ	Spinaches	Latvia	Latvia				leaves	green		4000				112

Table S9.8.4 Spinaches and similar (A00MH) (µg/100g) (continuation)

Food name	Scientific name	FoodEx2_ TermCode	FoodEx2_ TermName	Origin (country)	Purchase (country)	Water (%)	Process	Saponification	Part analysed	Colour	Lutein	Luteoxanthin	Lycopene	Neoxanthin	Phytoene	Ref.
Amaranth	*Amaranthus gangeticus L.*	A00ML	Amaranth leaves	India					without stems		32020			1460		98
Amaranth	*Amaranthus viridis L.*	A0DKD	green amaranth leaves	India					without stems		90430			12630		98
Amaranth	*Amaranthus tristis L.*	A0DKE	Chinese amaranth leaves	India					without stems		30300			1150		98
Mustard	*Brassica juncea L. var. red Lion*	A0DKP	red mustard leaves and similar-	Lithuania	Lithuania			no	leaves		5364			188		110
Mustard	*Brassica juncea L. var. red Lion*	A0DKP	red mustard leaves and similar-	Lithuania	Lithuania			no	leaves		5971			315		110
Mustard	*Brassica juncea L. var. red Lion*	A0DKP	red mustard leaves and similar-	Lithuania	Lithuania			no	leaves		6709			321		110

Table S9.8.4 Spinaches and similar (A00MH) (µg/100g) (continuation)

Food name	Scientific name	FoodEx2_ TermCode	FoodEx2_ TermName	Origin (country)	Purchase (country)	Water (%)	Process	Saponification	Part analysed	Colour	Lutein	Luteoxanthin	Lycopene	Neoxanthin	Phytoene	Ref.
Mustard	*Brassica juncea L. var. red Lion*	A0DKP	red mustard leaves and similar-	Lithuania	Lithuania			no	leaves		7973			387		110
Mustard	*Brassica juncea L. var. red Lion*	A0DKP	red mustard leaves and similar-	Lithuania	Lithuania			no	leaves		5869			233		110
Spinach	*Spinacea olerácea L.*	A00MJ	Spinaches	Spain	Spain				edible part	green	6422					24
Spinach	*Spinacea oleracea, L.*	A00MJ	Spinaches	Spain	Spain				edible part	green	4229					24
Spinach	*Spinacea oleracea, L.*	A00MJ	Spinaches	Spain	Spain	92			leaves+ stalk	green	4229± 1310					24
Spinach	*Spinacea oleracea, L.*	A00MJ	Spinaches	Spain	Spain	92			leaves+ stalk	green	6422± 1190					24
Spinach	*Spinacia oleracea L.*	A00MJ	Spinaches	Brazil					leaves	green	5793		nd	1743		109
Spinach	*Spinacia oleracea L.*	A00MJ	Spinaches	Italy	Italy						5930–7900		nd			25
Spinach	*Spinacia oleracea L.*	A00MJ	Spinaches	Germany	Germany	90.8			edible part		9540			160		53
Spinach	*Spinacia oleracea L.*	A00MJ	Spinaches	Germany	Germany				edible part		8700± 100					113
Spinach	*Spinacia oleracea L.*	A00MJ	Spinaches	Spain	Spain				edible part		6422					24
Spinach	*Spinacia oleracea L.*	A00MJ	Spinaches	Spain	Spain				edible part		4229					24
Spinach	*Spinacia oleracea L.*	A00MJ	Spinaches	Italy	Italy				all sample	L*30.5±1.1a*8±0.6b*10±0.8	218700± 15400					111
Spinach	*Spinacia oleracea L.*	A00MJ	Spinaches	Italy	Italy				all sample	L*33.7±1.1a*7.7±1.1b*9.3±1	167000± 10400					111
Spinach	*Spinacia oleracea L.*	A00MJ	Spinaches	Italy	Italy				all sample	L*33.1±0.8a*6.9±0.9b*9.2±0.8	194200± 4200					111
Spinach	*Spinacia oleracea L.*	A00MJ	Spinaches	Slovenia	Slovenia	90.7			leaves	green	4840-13900					114

Table S9.8.5 Spinaches and similar (A00MH) (µg/100g) (continuation)

Food name	Scientific name	FoodEx2_ TermCode	FoodEx2_ TermName	Origin (country)	Purchase (country)	Water (%)	Process	Saponification	Part analysed	Colour	Phytofluene	Violaxanthin	Z(v. cis)- lycopene	Z(v. cis)-β- carotene	Z(v. cis)-β- cryptoxanthin	Zeaxanthin	Ref.
Amaranth	*Amaranthus gangeticus L.*	A00ML	Amaranth leaves	India					without stems			26500				340	98
Amaranth	*Amaranthus tristis L.*	A0DKE	Chinese amaranth leaves	India					without stems			19150				230	98
Amaranth	*Amaranthus viridis L.*	A0DKD	green amaranth leaves	India					without stems			84060				1040	98
Mustard	*Brassica juncea L. var. red Lion*	A0DKP	red mustard leaves and similar-	Lithuania	Lithuania			no	leaves			463				1898	113
Mustard	*Brassica juncea L. var. red Lion*	A0DKP	red mustard leaves and similar-	Lithuania	Lithuania			no	leaves			1318				2565	110
Mustard	*Brassica juncea L. var. red Lion*	A0DKP	red mustard leaves and similar-	Lithuania	Lithuania			no	leaves			1595				3156	110
Mustard	*Brassica juncea L. var. red Lion*	A0DKP	red mustard leaves and similar-	Lithuania	Lithuania			no	leaves			2100				2831	110
Mustard	*Brassica juncea L. var. red Lion*	A0DKP	red mustard leaves and similar-	Lithuania	Lithuania			no	leaves			675				2653	110
Spinach	*Spinacea oleracea L.*	A00MJ	Spinaches	Spain	Spain				edible part	green						564	24
Spinach	*Spinacea oleracea L.*	A00MJ	Spinaches	Spain	Spain				edible part	green						377	24
Spinach	*Spinacea oleracea L.*	A00MJ	Spinaches	Spain	Spain	92			leaves+ stalk	green						377±103	24
Spinach	*Spinacea oleracea L.*	A00MJ	Spinaches	Spain	Spain	92			leaves+ stalk	green						564±75	24
Spinach	*Spinacia oleracea L.*	A00MJ	Spinaches	Brazil					leaves	green		2725				nd	109

Table S9.8.5 Spinaches and similar (A00MH) (µg/100g) (continuation)

Food name	Scientific name	FoodEx2_TermCode	FoodEx2_TermName	Origin (country)	Purchase (country)	Water (%)	Process	Saponification	Part analysed	Colour	Phytofluene	Violaxanthin	Z(v. cis)-lycopene	Z(v. cis)-β-carotene	Z(v. cis)-β-cryptoxanthin	Zeaxanthin	Ref.
Spinach	*Spinacia oleracea L.*	A00MJ	Spinaches	Germany	Germany	90.8			edible part			3040				350	53
Spinach	*Spinacia oleracea L.*	A00MJ	Spinaches	Spain	Spain				edible part							564	24
Spinach	*Spinacia oleracea L.*	A00MJ	Spinaches	Spain	Spain				edible part							377	24
Spinach	*Spinacia oleracea L.*	A00MJ#F28.A0BA1	Spinaches, PROCESS = Cooking and similar thermal preparation processes	USA			cooked	no						1280			28
Spinach	*Spinacia oleracea L.*	A00MJ	Spinaches	USA			raw	no						708			28

Table S9.9.1 Purslanes and similar (A00MN) (µg/100g)

Food name	Scientific name	FoodEx2_TermCode	FoodEx2_TermName	Origin (country)	Purchase (country)	Water (%)	Process	Saponification	Part analysed	Colour	α-carotene	β-carotene	β-cryptoxanthin	ζ-carotene	Antheraxanthin	Ref.
Purslane	*Portulaca oleracea L.*	A00MP	Purslanes	India					without stems			27050				98
Purslane	*Portulaca oleraceae L.*	A00MP	Purslanes	Portugal	Portugal				leaves	green	9	3500	nd			32

Table S9.9.2 Purslanes and similar (A00MN) (µg/100g) (continuation)

Food name	Scientific name	FoodEx2_ TermCode	FoodEx2_ TermName	Origin (country)	Purchase (country)	Water (%)	Process	Saponification	Part analysed	Colour	Lutein	Luteoxanthin	Lycopene	Neoxanthin	Phytoene	Ref.
Purslane	*Portulaca oleracea L.*	A00MP	Purslanes	India					without stems		50840			730		98
Purslane	*Portulaca oleraceae L.*	A00MP	Purslanes	Portugal	Portugal				leaves	green	5400		nd			32

Table S9.9.3 Purslanes and similar (A00MN) (µg/100g) (continuation)

Food name	Scientific name	FoodEx2_ TermCode	FoodEx2_ TermName	Origin (country)	Purchase (country)	Water (%)	Process	Saponification	Part analysed	Colour	Phytofluene	Violaxanthin	Z(v. cis)-lycopene	Z(v. cis)-β-carotene	Z(v. cis)-β-cryptoxanthin	Zeaxanthin	Ref.
Purslane	*Portulaca oleracea L.*	A00MP	Purslanes	India					without stems			11470				940	98

Table S9.10.1 Chards and similar (A0DJT) (µg/100g)

Food name	Scientific name	FoodEx2_ TermCode	FoodEx2_ TermName	Origin (country)	Purchase (country)	Water (%)	Process	Saponification	Part analysed	Colour	α-carotene	β-carotene	β-cryptoxanthin	ζ-carotene	Antheraxanthin	Ref.
Beetroot	*Beta vulgaris var. vulgaris L.*	A0DJS	Beetroot leaves	India					without stems		1540	12500				98
Beetroot	*Beta vulgaris var. vulgaris L.*	A0DJS	Beetroot leaves	Portugal	Portugal				leaves	green	nd	2500	nd			32
Beetroot	*Beta vulgaris L. ssp. vulgaris convar. cicla (L.) Alef.*	A0DJS	Beetroot leaves	Portugal	Portugal				edible part	purple	nd	2900	nd			32
Chard	*Beta vulgaris L.*	A00MX	Chards	Spain	Spain				stems + leaves	green		1360				24

Table S9.10.1 Chards and similar (A0DJT) (µg/100g) (continuation)

Food name	Scientific name	FoodEx2_ TermCode	FoodEx2_ TermName	Origin (country)	Purchase (country)	Water (%)	Process	Saponification	Part analysed	Colour	α-carotene	β-carotene	β-cryptoxanthin	ζ-carotene	Antheraxanthin	Ref.
Chard	*Beta vulgaris L.*	A00MX	Chards	Spain	Spain				stems and leaves	green		1095				24
Chard	*Beta vulgaris L.*	A00MX	Chards	Spain	Spain				Stems + leaves	green		1360				24
Chard	*Beta vulgaris L.*	A00MX	Chards	Spain	Spain				Stems + leaves	green		1095				24
Chard	*Beta vulgaris L. var. cicla*	A00MX	Chards	Brazil					leaves + stalk	green and white	35	2700	nd			109

Table S9.10.2 Chards and similar (A0DJT) (µg/100g) (continuation)

Food name	Scientific name	FoodEx2_ TermCode	FoodEx2_ TermName	Origin (country)	Purchase (country)	Water (%)	Process	Saponification	Part analysed	Colour	Capsanthin	Capsorubin	Cucurbitaxanthin	E(v. trans)-α-carotene	E(v. trans)-β-carotene	Ref.
Beetroot	*Beta vulgaris L. ssp. vulgaris convar. cicla (L.) Alef.*	A0DJS	Beetroot leaves	Netherlands	Netherlands				leaves	green					3110± 330	57
Beetroot	*Beta vulgaris L. ssp. vulgaris convar. cicla (L.) Alef.*	A0DJS	Beetroot leaves	Netherlands	Netherlands				leaves	green					2410± 230	57
Beetroot	*Beta vulgaris L. ssp. vulgaris convar. cicla (L.) Alef.*	A0DJS	Beetroot leaves	Netherlands	Netherlands				leaves	green					2790± 590	57

Table S9.10.3 Chards and similar (A0DJT) (µg/100g) (continuation)

Food name	Scientific name	FoodEx2_ TermCode	FoodEx2_ TermName	Origin (country)	Purchase (country)	Water (%)	Process	Saponification	Part analysed	Colour	E(v. trans)-β-cryptoxanthin	E(v. trans)-lutein	E(v. trans)-lycopene	E(v. trans)-zeaxanthin	Lactucaxanthin	Ref.
Beetroot	*Beta vulgaris L. ssp. vulgaris convar. cicla (L.) Alef.*	A0DJS	Beetroot leaves	Netherlands	Netherlands				leaves	green		5210±650				57
Beetroot	*Beta vulgaris L. ssp. vulgaris convar. cicla (L.) Alef.*	A0DJS	Beetroot leaves	Netherlands	Netherlands				leaves	green		3810±330				57
Beetroot	*Beta vulgaris L. ssp. vulgaris convar. cicla (L.) Alef.*	A0DJS	Beetroot leaves	Netherlands	Netherlands				leaves	green		4460±770		250±54		57

Table S9.10.4 Chards and similar (A0DJT) (µg/100g) (continuation)

Food name	Scientific name	FoodEx2_ TermCode	FoodEx2_ TermName	Origin (country)	Purchase (country)	Water (%)	Process	Saponification	Part analysed	Colour	Lutein	Luteoxanthin	Lycopene	Neoxanthin	Phytoene	Ref.
Beetroot	Beta vulgaris L. var. vulgaris	A0DJS	Beetroot leaves	India					without stems		26860			6390		98
Beetroot	Beta vulgaris L. ssp. vulgaris convar. cicla (L.) Alef.	A0DJS	Beetroot leaves	Portugal	Portugal				edible part	purple	3600		nd			32
Beetroot	Beta vulgaris L. var. vulgaris	A0DJS	Beetroot leaves	Portugal	Portugal				leaves	green	4400		nd			32
Chard	Beta vulgaris L.	A00MX	Chards	Spain	Spain				stems +leaves	green	1960					24

Table S9.10.4 Chards and similar (A0DJT) (µg/100g) (continuation)

Food name	Scientific name	FoodEx2_ TermCode	FoodEx2_ TermName	Origin (country)	Purchase (country)	Water (%)	Process	Saponification	Part analysed	Colour	Lutein	Luteoxanthin	Lycopene	Neoxanthin	Phytoene	Ref.
Chard	*Beta vulgaris L.*	A00MX	Chards	Spain	Spain				stems +leaves	green	1503					24
Chard	*Beta vulgaris L.*	A00MX	Chards	Spain	Spain				all sample	green	1960					24
Chard	*Beta vulgaris L.*	A00MX	Chards	Spain	Spain				edible part	green	1503					24
Chard	*Beta vulgaris L. var. cicla*	A00MX	Chards	Brazil					leaves + stalk	green + white	2700		nd	nd		109

Table S9.10.5 Chards and similar (A0DJT) (µg/100g) (continuation)

Food name	Scientific name	FoodEx2_ TermCode	FoodEx2_ TermName	Origin (country)	Purchase (country)	Water (%)	Process	Saponification	Part analysed	Colour	Phytofluene	Violaxanthin	Z(v. cis)- lycopene	Z(v. cis)-β- carotene	Z(v. cis)-β- cryptoxanthin	Zeaxanthin	Ref.
Beetroot	Beta vulgaris L. var. vulgaris	A0DJS	Beetroot leaves	India					without stems			3970				140	98
Chard	*Beta vulgaris L. var. cicla*	A00MX	Chards	Brazil					leaves + stalk	green +white		92				nd	109

Table S9.11.1 Other spinaches and similar leaves (A00MY) (µg/100g)

Food name	Scientific name	FoodEx2_ TermCode	FoodEx2_ TermName	Origin (country)	Purchase (country)	Water (%)	Process	Saponification	Part analysed	Colour	α-carotene	β-carotene	β-cryptoxanthin	ζ-carotene	Antheraxanthin	Ref.
Goosefoot	*Chenopodium album L.*	A00MZ	Goosefoot	India					without stems			114610				98

Table S9.11.2 Other spinaches and similar leaves (A00MY) (µg/100g) (continuation)

Food name	Scientific name	FoodEx2_ TermCode	FoodEx2_ TermName	Origin (country)	Purchase (country)	Water (%)	Process	Saponification	Part analysed	Colour	Lutein	Luteoxanthin	Lycopene	Neoxanthin	Phytoene	Ref.
Goosefoot	*Chenopodium album L.*	A00MZ	Goosefoot	India					without stems		187590					98

Table S9.11.3 Other spinaches and similar leaves (A00MY) (µg/100g) (continuation)

Food name	Scientific name	FoodEx2_ TermCode	FoodEx2_ TermName	Origin (country)	Purchase (country)	Water (%)	Process	Saponification	Part analysed	Colour	Phytofluene	Violaxanthin	Z(v. cis)-lycopene	Z(v. cis)-β-carotene	Z(v. cis)-β-cryptoxanthin	Zeaxanthin	Ref.
Goosefoot	*Chenopodium album L.*	A00MZ	Goosefoot	India					without stems			142590				5000	98

Table S9.12.1 Other leavesy vegeTable Ss (A04RC) (µg/100g)

Food name	Scientific name	FoodEx2_ TermCode	FoodEx2_ TermName	Origin (country)	Purchase (country)	Water (%)	Process	Saponification	Part analysed	Colour	α-carotene	β-carotene	β-cryptoxanthin	ζ-carotene	Antheraxanthin	Ref.
Dandelion	Taraxacum officinale Waggner	A0DJK	Dandelion leaves (forced)	Slovenia	Slovenia	87.4± 1.6			edible part			6340±940			470±150	97
Desert horse purslane	Trianthema portulacastrum L.	A04RC	Other leavesy vegetables	India					without stems			37760				98
Duckweed	Lemnaceae	A04RC	Other leavesy vegetables	Germany	Germany				Biomass	green		28000				115

Table S9.12.1 Other leavesy vegetables (A04RC) (µg/100g) (continuation)

Food name	Scientific name	FoodEx2_ TermCode	FoodEx2_ TermName	Origin (country)	Purchase (country)	Water (%)	Process	Saponification	Part analysed	Colour	α-carotene	β-carotene	β-cryptoxanthin	ζ-carotene	Antheraxanthin	Ref.
Herb Chanca piedra	Phyllanthus niruri L.	A04RC	Other leavesy vegetables	India					without stems			60880				98
Herb indian pennywort	Hydrocotyle asiatica L.	A04RC	Other leavesy vegetables	India					without stems			9020				98
Herb Jio	Commelina benghalensis L.	A04RC	Other leavesy vegetables	India					without stems		37170	92820				98
Hog weed	Boerhavia diffusa L.	A04RC	Other leavesy vegetables	India					without stems			27670				98
Indian spinach	Basella alba L.	A00MM	Malabar nightshades	India					without stems		18230	43820				98
Joy weed	Alternanthera sessilis (L.) Dc.	A04RC	Other leavesy vegetables	India					without stems			27070				98
Kenaf	Hibiscus cannabinus L.	A04RC	Other leavesy vegetables	India					without stems			26020				98
Khaki weed	Alternanthera pungens Kunth	A04RC	Other leavesy vegetables	India					without stems			34660				98
Moringa oleifera	Moringa oleifera	A04RC#F01.A0E7V	Other leavesy vegetables, SOURCE = Moringa (as plant)	Greece	Greece				leaves			2020±120				116
Peach	Arachis hypogaea	A04RC#F02.A0EKV$F01.A061C	Other leavesy vegetables, PART-NATURE = leaves used as leavesy vegetables (as part-nature), SOURCE = Peach (as plant)	Tanzania		75.4			young and tender leaves		trace	tr.				108
Peach	Arachis hypogaea	A04RC#F02.A0EKV$F01.A061C	Other leavesy vegetables, PART-NATURE = leaves used as leavesy vegetables (as part-nature), SOURCE = Peach (as plant)	Tanzania		75.4			young and tender leaves		trace	tr.				108
Peanut	Arachis hypogaea	A04RC#F02.A0EKV$F01.A05RT	Other leavesy vegetables, PART-NATURE = leaves used as leavesy vegetables (as part-nature), SOURCE = Peanuts (as plant)	Tanzania		75.4			young and tender leaves		1630	4160				108

Table S9.12.1 Other leavesy vegetables (A04RC) (µg/100g) (continuation)

Food name	Scientific name	FoodEx2_ TermCode	FoodEx2_ TermName	Origin (country)	Purchase (country)	Water (%)	Process	Saponification	Part analysed	Colour	α-carotene	β-carotene	β-cryptoxanthin	ζ-carotene	Antheraxanthin	Ref.
Peanut	Arachis hypogaea	A04RC#F02.A0EKV$F01.A05RT	Other leavesy vegetables, PART-NATURE = leaves used as leavesy vegetables (as part-nature), SOURCE = Peanuts (as plant)	Tanzania					young and tender leaves		2170	5540				108
Peanut	Arachis hypogaea	A04RC#F02.A0EKV$F01.A05RT	Other leavesy vegetables, PART-NATURE = leaves used as leavesy vegetables (as part-nature), SOURCE = Peanuts (as plant)	Tanzania		75.4			young and tender leaves		9610	6400				108
Peanut	Arachis hypogaea	A04RC#F02.A0EKV$F01.A05RT	Other leavesy vegetables, PART-NATURE = leaves used as leavesy vegetables (as part-nature), SOURCE = Peanuts (as plant)	Tanzania		75.4			young and tender leaves		11010	6370				108
Peanut	Arachis hypogaea	A04RC#F02.A0EKV$F01.A05RT	Other leavesy vegetables, PART-NATURE = leaves used as leavesy vegetables (as part-nature), SOURCE = Peanuts (as plant)	Tanzania		75.4			young and tender leaves		10740	6400				108
Peanut	Arachis hypogaea	A04RC#F02.A0EKV$F01.A05RT	Other leavesy vegetables, PART-NATURE = leaves used as leavesy vegetables (as part-nature), SOURCE = Peanuts (as plant)	Tanzania		75.4			young and tender leaves		130	tr.				108
Peanut	Arachis hypogaea	A04RC#F02.A0EKV$F01.A05RT	Other leavesy vegetables, PART-NATURE = leaves used as leavesy vegetables (as part-nature), SOURCE = Peanuts (as plant)	Tanzania		75.4			young and tender leaves		280	500				108
Peanut	Arachis hypogaea	A04RC#F02.A0EKV$F01.A05RT	Other leavesy vegetables, PART-NATURE = leaves used as leavesy vegetables (as part-nature), SOURCE = Peanuts (as plant)	Tanzania		75.4			young and tender leaves		280	330				108

Table S9.12.1 Other leavesy vegetables (A04RC) (µg/100g) (continuation)

Food name	Scientific name	FoodEx2_ TermCode	FoodEx2_ TermName	Origin (country)	Purchase (country)	Water (%)	Process	Saponification	Part analysed	Colour	α-carotene	β-carotene	β-cryptoxanthin	ζ-carotene	Antheraxanthin	Ref.
Peanut	*Arachis hypogaea*	A04RC#F02.A0EKV$F01.A05RT	Other leavesy vegetables, PART-NATURE = leaves used as leavesy vegetables (as part-nature), SOURCE = Peanuts (as plant)	Tanzania		75.4			young and tender leaves		170	370				108
Pumpkin	*Cucurbita moschata*	A04RC#F02.A0EKV$F01.A0E3S	Other leavesy vegetables, PART-NATURE = leaves used as leavesy vegetables (as part-nature), SOURCE = Butternut squash (as plant)	Tanzania		89.6			young and tender leaves		540	2160				108
Pumpkin	*Cucurbita moschata*	A04RC#F02.A0EKV$F01.A0E3S	Other leavesy vegetables, PART-NATURE = leaves used as leavesy vegetables (as part-nature), SOURCE = Butternut squash (as plant)	Tanzania		89.6			young and tender leaves		1560	7280				108
Pumpkin	*Cucurbita moschata*	A04RC#F02.A0EKV$F01.A0E3S	Other leavesy vegetables, PART-NATURE = leaves used as leavesy vegetables (as part-nature), SOURCE = Butternut squash (as plant)	Tanzania		89.6			young and tender leaves		430	1660				108
Pumpkin	*Cucurbita moschata*	A04RC#F02.A0EKV$F01.A0E3S	Other leavesy vegetables, PART-NATURE = leaves used as leavesy vegetables (as part-nature), SOURCE = Butternut squash (as plant)	Tanzania		89.6			young and tender leaves		530	1640				108
Pumpkin	*Cucurbita moschata*	A04RC#F02.A0EKV$F01.A0E3S	Other leavesy vegetables, PART-NATURE = leaves used as leavesy vegetables (as part-nature), SOURCE = Butternut squash (as plant)	Tanzania		89.6			young and tender leaves		540	3020				108
Pumpkin	*Cucurbita moschata*	A04RC#F02.A0EKV$F01.A0E3S	Other leavesy vegetables, PART-NATURE = leaves used as leavesy vegetables (as part-nature), SOURCE = Butternut squash (as plant)	Tanzania		89.6			young and tender leaves		730	3180				108

Table S9.12.1 Other leavesy vegetables (A04RC) (µg/100g) (continuation)

Food name	Scientific name	FoodEx2_ TermCode	FoodEx2_ TermName	Origin (country)	Purchase (country)	Water (%)	Process	Saponification	Part analysed	Colour	α-carotene	β-carotene	β-cryptoxanthin	ζ-carotene	Antheraxanthin	Ref.
Pumpkin	*Cucurbita moschata*	A04RC#F02.A0EKV$F01.A0E3S	Other leavesy vegetables, PART-NATURE = leaves used as leavesy vegetables (as part-nature), SOURCE = Butternut squash (as plant)	Tanzania		89.6			young and tender leaves		1890	11200				108
Pumpkin	*Cucurbita moschata*	A04RC#F02.A0EKV$F01.A0E3S	Other leavesy vegetables, PART-NATURE = leaves used as leavesy vegetables (as part-nature), SOURCE = Butternut squash (as plant)	Tanzania		89.6			young and tender leaves		10890	17400				108
Pumpkin	*Cucurbita moschata*	A04RC#F02.A0EKV$F01.A0E3S	Other leavesy vegetables, PART-NATURE = leaves used as leavesy vegetables (as part-nature), SOURCE = Butternut squash (as plant)	Tanzania		89.6			young and tender leaves)		17410	20380				108
Pumpkin	*Cucurbita moschata*	A04RC#F02.A0EKV$F01.A0E3S	Other leavesy vegetables, PART-NATURE = leaves used as leavesy vegetables (as part-nature), SOURCE = Butternut squash (as plant)	Tanzania		89.6			young and tender leaves		18180	24860				108
Pumpkin	*Cucurbita moschata*	A04RC#F02.A0EKV$F01.A0E3S	Other leavesy vegetables, PART-NATURE = leaves used as leavesy vegetables (as part-nature), SOURCE = Butternut squash (as plant)	Tanzania		89.6			young and tender leaves		28220	25900				108
Puncture vine	*Tribulus terrestris L.*	A04RC	Other leavesy vegetables	India					without stems		6100	30810				98
Rampion	*Phyteuma orbiculare L.*	A04RC	Other leavesy vegetables	Switzerland	Switzerland	40.9			leaves			3700				117
Sheep sorrel	*Rumex acetosella L.*	A04RC	Other leavesy vegetables	India					without stems			70830				98
Spider wisp	*Gynandropsis pentaphylla L.*	A04RC	Other leavesy vegetables	India					without stems			37040				98

Table S9.12.1 Other leavesy vegetables (A04RC) (µg/100g) (continuation)

Food name	Scientific name	FoodEx2_ TermCode	FoodEx2_ TermName	Origin (country)	Purchase (country)	Water (%)	Process	Saponification	Part analysed	Colour	α-carotene	β-carotene	β-cryptoxanthin	ζ-carotene	Antheraxanthin	Ref.
Watercress	*Nasturtium officinale W.T. Aiton*	A00ND	Watercresses	Brazil					leaves	green	nd	2720	nd			109
Watercress	*Nasturtium officinale W.T. Aiton*	A00ND	Watercresses	Spain	Spain				edible part	green		5919				24
Watercress	*Nasturtium officinale W.T. Aiton*	A00ND	Watercresses	Spain	Spain				edible part	green		5919				24
Winter squash	*Cucurbita maxima Duchesne*	A04RC	Other leavesy vegetables	India					without stems			10270				98

Table S9.12.2 Other leavesy vegetables (A04RC) (µg/100g) (continuation)

Food name	Scientific name	FoodEx2_ TermCode	FoodEx2_ TermName	Origin (country)	Purchase (country)	Water (%)	Process	Saponification	Part analysed	Colour	Lutein	Luteoxanthin	Lycopene	Neoxanthin	Phytoene	Ref.
Dandelion	*Taraxacum officinale Waggner*	A0DJK	Dandelion leaves (forced)	Slovenia	Slovenia	87.4± 1.6			edible part		5250±620			420±140		97
Desert horse purslane	*Trianthema portulacastrum L.*	A04RC	Other leavesy vegetables	India					without stems		41510			2500		98
Duckweed	*Lemnaceae*	A04RC	Other leavesy vegetables	Germany	Germany				biomass	green	70000					115
Herb Chanca piedra	*Phyllanthus niruri L.*	A04RC	Other leavesy vegetables	India					without stems		77550			33600		98
Herb indian pennywort	*Hydrocotyle asiatica L.*	A04RC	Other leavesy vegetables	India					without stems		15930			890		98
Herb Jio	*Commelina benghalensis L.*	A04RC	Other leavesy vegetables	India					without stems		181300					98

Table S9.12.2 Other leavesy vegetables (A04RC) (µg/100g) (continuation)

Food name	Scientific name	FoodEx2_ TermCode	FoodEx2_ TermName	Origin (country)	Purchase (country)	Water (%)	Process	Saponification	Part analysed	Colour	Lutein	Luteoxanthin	Lycopene	Neoxanthin	Phytoene	Ref.
Hog weed	*Boerhavia diffusa L.*	A04RC	Other leavesy vegetables	India					without stems		26830			1410		98
Indian spinach	*Basella alba L.*	A00MM	Malabar nightshades	India					without stems		113820			7740		98
Joy weed	*Alternanthera sessilis (L.) Dc.*	A04RC	Other leavesy vegetables	India					without stems		32470			13890		98
Kenaf	*Hibiscus cannabinus L.*	A04RC	Other leavesy vegetables	India					without stems		33970			5950		98
Khaki weed	*Alternanthera pungens Kunth*	A04RC	Other leavesy vegetables	India					without stems		71860					98
Moringa oleifera	*Moringa oleifera leaves*	A04RC#F01. A0E7V	Other leavesy vegetables, SOURCE = Moringa (as plant)	Greece	Greece				leaves		10030± 470					116
Puncture vine	*Tribulus terrestris L.*	A04RC	Other leavesy vegetables	India					without stems		56390			1340		98
Sheep sorrel	*Rumex acetosella L.*	A04RC	Other leavesy vegetables	India					without stems		144300			7700		98
Spider wisp	*Gynandropsis pentaphylla L.*	A04RC	Other leavesy vegetables	India					without stems		42650			49310		98
Watercress	*Nasturtium officinale R. Br.*	A00ND	Watercresses	Brazil					leaves	green	5610		nd	1770		109
Winter squash	*Cucurbita maxima Duchesne*	A04RC	Other leavesy vegetables	India					without stems		27180					98

Table S9.12.3 Other leavesy vegetables (A04RC) (µg/100g) (continuation)

Food name	Scientific name	FoodEx2_ TermCode	FoodEx2_ TermName	Origin (country)	Purchase (country)	Water (%)	Process	Saponification	Part analysed	Colour	Phytofluene	Violaxanthin	Z(v. cis)-lycopene	Z(v. cis)-β-carotene	Z(v. cis)-β-cryptoxanthin	Zeaxanthin	Ref.
Dandelion	*Taraxacum officinale Waggner*	A0DJK	Dandelion leaves (forced)	Slovenia	Slovenia	87.4± 1.6			edible part			650±180				80±20	97
Desert horse purslane	*Trianthema portulacastrum L.*	A04RC	Other leavesy vegetables	India					without stems			5000				440	98
Duckweed	*Lemnaceae*	A04RC	Other leavesy vegetables	Germany	Germany				biomass	green		46000				4300	115
herb Chanca piedra	*Phyllanthus niruri L.*	A04RC	Other leavesy vegetables	India					without stems			3670				1630	98
herb indian pennywort	*Hydrocotyle asiatica L.*	A04RC	Other leavesy vegetables	India					without stems			650					98
herb Jio	*Commelina benghalensis L.*	A04RC	Other leavesy vegetables	India					without stems			102930				2060	98
Hog weed	*Boerhavia diffusa L.*	A04RC	Other leavesy vegetables	India					without stems			3930				190	98
Indian spinach	*Basella alba L.*	A00MM	Malabar nightshades	India					without stems			6270				1760	98
Joy weed	*Alternanthera sessilis (L.) Dc.*	A04RC	Other leavesy vegetables	India					without stems			23930				260	98
Kenaf	*Hibiscus cannabinus L.*	A04RC	Other leavesy vegetables	India					without stems							140	98
Khaki weed	*Alternanthera pungens Kunth*	A04RC	Other leavesy vegetables	India					without stems			42030				670	98
Moringa oleifera	*Moringa oleifera leaves*	A04RC#F01.A0E7V	Other leavesy vegetables, SOURCE = Moringa (as plant)	Greece	Greece				leaves							1520±90	116
Puncture vine	*Tribulus terrestris L.*	A04RC	Other leavesy vegetables	India					without stems			8840				40	98
Rampion	*Phyteuma orbiculare L.*	A04RC	Other leavesy vegetables	Switzerland	Switzerland	40.9			leaves								117

Table S9.12.3 Other leavesy vegetables (A04RC) (µg/100g) (continuation)

Food name	Scientific name	FoodEx2_ TermCode	FoodEx2_ TermName	Origin (country)	Purchase (country)	Water (%)	Process	Saponification	Part analysed	Colour	Phytofluene	Violaxanthin	Z(v. cis)-lycopene	Z(v. cis)-β-carotene	Z(v. cis)-β-cryptoxanthin	Zeaxanthin	Ref.
Sheep sorrel	*Rumex acetosella L.*	A04RC	Other leavesy vegetables	India					without stems			1450					98
Spider wisp	*Gynandropsis pentaphylla L.*	A04RC	Other leavesy vegetables	India					without stems							1280	98
Watercress	*Nasturtium officinale*	A00ND	Watercresses	Spain	Spain				edible part	green							24
Watercress	*Nasturtium officinale*	A00ND	Watercresses	Spain	Spain				edible part	green							24
Watercress	*Nasturtium officinale R. Br.*	A00ND	Watercresses	Brazil					leaves	green		2610				nd	109
Winter squash	*Cucurbita maxima Duchesne*	A04RC	Other leavesy vegetables	India					without stems			15580				250	98

Table S9.13.1 Head brassica (A00FT) (µg/100g)

Food name	Scientific name	FoodEx2_ TermCode	FoodEx2_ TermName	Origin (country)	Purchase (country)	Water (%)	Process	Saponification	Part analysed	Colour	α-carotene	β-carotene	β-cryptoxanthin	ζ-carotene	Antheraxanthin	Ref.
Brussel sprouts	*Brassica oleracea L.*	A00FV	Brussels sprouts	Spain	Spain				edible part	green		77				24
Brussel sprouts	*Brassica oleracea var. gemmifera*	A00FV#F28.A0BA1	Brussels sprouts, PROCESS = Cooking and similar thermal preparation processes	USA			cooked	no			0		0			28

Table S9.13.1 Head brassica (A00FT) (µg/100g) (continuation)

Food name	Scientific name	FoodEx2_ TermCode	FoodEx2_ TermName	Origin (country)	Purchase (country)	Water (%)	Process	Saponification	Part analysed	Colour	α-carotene	β-carotene	β-cryptoxanthin	ζ-carotene	Antheraxanthin	Ref.
Brussel sprouts	*Brassica oleracea var. gemmifera*	A00FV#F28.A0BA1	Brussels sprouts, PROCESS = Cooking and similar thermal preparation processes	USA			cooked	no			0		0			28
Brussel sprouts	*Brassica oleraceae L. var. gemmifera zenker*	A00FV	Brussels sprouts	Spain	Spain	84			buds	green		77±10				24
Brussel sprouts	*Brassica oleraceae L. var. gemmifera zenker*	A00FV	Brussels sprouts	Spain	Spain	82			buds	green		162±18				24
Brussels sprouts	*Brassica oleracea L.*	A00FV	Brussels sprouts	Spain	Spain				edible part	green		162				24
Brussels sprouts	*Brassica oleracea L.*	A00FV	Brussels sprouts	Spain	Spain				edible part			162				24
Brussels sprouts	*Brassica oleracea L.*	A00FV	Brussels sprouts	Spain	Spain				edible part			77				24
Brussels sprouts	*Brassica oleracea L. cv. gemmifera*	A00FV	Brussels sprouts	Italy	Italy		steamed	no	edible part	green		700±200				118
Brussels sprouts	*Brassica oleracea L. cv. gemmifera*	A00FV	Brussels sprouts	Italy	Italy		steamed	no	edible part	green		500±0				118
Brussels sprouts	*Brassica oleracea L. cv. gemmifera*	A00FV#F28.A07KQ	Brussels sprouts, PROCESS = Freezing	Italy	Italy		raw+ frozen	no	edible part	green		1100±0				118
Brussels sprouts	*Brassica oleracea L. cv. gemmifera*	A00FV#F28.A07KQ	Brussels sprouts, PROCESS = Freezing	Italy	Italy		frozen+ boilled	no	edible part	green		1000± 100				118
Brussels sprouts	*Brassica oleracea L. cv. gemmifera*	A00FV#F28.A07KQ	Brussels sprouts, PROCESS = Freezing	Italy	Italy		frozen+ microwaved	no	edible part	green		1300±100				118

Table S9.13.1 Head brassica (A00FT) (µg/100g) (continuation)

Food name	Scientific name	FoodEx2_ TermCode	FoodEx2_ TermName	Origin (country)	Purchase (country)	Water (%)	Process	Saponification	Part analysed	Colour	α-carotene	β-carotene	β-cryptoxanthin	ζ-carotene	Antheraxanthin	Ref.
Brussels sprouts	*Brassica oleracea L. cv. gemmifera*	A00FV#F28.A 07KQ	Brussels sprouts, PROCESS = Freezing	Italy	Italy		frozen+ steamed	no	edible part	green		1000±0				118
Brussels sprouts	*Brassica oleracea L. cv. gemmifera*	A00FV#F28.A 07KQ	Brussels sprouts, PROCESS = Freezing	Italy	Italy		frozen+ steamed	no	edible part	green		1100±0				118
Brussels sprouts	*Brassica oleracea L. cv. gemmifera*	A00FV	Brussels sprouts	Italy	Italy		raw	no	edible part	green		1700± 100				118
Brussels sprouts	*Brassica oleracea L. cv. gemmifera*	A00FV	Brussels sprouts	Italy	Italy		boilled	no	edible part	green		1500± 100				118
Brussels sprouts	*Brassica oleracea L. cv. gemmifera*	A00FV	Brussels sprouts	Italy	Italy		microw aved	no	edible part	green		1000± 100				118
Brussels sprouts	*Brassica oleracea L. cv. gemmifera*	A00FV	Brussels sprouts	Italy	Italy		steamed	no	edible part	green		700±200				118
Brussels sprouts	*Brassica oleracea L. cv. gemmifera*	A00FV	Brussels sprouts	Italy	Italy		steamed	no	edible part	green		500±0				118
Brussels sprouts	*Brassica oleracea L. cv. gemmifera*	A00FV#F28.A 07KQ	Brussels sprouts, PROCESS = Freezing	Italy	Italy		raw	no	edible part	green		1100±0				118
Brussels sprouts	*Brassica oleracea L. cv. gemmifera*	A00FV#F28.A 07KQ	Brussels sprouts, PROCESS = Freezing	Italy	Italy		boilled	no	edible part	green		1000±100				118
Brussels sprouts	*Brassica oleracea L. cv. gemmifera*	A00FV#F28.A 07KQ	Brussels sprouts, PROCESS = Freezing	Italy	Italy		microw aved	no	edible part	green		1300±100				118
Brussels sprouts	*Brassica oleracea L. cv. gemmifera*	A00FV#F28.A 07KQ	Brussels sprouts, PROCESS = Freezing	Italy	Italy		steamed	no	edible part	green		1000±0				118

Table S9.13.1 Head brassica (A00FT) (µg/100g) (continuation)

Food name	Scientific name	FoodEx2_ TermCode	FoodEx2_ TermName	Origin (country)	Purchase (country)	Water (%)	Process	Saponification	Part analysed	Colour	α-carotene	β-carotene	β-cryptoxanthin	ζ-carotene	Antheraxanthin	Ref.
Brussels sprouts	*Brassica oleracea L. cv. gemmifera*	A00FV#F28.A07KQ	Brussels sprouts, PROCESS = Freezing	Italy	Italy		steamed	no	edible part	green		1100±0				118
Brussels sprouts	*Brassica oleracea var. gemmifera*	A00FV	Brussels sprouts	Germany	Germany	84.1			edible part		50	630				53
Cabbage	*Brassica oleracea L.*	A00GA	red cabbages	Spain	Spain				edible part	purple		7	tr.			24
Cabbage	*Brassica oleracea L.*	A00GA	red cabbages	Spain	Spain				edible part	purple		3	tr.			24
Cabbage	*Brassica oleracea L. var. capitata L.*	A00FY	Head cabbages	Brazil	Brazil				leaves	green	nd	38	nd			109
Cabbage	*Brassica oleracea L. var. capitata, f. alba D.C.*	A00GC	white cabbage	Spain	Spain	89			leaves	white		22±2				24
Cabbage	*Brassica oleracea L. var. capitata, f. alba D.C.*	A00GC	white cabbage	Spain	Spain	91			leaves	white		33±3				24
Cabbage	*Brassica oleracea L. var. capitata,f. D.C.*	A00FY	Head cabbages	Spain	Spain	88			leaves	red		3±0.2	tr.			24
Cabbage	*Brassica oleracea L. var. capitata,f. D.C.*	A00FY	Head cabbages	Spain	Spain	91			leaves	red		23±1	tr.			24
Cabbage	*Brassica oleracea var. capitata f. rubra*	A00GA	red cabbages	USA				no		red	0		0			28

Table S9.13.1 Head brassica (A00FT) (µg/100g) (continuation)

Food name	Scientific name	FoodEx2_ TermCode	FoodEx2_ TermName	Origin (country)	Purchase (country)	Water (%)	Process	Saponification	Part analysed	Colour	α-carotene	β-carotene	β-cryptoxanthin	ζ-carotene	Antheraxanthin	Ref.
Cabbage	*Brassica oleracea var. capitata f. rubra*	A00GA	red cabbages	Germany	Germany	87.3			edible part	red		50	1			53
Cabbage	*Brassica oleracea var. capitata f. rubra*	A00GA	red cabbages	USA				no		red	0		0			28
Savoy cabbage	*Brassica oleracea L.*	A00GB	Savoy cabbages	Spain	Spain				edible part			7				24
Savoy cabbage	*Brassica oleracea L.*	A00GB	Savoy cabbages	Spain	Spain				edible part			3				24

Table S9.13.2 Head brassica (A00FT) (µg/100g) (continuation)

Food name	Scientific name	FoodEx2_ TermCode	FoodEx2_ TermName	Origin (country)	Purchase (country)	Water (%)	Process	Saponification	Part analysed	Colour	Capsanthin	Capsorubin	Cucurbitaxanthin	E(v. trans)-α-carotene	E(v. trans)-β-carotene	Ref.
Brussel sprouts	*Brassica oleracea var. gemmifera*	A00FV#F28.A0BA1	Brussels sprouts, PROCESS = Cooking and similar thermal preparation processes	USA			cooked	no							218	28
Cabbage	*Brassica oleracea var. capitata f. rubra*	A00GA	red cabbages	USA				no		red					0	28

Table S9.13.3 Head brassica (A00FT) (µg/100g) (continuation)

Food name	Scientific name	FoodEx2_ TermCode	FoodEx2_ TermName	Origin (country)	Purchase (country)	Water (%)	Process	Saponification	Part analysed	Colour	E(v. trans)-β-cryptoxanthin	E(v. trans)-lutein	E(v. trans)-lycopene	E(v. trans)-zeaxanthin	Lactucaxanthin	Ref.
Brussel sprouts	*Brassica oleracea var. gemmifera*	A00FV#F28.A0BA1	Brussels sprouts, PROCESS = Cooking and similar thermal preparation processes	USA			cooked	no				155		0		28
Cabbage	*Brassica oleracea var. capitata f. rubra*	A00GA	red cabbages	USA				no		red		0		0		28

Table S9.13.4 Head brassica (A00FT) (µg/100g) (continuation)

Food name	Scientific name	FoodEx2_ TermCode	FoodEx2_ TermName	Origin (country)	Purchase (country)	Water (%)	Process	Saponification	Part analysed	Colour	Lutein	Luteoxanthin	Lycopene	Neoxanthin	Phytoene	Ref.
Brussel sprouts	*Brassica oleracea L.*	A00FV	Brussels sprouts	Spain	Spain				edible part	green						24
Brussel sprouts	*Brassica oleraceae L. var. gemmifera zenker*	A00FV	Brussels sprouts	Spain	Spain	84			buds	green	185±19					24
Brussel sprouts	*Brassica oleraceae L. var. gemmifera zenker*	A00FV	Brussels sprouts	Spain	Spain	82			buds	green	468±36					24
Brussels sprouts	*Brassica oleracea L.*	A00FV	Brussels sprouts	Spain	Spain				edible part	green	468					24
Brussels sprouts	*Brassica oleracea L.*	A00FV	Brussels sprouts	Spain	Spain				edible part		468					24
Brussels sprouts	*Brassica oleracea L.*	A00FV	Brussels sprouts	Spain	Spain				edible part		183					24

Table S9.13.4 Head brassica (A00FT) (µg/100g) (continuation)

Food name	Scientific name	FoodEx2_ TermCode	FoodEx2_ TermName	Origin (country)	Purchase (country)	Water (%)	Process	Saponification	Part analysed	Colour	Lutein	Luteoxanthin	Lycopene	Neoxanthin	Phytoene	Ref.
Brussels sprouts	*Brassica oleracea* L. *cv. gemmifera*	A00FV	Brussels sprouts	Italy	Italy		raw	no	edible part	green	900±100					118
Brussels sprouts	*Brassica oleracea* L. *cv. gemmifera*	A00FV	Brussels sprouts	Italy	Italy		boilled	no	edible part	green	2100±100					118
Brussels sprouts	*Brassica oleracea* L. *cv. gemmifera*	A00FV	Brussels sprouts	Italy	Italy		microwaved	no	edible part	green	1100±100					118
Brussels sprouts	*Brassica oleracea* L. *cv. gemmifera*	A00FV	Brussels sprouts	Italy	Italy		steamed	no	edible part	green	900±100					118
Brussels sprouts	*Brassica oleracea* L. *cv. gemmifera*	A00FV	Brussels sprouts	Italy	Italy		steamed	no	edible part	green	800±0					118
Brussels sprouts	*Brassica oleracea* L. *cv. gemmifera*	A00FV#F28.A07KQ	Brussels sprouts, PROCESS = Freezing	Italy	Italy		frozen	no	edible part	green	1600±100					118
Brussels sprouts	*Brassica oleracea* L. *cv. gemmifera*	A00FV#F28.A07KQ	Brussels sprouts, PROCESS = Freezing	Italy	Italy		frozen+ boilled	no	edible part	green	1700±200					118
Brussels sprouts	*Brassica oleracea* L. *cv. gemmifera*	A00FV#F28.A07KQ	Brussels sprouts, PROCESS = Freezing	Italy	Italy		frozen+ microwaved	no	edible part	green	1600±0					118
Brussels sprouts	*Brassica oleracea* L. *cv. gemmifera*	A00FV#F28.A07KQ	Brussels sprouts, PROCESS = Freezing	Italy	Italy		frozen+ steamed	no	edible part	green	1700±100					118
Brussels sprouts	*Brassica oleracea* L. *cv. gemmifera*	A00FV#F28.A07KQ	Brussels sprouts, PROCESS = Freezing	Italy	Italy		frozen+ steamed	no	edible part	green	1400±100					118
Brussels sprouts	*Brassica oleracea L. var. gemmifera*	A00FV	Brussels sprouts	Germany	Germany	84.1			edible part		2710			280		53
Cabbage	*Brassica oleracea L.*	A00GA	red cabbages	Spain	Spain				edible part	purple	23					24
Cabbage	*Brassica oleracea L.*	A00GA	red cabbages	Spain	Spain				edible part	purple	8					24

Table S9.13.4 Head brassica (A00FT) (µg/100g) (continuation)

Food name	Scientific name	FoodEx2_ TermCode	FoodEx2_ TermName	Origin (country)	Purchase (country)	Water (%)	Process	Saponification	Part analysed	Colour	Lutein	Luteoxanthin	Lycopene	Neoxanthin	Phytoene	Ref.
Cabbage	*Brassica oleracea L. var. capitata L.*	A00FY	Head cabbages	Brazil					leaves	green	18		nd	nd		109
Cabbage	*Brassica oleracea L. var. capitata, f. alba D.C.*	A00GC	white cabbage	Spain	Spain	89			leaves	white	59±2					24
Cabbage	*Brassica oleracea L. var. capitata, f. alba D.C.*	A00GC	white cabbage	Spain	Spain	91			leaves	white	93±20					24
Cabbage	*Brassica oleracea L. var. capitata,f. D.C.*	A00FY	Head cabbages	Spain	Spain	88			leaves	red	8±2					24
Cabbage	*Brassica oleracea L. var. capitata,f. D.C.*	A00FY	Head cabbages	Spain	Spain	91			leaves	red	7±1					24
Cabbage	*Brassica oleracea var. capitata f. rubra*	A00GA	red cabbages	Germany	Germany	87.3			edible part	red	150			58		53
Savoy cabbage	*Brassica oleracea L.*	A00GB	Savoy cabbages	Spain	Spain				edible part		23					24
Savoy cabbage	*Brassica oleracea L.*	A00GB	Savoy cabbages	Spain	Spain				edible part		8					24

Table S9.13.5 Head brassica (A00FT) (µg/100g) (continuation)

Food name	Scientific name	FoodEx2_ TermCode	FoodEx2_ TermName	Origin (country)	Purchase (country)	Water (%)	Process	Saponification	Part analysed	Colour	Phytofluene	Violaxanthin	Z(v. cis)-lycopene	Z(v. cis)-β-carotene	Z(v. cis)-β-cryptoxanthin	Zeaxanthin	Ref.
Brussel sprouts	*Brassica oleracea var. gemmifera*	A00FV#F28.A0BA1	Brussels sprouts, PROCESS = Cooking and similar thermal preparation processes	USA			cooked	no						42			28

Table S9.13.5 Head brassica (A00FT) (µg/100g) (continuation)

Food name	Scientific name	FoodEx2_ TermCode	FoodEx2_ TermName	Origin (country)	Purchase (country)	Water (%)	Process	Saponification	Part analysed	Colour	Phytofluene	Violaxanthin	Z(v. cis)- lycopene	Z(v. cis)-β-carotene	Z(v. cis)-β-cryptoxanthin	Zeaxanthin	Ref.
Brussels sprouts	*Brassica oleracea L. var. gemmifera*	A00FV	Brussels sprouts	Germany	Germany	84.1			edible part			1070					53
Cabbage	*Brassica oleracea L.*	A00GA	red cabbages	Spain	Spain				edible part	purple						4	24
Cabbage	*Brassica oleracea L.*	A00GA	red cabbages	Spain	Spain				edible part	purple						tr.	24
Cabbage	*Brassica oleracea L. var. capitata L.*	A00FY	Head cabbages	Brazil					leaves	green		nd				nd	109
Cabbage	*Brassica oleracea L. var. capitata, f. alba D.C.*	A00GC	white cabbage	Spain	Spain	89			leaves	white						6±2	24
Cabbage	*Brassica oleracea L. var. capitata, f. alba D.C.*	A00GC	white cabbage	Spain	Spain	91			leaves	white						6±3	24
Cabbage	*Brassica oleracea L. var. capitata,f. D.C.*	A00FY	Head cabbages	Spain	Spain	88			leaves	red						tr.	24
Cabbage	*Brassica oleracea L. var. capitata,f. D.C.*	A00FY	Head cabbages	Spain	Spain	91			leaves	red						4±1	24
Cabbage	*Brassica oleracea var. capitata f. rubra*	A00GA	red cabbages	Germany	Germany	87.3			edible part	red		80					53
Cabbage	*Brassica oleracea var. capitata f. rubra*	A00GA	red cabbages	USA				no		red				0			28
Savoy cabbage	*Brassica oleracea L.*	A00GB	Savoy cabbages	Spain	Spain				edible part							4	24

Table S9.14.1 leavesy brassica (A00FT) (µg/100g)

Food name	Scientific name	FoodEx2_ TermCode	FoodEx2_ TermName	Origin (country)	Purchase (country)	Water (%)	Process	Saponification	Part analysed	Colour	α-carotene	β-carotene	β-cryptoxanthin	ζ-carotene	Antheraxanthin	Ref.
Cabbage	*Brassica oleracea L.*	A00GE	leavesy brassica	Spain	Spain				edible part	green	90	4418	20			24
Cabbage	*Brassica oleracea L.*	A00GE	leavesy brassica	Spain	Spain				edible part	green		33				24
Cabbage	*Brassica oleracea L.*	A00GE	leavesy brassica	Spain	Spain				edible part	green		22				24
Cabbage	*Brassica oleracea L.*	A00GE	leavesy brassica	Spain	Spain				edible part		90	4418	20			24
Cabbage	*Brassica oleracea L.*	A00GE	leavesy brassica	Spain	Spain				edible part			33				24
Cabbage	*Brassica oleracea L.*	A00GE	leavesy brassica	Spain	Spain				edible part			22				24
Cabbage	*Brassica oleracea L. var. sabellica*	A00GL#F28.A 0BA1	Kales and similar-, PROCESS = Cooking and similar thermal preparation processes	USA			cooked	no			870		0			28

Table S9.14.2 leavesy brassica (A00FT) (µg/100g) (continuation)

Food name	Scientific name	FoodEx2_ TermCode	FoodEx2_ TermName	Origin (country)	Purchase (country)	Water (%)	Process	Saponification	Part analysed	Colour	Capsanthin	Capsorubin	Cucurbitaxanthin	E(v. trans)-α-carotene	E(v. trans)-β-carotene	Ref.
Cabagge	*Brassica olerácea L. var. sabellica*	A00GL#F28.A 0BA1	Kales and similar-, PROCESS = Cooking and similar thermal preparation processes	USA			cooked	no							0	28

Table S9.14.3 leavesy brassica (A00FT) (µg/100g) (continuation)

Food name	Scientific name	FoodEx2_ TermCode	FoodEx2_ TermName	Origin (country)	Purchase (country)	Water (%)	Process	Saponification	Part analysed	Colour	E(v. trans)-β-cryptoxanthin	E(v. trans)-lutein	E(v. trans)-lycopene	E(v. trans)-zeaxanthin	Lactucaxanthin	Ref.
Cabbage	*Brassica oleracea L. var. sabellica*	A00GL#F28.A0BA1	Kales and similar-, PROCESS = Cooking and similar thermal preparation processes	USA			cooked	no				8884		0		28

Table S9.14.4 leavesy brassica (A00FT) (µg/100g) (continuation)

Food name	Scientific name	FoodEx2_ TermCode	FoodEx2_ TermName	Origin (country)	Purchase (country)	Water (%)	Process	Saponification	Part analysed	Colour	Lutein	Luteoxanthin	Lycopene	Neoxanthin	Phytoene	Ref.
Cabbage	*Brassica oleracea L.*	A00GE	leavesy brassica	Spain	Spain				edible part	green	93					24
Cabbage	*Brassica oleracea L.*	A00GE	leavesy brassica	Spain	Spain				edible part	green	59					24
Cabbage	*Brassica oleracea L.*	A00GE	leavesy brassica	Spain	Spain				edible part		93					24
Cabbage	*Brassica oleracea L.*	A00GE	leavesy brassica	Spain	Spain				edible part		59					24

Table S9.14.5 leavesy brassica (A00FT) (µg/100g) (continuation)

Food name	Scientific name	FoodEx2_ TermCode	FoodEx2_ TermName	Origin (country)	Purchase (country)	Water (%)	Process	Saponification	Part analysed	Colour	Phytofluene	Violaxanthin	Z(v. cis)-lycopene	Z(v. cis)-β-carotene	Z(v. cis)-β-cryptoxanthin	Zeaxanthin	Ref.
Cabbage	*Brassica oleracea L.*	A00GE	leavesy brassica	Spain	Spain				edible part	green						6	24
Cabbage	*Brassica oleracea L.*	A00GE	leavesy brassica	Spain	Spain				edible part	green						6	24
Cabbage	*Brassica oleracea L.*	A00GE	leavesy brassica	Spain	Spain				edible part							6	24

Table S9.14.5 leavesy brassica (A00FT) (µg/100g) (continuation)

Food name	Scientific name	FoodEx2_TermCode	FoodEx2_TermName	Origin (country)	Purchase (country)	Water (%)	Process	Saponification	Part analysed	Colour	Phytofluene	Violaxanthin	Z(v. cis)-lycopene	Z(v. cis)-β-carotene	Z(v. cis)-β-cryptoxanthin	Zeaxanthin	Ref.
Cabbage	*Brassica oleracea L.*	A00GE	leavesy brassica	Spain	Spain				edible part							6	24
Cabbage	*Brassica oleracea L. var. sabellica*	A00GL#F28.A0BA1	Kales and similar-, PROCESS = Cooking and similar thermal preparation processes	USA			cooked	no						4693			28

Table S9.15.1 Chinese cabbages and similar (A00GF) (µg/100g)

Food name	Scientific name	FoodEx2_TermCode	FoodEx2_TermName	Origin (country)	Purchase (country)	Water (%)	Process	Saponification	Part analysed	Colour	α-carotene	β-carotene	β-cryptoxanthin	ζ-carotene	Antheraxanthin	Ref.
Mustard	*Brassica campestris*	A00GH	Indian mustards	Bangladesh					leaves			1404± 36.1				106
Pak choi	*Brassica rapa var. chinensis*	A00GJ#F10.A0F2S	Pak-choi, QUALITATIVE-INFO = red	Lithuania	Lithuania					red	2300 - 4670	1850 - 3340				104
Tatsoi	*Brassica rapa var. rosularis*	A0DLF	Chinese flat cabbages	Lithuania	Lithuania						2050 - 3790	1630 - 2600				104
Turnip greens	*Brassica rapa L. var. rapa*	A00LY	Turnip tops	Portugal	Portugal				leaves	green	ND	4400	nd			32

Table S9.15.2 Chinese cabbages and similar (A00GF) (µg/100g) (continuation)

Food name	Scientific name	FoodEx2_ TermCode	FoodEx2_ TermName	Origin (country)	Purchase (country)	Water (%)	Process	Saponification	Part analysed	Colour	Lutein	Luteoxanthin	Lycopene	Neoxanthin	Phytoene	Ref.
Mustard	*Brassica juncea*	A00GH	Indian mustards	Latvia	Latvia				leaves		10000					112
red pak choi	*Brassica rapa var. chinensis 'Rubi F1'*	A00GJ#F10.A0F2S	Pak-choi, QUALITATIVE-INFO = red	Lithuania	Lithuania									2080 - 20640		104
Tatsoi	*Brassica rapa var. rosularis*	A0DLF	Chinese flat cabbages	Lithuania	Lithuania									1580 - 9090		104
Turnip greens	*Brassica rapa L. var. rapa*	A00LY	Turnip tops	Portugal	Portugal				leaves	green	5600		nd			32

Table S9.15.3 Chinese cabbages and similar (A00GF) (µg/100g) (continuation)

Food name	Scientific name	FoodEx2_ TermCode	FoodEx2_ TermName	Origin (country)	Purchase (country)	Water (%)	Process	Saponification	Part analysed	Colour	Phytofluene	Violaxanthin	Z(v. cis)-lycopene	Z(v. cis)-β-carotene	Z(v. cis)-β-cryptoxanthin	Zeaxanthin	Ref.
Mustard	*Brassica campestris*	A00GH	Indian mustards	Bangladesh	Bangladesh				leaves								106
Mustard	*Brassica juncea*	A00GH	Indian mustards	Latvia	Latvia				leaves								112
Pak choi	*Brassica rapa var. chinensis 'Rubi F1'*	A00GJ#F10.A0F2S	Pak-choi, QUALITATIVE-INFO = red	Lithuania	Lithuania					red		10800 - 60560					104
Tatsoi	*Brassica rapa var. rosularis*	A0DLF	Chinese flat cabbages	Lithuania	Lithuania							8930 - 32220					104
Turnip greens	*Brassica rapa L. var. rapa*	A00LY	Turnip tops	Portugal	Portugal				leaves	green						nd	32

Table S9.16.1 Kales and similar (A00GL) (µg/100g)

Food name	Scientific name	FoodEx2_ TermCode	FoodEx2_ TermName	Origin (country)	Purchase (country)	Water (%)	Process	Saponification	Part analysed	Colour	α-carotene	β-carotene	β-cryptoxanthin	ζ-carotene	Antheraxanthin	Ref.
Cabbage	*Brassica oleracea L. var. costata DC. Glória Portugal*	A00GQ	Portuguese kales	Portugal	Portugal				leaves	green	nd	460	nd			32
Cabbage	*Brassica oleracea L. var. costata DC. Penca*	A00GQ	Portuguese kales	Portugal	Portugal				leaves	green	nd	2800	nd			32
Cabbage	*Brassica oleracea L. var. costata DC. Valhascos*	A00GQ	Portuguese kales	Portugal	Portugal				leaves	green	nd	3600	nd			32
Collards	*Brassica oleracea L. var. acephala, cv Manteiga*	A00GN	Collards	Brazil		12.4 ± 0.49			leaves	green		1661.8± 89,2				119
Kale	*Brassica oleracea L.*	A00GL	Kales and similar-	Italy	Italy							1020–7380				25
Kale	*Brassica oleracea L.*	A00GL	Kales and similar-	Spain	Spain				edible part	green		6202				24
Kale	*Brassica olerácea L.*	A00GL	Kales and similar-	Spain	Spain				edible part			6202				24
Kale	*Brassica oleracea L. cv. Manteiga*	A00GR	Stem kale	Brazil					leaves	green	nd	4120	nd			109
Kale	*Brassica oleracea L. var. sabellica*	A00GM	Curly kales	Germany	Germany	82.3			edible part		150	7280	120			53
Kale	*Brassica oleraceae L. var. acephala DC.*	A00GR	Stem kale	Portugal	Portugal				leaves	green	nd	4400	nd			32
Kohlrabi	*Brassica oleracea L. var. gongylodes*	A00GV	Kohlrabies	Germany	Germany	93.1			edible part		3	9				53

Table S9.16.2 Kales and similar (A00GL) (µg/100g) (continuation)

Food name	Scientific name	FoodEx2_ TermCode	FoodEx2_ TermName	Origin (country)	Purchase (country)	Water (%)	Process	Saponification	Part analysed	Colour	Capsanthin	Capsorubin	Cucurbitaxanthin	E(v. trans)-α-carotene	E(v. trans)-β-carotene	Ref.
Kale	*Brassica oleracea L.*	A00GL	Kales and similar-	Germany	Germany										1790± 370	113
Kale	*Brassica oleraceae L. var. acephala DC.*	A00GR	Stem kale	Portugal	Portugal				edible part	green					609± 32	57
Kale	*Brassica oleraceae L. var. acephala DC.*	A00GR	Stem kale	Portugal	Portugal				edible part	green					1730± 340	57
Kale	*Brassica oleraceae L. var. acephala DC.*	A00GR	Stem kale	Portugal	Portugal				edible part	green					5790± 900	57

Table S9.16.3 Kales and similar (A00GL) (µg/100g) (continuation)

Food name	Scientific name	FoodEx2_ TermCode	FoodEx2_ TermName	Origin (country)	Purchase (country)	Water (%)	Process	Saponification	Part analysed	Colour	E(v. trans)-β-cryptoxanthin	E(v. trans)-lutein	E(v. trans)-lycopene	E(v. trans)-zeaxanthin	Lactucaxanthin	Ref.
Kale	*Brassica oleracea L.*	A00GL	Kales and similar-	Germany	Germany							4110± 920		150±30		113
Kale	*Brassica oleraceae L. var. acephala DC.*	A00GR	Stem kale	Portugal	Portugal				edible part	green	62.4±7.8	11250± 730				57
Kale	*Brassica oleraceae L. var. acephala DC.*	A00GR	Stem kale	Portugal	Portugal				edible part	green		8410± 330				57
Kale	*Brassica oleraceae L. var. acephala DC.*	A00GR	Stem kale	Portugal	Portugal				edible part	green		10280± 830				57

Table S9.16.4 Kales and similar (A00GL) (µg/100g) (continuation)

Food name	Scientific name	FoodEx2_ TermCode	FoodEx2_ TermName	Origin (country)	Purchase (country)	Water (%)	Process	Saponification	Part analysed	Colour	Lutein	Luteoxanthin	Lycopene	Neoxanthin	Phytoene	Ref.
Kale	*Brassica oleracea L.*	A00GL	Kales and similar-	Italy	Italy						4800–11470					25
Kale	*Brassica oleracea L. var. sabellica*	A00GM	Curly kales	Germany	Germany	82.3			edible part		18630			660		53
Kale	*Brassica oleracea L. var. sabellica L.*	A00GM	Curly kales	Germany	Germany				Kale extract	green	0.341± 0.108					120
Cabbage	*Brassica oleracea L. var. costata DC. Glória Portugal*	A00GQ	Portuguese kales	Portugal	Portugal				leaves	green	520		nd			32
Cabbage	*Brassica oleracea L. var. costata DC. Penca*	A00GQ	Portuguese kales	Portugal	Portugal				leaves	green	3300		nd			32
Cabbage	*Brassica oleracea L. var. costata DC. Valhascos*	A00GQ	Portuguese kales	Portugal	Portugal				leaves	green	4700		nd			32
Kale	*Brassica oleracea L. cv. Manteiga*	A00GR	Stem kale	Brazil					leaves	green	5252		nd	1178		109
Kale	*Brassica oleraceae L. var. acephala DC.*	A00GR	Stem kale	Portugal	Portugal				leaves	green	5600		nd			32
Kohlrabi	*Brassica oleracea L. var. gongylodes*	A00GV	Kohlrabies	Germany	Germany	93.1			edible part		12					53

Table S9.16.5 Kales and similar (A00GL) (µg/100g) (continuation)

Food name	Scientific name	FoodEx2_ TermCode	FoodEx2_ TermName	Origin (country)	Purchase (country)	Water (%)	Process	Saponification	Part analysed	Colour	Phytofluene	Violaxanthin	Z(v. cis)-lycopene	Z(v. cis)-β-carotene	Z(v. cis)-β-cryptoxanthin	Zeaxanthin	Ref.
Cabbage	*Brassica oleracea L. var. costata DC. Glória Portugal*	A00GQ	Portuguese kales	Portugal	Portugal				leaves	green						ND	32
Cabbage	*Brassica oleracea L. var. costata DC. Penca*	A00GQ	Portuguese kales	Portugal	Portugal				leaves	green						nd	32
Cabbage	*Brassica oleracea L. var. costata DC. Valhascos*	A00GQ	Portuguese kales	Portugal	Portugal				leaves	green						nd	32
Kale	*Brassica oleracea L. cv. Manteiga*	A00GR	Stem kale	Brazil					leaves	green		3010				nd	109
Kale	*Brassica oleracea L. var. sabellica*	A00GM	Curly kales	Germany	Germany	82.3			edible part			5810					53
Kale	*Brassica oleracea L. var. sabellica L.*	A00GM	Curly kales	Germany	Germany				kale extract	green						0.050±0.036	120
Kale	*Brassica oleraceae L. var. acephala DC.*	A00GR	Stem kale	Portugal	Portugal				leaves	green						nd	153
Kohlrabi	*Brassica oleracea L. var. gongylodes*	A00GV	Kohlrabies	Germany	Germany	93.1			edible part			30				3	53

Table S9.17.1 Sprouts, shoots and similar (A00S) (µg/100g)

Food name	Scientific name	FoodEx2_ TermCode	FoodEx2_ TermName	Origin (country)	Purchase (country)	Water (%)	Process	Saponification	Part analysed	Colour	α-carotene	β-carotene	β-cryptoxanthin	ζ-carotene	Antheraxanthin	Ref.
Black bryony	*Tamus communis*	A00SF	Sprouts, shoots and similar	Spain	Spain	87			young shoots	green		4.58 (3.20-5.99)				91
Cress	*Lepidium sativum L.*	A00LK	Cresses	Italy	Italy							2720–3690				25

Table S9.17.2 Sprouts, shoots and similar (A00S) (µg/100g) (continuation)

Food name	Scientific name	FoodEx2_ TermCode	FoodEx2_ TermName	Origin (country)	Purchase (country)	Water (%)	Process	Saponification	Part analysed	Colour	Capsanthin	Capsorubin	Cucurbitaxanthin	E(v. trans)-α-carotene	E(v. trans)-β-carotene	Ref.
Pea shoots	*Pisum sativum*	A00ST	Peas shoots and sprouts	Portugal	Portugal				leaves	green					13200-13900	121

Table S9.17.3 Sprouts, shoots and similar (A00S) (µg/100g) (continuation)

Food name	Scientific name	FoodEx2_ TermCode	FoodEx2_ TermName	Origin (country)	Purchase (country)	Water (%)	Process	Saponification	Part analysed	Colour	Lutein	Luteoxanthin	Lycopene	Neoxanthin	Phytoene	Ref.
Cress	*Lepidium sativum L.*	A00LK	Cresses	Italy	Italy						5610–7540					25
Black bryony	*Tamus communis*	A00SF	Sprouts, shoots and similar	Spain	Spain	87			young shoots	green	10.54 (7.04-17.35)			10.55 (7.66-18.97)		91
Pea shoots	*Pisum sativum*	A00ST	Peas shoots and sprouts	Portugal	Portugal				leaves	green	12100-12700	400-1200		1500-1600		121

Table S9.17.4 Sprouts, shoots and similar (A00S) (µg/100g) (continuation)

Food name	Scientific name	FoodEx2_ TermCode	FoodEx2_ TermName	Origin (country)	Purchase (country)	Water (%)	Process	Saponification	Part analysed	Colour	Phytofluene	Violaxanthin	Z(v. cis)-lycopene	Z(v. cis)-β-carotene	Z(v. cis)-β-cryptoxanthin	Zeaxanthin	Ref.
Black bryony	*Tamus communis*	A00SF	Sprouts, shoots and similar	Spain	Spain	87			young shoots	green		5.10 (3.80-10.93)					91
Pea shoots	*Pisum sativum*	A00ST	Peas shoots and sprouts	Portugal	Portugal				leaves	green		300-600		3100±100			121

Table S9.18.1 Flowering brassica (A00FL) (µg/100g)

Food name	Scientific name	FoodEx2_ TermCode	FoodEx2_ TermName	Origin (country)	Purchase (country)	Water (%)	Process	Saponification	Part analysed	Colour	α-carotene	β-carotene	β-cryptoxanthin	ζ-carotene	Antheraxanthin	Ref.
Broccoli	*Brassica oleracea L*	A00FN	Broccoli	Netherlands	United Kingdom				tough stalk remove d	green		120±20				95
Broccoli	*Brassica olerácea L*	A00FN#F28.A 07KQ	Broccoli, PROCESS = Freezing	Italy	Italy		frozen+st eamed	no	edible part	green		3800± 200				118
Broccoli	*Brassica oleracea L.*	A00FN	Broccoli	Spain	Spain				edible part	green	1	450				24
Broccoli	*Brassica oleracea L.*	A00FN	Broccoli	Spain	Spain				edible part	green		414				24
Broccoli	*Brassica oleracea L.*	A00FN	Broccoli	Spain	Spain	89			buds+ stalk	green		414±20				24
Broccoli	*Brassica oleracea L.*	A00FN	Broccoli	Spain	Spain	89			buds+ stalk	green		450±40				24
Broccoli	*Brassica olerácea L.*	A00FN	Broccoli	Spain	Spain				edible part	green	1	450				24
Broccoli	*Brassica olerácea L.*	A00FN	Broccoli	Spain	Spain				edible part	green		414				24
Broccoli	*Brassica olerácea L.*	A00FN#F28.A 07KQ	Broccoli, PROCESS = Freezing	Italy	Italy		frozen	no	edible part	green		9400± 300				24

Table S9.18.1 Flowering brassica (A00FL) (µg/100g) (continuation)

Food name	Scientific name	FoodEx2_ TermCode	FoodEx2_ TermName	Origin (country)	Purchase (country)	Water (%)	Process	Saponification	Part analysed	Colour	α-carotene	β-carotene	β-cryptoxanthin	ζ-carotene	Antheraxanthin	Ref.
Broccoli	*Brassica olerácea L. cv.Italica*	A00FN	Broccoli	Italy	Italy		steamed	no	edible part	green		5500± 600				24
Broccoli	*Brassica olerácea L. cv.Italica*	A00FN#F28.A07KQ	Broccoli, PROCESS = Freezing	Italy	Italy		frozen+ microwaved	no	edible part	green		3800± 500				24
Broccoli	*Brassica oleracea L. var. botrytis*	A00FN	Broccoli	India	India				without stems			3850				98
Broccoli	*Brassica oleracea L. var. italica*	A00FN	Broccoli	Italy	Italy						nd	291–1750	nd			25
Broccoli	*Brassica oleracea L. var. italica*	A00FN	Broccoli	Germany	Germany	89.4			edible part			280	11			53
Broccoli	*Brassica oleracea L. var. italica*	A00FN	Broccoli	Spain	Spain					green		0.81				122
Broccoli	*Brassica oleracea L. var. italica*	A00FN#F28.A0BA1	Broccoli, PROCESS = Cooking and similar thermal preparation processes	USA			cooked	no			0		0			28
Broccoli	*Brassica oleracea L. var. italica Plenck*	A00FN	Broccoli	Brazil					root flower heads	green	nd	1893	nd			109
Broccoli	*Brassica olerácea L. var.Italica*	A00FN	Broccoli	Italy	Italy		boilled	no	edible part	green		4100± 1200				118
Broccoli	*Brassica olerácea L. var.Italica*	A00FN	Broccoli	Italy	Italy		microwaved	no	edible part	green		2000± 200				118
Broccoli	*Brassica olerácea L. var.Italica*	A00FN	Broccoli	Italy	Italy		steamed	no	edible part	green		4800± 700				118

Table S9.18.1 Flowering brassica (A00FL) (μg/100g) (continuation)

Food name	Scientific name	FoodEx2_ TermCode	FoodEx2_ TermName	Origin (country)	Purchase (country)	Water (%)	Process	Saponification	Part analysed	Colour	α-carotene	β-carotene	β-cryptoxanthin	ζ-carotene	Antheraxanthin	Ref.
Broccoli	*Brassica olerácea L. var.Italica*	A00FN#F28.A 07KQ	Broccoli, PROCESS = Freezing	Italy	Italy		frozen+ boilled	no	edible part	green		6500± 600				118
Broccoli	*Brassica olerácea L. var.Italica*	A00FN#F28.A 07KQ	Broccoli, PROCESS = Freezing	Italy	Italy		frozen+st eamed	no	edible part	green		3700± 100				118
Broccoli	*Brassica oleracea L., var. italica*	A00FN	Broccoli	Spain	Spain				heads	green		800±30				123
Broccoli	*Brassica oleraceaL. var.Italica*	A00FN	Broccoli	Italy	Italy		raw	no	edible part	green		5000± 400				121
Cauliflower	*Brassica oleracea L.*	A00FR	Cauliflowers	Spain	Spain				edible part	white		7				24
Cauliflower	*Brassica oleracea L.*	A00FR	Cauliflowers	Spain	Spain				edible part	white		2				24
Cauliflower	*Brassica oleracea L.*	A00FR	Cauliflowers	Spain	Spain				edible part			7				24
Cauliflower	*Brassica oleracea L.*	A00FR	Cauliflowers	Spain	Spain				edible part			2				24
Cauliflower	*Brassica oleracea L. var. botrytis*	A00FR	Cauliflowers	Germany	Germany	92.7			edible part			2	1			53
Cauliflower	*Brassica oleracea L. var.botrytis f. cauliflora Duch.*	A00FR	Cauliflowers	Spain	Spain	93			buds+ stalk	white		2±0.2				24
Cauliflower	*Brassica oleracea L. var.botrytis f. cauliflora Duch.*	A00FR	Cauliflowers	Spain	Spain	91			buds+ stalk	white		7±1				24

Table S9.18.2 Flowering brassica (A00FL) (μg/100g) (continuation)

Food name	Scientific name	FoodEx2_ TermCode	FoodEx2_ TermName	Origin (country)	Purchase (country)	Water (%)	Process	Saponification	Part analysed	Colour	Capsanthin	Capsorubin	Cucurbitaxanthin	E(v. trans)-α-carotene	E(v. trans)-β-carotene	Ref.
Broccoli	*Brassica oleracea var. italica*	A00FN#F28.A0BA1	Broccoli, PROCESS = Cooking and similar thermal preparation processes	USA			cooked	no							362	28

Table S9.18.3 Flowering brassica (A00FL) (μg/100g) (continuation)

Food name	Scientific name	FoodEx2_ TermCode	FoodEx2_ TermName	Origin (country)	Purchase (country)	Water (%)	Process	Saponification	Part analysed	Colour	E(v. trans)-β-cryptoxanthin	E(v. trans)-lutein	E(v. trans)-lycopene	E(v. trans)-zeaxanthin	Lactucaxanthin	Ref.
Broccoli	*Brassica oleracea var. italica*	A00FN#F28.A0BA1	Broccoli, PROCESS = Cooking and similar thermal preparation processes	USA			cooked	no				772		0		28

Table S9.18.4 Flowering brassica (A00FL) (µg/100g) (continuation)

Food name	Scientific name	FoodEx2_ TermCode	FoodEx2_ TermName	Origin (country)	Purchase (country)	Water (%)	Process	Saponification	Part analysed	Colour	Lutein	Luteoxanthin	Lycopene	Neoxanthin	Phytoene	Ref.
Broccoli	*Brassica oleracea L*	A00FN	Broccoli	Netherlands	United Kingdom				tough stalk removed	green	190±30			30±10		95
Broccoli	*Brassica oleracea L.*	A00FN	Broccoli	Spain	Spain				edible part	green	1043					24
Broccoli	*Brassica oleracea L.*	A00FN	Broccoli	Spain	Spain				edible part	green	1108					24
Broccoli	*Brassica oleracea L.*	A00FN	Broccoli	Spain	Spain	89			buds+ stalk	green	1108±50					24
Broccoli	*Brassica oleracea L.*	A00FN	Broccoli	Spain	Spain	89			buds+ stalk	green	1043					24
Broccoli	*Brassica olerácea L.*	A00FN	Broccoli	Spain	Spain				edible part	green	1043					24
Broccoli	*Brassica olerácea L.*	A00FN	Broccoli	Spain	Spain				edible part	green	1108					24
Broccoli	*Brassica oleracea L. var. botrytis*	A00FN	Broccoli	India					without stems		4500			520		98
Broccoli	*Brassica oleracea L. var. italica*	A00FN	Broccoli	Spain	Spain				heads	green	600±10					123
Broccoli	*Brassica oleracea L. var. italica*	A00FN	Broccoli	Spain	Spain				inflores cences	green	1,38					122
Broccoli	*Brassica oleracea L. var. italica*	A00FN	Broccoli	Spain	Spain				inflores cences	green	1.86					122
Broccoli	*Brassica olerácea L. var. italica*	A00FN	Broccoli	Italy	Italy						707–3300		nd			25
Broccoli	*Brassica olerácea L. var. italica*	A00FN	Broccoli	Germany	Germany	89.4			edible part		800			20		53

Table S9.18.4 Flowering brassica (A00FL) (μg/100g) (continuation)

Food name	Scientific name	FoodEx2_ TermCode	FoodEx2_ TermName	Origin (country)	Purchase (country)	Water (%)	Process	Saponification	Part analysed	Colour	Lutein	Luteoxanthin	Lycopene	Neoxanthin	Phytoene	Ref.
Broccoli	*Brassica oleracea L. var. italica Plenck*	A00FN	Broccoli	Brazil					root flower heads	green	3510		nd	747		109
Broccoli	*Brassica olerácea L. var.Italica*	A00FN	Broccoli	Italy	Italy		raw	no	edible part	green	8400±400					121
Broccoli	*Brassica olerácea L. var.Italica*	A00FN	Broccoli	Italy	Italy		boilled	no	edible part	green	5600± 1100					118
Broccoli	*Brassica olerácea L. var.Italica*	A00FN	Broccoli	Italy	Italy		microw aved	no	edible part	green	5500±0					118
Broccoli	*Brassica olerácea L. var.Italica*	A00FN	Broccoli	Italy	Italy		steamed	no	edible part	green	8800± 1300					118
Broccoli	*Brassica olerácea L. var.Italica*	A00FN	Broccoli	Italy	Italy		steamed	no	edible part	green	10500± 1100					118
Broccoli	*Brassica olerácea L. var.Italica*	A00FN#F28.A 07KQ	Broccoli, PROCESS = Freezing	Italy	Italy		frozen	no	edible part	green	12300± 400					118
Broccoli	*Brassica olerácea L. var.Italica*	A00FN#F28.A 07KQ	Broccoli, PROCESS = Freezing	Italy	Italy		frozen+ boilled	no	edible part	green	8800±200					118
Broccoli	*Brassica olerácea L. var.Italica*	A00FN#F28.A 07KQ	Broccoli, PROCESS = Freezing	Italy	Italy		frozen+ microw aved	no	edible part	green	6000±500					118
Broccoli	*Brassica olerácea L. var.Italica*	A00FN#F28.A 07KQ	Broccoli, PROCESS = Freezing	Italy	Italy		frozen+ steamed	no	edible part	green	6900±200					118
Broccoli	*Brassica olerácea L. var.Italica*	A00FN#F28.A 07KQ	Broccoli, PROCESS = Freezing	Italy	Italy		frozen+st eamed	no	edible part	green	5800±500					118
Cauliflower	*Brassica oleracea L.*	A00FR	Cauliflowers	Spain	Spain				edible part	white	15					24
Cauliflower	*Brassica oleracea L.*	A00FR	Cauliflowers	Spain	Spain				edible part	white	4					24

Table S9.18.4 Flowering brassica (A00FL) (µg/100g) (continuation)

Food name	Scientific name	FoodEx2_ TermCode	FoodEx2_ TermName	Origin (country)	Purchase (country)	Water (%)	Process	Saponification	Part analysed	Colour	Lutein	Luteoxanthin	Lycopene	Neoxanthin	Phytoene	Ref.
Cauliflower	*Brassica oleracea L.*	A00FR	Cauliflowers	Spain	Spain				edible part		15					24
Cauliflower	*Brassica oleracea L.*	A00FR	Cauliflowers	Spain	Spain				edible part		4					24
Cauliflower	*Brassica oleracea L. var. botrytis*	A00FR	Cauliflowers	Germany	Germany	92.7			edible part		15					53
Cauliflower	*Brassica oleracea L. var.botrytis f. cauliflora Duch.*	A00FR	Cauliflowers	Spain	Spain	93			buds+ stalk	white	4±0.4					24
Cauliflower	*Brassica oleracea L. var.botrytis f. cauliflora Duch.*	A00FR	Cauliflowers	Spain	Spain	91			buds+ stalk	white	15±1					24

Table S9.18.5 Flowering brassica (A00FL) (µg/100g) (continuation)

Food name	Scientific name	FoodEx2_ TermCode	FoodEx2_ TermName	Origin (country)	Purchase (country)	Water (%)	Process	Saponification	Part analysed	Colour	Phytofluene	Violaxanthin	Z(v. cis)- lycopene	Z(v. cis)-β- carotene	Z(v. cis)-β- cryptoxanthin	Zeaxanthin	Ref.
Broccoli	*Brassica oleracea L*	A00FN	Broccoli	Netherlands	United Kingdom				tough stalk removed	green		80±20					95
Broccoli	*Brassica oleracea L. var. botrytis*	A00FN	Broccoli	India					without stems			1450					98
Broccoli	*Brassica oleracea L. var. italica*	A00FN	Broccoli	Germany	Germany	89.4			edible part			180					53

Table S9.18.5 Flowering brassica (A00FL) (µg/100g) (continuation)

Food name	Scientific name	FoodEx2_ TermCode	FoodEx2_ TermName	Origin (country)	Purchase (country)	Water (%)	Process	Saponification	Part analysed	Colour	Phytofluene	Violaxanthin	Z(v. cis)-lycopene	Z(v. cis)-β-carotene	Z(v. cis)-β-cryptoxanthin	Zeaxanthin	Ref.
Broccoli	*Brassica olerácea L. var. italica*	A00FN#F28.A0BA1	Broccoli, PROCESS = Cooking and similar thermal preparation processes	USA	USA		cooked	no						43			28
Broccoli	*Brassica oleracea L. var. italica*	A00FN	Broccoli	Brazil	Brazil				root flower heads	green		530				nd	109
Cauliflower	*Brassica oleracea L.*	A00FR	Cauliflowers	Spain	Spain				edible part	white						tr.	24
Cauliflower	*Brassica oleracea L. var. botrytis*	A00FR	Cauliflowers	Germany	Germany	92.7			edible part			9					53

Table S9.19.1 Flowers used as vegetables (A0ESZ) (µg/100g)

Food name	Scientific name	FoodEx2_ TermCode	FoodEx2_ TermName	Origin (country)	Purchase (country)	Water (%)	Process	Saponification	Part analysed	Colour	α-carotene	β-carotene	β-cryptoxanthin	ζ-carotene	Antheraxanthin	Ref.
Celosia	*Allmania nodiflora L. R.Br.*	A0ESZ	Flowers used as vegetables	India					without stems			9630				98
Rampion	*Phyteuma orbiculare L..*	A0DGF	Other edible flowers	Switzerland	Switzerland	70.7			flowers			700				117

Table S9.19.2 Flowers used as vegetables (A0ESZ) (µg/100g) (continuation)

Food name	Scientific name	FoodEx2_ TermCode	FoodEx2_ TermName	Origin (country)	Purchase (country)	Water (%)	Process	Saponification	Part analysed	Colour	Lutein	Luteoxanthin	Lycopene	Neoxanthin	Phytoene	Ref.
Celosia	Allmania nodiflora L. R.Br.	A0ESZ	Flowers used as vegetables	India					without stems		23100			7740		98

Table S9.19.3 Flowers used as vegetables (A0ESZ) (µg/100g) (continuation)

Food name	Scientific name	FoodEx2_ TermCode	FoodEx2_ TermName	Origin (country)	Purchase (country)	Water (%)	Process	Saponification	Part analysed	Colour	Phytofluene	Violaxanthin	Z(v. cis)-lycopene	Z(v. cis)-β-carotene	Z(v. cis)-β-cryptoxanthin	Zeaxanthin	Ref.
Celosia	Allmania nodiflora L. R.Br.	A0ESZ	Flowers used as vegetables	India	India				without stems			8900				180	98

Table S9.20.1 Stems/stalks eaten as vegetables (A00RR) (µg/100g)

Food name	Scientific name	FoodEx2_ TermCode	FoodEx2_ TermName	Origin (country)	Purchase (country)	Water (%)	Process	Saponification	Part analysed	Colour	α-carotene	β-carotene	β-cryptoxanthin	ζ-carotene	Antheraxanthin	Ref.
Artichoke	*Cynara scolimus L.*	A00RS	Globe artichokes	Spain	Spain				edible part	green		59				24
Artichoke	*Cynara scolimus L.*	A00RS	Globe artichokes	Spain	Spain				edible part	green		47				24
Artichoke	*Cynara scolimus L.*	A00RS	Globe artichokes	Spain	Spain	84			inflores cence	green	47±5					24
Artichoke	*Cynara scolimus L.*	A00RS	Globe artichokes	Spain	Spain	89			inflores cence	green		59±1				24
Artichoke	*Cynara scolymus L.*	A00RS	Globe artichokes	Spain	Spain				edible part			59				24

Table S9.20.1 Stems/stalks eaten as vegetables (A00RR) (µg/100g)

Food name	Scientific name	FoodEx2_ TermCode	FoodEx2_ TermName	Origin (country)	Purchase (country)	Water (%)	Process	Saponification	Part analysed	Colour	α-carotene	β-carotene	β-cryptoxanthin	ζ-carotene	Antheraxanthin	Ref.
Artichoke	*Cynara scolymus L.*	A00RS	Globe artichokes	Spain	Spain				edible part			47				24
Artichoke heart	*Cynara cardunculus L. Globe artichoke group*	A00RS	Globe artichokes	USA				no			0		0			28
Asparagus	*Asparagus officinalis L.*	A00RT	Asparagus	Spain	Spain				edible part	green		387				24
Asparagus	*Asparagus officinalis L.*	A00RT	Asparagus	Spain	Spain				edible part	green		320				24
Asparagus	*Asparagus officinalis L.*	A00RT	Asparagus	Spain	Spain				edible part	green		387				24
Asparagus	*Asparagus officinalis L.*	A00RT	Asparagus	Spain	Spain				edible part	green		320				24
Asparagus	*Asparagus officinalis L.*	A00RT	Asparagus	Spain	Spain	93			stalk	green		320±50				24
Asparagus	*Asparagus officinalis L.*	A00RT	Asparagus	Spain	Spain	93			stalk	green		387±49				24
Asparagus	*Cynara scolimus L.*	A00RT#F28.A0BA1	Asparagus, PROCESS = Cooking and similar thermal preparation processes	USA			cooked	no			0		10			28
Celery	*Apium graveolens*	A00RY	Celeries	Spain	Spain				edible part	green		1109				24
Celery	*Apium graveolens*	A00RY	Celeries	Spain	Spain				edible part	green		570				24
Celery	*Apium graveolens L.*	A00RY	Celeries	Spain	Spain				edible part	white		65				24
Celery	*Apium graveolens L.*	A00RY	Celeries	Spain	Spain				edible part	green		1109				24
Celery	*Apium graveolens L.*	A00RY	Celeries	Spain	Spain				edible part	green		570				24
Celery	*Apium graveolens L.*	A00RY	Celeries	Spain	Spain	93			leaves+ stalk	green		570±14				24
Celery	*Apium graveolens L.*	A00RY	Celeries	Spain	Spain	92			leaves+ stalk	green		1109±77				24

Table S9.20.1 Stems/stalks eaten as vegetables (A00RR) (µg/100g)

Food name	Scientific name	FoodEx2_ TermCode	FoodEx2_ TermName	Origin (country)	Purchase (country)	Water (%)	Process	Saponification	Part analysed	Colour	α-carotene	β-carotene	β-cryptoxanthin	ζ-carotene	Antheraxanthin	Ref.
Celery	*Apium graveolens L.*	A00RY	Celeries	Spain	Spain	92			leaves+ stalk	green		517±67				24
Celery	*Apium graveolens L.*	A00RY	Celeries	Spain	Spain	94			leaves+ stalk	green		1088±46				24
Celery	*Apium graveolens L.*	A00RY	Celeries	Spain	Spain	94			leaves+ stalk	white		65±2				24
Celery	*Apium graveolens L.*	A00RY	Celeries	Spain	Spain	94			leaves+ stalk	white		58±8				24
Ladies' seal	*Bryonia dioica*	A00RR	Stems/stalks eaten as vegetables	Spain	Spain	84.3			young shoots	green		6.69 (1.49-19.53)				91
Leek	*Allium porrum L.*	A00SB	Leeks	Spain	Spain				edible part			99				24
Leek	*Allium porrum L.*	A00SB	Leeks	Spain	Spain				edible part			51				24
Leek	*Allium porrum, L.*	A00SB	Leeks	Italy	Italy						nd	3190	nd			25
Leek	*Allium porrum, L.*	A00SB	Leeks	Spain	Spain				edible part	green		99				24
Leek	*Allium porrum, L.*	A00SB	Leeks	Spain	Spain				edible part	green		51				24
Leek	*Allium porrum, L.*	A00SB	Leeks	Spain	Spain	86			bulb	green		51±4				24
Leek	*Allium porrum, L.*	A00SB	Leeks	Spain	Spain	90			bulb	green		99±7				24
Leek	*Allium porrum, L.*	A00SB	Leeks	Spain	Spain	95			bulb	green		13±2				24
Thistle	*Cynara carduncullus*	A00RX	Cardoons	Spain	Spain				edible part		49	3954				24
Thistle	*Cynara carduncunlus*	A00RX	Cardoons	Spain	Spain				edible part	green	49	3954				24

Table S9.20.2 Stems/stalks eaten as vegetables (A00RR) (µg/100g) (continuation)

Food name	Scientific name	FoodEx2_ TermCode	FoodEx2_ TermName	Origin (country)	Purchase (country)	Water (%)	Process	Saponification	Part analysed	Colour	Capsanthin	Capsorubin	Cucurbitaxanthin	E(v. trans)-α-carotene	E(v. trans)-β-carotene	Ref.
Artichoke	*Cynara cardunculus L. Globe artichoke group*	A00RS	Globe artichokes	USA				no							14	28
Asparagus	*Asparagus officinalis L. subsp. scolymus*	A00RT#F28.A0BA1	Asparagus, PROCESS = Cooking and similar thermal preparation processes	USA			cooked	no							265	28

Table S9.20.3 Stems/stalks eaten as vegetables (A00RR) (µg/100g) (continuation)

Food name	Scientific name	FoodEx2_ TermCode	FoodEx2_ TermName	Origin (country)	Purchase (country)	Water (%)	Process	Saponification	Part analysed	Colour	E(v. trans)-β-cryptoxanthin	E(v. trans)-lutein	E(v. trans)-lycopene	E(v. trans)-zeaxanthin	Lactucaxanthin	Ref.
Artichoke heart	*Cynara cardunculus L. Globe artichoke group*	A00RS	Globe artichokes	USA				no				62		18		28
Asparagus	*Asparagus officinalis L. subsp. scolymus*	A00RT#F28.A0BA1	Asparagus, PROCESS = Cooking and similar thermal preparation processes	USA			cooked	no				991		0		28

Table S9.20.4 Stems/stalks eaten as vegetables (A00RR) (µg/100g) (continuation)

Food name	Scientific name	FoodEx2_ TermCode	FoodEx2_ TermName	Origin (country)	Purchase (country)	Water (%)	Process	Saponification	Part analysed	Colour	Lutein	Luteoxanthin	Lycopene	Neoxanthin	Phytoene	Ref.
Artichoke	*Cynara scolimus L.*	A00RS	Globe artichokes	Spain	Spain				edible part	green	275					24
Artichoke	*Cynara scolimus L.*	A00RS	Globe artichokes	Spain	Spain				edible part	green	163					24
Artichoke	*Cynara scolimus L.*	A00RS	Globe artichokes	Spain	Spain	84			inflores cence	green	163±15					24
Artichoke	*Cynara scolimus L.*	A00RS	Globe artichokes	Spain	Spain	89			inflores cence	green	275±23					24
Artichoke	*Cynara scolymus L.*	A00RS	Globe artichokes	Spain	Spain				edible part		275					24
Artichoke	*Cynara scolymus L.*	A00RS	Globe artichokes	Spain	Spain				edible part		163					24
Asparagus	*Asparagus officinalis L.*	A00RT	Asparagus	Spain	Spain				edible part	green	738					24
Asparagus	*Asparagus officinalis L.*	A00RT	Asparagus	Spain	Spain				edible part	green	609					24
Asparagus	*Asparagus officinalis L.*	A00RT	Asparagus	Spain	Spain				edible part	green	738					24
Asparagus	*Asparagus officinalis L.*	A00RT	Asparagus	Spain	Spain				edible part	green	609					24
Asparagus	*Asparagus officinalis L.*	A00RT	Asparagus	Spain	Spain	93			stalk	green	609±20					24
Asparagus	*Asparagus officinalis L.*	A00RT	Asparagus	Spain	Spain	93			stalk	green	738±25					24
Celery	*Apium graveolens L.*	A00RY	Celeries	Spain	Spain				edible part	white	163					24
Celery	*Apium graveolens L.*	A00RY	Celeries	Spain	Spain				edible part	green	1335					24
Celery	*Apium graveolens L.*	A00RY	Celeries	Spain	Spain				edible part	green	860					24
Celery	*Apium graveolens L.*	A00RY	Celeries	Spain	Spain				edible part	green	1335					24
Celery	*Apium graveolens L.*	A00RY	Celeries	Spain	Spain				edible part	green	860					24
Celery	*Apium graveolens L.*	A00RY	Celeries	Spain	Spain	93			leaves+ stalk	green	860±17					24
Celery	*Apium graveolens L.*	A00RY	Celeries	Spain	Spain	92			leaves+ stalk	green	1335±91					24

Table S9.20.4 Stems/stalks eaten as vegetables (A00RR) (µg/100g) (continuation)

Food name	Scientific name	FoodEx2_ TermCode	FoodEx2_ TermName	Origin (country)	Purchase (country)	Water (%)	Process	Saponification	Part analysed	Colour	Lutein	Luteoxanthin	Lycopene	Neoxanthin	Phytoene	Ref.
Celery	*Apium graveolens L.*	A00RY	Celeries	Spain	Spain	92			leaves+ stalk	green	779±107					24
Celery	*Apium graveolens L.*	A00RY	Celeries	Spain	Spain	94			leaves+ stalk	green	1338±56					24
Celery	*Apium graveolens L.*	A00RY	Celeries	Spain	Spain	94			leaves+ stalk	white	163±10					24
Celery	*Apium graveolens L.*	A00RY	Celeries	Spain	Spain	94			leaves+ stalk	white	150±10					24
Ladies' seal	*Bryonia dioica*	A00RR	Stems/stalks eaten as vegetables	Spain	Spain	84.3			young shoots	green	19.13 (6.83-36.98)			17.37 (1.72-38.33)		91
Leek	*Allium porrum L.*	A00SB	Leeks	Spain	Spain				edible part		171					24
Leek	*Allium porrum L.*	A00SB	Leeks	Spain	Spain				edible part		76					24
Leek	*Allium porrum, L.*	A00SB	Leeks	Italy	Italy						3680		nd			25
Leek	*Allium porrum, L.*	A00SB	Leeks	Spain	Spain				edible part	green	171					24
Leek	*Allium porrum, L.*	A00SB	Leeks	Spain	Spain				edible part	green	76					24
Leek	*Allium porrum, L.*	A00SB	Leeks	Spain	Spain	86			bulb	green	76±6					24
Leek	*Allium porrum, L.*	A00SB	Leeks	Spain	Spain	90			bulb	green	171±14					24
Leek	*Allium porrum, L.*	A00SB	Leeks	Spain	Spain	95			bulb	green	26±1					24
Rhubarb	*Rheum rhabarbarum L. or Rheum x hybridum Murray*	A00SD	Rhubarbs	Italy	Italy								120			25

Table S9.20.5 Stems/stalks eaten as vegetables (A00RR) (μg/100g) (continuation)

Food name	Scientific name	FoodEx2_ TermCode	FoodEx2_ TermName	Origin (country)	Purchase (country)	Water (%)	Process	Saponification	Part analysed	Colour	Phytofluene	Violaxanthin	Z(v. cis)- lycopene	Z(v. cis)-β- carotene	Z(v. cis)-β- cryptoxanthin	Zeaxanthin	Ref.
Artichoke heart	*Cynara cardunculus L. Globe artichoke group*	A00RS	Globe artichokes	USA	USA			no						0			28
Asparagus	*Asparagus officinalis L. subsp. Scolymus*	A00RT#F28.A0BA1	Asparagus, PROCESS = Cooking and similar thermal preparation processes	USA	USA		cooked	no						45			28
Ladies' seal	*Bryonia dioica*	A00RR	Stems/stalks eaten as vegetables	Spain	Spain	84.3			young shoots	green		8.93 (0.28-21.52)					91
Leek	*Allium porrum L.*	A00SB	Leeks	Spain	Spain				edible part	green						19	24
Leek	*Allium porrum L.*	A00SB	Leeks	Spain	Spain				edible part	green						5	24
Leek	*Allium porrum L.*	A00SB	Leeks	Spain	Spain				edible part							19	24
Leek	*Allium porrum L.*	A00SB	Leeks	Spain	Spain				edible part							5	24
Leek	*Allium porrum L.*	A00SB	Leeks	Spain	Spain	90			bulb	green						19±2	24
Leek	*Allium porrum L.*	A00SB	Leeks	Spain	Spain	95			bulb	green						tr.	24
Leek	*Allium porrum, L.*	A00SB	Leeks	Spain	Spain	86			bulb	green						5±0.7	24

Table S9.21.1 Bulb vegetables (A00GX) (µg/100g)

Food name	Scientific name	FoodEx2_ TermCode	FoodEx2_ TermName	Origin (country)	Purchase (country)	Water (%)	Process	Saponification	Part analysed	Colour	α-carotene	β-carotene	β-cryptoxanthin	ζ-carotene	Antheraxanthin	Ref.
Onion	*Allium cepa L.*	A00HC	Onions	India					stalks			16900				98
Onion	*Allium cepa L.*	A00HC	Onions	Germany	Germany	91.3			edible part			2				53
Onion	*Allium cepa L.*	A00HC	Onions	Spain	Spain				edible part	white		3				24
Onion	*Allium cepa L.*	A00HC	Onions	Spain	Spain				edible part	white		1				24
Onion	*Allium cepa L.*	A00HC	Onions	Spain	Spain				edible part			3				24
Onion	*Allium cepa L.*	A00HC	Onions	Spain	Spain				edible part			1				24
Onion	*Allium cepa L.*	A00HC	Onions	Spain	Spain	91			bulb without skin	white		1±0.4				24
Onion	*Allium cepa L.*	A00HC	Onions	Spain	Spain	93			bulb without skin	white		3±0.3				24
Spring onion	*Allium fistolosum L.*	A00HH	Spring onions	Spain	Spain				edible part	green		142				24
Spring onion	*Allium fistolosum L.*	A00HH	Spring onions	Spain	Spain				edible part			142				24
Spring onion	*Allium fistulosum L.*	A00HH	Spring onions	USA			raw	no			0		4			28
Spring onion	*Allium fistulosum L.*	A00HH #F28.A07GR	Spring onions, PROCESS = Frying	USA			cooked in oil	no			0		0			28

Table S9.21.2 Bulb vegetables (A00GX) (µg/100g) (continuation)

Food name	Scientific name	FoodEx2_ TermCode	FoodEx2_ TermName	Origin (country)	Purchase (country)	Water (%)	Process	Saponification	Part analysed	Colour	Capsanthin	Capsorubin	Cucurbitaxanthin	E(v. trans)-α-carotene	E(v. trans)-β-carotene	Ref.
Spring onion	*Allium fistulosum L.*	A00HH	Spring onions	USA			raw	no							370	28
Spring onion	*Allium fistulosum L.*	A00HH #F28.A07GR	Spring onions, PROCESS = Frying	USA			cooked in oil	no							0	28

Table S9.21.3 Bulb vegetables (A00GX) (µg/100g) (continuation)

Food name	Scientific name	FoodEx2_ TermCode	FoodEx2_ TermName	Origin (country)	Purchase (country)	Water (%)	Process	Saponification	Part analysed	Colour	E(v. trans)-β-cryptoxanthin	E(v. trans)-lutein	E(v. trans)-lycopene	E(v. trans)-zeaxanthin	Lactucaxanthin	Ref.
Spring onion	*Allium fistulosum L.*	A00HH	Spring onions	USA			raw	no				782		0		28
Spring onion	*Allium fistulosum L.*	A00HH #F28.A07GR	Spring onions, PROCESS = Frying	USA			cooked in oil	no						2488		28

Table S9.21.4 Bulb vegetables (A00GX) (µg/100g) (continuation)

Food name	Scientific name	FoodEx2_ TermCode	FoodEx2_ TermName	Origin (country)	Purchase (country)	Water (%)	Process	Saponification	Part analysed	Colour	Lutein	Luteoxanthin	Lycopene	Neoxanthin	Phytoene	Ref.
Onion	*Allium cepa L.*	A00HC	Onions	India	India				stalks		30280			6610		98
Onion	*Allium cepa L.*	A00HC	Onions	Spain	Spain				edible part	white	2					24

Table S9.21.4 Bulb vegetables (A00GX) (µg/100g) (continuation)

Food name	Scientific name	FoodEx2_ TermCode	FoodEx2_ TermName	Origin (country)	Purchase (country)	Water (%)	Process	Saponification	Part analysed	Colour	Lutein	Luteoxanthin	Lycopene	Neoxanthin	Phytoene	Ref.
Onion	*Allium cepa L.*	A00HC	Onions	Germany	Germany	91.3			edible part		15					53
Onion	*Allium cepa L.*	A00HC	Onions	Spain	Spain				edible part	white	5					24
Onion	*Allium cepa L.*	A00HC	Onions	Spain	Spain				edible part		5					24
Onion	*Allium cepa L.*	A00HC	Onions	Spain	Spain				edible part		2					24
Onion	*Allium cepa L.*	A00HC	Onions	Spain	Spain	91			without skin	white	2±0.5					24
Onion	*Allium cepa L.*	A00HC	Onions	Spain	Spain	93			without skin	white	5±0.5					24

Table S9.21.5 Bulb vegetables (A00GX) (µg/100g) (continuation)

Food name	Scientific name	FoodEx2_ TermCode	FoodEx2_ TermName	Origin (country)	Purchase (country)	Water (%)	Process	Saponification	Part analysed	Colour	Phytofluene	Violaxanthin	Z(v. cis)-lycopene	Z(v. cis)-β-carotene	Z(v. cis)-β-cryptoxanthin	Zeaxanthin	Ref.
Onion	*Allium cepa L.*	A00HC	Onions	India	India				stalks			1830				210	98
Onion	*Allium cepa L.*	A00HC	Onions	Germany	Germany	91.3			edible part			3					53
Onion	*Allium cepa L.*	A00HC	Onions	Spain	Spain				edible part	white						tr.	24
Onion	*Allium cepa L.*	A00HC	Onions	Spain	Spain				edible part	white						tr.	24
Onion	*Allium cepa L.*	A00HC	Onions	Spain	Spain	91			without skin	white						tr.	24
Onion	*Allium cepa L.*	A00HC	Onions	Spain	Spain	93			without skin	white						tr.	24
Spring onion	*Allium fistulosum L.*	A00HH	Spring onions	USA			raw	no						62			28
Spring onion	*Allium fistulosum L.*	A00HH #F28.A07GR	Spring onions, PROCESS = Frying	USA			cooked in oil	no						0			28

Table S9.22.1 Legumes with pod (A00PB) (µg/100g)

Food name	Scientific name	FoodEx2_ TermCode	FoodEx2_ TermName	Origin (country)	Purchase (country)	Water (%)	Process	Saponification	Part analysed	Colour	α-carotene	β-carotene	β-cryptoxanthin	ζ-carotene	Antheraxanthin	Ref.
French beans	*Phaseolus vulgaris L.*	A00PE	Slicing bean (young pods)	Italy	Italy					green		503				25
French beans	*Phaseolus vulgaris L.*	A00PG	French beans (with pods)	Brazil					beans	green	nd	163	nd			109
French beans	*Phaseolus vulgaris L.*	A00PG	French beans (with pods)	Germany	Germany	87.4			edible part		30	250	14			53
French beans	*Phaseolus vulgaris L.*	A00PG	French beans (with pods)	Spain	Spain				edible part		79	238				24
French beans	*Phaseolus vulgaris L.*	A00PG	French beans (with pods)	Spain	Spain				edible part		35	166				24
French beans	*Phaseolus vulgaris L.*	A00PE#F28.A07KQ$F28.A0BA1	Slicing bean (young pods), PROCESS = Freezing, Cooking and similar thermal preparation processes	USA			frozen+ cooked	no			0		0			28
French beans	*Phaseoulus vulgaris L. var. Savi.*	A00PG	French beans (with pods)	Spain	Spain	93			Pod+seeds	green	35±2	166±10				24
French beans	*Phaseoulus vulgaris, L.*	A00PG	French beans (with pods)	Spain	Spain				edible part	green	79	238				24
French beans	*Phaseoulus vulgaris, L.*	A00PG	French beans (with pods)	Spain	Spain				edible part	green	35	166				24
French beans	*Phaseoulus vulgaris, Savi*	A00PG	French beans (with pods)	Spain	Spain	91			Pod+seeds	green	79±12	238±15				24

Table S9.22.2 Legumes with pod (A00PB) (continuation)

Food name	Scientific name	FoodEx2_ TermCode	FoodEx2_ TermName	Origin (country)	Purchase (country)	Water (%)	Process	Saponification	Part analysed	Colour	Capsanthin	Capsorubin	Cucurbitaxanthin	E(v. trans)-α-carotene	E(v. trans)-β-carotene	Ref.
French beans	*Phaseolus vulgaris L.*	A00PE#F28.A07KQ$F28.A0BA1	Slicing bean (young pods), PROCESS = Freezing, Cooking and similar thermal preparation processes	USA			frozen+ cooked	no		green					0	28

Table S9.22.3 Legumes with pod (A00PB) (μg/100g) (continuation)

Food name	Scientific name	FoodEx2_ TermCode	FoodEx2_ TermName	Origin (country)	Purchase (country)	Water (%)	Process	Saponification	Part analysed	Colour	E(v. trans)-β-cryptoxanthin	E(v. trans)-lutein	E(v. trans)-lycopene	E(v. trans)-zeaxanthin	Lactucaxanthin	Ref.
French beans	*Phaseolus vulgaris L.*	A00PE#F28.A07KQ$F28.A0BA1	Slicing bean (young pods), PROCESS = Freezing, Cooking and similar thermal preparation processes	USA			frozen+ cooked	no				306		0		28

Table S9.22.4 Legumes with pod (A00PB) (µg/100g) (continuation)

Food name	Scientific name	FoodEx2_TermCode	FoodEx2_TermName	Origin (country)	Purchase (country)	Water (%)	Process	Saponification	Part analysed	Colour	Lutein	Luteoxanthin	Lycopene	Neoxanthin	Phytoene	Ref.
French beans	*Phaseolus vulgaris L.*	A00PE	Slicing bean (young pods)	Italy	Italy						883					25
French beans	*Phaseolus vulgaris L.*	A00PG	French beans (with pods)	Brazil	Brazil				beans	green	250		nd	tr.		109
French beans	*Phaseolus vulgaris L.*	A00PG	French beans (with pods)	Germany	Germany	87.4			edible part		760		24			53
French beans	*Phaseolus vulgaris L.*	A00PG	French beans (with pods)	Spain	Spain				edible part		487					24
French beans	*Phaseolus vulgaris L.*	A00PG	French beans (with pods)	Spain	Spain				edible part		365					24
French beans	*Phaseoulus vulgaris L. var. Savi*	A00PG	French beans (with pods)	Spain	Spain	93			pod	green	365±7					24
French beans	*Phaseoulus vulgaris L. var. Savi*	A00PG	French beans (with pods)	Spain	Spain	91			pod	green	487±5					24
French beans	*Phaseoulus vulgaris, L.*	A00PG	French beans (with pods)	Spain	Spain				edible part	green	487					24
French beans	*Phaseoulus vulgaris, L.*	A00PG	French beans (with pods)	Spain	Spain				edible part	green	365					24

Table S9.22.5 Legumes with pod (A00PB) (µg/100g) (continuation)

Food name	Scientific name	FoodEx2_TermCode	FoodEx2_TermName	Origin (country)	Purchase (country)	Water (%)	Process	Saponification	Part analysed	Colour	Phytofluene	Violaxanthin	Z(v. cis)-lycopene	Z(v. cis)-β-carotene	Z(v. cis)-β-cryptoxanthin	Zeaxanthin	Ref.
French beans	*Phaseolus vulgaris L.*	A00PG	French beans (with pods)	Brazil	Brazil				beans	green		tr.				nd	109
French beans	*Phaseolus vulgaris*	A00PG	French beans (with pods)	Germany	Germany	87.4			edible part			10					53

Table S9.22.5 Legumes with pod (A00PB) (µg/100g) (continuation)

Food name	Scientific name	FoodEx2_TermCode	FoodEx2_TermName	Origin (country)	Purchase (country)	Water (%)	Process	Saponification	Part analysed	Colour	Phytofluene	Violaxanthin	Z(v. cis)-lycopene	Z(v. cis)-β-carotene	Z(v. cis)-β-cryptoxanthin	Zeaxanthin	Ref.
French beans	*Phaseolus vulgaris L.*	A00PE#F28.A07KQ$F28.A0BA1	Slicing bean (young pods), PROCESS = Freezing, PROCESS = Cooking and similar thermal preparation processes	USA	USA		frozen+ cooked	no						0			28

Table S9.23.1 Tomatoes and similar (A00HQ) (µg/100g)

Food name	Scientific name	FoodEx2_TermCode	FoodEx2_TermName	Origin (country)	Purchase (country)	Water (%)	Process	Saponification	Part analysed	Colour	α-carotene	β-carotene	β-cryptoxanthin	ζ-carotene	Antheraxanthin	Ref.
Goji berries	*Lycium barbarum*	A0DMR	Wolfberries	Spain	Spain				edible part	red		483	1100			24
Goji berries	*Lycium barbarum*	A0DMR	Wolfberries	Spain	Spain				edible part			483	1100			24
Tomato	*Lycopersicon esculentum*	A00HY	Cherry tomatoes	Spain	Spain	92.6-96			edible part	pink-red		7300±800				124
Tomato	*Lycopersicon esculentum*	A00HY	Cherry tomatoes	Spain	Spain	92.6-96			edible part	pink-red		4800±500				124
Tomato	*Lycopersicon esculentum*	A0DMX	Tomatoes	Spain	Spain	92.6-96			edible part	pink-red		5600±700				124
Tomato	*Lycopersicon esculentum M.*	A00HX	Pear-shaped tomatoes	Spain	Spain				edible part	red		393				24
Tomato	*Lycopersicon esculentum M.*	A0DMX	Tomatoes	Portugal	Portugal				with skin and seeds	red			<6			125

Table S9.23.1 Tomatoes and similar (A00HQ) (µg/100g) (continuation)

Food name	Scientific name	FoodEx2_ TermCode	FoodEx2_ TermName	Origin (country)	Purchase (country)	Water (%)	Process	Saponification	Part analysed	Colour	α-carotene	β-carotene	β-cryptoxanthin	ζ-carotene	Antheraxanthin	Ref.
Tomato	*Lycopersicon esculentum M.*	A0DMX	Tomatoes	Spain	Spain				edible part	red		494				24
Tomato	*Lycopersicon esculentum M.*	A0DMX	Tomatoes	Spain	Spain				edible part	red		315				24
Tomato	*Lycopersicon esculentum M. var. canary*	A0DMX	Tomatoes	Spain	Spain				edible part	red		443				24
Tomato	*Lycopersicon esculentum M. var. Lido*	A0DMX	Tomatoes	Portugal	Portugal				With skin and seeds	red			<6			125
Tomato	*Lycopersicon esculentum Mill.*	A0DMX	Tomatoes	Spain	United Kingdom					-		5610±1360				95
Tomato	*Lycopersicon esculentum Mill. cv. Carmen*	A0DMX	Tomatoes	Brazil					fruit	red	nd	1150	nd			109
Tomato	*Lycopersicon esculentum Mill., cv. Durinta*	A0DMX	Tomatoes	Germany	Germany			no	fruit			8000± 200				126
Tomato	*Lycopersicon esculentum Mill., cv. Durinta*	A0DMX	Tomatoes	Germany	Germany			no	fruit			9000± 1000				126
Tomato	*Lycopersicon esculentum Mill., cv. Durinta*	A0DMX	Tomatoes	Germany	Germany			no	fruit			6000±0				126
Tomato	*Lycopersicon esculentum Mill., cv. Durinta*	A0DMX	Tomatoes	Germany	Germany			no	fruit			9000±0				126
Tomato	*Lycopersicon esculentum Mill., cv. Durinta*	A0DMX	Tomatoes	Germany	Germany			no	fruit			8000±0				126
Tomato	*Lycopersicon esculentum Mill., cv. Durinta*	A0DMX	Tomatoes	Germany	Germany			no	fruit			8000± 1000				126
Tomato	*Lycopersicon esculentum Mill., cv. Durinta*	A0DMX	Tomatoes	Germany	Germany			no	fruit			6000±0				126
Tomato	*Lycopersicon esculentum Mill., cv. Durinta*	A0DMX	Tomatoes	Germany	Germany			no	fruit			5000± 1000				126

Table S9.23.1 Tomatoes and similar (A00HQ) (μg/100g) (continuation)

Food name	Scientific name	FoodEx2_ TermCode	FoodEx2_ TermName	Origin (country)	Purchase (country)	Water (%)	Process	Saponification	Part analysed	Colour	α-carotene	β-carotene	β-cryptoxanthin	ζ-carotene	Antheraxanthin	Ref.
Tomato	*Lycopersicon esculentum Mill., cv. Durinta*	A0DMX	Tomatoes	Germany	Germany			no	fruit			6000± 2000				126
Tomato	*Lycopersicon esculentum Mill., hybrid HC01*	A0DMX	Tomatoes	Italy	Italy			no	fruit	red		310		100		127
Tomato	*Lycopersicon esculentum Mill., hybrid HC02*	A0DMX	Tomatoes	Italy	Italy			no	fruit	red		330		100		127
Tomato	*Lycopersicon esculentum Mill., hybrid HC03*	A0DMX	Tomatoes	Italy	Italy			no	fruit	red		440		110		127
Tomato	*Lycopersicon esculentum Mill., hybrid HC04*	A0DMX	Tomatoes	Italy	Italy			no	fruit	red		350		80		127
Tomato	*Lycopersicon esculentum Mill., hybrid HC05*	A0DMX	Tomatoes	Italy	Italy			no	fruit	red		310		60		127
Tomato	*Lycopersicon esculentum var. Daniela long life*	A0DMX	Tomatoes	Spain	Spain	92.6-96			edible part	pink-red		2000±400				124
Tomato	*Lycopersicon esculentum var. cerasiforme*	A00HY	Cherry tomatoes	Italy	Italy							300–1100				25
Tomato	*Lycopersicon esculentum var. Lido*	A0DMX	Tomatoes	Spain	Spain	92.6-96			edible part	pink-red		500±200				128
Tomato	*Lycopersicon esculentum var. Racimo*	A0DMX	Tomatoes	Spain	Spain	92.6-96			edible part	pink-red		400±100				128
Tomato	*Lycopersicon esculentum var. Raf*	A0DMX	Tomatoes	Spain	Spain	92.6-96			edible part	pink-red		1500±300				124
Tomato	*Lycopersicon esculentum var. Rambo*	A0DMX	Tomatoes	Spain	Spain	92.6-96			edible part	pink-red		2800±400				124
Tomato	*Lycopersicum esculentum Mill.*	A00HX	Pear-shaped tomatoes	Spain	Spain				edible part			393				24

Table S9.23.1 Tomatoes and similar (A00HQ) (µg/100g) (continuation)

Food name	Scientific name	FoodEx2_ TermCode	FoodEx2_ TermName	Origin (country)	Purchase (country)	Water (%)	Process	Saponification	Part analysed	Colour	α-carotene	β-carotene	β-cryptoxanthin	ζ-carotene	Antheraxanthin	Ref.
Tomato	*Lycopersicum esculentum mill.*	A0DMX	Tomatoes	Spain	Spain				edible part			494				24
Tomato	*Lycopersicum esculentum mill. Va. Canary*	A0DMX	Tomatoes	Spain	Spain				edible part			443				24
Tomato	*Solanum lycopersicum*	A0DMX	Tomatoes	Italy	Italy						nd	320–1500	nd			25
Tomato	*Solanum lycopersicum*	A0DMX	Tomatoes	Germany	Germany	93.8			edible part		150	890				53
Tomato	*Solanum lycopersicum*	A0DMX	Tomatoes	Germany	Germany	94.2			edible part		150	610	360			53
Tomato	*Solanum lycopersicum*	A0DMX	Tomatoes	Italy	Italy							390				53
Tomato	*Solanum lycopersicum*	A0DMX	Tomatoes	Italy	Italy							530				128
Tomato	*Solanum lycopersicum*	A0DMX	Tomatoes	Italy	Italy	na			whole fruit	red		1600-3900				129
Tomato	*Solanum lycopersicum*	A0DMX	Tomatoes	Spain	Spain	94.85			fruit	red		76				130
Tomato	*Solanum lycopersicum*	A0DMX	Tomatoes	USA				no			0		0			28
Tomato	*Solanum lycopersicum var. Heinz(H)*	A0DMX	Tomatoes	Spain	Spain	95.14			fruit	red		68				130
Tomato	*Solanum lycopersicum var. Heinz(H)*	A0DMX	Tomatoes	Spain	Spain	95.38			fruit	red		105				130
Tomato	*Solanum lycopersicum L.*	A0DMX	Tomatoes	Irland	Ireland		raw	no	fruit			342.8± 12.9				131
Tomato	*Solanum lycopersicum L.*	A0DMX	Tomatoes	Irland	Ireland		raw	no	fruit			234.5± 23.3				131
Tomato	*Solanum lycopersicum L.*	A0DMX	Tomatoes	Spain	Spain		Raw	no	fruit			350.8± 106.2				131
Tomato	*Solanum lycopersicum L.*	A0DMX	Tomatoes									215				131
Tomato	*Solanum lycopersicum L.*	A0DMX	Tomatoes									600-1000				131

Table S9.23.1 Tomatoes and similar (A00HQ) (µg/100g) (continuation)

Food name	Scientific name	FoodEx2_ TermCode	FoodEx2_ TermName	Origin (country)	Purchase (country)	Water (%)	Process	Saponification	Part analysed	Colour	α-carotene	β-carotene	β-cryptoxanthin	ζ-carotene	Antheraxanthin	Ref.
Tomato	*Solanum lycopersicum L.*	A0DMX	Tomatoes									415				131
Tomato	*Solanum lycopersicum L.*	A0DMX	Tomatoes									285-617				131
Tomato	*Solanum lycopersicum L.*	A0DMX	Tomatoes									220-750				131
Tomato	*Solanum lycopersicum L.*	A0DMX	Tomatoes									390				131
Tomato	*Solanum lycopersicum L.*	A0DMX	Tomatoes									330				131
Tomato	*Solanum lycopersicum L.*	A0DMX	Tomatoes									830-920				131
Tomato	*Solanum lycopersicum L.*	A0DMX	Tomatoes									1100				131
Tomato	*Solanum lycopersicum L.*	A0DMX	Tomatoes									494				131
Tomato	*Solanum lycopersicum L.*	A0DMX	Tomatoes									660				131
Tomato	*Solanum lycopersicum L.*	A0DMX	Tomatoes									393				131
Tomato	*Solanum lycopersicum L.*	A0DMX	Tomatoes									280				131
Tomato	*Solanum lycopersicum L.*	A0DMX	Tomatoes									778				131
Tomato	*Solanum lycopersicum L.*	A0DMX	Tomatoes									377				131
Tomato	*Solanum lycopersicum L.*	A0DMX	Tomatoes									890				131
Tomato	*Solanum lycopersicum L.*	A0DMX	Tomatoes									740				131
Tomato	*Solanum lycopersicum L.*	A0DMX	Tomatoes									320				131
Tomato	*Solanum lycopersicum L.*	A0DMX	Tomatoes									608				131
Tomato	*Solanum lycopersicum L.*	A0DMX	Tomatoes									670-890				131
Tomato	*Solanum lycopersicum L. cv Cervil*	A00HY	Cherry tomatoes	France	France			no	fruit	green		190				132

Table S9.23.1 Tomatoes and similar (A00HQ) (µg/100g) (continuation)

Food name	Scientific name	FoodEx2_ TermCode	FoodEx2_ TermName	Origin (country)	Purchase (country)	Water (%)	Process	Saponification	Part analysed	Colour	α-carotene	β-carotene	β-cryptoxanthin	ζ-carotene	Antheraxanthin	Ref.
Tomato	*Solanum lycopersicum L. cv Cervil*	A00HY	Cherry tomatoes	France	France			no	fruit			920				132
Tomato	*Solanum lycopersicum L. cv Cervil*	A00HY	Cherry tomatoes	France	France			no	fruit			810				132
Tomato	*Solanum lycopersicum L. cv Cervil*	A00HY	Cherry tomatoes	France	France			no	fruit			780				132
Tomato	*Solanum lycopersicum L. cv Cervil*	A00HY	Cherry tomatoes	France	France			no	fruit			610				132
Tomato	*Solanum lycopersicum L. cv Cervil*	A00HY	Cherry tomatoes	France	France			no	fruit			170				132
Tomato	*Solanum lycopersicum L. cv Cervil*	A00HY	Cherry tomatoes	France	France			no	fruit			920				132
Tomato	*Solanum lycopersicum L. cv Cervil*	A00HY	Cherry tomatoes	France	France			no	fruit			710				132
Tomato	*Solanum lycopersicum L. cv Cervil*	A00HY	Cherry tomatoes	France	France			no	fruit			1020				132
Tomato	*Solanum lycopersicum L. cv Cervil*	A00HY	Cherry tomatoes	France	France			no	fruit			680				132
Tomato	*Solanum lycopersicum L. cv Cervil*	A00HY	Cherry tomatoes	France	France				fruit	red		1610± 110				133
Tomato	*Solanum lycopersicum L. cv Cervil*	A00HY	Cherry tomatoes	France	France				fruit	red		1480±0				133
Tomato	*Solanum lycopersicum L. cv Cervil*	A00HY	Cherry tomatoes	France	France				fruit	red		1610±80				133
Tomato	*Solanum lycopersicum L. cv Cervil*	A00HY	Cherry tomatoes	France	France				fruit	red		1540±70				133
Tomato	*Solanum lycopersicum L. cv Cervil*	A00HY	Cherry tomatoes	France	France				fruit	red		1380± 130				133
Tomato	*Solanum lycopersicum L. cv Cervil*	A00HY	Cherry tomatoes	France	France				fruit	red		1140±30				133

Table S9.23.1 Tomatoes and similar (A00HQ) (µg/100g) (continuation)

Food name	Scientific name	FoodEx2_ TermCode	FoodEx2_ TermName	Origin (country)	Purchase (country)	Water (%)	Process	Saponification	Part analysed	Colour	α-carotene	β-carotene	β-cryptoxanthin	ζ-carotene	Antheraxanthin	Ref.
Tomato	*Solanum lycopersicum L. cv. Ailsa Craig (Ac)*	A0DMX	Tomatoes	Italy	Italy				fruit	red		14980				134
Tomato	*Solanum lycopersicum L. cv. Ailsa Craig (Ac)*	A0DMX	Tomatoes	Italy	Italy				fruit	red		21120				134
Tomato	*Solanum lycopersicum L. cv. Ailsa Craig (Ac)*	A0DMX	Tomatoes	Italy	Italy				fruit	red		7030				134
Tomato	*Solanum lycopersicum L. cv. Ailsa Craig (Ac)*	A0DMX	Tomatoes	Italy	Italy				fruit	red		8320				134
Tomato	*Solanum lycopersicum L. cv. Ailsa Craig (Ac)*	A0DMX	Tomatoes	Italy	Italy				fruit	red		7430				134
Tomato	*Solanum lycopersicum L. cv. Ailsa Craig (Ac)*	A0DMX	Tomatoes	Italy	Italy				fruit	red		11360				134
Tomato	*Solanum lycopersicum L. cv. Ailsa Craig (Ac)*	A0DMX	Tomatoes	Italy	Italy				fruit	strong purple		7080				134
Tomato	*Solanum lycopersicum L. cv. Ailsa Craig (Ac)*	A0DMX	Tomatoes	Italy	Italy				fruit	strong purple		8750				134
Tomato	*Solanum lycopersicum L.*	A0DMX	Tomatoes	Spain	Spain	na			fruit	red	59-137	2530-1280				135
Tomato	*Solanum lycopersicum L.*	A00HY	Cherry tomatoes	Irland	Ireland		raw	no	fruit			489±43.7				131
Tomato	*Solanum lycopersicum L.*	A00HT	Plum tomato	Irland	Ireland		raw	no	fruit			441.1± 68.3				131
Tomato	*Solanum lycopersicum L.*	A00HY	Cherry tomatoes	Spain	Spain		Raw	no	fruit			462.3± 93.4				131

Table S9.23.1 Tomatoes and similar (A00HQ) (µg/100g) (continuation)

Food name	Scientific name	FoodEx2_ TermCode	FoodEx2_ TermName	Origin (country)	Purchase (country)	Water (%)	Process	Saponification	Part analysed	Colour	α-carotene	β-carotene	β-cryptoxanthin	ζ-carotene	Antheraxanthin	Ref.
Tomato	*Solanum lycopersicum L.*	A00HY	Cherry tomatoes	Spain	Spain		Raw	no	fruit			583,5± 89.2				131
Tomato	*Solanum lycopersicum L.*	A00HT	Plum tomato	Spain	Spain		Raw	no	fruit			361.6± 109.5				131
Tomato	*Solanum lycopersicum L.*	A00HY	Cherry tomatoes									3100				131
Tomato	*Solanum lycopersicum L.*	A00HY	Cherry tomatoes									700-1200				131
Tomato	*Solanum lycopersicum L.*	A00HY	Cherry tomatoes									473				131
Tomato	*Solanum lycopersicum L.*	A00HY	Cherry tomatoes									7300				131
Tomato	*Solanum lycopersicum L.*	A00HY	Cherry tomatoes									500-1100				131
Tomato	*Solanum lycopersicum L.*	A00HY	Cherry tomatoes									519-1,063				131
Tomato	*Solanum lycopersicum L.*	A00HY	Cherry tomatoes									990				131
Tomato	*Solanum lycopersicum L.*	A00HY	Cherry tomatoes									300				131
Tomato	*Solanum lycopersicum L.*	A00HY	Cherry tomatoes									920-1050				131
Tomato	*Solanum lycopersicum L.*	A00HX	Pear-shaped tomatoes	Spain	Spain	94			fruit	red						24
Tomato	*Solanum lycopersicum L.*	A0DMX	Tomatoes	Spain	Spain				edible part	red		186				24
Tomato	*Solanum lycopersicum L.*	A0DMX	Tomatoes	Spain	Spain	94			fruit	red		494±124				24
Tomato	*Solanum lycopersicum L.*	A00HX	Pear-shaped tomatoes	Spain	Spain	94			fruit	red		393±39				24

Table S9.23.1 Tomatoes and similar (A00HQ) (µg/100g) (continuation)

Food name	Scientific name	FoodEx2_ TermCode	FoodEx2_ TermName	Origin (country)	Purchase (country)	Water (%)	Process	Saponification	Part analysed	Colour	α-carotene	β-carotene	β-cryptoxanthin	ζ-carotene	Antheraxanthin	Ref.
Tomato	*Solanum lycopersicum L.*	A0DMX	Tomatoes	Spain	Spain	81			fruit	red		315±67				24
Tomato	*Solanum lycopersicum L. Var. Canary*	A0DMX	Tomatoes	Spain	Spain	94			fruit	red		443±37				24
Tomato	*Solanum lycopersicum var. Diamond*	A0DMX	Tomatoes	Spain	Spain	95,48			fruit	red		200				131
Tomato	*Solanum lycopersicum var. Heinz(H)*	A0DMX	Tomatoes	Spain	Spain	95.2			fruit	red		127				131
Tomato	*Solanum lycopersicum var. Kalvert*	A0DMX	Tomatoes	Spain	Spain	95,15			fruit	red		227				131
Tomato	*Solanum lycopersicum L.*	A0DMX	Tomatoes	United Kingdom	Unite Kingdom				fruit			430-1700				86
Tomato	*Solanum lycopersicum L.*	A0DMX	Tomatoes	USA					fruit			280±20				86
Tomato	*Solanum lycopersicum L.*	A0DMX	Tomatoes	Malaysia					fruit			360				86

Table S9.23.2 Tomatoes and similar (A00HQ) (µg/100g) (continuation)

Food name	Scientific name	FoodEx2_ TermCode	FoodEx2_ TermName	Origin (country)	Purchase (country)	Water (%)	Process	Saponification	Part analysed	Colour	Capsanthin	Capsorubin	Cucurbitaxanthin	E(v. trans)-α-carotene	E(v. trans)-β-carotene	Ref.
Tomato		A0DMX#F20.A0F7P$F20.A07RC	Tomatoes, PART-CONSUMED-ANALYSED = Only peel, With seeds	Greece	Greece	7.65 ± 0.21			only skin and seeds	red					48.48±0.92	136

Table S9.23.2 Tomatoes and similar (A00HQ) (µg/100g) (continuation)

Food name	Scientific name	FoodEx2_ TermCode	FoodEx2_ TermName	Origin (country)	Purchase (country)	Water (%)	Process	Saponification	Part analysed	Colour	Capsanthin	Capsorubin	Cucurbitaxanthin	E(v. trans)-α-carotene	E(v. trans)-β-carotene	Ref.
Tomato	Lycopersicon esculentum M.	A0DMX	Tomatoes	Portugal	Portugal				edible part	red					170±100	57
Tomato	Lycopersicon esculentum M.	A0DMX	Tomatoes	Portugal	Portugal				edible part	red					513±75	57
Tomato	Lycopersicon esculentum M.	A0DMX	Tomatoes	Portugal	Portugal				edible part	red					255±29	57
Tomato	Lycopersicon esculentum M. var. "for salad"	A0DMX	Tomatoes	Portugal	Portugal				with skin and seeds	red				<8	390±56	125
Tomato	Lycopersicon esculentum M. var. Lido	A0DMX	Tomatoes	Portugal	Portugal				with skin and seeds	red				<8	1000 (210)±140	125
Tomato	Solanum lycopersicum	A0DMX	Tomatoes	USA				no							0	28
Tomato	Solanum lycopersicum L. cv. Velasco	A0DMX	Tomatoes	Spain	Spain				fruit	red					2570	137

Table S9.23.3 Tomatoes and similar (A00HQ) (µg/100g) (continuation)

Food name	Scientific name	FoodEx2_ TermCode	FoodEx2_ TermName	Origin (country)	Purchase (country)	Water (%)	Process	Saponification	Part analysed	Colour	E(v. trans)-β-cryptoxanthin	E(v. trans)-lutein	E(v. trans)-lycopene	E(v. trans)-zeaxanthin	Lactucaxanthin	Ref.
Tomato		A0DMX#F20.A0F7P$F20.A07RC	Tomatoes, PART-CONSUMED-ANALYSED = Only peel, PART-CONSUMED-ANALYSED = With seeds	Greece	Greece	7.65 ± 0.21			only skin and seeds	red		39.14± 0.21	64.84± 0.87			136

Table S9.23.3 Tomatoes and similar (A00HQ) (µg/100g) (continuation)

Food name	Scientific name	FoodEx2_ TermCode	FoodEx2_ TermName	Origin (country)	Purchase (country)	Water (%)	Process	Saponification	Part analysed	Colour	E(v. trans)-β-cryptoxanthin	E(v. trans)-lutein	E(v. trans)-lycopene	E(v. trans)-zeaxanthin	Lactucaxanthin	Ref.
Tomato	*Lycopersicon esculentum M.*	A0DMX	Tomatoes	Portugal	Portugal				edible part	red		102.0± 7.3	6700± 1000			57
Tomato	*Lycopersicon esculentum M.*	A0DMX	Tomatoes	Portugal	Portugal				edible part	red			8340± 870			57
Tomato	*Lycopersicon esculentum M.*	A0DMX	Tomatoes	Portugal	Portugal				edible part	red		77.1±6.3	9050± 900			57
Tomato	*Lycopersicon esculentum M.*	A00HY	Cherry tomatoes	Spain	Spain	92.6-96			edible part	pink-red		800±200				124
Tomato	*Lycopersicon esculentum M.*	A00HY	Cherry tomatoes	Spain	Spain	92.6-96			edible part	pink-red		1900± 400				124
Tomato	*Lycopersicon esculentum M.*	A00HX	Pear-shaped tomatoes	Spain	Spain	92.6-96			edible part	pink-red		800±300				124
Tomato	*Lycopersicon esculentum M. var. Racimo*	A0DMX	Tomatoes	Spain	Spain	92.6-96			edible part	pink-red		300±100				124
Tomato	*Lycopersicon esculentum M. var. Rambo*	A0DMX	Tomatoes	Spain	Spain	92.6-96			edible part	pink-red		800±200				124
Tomato	*Lycopersicon esculentum M. var. Daniela long life*	A0DMX	Tomatoes	Spain	Spain	92.6-96			edible part	pink-red		700±200				124
Tomato	*Lycopersicon esculentum M. var. Lido*	A0DMX	Tomatoes	Spain	Spain	92.6-96			edible part	pink-red		300±200				124
Tomato	*Lycopersicon esculentum M. var. Raf*	A0DMX	Tomatoes	Spain	Spain	92.6-96			edible part	pink-red		1000± 300				124
Tomato	*Solanum lycopersicum L.*	A0DMX#F20. A0F7Q	Tomatoes, PART-CONSUMED-ANALYSED = Only skin	Finland	Finland				skin	-			23435			26

Table S9.23.3 Tomatoes and similar (A00HQ) (µg/100g) (continuation)

Food name	Scientific name	FoodEx2_ TermCode	FoodEx2_ TermName	Origin (country)	Purchase (country)	Water (%)	Process	Saponification	Part analysed	Colour	E(v. trans)-β-cryptoxanthin	E(v. trans)-lutein	E(v. trans)-lycopene	E(v. trans)-zeaxanthin	Lactucaxanthin	Ref.
Tomato	*Solanum lycopersicum L.*	A0DMX	Tomatoes	USA	USA			no	raw			32		0		28
Tomato	*Solanum lycopersicum L. cv. Velasco*	A0DMX	Tomatoes	Spain	Spain				fruit	red			10040			137

Table S9.23.4 Tomatoes and similar (A00HQ) (µg/100g) (continuation)

Food name	Scientific name	FoodEx2_ TermCode	FoodEx2_ TermName	Origin (country)	Purchase (country)	Water (%)	Process	Saponification	Part analysed	Colour	Lutein	Luteoxanthin	Lycopene	Neoxanthin	Phytoene	Ref.
Goji berries	*Lycium barbarum*	A0DMR	Wolfberries	Spain	Spain				edible part	red	311					24
Goji berries	*Lycium barbarum*	A0DMR	Wolfberries	Spain	Spain				edible part		311					24
Tomato		A0DMX#F20. A0F7P$F20.A07RC	Tomatoes, PART-CONSUMED-ANALYSED = Only peel, PART-CONSUMED-ANALYSED = With seeds	Greece	Greece	7.65 ± 0.21			only Skin and seeds	red	99.42 (Sum of all trans-, 9-cis and 13-cis-lutein)					136
Tomato	*Lycopersicon esculentum M.*	A00HX	Pear-shaped tomatoes	Spain	Spain				edible part	red	72		62273			24
Tomato	*Lycopersicon esculentum M.*	A00HY	Cherry tomatoes	Spain	Spain	92.6-96			edible part	pink-red			35000± 3400			124
Tomato	*Lycopersicon esculentum M.*	A00HY	Cherry tomatoes	Spain	Spain	92.6-96			edible part	pink-red			2800±400			124
Tomato	*Lycopersicon esculentum M.*	A00HX	Pear-shaped tomatoes	Spain	Spain	92.6-96			edible part	pink-red			12000± 1200			124
Tomato	*Lycopersicon esculentum M.*	A0DMX	Tomatoes	Spain	Spain				edible part	red	44		1604			24

Table S9.23.4 Tomatoes and similar (A00HQ) (µg/100g) (continuation)

Food name	Scientific name	FoodEx2_ TermCode	FoodEx2_ TermName	Origin (country)	Purchase (country)	Water (%)	Process	Saponification	Part analysed	Colour	Lutein	Luteoxanthin	Lycopene	Neoxanthin	Phytoene	Ref.
Tomato	*Lycopersicon esculentum M.*	A0DMX	Tomatoes	Spain	Spain				edible part	red	52		2116			24
Tomato	*Lycopersicon esculentum M.*	A0DMX	Tomatoes	Spain	Spain				edible part	red	114		14571			24
Tomato	*Lycopersicon esculentum M. var Lido*	A0DMX	Tomatoes	Spain	Spain	92.6-96			edible part	pink-red			29100± 2600			124
Tomato	*Lycopersicon esculentum M. var. "for salad"*	A0DMX	Tomatoes	Portugal	Portugal				with skin +seeds	red	80±15		2300± 570			125
Tomato	*Lycopersicon esculentum M. var. Daniela long life*	A0DMX	Tomatoes	Spain	Spain	92.6-96			edible part	pink-red			12900± 1100			124
Tomato	*Lycopersicon esculentum M. var. Lido*	A0DMX	Tomatoes	Portugal	Portugal				edible part	red	100 (49)±17		8000 17000± 2000			125
Tomato	*Lycopersicon esculentum M. var. Racimo*	A0DMX	Tomatoes	Spain	Spain	92.6-96			edible part	pink-red			23400± 2400			124
Tomato	*Lycopersicon esculentum M. var. Raf*	A0DMX	Tomatoes	Spain	Spain	92.6-96			edible part	pink-red			39700± 4300			124
Tomato	*Lycopersicon esculentum M. var. Rambo*	A0DMX	Tomatoes	Spain	Spain	92.6-96			edible part	pink-red			9700± 1100			124
Tomato	*Lycopersicon esculentum Mill*	A0DMX	Tomatoes	Italy	Italy			no	fruit	red			5890		630	127
Tomato	*Lycopersicon esculentum Mill*	A0DMX	Tomatoes	Italy	Italy			no	fruit	red			8310		720	127
Tomato	*Lycopersicon esculentum Mill*	A0DMX	Tomatoes	Italy	Italy			no	fruit	red			10220		860	127
Tomato	*Lycopersicon esculentum Mill*	A0DMX	Tomatoes	Italy	Italy			no	fruit	red			7880		740	127
Tomato	*Lycopersicon esculentum Mill*	A0DMX	Tomatoes	Italy	Italy			no	fruit	red			6710		660	127
Tomato	*Lycopersicon esculentum Mill.*	A0DMX	Tomatoes	Spain	United Kingdom				fruit (whole)	-	9490± 1940		52250± 9260		23420±39 10	95
Tomato	*Lycopersicon esculentum Mill. cv. Carmen*	A0DMX	Tomatoes	Brazil					fruit	red	100		3540	nd		109

Table S9.23.4 Tomatoes and similar (A00HQ) (µg/100g) (continuation)

Food name	Scientific name	FoodEx2_ TermCode	FoodEx2_ TermName	Origin (country)	Purchase (country)	Water (%)	Process	Saponification	Part analysed	Colour	Lutein	Luteoxanthin	Lycopene	Neoxanthin	Phytoene	Ref.
Tomato	*Lycopersicon esculentum Mill., cv. Durinta*	A0DMX	Tomatoes	Germany	Germany			no	fruit				101000± 17000			126
Tomato	*Lycopersicon esculentum Mill., cv. Durinta*	A0DMX	Tomatoes	Germany	Germany			no	fruit				96000± 11000			126
Tomato	*Lycopersicon esculentum Mill., cv. Durinta*	A0DMX	Tomatoes	Germany	Germany			no	fruit				95000± 20000			126
Tomato	*Lycopersicon esculentum Mill., cv. Durinta*	A0DMX	Tomatoes	Germany	Germany			no	fruit				91000± 3000			126
Tomato	*Lycopersicon esculentum Mill., cv. Durinta*	A0DMX	Tomatoes	Germany	Germany			no	fruit				99000± 19000			126
Tomato	*Lycopersicon esculentum Mill., cv. Durinta*	A0DMX	Tomatoes	Germany	Germany			no	fruit				87000± 3000			126
Tomato	*Lycopersicon esculentum Mill., cv. Durinta*	A0DMX	Tomatoes	Germany	Germany			no	fruit				80000± 11000			126
Tomato	*Lycopersicon esculentum Mill., cv. Durinta*	A0DMX	Tomatoes	Germany	Germany			no	fruit				75000± 16000			126
Tomato	*Lycopersicon esculentum Mill., cv. Durinta*	A0DMX	Tomatoes	Germany	Germany			no	fruit				82000± 8000			126
Tomato	*Lycopersicon esculentum var. cerasiforme*	A00HY	Cherry tomatoes	Italy	Italy						nd – 25		800– 12000			25
Tomato	*Lycopersicum esculentum M.*	A0DMX	Tomatoes	Spain	Spain				edible part		44		1604			24
Tomato	*Lycopersicum esculentum M.*	A0DMX	Tomatoes	Spain	Spain				edible part		52		2116			24
Tomato	*Lycopersicum esculentum M. var. Heinz*	A0DMX	Tomatoes	Spain	Spain			no	without skin and seeds	red			116300± 4440			138
Tomato	*Lycopersicum esculentum M. , var. Heinz*	A0DMX	Tomatoes	Spain	Spain		Raw	no	without skin and seeds	red			130000± 9420			138

Table S9.23.4 Tomatoes and similar (A00HQ) (µg/100g) (continuation)

Food name	Scientific name	FoodEx2_ TermCode	FoodEx2_ TermName	Origin (country)	Purchase (country)	Water (%)	Process	Saponification	Part analysed	Colour	Lutein	Luteoxanthin	Lycopene	Neoxanthin	Phytoene	Ref.
Tomato	*Lycopersicum esculentum M. var. Heinz*	A0DMX	Tomatoes	Spain	Spain			no	without skin and seeds	red			121700± 14000			138
Tomato	*Lycopersicum esculentum M. var. Heinz*	A0DMX	Tomatoes	Spain	Spain			no	without skin and seeds	red			121500± 6660			138
Tomato	*Lycopersicum esculentum M. var. Heinz*	A0DMX	Tomatoes	Spain	Spain			no	without skin and seeds	red			115100± 1130			138
Tomato	*Lycopersicum esculentum M. var. Heinz*	A0DMX	Tomatoes	Spain	Spain			no	without skin and seeds	red			122200± 7730			138
Tomato	*Lycopersicum esculentum M. var. Heinz*	A0DMX	Tomatoes	Spain	Spain			no	without skin and seeds	red			94770± 6330			138
Tomato	*Lycopersicum esculentum M. var. Heinz*	A0DMX	Tomatoes	Spain	Spain			no	without skin and seeds	red			95630± 7270			138
Tomato	*Solanum lycopersicum*	A0DMX	Tomatoes	Italy	Italy						46–213		850–12 700			25
Tomato	*Solanum lycopersicum*	A0DMX	Tomatoes	Germany	Germany	93.8			edible part		210		11440			53
Tomato	*Solanum lycopersicum*	A0DMX	Tomatoes	Germany	Germany	94.2			edible part		90		302			53
Tomato	*Solanum lycopersicum*	A0DMX	Tomatoes	Greece	Greece	na			Whole plant	red			6083-6214			139
Tomato	*Solanum lycopersicum*	A0DMX	Tomatoes	USA			raw	no								28
Tomato	*Solanum lycopersicum L.*	A00HX	Pear-shaped tomatoes	Spain	Spain	94			Fruit	red					2795±446	24

Table S9.23.4 Tomatoes and similar (A00HQ) (µg/100g) (continuation)

Food name	Scientific name	FoodEx2_ TermCode	FoodEx2_ TermName	Origin (country)	Purchase (country)	Water (%)	Process	Saponification	Part analysed	Colour	Lutein	Luteoxanthin	Lycopene	Neoxanthin	Phytoene	Ref.
Tomato	*Solanum lycopersicum L.*	A0DMX#F20. A07QF$F20.A 07RD	Tomatoes, PART-CONSUMED-ANALYSED = W/o peel, PART-CONSUMED-ANALYSED = W/o seeds	Spain	Spain				peel, jelly and seeds were removed	L* 48.40±7. 19, a* 29.51±7. 74, b* 28.55±4. 04			0.08-1.42			140
Tomato	*Solanum lycopersicum L.*	A0DMX	Tomatoes	Spain	Spain				edible part	red			9708			24
Tomato	*Solanum lycopersicum L.*	A0DMX	Tomatoes	Italy	Italy				fruit				7620			128
Tomato	*Solanum lycopersicum L.*	A0DMX	Tomatoes	Italy	Italy				fruit				14130			128
Tomato	*Solanum lycopersicum L.*	A0DMX	Tomatoes	Hungary	Hungary				all sample				10933± 1450			61
Tomato	*Solanum lycopersicum L.*	A0DMX	Tomatoes	Hungary	Hungary				all sample				11233± 1000			61
Tomato	*Solanum lycopersicum L.*	A0DMX	Tomatoes	Hungary	Hungary				all sample				7700± 431			61
Tomato	*Solanum lycopersicum L.*	A0DMX	Tomatoes	Hungary	Hungary				all sample				9250±850			61
Tomato	*Solanum lycopersicum L.*	A0DMX	Tomatoes	Hungary	Hungary				all sample				9250±450			61
Tomato	*Solanum lycopersicum L.*	A0DMX	Tomatoes	Hungary	Hungary				all sample				8767±733			61
Tomato	*Solanum lycopersicum L.*	A0DMX	Tomatoes	Spain	Spain				pulp	red					1000 (600-1380)	22
Tomato	*Solanum lycopersicum L.*	A0DMX	Tomatoes	Spain	Spain				fruit	red					1860	22
Tomato	*Solanum lycopersicum L.*	A0DMX	Tomatoes	Spain	Spain				fruit	red					4940 (1520-8360)	22
Tomato	*Solanum lycopersicum L.*	A0DMX#F20. A0F7Q	Tomatoes, PART-CONSUMED-ANALYSED = Only skin	Finland	Finland				skin	-						26
Tomato	*Solanum lycopersicum L.*	A0DMX	Tomatoes	Spain	Spain				fruit	red	108-294					135

Table S9.23.4 Tomatoes and similar (A00HQ) (µg/100g) (continuation)

Food name	Scientific name	FoodEx2_ TermCode	FoodEx2_ TermName	Origin (country)	Purchase (country)	Water (%)	Process	Saponification	Part analysed	Colour	Lutein	Luteoxanthin	Lycopene	Neoxanthin	Phytoene	Ref.
Tomato	*Solanum lycopersicum L.*	A0DMX	Tomatoes	Italy	Italy				whole fruit	red			15200-37300			129
Tomato	*Solanum lycopersicum L.*	A0DMX	Tomatoes	Spain	Spain	94			fruit	red	52±12		2116± 583			54
Tomato	*Solanum lycopersicum L.*	A0DMX	Tomatoes	Spain	Spain	94			fruit	red	44±1		1604± 283			54
Tomato	*Solanum lycopersicum L.*	A00HX	Pear-shaped tomatoes	Spain	Spain	94			fruit	red	72±7		62273± 7944			54
Tomato	*Solanum lycopersicum L.*	A0DMX	Tomatoes	Spain	Spain	81			fruit	red	114±7		14571± 3437			54
Tomato	*Solanum lycopersicum L.*	A0DMX	Tomatoes	Spain	Spain	94			fruit	red					923±424	54
Tomato	*Solanum lycopersicum L.*	A0DMX	Tomatoes	Spain	Spain	94			fruit	red					489±68	54
Tomato	*Solanum lycopersicum L.*	A00HY	Cherry tomatoes	Irland	Ireland		raw	no	fruit		31.8±3.8		2304.4 ±258.2			131
Tomato	*Solanum lycopersicum L.*	A00HT	Plum tomato	Irland	Ireland		raw	no	fruit		16.5±1.3		2589± 150.7			131
Tomato	*Solanum lycopersicum L.*	A00HY	Cherry tomatoes	Spain	Spain		Raw	no	fruit		183.4± 18.2		1808.6± 119.7			131
Tomato	*Solanum lycopersicum L.*	A00HY	Cherry tomatoes	Spain	Spain		Raw	no	fruit		80.8±15		4194.2± 502.7			131
Tomato	*Solanum lycopersicum L.*	A00HT	Plum tomato	Spain	Spain		Raw	no	fruit		109.8± 31.8		7959.9± 347.7			131
Tomato	*Solanum lycopersicum L.*	A00HY	Cherry tomatoes										8130			131
Tomato	*Solanum lycopersicum L.*	A00HY	Cherry tomatoes										3400-7100			131
Tomato	*Solanum lycopersicum L.*	A00HY	Cherry tomatoes								101		3780			131
Tomato	*Solanum lycopersicum L.*	A00HY	Cherry tomatoes								800		35000			131
Tomato	*Solanum lycopersicum L.*	A00HY	Cherry tomatoes										4300-12000			131
Tomato	*Solanum lycopersicum L.*	A00HY	Cherry tomatoes								nd-25		7061-11969			131
Tomato	*Solanum lycopersicum L.*	A00HY	Cherry tomatoes										3430			131

Table S9.23.4 Tomatoes and similar (A00HQ) (µg/100g) (continuation)

Food name	Scientific name	FoodEx2_ TermCode	FoodEx2_ TermName	Origin (country)	Purchase (country)	Water (%)	Process	Saponification	Part analysed	Colour	Lutein	Luteoxanthin	Lycopene	Neoxanthin	Phytoene	Ref.
Tomato	*Solanum lycopersicum L.*	A00HY	Cherry tomatoes										800			131
Tomato	*Solanum lycopersicum L.*	A00HY	Cherry tomatoes										7200-10800			131
Tomato	*Solanum lycopersicum L.*	A0DMX	Tomatoes	Irland	Ireland		raw	no	fruit		29.2±2.4		1471.1± 146.1			131
Tomato	*Solanum lycopersicum L.*	A0DMX	Tomatoes	Irland	Ireland		raw	no	fruit		44.9±2.3		3169.4± 509			131
Tomato	*Solanum lycopersicum L.*	A0DMX	Tomatoes	Spain	Spain		raw	no	fruit		99.9±19.6		2615.6± 328.3			131
Tomato	*Solanum lycopersicum L.*	A0DMX	Tomatoes										12540			131
Tomato	*Solanum lycopersicum L.*	A0DMX	Tomatoes										9941			131
Tomato	*Solanum lycopersicum L.*	A0DMX	Tomatoes								83		6441			131
Tomato	*Solanum lycopersicum L.*	A0DMX	Tomatoes										3100-4500			131
Tomato	*Solanum lycopersicum L.*	A0DMX	Tomatoes								78		2937			131
Tomato	*Solanum lycopersicum L.*	A0DMX	Tomatoes										4900-8000			131
Tomato	*Solanum lycopersicum L.*	A0DMX	Tomatoes								77-338		5182-8474			131
Tomato	*Solanum lycopersicum L.*	A0DMX	Tomatoes										4050-8300			131
Tomato	*Solanum lycopersicum L.*	A0DMX	Tomatoes										3030			131
Tomato	*Solanum lycopersicum L.*	A0DMX	Tomatoes										5550			131
Tomato	*Solanum lycopersicum L.*	A0DMX	Tomatoes										3200-3800			131
Tomato	*Solanum lycopersicum L.*	A0DMX	Tomatoes										1900			131
Tomato	*Solanum lycopersicum L.*	A0DMX	Tomatoes								52		2116			131
Tomato	*Solanum lycopersicum L.*	A0DMX	Tomatoes								100		3100			131

Table S9.23.4 Tomatoes and similar (A00HQ) (µg/100g) (continuation)

Food name	Scientific name	FoodEx2_ TermCode	FoodEx2_ TermName	Origin (country)	Purchase (country)	Water (%)	Process	Saponification	Part analysed	Colour	Lutein	Luteoxanthin	Lycopene	Neoxanthin	Phytoene	Ref.
Tomato	*Solanum lycopersicum L.*	A0DMX	Tomatoes								130		3025			131
Tomato	*Solanum lycopersicum L.*	A0DMX	Tomatoes										1740			131
Tomato	*Solanum lycopersicum L.*	A0DMX	Tomatoes								130		3920			131
Tomato	*Solanum lycopersicum L.*	A0DMX	Tomatoes										19590			131
Tomato	*Solanum lycopersicum L.*	A0DMX	Tomatoes								50		2730			131
Tomato	*Solanum lycopersicum L.*	A0DMX	Tomatoes								210		11440			131
Tomato	*Solanum lycopersicum L.*	A0DMX	Tomatoes								210		4440			131
Tomato	*Solanum lycopersicum L.*	A0DMX	Tomatoes								100		3540			131
Tomato	*Solanum lycopersicum L.*	A0DMX	Tomatoes								77		2718			131
Tomato	*Solanum lycopersicum L.*	A0DMX	Tomatoes										3250			131
Tomato	*Solanum lycopersicum L.*	A0DMX	Tomatoes										3980-5220			131
Tomato	*Solanum lycopersicum L. cv Cervil*	A00HY	Cherry tomatoes	France	France			no	fruit	green			50		90	132
Tomato	*Solanum lycopersicum L. cv Cervil*	A00HY	Cherry tomatoes	France	France			no	fruit				4480		1810	132
Tomato	*Solanum lycopersicum L. cv Cervil*	A00HY	Cherry tomatoes	France	France			no	fruit				2970		1080	132
Tomato	*Solanum lycopersicum L. cv Cervil*	A00HY	Cherry tomatoes	France	France			no	fruit				4130		1180	132
Tomato	*Solanum lycopersicum L. cv Cervil*	A00HY	Cherry tomatoes	France	France			no	fruit				3510		810	132
Tomato	*Solanum lycopersicum L. cv Cervil*	A00HY	Cherry tomatoes	France	France			no	fruit				40		30	132

Table S9.23.4 Tomatoes and similar (A00HQ) (µg/100g) (continuation)

Food name	Scientific name	FoodEx2_ TermCode	FoodEx2_ TermName	Origin (country)	Purchase (country)	Water (%)	Process	Saponification	Part analysed	Colour	Lutein	Luteoxanthin	Lycopene	Neoxanthin	Phytoene	Ref.
Tomato	*Solanum lycopersicum L. cv Cervil*	A00HY	Cherry tomatoes	France	France			no	fruit				3570		1440	132
Tomato	*Solanum lycopersicum L. cv Cervil*	A00HY	Cherry tomatoes	France	France			no	fruit				2200		1080	132
Tomato	*Solanum lycopersicum L. cv Cervil*	A00HY	Cherry tomatoes	France	France			no	fruit				3030		1020	132
Tomato	*Solanum lycopersicum L. cv Cervil*	A00HY	Cherry tomatoes	France	France			no	fruit				1700		830	132
Tomato	*Solanum lycopersicum L. cv Cervil*	A00HY	Cherry tomatoes	France	France				fruit	red			5200± 610			133
Tomato	*Solanum lycopersicum L. cv Cervil*	A00HY	Cherry tomatoes	France	France				fruit	red			5630± 550			133
Tomato	*Solanum lycopersicum L. cv Cervil*	A00HY	Cherry tomatoes	France	France				fruit	red			3980± 480			133
Tomato	*Solanum lycopersicum L. cv Cervil*	A00HY	Cherry tomatoes	France	France				fruit	red			4740± 550			133
Tomato	*Solanum lycopersicum L. cv Cervil*	A00HY	Cherry tomatoes	France	France				fruit	red			4470± 490			133
Tomato	*Solanum lycopersicum L. cv Cervil*	A00HY	Cherry tomatoes	France	France				fruit	red			4000± 670			133
Tomato	*Solanum lycopersicum L. cv. Ailsa Craig (Ac)*	A0DMX	Tomatoes	Italy	Italy				fruit	red	780		83350			134
Tomato	*Solanum lycopersicum L. cv. Ailsa Craig (Ac)*	A0DMX	Tomatoes	Italy	Italy				fruit	strong purple	310		32580			134
Tomato	*Solanum lycopersicum L. cv. Ailsa Craig (Ac)*	A0DMX	Tomatoes	Italy	Italy				fruit	red	540		32270			134
Tomato	*Solanum lycopersicum L. cv. Ailsa Craig (Ac)*	A0DMX	Tomatoes	Italy	Italy				fruit	red	790		60050			134

Table S9.23.4 Tomatoes and similar (A00HQ) (µg/100g) (continuation)

Food name	Scientific name	FoodEx2_ TermCode	FoodEx2_ TermName	Origin (country)	Purchase (country)	Water (%)	Process	Saponification	Part analysed	Colour	Lutein	Luteoxanthin	Lycopene	Neoxanthin	Phytoene	Ref.
Tomato	*Solanum lycopersicum L. cv. Ailsa Craig (Ac)*	A0DMX	Tomatoes	Italy	Italy				fruit	red	400		25260			134
Tomato	*Solanum lycopersicum L. cv. Ailsa Craig (Ac)*	A0DMX	Tomatoes	Italy	Italy				fruit	red	520		74780			134
Tomato	*Solanum lycopersicum L. cv. Ailsa Craig (Ac)*	A0DMX	Tomatoes	Italy	Italy				fruit	red	760		74870			134
Tomato	*Solanum lycopersicum L. cv. Ailsa Craig (Ac)*	A0DMX	Tomatoes	Italy	Italy				fruit	strong purple	729		98940			134
Tomato	*Solanum lycopersicum L. cv. Velasco*	A0DMX	Tomatoes	Spain	Spain				fruit	red	1120				1680	137
Tomato	*Solanum lycopersicum L. v. Lazarino*	A0DMX	Tomatoes	Spain	Spain											141
Tomato	*Solanum lycopersicum var. Campbell's seeds*	A0DMX	Tomatoes	Spain	Spain	94.85			fruit	red			15330			130
Tomato	*Solanum lycopersicum var. Diamond seeds*	A0DMX	Tomatoes	Spain	Spain	95.48			fruit	red			17010			130
Tomato	*Solanum lycopersicum var. Heinz(H)*	A0DMX	Tomatoes	Spain	Spain	95.2			fruit	red			12210			130
Tomato	*Solanum lycopersicum var. Heinz(H)*	A0DMX	Tomatoes	Spain	Spain	95.14			fruit	red			14960			130
Tomato	*Solanum lycopersicum var. Heinz(H)-9036*	A0DMX	Tomatoes	Spain	Spain	95.38			fruit	red			11360			130
Tomato	*Solanum lycopersicum var. Kalvert*	A0DMX	Tomatoes	Spain	Spain	95.15			fruit	red			16710			130
Tomato	*Solanum lycopersicum L.*	A0DMX	Tomatoes	United Kingdom	United Kingdom				fruit				1200-5000			86

Table S9.23.4 Tomatoes and similar (A00HQ) (µg/100g) (continuation)

Food name	Scientific name	FoodEx2_TermCode	FoodEx2_TermName	Origin (country)	Purchase (country)	Water (%)	Process	Saponification	Part analysed	Colour	Lutein	Luteoxanthin	Lycopene	Neoxanthin	Phytoene	Ref.
Tomato	*Solanum lycopersicum L.*	A0DMX	Tomatoes	USA					fruit				3900±100			86
Tomato	*Solanum lycopersicum L.*	A0DMX	Tomatoes	Malaysia					fruit				700			86
Ttomato	*Lycopersicum esculentum M.*	A00HX	Pear-shaped tomatoes	Spain	Spain				edible part		72		62273			24

Table S9.23.5 Tomatoes and similar (A00HQ) (µg/100g) (continuation)

Food name	Scientific name	FoodEx2_TermCode	FoodEx2_TermName	Origin (country)	Purchase (country)	Water (%)	Process	Saponification	Part analysed	Colour	Phytofluene	Violaxanthin	Z(v. cis)-lycopene	Z(v. cis)-β-carotene	Z(v. cis)-β-cryptoxanthin	Zeaxanthin	Ref.
Goji berries	*Lycium barbarum*	A0DMR	Wolfberries	Spain	Spain				edible part	red						3260	24
Goji berries	*Lycium barbarum*	A0DMR	Wolfberries	Spain	Spain				edible part							3260	24
Tomato		A0DMX#F20.A0F7P$F20.A07RC	Tomatoes, PART-CONSUMED-ANALYSED = Only peel, With seeds	Greece	Greece	7.65 ± 0.21			only Skin and seeds	red				8.66 (sum of 9-cis and 13-cis)±0.01			136
Tomato	*Lycopersicon esculentum M. var. "for salad"*	A0DMX	Tomatoes	Portugal	Portugal				with skin and seeds	red						<8	125
Tomato	*Lycopersicon esculentum M. var. Lido*	A0DMX	Tomatoes	Portugal	Portugal				with skin and seeds	red						<8	125
Tomato	*Lycopersicon esculentum Mill*	A0DMX	Tomatoes	Italy	Italy			no	fruit	red	500						127

Table S9.23.5 Tomatoes and similar (A00HQ) (µg/100g) (continuation)

Food name	Scientific name	FoodEx2_ TermCode	FoodEx2_ TermName	Origin (country)	Purchase (country)	Water (%)	Process	Saponification	Part analysed	Colour	Phytofluene	Violaxanthin	Z(v. cis)-lycopene	Z(v. cis)-β-carotene	Z(v. cis)-β-cryptoxanthin	Zeaxanthin	Ref.
Tomato	*Lycopersicon esculentum Mill.*	A0DMX	Tomatoes	Spain	United Kingdom				fruit (whole)	-		2550±580					95
Tomato	*Lycopersicon esculentum Mill.*	A0DMX	Tomatoes	Italy	Italy			no	fruit	red	450						127
Tomato	*Lycopersicon esculentum Mill.*	A0DMX	Tomatoes	Italy	Italy			no	fruit	red	620						127
Tomato	*Lycopersicon esculentum Mill.*	A0DMX	Tomatoes	Italy	Italy			no	fruit	red	560						127
Tomato	*Lycopersicon esculentum Mill.*	A0DMX	Tomatoes	Italy	Italy			no	fruit	red	490						127
Tomato	*Lycopersicon esculentum Mill. cv. Carmen*	A0DMX	Tomatoes	Brazil					fruit	red		nd				nd	109
Tomato	*Solanum lycopersicum*	A0DMX	Tomatoes	USA			raw	no						0			28
Tomato	*Solanum lycopersicum L.*	A0DMX	Tomatoes	Spain	Spain				Pulp	red	450 (400-510)						22
Tomato	*Solanum lycopersicum L.*	A0DMX	Tomatoes	Spain	Spain				fruit	red	820						22
Tomato	*Solanum lycopersicum L.*	A0DMX	Tomatoes	Spain	Spain				fruit	red	2850 (2080-3630)						22
Tomato	*Solanum lycopersicum L. cv Cervil*	A00HY	Cherry tomatoes	France	France			no	fruit	green	60						136
Tomato	*Solanum lycopersicum L. cv Cervil*	A00HY	Cherry tomatoes	France	France			no	fruit		1080						132
Tomato	*Solanum lycopersicum L. cv Cervil*	A00HY	Cherry tomatoes	France	France			no	fruit		720						132
Tomato	*Solanum lycopersicum L. cv Cervil*	A00HY	Cherry tomatoes	France	France			no	fruit		700						132
Tomato	*Solanum lycopersicum L. cv Cervil*	A00HY	Cherry tomatoes	France	France			no	fruit		520						132

Table S9.23.5 Tomatoes and similar (A00HQ) (µg/100g) (continuation)

Food name	Scientific name	FoodEx2_ TermCode	FoodEx2_ TermName	Origin (country)	Purchase (country)	Water (%)	Process	Saponification	Part analysed	Colour	Phytofluene	Violaxanthin	Z(v. cis)-lycopene	Z(v. cis)-β-carotene	Z(v. cis)-β-cryptoxanthin	Zeaxanthin	Ref.
Tomato	*Solanum lycopersicum L. cv Cervil*	A00HY	Cherry tomatoes	France	France			no	fruit		50						132
Tomato	*Solanum lycopersicum L. cv Cervil*	A00HY	Cherry tomatoes	France	France			no	fruit		1080						132
Tomato	*Solanum lycopersicum L. cv Cervil*	A00HY	Cherry tomatoes	France	France			no	fruit		860						132
Tomato	*Solanum lycopersicum L. cv Cervil*	A00HY	Cherry tomatoes	France	France			no	fruit		860						132
Tomato	*Solanum lycopersicum L. cv Cervil*	A00HY	Cherry tomatoes	France	France			no	fruit		700						132
Tomato	*Solanum lycopersicum L. cv. Velasco*	A0DMX	Tomatoes	Spain	Spain				fruit	red	780	310	810	150			137

Table S9.24.1 Peppers and similar (A00HZ) (µg/100g)

Food name	Scientific name	FoodEx2_ TermCode	FoodEx2_ TermName	Origin (country)	Purchase (country)	Water (%)	Process	Saponification	Part analysed	Colour	α-carotene	β-carotene	β-cryptoxanthin	ζ-carotene	Antheraxanthin	Ref.
Chili pepper	*Capsicum annuum*	A00JB#F10.A0F2S	Chili peppers, QUALITATIVE-INFO = red	Italy	Italy				whole pepper	red		694.1–2186.9	249.3–1587.5		174.7–527.8	142
Chili pepper	*Capsicum annuum*	A0DMM	Capsicum annuum hot cultivars	Spain	Spain				all sample	red/ yellow	224	245 - 1484	270			144
Chili pepper	*Capsicum baccatum*	A0DML	Capsicum baccatum	Spain	Spain				all sample	red/yellow/Orange	163 - 178	875 - 2544	130 - 421			144

Table S9.24.1 Peppers and similar (A00HZ) (µg/100g) (continuation)

Food name	Scientific name	FoodEx2_ TermCode	FoodEx2_ TermName	Origin (country)	Purchase (country)	Water (%)	Process	Saponification	Part analysed	Colour	α-carotene	β-carotene	β-cryptoxanthin	ζ-carotene	Antheraxanthin	Ref.
Chili pepper	*Capsicum chinense* **var.** ***Naga morich green***	A0DMK	Chinese capsicum	Italy	Italy				berries (fruit)	green		4.6±1.1				143
Chili pepper	*Capsicum pubescens*	A0DMH	Rocoto capsicum	Spain	Spain				all sample	red/ yellow/O range	116	931 - 2409	134 - 445			171
Chilli pepper	*Capsicum annuum* **var.** ***Sinpezon***	A0DMM	Capsicum annuum hot cultivars	Italy	Italy				berries (fruit)	red		10.8±1			0.5±0.1	143
Chilli pepper	*Capsicum chinense* **var.** ***Habanero chocolate***	A0DMK	Chinese capsicum	Italy	Italy				berries (fruit)	chocolate			0.8±0.08			143
Chilli pepper	*Capsicum chinense* **var.** ***Habanero orange***	A0DMK	Chinese capsicum	Italy	Italy				berries (fruit)	orange					9.9±1.1	143
Chilli pepper	*Capsicum chinense* **var.** ***Habanero red type II***	A0DMK	Chinese capsicum	Italy	Italy				berries (fruit)	dark red		15.8±1.8	2.1±0.2			143
Chilli pepper	*Capsicum chinense* **var.** ***Naga morich red***	A0DMK	Chinese capsicum	Italy	Italy				berries (fruit)	red / green		9.8±1.3			0.9±0.02	143
Chilli pepper	*Capsicum chinense* **var.** ***Scotch bonnet***	A0DMK	Chinese capsicum	Italy	Italy				berries (fruit)	yellow	9.8±1.2	15.1±2.3				143
Chilli pepper	*Capsicum chinese* **var.** ***Habanero golden***	A0DMK	Chinese capsicum	Italy	Italy				berries (fruit)	yellow-gold	9.1±0.7	19.1±1.7				143
Pepper	*Capsicum annum*	A00JA	Sweet peppers	Spain	Spain				edible part	red	62	2220				24
Pepper	*Capsicum annum*	A00JA	Sweet peppers	Spain	Spain				edible part	red		693	371			24
Pepper	*Capsicum annum*	A00JA	Sweet peppers	Spain	Spain				edible part	red		414	251			24
Pepper	*Capsicum annum*	A00JA	Sweet peppers	Spain	Spain				edible part	green		255				24

Table S9.24.1 Peppers and similar (A00HZ) (µg/100g) (continuation)

Food name	Scientific name	FoodEx2_ TermCode	FoodEx2_ TermName	Origin (country)	Purchase (country)	Water (%)	Process	Saponification	Part analysed	Colour	α-carotene	β-carotene	β-cryptoxanthin	ζ-carotene	Antheraxanthin	Ref.
Pepper	*Capsicum annum*	A00JA	Sweet peppers	Spain	Spain				edible part	green		205				24
Pepper	*Capsicum annum L.*	A00JA#F10.A 0F2Q	Sweet peppers, QUALITATIVE-INFO = green	USA				no		green	0		0			28
Pepper	*Capsicum annum L.*	A00JA#F10.A 0F5J	Sweet peppers, QUALITATIVE-INFO = Orange	USA				no		orange	98		136			28
Pepper	*Capsicum annum L.*	A00JA#F10.A 0F2S	Sweet peppers, QUALITATIVE-INFO = red	USA				no		red	85		0			28
Pepper	*Capsicum annum L.*	A00JA#F10.A 0F5H	Sweet peppers, QUALITATIVE-INFO = yellow	USA				no		yellow	17		0			28
Pepper	*Capsicum annum L.*	A00JB#F10.A 0F2S	Chili peppers, QUALITATIVE-INFO = red	Turkey	Turkey	14.07 ± 1.50			fruits with seeds	red		12450± 620	16550± 1230			145
Pepper	*Capsicum annum L. '*	A00JB#F10.A 0F2S	Chili peppers, QUALITATIVE-INFO = red	Turkey	Turkey	13.71 ± 1.24			fruits with seeds	red		7290±510	11350± 1100			145
Pepper	*Capsicum annum L. cv 'AmazonF1'*	A00JB#F10.A 0F2S	Chili peppers, QUALITATIVE-INFO = red	Turkey	Turkey	13.49 ± 2.78			fruits with seeds	red		8390± 1530	16300± 2460			145
Pepper	*Capsicum annum L. cv 'Serademre 8'*	A00JB#F10.A 0F2S	Chili peppers, QUALITATIVE-INFO = red	Turkey	Turkey	15.04 ± 0.30			fruit with seeds	red		6950±840	11500±60			145
Pepper	*Capsicum annuum*	A00JA	Sweet peppers	Italy	Italy							6530–15 400				25
Pepper	*Capsicum annuum*	A00JA#F10.A 0F2Q	Sweet peppers, QUALITATIVE-INFO = green	Italy	Italy					green	nd – 139	2–335	nd – 110			25
Pepper	*Capsicum annuum*	A00JA#F10.A 0F5J	Sweet peppers, QUALITATIVE-INFO = Orange	Italy	Italy					orange	72	400	3			25

Table S9.24.1 Peppers and similar (A00HZ) (µg/100g) (continuation)

Food name	Scientific name	FoodEx2_ TermCode	FoodEx2_ TermName	Origin (country)	Purchase (country)	Water (%)	Process	Saponification	Part analysed	Colour	α-carotene	β-carotene	β-cryptoxanthin	ζ-carotene	Antheraxanthin	Ref.
Pepper	*Capsicum annuum*	A00JA#F10.A0F2S	Sweet peppers, QUALITATIVE-INFO = red	Italy	Italy					red	nd – 287	1441–2390	248–447			25
Pepper	*Capsicum annuum*	A00JA#F10.A0F5H	Sweet peppers, QUALITATIVE-INFO = yellow	Italy	Italy					yellow	10–28	42–62	15–41			25
Pepper	*Capsicum annuum*	A00JA#F10.A0F2S	Sweet peppers, QUALITATIVE-INFO = red	Germany	Germany	87.1			edible part	red	510	3250	1010			53
Pepper	*Capsicum annuum*	A00JA#F10.A0F2Q	Sweet peppers, QUALITATIVE-INFO = green	Germany	Germany	92.5			edible part	green	10	10	2			53
Pepper	*Capsicum annuum*	A00JA	Sweet peppers	Hungary	Hungary	20.3			whole plant	red		198.6				146
Pepper	*Capsicum annuum*	A00JB#F10.A0F2S	Chili peppers, QUALITATIVE-INFO = red	Turkey	Turkey	86.7			ripe fruits	red		12030± 1100	15430± 1090			145
Pepper	*Capsicum annuum*	A0DMM	Capsicum annuum hot cultivars	Spain	Spain				fruit	orange						22
Pepper	*Capsicum annuum*	A0DMM	Capsicum annuum hot cultivars	Spain	Spain				fruit	red						22
Pepper	*Capsicum annuum*	A0DMM	Capsicum annuum hot cultivars	Spain	Spain				fruit	yellow						22
Pepper	*Capsicum annuum L.*	A00JA	Sweet peppers	Spain	Spain				edible part	red	62	2200				24
Pepper	*Capsicum annuum L.*	A00JA	Sweet peppers	USA		92.6–94.0			edible part			84-762				147
Pepper	*Capsicum annuum L.*	A0DMM	Capsicum annuum hot cultivars	Spain	Spain				fruit	red		24520	39263		47477	148
Pepper	*Capsicum annuum L.*	A0DMM	Capsicum annuum hot cultivars	Spain	Spain				fruit	red		2512			379	148
Pepper	*Capsicum annuum L.*	A0DMM	Capsicum annuum hot cultivars	Spain	Spain				fruit	red		1496	351		854	148

Table S9.24.1 Peppers and similar (A00HZ) (µg/100g) (continuation)

Food name	Scientific name	FoodEx2_ TermCode	FoodEx2_ TermName	Origin (country)	Purchase (country)	Water (%)	Process	Saponification	Part analysed	Colour	α-carotene	β-carotene	β-cryptoxanthin	ζ-carotene	Antheraxanthin	Ref.
Pepper	*Capsicum annuum L.*	A0DMM	Capsicum annuum hot cultivars	Spain	Spain				fruit	red		19289	22648		30213	148
Pepper	*Capsicum annuum L.*	A0DMM	Capsicum annuum hot cultivars	Spain	Spain				fruit	red		32751	31550		37118	148
Pepper	*Capsicum annuum L.*	A0DMM	Capsicum annuum hot cultivars	Spain	Spain				fruit	red		26528	30444		45295	148
Pepper	*Capsicum annuum L.*	A0DMM	Capsicum annuum hot cultivars	Spain	Spain				fruit	red		4124			564	148
Pepper	*Capsicum annuum L.*	A0DMM	Capsicum annuum hot cultivars	Spain	Spain				fruit	red		2366			465	148
Pepper	*Capsicum annuum L.*	A0DMM	Capsicum annuum hot cultivars	Spain	Spain				fruit	red		40033	41752		30350	148
Pepper	*Capsicum annuum L.*	A0DMM	Capsicum annuum hot cultivars	Spain	Spain				fruit	red		31750	31934		29689	148
Pepper	*Capsicum annuum L.*	A0DMM	Capsicum annuum hot cultivars	Spain	Spain				fruit	red		41972	36462		53563	148
Pepper	*Capsicum annuum L.*	A0DMM	Capsicum annuum hot cultivars	Spain	Spain				fruit	red		3684			553	148
Pepper	*Capsicum annuum L.*	A0DMM	Capsicum annuum hot cultivars	Spain	Spain				fruit	red		2150			499	148
Pepper	*Capsicum annuum L.*	A0DMM	Capsicum annuum hot cultivars	Spain	Spain				fruit	red		32412	34387		26744	148
Pepper	*Capsicum annuum L.*	A0DMM	Capsicum annuum hot cultivars	Spain	Spain				fruit	red		34759	30816		35128	148
Pepper	*Capsicum annuum L.*	A0DMM	Capsicum annuum hot cultivars	Spain	Spain				fruit	red		48692	45000		49406	148
Pepper	*Capsicum annuum L.*	A0DMM	Capsicum annuum hot cultivars	Spain	Spain				fruit	red		2176			364	148

Table S9.24.1 Peppers and similar (A00HZ) (µg/100g) (continuation)

Food name	Scientific name	FoodEx2_ TermCode	FoodEx2_ TermName	Origin (country)	Purchase (country)	Water (%)	Process	Saponification	Part analysed	Colour	α-carotene	β-carotene	β-cryptoxanthin	ζ-carotene	Antheraxanthin	Ref.
Pepper	*Capsicum annuum L.*	A0DMM	Capsicum annuum hot cultivars	Spain	Spain				fruit	red		1701			358	148
Pepper	*Capsicum annuum L.*	A0DMM	Capsicum annuum hot cultivars	Spain	Spain				fruit	red		15369	17961		15857	148
Pepper	*Capsicum annuum L.*	A0DMM	Capsicum annuum hot cultivars	Spain	Spain				fruit	red		30414	23456		22698	148
Pepper	*Capsicum annuum L.*	A0DMM	Capsicum annuum hot cultivars	Spain	Spain				fruit	red		25279	19533		26275	148
Pepper	*Capsicum annuum L.*	A0DMM	Capsicum annuum hot cultivars	Spain	Spain				fruit	red		2302			876	148
Pepper	*Capsicum annuum L.*	A0DMM	Capsicum annuum hot cultivars	Spain	Spain				fruit	red		2325			491	148
Pepper	*Capsicum annuum L.*	A0DMM	Capsicum annuum hot cultivars	Spain	Spain				fruit	red		34212	41291		19367	148
Pepper	*Capsicum annuum L.*	A0DMM	Capsicum annuum hot cultivars	Spain	Spain				fruit	red		65155	37626		36619	148
Pepper	*Capsicum annuum L.*	A0DMM	Capsicum annuum hot cultivars	Spain	Spain				fruit	red		44754	34433		44970	148
Pepper	*Capsicum annuum L.*	A0DMM	Capsicum annuum hot cultivars	Spain	Spain				fruit	red		4572			657	148
Pepper	*Capsicum annuum L.*	A0DMM	Capsicum annuum hot cultivars	Spain	Spain				fruit	red		5195			657	148
Pepper	*Capsicum annuum L.*	A0DMM	Capsicum annuum hot cultivars	Spain	Spain				fruit	red		39987	50069		20257	148
Pepper	*Capsicum annuum L.*	A0DMM	Capsicum annuum hot cultivars	Spain	Spain				fruit	red		48912	57207		27890	148
Pepper	*Capsicum annuum L.*	A0DMM	Capsicum annuum hot cultivars	Spain	Spain				fruit	red		68301	68173		53674	148

Table S9.24.1 Peppers and similar (A00HZ) (µg/100g) (continuation)

Food name	Scientific name	FoodEx2_ TermCode	FoodEx2_ TermName	Origin (country)	Purchase (country)	Water (%)	Process	Saponification	Part analysed	Colour	α-carotene	β-carotene	β-cryptoxanthin	ζ-carotene	Antheraxanthin	Ref.
Pepper	*Capsicum annuum L.*	A0DMM	Capsicum annuum hot cultivars	Spain	Spain				fruit	red		1380			164	148
Pepper	*Capsicum annuum L.*	A0DMM	Capsicum annuum hot cultivars	Spain	Spain				fruit	red		2301			545	148
Pepper	*Capsicum annuum L.*	A0DMM	Capsicum annuum hot cultivars	Spain	Spain				fruit	red		62352	63351		55298	148
Pepper	*Capsicum annuum L.*	A0DMM	Capsicum annuum hot cultivars	Spain	Spain				fruit	red		49373	55288		61326	148
Pepper	*Capsicum annuum L.*	A0DMM	Capsicum annuum hot cultivars	Spain	Spain				fruit	red		43501	33530		54832	148
Pepper	*Capsicum annuum L.*	A0DMM	Capsicum annuum hot cultivars	Spain	Spain				fruit	red		2556			531	148
Pepper	*Capsicum annuum L.*	A0DMM	Capsicum annuum hot cultivars	Spain	Spain				fruit	red		3193	2021			148
Pepper	*Capsicum annuum L.*	A0DMM	Capsicum annuum hot cultivars	Spain	Spain				fruit	red		45403	47008			148
Pepper	*Capsicum annuum L.*	A0DMM	Capsicum annuum hot cultivars	Spain	Spain				fruit	red		51099	47273		78525	148
Pepper	*Capsicum annuum L.*	A0DMM	Capsicum annuum hot cultivars	Spain	Spain				fruit	red		71951	60761		86060	148
Pepper	*Capsicum annuum L.*	A0DMM	Capsicum annuum hot cultivars	Spain	Spain				fruit	red		3658			394	148
Pepper	*Capsicum annuum L.*	A0DMM	Capsicum annuum hot cultivars	Spain	Spain				fruit	red		3127	2134		2071	148
Pepper	*Capsicum annuum L.*	A0DMM	Capsicum annuum hot cultivars	Spain	Spain				fruit	red		42308	47571		26718	148
Pepper	*Capsicum annuum L.*	A0DMM	Capsicum annuum hot cultivars	Spain	Spain				fruit	red		73669	95977		41230	148

Table S9.24.1 Peppers and similar (A00HZ) (μg/100g) (continuation)

Food name	Scientific name	FoodEx2_ TermCode	FoodEx2_ TermName	Origin (country)	Purchase (country)	Water (%)	Process	Saponification	Part analysed	Colour	α-carotene	β-carotene	β-cryptoxanthin	ζ-carotene	Antheraxanthin	Ref.
Pepper	*Capsicum annuum L.*	A0DMM	Capsicum annuum hot cultivars	Spain	Spain				fruit	red		53985	47559		45996	148
Pepper	*Capsicum annuum L.*	A0DMM	Capsicum annuum hot cultivars	Spain	Spain				fruit	red		4978			566	148
Pepper	*Capsicum annuum L.*	A0DMM	Capsicum annuum hot cultivars	Spain	Spain				fruit	red		4150	1404		2512	148
Pepper	*Capsicum annuum L.*	A0DMM	Capsicum annuum hot cultivars	Spain	Spain				fruit	red		11217	14799		17967	148
Pepper	*Capsicum annuum L.*	A0DMM	Capsicum annuum hot cultivars	Spain	Spain				fruit	red		19972	28520		32859	148
Pepper	*Capsicum annuum L.*	A0DMM	Capsicum annuum hot cultivars	Spain	Spain				edible part	red		693	371			24
Pepper	*Capsicum annuum L.*	A0DMM	Capsicum annuum hot cultivars	Spain	Spain				edible part	red		414	251			24
Pepper	*Capsicum annuum L.*	A0DMM	Capsicum annuum hot cultivars	Spain	Spain				edible part	green		255				24
Pepper	*Capsicum annuum L.*	A0DMM	Capsicum annuum hot cultivars	Spain	Spain				edible part	green		205				24
Pepper	*Capsicum annuum L.*	A0DMM	Capsicum annuum hot cultivars	Spain	Spain				edible part	red	387.9		282.6± 17.5			56
Pepper	*Capsicum annuum L.*	A0DMM	Capsicum annuum hot cultivars	Netherlands	United Kingdom				without seeds	red	2500±570	22190± 5300	3420±320		4340±310	95
Pepper	*Capsicum annuum L.*	A0DMM	Capsicum annuum hot cultivars	Israel	United Kingdom				without seeds	yellow	2010±250	7530± 1170	31850± 6050			95
Pepper	*Capsicum annuum L.*	A0DMM	Capsicum annuum hot cultivars	Netherlands	Netherlands				without seeds	green		1900±730				95
Pepper	*Capsicum annuum L. cv.Datler*	A0DMM	Capsicum annuum hot cultivars	Spain	Spain				fruit	red					674	148

Table S9.24.1 Peppers and similar (A00HZ) (µg/100g) (continuation)

Food name	Scientific name	FoodEx2_ TermCode	FoodEx2_ TermName	Origin (country)	Purchase (country)	Water (%)	Process	Saponification	Part analysed	Colour	α-carotene	β-carotene	β-cryptoxanthin	ζ-carotene	Antheraxanthin	Ref.
Pepper	*Capsicum annuum L. cv.Datler*	A0DMM	Capsicum annuum hot cultivars	Spain	Spain				fruit	red					230	148
Pepper	*Capsicum annuum L. cv.Datler*	A0DMM	Capsicum annuum hot cultivars	Spain	Spain				fruit	red					48134	148
Pepper	*Capsicum annuum L. cv.Datler*	A0DMM	Capsicum annuum hot cultivars	Spain	Spain				fruit	red					50483	148
Pepper	*Capsicum annuum L. cv.Datler*	A0DMM	Capsicum annuum hot cultivars	Spain	Spain				fruit	red					63633	148
Pepper	*Capsicum annuum L. cv.Mulato*	A0DMM	Capsicum annuum hot cultivars	Spain	Spain				fruit	red					533	148
Pepper	*Capsicum annuum L. cv.Mulato*	A0DMM	Capsicum annuum hot cultivars	Spain	Spain				fruit	red					3625	148
Pepper	*Capsicum annuum L. cv.Mulato*	A0DMM	Capsicum annuum hot cultivars	Spain	Spain				fruit	red					15975	148
Pepper	*Capsicum annuum L. cv.Mulato*	A0DMM	Capsicum annuum hot cultivars	Spain	Spain				fruit	red					22360	148
Pepper	*Capsicum annuum L. cv.Mulato*	A0DMM	Capsicum annuum hot cultivars	Spain	Spain				fruit	red					55002	148
Pepper	*Capsicum annuum L. Hybrid F1 Magali*	A00JA#F20.A07RD	Sweet peppers, PART-CONSUMED-ANALYSED = W/o seeds	Brazil					with skin, without seeds	red	nd	580	nd			109
Pepper	*Capsicum annuum L. var. Agridulce*	A00JB#F10.A0F2S	Chili peppers, QUALITATIVE-INFO = red	Spain	Spain				fruit	green		798				149

Table S9.24.1 Peppers and similar (A00HZ) (µg/100g) (continuation)

Food name	Scientific name	FoodEx2_ TermCode	FoodEx2_ TermName	Origin (country)	Purchase (country)	Water (%)	Process	Saponification	Part analysed	Colour	α-carotene	β-carotene	β-cryptoxanthin	ζ-carotene	Antheraxanthin	Ref.
Pepper	*Capsicum annuum L.* ***var. Agridulce***	A0DMM	Capsicum annuum hot cultivars	Spain	Spain				fruits	red		9951	7672		4408	149
Pepper	*Capsicum annuum L.* ***var. Bola***	A0DMM	Capsicum annuum hot cultivars	Spain	Spain				fruits	green		623				149
Pepper	*Capsicum annuum L.* ***var. Bola***	A0DMM	Capsicum annuum hot cultivars	Spain	Spain				fruits	red		5128	3559		3318	149
Pepper	*Capsicum annuum L. var. Grosum, Bailey.*	A0DMM#F20.A07RD	Capsicum annuum hot cultivars, PART-CONSUMED-ANALYSED = W/o seeds	Spain	Spain	90			without seeds	red		478±50	251±24			24
Pepper	*Capsicum annuum L. var. Grosum, Bailey.*	A0DMM#F20.A07RD	Capsicum annuum hot cultivars, PART-CONSUMED-ANALYSED = W/o seeds	Spain	Spain	91			without seeds	red		768±93	371±36			24
Pepper	*Capsicum annuum L. var. Grosum, Bailey.*	A0DMM#F20.A07RD	Capsicum annuum hot cultivars, PART-CONSUMED-ANALYSED = W/o seeds	Spain	Spain	94			without seeds	green		205±11				24
Pepper	*Capsicum annuum L. var. Grosum, Bailey.*	A0DMM#F20.A07RD	Capsicum annuum hot cultivars, PART-CONSUMED-ANALYSED = W/o seeds	Spain	Spain				without seeds	green		255±10				24
Pepper	*Capsicum annuum, L.*	A0DMM	Capsicum annuum hot cultivars	Spain	Spain				fruit	red	387.9	1272.1	282.6			56

Table S9.24.2 Peppers and similar (A00HZ) (µg/100g) (continuation)

Food name	Scientific name	FoodEx2_ TermCode	FoodEx2_ TermName	Origin (country)	Purchase (country)	Water (%)	Process	Saponification	Part analysed	Colour	Capsanthin	Capsorubin	Cucurbitaxanthin	E(v. trans)-α-carotene	E(v. trans)-β-carotene	Ref.
Chili pepper	*Capsicum annuum*	A00JB#F10.A0F2S	Chili peppers, QUALITATIVE-INFO = red	Italy	Italy					red	282.6–1865.3					142
Chili pepper	*Capsicum annuum*	A0DMM	Capsicum annuum hot cultivars	Spain	Spain				all sample	red/ yellow	16332					144
Chili pepper	*Capsicum baccatum*	A0DML	Capsicum baccatum	Spain	Spain				all sample	red/ yellow/ Orange	4486 - 12923					144
Chili pepper	*Capsicum pubescens*	A0DMH	Rocoto capsicum	Spain	Spain				all sample	red/ yellow/ Orange	5713 - 7895					144
Chilli pepper	*Capsicum annuum* ***var. Jalapeno***	A0DMM	Capsicum annuum hot cultivars	Italy	Italy				berries (fruit)	red	12.5±1.2					143
Chilli pepper	*Capsicum annuum* ***var. Serrano***	A0DMM	Capsicum annuum hot cultivars	Italy	Italy				berries (fruit)	red	7.9±0.8					143
Chilli pepper	*Capsicum annuum* ***var. Sinpezon***	A0DMM	Capsicum annuum hot cultivars	Italy	Italy				berries (fruit)	red	2.9±0.4					143
Chilli pepper	*Capsicum chinense* ***var. Habanero chocolate***	A0DMK	Chinese capsicum	Italy	Italy				berries (fruit)	chocolate	8.6±1.1					143
Chilli pepper	*Capsicum chinense* ***var. Habanero orange***	A0DMK	Chinese capsicum	Italy	Italy				berries (fruit)	orange	4.5±0.6					143
Chilli pepper	*Capsicum chinense* ***var. Habanero red***	A0DMK	Chinese capsicum	Italy	Italy				berries (fruit)	dark orange	5.4±0.8					143
Chilli pepper	*Capsicum chinense* ***var. Habanero red***	A0DMK	Chinese capsicum	Italy	Italy				berries (fruit)	dark red	6.4±0.8					143
Chilli pepper	*Capsicum chinense* ***var. Habanero white***	A0DMK	Chinese capsicum	Italy	Italy				berries (fruit)	white	0.7±0.02					143

Table S9.24.2 Peppers and similar (A00HZ) (µg/100g) (continuation)

Food name	Scientific name	FoodEx2_ TermCode	FoodEx2_ TermName	Origin (country)	Purchase (country)	Water (%)	Process	Saponification	Part analysed	Colour	Capsanthin	Capsorubin	Cucurbitaxanthin	E(v. trans)-α-carotene	E(v. trans)-β-carotene	Ref.
Chilli pepper	*Capsicum chinense var. Naga morich red*	A0DMK	Chinese capsicum	Italy	Italy				berries (fruit)	red / green	8.1±1.1					143
Chilli pepper	*Capsicum frutescens*	A0DMJ	Tabasco capsicum	Italy	Italy				berries (fruit)	red	12.5±1.2					143
Pepper	*Capsicum annum L.*	A00JA#F10.A 0F2Q	Sweet peppers, QUALITATIVE-INFO = green	USA				no		green					38	28
Pepper	*Capsicum annum L.*	A00JA#F10.A 0F5J	Sweet peppers, QUALITATIVE-INFO = Orange	USA				no		orange					926	28
Pepper	*Capsicum annum L.*	A00JA#F10.A 0F2S	Sweet peppers, QUALITATIVE-INFO = red	USA				no		red					153	28
Pepper	*Capsicum annum L.*	A00JA#F10.A 0F5H	Sweet peppers, QUALITATIVE-INFO = yellow	USA				no		yellow					38	28
Pepper	*Capsicum annum L.*	A00JB#F10.A 0F2S	Chili peppers, QUALITATIVE-INFO = red	Turkey	Turkey	14.07 ± 1.50			with seeds	red	127000± 21780	5860± 1600	27820± 6090			145
Pepper	*Capsicum annum L. cv 'AmazonF1'*	A00JB#F10.A 0F2S	Chili peppers, QUALITATIVE-INFO = red	Turkey	Turkey	13.49 ± 2.78			with seeds	red	85000± 10950	2590± 1080	17120± 5640			145
Pepper	*Capsicum annum L. cv 'Kusak*	A00JB#F10.A 0F2S	Chili peppers, QUALITATIVE-INFO = red	Turkey	Turkey	13.71 ± 1.24			with seeds	red	76900± 7130	4020±410	18140± 2290			145
Pepper	*Capsicum annum L. cv 'Serademre 8'*	A00JB#F10.A 0F2S	Chili peppers, QUALITATIVE-INFO = red	Turkey	Turkey	15.04 ± 0.30			with seeds	red	89150± 9520	4000± 350	22230± 3630			145
Pepper	*Capsicum annuum cv*	A00JB#F10.A 0F2S	Chili peppers, QUALITATIVE-INFO = red	Turkey	Turkey	86.7			ripe fruits	red	127000± 8940	6720±370	26740± 560			145
Pepper	*Capsicum annuum L.*	A0DMM	Capsicum annuum hot cultivars	Spain	Spain				fruit	red	443535	23291				148
Pepper	*Capsicum annuum L.*	A0DMM	Capsicum annuum hot cultivars	Spain	Spain				fruit	red	7683	326				148
Pepper	*Capsicum annuum L.*	A0DMM	Capsicum annuum hot cultivars	Spain	Spain				fruit	red	245514	9022				148

Table S9.24.2 Peppers and similar (A00HZ) (µg/100g) (continuation)

Food name	Scientific name	FoodEx2_ TermCode	FoodEx2_ TermName	Origin (country)	Purchase (country)	Water (%)	Process	Saponification	Part analysed	Colour	Capsanthin	Capsorubin	Cucurbitaxanthin	E(v. trans)-α-carotene	E(v. trans)-β-carotene	Ref.
Pepper	*Capsicum annuum L.*	A0DMM	Capsicum annuum hot cultivars	Spain	Spain				fruit	red	356931	16280				148
Pepper	*Capsicum annuum L.*	A0DMM	Capsicum annuum hot cultivars	Spain	Spain				fruit	red	414219	19732				148
Pepper	*Capsicum annuum L.*	A0DMM	Capsicum annuum hot cultivars	Spain	Spain				fruit	red	274398	15919				148
Pepper	*Capsicum annuum L.*	A0DMM	Capsicum annuum hot cultivars	Spain	Spain				fruit	red	328644	19093				148
Pepper	*Capsicum annuum L.*	A0DMM	Capsicum annuum hot cultivars	Spain	Spain				fruit	red	560570	40887				148
Pepper	*Capsicum annuum L.*	A0DMM	Capsicum annuum hot cultivars	Spain	Spain				fruit	red	253445	13933				148
Pepper	*Capsicum annuum L.*	A0DMM	Capsicum annuum hot cultivars	Spain	Spain				fruit	red	381159	22088				148
Pepper	*Capsicum annuum L.*	A0DMM	Capsicum annuum hot cultivars	Spain	Spain				fruit	red	636308	35442				148
Pepper	*Capsicum annuum L.*	A0DMM	Capsicum annuum hot cultivars	Spain	Spain				fruit	red	146706	6440				148
Pepper	*Capsicum annuum L.*	A0DMM	Capsicum annuum hot cultivars	Spain	Spain				fruit	red	257922	10578				148
Pepper	*Capsicum annuum L.*	A0DMM	Capsicum annuum hot cultivars	Spain	Spain				fruit	red	301257	12576				148
Pepper	*Capsicum annuum L.*	A0DMM	Capsicum annuum hot cultivars	Spain	Spain				fruit	red	216962	9008				148
Pepper	*Capsicum annuum L.*	A0DMM	Capsicum annuum hot cultivars	Spain	Spain				fruit	red	410392	15605				148
Pepper	*Capsicum annuum L.*	A0DMM	Capsicum annuum hot cultivars	Spain	Spain				fruit	red	571435	18119				148

Table S9.24.2 Peppers and similar (A00HZ) (µg/100g) (continuation)

Food name	Scientific name	FoodEx2_ TermCode	FoodEx2_ TermName	Origin (country)	Purchase (country)	Water (%)	Process	Saponification	Part analysed	Colour	Capsanthin	Capsorubin	Cucurbitaxanthin	E(v. trans)-α-carotene	E(v. trans)-β-carotene	Ref.
Pepper	*Capsicum annuum L.*	A0DMM	Capsicum annuum hot cultivars	Spain	Spain				fruit	red	2545219	8488				148
Pepper	*Capsicum annuum L.*	A0DMM	Capsicum annuum hot cultivars	Spain	Spain				fruit	red	390488	15345				148
Pepper	*Capsicum annuum L.*	A0DMM	Capsicum annuum hot cultivars	Spain	Spain				fruit	red	598510	23150				148
Pepper	*Capsicum annuum L.*	A0DMM	Capsicum annuum hot cultivars	Spain	Spain				fruit	red	307					148
Pepper	*Capsicum annuum L.*	A0DMM	Capsicum annuum hot cultivars	Spain	Spain				fruit	red	469715	23057				148
Pepper	*Capsicum annuum L.*	A0DMM	Capsicum annuum hot cultivars	Spain	Spain				fruit	red	540512	25738				148
Pepper	*Capsicum annuum L.*	A0DMM	Capsicum annuum hot cultivars	Spain	Spain				fruit	red	605135	29076				148
Pepper	*Capsicum annuum L.*	A0DMM	Capsicum annuum hot cultivars	Spain	Spain				fruit	red	28124	1399				148
Pepper	*Capsicum annuum L.*	A0DMM	Capsicum annuum hot cultivars	Spain	Spain				fruit	red	371136	6666				148
Pepper	*Capsicum annuum L.*	A0DMM	Capsicum annuum hot cultivars	Spain	Spain				fruit	red	799443	27781				148
Pepper	*Capsicum annuum L.*	A0DMM	Capsicum annuum hot cultivars	Spain	Spain				fruit	red	794968	32254				148
Pepper	*Capsicum annuum L.*	A0DMM	Capsicum annuum hot cultivars	Spain	Spain				fruit	red	18756	720				148
Pepper	*Capsicum annuum L.*	A0DMM	Capsicum annuum hot cultivars	Spain	Spain				fruit	red	258702	19628				148
Pepper	*Capsicum annuum L.*	A0DMM	Capsicum annuum hot cultivars	Spain	Spain				fruit	red	362877	25519				148

Table S9.24.2 Peppers and similar (A00HZ) (µg/100g) (continuation)

Food name	Scientific name	FoodEx2_ TermCode	FoodEx2_ TermName	Origin (country)	Purchase (country)	Water (%)	Process	Saponification	Part analysed	Colour	Capsanthin	Capsorubin	Cucurbitaxanthin	E(v. trans)-α-carotene	E(v. trans)-β-carotene	Ref.
Pepper	*Capsicum annuum L.*	A0DMM	Capsicum annuum hot cultivars	Spain	Spain				fruit	red	414103	28877				148
Pepper	*Capsicum annuum L.*	A0DMM	Capsicum annuum hot cultivars	Spain	Spain				fruit	red	21391	3733				148
Pepper	*Capsicum annuum L.*	A0DMM	Capsicum annuum hot cultivars	Spain	Spain				fruit	red	159486	6604				148
Pepper	*Capsicum annuum L.*	A0DMM	Capsicum annuum hot cultivars	Spain	Spain				fruit	red	271506	15350				148
Pepper	*Capsicum annuum L.*	A0DMM	Capsicum annuum hot cultivars	Spain	Spain				edible part	red				90.4±4.9	1135.3± 140.6	56
Pepper	*Capsicum annuum L.*	A0DMM	Capsicum annuum hot cultivars	Netherlands	United Kingdom				without seeded	red	14920± 1370	3100±320				95
Pepper	*Capsicum annuum L. cv.Datler*	A0DMM	Capsicum annuum hot cultivars	Spain	Spain				fruit	red	1829	277				148
Pepper	*Capsicum annuum L. cv.Datler*	A0DMM	Capsicum annuum hot cultivars	Spain	Spain				fruit	red	444785	17830				148
Pepper	*Capsicum annuum L. cv.Datler*	A0DMM	Capsicum annuum hot cultivars	Spain	Spain				fruit	red	466991	19430				148
Pepper	*Capsicum annuum L. cv.Datler*	A0DMM	Capsicum annuum hot cultivars	Spain	Spain				fruit	red	543216	28433				148
Pepper	*Capsicum annuum L. cv.Mulato*	A0DMM	Capsicum annuum hot cultivars	Spain	Spain				fruit	red	32500	3516				148
Pepper	*Capsicum annuum L. cv.Mulato*	A0DMM	Capsicum annuum hot cultivars	Spain	Spain				fruit	red	222002	15203				148
Pepper	*Capsicum annuum L. cv.Mulato*	A0DMM	Capsicum annuum hot cultivars	Spain	Spain				fruit	red	364500	15203				148

Table S9.24.2 Peppers and similar (A00HZ) (µg/100g) (continuation)

Food name	Scientific name	FoodEx2_TermCode	FoodEx2_TermName	Origin (country)	Purchase (country)	Water (%)	Process	Saponification	Part analysed	Colour	Capsanthin	Capsorubin	Cucurbitaxanthin	E(v. trans)-α-carotene	E(v. trans)-β-carotene	Ref.
Pepper	*Capsicum annuum L. cv.Mulato*	A0DMM	Capsicum annuum hot cultivars	Spain	Spain				fruit	red	501188	18183				148
Pepper	*Capsicum annuum L.* ***var. Agridulce***	A0DMM	Capsicum annuum hot cultivars	Spain	Spain				fruit	red	65647	7898				149
Pepper	*Capsicum annuum L.* ***var. Bola***	A0DMM	Capsicum annuum hot cultivars	Spain	Spain				fruit	red	52321	5344				149
Pepper	*Capsicum annuum, L.*	A0DMM	Capsicum annuum hot cultivars	Spain	Spain				fruit	red				90.4	1135.3	56

Table S9.24.3 Peppers and similar (A00HZ) (µg/100g) (continuation)

Food name	Scientific name	FoodEx2_TermCode	FoodEx2_TermName	Origin (country)	Purchase (country)	Water (%)	Process	Saponification	Part analysed	Colour	E(v. trans)-β-cryptoxanthin	E(v. trans)-lutein	E(v. trans)-lycopene	E(v. trans)-zeaxanthin	Lactucaxanthin	Ref.
Pepper	*Capsicum annum L.*	A00JA#F10.A0F2Q	Sweet peppers, QUALITATIVE-INFO = green	USA				no		green		173		0		28
Pepper	*Capsicum annum L.*	A00JA#F10.A0F5J	Sweet peppers, QUALITATIVE-INFO = Orange	USA				no		orange		208		1665		28
Pepper	*Capsicum annum L.*	A00JA#F10.A0F2S	Sweet peppers, QUALITATIVE-INFO = red	USA				no		red		0		22		28
Pepper	*Capsicum annum L.*	A00JA#F10.A0F5H	Sweet peppers, QUALITATIVE-INFO = yellow	USA				no		yellow		139		18		28

Table S9.24.4 Peppers and similar (A00HZ) (µg/100g) (continuation)

Food name	Scientific name	FoodEx2_ TermCode	FoodEx2_ TermName	Origin (country)	Purchase (country)	Water (%)	Process	Saponification	Part analysed	Colour	Lutein	Luteoxanthin	Lycopene	Neoxanthin	Phytoene	Ref.
Chili pepper	*Capsicum annuum*	A0DMM	Capsicum annuum hot cultivars	Spain	Spain				all sample	red/ yellow	573					144
Chili pepper	*Capsicum baccatum*	A0DML	Capsicum baccatum	Spain	Spain				all sample	red/ yellow/ Orange	1013					144
Chili pepper	*Capsicum chinense* ***var. Naga morich green***	A0DMK	Chinese capsicum	Italy	Italy				berries (fruit)	green	29.5±3.1					143
Chili pepper	*Capsicum pubescens*	A0DMH	Rocoto capsicum	Spain	Spain				all sample	red/ yellow/ Orange	933 - 1346					144
Chilli pepper	*Capsicum annuum* ***var. Sinpezon***	A0DMM	Capsicum annuum hot cultivars	Italy	Italy				berries (fruit)	red	0.4±0.02					143
Chilli pepper	*Capsicum chinense* ***var. Habanero chocolate***	A0DMK	Chinese capsicum	Italy	Italy				berries (fruit)	chocolate	4.4±0.6	0.4±0.02				143
Chilli pepper	*Capsicum chinense* ***var. Habanero orange***	A0DMK	Chinese capsicum	Italy	Italy				berries (fruit)	orange	4.8±0.7	0.7±0.02				143
Chilli pepper	*Capsicum chinense* ***var. Habanero red type II***	A0DMK	Chinese capsicum	Italy	Italy				berries (fruit)	dark red		0.1±0.02				143
Chilli pepper	*Capsicum chinense* ***var. Habanero white***	A0DMK	Chinese capsicum	Italy	Italy				berries (fruit)	white	48.3±6.2					143
Chilli pepper	*Capsicum chinense* ***var. Scotch bonnet***	A0DMK	Chinese capsicum	Italy	Italy				berries (fruit)	yellow	15.6±2.2					143
Chilli pepper	*Capsicum chinese* ***var.*** *Habanero golden*	A0DMK	Chinese capsicum	Italy	Italy				berries (fruit)	yellow-gold	17.3±0.9	0.1±0.01				143

Table S9.24.4 Peppers and similar (A00HZ) (µg/100g) (continuation)

Food name	Scientific name	FoodEx2_ TermCode	FoodEx2_ TermName	Origin (country)	Purchase (country)	Water (%)	Process	Saponification	Part analysed	Colour	Lutein	Luteoxanthin	Lycopene	Neoxanthin	Phytoene	Ref.
Pepper	*Capsicum annum*	A00JA	Sweet peppers	Spain	Spain				edible part	green	377					24
Pepper	*Capsicum annum*	A00JA	Sweet peppers	Spain	Spain				edible part	green	341					24
Pepper	*Capsicum annuum*	A00JA	Sweet peppers	Italy	Italy					red	nd					24
Pepper	*Capsicum annuum*	A00JA#F10.A 0F2Q	Sweet peppers, QUALITATIVE-INFO = green	Italy	Italy					green	92–911		nd			24
Pepper	*Capsicum annuum*	A00JA#F10.A 0F5J	Sweet peppers, QUALITATIVE-INFO = Orange	Italy	Italy					orange	245					24
Pepper	*Capsicum annuum*	A00JA#F10.A 0F2S	Sweet peppers, QUALITATIVE-INFO = red	Italy	Italy					red	248–8506					24
Pepper	*Capsicum annuum*	A00JA#F10.A 0F5H	Sweet peppers, QUALITATIVE-INFO = yellow	Italy	Italy					yellow	419–638					24
Pepper	*Capsicum annuum*	A00JA#F10.A 0F2Q	Sweet peppers, QUALITATIVE-INFO = green	Germany	Germany	92.5			edible part	green	410			120		53
Pepper	*Capsicum annuum*	A00JB#F10.A 0F2S	Chili peppers, QUALITATIVE-INFO = red	Italy	Italy				whole pepper	red	15.1–52.2			8.7–82.2		142
Pepper	*Capsicum annuum*	A0DMM	Capsicum annuum hot cultivars	Spain	Spain				fruit	orange					1010	22
Pepper	*Capsicum annuum*	A0DMM	Capsicum annuum hot cultivars	Spain	Spain				fruit	red					1690	22
Pepper	*Capsicum annuum*	A0DMM	Capsicum annuum hot cultivars	Spain	Spain				fruit	yellow					420	22
Pepper	*Capsicum annuum L*	A0DMM	Capsicum annuum hot cultivars	Spain	Spain				fruit	red	2496			872		148
Pepper	*Capsicum annuum L.*	A0DMM	Capsicum annuum hot cultivars	Spain	Spain				fruit	red	9309			2113		148
Pepper	*Capsicum annuum L.*	A0DMM	Capsicum annuum hot cultivars	Spain	Spain				fruit	red	2194			2736		148
Pepper	*Capsicum annuum L.*	A0DMM	Capsicum annuum hot cultivars	Spain	Spain				fruit	red	2129			791		148

Table S9.24.4 Peppers and similar (A00HZ) (µg/100g) (continuation)

Food name	Scientific name	FoodEx2_ TermCode	FoodEx2_ TermName	Origin (country)	Purchase (country)	Water (%)	Process	Saponification	Part analysed	Colour	Lutein	Luteoxanthin	Lycopene	Neoxanthin	Phytoene	Ref.
Pepper	*Capsicum annuum L.*	A0DMM	Capsicum annuum hot cultivars	Spain	Spain				fruit	red	5875			1768		148
Pepper	*Capsicum annuum L.*	A0DMM	Capsicum annuum hot cultivars	Spain	Spain				fruit	red	3497			2330		148
Pepper	*Capsicum annuum L.*	A0DMM	Capsicum annuum hot cultivars	Spain	Spain				fruit	red	3200			1356		148
Pepper	*Capsicum annuum L.*	A0DMM	Capsicum annuum hot cultivars	Spain	Spain				fruit	red	7596			2445		148
Pepper	*Capsicum annuum L.*	A0DMM	Capsicum annuum hot cultivars	Spain	Spain				fruit	red	6138			2106		148
Pepper	*Capsicum annuum L.*	A0DMM	Capsicum annuum hot cultivars	Spain	Spain				fruit	red	4906			1102		148
Pepper	*Capsicum annuum L.*	A0DMM	Capsicum annuum hot cultivars	Spain	Spain				fruit	red	18744					148
Pepper	*Capsicum annuum L.*	A0DMM	Capsicum annuum hot cultivars	Spain	Spain				fruit	red	7278			2113		148
Pepper	*Capsicum annuum L.*	A0DMM	Capsicum annuum hot cultivars	Spain	Spain				fruit	red	5499			1970		148
Pepper	*Capsicum annuum L.*	A0DMM	Capsicum annuum hot cultivars	Spain	Spain				fruit	red	2259			735		148
Pepper	*Capsicum annuum L.*	A0DMM	Capsicum annuum hot cultivars	Spain	Spain				fruit	red	7113			1973		148
Pepper	*Capsicum annuum L.*	A0DMM	Capsicum annuum hot cultivars	Spain	Spain				fruit	red	6548			2313		148
Pepper	*Capsicum annuum L.*	A0DMM	Capsicum annuum hot cultivars	Spain	Spain				fruit	red	4495					148
Pepper	*Capsicum annuum L.*	A0DMM	Capsicum annuum hot cultivars	Spain	Spain				fruit	red	11826			4217		148
Pepper	*Capsicum annuum L.*	A0DMM	Capsicum annuum hot cultivars	Spain	Spain				fruit	red	14837			5055		148

Table S9.24.4 Peppers and similar (A00HZ) (µg/100g) (continuation)

Food name	Scientific name	FoodEx2_ TermCode	FoodEx2_ TermName	Origin (country)	Purchase (country)	Water (%)	Process	Saponification	Part analysed	Colour	Lutein	Luteoxanthin	Lycopene	Neoxanthin	Phytoene	Ref.
Pepper	*Capsicum annuum L.*	A0DMM	Capsicum annuum hot cultivars	Spain	Spain				fruit	red	3061			1418		148
Pepper	*Capsicum annuum L.*	A0DMM	Capsicum annuum hot cultivars	Spain	Spain				fruit	red	6116			2977		148
Pepper	*Capsicum annuum L.*	A0DMM	Capsicum annuum hot cultivars	Spain	Spain				fruit	red	9683			3138		148
Pepper	*Capsicum annuum L.*	A0DMM	Capsicum annuum hot cultivars	Spain	Spain				fruit	red	1263					148
Pepper	*Capsicum annuum L.*	A0DMM	Capsicum annuum hot cultivars	Spain	Spain				fruit	red	11083			3276		148
Pepper	*Capsicum annuum L.*	A0DMM	Capsicum annuum hot cultivars	Spain	Spain				fruit	red	3305			4731		148
Pepper	*Capsicum annuum L.*	A0DMM	Capsicum annuum hot cultivars	Spain	Spain				fruit	red	1492					148
Pepper	*Capsicum annuum L.*	A0DMM	Capsicum annuum hot cultivars	Spain	Spain				fruit	red	14047			4485		148
Pepper	*Capsicum annuum L.*	A0DMM	Capsicum annuum hot cultivars	Spain	Spain				fruit	red	4005			3471		148
Pepper	*Capsicum annuum L.*	A0DMM	Capsicum annuum hot cultivars	Spain	Spain				fruit	red	3277			483		148
Pepper	*Capsicum annuum L.*	A0DMM	Capsicum annuum hot cultivars	Spain	Spain				edible part	green	377					24
Pepper	*Capsicum annuum L.*	A0DMM	Capsicum annuum hot cultivars	Spain	Spain				edible part	green	341					24
Pepper	*Capsicum annuum L.*	A0DMM	Capsicum annuum hot cultivars	Netherlands	United Kingdom				without seed	red				6480±470	3400± 1060	95
Pepper	*Capsicum annuum L.*	A0DMM	Capsicum annuum hot cultivars	Israel	United Kingdom				without seed	yellow	16560± 3130			35301± 6520	6170± 1390	95
Pepper	*Capsicum annuum L.*	A0DMM	Capsicum annuum hot cultivars	Netherlands	Netherlands				without seed	green	6410±180				30±10	95

Table S9.24.4 Peppers and similar (A00HZ) (µg/100g) (continuation)

Food name	Scientific name	FoodEx2_ TermCode	FoodEx2_ TermName	Origin (country)	Purchase (country)	Water (%)	Process	Saponification	Part analysed	Colour	Lutein	Luteoxanthin	Lycopene	Neoxanthin	Phytoene	Ref.
Pepper	*Capsicum annuum L. cv.Datler*	A0DMM	Capsicum annuum hot cultivars	Spain	Spain				fruit	red	5940			1827		148
Pepper	*Capsicum annuum L. cv.Datler*	A0DMM	Capsicum annuum hot cultivars	Spain	Spain				fruit	red	1199			2041		148
Pepper	*Capsicum annuum L. cv.Datler*	A0DMM	Capsicum annuum hot cultivars	Spain	Spain				fruit	red	912					148
Pepper	*Capsicum annuum L. cv.Mulato*	A0DMM	Capsicum annuum hot cultivars	Spain	Spain				fruit	red	24643			7480		148
Pepper	*Capsicum annuum L. cv.Mulato*	A0DMM	Capsicum annuum hot cultivars	Spain	Spain				fruit	red	32020			12005		148
Pepper	*Capsicum annuum L. Hybrid F1 Magali*	A00JA#F20.A07RD	Sweet peppers, PART-CONSUMED-ANALYSED = W/o seeds	Brazil					with skin, without seeds	red	750		nd	nd		109
Pepper	*Capsicum annuum L.* ***var. Agridulce***	A00JB#F10.A0F2S	Chili peppers, QUALITATIVE-INFO = red	Spain	Spain				fruit	green	1409			885		153
Pepper	*Capsicum annuum L.* ***var. Bola***	A0DMM	Capsicum annuum hot cultivars	Spain	Spain				fruit	green	795			812		149
Pepper	*Capsicum annuum L. var. Grosum, Bailey.*	A0DMM#F20.A07RD	Capsicum annuum hot cultivars, PART-CONSUMED-ANALYSED = W/o seeds	Spain	Spain	94			without seeds	green	341±16					24
Pepper	*Capsicum annuum L. var. Grosum, Bailey.*	A0DMM#F20.A07RD	Capsicum annuum hot cultivars, PART-CONSUMED-ANALYSED = W/o seeds	Spain	Spain				without seeds	green	377±83					24

Table S9.24.4 Peppers and similar (A00HZ) (µg/100g) (continuation)

Food name	Scientific name	FoodEx2_ TermCode	FoodEx2_ TermName	Origin (country)	Purchase (country)	Water (%)	Process	Saponification	Part analysed	Colour	Lutein	Luteoxanthin	Lycopene	Neoxanthin	Phytoene	Ref.
Pepper	*Capsicum annuum L. var. Grosum, Bailey.*	A0DMM#F20. A07RD	Capsicum annuum hot cultivars, PART-CONSUMED-ANALYSED = W/o seeds	Spain	Spain	90			without seeds	red					721±8	24
Pepper	*Capsicum annuum L. var. Grosum, Bailey.*	A0DMM#F20. A07RD	Capsicum annuum hot cultivars, PART-CONSUMED-ANALYSED = W/o seeds	Spain	Spain	91			without seeds	red					1034±35	24

Table S9.24.5 Peppers and similar (A00HZ) (µg/100g) (continuation)

Food name	Scientific name	FoodEx2_ TermCode	FoodEx2_ TermName	Origin (country)	Purchase (country)	Water (%)	Process	Saponification	Part analysed	Colour	Phytofluene	Violaxanthin	Z(v. cis)-lycopene	Z(v. cis)-β-carotene	Z(v. cis)-β-cryptoxanthin	Zeaxanthin	Ref.
Chilli pepper	*Capsicum annuum*	A0DMM	Capsicum annuum hot cultivars	Spain	Spain				all sample	red/ yellow		2654 - 4627					144
Chilli pepper	*Capsicum annuum var. Sinpezon*	A0DMM	Capsicum annuum hot cultivars	Italy	Italy				berries (fruit)	red						0.1±0.02	143
Chilli pepper	*Capsicum baccatum*	A0DML	Capsicum baccatum	Spain	Spain				all sample	red/ yellow/ orange		1516 - 7211					144
Chilli pepper	*Capsicum chinense var. Habanero chocolate*	A0DMK	Chinese capsicum	Italy	Italy				berries (fruit)	chocola te						2.1±0.8	143

Table S9.24.5 Peppers and similar (A00HZ) (µg/100g) (continuation)

Food name	Scientific name	FoodEx2_TermCode	FoodEx2_TermName	Origin (country)	Purchase (country)	Water (%)	Process	Saponification	Part analysed	Colour	Phytofluene	Violaxanthin	Z(v. cis)-lycopene	Z(v. cis)-β-carotene	Z(v. cis)-β-cryptoxanthin	Zeaxanthin	Ref.
Chilli pepper	*Capsicum chinense var. Habanero orange*	A0DMK	Chinese capsicum	Italy	Italy				berries (fruit)	orange						10.8±0.9	143
Chilli pepper	*Capsicum chinense var. Habanero red type I*	A0DMK	Chinese capsicum	Italy	Italy				berries (fruit)	dark orange						0.7±0.01	143
Chilli pepper	*Capsicum chinense var. Habanero red type II*	A0DMK	Chinese capsicum	Italy	Italy				berries (fruit)	dark red						3.5±0.2	143
Chilli pepper	*Capsicum chinense var. Naga morich*	A0DMK	Chinese capsicum	Italy	Italy				berries (fruit)	green						2.1±0.4	143
Chilli pepper	*Capsicum chinense var. Naga morich red*	A0DMK	Chinese capsicum	Italy	Italy				berries (fruit)	red / green						0.5±0.02	143
Chilli pepper	*Capsicum chinense var. Scotch bonnet*	A0DMK	Chinese capsicum	Italy	Italy				berries (fruit)	yellow						1.6±0.4	143
Chilli pepper	*Capsicum chinese var. Habanero golden*	A0DMK	Chinese capsicum	Italy	Italy				berries (fruit)	yellow-gold					7.3±0,3	0.3±0.01	143
Chilli pepper	*Capsicum frutescens*	A0DMJ	Tabasco capsicum	Italy	Italy				berries (fruit)	red						0.2±0.01	143
Chilli pepper	*Capsicum pubescens*	A0DMH	Rocoto capsicum	Spain	Spain				all sample	red/ yellow/ Orange		1051 - 3583					144

Table S9.24.5 Peppers and similar (A00HZ) (µg/100g) (continuation)

Food name	Scientific name	FoodEx2_ TermCode	FoodEx2_ TermName	Origin (country)	Purchase (country)	Water (%)	Process	Saponification	Part analysed	Colour	Phytofluene	Violaxanthin	Z(v. cis)- lycopene	Z(v. cis)-β-carotene	Z(v. cis)-β-cryptoxanthin	Zeaxanthin	Ref.
Chilli peppers	*Capsicum annuum var. Serrano*	A0DMM	Capsicum annuum hot cultivars	Italy	Italy				berries (fruit)	red						0.6±0.02	143
Pepper	*Capsicum annum*	A00JA	Sweet peppers	Spain	Spain				edible part	red						390	24
Pepper	*Capsicum annum*	A00JA	Sweet peppers	Spain	Spain				edible part	red						289	24
Pepper	*Capsicum annum L.*	A00JA#F10 .A0F2Q	Sweet peppers, QUALITATIVE-INFO = green	USA				no		green				6			28
Pepper	*Capsicum annum L.*	A00JA#F10 .A0F5J	Sweet peppers, QUALITATIVE-INFO = Orange	USA				no		orange				98			28
Pepper	*Capsicum annum L.*	A00JA#F10 .A0F2S	Sweet peppers, QUALITATIVE-INFO = red	USA				no		red				201			28
Pepper	*Capsicum annum L.*	A00JA#F10 .A0F5H	Sweet peppers, QUALITATIVE-INFO = yellow	USA				no		yellow				11			28
Pepper	*Capsicum annum L.*	A00JB#F10 .A0F2S	Chili peppers, QUALITATIVE-INFO = red	Turkey	Turkey	14.07 ± 1.50			with seeds	red		8930± 1930				32710± 10810	145
Pepper	*Capsicum annum L. cv 'AmazonF1'*	A00JB#F10 .A0F2S	Chili peppers, QUALITATIVE-INFO = red	Turkey	Turkey	13.49 ± 2.78			with seeds	red		5930±810				46240± 5560	145
Pepper	*Capsicum annum L. cv 'Kusak*	A00JB#F10 .A0F2S	Chili peppers, QUALITATIVE-INFO = red	Turkey	Turkey	13.71 ± 1.24			with seeds	red		5030±960				21280± 3840	145
Pepper	*Capsicum annum L. cv 'Serademre 8*	A00JB#F10 .A0F2S	Chili peppers, QUALITATIVE-INFO = red	Turkey	Turkey	15.04 ± 0.30			with seeds	red		6430±850				28000±70	145
Pepper	*Capsicum annuum*	A00JA#F10 .A0F2Q	Sweet peppers, QUALITATIVE-INFO = green	Italy	Italy					green						nd – 42	25

Table S9.24.5 Peppers and similar (A00HZ) (µg/100g) (continuation)

Food name	Scientific name	FoodEx2_ TermCode	FoodEx2_ TermName	Origin (country)	Purchase (country)	Water (%)	Process	Saponification	Part analysed	Colour	Phytofluene	Violaxanthin	Z(v. cis)-lycopene	Z(v. cis)-β-carotene	Z(v. cis)-β-cryptoxanthin	Zeaxanthin	Ref.
Pepper	*Capsicum annuum*	A00JA#F10 .A0F5J	Sweet peppers, QUALITATIVE-INFO = Orange	Italy	Italy					orange						nd	25
Pepper	*Capsicum annuum*	A00JA#F10 .A0F2S	Sweet peppers, QUALITATIVE-INFO = red	Italy	Italy					red						593–1350	25
Pepper	*Capsicum annuum*	A00JA#F10 .A0F5H	Sweet peppers, QUALITATIVE-INFO = yellow	Italy	Italy					yellow						nd	25
Pepper	*Capsicum annuum*	A00JA#F10 .A0F2S	Sweet peppers, QUALITATIVE-INFO = red	Germany	Germany	87.1			edible part	red						2200	53
Pepper	*Capsicum annuum*	A00JA#F10 .A0F2Q	Sweet peppers, QUALITATIVE-INFO = green	Germany	Germany	92.5			edible part	green		120					53
Pepper	*Capsicum annuum*	A00JB#F10 .A0F2S	Chili peppers, QUALITATIVE-INFO = red	Italy	Italy					red		121.2–548.6				68.6–448.4	142
Pepper	*Capsicum annuum*	A00JB#F10 .A0F2S	Chili peppers, QUALITATIVE-INFO = red	Turkey	Turkey	86.7			ripe fruits	red		10110± 880				40990± 5500	145
Pepper	*Capsicum annuum*	A0DMM	Capsicum annuum hot cultivars	Spain	Spain				fruit	orange	310						22
Pepper	*Capsicum annuum*	A0DMM	Capsicum annuum hot cultivars	Spain	Spain				fruit	red	510						22
Pepper	*Capsicum annuum*	A0DMM	Capsicum annuum hot cultivars	Spain	Spain				fruit	yellow	220						22
Pepper	*Capsicum annuum L.*	A0DMM	Capsicum annuum hot cultivars	Spain	Spain				fruit	red		21785				63590	148
Pepper	*Capsicum annuum L.*	A0DMM	Capsicum annuum hot cultivars	Spain	Spain				fruit	red		2829					148
Pepper	*Capsicum annuum L.*	A0DMM	Capsicum annuum hot cultivars	Spain	Spain				fruit	red		2395				3641	148

Table S9.24.5 Peppers and similar (A00HZ) (µg/100g) (continuation)

Food name	Scientific name	FoodEx2_ TermCode	FoodEx2_ TermName	Origin (country)	Purchase (country)	Water (%)	Process	Saponification	Part analysed	Colour	Phytofluene	Violaxanthin	Z(v. cis)-lycopene	Z(v. cis)-β-carotene	Z(v. cis)-β-cryptoxanthin	Zeaxanthin	Ref.
Pepper	*Capsicum annuum L.*	A0DMM	Capsicum annuum hot cultivars	Spain	Spain				fruit	red		7115				47661	148
Pepper	*Capsicum annuum L.*	A0DMM	Capsicum annuum hot cultivars	Spain	Spain				fruit	red		17640				55909	148
Pepper	*Capsicum annuum L.*	A0DMM	Capsicum annuum hot cultivars	Spain	Spain				fruit	red		20901				56293	148
Pepper	*Capsicum annuum L.*	A0DMM	Capsicum annuum hot cultivars	Spain	Spain				fruit	red		2286					148
Pepper	*Capsicum annuum L.*	A0DMM	Capsicum annuum hot cultivars	Spain	Spain				fruit	red		3369				4173	148
Pepper	*Capsicum annuum L.*	A0DMM	Capsicum annuum hot cultivars	Spain	Spain				fruit	red		8321				56375	148
Pepper	*Capsicum annuum L.*	A0DMM	Capsicum annuum hot cultivars	Spain	Spain				fruit	red		16336				57715	148
Pepper	*Capsicum annuum L.*	A0DMM	Capsicum annuum hot cultivars	Spain	Spain				fruit	red		33239				67827	148
Pepper	*Capsicum annuum L.*	A0DMM	Capsicum annuum hot cultivars	Spain	Spain				fruit	red		3702					148
Pepper	*Capsicum annuum L.*	A0DMM	Capsicum annuum hot cultivars	Spain	Spain				fruit	red		3569					148
Pepper	*Capsicum annuum L.*	A0DMM	Capsicum annuum hot cultivars	Spain	Spain				fruit	red		8026				49269	148
Pepper	*Capsicum annuum L.*	A0DMM	Capsicum annuum hot cultivars	Spain	Spain				fruit	red						53142	148
Pepper	*Capsicum annuum L.*	A0DMM	Capsicum annuum hot cultivars	Spain	Spain				fruit	red		29857				71692	148
Pepper	*Capsicum annuum L.*	A0DMM	Capsicum annuum hot cultivars	Spain	Spain				fruit	red		2342					148
Pepper	*Capsicum annuum L.*	A0DMM	Capsicum annuum hot cultivars	Spain	Spain				fruit	red		2779					148

Table S9.24.5 Peppers and similar (A00HZ) (µg/100g) (continuation)

Food name	Scientific name	FoodEx2_TermCode	FoodEx2_TermName	Origin (country)	Purchase (country)	Water (%)	Process	Saponification	Part analysed	Colour	Phytofluene	Violaxanthin	Z(v. cis)-lycopene	Z(v. cis)-β-carotene	Z(v. cis)-β-cryptoxanthin	Zeaxanthin	Ref.
Pepper	*Capsicum annuum L.*	A0DMM	Capsicum annuum hot cultivars	Spain	Spain				fruit	red		4276				35556	148
Pepper	*Capsicum annuum L.*	A0DMM	Capsicum annuum hot cultivars	Spain	Spain				fruit	red		10247				36186	148
Pepper	*Capsicum annuum L.*	A0DMM	Capsicum annuum hot cultivars	Spain	Spain				fruit	red		10954				40570	148
Pepper	*Capsicum annuum L.*	A0DMM	Capsicum annuum hot cultivars	Spain	Spain				fruit	red		1580					148
Pepper	*Capsicum annuum L.*	A0DMM	Capsicum annuum hot cultivars	Spain	Spain				fruit	red		3210					148
Pepper	*Capsicum annuum L.*	A0DMM	Capsicum annuum hot cultivars	Spain	Spain				fruit	red						65097	148
Pepper	*Capsicum annuum L.*	A0DMM	Capsicum annuum hot cultivars	Spain	Spain				fruit	red		15506				94839	148
Pepper	*Capsicum annuum L.*	A0DMM	Capsicum annuum hot cultivars	Spain	Spain				fruit	red		21943				135178	148
Pepper	*Capsicum annuum L.*	A0DMM	Capsicum annuum hot cultivars	Spain	Spain				fruit	red		6312					148
Pepper	*Capsicum annuum L.*	A0DMM	Capsicum annuum hot cultivars	Spain	Spain				fruit	red		7714					148
Pepper	*Capsicum annuum L.*	A0DMM	Capsicum annuum hot cultivars	Spain	Spain				fruit	red		7742				123113	148
Pepper	*Capsicum annuum L.*	A0DMM	Capsicum annuum hot cultivars	Spain	Spain				fruit	red		10305				83500	148
Pepper	*Capsicum annuum L.*	A0DMM	Capsicum annuum hot cultivars	Spain	Spain				fruit	red		25535				165010	148
Pepper	*Capsicum annuum L.*	A0DMM	Capsicum annuum hot cultivars	Spain	Spain				fruit	red		1726					148
Pepper	*Capsicum annuum L.*	A0DMM	Capsicum annuum hot cultivars	Spain	Spain				fruit	red		4421				519	148

Table S9.24.5 Peppers and similar (A00HZ) (μg/100g) (continuation)

Food name	Scientific name	FoodEx2_ TermCode	FoodEx2_ TermName	Origin (country)	Purchase (country)	Water (%)	Process	Saponification	Part analysed	Colour	Phytofluene	Violaxanthin	Z(v. cis)-lycopene	Z(v. cis)-β-carotene	Z(v. cis)-β-cryptoxanthin	Zeaxanthin	Ref.
Pepper	*Capsicum annuum L.*	A0DMM	Capsicum annuum hot cultivars	Spain	Spain				fruit	red		23994				103275	148
Pepper	*Capsicum annuum L.*	A0DMM	Capsicum annuum hot cultivars	Spain	Spain				fruit	red		23025				105990	148
Pepper	*Capsicum annuum L.*	A0DMM	Capsicum annuum hot cultivars	Spain	Spain				fruit	red		22303				74089	148
Pepper	*Capsicum annuum L.*	A0DMM	Capsicum annuum hot cultivars	Spain	Spain				fruit	red		4955					148
Pepper	*Capsicum annuum L.*	A0DMM	Capsicum annuum hot cultivars	Spain	Spain				fruit	red		1351				3096	148
Pepper	*Capsicum annuum L.*	A0DMM	Capsicum annuum hot cultivars	Spain	Spain				fruit	red		9727				63589	148
Pepper	*Capsicum annuum L.*	A0DMM	Capsicum annuum hot cultivars	Spain	Spain				fruit	red		19435				95379	148
Pepper	*Capsicum annuum L.*	A0DMM	Capsicum annuum hot cultivars	Spain	Spain				fruit	red		16971				80678	148
Pepper	*Capsicum annuum L.*	A0DMM	Capsicum annuum hot cultivars	Spain	Spain				fruit	red		3707					148
Pepper	*Capsicum annuum L.*	A0DMM	Capsicum annuum hot cultivars	Spain	Spain				fruit	red		5348				2329	148
Pepper	*Capsicum annuum L.*	A0DMM	Capsicum annuum hot cultivars	Spain	Spain				fruit	red		8741				32067	148
Pepper	*Capsicum annuum L.*	A0DMM	Capsicum annuum hot cultivars	Spain	Spain				fruit	red		27282				66728	148
Pepper	*Capsicum annuum L.*	A0DMM	Capsicum annuum hot cultivars	Spain	Spain				fruit	red		24402				45431	148
Pepper	*Capsicum annuum L.*	A0DMM	Capsicum annuum hot cultivars	Spain	Spain				fruit	red		6839					148
Pepper	*Capsicum annuum L.*	A0DMM	Capsicum annuum hot cultivars	Spain	Spain				fruit	red		4362				8711	148

Table S9.24.5 Peppers and similar (A00HZ) (µg/100g) (continuation)

Food name	Scientific name	FoodEx2_ TermCode	FoodEx2_ TermName	Origin (country)	Purchase (country)	Water (%)	Process	Saponification	Part analysed	Colour	Phytofluene	Violaxanthin	Z(v. cis)-lycopene	Z(v. cis)-β-carotene	Z(v. cis)-β-cryptoxanthin	Zeaxanthin	Ref.
Pepper	*Capsicum annuum L.*	A0DMM	Capsicum annuum hot cultivars	Spain	Spain				fruit	red		3954				30036	148
Pepper	*Capsicum annuum L.*	A0DMM	Capsicum annuum hot cultivars	Spain	Spain				fruit	red		6089				35845	148
Pepper	*Capsicum annuum L.*	A0DMM	Capsicum annuum hot cultivars	Spain	Spain				edible part	red						390	24
Pepper	*Capsicum annuum L.*	A0DMM	Capsicum annuum hot cultivars	Spain	Spain				edible part	red						289	24
Pepper	*Capsicum annuum L.*	A0DMM	Capsicum annuum hot cultivars	Spain	Spain				edible part	red				136.8			56
Pepper	*Capsicum annuum L.*	A0DMM	Capsicum annuum hot cultivars	Netherlands	United Kingdom				without seed	red						1100	95
Pepper	*Capsicum annuum L.*	A0DMM	Capsicum annuum hot cultivars	Israel	United Kingdom				without seed	yellow						910	95
Pepper	*Capsicum annuum L.*	A0DMM	Capsicum annuum hot cultivars	Netherlands	Netherlands				without seed	green		3550± 600					95
Pepper	*Capsicum annuum L. cv.Datler*	A0DMM	Capsicum annuum hot cultivars	Spain	Spain				fruit	red		1608					148
Pepper	*Capsicum annuum L. cv.Datler*	A0DMM	Capsicum annuum hot cultivars	Spain	Spain				fruit	red		2055				3078	148
Pepper	*Capsicum annuum L. cv.Datler*	A0DMM	Capsicum annuum hot cultivars	Spain	Spain				fruit	red		12221				97448	148
Pepper	*Capsicum annuum L. cv.Datler*	A0DMM	Capsicum annuum hot cultivars	Spain	Spain				fruit	red		23767				82084	148
Pepper	*Capsicum annuum L. cv.Datler*	A0DMM	Capsicum annuum hot cultivars	Spain	Spain				fruit	red		25511				80920	148
Pepper	*Capsicum annuum L. cv.Mulato*	A0DMM	Capsicum annuum hot cultivars	Spain	Spain				fruit	red		8125					148

Table S9.24.5 Peppers and similar (A00HZ) (µg/100g) (continuation)

Food name	Scientific name	FoodEx2_ TermCode	FoodEx2_ TermName	Origin (country)	Purchase (country)	Water (%)	Process	Saponification	Part analysed	Colour	Phytofluene	Violaxanthin	Z(v. cis)- lycopene	Z(v. cis)-β- carotene	Z(v. cis)-β- cryptoxanthin	Zeaxanthin	Ref.
Pepper	*Capsicum annuum L. cv.Mulato*	A0DMM	Capsicum annuum hot cultivars	Spain	Spain				fruit	red		9227				4510	148
Pepper	*Capsicum annuum L. cv.Mulato*	A0DMM	Capsicum annuum hot cultivars	Spain	Spain				fruit	red		18545				46656	148
Pepper	*Capsicum annuum L. cv.Mulato*	A0DMM	Capsicum annuum hot cultivars	Spain	Spain				fruit	red		18545				60044	148
Pepper	*Capsicum annuum L. cv.Mulato*	A0DMM	Capsicum annuum hot cultivars	Spain	Spain				fruit	red		23880				72950	148
Pepper	*Capsicum annuum L. Hybrid F1 Magali*	A00JA#F20 .A07RD	Sweet peppers, PART-CONSUMED-ANALYSED = W/o seeds	Brazil					with skin, without seeds	red		270				nd	109
Pepper	*Capsicum annuum L. var. Agridulce*	A00JB#F10 .A0F2S	Chili peppers, QUALITATIVE-INFO = red	Spain	Spain				fruit	green		793					149
Pepper	*Capsicum annuum L. var. Agridulce*	A0DMM	Capsicum annuum hot cultivars	Spain	Spain				fruit	red		8417				9996	149
Pepper	*Capsicum annuum L. var. Bola*	A0DMM	Capsicum annuum hot cultivars	Spain	Spain				fruit	green		1040					149
Pepper	*Capsicum annuum L. var. Bola*	A0DMM	Capsicum annuum hot cultivars	Spain	Spain				fruit	red		5268				4030	149
Pepper	*Capsicum annuum L. var. Grosum, Bailey.*	A0DMM#F 20.A07RD	Capsicum annuum hot cultivars, PART-CONSUMED-ANALYSED = W/o seeds	Spain	Spain	90			fruit without seeds	red						289±37	24

Table S9.24.5 Peppers and similar (A00HZ) (µg/100g) (continuation)

Food name	Scientific name	FoodEx2_ TermCode	FoodEx2_ TermName	Origin (country)	Purchase (country)	Water (%)	Process	Saponification	Part analysed	Colour	Phytofluene	Violaxanthin	Z(v. cis)-lycopene	Z(v. cis)-β-carotene	Z(v. cis)-β-cryptoxanthin	Zeaxanthin	Ref.
Pepper	*Capsicum annuum L. var. Grosum, Bailey.*	A0DMM#F20.A07RD	Capsicum annuum hot cultivars, PART-CONSUMED-ANALYSED = W/o seeds	Spain	Spain	91			fruit without seeds	red						390±90	24
Pepper	*Capsicum annuum, L.*	A0DMM	Capsicum annuum hot cultivars	Spain	Spain				fruit	red				9 cis-b carotene 86,8 / 13-cis-b-carotene 50,0			56

Table S9.25.1 Aubergines and similar (A00JC) (µg/100g)

Food name	Scientific name	FoodEx2_ TermCode	FoodEx2_ TermName	Origin (country)	Purchase (country)	Water (%)	Process	Saponification	Part analysed	Colour	α-carotene	β-carotene	β-cryptoxanthin	ζ-carotene	Antheraxanthin	Ref.
Eggplant	*Solanum melongena*	A00JD	Aubergines	Italy	Italy						nd	1080	nd			25
Eggplant	*Solanum melongena*	A00JD	Aubergines	Spain	Spain				edible part	white		50				24
Eggplant	*Solanum melongena*	A00JD	Aubergines	Spain	Spain				edible part			50				24

Table S9.25.2 Aubergines and similar (A00JC) (µg/100g) (continuation)

Food name	Scientific name	FoodEx2_ TermCode	FoodEx2_ TermName	Origin (country)	Purchase (country)	Water (%)	Process	Saponification	Part analysed	Colour	Lutein	Luteoxanthin	Lycopene	Neoxanthin	Phytoene	Ref.
Eggplant	*Solanum melongena*	A00JD	Aubergines	Italy	Italy						170		nd			25

Table S9.26.1 Cucurbits with edible peel (A00JL) (µg/100g)

Food name	Scientific name	FoodEx2_ TermCode	FoodEx2_ TermName	Origin (country)	Purchase (country)	Water (%)	Process	Saponification	Part analysed	Colour	α-carotene	β-carotene	β-cryptoxanthin	ζ-carotene	Antheraxanthin	Ref.
Bottle gourd	*Lagenaria vulgaris*	A00KL	Bottle gourds	Bangladesh	Bangladesh				leaves			2034± 248				106
Cucumber	*Cucumis sativus*	A00JM	Cucumbers	Spain	Spain				edible part			11				24
Cucumber	*Cucumis sativus L.*	A00JM	Cucumbers	Italy	Italy						nd	112–270	nd			25
Cucumber	*Cucumis sativus L.*	A00JM	Cucumbers	Spain	Spain				edible part	green		11				24
Cucumber	*Cucumis sativus L.*	A00JM#F20. A07QF	Cucumbers, PART-CONSUMED-ANALYSED = W/o peel	Spain	Spain	96			pulp+seeds without skin	white		11±1				24
Cucumber	*Cucumis sativus L.*	A00JM	Cucumbers	USA				no			0		0			52
Gac fruit	*Momordica cochinchinensis Spreng.*	A16QK	Gac	Australia		72-89			the aril around the seeds	orange		0.2-3.3				150
Summer squash	*Cucurbita pepo L.*	A00JS	Summer squashes	Germany	Germany	95.2			edible part			200	11			53
Summer squash	*Cucurbita pepo L.*	A00JS	Summer squashes	Spain	Spain				edible part	green/ green+ white		26	11			24

Table S9.26.1 Cucurbits with edible peel (A00JL) (µg/100g) (continuation)

Food name	Scientific name	FoodEx2_ TermCode	FoodEx2_ TermName	Origin (country)	Purchase (country)	Water (%)	Process	Saponification	Part analysed	Colour	α-carotene	β-carotene	β-cryptoxanthin	ζ-carotene	Antheraxanthin	Ref.
Summer squash	*Cucurbita pepo L.*	A00JS	Summer squashes	Spain	Spain				edible part	green/ green+ white		23	6			24
Summer squash	*Cucurbita pepo L.*	A00JS#F28.A0BA1$F20.A07QE	Summer squashes, PROCESS = Cooking and similar thermal preparation processes, PART-CONSUMED-ANALYSED = With peel	USA			cooked	no	with skin		0		0			28
Summer squash	*Curcubita pepo L.*	A00JS	Summer squashes	Spain	Spain				edible part	white		26	11			24
Summer squash	*Curcubita pepo L.*	A00JS	Summer squashes	Spain	Spain				edible part	white		23	6			24
Summer squash	*Curcubita pepo, L. var. medullusa, Alef.*	A00JS	Summer squashes	Spain	Spain	95			pulp+ seeds	orange		21±2	6±2			24
Summer squash	*Curcubita pepo, L. var. medullusa, Alef.*	A00JS	Summer squashes	Spain	Spain	95			pulp+seeds	orange		28±2	11±1			24

Table S9.26.2 Cucurbits with edible peel (A00JL) (µg/100g) (continuation)

Food name	Scientific name	FoodEx2_ TermCode	FoodEx2_ TermName	Origin (country)	Purchase (country)	Water (%)	Process	Saponification	Part analysed	Colour	Capsanthin	Capsorubin	Cucurbitaxanthin	E(v. trans)-α-carotene	E(v. trans)-β-carotene	Ref.
Cucumber	*Cucumis sativus L.*	A00JM	Cucumbers	USA				no							87	28
Summer squash	*Cucurbita pepo L.*	A00JS#F28.A0BA1$F20.A07QE	Summer squashes, PROCESS = Cooking and similar thermal preparation processes, PART-CONSUMED-ANALYSED = With peel	USA			cooked	no	with skin						311	28

Table S9.26.3 Cucurbits with edible peel (A00JL) (µg/100g) (continuation)

Food name	Scientific name	FoodEx2_ TermCode	FoodEx2_ TermName	Origin (country)	Purchase (country)	Water (%)	Process	Saponification	Part analysed	Colour	E(v. trans)-β-cryptoxanthin	E(v. trans)-lutein	E(v. trans)-lycopene	E(v. trans)-zeaxanthin	Lactucaxanthin	Ref.
Cucumber	*Cucumis sativus L.*	A00JM	Cucumbers	USA				no				361		0		28
Summer squash	*Cucurbita pepo L.*	A00JS#F28.A0BA1$F20.A07QE	Summer squashes, PROCESS = Cooking and similar thermal preparation processes, PART-CONSUMED-ANALYSED = With peel	USA			cooked	no	with skin			1355		0		28

Table S9.26.4 Cucurbits with edible peel (A00JL) (µg/100g) (continuation)

Food name	Scientific name	FoodEx2_ TermCode	FoodEx2_ TermName	Origin (country)	Purchase (country)	Water (%)	Process	Saponification	Part analysed	Colour	Lutein	Luteoxanthin	Lycopene	Neoxanthin	Phytoene	Ref.
Cucumber	*Cucumis sativus L.*	A00JM	Cucumbers	Spain	Spain				edible part		16					24
Cucumber	*Cucumis sativus L.*	A00JM	Cucumbers	Italy	Italy						459–840		nd			25
Cucumber	*Cucumis sativus L.*	A00JM	Cucumbers	Spain	Spain				edible part	green	16					24
Cucumber	*Cucumis sativus L.*	A00JM#F20.A07QF	Cucumbers, PART-CONSUMED-ANALYSED = W/o peel	Spain	Spain	96			Pulp+ seeds without skin	white	16±1					24
Gac fruit	*Momordica cochinchinensis Spreng.*	A16QK	Gac	Australia		72-89			the aril around the seeds	orange			0.2-4.5			150
Summer squash	*Cucurbita pepo L.*	A00JS	Summer squashes	Germany	Germany	95.2			edible part		1330			10		53
Summer squash	*Cucurbita pepo L.*	A00JS	Summer squashes	Spain	Spain				edible part	green/ green+ white	169					24
Summer squash	*Cucurbita pepo L.*	A00JS	Summer squashes	Spain	Spain				edible part	green/ green+ white	108					24
Summer squash	*Curcubita pepo L.*	A00JS	Summer squashes	Spain	Spain				edible part	white	169					24
Summer squash	*Curcubita pepo L.*	A00JS	Summer squashes	Spain	Spain				edible part	white	108					24
4Summer squash	*Curcubita pepo, L. var. medullusa, Alef.*	A00JS	Summer squashes	Spain	Spain	95			pulp+ seeds	orange	108±13					24
Summer squash	*Curcubita pepo, L. var. medullusa, Alef.*	A00JS	Summer squashes	Spain	Spain	95			pulp+ seeds	orange	169±10					24

Table S9.26.5 Cucurbits with edible peel (A00JL) (µg/100g) (continuation)

Food name	Scientific name	FoodEx2_TermCode	FoodEx2_TermName	Origin (country)	Purchase (country)	Water (%)	Process	Saponification	Part analysed	Colour	Phytofluene	Violaxanthin	Z(v. cis)-lycopene	Z(v. cis)-β-carotene	Z(v. cis)-β-cryptoxanthin	Zeaxanthin	Ref.
Cucumber	*Cucumis sativus*	A00JM	Cucumbers	USA				no						15			28
Summer squash	*Cucurbita pepo*	A00JS	Summer squashes	Germany	Germany	95.2			edible part			150					53
Summer squash	*Cucurbita pepo*	A00JS#F28.A0BA1$F20.A07QE	Summer squash, PROCESS = Cooking and similar thermal preparation processes, PART-CONSUMED-ANALYSED = With peel	USA			cooked	no	with skin					46			28

Table S9.27.1 Cucurbits with inedible peel (A00KD) (µg/100g)

Food name	Scientific name	FoodEx2_TermCode	FoodEx2_TermName	Origin (country)	Purchase (country)	Water (%)	Process	Saponification	Part analysed	Colour	α-carotene	β-carotene	β-cryptoxanthin	ζ-carotene	Antheraxanthin	Ref.
Butternut squash	*Cucurbita moschata 'Butternut'*	A0DLT#F28.A0BA1	Butternut squashes, PROCESS = Cooking and similar thermal preparation processes	USA			cooked	no			183		0			28
Butternut squash	*Cucurbita moschata Duch.*	A0DLT	Butternut squashes	Italy	Italy				pulp	0.50-0.67 in Hunter scale as a/b	5250-7990	7710-11010				151

Table S9.27.1 Cucurbits with inedible peel (A00KD) (µg/100g) (continuation)

Food name	Scientific name	FoodEx2_ TermCode	FoodEx2_ TermName	Origin (country)	Purchase (country)	Water (%)	Process	Saponification	Part analysed	Colour	α-carotene	β-carotene	β-cryptoxanthin	ζ-carotene	Antheraxanthin	Ref.
Melon	*Cucumis melo L. var. cantalupensis*	A00KF	Melons	USA			raw	no			60		5			28
Melon	*Cucumis melo L.*	A00KF	Melons	Spain	Spain				edible part	yellow		21				24
Melon	*Cucumis melo L.*	A00KF	Melons	Spain	Spain				edible part			21				24
Melon	*Cucumis melo L.*	A00KF	Melons	Spain	Spain	88			without skin or seeds	white		21±5				24
Melon	*Cucumis melo L. (Inodorus Group)*	A00KF	Melons	USA				no			0		0			28
Melon	*Cucumis melo L. var. Amarelo*	A00KF	Melons	Brazil					edible part	white / yellow	nd	10	nd			109
Pumpkin	*Cucurbita maxima*	A00KH	Pumpkins	Spain	Spain				edible part			490	60			24
Pumpkin	*Cucurbita maxima*	A00KH	Pumpkins	Spain	Spain				edible part		31	188				24
Pumpkin	*Cucurbita maxima*	A00KH	Pumpkins	Spain	Spain				edible part		53	692				24
Pumpkin	*Cucurbita maxima var. Autumn Cup*	A00KH	Pumpkins	Austria	Austria				flesh fruit		800	5200				152
Pumpkin	*Cucurbita maxima var. Buen Gusto*	A00KH	Pumpkins	Austria	Austria				flesh fruit		1000	3300				152
Pumpkin	*Cucurbita maxima var. Flat white Boer*	A00KH	Pumpkins	Austria	Austria				flesh fruit		7500	6200				152
Pumpkin	*Cucurbita maxima var. Gelber Zentner*	A00KH	Pumpkins	Austria	Austria				flesh fruit		0	2200				152
Pumpkin	*Cucurbita maxima var. Hyvita*	A00KH	Pumpkins	Austria	Austria				flesh fruit		990	2500				152

Table S9.27.1 Cucurbits with inedible peel (A00KD) (µg/100g) (continuation)

Food name	Scientific name	FoodEx2_ TermCode	FoodEx2_ TermName	Origin (country)	Purchase (country)	Water (%)	Process	Saponification	Part analysed	Colour	α-carotene	β-carotene	β-cryptoxanthin	ζ-carotene	Antheraxanthin	Ref.
Pumpkin	*Cucurbita maxima var. Imperial Elite*	A00KH	Pumpkins	Austria	Austria				flesh fruit		1100	7400				152
Pumpkin	*Cucurbita maxima var. Japan 117*	A00KH	Pumpkins	Austria	Austria				flesh fruit		1000	7200				152
Pumpkin	*Cucurbita maxima var. Mini green Hubbard*	A00KH	Pumpkins	Austria	Austria				flesh fruit		420	1400				152
Pumpkin	*Cucurbita maxima* **var.** ***Mishti kumra***	A00KH	Pumpkins	Bangladesh					fruit			362±89.3				257
Pumpkin	*Cucurbita maxima var. Snow Delite*	A00KH	Pumpkins	Austria	Austria				flesh fruit		1500	6400				152
Pumpkin	*Cucurbita maxima var. Uchiki Kuri*	A00KH	Pumpkins	Austria	Austria				flesh fruit		1400	2500				152
Pumpkin	*Cucurbita maxima var. Umber Cup*	A00KH	Pumpkins	Austria	Austria				flesh fruit		790	3700				152
Pumpkin	*Cucurbita maxima var. Walfish*	A00KH	Pumpkins	Austria	Austria				flesh fruit		900	4300				152
Pumpkin	*Cucurbita maxima x C. moschata var. Tetsuka Buto*	A00KH	Pumpkins	Austria	Austria				flesh fruit		2400	3500				152
Pumpkin	*Cucurbita moschata Duchesne cv. Menina Brasileira and cv. Goianinha*	A0DLT#F20.A07QF$F20.A07RD	Butternut squashes, PART-CONSUMED-ANALYSED = W/o peel, W/o seeds	Brazil					without skin and seed	orange	2530	6170	nd			109
Pumpkin	*Cucurbita moschata var. Burpee Butterbush*	A0DLT	Butternut squashes	Austria	Austria				flesh fruit		980	3100				152

Table S9.27.1 Cucurbits with inedible peel (A00KD) (µg/100g) (continuation)

Food name	Scientific name	FoodEx2_ TermCode	FoodEx2_ TermName	Origin (country)	Purchase (country)	Water (%)	Process	Saponification	Part analysed	Colour	α-carotene	β-carotene	β-cryptoxanthin	ζ-carotene	Antheraxanthin	Ref.
Pumpkin	*Cucurbita moschata var. Long Island Cheese*	A0DLT	Butternut squashes	Austria	Austria				flesh fruit		5900	7000				152
Pumpkin	*Cucurbita moschata var. Martinica*	A0DLT	Butternut squashes	Austria	Austria				flesh fruit		1600	5400				152
Pumpkin	*Cucurbita moschata var. Mousquée de Provence*	A0DLT	Butternut squashes	Austria	Austria				flesh fruit		2800	4900				152
Pumpkin	*Cucurbita pepo*	A00KH	Pumpkins	Italy	Italy							490	60			25
Pumpkin	*Cucurbita pepo L.*	A00KH	Pumpkins	Poland	Poland	8.2			seed and oil	yellow-green	10 -- 20	80-210	20-50			153
Pumpkin	*Cucurbita pepo var. Acorn Table*	A00KH	Pumpkins	Austria	Austria				flesh fruit		150	2100				152
Pumpkin	*Cucurbita pepo var. Acorn Tay Bell*	A00KH	Pumpkins	Austria	Austria				flesh fruit		150	2100				152
Pumpkin	*Cucurbita pepo var. Carneval di Venezia*	A00KH	Pumpkins	Austria	Austria				flesh fruit		30	60				152
Pumpkin	*Cucurbita pepo var. Melonette Jaspée Vende*	A00KH	Pumpkins	Austria	Austria				flesh fruit		50	1300				152
Pumpkin	*Cucurbita pepo var. Tonda padana (Americano)*	A00KH	Pumpkins	Austria	Austria				flesh fruit		120	2300				152

Table S9.27.1 Cucurbits with inedible peel (A00KD) (µg/100g) (continuation)

Food name	Scientific name	FoodEx2_ TermCode	FoodEx2_ TermName	Origin (country)	Purchase (country)	Water (%)	Process	Saponification	Part analysed	Colour	α-carotene	β-carotene	β-cryptoxanthin	ζ-carotene	Antheraxanthin	Ref.
Pumpkin	*Cucurbita pepo var. turbinata*	A00KH#F20.A07QF	Pumpkins, PART-CONSUMED-ANALYSED = W/o peel	USA				no	without skin		0		0			28
Pumpkin	*Cucurbita pepo 'yellow crookneck'*	A00KH#F28.A0BA1$F10.A0F5H	Pumpkins, PROCESS = Cooking and similar thermal preparation processes, QUALITATIVE-INFO = yellow	USA			cooked	no		yellow	0		0			28
Pumpkin	*Curcubita maxima*	A00KH	Pumpkins	Spain	Spain				edible part	orange		490	60			24
Pumpkin	*Curcubita maxima*	A00KH	Pumpkins	Spain	Spain				edible part	orange	31	188				24
Pumpkin	*Curcubita maxima*	A00KH	Pumpkins	Spain	Spain				edible part	orange	53	692				24
Watermellon	*Citrullus lanatus (Thunb) Matsumura & Nakai*	A00KJ	Watermelons	Italy	Italy					red		46-546				154
Watermelon	*Citrullus lanatus*	A00KJ	Watermelons	Spain	Spain				edible part	red		77	62			24
Watermelon	*Citrullus lanatus*	A00KJ	Watermelons	Spain	Spain				edible part			77	62			24
Watermelon	*Citrullus lanatus*	A00KJ	Watermelons	USA				no			0		5			28
Watermelon	*Citrullus lanatus (Thunb.) Matsum & Nakai*	A00KJ	Watermelons	Brazil						red	nd	365	nd			109
Watermelon	*Citrullus vulgaris*	A00KJ#F10.A0F2S	WatermelonsQUALITATIVE-INFO = red	Indonesia					fruit	red		592 (314-777)	nd			59
Watermelon	*Citrullus vulgaris*	A00KJ#F10.A0F5H	Watermelons, QUALITATIVE-INFO = yellow	Indonesia					fruit	yellow		140 (56-287)	90 (59-110)			59

Table S9.27.1 Cucurbits with inedible peel (A00KD) (µg/100g) (continuation)

Food name	Scientific name	FoodEx2_ TermCode	FoodEx2_ TermName	Origin (country)	Purchase (country)	Water (%)	Process	Saponification	Part analysed	Colour	α-carotene	β-carotene	β-cryptoxanthin	ζ-carotene	Antheraxanthin	Ref.
Watermelon	*Citrullus vulgaris*	A00KJ#F10.A0F2S	Watermelons QUALITATIVE-INFO = red	Italy	Italy						nd	314–777	nd			25
Watermelon	*Citrullus vulgaris*	A00KJ#F10.A0F5H	Watermelons, QUALITATIVE-INFO = yellow	Italy	Italy						nd	56–287	59–110			25
Watermelon	*Citrullus vulgaris, Schered.*	A00KJ#F20.A07QF$F20.A07RD	Watermelons, PART-CONSUMED-ANALYSED = W/o peel, W/o seeds	Spain	Spain	92			without rind or seeds	red		77±29	62±20			24
Watermelon	*Citrullus vulgaris, Schrad*	A00KJ	Watermelons	Spain	Spain				fruit	red		57.6	1.2			56
Watermelon	*Citrullus vulgaris, Schrad*	A00KJ	Watermelons	Spain	Spain				edible part	red			1.2±0.5			56

Table S9.27.2 Cucurbits with inedible peel (A00KD) (µg/100g) (continuation)

Food name	Scientific name	FoodEx2_ TermCode	FoodEx2_ TermName	Origin (country)	Purchase (country)	Water (%)	Process	Saponification	Part analysed	Colour	Capsanthin	Capsorubin	Cucurbitaxanthin	E(v. trans)-α-carotene	E(v. trans)-β-carotene	Ref.
Butternut squash	*Cucurbita moschata 'Butternut'*	A0DLT#F28.A0BA1	Butternut squashes, PROCESS = Cooking and similar thermal preparation processes	USA			cooked	no							569	28
Melon	*Cucumis melo L. (Inodorus Group)*	A00KF	Melons	USA				no							18	28

Table S9.27.2 Cucurbits with inedible peel (A00KD) (µg/100g) (continuation)

Food name	Scientific name	FoodEx2_ TermCode	FoodEx2_ TermName	Origin (country)	Purchase (country)	Water (%)	Process	Saponification	Part analysed	Colour	Capsanthin	Capsorubin	Cucurbitaxanthin	E(v. trans)-α-carotene	E(v. trans)-β-carotene	Ref.
Melon	*Cucumis melo var. cantalupensis*	A00KF	Melons	USA			raw	no							2063	28
Pumpkin	*Cucurbita pepo var. turbinata*	A00KH#F20.A07QF	Pumpkins, PART-CONSUMED-ANALYSED = W/o peel	USA			raw	no	no skin						224	28
Pumpkin	*Cucurbita pepo 'yellow crookneck'*	A00KH#F28.A0BA1$F10.A0F5H	Pumpkins, PROCESS = Cooking and similar thermal preparation processes, QUALITATIVE-INFO = yellow	USA			cooked	no		yellow					116	28
Pumpkin	*Curcubita pepo L. var. styriaca Greb.*	A00KH	Pumpkins	Portugal	Portugal				edible part	orange				44±12	186±58	57
Pumpkin	*Curcubita pepo L. var. styriaca Greb.*	A00KH	Pumpkins	Portugal	Portugal				edible part	orange				65.17± 0.75	269±33	57
Pumpkin	*Curcubita pepo L. var. styriaca Greb.*	A00KH	Pumpkins	Portugal	Portugal				edible part	orange				56.4±7.7	232.47± 0.64	57
Watermelon	*Citrullus lanatus*	A00KJ	Watermelons	USA				no							126	28
Watermelon	*Citrullus vulgaris, Schrad*	A00KJ	Watermelons	Spain	Spain				fruit	red					57.6	56
Watermelon	*Citrulluss vulgaris, Schrad*	A00KJ	Watermelons	Spain	Spain				edible part	red					57.6±4.8	56

Table S9.27.3 Cucurbits with inedible peel (A00KD) (µg/100g) (continuation)

Food name	Scientific name	FoodEx2_ TermCode	FoodEx2_ TermName	Origin (country)	Purchase (country)	Water (%)	Process	Saponification	Part analysed	Colour	E(v. trans)-β-cryptoxanthin	E(v. trans)-lutein	E(v. trans)-lycopene	E(v. trans)-zeaxanthin	Lactucaxanthin	Ref.
Butternut Squash	*Cucurbita moschata 'Butternut'*	A0DLT#F28.A0BA1	Butternut squashes, PROCESS = Cooking and similar thermal preparation processes	USA			cooked	no				57		0		28
Melon	*Cucumis melo L. (Inodorus Group)*	A00KF	Melons	USA				no				25		0		28
Melon	*Cucumis melo var. cantalupensis*	A00KF	Melons	USA			raw	no				19		0		28
Pumpkin	*Cucurbita pepo var. turbinata*	A00KH#F20.A07QF	Pumpkins, PART-CONSUMED-ANALYSED = W/o peel	USA			raw	no	no skin			47		0		28
Pumpkin	*Cucurbita pepo 'yellow crookneck'*	A00KH#F28.A0BA1$F10.A0F5H	Pumpkins, PROCESS = Cooking and similar thermal preparation processes, QUALITATIVE-INFO = yellow	USA			cooked	no		yellow		150		0		28
Pumpkin	*Curcubita pepo L. var. styriaca Greb.*	A00KH	Pumpkins	Portugal	Portugal				edible part	orange		76±13				57
Pumpkin	*Curcubita pepo L. var. styriaca Greb.*	A00KH	Pumpkins	Portugal	Portugal				edible part	orange		49±15				57
Watermelon	*Citrullus lanatus*	A00KJ	Watermelons	USA				no				4		0		28
Watermelon	*Citrullus vulgaris*	A00KJ	Watermelons	Finland	Finland				pulp	-			3080			26

Table S9.27.4 Cucurbits with inedible peel (A00KD) (µg/100g) (continuation)

Food name	Scientific name	FoodEx2_ TermCode	FoodEx2_ TermName	Origin (country)	Purchase (country)	Water (%)	Process	Saponification	Part analysed	Colour	Lutein	Luteoxanthin	Lycopene	Neoxanthin	Phytoene	Ref.
Butternut squash	*Cucurbita moschata Duch.*	A0DLT	Butternut squashes	Italy	Italy				squash pulp	0.50-0.67 in Hunter scale as a/b	610-3080					151
Melon	*Cucumis melo*	A00KF	Melons	Spain	Spain				edible part		2					24
Melon	*Cucumis melo L. var. Amarelo*	A00KF	Melons	Brazil					fruit, edible part	white / yellow	tr.		nd	nd		109
Melon	*Cucumis melo, L.*	A00KF	Melons	Spain	Spain				edible part	yellow	2					24
Melon	*Cucumis melo, L.*	A00KF	Melons	Spain	Spain				fruit	green					40	22
Melon	*Cucumis melo, L.*	A00KF	Melons	Spain	Spain				pulp	yellow					250	22
Melon	*Cucumis melo, L.*	A00KF	Melons	Spain	Spain	88			without skin or seeds	white	2±0,5					24
Pumpkin	*Cucurbita maxima*	A00KH	Pumpkins	Spain	Spain				edible part		728					24
Pumpkin	*Cucurbita moschata Duchesne cv. Menina Brasileira and cv. Goianinha*	A0DLT#F20.A07QF$F20.A07RD	Butternut squashes, PART-CONSUMED-ANALYSED = W/o peel, W/o seeds	Brazil					pulp without skin and seed	orange	1785		nd	705		109
Pumpkin	*Cucurbita pepo*	A00KH	Pumpkins	Italy	Italy						630		500			25
Pumpkin	*Cucurbita pepo L.*	A00KH	Pumpkins	Poland	Poland	8.2			seed and oil	yellow-green	100-270					153
Pumpkin	*Curcubita maxima*	A00KH	Pumpkins	Spain	Spain				edible part	orange	623					24
Pumpkin	*Curcubita maxima*	A00KH	Pumpkins	Spain	Spain				edible part	orange	728					24

Table S9.27.4 Cucurbits with inedible peel (A00KD) (µg/100g) (continuation)

Food name	Scientific name	FoodEx2_ TermCode	FoodEx2_ TermName	Origin (country)	Purchase (country)	Water (%)	Process	Saponification	Part analysed	Colour	Lutein	Luteoxanthin	Lycopene	Neoxanthin	Phytoene	Ref.
Watermellon	*Citrullus lanatus (Thunb) Matsumura & Nakai*	A00KJ	Watermelons	Italy	Italy					red			959-1681			154
Watermelon	*Citrullus lanatus*	A00KJ	Watermelons	Spain	Spain				edible part	red	40		2454			24
Watermelon	*Citrullus lanatus*	A00KJ	Watermelons	Spain	Spain				edible part		40		2454			24
Watermelon	*Citrullus lanatus*	A00KJ	Watermelons	Spain	Spain				Pulp	red					1170	22
Watermelon	*Citrullus lanatus (Thunb.) Matsum & Nakai*	A00KJ	Watermelons	Brazil						red	nd		3550	nd		109
Watermelon	*Citrullus vulgaris*	A00KJ#F10.A0F2S	Watermelons, QUALITATIVE-INFO = red	Indonesia		na			fruit	red			11389 (8731-13523)			59
Watermelon	*Citrullus vulgaris*	A00KJ#F10.A0F5H	Watermelons, QUALITATIVE-INFO = yellow	Indonesia		na			fruit	yellow			71 (nd - 109)			59
Watermelon	*Citrullus vulgaris*	A00KJ#F10.A0F2S	Watermelons, QUALITATIVE-INFO = red	Italy	Italy					red			4770–13523			25
Watermelon	*Citrullus vulgaris*	A00KJ#F10.A0F5H	Watermelons, QUALITATIVE-INFO = yellow	Italy	Italy					yellow			nd – 109			25
Watermelon	*Citrullus vulgaris, Schered.*	A00KJ#F20.A07QF$F20.A07RD	Watermelons, PART-CONSUMED-ANALYSED = W/o peel, W/o seeds	Spain	Spain	92			without rind or seeds	red	40±13		2454± 319		1122± 812	24

Table S9.27.5 Cucurbits with inedible peel (A00KD) (µg/100g) (continuation)

Food name	Scientific name	FoodEx2_ TermCode	FoodEx2_ TermName	Origin (country)	Purchase (country)	Water (%)	Process	Saponification	Part analysed	Colour	Phytofluene	Violaxanthin	Z(v. cis)-lycopene	Z(v. cis)-β-carotene	Z(v. cis)-β-cryptoxanthin	Zeaxanthin	Ref.
Butternut squash	*Cucurbita moschata 'Butternut'*	A0DLT#F28.A0BA1	Butternut squashes, PROCESS = Cooking and similar thermal preparation processes	USA	USA		cooked	no						38			28
Melon	*Cucumis melo L. (Inodorus Group)*	A00KF	Melons	USA	USA			no						3			28
Melon	*Cucumis melo L. var. Amarelo*	A00KF	Melons	Brazil					fruit, edible part	white / yellow		tr.				nd	109
Melon	*Cucumis melo var. cantalupensis*	A00KF	Melons	USA	USA		raw	no						25			28
Melon	*Cucumis melo, L.*	A00KF	Melons	Spain	Spain				edible part	yellow						tr.	24
Melon	*Cucumis melo, L.*	A00KF	Melons	Spain	Spain				Fruit	green	40						22
Melon	*Cucumis melo, L.*	A00KF	Melons	Spain	Spain	88			without skin or seeds	white						tr.	24
Pumpkin	*Cucurbita moschata Duchesne cv. Menina Brasileira and cv. Goianinha*	A0DLT#F20.A07QF$F20.A07RD	Butternut squashes, PART-CONSUMED-ANALYSED = W/o peel, PART-CONSUMED-ANALYSED = W/o seeds	Brazil	Brazil				pulp without skin and seed	orange		tr.				nd	109
Pumpkin	*Cucurbita pepo var. turbinata*	A00KH#F20.A07QF	Pumpkins, PART-CONSUMED-ANALYSED = W/o peel	USA			raw	no	no skin					16			28

Table S9.27.5 Cucurbits with inedible peel (A00KD) (µg/100g) (continuation)

Food name	Scientific name	FoodEx2_ TermCode	FoodEx2_ TermName	Origin (country)	Purchase (country)	Water (%)	Process	Saponification	Part analysed	Colour	Phytofluene	Violaxanthin	Z(v. cis)-lycopene	Z(v. cis)-β-carotene	Z(v. cis)-β-cryptoxanthin	Zeaxanthin	Ref.
Pumpkin	*Cucurbita pepo 'yellow crookneck'*	A00KH#F28.A0BA1$F10.A0F5H	Pumpkins, PROCESS = Cooking and similar thermal preparation processes, QUALITATIVE-INFO = yellow	USA			cooked	no						0			28
Watermelon	*Citrullus lanatus*	A00KJ	Watermelons	Spain	Spain				Pulp	red	440						22
Watermelon	*Citrullus lanatus*	A00KJ	Watermelons	USA				no						0			28
Watermelon	*Citrullus lanatus (Thunb.) Matsum & Nakai*	A00KJ	Watermelons	Brazil						red		nd				nd	109

Table S9.28.1 Sweet corn and similar (A0DLP) (µg/100g)

Food name	Scientific name	FoodEx2_ TermCode	FoodEx2_ TermName	Origin (country)	Purchase (country)	Water (%)	Process	Saponification	Part analysed	Colour	α-carotene	β-carotene	β-cryptoxanthin	ζ-carotene	Antheraxanthin	Ref.
Sweet corn	*Zea mays L. convar. saccharata var. rugosa*	A00KP	Sweet corn	USA					kernels		10±2	39±7	34±6			155
Sweet corn	*Zea mays L. convar. saccharata var. rugosa*	A00KP	Sweet corn	USA					kernels		26±1	797±35	116±6			155
Sweet corn	*Zea mays L. convar. saccharata var. rugosa*	A00KP	Sweet corn	USA					kernels		3±0	14±2	8±1			155
Sweet corn	*Zea mays L. convar. saccharata var. rugosa*	A00KP	Sweet corn	USA					kernels		9±1	41±15	62±5			155
Sweet corn	*Zea mays L. convar. saccharata var. rugosa*	A00KP	Sweet corn	USA					kernels		14±9	146±108	29±4			155

Table S9.28.2 Sweet corn and similar (A0DLP) (µg/100g) (continuation)

Food name	Scientific name	FoodEx2_ TermCode	FoodEx2_ TermName	Origin (country)	Purchase (country)	Water (%)	Process	Saponification	Part analysed	Colour	Lutein	Luteoxanthin	Lycopene	Neoxanthin	Phytoene	Ref.
Sweet corn	*Zea mays L. convar. saccharata var. rugosa*	A00KP	Sweet corn	USA					kernels		1451± 156					155
Sweet corn	*Zea mays L. convar. saccharata var. rugosa*	A00KP	Sweet corn	USA					kernels		200±11					155
Sweet corn	*Zea mays L. convar. saccharata var. rugosa*	A00KP	Sweet corn	USA					kernels		161±4					155
Sweet corn	*Zea mays L. convar. saccharata var. rugosa*	A00KP	Sweet corn	USA					kernels		737±288					155
Sweet corn	*Zea mays L. convar. saccharata var. rugosa*	A00KP	Sweet corn	USA					kernels		11±4					155

Table S9.28.3 Sweet corn and similar (A0DLP) (µg/100g) (continuation)

Food name	Scientific name	FoodEx2_ TermCode	FoodEx2_ TermName	Origin (country)	Purchase (country)	Water (%)	Process	Saponification	Part analysed	Colour	Phytofluene	Violaxanthin	Z(v. cis)-lycopene	Z(v. cis)-β-carotene	Z(v. cis)-β-cryptoxanthin	Zeaxanthin	Ref.
Sweet corn	*Zea mays L. convar. saccharata var. rugosa*	A00KP	Sweet corn	USA					kernels							752±17	155
Sweet corn	*Zea mays L. convar. saccharata var. rugosa*	A00KP	Sweet corn	USA					kernels							9±1	155
Sweet corn	*Zea mays L. convar. saccharata var. rugosa*	A00KP	Sweet corn	USA					kernels							161±4	155
Sweet corn	*Zea mays L. convar. saccharata var. rugosa*	A00KP	Sweet corn	USA					kernels							177±8	155
Sweet corn	*Zea mays L. convar. saccharata var. rugosa*	A00KP	Sweet corn	USA					kernels							223±82	155

Table S9.29.1 Root and tuber vegetables (excluding starchy- and sugar-) (A00QF) (µg/100g)

Food name	Scientific name	FoodEx2_ TermCode	FoodEx2_ TermName	Origin (country)	Purchase (country)	Water (%)	Process	Saponification	Part analysed	Colour	α-carotene	β-carotene	β-cryptoxanthin	ζ-carotene	Antheraxanthin	Ref.
Beet	Beta vulgaris L. var. cicla	A00QG	Beetroots	Spain	Spain	93			leaves+ stalk	purple		1095±61				24
Beet	Beta vulgaris L. var. cicla	A00QG	Beetroots	Spain	Spain	95			leaves+ stalk	purple		1360±34				24
Beet	Beta vulgaris L., 'Bulls Blood'	A00QG	Beetroots	Lithuania	Lithuania			no	leaves		5	9				110
Beet	Beta vulgaris L., 'Bulls Blood'	A00QG	Beetroots	Lithuania	Lithuania			no	leaves		3	11				110
Beet	Beta vulgaris L., 'Bulls Blood'	A00QG	Beetroots	Lithuania	Lithuania			no	leaves		9	87				110
Beet	Beta vulgaris L., 'Bulls Blood'	A00QG	Beetroots	Lithuania	Lithuania			no	leaves		3	12				110
Beet	Beta vulgaris L., 'Bulls Blood'	A00QG	Beetroots	Lithuania	Lithuania			no	leaves		3	39				110
Carrot	Daucus carota L	A00QH#F20.A 07QF	Carrots, PART-CONSUMED-ANALYSED = W/o peel	United Kingdom	United Kingdom				peeled		4450± 940	32000± 2880				95
Carrot	Daucus carota L.	A00QH	Carrots	Italy	Italy						2840–4960	4350–8840				25
Carrot	Daucus carota L.	A00QH	Carrots	Germany	Germany	91.3			edible part		4890	9020				53
Carrot	Daucus carota L.	A00QH	Carrots	Germany	Germany	84.3			edible part		3060	6500	12			53
Carrot	Daucus carota L.	A00QH	Carrots	Germany	Germany				edible part		4120	4650	28			53
Carrot	Daucus carota L.	A00QH	Carrots	Spain	Spain				edible part	orange	3245	8162				24
Carrot	Daucus carota L.	A00QH	Carrots	Spain	Spain				edible part	orange	2895	6628				24
Carrot	Daucus carota L.	A00QH	Carrots	Spain	Spain				edible part	orange	3700	9800				24
Carrot	Daucus carota L.	A00QH	Carrots	Turkey	Turkey				root	orange	1344-3011	4160-7162				156

Table S9.29.1 Root and tuber vegetables (excluding starchy- and sugar-) (A00QF) (µg/100g) (continuation)

Food name	Scientific name	FoodEx2_ TermCode	FoodEx2_ TermName	Origin (country)	Purchase (country)	Water (%)	Process	Saponification	Part analysed	Colour	α-carotene	β-carotene	β-cryptoxanthin	ζ-carotene	Antheraxanthin	Ref.
Carrot	Daucus carota L.	A00QH	Carrots	Spain	Spain				edible part		3245	8162				24
Carrot	Daucus carota L.	A00QH	Carrots	Spain	Spain				edible part		2895	6628				24
Carrot	Daucus carota L.	A00QH	Carrots	Italy	Italy				all sample	L*52.2±0.7a*24.1±1.5b*36.1±1	82100±1100	128400±800				111
Carrot	Daucus carota L.	A00QH	Carrots	Italy	Italy				all sample	L*52.1±0.8a*22.6±1.1b*35.7±1.9	85600±2400	101600±700				111
Carrot	Daucus carota L.	A00QH	Carrots	Italy	Italy				all sample	L*50.2±1.1a*21.4±1.4b*35.3±1.5	68100±9100	113000±16700				111
Carrot	Daucus carota L.	A00QH	Carrots	Poland	Poland				roots			4820-9520				157
Carrot	Daucus carota L. cv Nerac	A00QH	Carrots	Ireland	Ireland				roots			188000±5000				158
Carrot	Daucus carota L. cv. Nantes	A00QH	Carrots	Brazil					tuber	orange	3500	6150	nd			109
Carrot	Daucus carota L. HCM	A00QH	Carrots	France	France				root	dark-orange	7583± 619	17206±643				159
Carrot	Daucus carota L. subsp. sativus	A00QH	Carrots	Finland	Finland						2200-4900	4600-10300				86
Carrot	Daucus carota L. subsp. sativus	A00QH	Carrots	Spain	Spain						2900±300	6600±0				86
Carrot	Daucus carota L. subsp. sativus	A00QH	Carrots	United Kingdom	United Kingdom						2700-3600	8500-10800				86
Carrot	Daucus carota L. subsp. sativus	A00QH	Carrots	USA							3900	5600				86

Table S9.29.1 Root and tuber vegetables (excluding starchy- and sugar-) (A00QF) (µg/100g) (continuation)

Food name	Scientific name	FoodEx2_ TermCode	FoodEx2_ TermName	Origin (country)	Purchase (country)	Water (%)	Process	Saponification	Part analysed	Colour	α-carotene	β-carotene	β-cryptoxanthin	ζ-carotene	Antheraxanthin	Ref.
Carrot	Daucus carota L. subsp. sativus	A00QH	Carrots	Egypt							3400	6300				86
Carrot	Daucus carota L. subsp. sativus	A00QH	Carrots	Taiwan							2800± 300	5400± 600				86
Carrot	Daucus carota L. subsp. sativus	A00QH	Carrots	Malaysia							3400	6800				86
Carrot	Daucus carota L. var. Commercial French	A00QH	Carrots	France	France				root	orange	2322± 233	5404± 305				159
Carrot	Daucus carota L. var. Blanche à collet vert	A00QH	Carrots	France	France				root	white	nd	nd				159
Carrot	Daucus carota L. var. Blanche des vosges	A00QH	Carrots	France	France				root	white	nd	nd				159
Carrot	Daucus carota L. var. Carentan	A00QH	Carrots	France	France				root	orange	1644±50	5932± 360				159
Carrot	Daucus carota L. var. Commercial French	A00QH	Carrots	France	France				root	orange	1972± 183	5433± 462				159
Carrot	Daucus carota L. var. Commercial French	A00QH	Carrots	France	France				root	orange	3131± 263	6633± 564				159
Carrot	Daucus carota L. var. Commercial French	A00QH	Carrots	France	France				root	orange	1419±99	4149± 112				159

Table S9.29.1 Root and tuber vegetables (excluding starchy- and sugar-) (A00QF) (µg/100g) (continuation)

Food name	Scientific name	FoodEx2_ TermCode	FoodEx2_ TermName	Origin (country)	Purchase (country)	Water (%)	Process	Saponification	Part analysed	Colour	α-carotene	β-carotene	β-cryptoxanthin	ζ-carotene	Antheraxanthin	Ref.
Carrot	Daucus carota L. var. Commercial French	A00QH	Carrots	France	France				root	orange	2291± 224	6190± 403				159
Carrot	Daucus carota L. var. Commercial French	A00QH	Carrots	France	France				root	orange	1916± 138	4730± 319				159
Carrot	Daucus carota L. var. Kokubu	A00QH	Carrots	France	France				root	orange	1748±29	3740±25				159
Carrot	Daucus carota L. var. La Merveille	A00QH	Carrots	France	France				root	orange	2092±36	5869± 101				159
Carrot	Daucus carota L. var. Nantaise améliorée	A00QH	Carrots	France	France				root	orange	1369± 150	3625± 329				159
Carrot	Daucus carota L. var. San NaÏ	A00QH	Carrots	France	France				root	orange	1333±85	3206± 182				159
Carrot	Daucus carota L. var. sativa, D.C.	A00QH	Carrots	Spain	Spain	88			root	orange	2895± 276	6628±45				24
Carrot	Daucus carota L. var. sativa, D.C.	A00QH	Carrots	Spain	Spain	90			root	orange	3245± 128	8162± 364				24
Carrot	Daucus carota L. var. Violette jordanienne	A00QH	Carrots	France	France				root	purple	nd	381±24				159
Carrot	Daucus carota L. var. Violette turque	A00QH	Carrots	France	France				root	purple	nd	318±18				159
Carrot	Daucus carota L. var. yellowstone	A00QH	Carrots	France	France				root	yellow	nd	332±15				159

Table S9.29.1 Root and tuber vegetables (excluding starchy- and sugar-) (A00QF) (µg/100g) (continuation)

Food name	Scientific name	FoodEx2_ TermCode	FoodEx2_ TermName	Origin (country)	Purchase (country)	Water (%)	Process	Saponification	Part analysed	Colour	α-carotene	β-carotene	β-cryptoxanthin	ζ-carotene	Antheraxanthin	Ref.
Carrot	Daucus carota L., var. Bolero	A00QH	Carrots	Turkey	Turkey				root		1642± 101 3011±217	4826± 465 7162±503				160
Carrot	Daucus carota L., var. Fontana	A00QH	Carrots	Norway	Norway				root		5490-11270	2210-5510				161
Carrot	Daucus carota L., var. Maestro-F1	A00QH	Carrots	Turkey	Turkey				root		1706±60 - 2478± 179	4160±148 - 6706±42				160
Carrot	Daucus carota L., var. Merida	A00QH	Carrots	Norway	Norway				root		5550-10200	1980-5220				161
Carrot	Daucus carota L., var. Nanco	A00QH	Carrots	Turkey	Turkey				root		1473±34 - 2630±33	4310± 118 - 6440± 146				160
Carrot	Daucus carota L., var. Nandrin	A00QH	Carrots	Norway	Norway				root		5630-12080	1910-4530				161
Carrot	Daucus carota L., var. Nantindo	A00QH	Carrots	Turkey	Turkey				root		1665±113 - 3006±66	4767±123 - 6637±58				160
Carrot	Daucus carota L., var. Newburg	A00QH	Carrots	Norway	Norway				root		6100-10800	2370-4090				161
Carrot	Daucus carota L., var. Presto-F1	A00QH	Carrots	Turkey	Turkey				root		2346±45 - 2367±217	5185±468 6198±138				160
Carrot	Daucus carota L., var. PS-F1	A00QH	Carrots	Turkey	Turkey				root		2688±225 - 2967±36	6168±302 6373±476				160
Carrot	Daucus carota L., var. Tito	A00QH	Carrots	Turkey	Turkey				root		1344±17 - 1718±153	4563±311 4835±473				160
Carrot	Daucus carota var. Jaune obtuse du Doubs	A00QH	Carrots	France	France				root	yellow	nd	332±21				159

Table S9.29.1 Root and tuber vegetables (excluding starchy- and sugar-) (A00QF) (µg/100g) (continuation)

Food name	Scientific name	FoodEx2_ TermCode	FoodEx2_ TermName	Origin (country)	Purchase (country)	Water (%)	Process	Saponification	Part analysed	Colour	α-carotene	β-carotene	β-cryptoxanthin	ζ-carotene	Antheraxanthin	Ref.
Carrot	Daucus carota var. New Kuroda	A00QH	Carrots	France	France				root	orange	1635±17	3632±64				159
Carrot	Daucus carota L. var. De Guérande	A00QH	Carrots	France	France				root	orange	1278± 234	3354±457				159
Turnip	Brassica napus	A00RE	Turnips	Spain	Spain				edible part	white		4575				24
Turnip	Brassica napus	A00RE	Turnips	Spain	Spain				edible part	white		72				24
Turnip	Brassica napus	A00RE	Turnips	Spain	Spain				edible part			4575				24
Turnip	Brassica rapa L.	A00RE	Turnips	Spain	Spain				edible part			72				24

Table S9.29.2 Root and tuber vegetables (excluding starchy- and sugar-) (A00QF) (µg/100g) (continuation)

Food name	Scientific name	FoodEx2_ TermCode	FoodEx2_ TermName	Origin (country)	Purchase (country)	Water (%)	Process	Saponification	Part analysed	Colour	Lutein	Luteoxanthin	Lycopene	Neoxanthin	Phytoene	Ref.
Beet	*Beta vulgaris L. var. cicla*	A00QG	Beetroots	Spain	Spain	93			leaves+ stalk	purple	1503±15					24
Beet	*Beta vulgaris L. var. cicla*	A00QG	Beetroots	Spain	Spain	95			leaves+ stalk	purple	1960±85					24
Beet	*Beta vulgaris L., 'Bulls Blood'*	A00QG	Beetroots	Lithuania	Lithuania			no	leaves		10385			116		110
Beet	*Beta vulgaris L., 'Bulls Blood'*	A00QG	Beetroots	Lithuania	Lithuania			no	leaves		8778			124		110
Beet	*Beta vulgaris L., 'Bulls Blood'*	A00QG	Beetroots	Lithuania	Lithuania			no	leaves		11852			134		110

Table S9.29.2 Root and tuber vegetables (excluding starchy- and sugar-) (A00QF) (µg/100g) (continuation)

Food name	Scientific name	FoodEx2_ TermCode	FoodEx2_ TermName	Origin (country)	Purchase (country)	Water (%)	Process	Saponification	Part analysed	Colour	Lutein	Luteoxanthin	Lycopene	Neoxanthin	Phytoene	Ref.
Beet	*Beta vulgaris L., 'Bulls Blood'*	A00QG	Beetroots	Lithuania	Lithuania			no	leaves		10173			108		110
Beet	*Beta vulgaris L., 'Bulls Blood'*	A00QG	Beetroots	Lithuania	Lithuania			no	leaves		11066			96		110
Carrot	*Daucus carota L. var. Nantaise améliorée*	A00QH	Carrots	France	France				root	orange	61±8					110
Carrot	*Daucus carota L*	A00QH#F20.A07QF	Carrots, PART-CONSUMED-ANALYSED = W/o peel	United Kingdom	United Kingdom				root (peeled)		350±60				3650± 1400	220
Carrot	*Daucus carota L.*	A00QH	Carrots	Italy	Italy						254–510					25
Carrot	*Daucus carota L.*	A00QH	Carrots	Germany	Germany	91.3			edible part		360					53
Carrot	*Daucus carota L.*	A00QH	Carrots	Germany	Germany	84.3			edible part		560					53
Carrot	*Daucus carota L.*	A00QH	Carrots	Germany	Germany				edible part		440		15			53
Carrot	*Daucus carota L.*	A00QH	Carrots	Spain	Spain				edible part	orange	273					24
Carrot	*Daucus carota L.*	A00QH	Carrots	Spain	Spain				edible part	orange	288					24
Carrot	*Daucus carota L.*	A00QH	Carrots	Turkey	Turkey				root	orange	2-144					156
Carrot	*Daucus carota L.*	A00QH	Carrots	Turkey	Turkey				roots		36±4 - 62±14					160
Carrot	*Daucus carota L.*	A00QH	Carrots	Spain	Spain				edible part		273					24
Carrot	*Daucus carota L.*	A00QH	Carrots	Spain	Spain				edible part		288					24
Carrot	*Daucus carota L.*	A00QH	Carrots	Italy	Italy				all sample	L*52.2±0.7a*24.1±1.5b*36.1±1	4900± 1000				13400± 800	111

Table S9.29.2 Root and tuber vegetables (excluding starchy- and sugar-) (A00QF) (µg/100g) (continuation)

Food name	Scientific name	FoodEx2_ TermCode	FoodEx2_ TermName	Origin (country)	Purchase (country)	Water (%)	Process	Saponification	Part analysed	Colour	Lutein	Luteoxanthin	Lycopene	Neoxanthin	Phytoene	Ref.
Carrot	*Daucus carota L.*	A00QH	Carrots	Italy	Italy				all sample	L*52.1±0.8a*22.6±1.1b*35.7±1.9	5900± 400				17400± 300	111
Carrot	*Daucus carota L.*	A00QH	Carrots	Italy	Italy				all sample	L*50.2±1.1a*21.4±1.4b*35.3±1.5	4200± 500				13700± 100	111
Carrot	*Daucus carota L.*	A00QH	Carrots	Spain	Spain				flesh	Orange					1340	22
Carrot	*Daucus carota L. cv. Nantes*	A00QH	Carrots	Brazil					tuber	orange	510		nd	nd		109
Carrot	*Daucus carota L. HCM*	A00QH	Carrots	France	France				root	dark-orange	103±14					159
Carrot	*Daucus carota L. var. Blanche à collet vert*	A00QH	Carrots	France	France				root	white	nd					159
Carrot	*Daucus carota L. var. Blanche des vosges*	A00QH	Carrots	France	France				root	white	nd					159
Carrot	*Daucus carota L. var. Carentan*	A00QH	Carrots	France	France				root	orange	145±14					159
Carrot	*Daucus carota L. var. Commercial French*	A00QH	Carrots	France	France	-			root	orange	94±4					159
Carrot	*Daucus carota L. var. De Guérande*	A00QH	Carrots	France	France				root	orange	161±28					159
Carrot	*Daucus carota L. var. Jaune obtuse du Doubs*	A00QH	Carrots	France	France				root	yellow	138±24					159

Table S9.29.2 Root and tuber vegetables (excluding starchy- and sugar-) (A00QF) (µg/100g) (continuation)

Food name	Scientific name	FoodEx2_ TermCode	FoodEx2_ TermName	Origin (country)	Purchase (country)	Water (%)	Process	Saponification	Part analysed	Colour	Lutein	Luteoxanthin	Lycopene	Neoxanthin	Phytoene	Ref.
Carrot	*Daucus carota L. var. Kokubu*	A00QH	Carrots	France	France				root	orange	161±4					159
Carrot	*Daucus carota L. var. La Merveille*	A00QH	Carrots	France	France				root	orange	175±9					159
Carrot	*Daucus carota L. var. New Kuroda*	A00QH	Carrots	France	France				root	orange	93±3					159
Carrot	*Daucus carota L. var. San Naï*	A00QH	Carrots	France	France				root	orange	180±5					159
Carrot	*Daucus carota L. var. sativa, D.C.*	A00QH	Carrots	Spain	Spain	88			root	Orange	288±33					24
Carrot	*Daucus carota L. var. sativa, D.C.*	A00QH	Carrots	Spain	Spain	90			root	Orange	273±25					24
Carrot	*Daucus carota L. var. sativa, D.C.*	A00QH	Carrots	Spain	Spain	88			root	Orange					1769±86	24
Carrot	*Daucus carota L. var. sativa, D.C.*	A00QH	Carrots	Spain	Spain	90			root	Orange					1197±414	24
Carrot	*Daucus carota L. var. Violette jordanienne*	A00QH	Carrots	France	France				root	purple	224±9					159
Carrot	*Daucus carota L. var. Violette turque*	A00QH	Carrots	France	France				root	purple	176±19					159
Carrot	*Daucus carota L. var. yellowstone*	A00QH	Carrots	France	France				root	yellow	232±12					159
Carrot	*Daucus carota L.,*	A00QH	Carrots	Turkey	Turkey				root		6±1 - 107±6					160

Table S9.29.2 Root and tuber vegetables (excluding starchy- and sugar-) (A00QF) (µg/100g) (continuation)

Food name	Scientific name	FoodEx2_TermCode	FoodEx2_TermName	Origin (country)	Purchase (country)	Water (%)	Process	Saponification	Part analysed	Colour	Lutein	Luteoxanthin	Lycopene	Neoxanthin	Phytoene	Ref.
Carrot	*Daucus carota L., var. Bolero*	A00QH	Carrots	Turkey	Turkey				root		29 ±5-46±1					160
Carrot	*Daucus carota L., var. Maestro-F1*	A00QH	Carrots	Turkey	Turkey				root		32±3 - 144±2					160
Carrot	*Daucus carota L., var. Nanco*	A00QH	Carrots	Turkey	Turkey				root		2±2 - 118±6					160
Carrot	*Daucus carota L., var. Nantindo*	A00QH	Carrots	Turkey	Turkey				root		37±4 - 110±2					160
Carrot	*Daucus carota L., var. Tito*	A00QH	Carrots	Turkey	Turkey				root		22±1 - 60±3					160
Carrot	*Daucus carota L.var. Commercial French*	A00QH	Carrots	France	France				root	orange	164±29					159
Carrot	*Daucus carota L.var. Commercial French*	A00QH	Carrots	France	France				root	orange	60±7					159
Carrot	*Daucus carota L.var. Commercial French*	A00QH	Carrots	France	France				root	orange	157±8					159
Carrot	*Daucus carota L.var. Commercial French*	A00QH	Carrots	France	France				root	orange	116±14					159
Carrot	*Daucus carota L.var. Commercial French*	A00QH	Carrots	France	France				root	orange	157±8					159
Swede	*Brassica rapa*	A00RD	Swedes	United Kingdom	United Kingdom				peeled				50±20		120±30	220

Table S9.29.3 Root and tuber vegetables (excluding starchy- and sugar-) (A00QF) (µg/100g) (continuation)

Food name	Scientific name	FoodEx2_ TermCode	FoodEx2_ TermName	Origin (country)	Purchase (country)	Water (%)	Process	Saponification	Part analysed	Colour	Phytofluene	Violaxanthin	Z(v. cis)- lycopene	Z(v. cis)-β- carotene	Z(v. cis)-β- cryptoxanthin	Zeaxanthin	Ref.
Beet	*Beta vulgaris L., 'Bulls Blood'*	A00QG	Beetroots	Lithuania	Lithuania			no	leaves			1907				139	110
Beet	*Beta vulgaris L., 'Bulls Blood'*	A00QG	Beetroots	Lithuania	Lithuania			no	leaves			1643				185	110
Beet	*Beta vulgaris L., 'Bulls Blood'*	A00QG	Beetroots	Lithuania	Lithuania			no	leaves			3855				118	110
Beet	*Beta vulgaris L., 'Bulls Blood'*	A00QG	Beetroots	Lithuania	Lithuania			no	leaves			3298				213	110
Beet	*Beta vulgaris L., 'Bulls Blood'*	A00QG	Beetroots	Lithuania	Lithuania			no	leaves			3909				320	110
Carrot	*Daucus carota L.*	A00QH	Carrots	Germany	Germany				edible part			14					53
Carrot	*Daucus carota L.*	A00QH	Carrots	Italy	Italy				all sample	L*52.2±0.7a*24.1±1.5b*36.1±1	9100± 200						114
Carrot	*Daucus carota L.*	A00QH	Carrots	Italy	Italy				all sample	L*52.1±0.8a*22.6±1.1b*35.7±1.9	11200± 400						111
Carrot	*Daucus carota L.*	A00QH	Carrots	Italy	Italy				all sample	L*50.2±1.1a*21.4±1.4b*35.3±1.5	9700± 100						111
Carrot	*Daucus carota L.*	A00QH	Carrots	Spain	Spain				flesh	orange	570						22
Carrot	*Daucus carota L. cv. Nantes*	A00QH	Carrots	Brazil					tuber	orange		nd				nd	109
Swede	*Brassica rapa*	A00RD	Swedes	United Kingdom	United Kingdom				peeled	-		10±0					220

Table S9.30.1 Algae and prokaryotes organisms (A00VA) (µg/100g)

Food name	Scientific name	FoodEx2_ TermCode	FoodEx2_ TermName	Origin (country)	Purchase (country)	Water (%)	Process	Saponification	Part analysed	Colour	α-carotene	β-carotene	β-cryptoxanthin	ζ-carotene	Antheraxanthin	Ref.
Microalgae	*Dunaliella salina*	A0DCP	Other algae	Israel					plant	green		491-3751				162
Microalgae	*Dunaliella salina*	A0DCP	Other algae	Israel					all sample		78 - 165	978 - 2250				162
Microalgae	*Heterochlorella luteoviridis*	A0DCP	Other algae	Brazil					biomass							163
Microalgae	*Neochloris oleoabundans*	A0DCP	Other algae	United States	Spain				extracts			620 - 1761				164

Table S9.30.2 Algae and prokaryotes organisms (A00VA) (µg/100g) (continuation)

Food name	Scientific name	FoodEx2_ TermCode	FoodEx2_ TermName	Origin (country)	Purchase (country)	Water (%)	Process	Saponification	Part analysed	Colour	Capsanthin	Capsorubin	Cucurbitaxanthin	E(v. trans)-α-carotene	E(v. trans)-β-carotene	Ref.
Microalgae	*Dunaliella salina*	A0DCP	Other algae	Israel		-			plant	green						162
Microalgae	*Dunaliella salina*	A0DCP	Other algae	Israel					all sample							162
Microalgae	*Heterochlorella luteoviridis*	A0DCP	Other algae	Brazil					biomass						18500± 200	163
Microalgae	*Neochloris oleoabundans*	A0DCP	Other algae	United States	Spain				extracts							164

Table S9.30.3 Algae and prokaryotes organisms (A00VA) (µg/100g) (continuation)

Food name	Scientific name	FoodEx2_ TermCode	FoodEx2_ TermName	Origin (country)	Purchase (country)	Water (%)	Process	Saponification	Part analysed	Colour	E(v. trans)-β-cryptoxanthin	E(v. trans)-lutein	E(v. trans)-lycopene	E(v. trans)-zeaxanthin	Lactucaxanthin	Ref.
Microalgae	*Dunaliella salina*	A0DCP	Other algae	Israel		-			plant	green						162
Microalgae	*Dunaliella salina*	A0DCP	Other algae	Israel					all sample							162
Microalgae	*Heterochlorella luteoviridis*	A0DCP	Other algae	Brazil					biomass			85600± 5600		24400± 2000		163
Microalgae	*Neochloris oleoabundans*	A0DCP	Other algae	United States	Spain				extracts							164

Table S9.31.1 Herbs and edible flowers (A00VQ) (µg/100g)

Food name	Scientific name	FoodEx2_ TermCode	FoodEx2_ TermName	Origin (country)	Purchase (country)	Water (%)	Process	Saponification	Part analysed	Colour	α-carotene	β-carotene	β-cryptoxanthin	ζ-carotene	Antheraxanthin	Ref.
Basil	*Ocinum basiliscum*	A00VV	Basil	Italy	Italy						nd	4820	89			25
Betel	*Piper betle L.*	A0DGC	Wild betel leaves	India					leaves without stems		18420	13350				98
Celery	*Apium graveolens*	A00XA	Celery leaves	Spain	Spain				edible part	white		65				24
Coriander	*Coriandrum sativum L.*	A00XF	Coriander leaves	India					leaves without stems			67500				98
Coriander	*Coriandrum sativum L.*	A00XF	Coriander leaves	USA				no			0		0			28
Culinary herbs	*Cyphostemma digitatum*	A04MA	Aromatic herbs	Yemen					leaves			14600± 460				165
Culinary herbs	*Cyphostemma digitatum*	A04MA	Aromatic herbs	Yemen					leaves			14380± 460				165

Table S9.31.1 Herbs and edible flowers (A00VQ) (µg/100g) (continuation)

Food name	Scientific name	FoodEx2_ TermCode	FoodEx2_ TermName	Origin (country)	Purchase (country)	Water (%)	Process	Saponification	Part analysed	Colour	α-carotene	β-carotene	β-cryptoxanthin	ζ-carotene	Antheraxanthin	Ref.
Curry	*Murraya koenigii L.*	A00XG	Curry leaves	India					leaves without stems		2870	8950				98
Dill	*Anethum graveolens L.*	A00XH	Dill leaves	Italy	Italy						94	5450	410			25
Parsley	*Petroselinum crispum*	A00YE	Parsley	Italy	Italy						nd	4440–4680	nd			25
Parsley	*Petroselinum crispum*	A00YE	Parsley	Germany	Germany	89.5			edible part		170	5500	110			53
Parsley	*Petroselinum crispum*	A00YE	Parsley	Lithuania	Lithuania			no	leaves		3	54				110
Parsley	*Petroselinum crispum*	A00YE	Parsley	Lithuania	Lithuania			no	leaves		5	53				110
Parsley	*Petroselinum crispum*	A00YE	Parsley	Lithuania	Lithuania			no	leaves		2	43				110
Parsley	*Petroselinum crispum*	A00YE	Parsley	Lithuania	Lithuania			no	leaves		22	6				110
Parsley	*Petroselinum crispum*	A00YE	Parsley	Lithuania	Lithuania			no	leaves		12	86				110
Parsley	*Petroselinum crispum*	A00YE	Parsley	USA				no			0		0			28
Sage	*Salvia officinalis L.*	A00YH	Sage	Italy	Italy						nd	2780	87			25
Spearmint	*Mentha spicata L.*	A00YC	Spearmint	India					leaves without stems			7480				98

Table S9.31.2 Herbs and edible flowers (A00VQ) (µg/100g) (continuation)

Food name	Scientific name	FoodEx2_ TermCode	FoodEx2_ TermName	Origin (country)	Purchase (country)	Water (%)	Process	Saponification	Part analysed	Colour	Capsanthin	Capsorubin	Cucurbitaxanthin	E(v. trans)-α-carotene	E(v. trans)-β-carotene	Ref.
Coriander	*Coriandrum sativum L.*	A00XF	Coriander leaves	USA				no							5531	28
Dill	*Anethum graveolens L.*	A00XH	Dill leaves	Germany	Germany										10800± 2120	113
Parsley	*Petroselinum crispum*	A00YE	Parsley	Germany	Germany										3610±60	113
Parsley	*Petroselinum crispum*	A00YE	Parsley	USA				no							2264	28

Table S9.31.3 Herbs and edible flowers (A00VQ) (µg/100g) (continuation)

Food name	Scientific name	FoodEx2_ TermCode	FoodEx2_ TermName	Origin (country)	Purchase (country)	Water (%)	Process	Saponification	Part analysed	Colour	E(v. trans)-β-cryptoxanthin	E(v. trans)-lutein	E(v. trans)-lycopene	E(v. trans)-zeaxanthin	Lactucaxanthin	Ref.
Coriander	*Coriandrum sativum L.*	A00XF	Coriander leaves	USA				no				7703		0		28
culinary herbs	*Cyphostemma digitatum*	A04MA	Aromatic herbs	Yemen					leaves		670±30	18890± 730		9460± 300		165
culinary herbs	*Cyphostemma digitatum*	A04MA	Aromatic herbs	Yemen					leaves			190±30		490±10		165
Dill	*Anethum graveolens L.*	A00XH	Dill leaves	Germany	Germany							21490± 4520		540±160		113
Parsley	*Petroselinum crispum*	A00YE	Parsley	Germany	Germany							8840±20		180±20		113
Parsley	*Petroselinum crispum*	A00YE	Parsley	USA				no				4326		0		28

Table S9.31.4 Herbs and edible flowers (A00VQ) (µg/100g) (continuation)

Food name	Scientific name	FoodEx2_TermCode	FoodEx2_TermName	Origin (country)	Purchase (country)	Water (%)	Process	Saponification	Part analysed	Colour	Lutein	Luteoxanthin	Lycopene	Neoxanthin	Phytoene	Ref.
Basil	*Ocinum basiliscum*	A00VV	Basil	Italy	Italy						7050		nd			25
Basil	*Ocinum basiliscum, variety Aromat*	A00VV	Basil	Latvia	Latvia				leaves	violet	18000					112
Basil	*Ocinum basiliscum, variety Genovese*	A00VV	Basil	Latvia	Latvia				leaves	green	18000					112
Betel	*Piper betle L.*	A0DGC	Wild betel leaves	India	India				leaves without stems		36430			820		98
Celery	*Apium graveolens*	A00XA	Celery leaves	Spain	Spain				edible part	white	163					24
Common nettles	*Urtica dioica*	A00PA	Common nettle	Latvia	Latvia				leaves	green	38000					112
Coriander	*Coriandrum sativum L.*	A00XF	Coriander leaves	India	India				leaves without stems		9920			5470		98
Curry	*Murraya koenigii L.*	A00XG	Curry leaves	India	India				leaves without stems		27200			4390		98
Dill	*Anethum graveolens L.*	A00XH	Dill leaves	Italy	Italy						13820		nd			25
Parsley	*Petroselinum crispum*	A00YE	Parsley	Italy	Italy						6400–10 650		nd			25
Parsley	*Petroselinum crispum*	A00YE	Parsley	Germany	Germany	89.5			edible part		13780			370		53
Parsley	*Petroselinum crispum*	A00YE	Parsley	Lithuania	Lithuania			no	leaves		12291			1226		110
Parsley	*Petroselinum crispum*	A00YE	Parsley	Lithuania	Lithuania			no	leaves		15991			1738		110
Parsley	*Petroselinum crispum*	A00YE	Parsley	Lithuania	Lithuania			no	leaves		19068			2013		110
Parsley	*Petroselinum crispum*	A00YE	Parsley	Lithuania	Lithuania			no	leaves		17108			1830		110
Parsley	*Petroselinum crispum*	A00YE	Parsley	Lithuania	Lithuania			no	leaves		17051			1646		110

Table S9.31.4 Herbs and edible flowers (A00VQ) (µg/100g) (continuation)

Food name	Scientific name	FoodEx2_ TermCode	FoodEx2_ TermName	Origin (country)	Purchase (country)	Water (%)	Process	Saponification	Part analysed	Colour	Lutein	Luteoxanthin	Lycopene	Neoxanthin	Phytoene	Ref.
Sage	*Salvia officinalis L.*	A00YH	Sage	Italy	Italy						6350		nd			25
Spearmint	*Mentha spicata L.*	A00YC	Spearmint	India	India				leaves without stems		17740			2110		98

Table S9.31.5 Herbs and edible flowers (A00VQ) (µg/100g) (continuation)

Food name	Scientific name	FoodEx2_ TermCode	FoodEx2_ TermName	Origin (country)	Purchase (country)	Water (%)	Process	Saponification	Part analysed	Colour	Phytofluene	Violaxanthin	Z(v. cis)-lycopene	Z(v. cis)-β-carotene	Z(v. cis)-β-cryptoxanthin	Zeaxanthin	Ref.
Betel leaves	*Piper betle L.*	A0DGC	Wild betel leaves	India					leaves without stems			890				470	98
Coriander	*Coriandrum sativum L.*	A00XF	Coriander leaves	India					leaves without stems			83430					98
Coriander	*Coriandrum sativum L.*	A00XF	Coriander leaves	USA				no	leaves					933			28
Curry	*Murraya koenigii L.*	A00XG	Curry leaves	India					leaves without stems			6680				160	98
Parsley	*Petroselinum crispum*	A00YE	Parsley	Germany	Germany	89.5			edible part			3590				340	53
Parsley	*Petroselinum crispum*	A00YE	Parsley	Lithuania	Lithuania			no	leaves			1459				84	110
Parsley	*Petroselinum crispum*	A00YE	Parsley	Lithuania	Lithuania			no	leaves			2131				9	110
Parsley	*Petroselinum crispum*	A00YE	Parsley	Lithuania	Lithuania			no	leaves			2858				112	110
Parsley	*Petroselinum crispum*	A00YE	Parsley	Lithuania	Lithuania			no	leaves			2314				1242	110

Table S9.31.5 Herbs and edible flowers (A00VQ) (µg/100g) (continuation)

Food name	Scientific name	FoodEx2_ TermCode	FoodEx2_ TermName	Origin (country)	Purchase (country)	Water (%)	Process	Saponification	Part analysed	Colour	Phytofluene	Violaxanthin	Z(v. cis)-lycopene	Z(v. cis)-β-carotene	Z(v. cis)-β-cryptoxanthin	Zeaxanthin	Ref.
Parsley	*Petroselinum crispum*	A00YE	Parsley	Lithuania	Lithuania			no	leaves			2181				1440	110
Parsley	*Petroselinum crispum*	A00YE	Parsley	USA				no						317			28
Spearmint	*Mentha spicata L.*	A00YC	Spearmint	India					leaves without stems			5620				260	98

Table S9.32.1 Processed or preserved vegetables and similar (A00ZA) (µg/100g)

Food name	Scientific name	FoodEx2_ TermCode	FoodEx2_ TermName	Origin (country)	Purchase (country)	Water (%)	Process	Saponification	Part analysed	Colour	α-carotene	β-carotene	β-cryptoxanthin	ζ-carotene	Antheraxanthin	Ref.
Carrot	*Daucus carota cv Nerac*	A00ZN#F27.A00QH	Mashed vegetable puree, SOURCE-COMMODITIES = Carrots	Belgium	Belgium		puree	no		orange		88863				166
Carrot	*Daucus carota cv Nerac*	A00ZN#F27.A00QH	Mashed vegetable puree, SOURCE-COMMODITIES = Carrots	Belgium	Belgium		puree	no		orange		93662				166
Carrot	*Daucus carota cv Nerac*	A00ZN#F27.A00QH	Mashed vegetable puree, SOURCE-COMMODITIES = Carrots	Belgium	Belgium		puree	no		orange		95918				166
Carrot	*Daucus carota cv Nerac*	A00ZN#F27.A00QH	Mashed vegetable puree, SOURCE-COMMODITIES = Carrots	Belgium	Belgium		puree	no		orange		89425				166

Table S9.32.1 Processed or preserved vegetables and similar (A00ZA) (µg/100g) (continuation)

Food name	Scientific name	FoodEx2_ TermCode	FoodEx2_ TermName	Origin (country)	Purchase (country)	Water (%)	Process	Saponification	Part analysed	Colour	α-carotene	β-carotene	β-cryptoxanthin	ζ-carotene	Antheraxanthin	Ref.
Carrot	*Daucus carota L.*	A0ETQ#F27.A00QH	Canned/jarred vegetables, SOURCE-COMMODITIES = Carrots	Spain	Spain		canned		edible part		3700	9800				24
Grass	*Cerastium fontanum*	A00ZQ#F27.A0DHN$F03.A07Y3	Dried vegetables, SOURCE-COMMODITIES = Creeping thyme, PHYSICAL-STATE = Coarse powder	Spain	Spain		lyophilized ground		grass			3760± 709			1550±64	167
Grass	*Erica vagans*	A00ZQ#F27.A0DHN$F03.A07Y3	Dried vegetables, SOURCE-COMMODITIES = Creeping thyme, PHYSICAL-STATE = Coarse powder	Spain	Spain		lyophilized ground		grass			3060±98			1550±64	167
Grass	*Potentilla montana*	A00ZQ#F27.A0DHN$F03.A07Y3	Dried vegetables, SOURCE-COMMODITIES = Creeping thyme, PHYSICAL-STATE = Coarse powder	Spain	Spain		lyophilized ground		grass			3060±98			1680±4	167
Grass	*Ranunculus bulbosus*	A00ZQ#F27.A0DHN$F03.A07Y3	Dried vegetables, SOURCE-COMMODITIES = Creeping thyme, PHYSICAL-STATE = Coarse powder	Spain	Spain		lyophilized ground		grass		2760± 315	2560± 344	2090± 114		1810±25	167
Grass	*Thymus praecox*	A00ZQ#F27.A0DHN$F03.A07Y3	Dried vegetables, SOURCE-COMMODITIES = Creeping thyme, PHYSICAL-STATE = Coarse powder	Spain	Spain		lyophilized ground		grass			1830±13				167
Grass	*Trifolium repens*	A00ZQ#F27.A0DHN$F03.A07Y3	Dried vegetables, SOURCE-COMMODITIES = Creeping thyme, PHYSICAL-STATE = Coarse powder	Spain	Spain		lyophilized ground		grass			2910±148			1720±41	167
Tomato	*Lycopersicon esculentum L.*	A00ZD	Tomato puree	Italy	Italy		puree				nd	383–548	nd			25
Tomato	*Lycopersicon esculentum L.*	A0ETQ#F27.A0DMX	Canned/jarred vegetables, SOURCE-COMMODITIES = Tomatoes	Italy	Italy		canned				nd	217–283	nd			25
Tomato	*Lycopersicum esculentum L.*	A0ETQ#F27.A0DMX	Canned/jarred vegetables, SOURCE-COMMODITIES = Tomatoes	Spain	Spain		canned		edible part			186				24

Table S9.32.2 Processed or preserved vegetables and similar (A00ZA) (µg/100g) (continuation)

Food name	Scientific name	FoodEx2_ TermCode	FoodEx2_ TermName	Origin (country)	Purchase (country)	Water (%)	Process	Saponification	Part analysed	Colour	E(v. trans)-β-cryptoxanthin	E(v. trans)-lutein	E(v. trans)-lycopene	E(v. trans)-zeaxanthin	Lactucaxanthin	Ref.
Grass	*Cerastium fontanum*	A00ZQ#F27.A0DHN$F03.A07Y3	Dried vegetables, SOURCE-COMMODITIES = Creeping thyme, PHYSICAL-STATE = Coarse powder	Spain	Spain		lyophilized ground		grass			8540± 590				167
Grass	*Erica vagans*	A00ZQ#F27.A0DHN$F03.A07Y3	Dried vegetables, SOURCE-COMMODITIES = Creeping thyme, PHYSICAL-STATE = Coarse powder	Spain	Spain		lyophilized ground		grass			8540± 590				167
Grass	*Potentilla montana*	A00ZQ#F27.A0DHN$F03.A07Y3	Dried vegetables, SOURCE-COMMODITIES = Creeping thyme, PHYSICAL-STATE = Coarse powder	Spain	Spain		lyophilized ground		grass			15800± 378				167
Grass	*Ranunculus bulbosus*	A00ZQ#F27.A0DHN$F03.A07Y3	Dried vegetables, SOURCE-COMMODITIES = Creeping thyme, PHYSICAL-STATE = Coarse powder	Spain	Spain		lyophilized ground		grass			9560±24				167
Grass	*Thymus praecox*	A00ZQ#F27.A0DHN$F03.A07Y3	Dried vegetables, SOURCE-COMMODITIES = Creeping thyme, PHYSICAL-STATE = Coarse powder	Spain	Spain		lyophilized ground		grass			4580±29				167
Grass	*Trifolium repens*	A00ZQ#F27.A0DHN$F03.A07Y3	Dried vegetables, SOURCE-COMMODITIES = Creeping thyme, PHYSICAL-STATE = Coarse powder	Spain	Spain		lyophilized ground		grass			1330± 918				167
Tomato	*Lycopersicon esculentum L.*	A00ZE#F28.A07KG$F20.A0F7Q	Preserved concentrated tomatoes, PROCESS = Drying (dehydration), PART-CONSUMED-ANALYSED = Only skin	Finland	Finland		dried		skin				28320			26
Tomato	*Lycopersicon esculentum L.*	A00ZF	Tomato paste	Finland	Finland				tomato paste				32040			26

Table S9.32.3 Processed or preserved vegetables and similar (A00ZA) (µg/100g) (continuation)

Food name	Scientific name	FoodEx2_ TermCode	FoodEx2_ TermName	Origin (country)	Purchase (country)	Water (%)	Process	Saponification	Part analysed	Colour	Lutein	Luteoxanthin	Lycopene	Neoxanthin	Phytoene	Ref.
Grass	*Cerastium fontanum*	A00ZQ#F27.A0DHN$F03.A07Y3	Dried vegetables, SOURCE-COMMODITIES = Creeping thyme, PHYSICAL-STATE = Coarse powder	Spain	Spain		lyophilized ground		grass		3640±174					167
Grass	*Erica vagans*	A00ZQ#F27.A0DHN$F03.A07Y3	Dried vegetables, SOURCE-COMMODITIES = Creeping thyme, PHYSICAL-STATE = Coarse powder	Spain	Spain		lyophilized ground		grass		3640± 174					167
Grass	*Potentilla montana*	A00ZQ#F27.A0DHN$F03.A07Y3	Dried vegetables, SOURCE-COMMODITIES = Creeping thyme, PHYSICAL-STATE = Coarse powder	Spain	Spain		lyophilized ground		grass		7720±97			1950±79		167
Grass	*Ranunculus bulbosus*	A00ZQ#F27.A0DHN$F03.A07Y3	Dried vegetables, SOURCE-COMMODITIES = Creeping thyme, PHYSICAL-STATE = Coarse powder	Spain	Spain		lyophilized ground		grass		3440± 291			1890±18		167
Grass	*Thymus praecox*	A00ZQ#F27.A0DHN$F03.A07Y3	Dried vegetables, SOURCE-COMMODITIES = Creeping thyme, PHYSICAL-STATE = Coarse powder	Spain	Spain		lyophilized ground		grass		2240±31			1510±8		167
Grass	*Trifolium repens*	A00ZQ#F27.A0DHN$F03.A07Y3	Dried vegetables, SOURCE-COMMODITIES = Creeping thyme, PHYSICAL-STATE = Coarse powder	Spain	Spain		lyophilized ground		grass		4250±110			2030±56		167
Tomato	*Lycopersicon esculentum L.*	A00ZD	Tomato puree	Italy	Italy		puree				nd		13 160–26 110			25
Tomato	*Lycopersicon esculentum L.*	A00ZD	Tomato puree	Italy	Italy		puree		puree	red			830.0			168
Tomato	*Lycopersicon esculentum L.*	A0ETQ#F27.A0DMX	Canned/jarred vegetables, SOURCE-COMMODITIES = Tomatoes	Italy	Italy		canned				nd		8480–11820			25
Tomato	*Lycopersicum esculentum L.*	A0ETQ#F27.A0DMX	Canned/jarred vegetables, SOURCE-COMMODITIES = Tomatoes	Spain	Spain		canned		edible part				9708			24
Tomato	*Solanum lycopersicum L.*	A00ZD	Tomato puree	Spain	Spain		puree		Fruit	red					2400	22

Table S9.32.3 Processed or preserved vegetables and similar (A00ZA) (µg/100g) (continuation)

Food name	Scientific name	FoodEx2_ TermCode	FoodEx2_ TermName	Origin (country)	Purchase (country)	Water (%)	Process	Saponification	Part analysed	Colour	Lutein	Luteoxanthin	Lycopene	Neoxanthin	Phytoene	Ref.
Tomato	*Solanum lycopersicum L.*	A04MB#F28. A07KG$F20.A 0F7P	Processed tomato products, PROCESS = Drying (dehydration), PART-CONSUMED-ANALYSED = Only peel	Spain	Spain				dry peel				0.2±0.02			169
Tomato	*Solanum lycopersicum L.*	A04MB#F28. A07KG$F03.A 06JD	Processed tomato products, PROCESS = Drying (dehydration), PHYSICAL-STATE = Powder	Spain	Spain				powder				0.1±0.01			169
Tomato	*Solanum lycopersicum L.*	A04MB#F28. A07KG$F20.A 0F7P	Processed tomato products, PROCESS = Drying (dehydration), PART-CONSUMED-ANALYSED = Only peel	Spain	Spain				dry peel				0.1±0.01			169
Tomato	*Solanum lycopersicum L.*	A04MB#F28. A07KG$F03.A 06JD	Processed tomato products, PROCESS = Drying (dehydration), PHYSICAL-STATE = Powder	Spain	Spain				powder				0.			169

Table S9.32.4 Processed or preserved vegetables and similar (A00ZA) (µg/100g) (continuation)

Food name	Scientific name	FoodEx2_ TermCode	FoodEx2_ TermName	Origin (country)	Purchase (country)	Water (%)	Process	Saponification	Part analysed	Colour	Phytofluene	Violaxanthin	Z(v. cis)-lycopene	Z(v. cis)-β-carotene	Z(v. cis)-β-cryptoxanthin	Zeaxanthin	Ref.
Grass	*Potentilla montana*	A00ZQ#F2 7.A0DHN$ F03.A07Y3	Dried vegetables, SOURCE-COMMODITIES = Creeping thyme, PHYSICAL-STATE = Coarse powder	Spain	Spain		lyophilized ground		grass			1620±28				304±27	169
Grass	*Ranunculus bulbosus*	A00ZQ#F2 7.A0DHN$ F03.A07Y3	Dried vegetables, SOURCE-COMMODITIES = Creeping thyme, PHYSICAL-STATE = Coarse powder	Spain	Spain		lyophilized ground		grass			1780±46				1730±96	169

Table S9.32.4 Processed or preserved vegetables and similar (A00ZA) (µg/100g) (continuation)

Food name	Scientific name	FoodEx2_ TermCode	FoodEx2_ TermName	Origin (country)	Purchase (country)	Water (%)	Process	Saponification	Part analysed	Colour	Phytofluene	Violaxanthin	Z(v. cis)-lycopene	Z(v. cis)-β-carotene	Z(v. cis)-β-cryptoxanthin	Zeaxanthin	Ref.
Grass	*Trifolium repens*	A00ZQ#F27.A0DHN$F03.A07Y3	Dried vegetables, SOURCE-COMMODITIES = Creeping thyme, PHYSICAL-STATE = Coarse powder	Spain	Spain		lyophilized ground		grass			2170±437				1890±226	169
Tomato	*Lycopersicon esculentum L.*	A00ZD	Tomato puree	Italy	Italy		puree									nd	25
Tomato	*Lycopersicon esculentum L.*	A0ETQ#F27.A0DMX	Canned/jarred vegetables, SOURCE-COMMODITIES = Tomatoes	Italy	Italy		canned									nd	25
Tomato	*Solanum lycopersicum L.*	A00ZD	Tomato puree	Spain	Spain		puree		fruit	red	1080						22

Table S10. Grains and grain-based products (A000J)

Table S10.1.1 Cereal grains (and cereal-like grains) (A000L) (µg/100g)

Food name	Scientific name	FoodEx2_ TermCode	FoodEx2_ TermName	Origin (country)	Purchase (country)	Water (%)	Saponification	Part analysed	Colour	α-carotene	β-carotene	β-cryptoxanthin	Antheraxanthin	Ref.
Barley	*Hordeum vulgare L. var. AF Cesar*	A0D9Y	Barley and similar-	Czech Republic	Czech Republic			grain		nd	3		18	170
Einkorn wheat	*T. aegilopoides*	A001S	Einkorn wheat grain	Italy	Italy			sSeeds	light yellow		70±8	9±0.4		171
Einkorn wheat	*T. aestivum*	A001S	Einkorn wheat grain	Italy	Italy			seeds	light yellow		nd	nd		171
Einkorn wheat	*T. dicoccum*	A001S	Einkorn wheat grain	Italy	Italy			seeds	light yellow		nd	nd		171
Einkorn wheat	*T. durum*	A001S	Einkorn wheat grain	Italy	Italy			seeds	light yellow		50±3	2±0.2		171
Einkorn wheat	*T. monococcum*	A001S	Einkorn wheat grain	Italy	Italy			seeds	light yellow		60±18	6±1.6		171

Table S10.1.1 Cereal grains (and cereal-like grains) (A000L) (µg/100g) (continuation)

Food name	Scientific name	FoodEx2_ TermCode	FoodEx2_ TermName	Origin (country)	Purchase (country)	Water (%)	Saponification	Part analysed	Colour	α-carotene	β-carotene	β-cryptoxanthin	Antheraxanthin	Ref.
Einkorn wheat	*T. spelta*	A001S	Einkorn wheat grain	Italy	Italy			seeds	light yellow		20±2	nd		171
Einkorn wheat	*T. thaoudar*	A001S	Einkorn wheat grain	Italy	Italy			seeds	light yellow		50±6	5±0.7		171
Einkorn wheat	*T. urartu*	A001S	Einkorn wheat grain	Italy	Italy			seeds	light yellow		nd	nd		171
Einkorn wheat	*Triticum monococcum L. subsp. monococcum*	A001S	Einkorn wheat grain	Italy	Italy			whole meal				70±0,1		172
Einkorn wheat	*Triticum monococcum L. subsp. monococcum*	A001S	Einkorn wheat grain	Italy	Italy			whole meal				7±0,2		172
Einkorn wheat	*Triticum monococcum L. subsp. monococcum*	A001S	Einkorn wheat grain	Italy	Italy			whole meal				3±0.1		172
Einkorn wheat	*Triticum monococcum L. subsp. monococcum*	A001S	Einkorn wheat grain	Italy	Italy			whole meal				4±0.1		172
Einkorn wheat	*Triticum monococcum L. subsp. monococcum*	A001S	Einkorn wheat grain	Italy	Italy			whole meal				5±0.1		172
Einkorn wheat	*Triticum monococcum L. subsp. monococcum*	A001S	Einkorn wheat grain	Italy	Italy			whole meal				9±0.3		172
Einkorn wheat	*Triticum monococcum L. subsp. monococcum var. Monarca*	A001S	Einkorn wheat grain	Italy	Italy			seeds	L*a*b* values 52.9/8.9/22 .4		32 (sum of α- and β-carotene)± 1	4±0		173

Table S10.1.1 Cereal grains (and cereal-like grains) (A000L) (µg/100g) (continuation)

Food name	Scientific name	FoodEx2_ TermCode	FoodEx2_ TermName	Origin (country)	Purchase (country)	Water (%)	Saponification	Part analysed	Colour	α-carotene	β-carotene	β-cryptoxanthin	Antheraxanthin	Ref.
Einkorn wheat	*Triticum monococcum L. subsp. monococcum var. Monlis*	A001S	Einkorn wheat grain	Italy	Italy			seeds	L*a*b* values 53.3/9.1/22.8		87 (sum of α- and β-carotene)± 3	13±1		173
Maize	*Zea mays L.*	A000T	Maize grain	Spain	Spain			edible part	yellow	33	30			24
Maize	*Zea mays L.*	A000T	Maize grain	Croatia	Croatia			all sample			117.3± 8.17	158.5±9.5		174
Maize	*Zea mays L.*	A000T#F28.A07KQ$F28.A0BA1	Maize grain, PROCESS = Freezing, PROCESS = Cooking and similar thermal preparation processes	USA			no			15		0		28
Rice	*Oryza sativa L. Camargue long noir complet Bio cv. Artemide*	A001D#F10.A166Z	Rice grain, QUALITATIVE-INFO = Black	France	France			seeds	black		20±1			176
Rice	*Oryza sativa L. Camargue long noir complet Bio, cv. Artemide*	A001D#F10.A166Z	Rice grain, QUALITATIVE-INFO = Black	France	France			seeds	black		20±1			176
Tritordeum	*Tritordeum martinii A. Pujadasnothosp. nov.*	A001Y	Other cereals	Czech Republic	Spain			grain	yellow endosperm	7	4		67	170
Wheat	*Triticum aestivum L.*	A001N	Common wheat grain	Czech Republic				grain	yellow endosperm	6	22		44	170
Wheat	*Triticum aestivum L.*	A001N	Common wheat grain	Czech Republic	Czech Republic			grain	purple pericarp	nd	4		15	170
0Wheat	*Triticum aestivum L.*	A001N	Common wheat grain	Czech Republic				grain	blue aleurone	nd	29		16	170
Wheat	*Triticum aestivum L.*	A001N	Common wheat grain	Czech Republic	Czech Republic			grain	blue aleurone	nd	nd		8	170
Wheat	*Triticum aestivum L. ssp. aestivum cv Blasco*	A001N	Common wheat grain	Italy	Italy			seeds			9 (sum of α- and β-carotene)± 1	n.d.		173

Table S10.1.1 Cereal grains (and cereal-like grains) (A000L) (µg/100g) (continuation)

Food name	Scientific name	FoodEx2_ TermCode	FoodEx2_ TermName	Origin (country)	Purchase (country)	Water (%)	Saponification	Part analysed	Colour	α-carotene	β-carotene	β-cryptoxanthin	Antheraxanthin	Ref.
Wheat	*Triticum aestivum L. ssp. aestivum cv Bramante*	A001N	Common wheat grain	Italy	Italy			seeds			16 (sum of α- and β-carotene)± 1	n.d.		173
Wheat	*Triticum aestivum L. var.*	A001N	Common wheat grain	Czech Republic	Czech Republic			grain	blue aleurone	nd	nd		34	170
Wheat	*Triticum aestivum L. var. Bohemia*	A001N	Common wheat grain	Czech Republic	Czech Republic			grain	convention al red grain	nd	nd		140	170
Wheat	*Triticum aestivum L. var. Konini*	A001N	Common wheat grain	Czech Republic				grain	purple pericarp	5	18		37	170
Wheat	*Triticum aestivum L. var. PS Karkulka*	A001N	Common wheat grain	Czech Republic	Slovakia			grain	purple pericarp	nd	9		18	170
Wheat	*Triticum aestivum L. var. purple*	A001N	Common wheat grain	Czech Republic				grain	purple pericarp	nd	2		15	170
Wheat	*Triticum aestivum L. var. purple Feed*	A001N	Common wheat grain	Czech Republic				grain	purple pericarp	nd	nd		10.5	170
Wheat	*Triticum aestivum L. var. Tschermak's B.S.*	A001N	Common wheat grain	Czech Republic	Austria			grain	blue aleurone	1	11		22	170
Wheat	*Triticum aestivum L. var. Xiao Yian*	A001N	Common wheat grain	Czech Republic				grain	blue aleurone	nd	nd		11	170

Table S10.1.2 Cereal grains (and cereal-like grains) (A000L) (µg/100g) (continuation)

Food name	Scientific name	FoodEx2_ TermCode	FoodEx2_ TermName	Origin (country)	Purchase (country)	Water (%)	Saponification	Part analysed	Colour	E(v. trans)-α-carotene	E(v. trans)-β-carotene	E(v. trans)-β-cryptoxanthin	E(v. trans)-lutein	Ref.
Maize	*Zea mays L.*	A000T	Maize grain	Netherlands	Netherlands			kernels		20±7	424±6	157±3		175
Maize	*Zea mays L.*	A000T	Maize grain	Netherlands	Netherlands			kernels		44±9	447±28	29±2		175
Maize	*Zea mays L.*	A000T	Maize grain	Netherlands	Netherlands			kernels		58±0	40±2	93±1		175
Maize	*Zea mays L.*	A000T	Maize grain	Netherlands	Netherlands			kernels		3±1	246±8	453±8		175
Maize	*Zea mays L.*	A000T	Maize grain	Netherlands	Netherlands			kernels		44±1	879±28	37±3		175
Maize	*Zea mays L.*	A000T	Maize grain	Netherlands	Netherlands			kernels		41±7	448±15	260±10		175
Maize	*Zea mays L.*	A000T	Maize grain	Netherlands	Netherlands			kernels		17±0	368±2	988±5		175
Maize	*Zea mays L.*	A000T	Maize grain	Netherlands	Netherlands			kernels		23±2	56±2	37±2		175
Maize	*Zea mays L.*	A000T	Maize grain	Netherlands	Netherlands			kernels		11±0	37±0	41±4		175
Maize	*Zea mays L.*	A000T	Maize grain	United States	United States					6±4	253±13	375±15		175
Maize	*Zea mays L.*	A000T	Maize grain	Netherlands	Netherlands			kernels		6±4	253±13	375±15		175
Maize	*Zea mays L.*	A000T	Maize grain	Netherlands	Netherlands			kernels		16±3	303±16	251±8		175
Maize	*Zea mays L.*	A000T	Maize grain	Netherlands	Netherlands			kernels		86±6	277±8	84±2		175
Maize	*Zea mays L.*	A000T	Maize grain	Netherlands	Netherlands			kernels		23±1	305±20	371±15		175
Maize	*Zea mays L.*	A000T#F28.A07KQ$F28.A0BA1	Maize grain, PROCESS = Freezing, PROCESS = Cooking and similar thermal preparation processes	USA			no				14		202	28

Table S10.1.3 Cereal grains (and cereal-like grains) (A000L) (µg/100g) (continuation)

Food name	Scientific name	FoodEx2_ TermCode	FoodEx2_ TermName	Origin (country)	Purchase (country)	Water (%)	Saponification	Part analysed	Colour	E(v. trans)-zeaxanthin	Lutein	Lycopene	Z(v. cis)-β-carotene	Zeaxanthin	Ref.
Barley	*Hordeum vulgare L. var. AF Cesar*	A0D9Y	Barley and similar-	Czech Republic	Czech Republic			grain	standard		94			126	170
Durum	*Durum wheat accessions*	A001P	Durum wheat grain	Spain	Spain			grains	yellow		90				177
Einkorn wheat	*T. aegilopoides*	A001S	Einkorn wheat grain	Italy	Italy			seeds	light yellow		690±75			60±1	171
Einkorn wheat	*T. aestivum*	A001S	Einkorn wheat grain	Italy	Italy			seeds	light yellow		110±3			30±1	171
Einkorn wheat	*T. dicoccum*	A001S	Einkorn wheat grain	Italy	Italy			seeds	light yellow		160±2			30±1	171
Einkorn wheat	*T. durum*	A001S	Einkorn wheat grain	Italy	Italy			seeds	light yellow		530±10			30±1	171
Einkorn wheat	*T. monococcum*	A001S	Einkorn wheat grain	Italy	Italy			seeds	light yellow		610±51			60±4	171
Einkorn wheat	*T. spelta*	A001S	Einkorn wheat grain	Italy	Italy			seeds	light yellow		240±9			40±0	171
Einkorn wheat	*T. thaoudar*	A001S	Einkorn wheat grain	Italy	Italy			seeds	light yellow		670±75			80±10	171
Einkorn wheat	*T. urartu*	A001S	Einkorn wheat grain	Italy	Italy			seeds	light yellow		200±0			70±2	171
Einkorn wheat	*Triticum monococcum L. subsp. monococcum*	A001S	Einkorn wheat grain	Italy	Italy			whole meal			980±37			76±2,6	172
Einkorn wheat	*Triticum monococcum L. subsp. monococcum*	A001S	Einkorn wheat grain	Italy	Italy			whole meal			680±15			73±2,9	172
Einkorn wheat	*Triticum monococcum L. subsp. monococcum*	A001S	Einkorn wheat grain	Italy	Italy			whole meal			570±7			41±1,1	172
Einkorn wheat	*Triticum monococcum L. subsp. monococcum*	A001S	Einkorn wheat grain	Italy	Italy			whole meal			700±17			53±1,9	172

Table S10.1.3 Cereal grains (and cereal-like grains) (A000L) (µg/100g) (continuation)

Food name	Scientific name	FoodEx2_ TermCode	FoodEx2_ TermName	Origin (country)	Purchase (country)	Water (%)	Saponification	Part analysed	Colour	E(v. trans)-zeaxanthin	Lutein	Lycopene	Z(v. cis)-β-carotene	Zeaxanthin	Ref.
Einkorn wheat	*Triticum monococcum L. subsp. monococcum*	A001S	Einkorn wheat grain	Italy	Italy			whole meal			440±6			49±1,1	172
Einkorn wheat	*Triticum monococcum L. subsp. monococcum*	A001S	Einkorn wheat grain	Italy	Italy			whole meal			1060±49			109±6,8	172
Einkorn wheat	*Triticum monococcum L. subsp. monococcum var. Monarca*	A001S	Einkorn wheat grain	Italy	Italy	not given		seeds	L*a*b* values 52.9/8.9/22.4		360±3			38±0	173
Einkorn wheat	*Triticum monococcum L. subsp. monococcum var. Monlis*	A001S	Einkorn wheat grain	Italy	Italy	not given		seeds	L*a*b* values 53.3/9.1/22.8		571±16			41±1	173
Einkorn wheat	*Triticum monococcum L.*	A001S	Einkorn wheat grain	Germany	Germany			whole grain	ni		614.0				178
Maize	*Zea mays L.*	A000T#F28. A07KQ$F28. A0BA1	Maize grain, PROCESS = Freezing, PROCESS = Cooking and similar thermal preparation processes	USA			no			202			0		28
Maize	*Zea mays L.*	A000T	Maize grain	Netherlands	Netherlands			kernels			811±64		177±2	595±29	175
Maize	*Zea mays L.*	A000T	Maize grain	Netherlands	Netherlands			kernels			847±31		140±5	129±14	175
Maize	*Zea mays L.*	A000T	Maize grain	Netherlands	Netherlands			kernels			640±31		11±1	175±13	175
Maize	*Zea mays L.*	A000T	Maize grain	Netherlands	Netherlands			kernels			41±3		103±1	1625±73	175

Table S10.1.3 Cereal grains (and cereal-like grains) (A000L) (µg/100g) (continuation)

Food name	Scientific name	FoodEx2_ TermCode	FoodEx2_ TermName	Origin (country)	Purchase (country)	Water (%)	Saponification	Part analysed	Colour	E(v. trans)-zeaxanthin	Lutein	Lycopene	Z(v. cis)-β-carotene	Zeaxanthin	Ref.
Maize	*Zea mays L.*	A000T	Maize grain	Netherlands	Netherlands			kernels			535±18		301±29	173±6	175
Maize	*Zea mays L.*	A000T	Maize grain	Netherlands	Netherlands			kernels			790±18		131±13	1302±21	175
Maize	*Zea mays L.*	A000T	Maize grain	Netherlands	Netherlands			kernels			<0.1±0		104±3	1680±43	175
Maize	*Zea mays L.*	A000T	Maize grain	Netherlands	Netherlands			kernels			553±62		<0.1±0	190±21	175
Maize	*Zea mays L.*	A000T	Maize grain	Netherlands	Netherlands			kernels			378±8		98±13	311±7	175
Maize	*Zea mays L.*	A000T	Maize grain	USA							47±0		87±7	1270±45	175
Maize	*Zea mays L.*	A000T	Maize grain	Spain	Spain			edible part	yellow		411			218	24
Maize	*Zea mays L.*	A000T	Maize grain	Netherlands	Netherlands			kernels			47±0		87±7	1270±45	175
Maize	*Zea mays L.*	A000T	Maize grain	Netherlands	Netherlands			kernels			272±7		88±16	1726±42	175
Maize	*Zea mays L.*	A000T	Maize grain	Netherlands	Netherlands			kernels			2047±75		99±20	305±4	175
Maize	*Zea mays L.*	A000T	Maize grain	Netherlands	Netherlands			kernels			401±10		101±7	2070±38	175
Maize	*Zea mays L.*	A000T	Maize grain	Croatia	Croatia						1135.3± 34.8			1183.8± 49.5	174
Rice	*Oryza sativa L. Camargue long blanc Bio, cv. Arelate*	A001D#F10. A0F2R	Rice grain, QUALITATIV E-INFO = white	France	France			seeds	white		0.6±0.1			0.2±0.1	176
Rice	*Oryza sativa L. Camargue long blanc Bio, cv. Arelate*	A001D#F10. A0F2R	Rice grain, QUALITATIV E-INFO = white	France	France	not given		seeds	white		0.6±0,1			0.2±0,1	176

Table S10.1.3 Cereal grains (and cereal-like grains) (A000L) (µg/100g) (continuation)

Food name	Scientific name	FoodEx2_TermCode	FoodEx2_TermName	Origin (country)	Purchase (country)	Water (%)	Saponification	Part analysed	Colour	E(v. trans)-zeaxanthin	Lutein	Lycopene	Z(v. cis)-β-carotene	Zeaxanthin	Ref.
Rice	*Oryza sativa L. Camargue long complet Bio, cv. Arelate*	A001E#F21.A07SE	Rice grain, brown, PRODUCTION-METHOD = Organic production	France	France			seeds	brown		7±2			1±1	176
Rice	*Oryza sativa L. Camargue long noir complet Bio cv. Artemide*	A001D#F10.A166Z	Rice grain, QUALITATIVE-INFO = Black	France	France			seeds	black		430±340	16±4		190±20	176
Rice	*Oryza sativa L. Camargue long noir complet Bio, cv. Artemide*	A001D#F10.A166Z	Rice grain, QUALITATIVE-INFO = Black	France	France			seeds	black		430±340	16±4		190±20	176
Rice	*Oryza sativa L. Camargue rouge long complet, cv. TamTam*	A001H	Rice grain, red	France	France			seeds	red		40±10			10±10	176
Tritordeum	*Tritordeum martinii A. Pujadasnotho sp.*	A001Y	Other cereals	Czech Republic	Spain			grains	yellow endosperm		1045			94	170
Tritordeum	*Tritordeum martinii A. Pujadasnotho sp. nov.*	A001Y	Other cereals	Czech Republic	Spain			grains	yellow endosperm		1045			94	170
Tritordeum	*xTritordeum Ascherson et Graebner*	A001Y	Other cereals	Spain	Spain			grains	yellow		290				177
Tritordeum	*xTritordeum Ascherson et Graebner*	A001Y	Other cereals	Spain	Spain			grains	yellow		260				177
Wheat	*Triticum aestivum L.*	A001N	Common wheat grain	Czech Republic				grains	yellow endosperm		516			117	170
Wheat	*Triticum aestivum L.*	A001N	Common wheat grain	Czech Republic	Czech Republic			grains	purple pericarp		235			510	170

Table S10.1.3 Cereal grains (and cereal-like grains) (A000L) (µg/100g) (continuation)

Food name	Scientific name	FoodEx2_ TermCode	FoodEx2_ TermName	Origin (country)	Purchase (country)	Water (%)	Saponification	Part analysed	Colour	E(v. trans)-zeaxanthin	Lutein	Lycopene	Z(v. cis)-β-carotene	Zeaxanthin	Ref.
Wheat	*Triticum aestivum L.*	A001N	Common wheat grain	Czech Republic	United States			grains	blue aleurone		139			54	170
Wheat	*Triticum aestivum L.*	A001N	Common wheat grain	Czech Republic	Czech Republic			grains	blue aleurone		552			161	170
Wheat	*Triticum aestivum L. ssp. aestivum cv. Blasco*	A001N	Common wheat grain	Italy	Italy			seeds	not given		75±1			21±0	173
Wheat	*Triticum aestivum L. ssp. aestivum cv. Bramante*	A001N	Common wheat grain	Italy	Italy			seeds	not given		145±1			16±0	173
Wheat	*Triticum aestivum L. var. Bohemia*	A001N	Common wheat grain	Czech Republic	Czech Republic			grains	red grain		243			100	170
Wheat	*Triticum aestivum L. var. Konini*	A001N	Common wheat grain	Czech Republic				grains	purple pericarp		465			87	170
Wheat	*Triticum aestivum L. var. PS Karkulka*	A001N	Common wheat grain	Czech Republic	Slovakia			grains	purple pericarp		223			68	170
Wheat	*Triticum aestivum L. var. purple*	A001N	Common wheat grain	Czech Republic				grains	purple pericarp		271			350	170
Wheat	*Triticum aestivum L. var. purple Feed*	A001N	Common wheat grain	Czech Republic				grains	purple pericarp		258			54	170
Wheat	*Triticum aestivum L. var. Tschermak's B.S.*	A001N	Common wheat grain	Czech Republic	Austria			grains	blue aleurone		228			59	170
Wheat	*Triticum aestivum L. var. Xiao Yian*	A001N	Common wheat grain	Czech Republic	China			grains	blue aleurone		134			39	170
Wheat	*Triticum aestivum L..*	A001N	Common wheat grain	Czech Republic	Czech Republic			grains	blue aleurone		203			60	170

Table S10.2.1 Cereal and cereal-like flours (A04KS) (µg/100g)

Food name	Scientific name	FoodEx2_ TermCode	FoodEx2_ TermName	Origin (country)	Purchase (country)	Water (%)	Saponification	Part analysed	Colour	α-carotene	β-carotene	β-cryptoxanthin	Antheraxanthin	Ref.
Flour		A04KS	Cereal and cereal-like flours	Italy	Italy	11		flour				1.3		179
Flour		A04KS	Cereal and cereal-like flours	Italy	Italy	11		flour				0.55		179
Maize	*Zea mays L.*	A002P#F10.A0F5H	Maize meal, QUALITATIVE-INFO = yellow	USA			no		yellow	0		46		28
Maize	*Zea mays L.*	A002P#F10.A0F2R	Maize meal, QUALITATIVE-INFO = white	USA			no		white	0		0		28
Maize	*Zea mays L.*	A002Q	Maize flour	Italy	Italy			flour		5±10	52±25	247±127		180
Maize	*Zea mays L.*	A002Q	Maize flour	Italy	Italy			flour		24±12	80±30	46±25		180
Maize	*Zea mays L.*	A002Q	Maize flour	Italy	Italy			flour		27±25	96±52	209±154		180
Maize	*Zea mays L.*	A002Q	Maize flour	Italy	Italy	-		flour		21±10	93±46	364±151		180
Maize	*Zea mays L.*	A002Q	Maize flour	Italy	Italy			flour		22±7	104±37	421±120		180
Wheat	*Triticum spp*	A003X	Wheat flour	Italy	Italy					nd	nd	nd		25
Wheat	*Triticum spp*	A004C	Wheat flour, durum	Italy	Italy					nd	nd	nd		25

Table S10.2.2 Cereal and cereal-like flours (A04KS) (µg/100g) (continuation)

Food name	Scientific name	FoodEx2_ TermCode	FoodEx2_ TermName	Origin (country)	Purchase (country)	Water (%)	Saponification	Part analysed	Colour	E(v. trans)-α-carotene	E(v. trans)-β-carotene	E(v. trans)-β-cryptoxanthin	E(v. trans)-lutein	Ref.
Maize	*Zea mays L.*	A002P#F10.A0F5H	Maize meal, QUALITATIVE-INFO = yellow	USA	USA		no		yellow		29		1	28
Maize	*Zea mays L.*	A002P#F10.A0F2R	Maize meal, QUALITATIVE-INFO = white	USA	USA		no		white		0		13	28

Table S10.2.3 Cereal and cereal-like flours (A04KS) (µg/100g) (continuation)

Food name	Scientific name	FoodEx2_ TermCode	FoodEx2_ TermName	Origin (country)	Purchase (country)	Water (%)	Saponification	Part analysed	Colour	E(v. trans)-zeaxanthin	Lutein	Lycopene	Z(v. cis)-β-carotene	Zeaxanthin	Ref.
Flour		A04KS	Cereal and cereal-like flours	Italy	Italy	11		flour	-		50 - 1000			10 - 1800	179
Flour		A04KS	Cereal and cereal-like flours	Italy	Italy	11		flour	-		20 - 650			0 - 35	179
Maize	*Zea mays*	A002P#F10. A0F5H	Maize meal, QUALITATIVE-INFO = yellow	USA	USA		no		yellow	531			0		28
Maize	*Zea mays*	A002P#F10. A0F2R	Maize meal, QUALITATIVE-INFO = white	USA	USA		no		white	13			0		28
Maize	*Zea mays L.*	A002Q	Maize flour	Italy	Italy			flour	-		713			1177	180
Maize	*Zea mays L.*	A002Q	Maize flour	Italy	Italy			flour	-		1162			361	180
Maize	*Zea mays L.*	A002Q	Maize flour	Italy	Italy			flour	-		1334			969	180
Maize	*Zea mays L.*	A002Q	Maize flour	Italy	Italy	-		flour	-		843			1422	180
Maize	*Zea mays L.*	A002Q	Maize flour	Italy	Italy			flour	-		945			1492	180
Wheat	*Triticum spp*	A003X	Wheat flour	Italy	Italy						76–116	nd			25
Wheat	*Triticum spp*	A004C	Wheat flour, durum	Italy	Italy						164	nd		nd	25

Table S10.3.1 Groats (A0BY1) (µg/100g)

Food name	Scientific name	FoodEx2_ TermCode	FoodEx2_ TermName	Origin (country)	Purchase (country)	Water (%)	Saponification	Part analysed	Colour	α-carotene	β-carotene	β-cryptoxanthin	Antheraxanthin	Ref.
Wheat	*wheat Triticum durum*	A004E	Wheat groats	France	Belgium						50			20

Table S10.4.1 Bread and similar products (A004V) (µg/100g)

Food name	Scientific name	FoodEx2_ TermCode	FoodEx2_ TermName	Origin (country)	Purchase (country)	Water (%)	Saponification	Part analysed	Colour	α-carotene	β-carotene	β-cryptoxanthin	Antheraxanthin	Ref.
Bread		A004Y	Wheat bread and rolls, white (refined flour)	USA			no		white	0		0		28

Table S10.4.2 Bread and similar products (A004V) (µg/100g) (continuation)

Food name	Scientific name	FoodEx2_ TermCode	FoodEx2_ TermName	Origin (country)	Purchase (country)	Water (%)	Saponification	Part analysed	Colour	E(v. trans)-α-carotene	E(v. trans)-β-carotene	E(v. trans)-β-cryptoxanthin	E(v. trans)-lutein	Ref.
Bread		A004Y	Wheat bread and rolls, white (refined flour)	USA			no		white		0		15	28

Table S10.4.3 Bread and similar products (A004V) (µg/100g) (continuation)

Food name	Scientific name	FoodEx2_ TermCode	FoodEx2_ TermName	Origin (country)	Purchase (country)	Water (%)	Saponification	Part analysed	Colour	E(v. trans)-zeaxanthin	Lutein	Lycopene	Z(v. cis)-β-carotene	Zeaxanthin	Ref.
Bread		A004Y	Wheat bread and rolls, white (refined flour)	USA			no		white	0			0		28

Table S10.5.1 Pasta, doughs and similar products (A04QT) (µg/100g)

Food name	Scientific name	FoodEx2_ TermCode	FoodEx2_ TermName	Origin (country)	Purchase (country)	Water (%)	Saponification	Part analysed	Colour	α-carotene	β-carotene	β-cryptoxanthin	Antheraxanthin	Ref.
Egg pasta		A007M	Dried egg pasta	Italy	Italy			dried pasta		The sum of alpha and beta-carotene - 14.0±0.9		18.4±2.9		50
Spinach noodles		A04LF#F28.A0BA1$F04.A00MJ	Pasta-like products, PROCESS = Cooking and similar thermal preparation processes, INGREDIENT = Spinaches	USA			no			0		0		28

Table S10.5.2 Pasta, doughs and similar products (A04QT) (µg/100g) (continuation)

Food name	Scientific name	FoodEx2_ TermCode	FoodEx2_ TermName	Origin (country)	Purchase (country)	Water (%)	Saponification	Part analysed	Colour	E(v. trans)-α-carotene	E(v. trans)-β-carotene	E(v. trans)-β-cryptoxanthin	E(v. trans)-lutein	Ref.
Spinach noodles		A04LF#F28.A0BA1$F04.A00MJ	Pasta-like products, PROCESS = Cooking and similar thermal preparation processes, INGREDIENT = Spinaches	USA			no				21		176	28

Table S10.5.3 Pasta, doughs and similar products (A04QT) (µg/100g) (continuation)

Food name	Scientific name	FoodEx2_TermCode	FoodEx2_TermName	Origin (country)	Purchase (country)	Water (%)	Saponification	Part analysed	Colour	E(v. trans)-zeaxanthin	Lutein	Lycopene	Z(v. cis)-β-carotene	Zeaxanthin	Ref.
Egg pasta		A007M	Dried egg pasta	Italy	Italy			dried pasta			656.5± 20.4			160.9± 0.3	50
Spinach noodles		A04LF#F28.A0BA1$F04.A00MJ	Pasta-like products, PROCESS = Cooking and similar thermal preparation processes, INGREDIENT = Spinaches	USA			no			0			0		28

Table S10.6.1 Fine bakery wares (A009T) (µg/100g)

Food name	Scientific name	FoodEx2_TermCode	FoodEx2_TermName	Origin (country)	Purchase (country)	Water (%)	Saponification	Part analysed	Colour	α-carotene	β-carotene	β-cryptoxanthin	Antheraxanthin	Ref.
Muffin		A00BC#F04.A002Q	Muffins, INGREDIENT = Maize flour	USA			no			0		8		28

Table S10.6.2 Fine bakery wares (A009T) (µg/100g) (continuation)

Food name	Scientific name	FoodEx2_TermCode	FoodEx2_TermName	Origin (country)	Purchase (country)	Water (%)	Saponification	Part analysed	Colour	E(v. trans)-α-carotene	E(v. trans)-β-carotene	E(v. trans)-β-cryptoxanthin	E(v. trans)-lutein	Ref.
Muffin		A00BC#F04.A002Q	Muffins, INGREDIENT = Maize flour	USA	USA		no				0		86	28

Table S10.6.3 Fine bakery wares (A009T) (µg/100g) (continuation)

Food name	Scientific name	FoodEx2_ TermCode	FoodEx2_ TermName	Origin (country)	Purchase (country)	Water (%)	Saponification	Part analysed	Colour	E(v. trans)-zeaxanthin	Lutein	Lycopene	Z(v. cis)-β-carotene	Zeaxanthin	Ref.
Muffin		A00BC#F04. A002Q	Muffins, INGREDIENT = Maize flour	USA			no			51			0		28

Table S10.7.1 Breakfast cereals (A00CV) (µg/100g)

Food name	Scientific name	FoodEx2_ TermCode	FoodEx2_ TermName	Origin (country)	Purchase (country)	Water (%)	Saponification	Part analysed	Colour	α-carotene	β-carotene	β-cryptoxanthin	Antheraxanthin	Ref.
Corn Cereals – “Cap'n Crunch”	*Zea mays L:*	A00EM	Processed mixed cereal-based flakes	USA			no			0		3		28
Corn Cereals “Frosted Flakes”	*Zea mays L.*	A00DD	Processed maize-based flakes	USA			no			2		9		28
Corn Cereals “Apple Jacks”	*Zea mays L.*	A00DD	Processed maize-based flakes	USA			no			0		5		28
Corn Cereals “Corn Chex”	*Zea mays L.*	A00DD	Processed maize-based flakes	USA			no			2		15		28
Corn Cereals “Corn pops”	*Zea mays L.*	A00DD	Processed maize-based flakes	USA			no			0		0		28
Corn Cereals “Life”	*Zea mays L.*	A00DD	Processed maize-based flakes	USA			no			0		3		28
Corn Cereals “Reese's Puffs”	*Zea mays L:*	A00DD#F04.A06SD	Processed maize-based flakes, INGREDIENT = Peanut butter flavour	USA			no			0		5		28

Table S10.7.1 Breakfast cereals (A00CV) (µg/100g) (continuation)

Food name	Scientific name	FoodEx2_ TermCode	FoodEx2_ TermName	Origin (country)	Purchase (country)	Water (%)	Saponification	Part analysed	Colour	α-carotene	β-carotene	β-cryptoxanthin	Antheraxanthin	Ref.
Corn Cereals Fruit Loops”	*Zea mays L.*	A00DD	Processed maize-based flakes	USA			no			0		4		28
Corn flakes	*Zea mays L.*	A00DD	Processed maize-based flakes	USA			no			0		11		28
Corn flakes	*Zea mays L.*	A00DD	Processed maize-based flakes	Italy	Italy					nd	nd	nd		25
Popcorn	*Zea mays L.*	A00DC	Popcorn (maize, popped)	USA			no			0		24		28
Puffed einkorn	*Triticum monococcum L. subsp. monococcum Monarca*	A04LL#F27.A001S	Popped cereals, SOURCE-COMMODITIES = Einkorn wheat grain	Italy	Italy	5.2		puffed kernels	L*a*b* values 52/10.6/22.5		29 (sum of α- and β-carotene)±0	63±0		173
Puffed einkorn	*Triticum monococcum L. subsp. monococcum Monlis*	A04LL#F27.A001S	Popped cereals, SOURCE-COMMODITIES = Einkorn wheat grain	Italy	Italy	5.2		puffed kernels	L*a*b* values 48.6/10.5/20.4		64 (sum of α- and β-carotene)±1	120±1		173
Puffed wheat	*Triticum aestivum L. ssp. aestivum cv. Blasco*	A00EE	Wheat, popped	Italy	Italy	5		puffed kernels	L*a*b* values 58.7/9.4/20.1		7 (sum of α- and β-carotene)±0	12±0		173
Puffed wheat	*Triticum aestivum L. ssp. aestivum cv. Bramante*	A00EE	Wheat, popped	Italy	Italy	5		puffed kernels	L*a*b* values 60.6/7.3/16.7		14 (sum of α- and β-carotene)±1	20±0		173

Table S10.7.2 Breakfast cereals (A00CV) (µg/100g) (continuation)

Food name	Scientific name	FoodEx2_ TermCode	FoodEx2_ TermName	Origin (country)	Purchase (country)	Water (%)	Saponification	Part analysed	Colour	E(v. trans)-α-carotene	E(v. trans)-β-carotene	E(v. trans)-β-cryptoxanthin	E(v. trans)-lutein	Ref.
Corn Cereals “Apple Jacks”	*Zea mays L.*	A00DD	Processed maize-based flakes	USA			no				0		43	28
Corn Cereals “Cap'n Crunch”	*Zea mays L.*	A00EM	Processed mixed cereal-based flakes	USA			no				0		42	28
Corn Cereals “Corn Chex”	*Zea mays L.*	A00DD	Processed maize-based flakes	USA			no				7		151	28
Corn flakes	*Zea mays L.*	A00DD	Processed maize-based flakes	USA			no						40	28
Corn Cereals “Corn pops”	*Zea mays L.*	A00DD	Processed maize-based flakes	USA			no				3		42	28
Corn Cereals “Frosted Flakes”	*Zea mays L.*	A00DD	Processed maize-based flakes	USA			no				4		33	28
Corn Cereals “Fruit Loops”	*Zea mays L.*	A00DD	Processed maize-based flakes	USA			no				0		41	28
Corn Cereals “Life”	*Zea mays L.*	A00DD	Processed maize-based flakes	USA			no				5		51	28
Corn Cereals “Reese's Puffs”	*Zea mays L.*	A00DD#F04.A06SD	Processed maize-based flakes, INGREDIENT = Peanut butter flavour	USA			no				0		46	28
Popcorn	*Zea mays L.*	A00DC	Popcorn (maize, popped)	USA			no				17		64	28

Table S10.7.3 Breakfast cereals (A00CV) (µg/100g) (continuation)

Food name	Scientific name	FoodEx2_ TermCode	FoodEx2_ TermName	Origin (country)	Purchase (country)	Water (%)	Saponification	Part analysed	Colour	E(v. trans)-zeaxanthin	Lutein	Lycopene	Z(v. cis)-β-carotene	Zeaxanthin	Ref.
Cornflakes	*Zea mays L.*	A00DD	Processed maize-based flakes	Italy	Italy						nd – 52	nd		102–297	25
Puffed wheat	*Triticum aestivum L. ssp. aestivum cv 'Bramante'*	A00EE	Wheat, popped	Italy	Italy	5		puffed kernels	L*a*b* values 60.6/7.3/16.7		49±5			8±0	173
Corn Cereals “Frosted Flakes”	*Zea mays L.*	A00DD	Processed maize-based flakes	USA			no			81			6		28
Corn Cereals “Apple Jacks”	*Zea mays L.*	A00DD	Processed maize-based flakes	USA			no			24			0		28
Corn Cereals “Cap'n Crunch”	*Zea mays L.*	A00EM	Processed mixed cereal-based flakes	USA			no			20			0		28
Corn Cereals “Corn Chex”	*Zea mays L.*	A00DD	Processed maize-based flakes	USA			no			115			5		28
Corn Cereals “Corn flakes”	*Zea mays L.*	A00DD	Processed maize-based flakes	USA			no			49			4		28
Corn Cereals “Corn pops”	*Zea mays L.*	A00DD	Processed maize-based flakes	USA			no			36			0		28
Corn Cereals “Fruit Loops”	*Zea mays L.*	A00DD	Processed maize-based flakes	USA			no			24			0		28
Corn Cereals “Life”	*Zea mays L.*	A00DD	Processed maize-based flakes	USA			no			25			0		28
Corn Cereals “Reese's Puffs”	*Zea mays L.*	A00DD#F04. A06SD	Processed maize-based flakes, INGREDIENT = Peanut butter flavour	USA			no			34			0		28

Table S10.7.3 Breakfast cereals (A00CV) (µg/100g) (continuation)

Food name	Scientific name	FoodEx2_ TermCode	FoodEx2_ TermName	Origin (country)	Purchase (country)	Water (%)	Saponification	Part analysed	Colour	E(v. trans)-zeaxanthin	Lutein	Lycopene	Z(v. cis)-β-carotene	Zeaxanthin	Ref.
Popcorn	*Zea mays L.*	A00DC	Popcorn (maize, popped)	USA			no			141			0		28
Puffed einkorn	*Triticum monococcum L. subsp. monococcum 'Monarca'*	A04LL#F27.A001S	Popped cereals, SOURCE-COMMODITIES = Einkorn wheat grain	Italy	Italy	5.2		puffed kernels	L*a*b* values 52/10.6/22.5		86±2			18±1	173
Puffed einkorn	*Triticum monococcum L. subsp. monococcum 'Monlis'*	A04LL#F27.A001S	Popped cereals, SOURCE-COMMODITIES = Einkorn wheat grain	Italy	Italy	5.2		puffed kernels	L*a*b* values 48.6/10.5/20.4		95±4			20±0	173
Puffed wheat	*Triticum aestivum L. ssp. aestivum cv 'Blasco'*	A00EE	Wheat, popped	Italy	Italy	5		puffed kernels	L*a*b* values 58.7/9.4/20.1		14±1			9±0	173
